# Boron-Enabled Stereoselective Synthesis of Polysubstituted Housanes

**DOI:** 10.1021/jacs.5c17624

**Published:** 2025-12-11

**Authors:** Hao Fang, Aimara García-Camacho, Ho Seong Hwang, Atthawut Sudsamart, Constantin G. Daniliuc, Oleksandr O. Grygorenko, Ignacio Funes-Ardoiz, John J. Molloy

**Affiliations:** † Department of Biomolecular Systems, 28321 Max-Planck-Institute of Colloids and Interfaces, 14476 Potsdam, Germany; ‡ Department of Chemistry and Biochemistry, Freie Universität Berlin, 14195 Berlin, Germany; § Department of Chemistry, Instituto de Investigación Química de la Universidad de La Rioja (IQUR), 16764 Universidad de La Rioja, Madre de Dios 53, 26006 Logroño, Spain; ∥ Organisch-Chemisches Institut, 9185 Universität Münster, 48419 Münster, Germany; ⊥ Enamine Ltd., Winston Churchill Street 78, 02094 Kyiv, Ukraine; # Taras Shevchenko National University of Kyiv, Volodymyrska Street 60, 01601 Kyiv, Ukraine

## Abstract

The efficient design of (C)­sp^3^-rich molecular scaffolds with defined exit vectors is central to expanding drug-like chemical space. Here, we report a boron-enabled strategy for the synthesis of polysubstituted housanes from nonsymmetrical dienes. A *geminal* diboron system ensures site-, regio-, and diastereoselectivity in an energy transfer-catalyzed [2 + 2] cycloaddition of nonsymmetrical dienes while also facilitating the mild generation of a cyclobutyl anion that triggers a stereospecific intramolecular annulation via conjugate addition, delivering complex housanes, with three defined exit vectors, in just two steps. Systematic derivatization across all substituents demonstrates the breadth of chemical diversification, while mechanistic and density functional theory (DFT) computational studies reveal the stereoelectronic origins of diastereoselectivity and the counterintuitive electrophile-driven reactivity of the housane framework. This work establishes housanes as stable, derivatizable, and structurally rigid fragments that provide multidirectional exit vectors, offering a powerful platform for the exploration of three-dimensional (3D) chemical space in medicinal chemistry.

## Graphical abstract

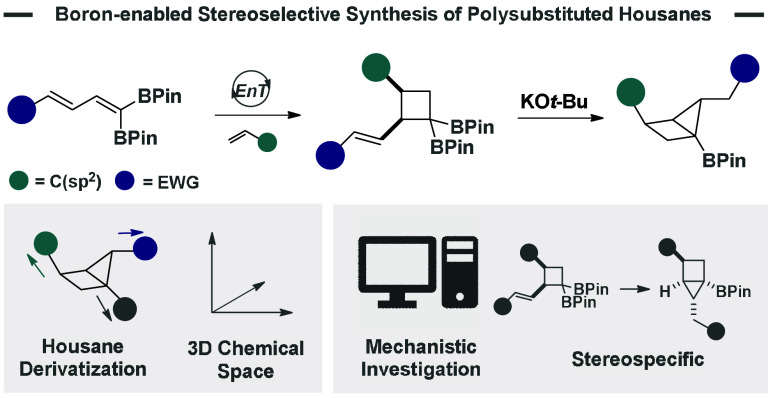

## Introduction

The design and synthesis of molecules that occupy a defined chemical space is pivotal for optimizing biomolecular interactions, thereby facilitating the discovery of next-generation therapeutics.^1^ While the theoretical expanse of chemical space is almost infinite,^2^ synthetic chemists are constrained by the available methodologies capable of positioning functional groups in a defined spatial orientation. Many strategies are used to great effect to strategically position exit vectors to target specific biomolecular interactions. For example, the bidirectional substitution of aromatic rings is exemplar and has been employed to explore two-dimensional (2D) chemical space with ease (Figure 1A).^3^ However, recently, the intricate three-dimensional (3D) topology of target biomolecules has driven a paradigm shift toward sp^3^-rich systems that emulate bidirectional aromatic analogues.^4^ Elegant strategies have enabled access to cubanes^5^ and ladderanes,^6^ while related systems, bicyclopentanes,^7^ hexanes,^8^ heptanes,^9^ and other analogues,^10^ are also designed to preserve analogous spatial trajectories while modulating physicochemical properties. Although “escape from flatland”^11^ and “return to flatland”^12^ are still hotly debated for clinical success, *de novo* strategies to small fragments that contain unique exit vectors are irrefutably required to augment future drug design.

**Figure 1:**
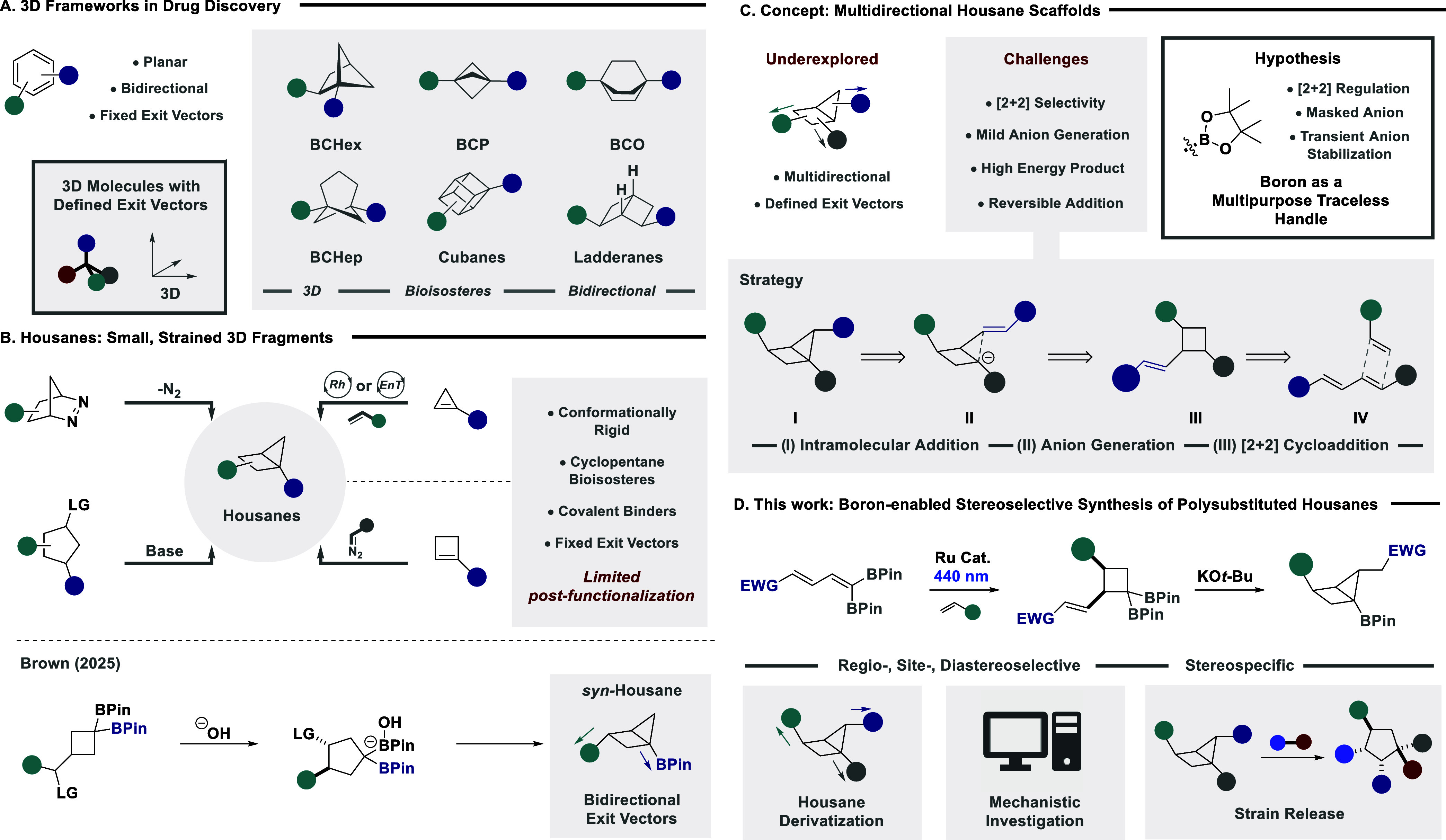
(A) Exploration of 2D and 3D chemical space in drug discovery. (B) Strategies for the synthesis of housane scaffolds. (C) Conceptual approach to polysubstituted housanes. (D) Boron-enabled stereoselective synthesis of polysubstituted housanes.

Housanes are small fused bicyclic systems, where their conformational rigidity and fixed exit vectors allow them to mimic cyclopentanes (Figure 1B),^13^ key frameworks in contemporary medicinal chemistry.^14^ While classic approaches for their synthesis have focused on nitrogen extrusion^15^ and carbene addition across cyclobutenes,^16^ prominent strategies have also emerged leveraging energy transfer (EnT)^17,18^ and transition metal catalysis^19^ of cyclopropenes, and transannular nucleophilic substitution.^13c,20^ However, strategies that enable facile postfunctionalization are conspicuously underexplored yet desirable, given the unique exit vector properties of the housane framework. In 2025, Brown and co-workers described a prominent advance, accessing borylated housanes, via a unique boron-driven ring opening, and transannular ring-closing strategy, providing *syn*-exit vectors for the subsequent exploration of chemical space.^21^

The expansion to 3D fragments that contain multidirectional exit vectors is pivotal in drug design to maximize chemical space exploration while eliciting additional biomolecular interactions.^22^ We anticipated that the inception of such a strategy for housane scaffolds would expedite their uptake in medicinal chemistry programs (Figure 1C). Retrosynthetic analysis of high-energy trisubstituted housane **I** revealed an ambitious, novel exocyclic annulation via a conjugate addition reaction from **II**. It was envisaged that anion **II** could be generated from the parent cyclobutane **III**, which could be accessed via a potential site- and regioselective [2 + 2] cycloaddition of **IV**.^23^ To facilitate this strategy, intractable challenges must be overcome, such as (1) establishing site- and regioselectivity in the EnT-enabled [2 + 2] cycloaddition of dienes with ostensibly nonequivalent termini; (2) the mild generation of a cyclobutyl anion; and (3) achieving reaction directionality to generate a strained housane system via an intramolecular conjugate addition. To circumnavigate these obstacles, it was anticipated that boron could serve as a traceless multipurpose handle that can be leveraged to fine-tune selectivity in [2 + 2] cycloaddition,^24,25^ and provide a handle for mild anion generation in our conceptual strategy.^26^

Herein, we describe the boron-enabled synthesis of polysubstituted housanes from nonsymmetrical dienes (Figure 1D). The judicious choice of a *geminal* diboron system enables complete site selectivity and regioselectivity in a challenging EnT-catalyzed intermolecular [2 + 2] cycloaddition. The mild base-mediated activation of the *gem*-diboron cyclobutane intermediate triggers a stereospecific intramolecular conjugate addition reaction to furnish complex housanes with three defined exit vectors. Systematic chemoselective derivatization of all housane substituents demonstrates the power of the method for the efficient exploration of the 3D chemical space using housane scaffolds. In-depth mechanistic studies reveal key insights into the stereospecific nature of the ring-closing conjugate addition, while strain release evaluation unveils counterintuitive reactivity with electrophiles, in stark contrast to current state of the art showcasing favorable reactivity of housanes with nucleophiles.^13c,20b^

## Results and Discussion

The targeted synthesis of housanes requires the initial inception of a selective intermolecular [2 + 2] cycloaddition on diene frameworks, retaining a Michael acceptor in the core scaffold for future conjugate addition. Our group has previously established selective intermolecular [2 + 2] cycloaddition of dienes via EnT catalysis;^24^ however, reactivity/selectivity was heavily contingent on the core scaffold containing a phenyl substituent, to differentiate both termini. Cognizant of the electronic influence that functional groups can impart on the site- and regioselectivity for EnT-catalyzed [2 + 2] cycloaddition, we commenced our study probing boron substituent effects of δ-boryl acrylates (Scheme 1). Boronates of varying electron density on the δ-carbon (**1**–**4**)^27^ were exposed to a low excited state energy ruthenium photocatalyst in the presence of styrene.^28^ The use of a *geminal* system, previously employed in [2 + 2] cycloaddition by Masarwa and co-workers,^25f^ and our own research group,^24^ afforded the target cyclobutane in good yield, favoring *syn*-diastereoselectivity (**1**). However, the use of alternative boron moieties (**2**–**4**) led to a complex mixture of products, including dimers and isomers resulting from poor site-, regio-, and diastereoselectivity and geometrical isomerization.^29^ These results indicate that while standard borylated dienes (BPin, BMIDA, and BF_3_K) can be efficiently excited via EnT, these groups are not electronically biased in comparison to acrylates to favor an initial addition from a specific terminus of the diene. It is anticipated that the *geminal* diboron system is efficiently stereoelectronically tuned from the additional boron substituent to mitigate dimerization and promote reactivity at this site upon excitation, leading to comprehensive site- and regioselectivity.

**Scheme 1:**
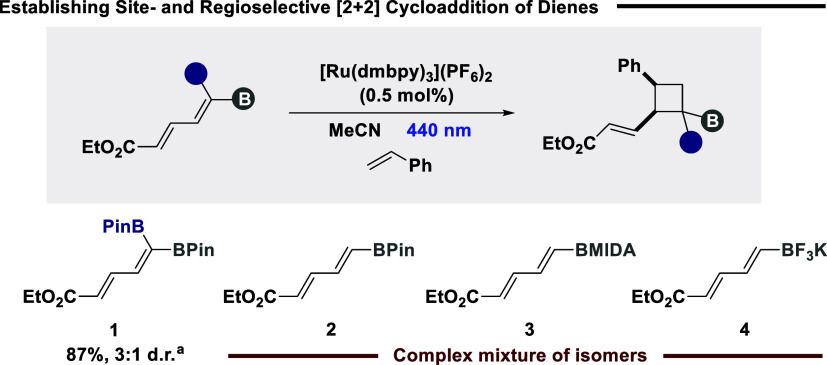
Probing Boron Substituent Influence on Selective [2 + 2] Cycloaddition^a^

a Determined by ^1^H NMR spectroscopy against a known internal standard (1,3,5-trimethoxybenzene).

With efficient site- and regioselective [2 + 2] cycloaddition established via EnT catalysis, the scope and compatibility of the protocol were explored for a series of borylated dienes and alkene coupling partners (Scheme 2). Pleasingly, the protocol was amenable to a series of styrenes, including electron-neutral (**5** and **6**), electron-rich (**7** and **8**), and electron-deficient (**9**–**12**) examples, providing the target cyclobutanes with complete site-, and regioselectivity and favorable *syn*-diastereoselectivity. Incorporating substituents in the *ortho*, *meta*, or *para* position had little to no effect on the target reactivity, while the use of bromides was also effective to provide a synthetic handle for further diversification (**10** and **11**). The use of heterocycles was tolerated, as demonstrated by both benzofuran (**13**) and indole (**14**) examples, while simple butadiene was also applicable to provide an additional pendent alkene functionality (**15**). A styrene derived from the therapeutic, fenofibrate (**16**), could also be employed, demonstrating the potential to incorporate bioactive fragments in the developed method. It is pertinent to note that deviation from styrene and diene coupling partners resulted in little to no product formation due to either limited reactivity or poor regioselectivity (see Supporting Information (SI) for full details on unsuccessful substrates). Modification of the core diene was tolerated with various carbonyl derivatives such as esters (**17**–**20**), thioesters (**21**), and amides (**22**–**24**), providing the target cycloadducts with complete site selectivity and regioselectivity. The use of a BPin moiety was also effective (**25**), while expansion of the scope to therapeutic derivatives from paliperidone (**26**), gemfibrozil (**27**), and vortioxetine (**28**) could also be successfully achieved under model reaction conditions. While nitrile and nitro groups were targeted as potential potent Michael acceptors, synthesis of the parent diene was unsuccessful (see SI for the full details).

**Scheme 2:**
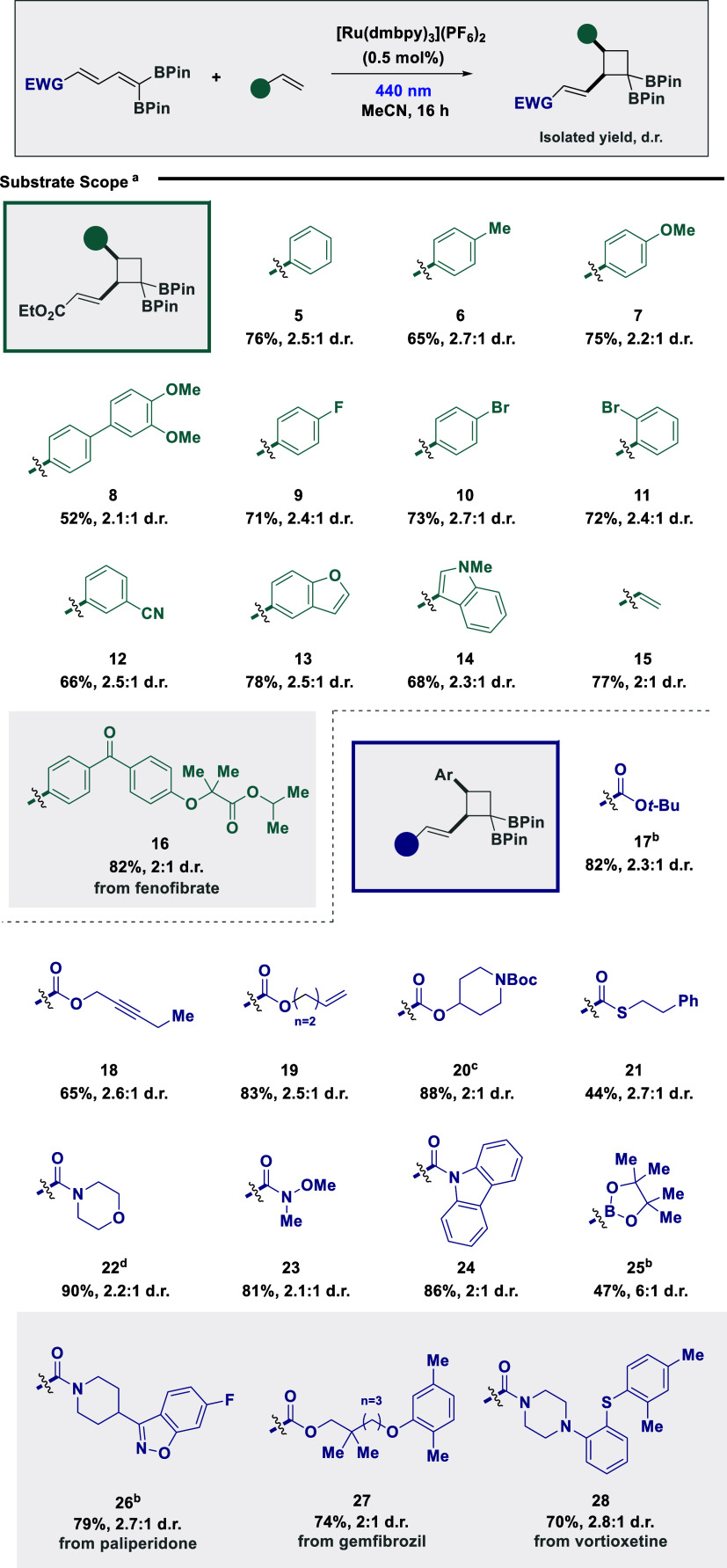
Establishing the Substrate Scope of Intermolecular [2 + 2] Cycloaddition^a^

a Reactions were performed in MeCN on a 0.2 mmol scale using diene (1 equiv), alkene (5 equiv), photocat (0.5 mol %) under 440 nm irradiation (10 W) for 16h. d.r. was determined by ^1^H NMR spectroscopy. ^*b*^Ar = 4-methoxy. ^*c*^Ar = 4-phenyl. ^*d*^Reaction was run for 48 h.

The strategic use of *geminal* diboron systems to mildly generate α-boryl anions when exposed to a strong base has been used to great effect.^26^ With expedient access to cyclobutanes containing a pendent Michael acceptor and a spatially proximal *gem*-diboron functionality established, we next sought to probe the efficiency of the base-mediated, ring-closing housane formation (Table 1). Exposure of the major *syn*-isomer **5**_**syn**_ to potassium tert-butoxide resulted in the formation of a single housane diastereoisomer after 30 min (Entry 1), containing three fixed exit vector handles. This result demonstrates that ring closing is unexpectedly achieved in a highly diastereoselective manner, forming a complex housane after two synthetic steps. The increase in base equivalents was detrimental, leading to the formation of **29**′ via transesterification (Entries 2 and 3). While the use of THF led to slightly lower yields (Entry 4), reactions in MeCN (Entry 5) were comparable to toluene. Alternative bases were ineffective in toluene, presumably due to sparing solubility (Entries 6 and 7). Translation to DMSO as media led to comparable poor results using CsF (Entry 8), while KOH produced the target housane in 52% yield (Entry 9).

**Table 1:**
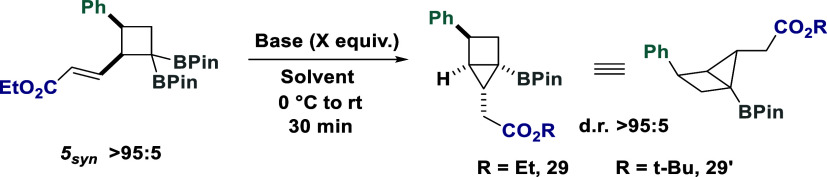
Optimization of Reaction Conditions^a^

entry	base	equiv.	**solvent**	**29** (%)^b^	**29′** (%)^b^
**1**	**KO** ***t-*** **Bu**	**1.5**	**toluene**	**79**	**<5**
2	KO*t-*Bu	1.8	toluene	71	10
3	KO*t-*Bu	2	toluene	65	23
4	KO*t-*Bu	1.5	THF	65	<5
5	KO*t-*Bu	1.5	MeCN	77	<5
6	CsF	1.5	toluene	<5	<5
7	KOH	1.5	toluene	<5	<5
8^c^	CsF	1.5	DMSO	<5	<5
9^c^	KOH	1.5	DMSO	52	<5

a Standard conditions: **5**_**syn**_ (0.1 mmol), base (*x* equiv), solvent (0.05 M), 0 °C to rt, 30 min. KO*t*-Bu was added as a 1 M solution in THF.

b Determined by ^1^H NMR spectroscopy against a known internal standard (1,3,5-trimethoxybenzene).

c Reactions were run for 2 h.

Having established conditions for the stereoselective synthesis of housanes under the auspices of base activation, we next set out to establish the scope and limitations of the reaction (Scheme 3). Modifications of the aryl substituent were tolerated, enabling housane formation for electron-neutral (**29** and **30**), electron-rich (**31** and **32**), and electron-poor (**33**–**36**) examples. The incorporation of heterocycles (**37** and **38**) and a pendent alkene (**39**) was possible under model reaction conditions. Variation of the carbonyl substituent could be achieved with both esters (**40** to **42**) and amides tolerated (**43** and **44**). It is pertinent to note that substrate **43** was formed due to concomitant N–O bond cleavage of the parent Weinreb amide in the presence of potassium tert-butoxide.^30^ Housanes containing therapeutic scaffolds could be easily accessed using the developed protocol for forging derivatives of paliperidone (**45**), gemfibrozil (**46**), vortioxetine (**47**), and fenofibrate (**48**). Pleasingly, the absolute configuration of the single diastereomer (**35**) could be confirmed by X-ray crystallography. The use of carbonyl derivatives that are comparatively more electrophilic was unsuccessful due to competing transesterification of *tert*-butoxide (Scheme 2, **18** and **21**), while BPin substrate **25** led to no reactivity, presumably due to an additional boron reservoir for tert-butoxide complexation.

**Scheme 3:**
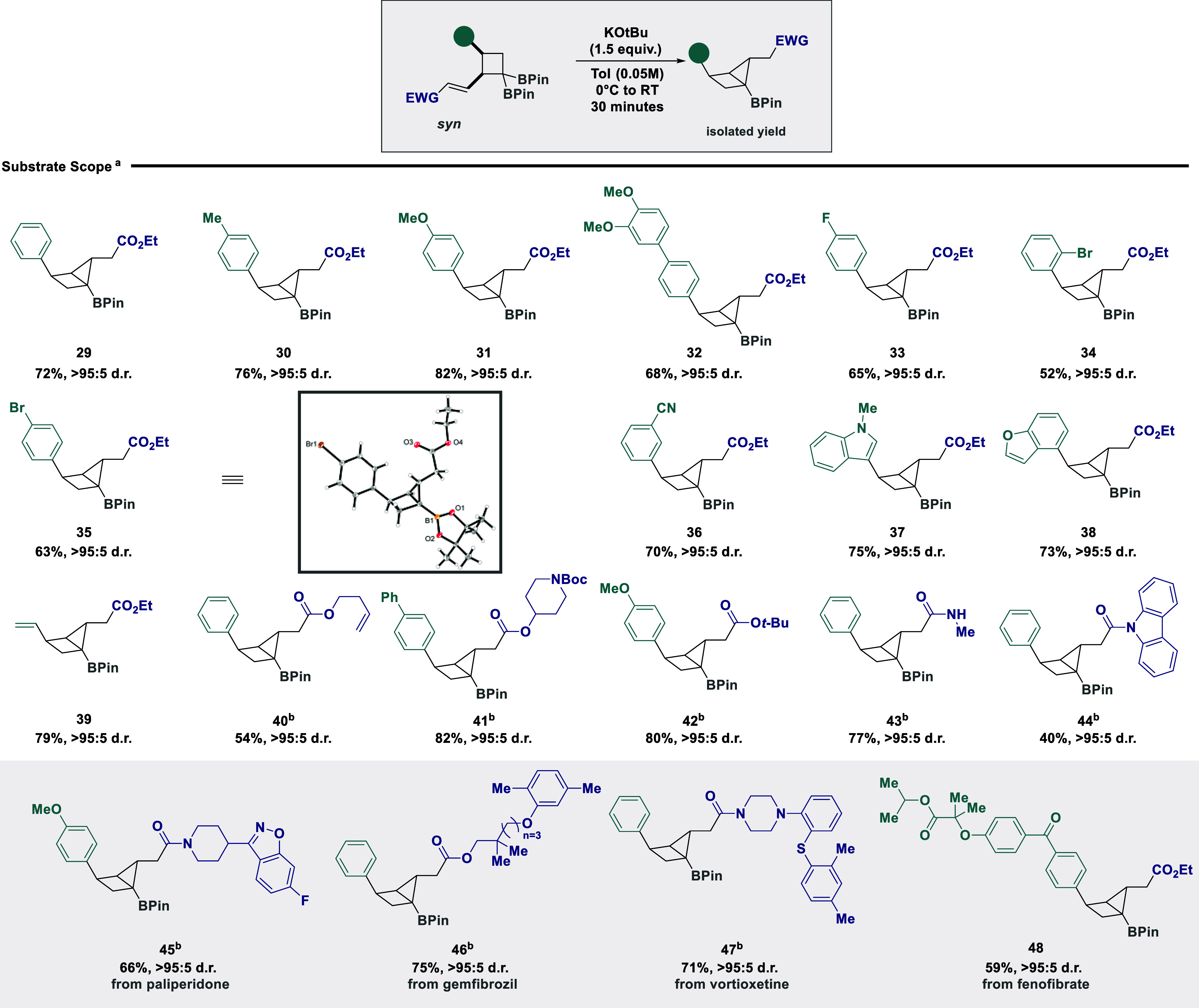
Establishing the Substrate Scope of Housane Formation^a^

a Reactions were performed in toluene using *gem*-diboron cyclobutane (1 equiv), KO*t*-Bu (1.5 equiv, 1 M solution in THF), 0 °C to rt, 30 min. d.r. was determined by ^1^H NMR spectroscopy. ^*b*^Reaction was performed using KO*t*-Bu (2 equiv) for 1 h.

To evaluate the unique diastereoselectivity observed in housane formation experimentally, a computational investigation was initiated. In particular, we studied the mechanism for formation of housane **29** (Figure 2A), in relation to base initiation (using tert-butoxide anion as base) and the subsequent ring closure, through density functional theory (DFT) calculations at the SMD (MeCN) ωB97xD/Def2TZVPP//ωB97xD/Def2SVP level of theory,^31^ including quasi-harmonic corrections with the Goodvibes program.^32^ The mechanism is initiated with the tert-butoxide anion attack of a BPin moiety. The most favorable attack is on the BPin unit that is least sterically encumbered, the BPin that is *anti* to both phenyl and ester groups (Δ*G*‡**TS1** = 8.6 kcal/mol), forming a more stable intermediate (**int1**, −13.8 kcal/mol). Subsequent deborylation occurs (Δ*G*‡**TS2** = 16.5 kcal/mol) to form a carbanion intermediate (**int2**, −13.6 kcal/mol). Despite the anionic character of the resulting carbon, the strong interaction between the anion and the boron p-orbital forms a stabilized borata-alkene-like structure supporting the extrusion of tBuOBPin (trans–C–C–B angle of 171°),^33^ enabling efficient attack of the intramolecular acrylate unit from the top face. **Int2** with the negative charge *syn* to the Michael acceptor enables cyclization through a free energy barrier of 1.3 kcal/mol. Since the carbanion species (**int2**) is a rotamer with respect to its acrylate chain, there are two possible pathways for ring closure: with the acrylate chain *anti* or *syn* in relation to the phenyl substituent (see Figure 2B, left). The most stable pathway is the one involving the *anti*-phenyl isomer (we refer to this as “*anti*-isomer”). This species undergoes ring closure faster than the other “syn-isomer” (ΔΔ*G*‡ = 1.4 kcal/mol). Moreover, the obtained enolate (**29**enolate, −19.7 kcal/mol) rapidly evolves to form an even more stable 6-membered ring (**29**_**B–O**_, −27.4 kcal/mol) due to a facile interaction between the enolate and the B atom of the pinacolboronic ester unit, making this step irreversible and both thermodynamically and kinetically favored. The energetic difference between the two isomers for ring closure is due to the steric hindrance between the phenyl and acrylic residues that are present in the *syn*-isomer and are not present in the *anti*-isomer. These considerations provide a predicted ratio of 90:10 at 0 °C, in close agreement with the experimental results, with the result being that housane **29**, derived from the *anti*-isomer carbanion, **int2**, is the only diastereoisomer observed. In order to support the key role of phenyl group orientation in modulating the stereospecificity of **5**_**syn**_, we probed reactivity with the corresponding *anti* starting material (5_anti_). In this case, *anti* and *syn*-isomers formed after deborylation are found to be very close in energy (0.7 kcal/mol), and there is almost no difference in energetic barriers for ring closure (0.3 kcal/mol) (see Figure 2B, right). This is intuitive, as there is no destabilizing steric clash between phenyl and acrylic chains in either isomer due to the reverse configuration of the phenyl moiety. It also agrees with the experimental results, as both diastereoisomers (**29a** and **29b**) are observed using **5**_**anti**_ in the housane formation reaction. Therefore, it should be noted that the steric hindrance posed by the phenyl group and the ester group is crucial for diastereoselectivity. Use of a crude starting material from [2 + 2] cycloaddition (mixture of **5**_**syn**_ and **5**_**ant**i_) could also be successfully used in housane formation, strongly favoring formation of the main product **29**, which could be easily isolated as a single stereoisomer (see the Supporting Information for full details).

**Figure 2:**
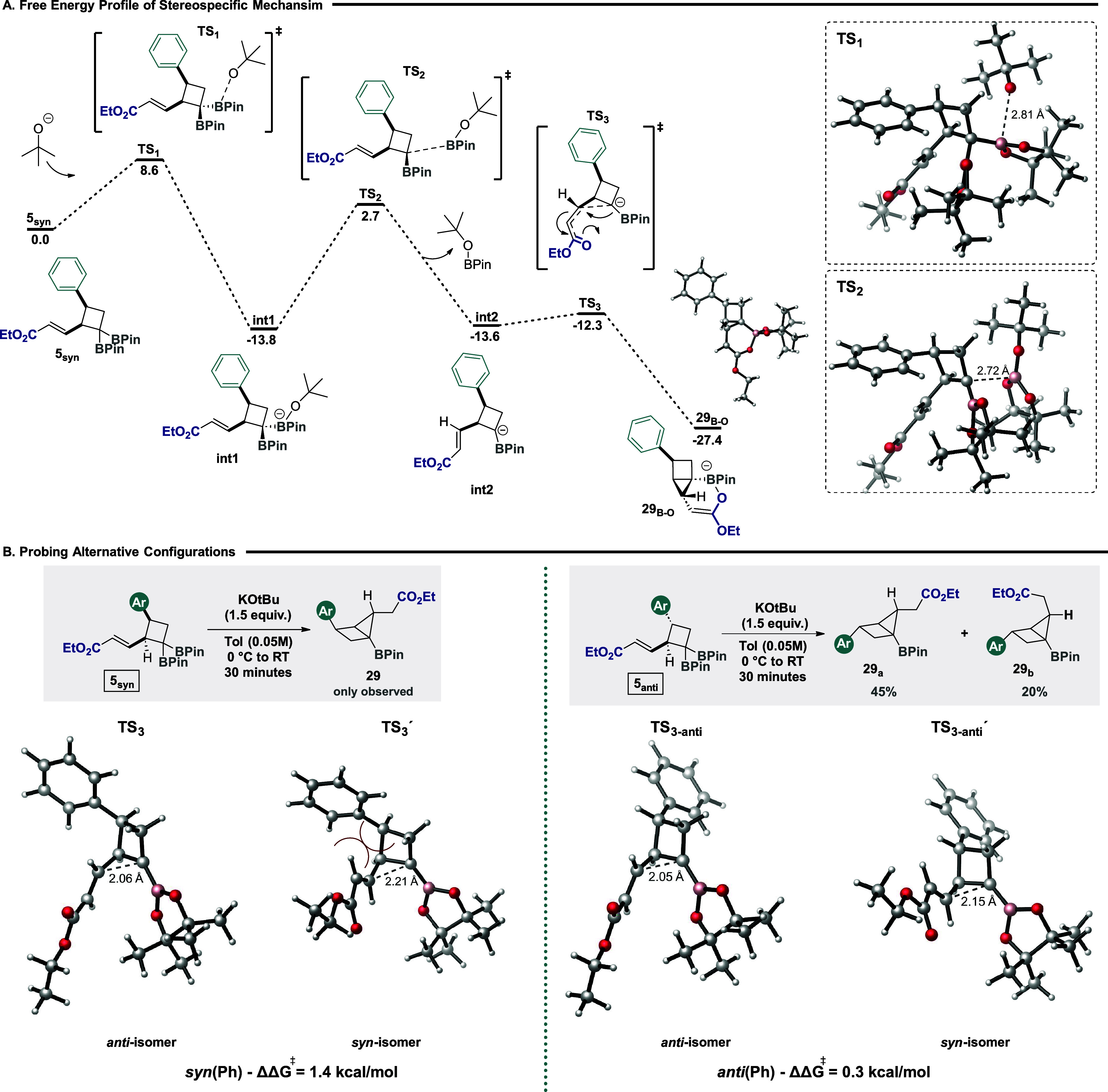
(A) Free energy profile of the stereospecific mechanism of housane formation at the ωB97xD/Def2TZVPP//ωB97xD/Def2SVP SMD­(MeCN) level of theory. Free energies are in kcal/mol. (B) Structures and energies of the possible configurations for the ring closure transition states for cycloadduct **5**_**syn**_ (left) and cycloadduct **5**_**anti**_ (right). Free energies in kcal/mol.

As the overarching vision of this study was to provide a platform to easily derivatize polysubstituted housanes, we next sought to systematically explore each exit vector as a potential site of further functionalization (Figure 3A). First, targeting the ester component (Figure 3A, blue), the facile reduction using lithium aluminum hydride could be achieved to access hemiboronic acid **49**, a potent functionality in drug discovery.^34^ The mild hydrolysis of the ester component was tolerated to furnish a carboxylic acid handle that was primed for further coupling strategies (**50**). Given the relative ease with which the C–B bond can be interconverted to alternative functionalities, it was next targeted for subsequent synthetic manipulations (Figure 3A, gray).^35^ Conversion to trifluoroborate **51** provided an intermediate that could undergo controlled protodeboronation (**52**),^36^ Chan-Lam coupling (**53**),^37^ and Suzuki-Miyaura cross-coupling (**54**).^38^ Despite the established instability of housanes to strong nucleophiles,^13c,20b^ Matteson homologation was successful to form alcohol **55**, which could be subsequently oxidized to the corresponding carboxylic acid **56**. Oxidation of the boronic ester resulted in a concomitant ring opening to provide a substituted cyclopentanone as a single diastereoisomer (**57**). Selective alkene activation of substrate **39** followed, enabling a facile hydroboration to form bis-boron product **58**. Intriguingly, the generation and use of radicals were well tolerated, with no observed ring opening of the housane structure (**59**). This result provides direct experimental evidence that the housane structures generated in this study are stable to open-shell intermediates, which supports elegant computation studies by Duarte, Anderson, and co-workers.^39^ It is pertinent to note that throughout all derivatization reactions, the diastereomeric purity of the housane structure was retained.

**Figure 3:**
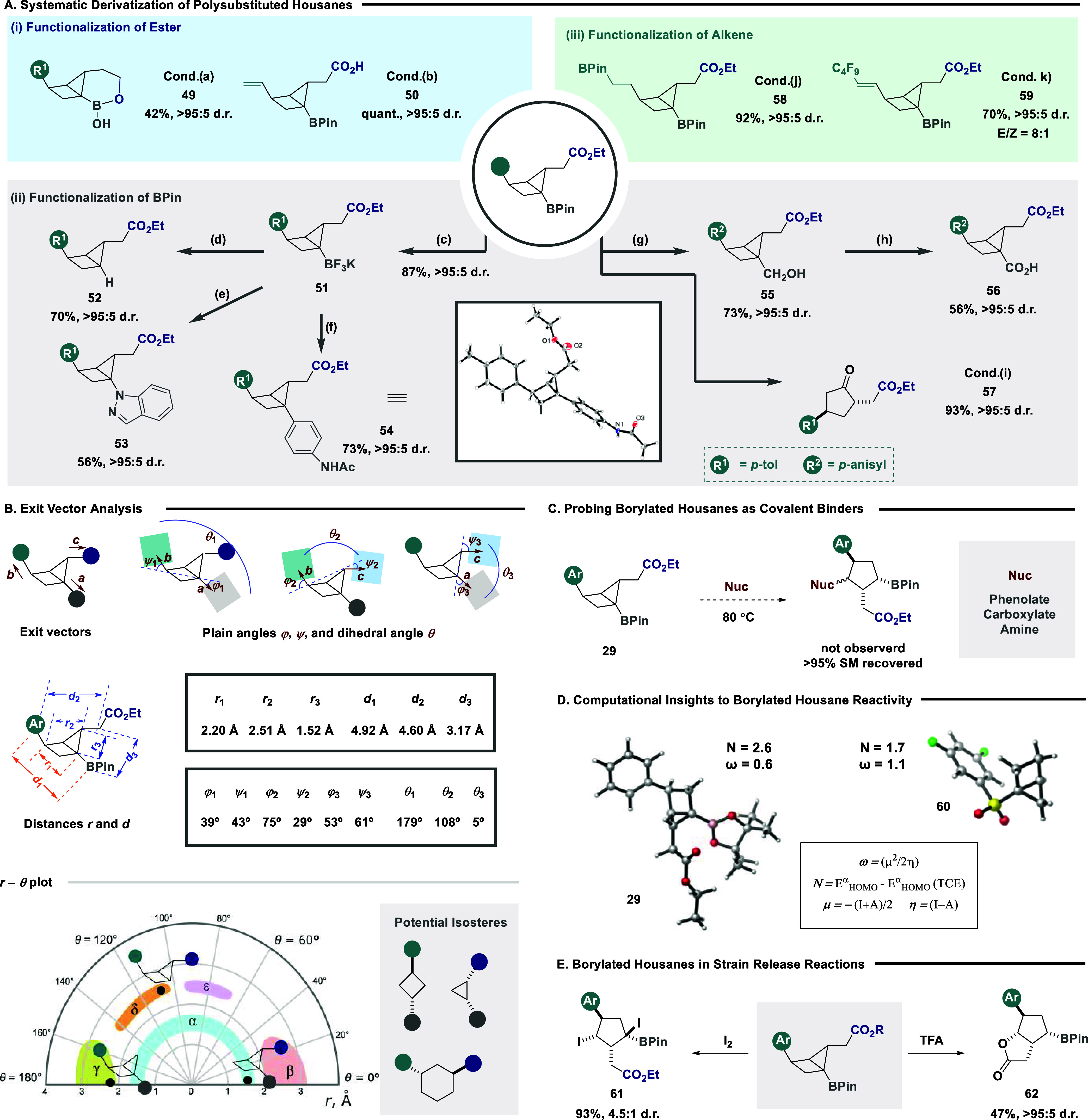
(A) Systematic derivatization of housane substituents; conditions (a): LiAlH_4_ (2.5 equiv), 0 °C; (b) LiOH (3 equiv), rt; (c) KF (4 equiv), L-tartaric acid (2.05 equiv), MeCN/MeOH, rt; (d) *t*-butylcatechol (6 equiv), Tol, 80 °C; (e) indazole (2 equiv), phenanthroline (1.2 equiv), Cu­(OAc)_2_ (1.2 equiv), K_3_PO_4_ (1 M in H_2_O, 3.0 equiv), DCE, 80 °C; (f) 4-bromoacetanilide (2 equiv), Pd­(dppf)­Cl_2_ (10 mol %), and Cs_2_CO_3_ (3 equiv), Tol/H_2_O, 90 °C; (g) CH_2_Br_2_ (2.5 equiv), *n*-BuLi (1.4 equiv), THF, −78 °C, then NaBO_3_·H_2_O; (h) NaIO_4_ (4 equiv) and RuCl_3_ (5 mol %); (i) NaBO_3_·H_2_O (5 equiv), THF, H_2_O buffer (pH = 7), 0 °C to rt; (j) [Ir­(cod)­Cl]_2_ (1 mol %), dppm (2 mol %), pinacolborane (1 equiv), DCM, rt; (k) nonafluoro-1-iodobutane (1.5 equiv), DBU (1.5 equiv), MeCN, 390 nm. (B) Exit vector analysis of fixed housane handles. (C) Probing the reactivity of the housane bridge. (D) Computational insights into nucleophilicity and electrophility parameters of model housanes. (E) Strain release-driven reactivity of housane with strong electrophiles and Brønsted acids.

To expedite the use of the generated housane scaffolds as potential medicinal chemistry fragments, we explored exit vector analysis from X-ray crystallography data for compound **35**.^40,41^ This method simulates functional groups attached to the ring system as exit vectors, with the substitution atoms being used as the starting points and the corresponding bonds as the direction. In the case of trisubstituted scaffolds, three exit vectors (***a***, ***b***, and ***c***) are necessary (Figure 3B). The corresponding geometric parameters (i.e., distances *r* and *d*, plane angles φ and ψ, and dihedral angles θ) are defined for each pair of vectors (***a***/***b***, ***b***/***c***, and ***a***/***c***). As might be expected, the relative orientation of exit vector pairs ***a***/***b*** (corresponding to BPin and aryl groups) and ***a***/***c*** (to BPin and CO_2_Et groups) resembles closely *trans*-1,3-disubstituted cyclobutane and *cis*-1,2-disubstituted cyclopropane, respectively (see Supporting Information, Figures S6 and S7 for more details). For the ***b***/***c*** pair (i.e., aryl and CO_2_Et groups), there is close similarity to the *trans*-1,3-disubstituted cyclohexane scaffold. Considering the relatively high occurrence of the above chemotypes in modern drug discovery, it is envisaged that all can be targets for isosteric replacements with the housane derivatives described in our work. Importantly, an additional exit vector is provided in each case, which is not accessible by the parent monocyclic scaffold.

The use of housanes with an electron withdrawing group strategically positioned at the bridgehead have been elegantly shown to be attacked by nucleophiles by both Baran and Grygorenko and co-workers to generate highly desirable polysubstituted cyclopentantanes.^13c,20b^ Inspired by the ramifications this can have for covalent binder design, in combination with the structural analogy of our positioned boron pinacol ester, we next explored the possibility of nucleophilic ring opening of our housane scaffolds (Figure 3C). Despite significant efforts, also at elevated temperatures, ring opening was not observed. We anticipated this was potentially down to two possible contributing factors: (1) the boron p-orbital acts as a nucleophilic reservoir; (2) nucleophilicity and electrophilicity parameters of the developed scaffolds do not mimic established systems. To probe this, we compared the electrophilicity and nucleophilicity values of our own housane scaffold to that of Baran and co-workers (**60**, Figure 3D).^20b,42^ Interestingly, the relative values follow an alternative trend, implying that the nucleophilicity of compound **29** is higher than that of the sulfone derived housane, while electrophilicity, in contrast, follows the opposite trend. These computational results suggest our generated housanes could potentially be activated in the presence of strong electrophiles or acids. Inspired by these results, the core housane scaffold was exposed to both iodine and TFA (Figure 3E). Pleasingly, ring opening was observed, enabling access to substrates **61** and **62** containing a cyclopentane scaffold with four contiguous stereocenters. It is pertinent to note that this complex cyclopentane scaffold is accessed in overall three steps from a diene precursor.

## Conclusion

In summary, we have established a boron-enabled strategy that delivers polysubstituted housanes with comprehensive selectivity from diene precursors. The judicious use of a *geminal* diboron motif not only addressed long-standing challenges in [2 + 2] cycloaddition but also unlocked a viable mild intramolecular cyclization to furnish housanes with high diastereoselectivity containing three defined exit vectors. The resulting scaffolds display remarkable stability, tunable reactivity, and compatibility with diverse derivatization protocols, enabling rapid exploration of previously inaccessible 3D chemical space. Beyond synthetic advances, the counterintuitive electrophile-driven reactivity highlights an untapped dimension of housane chemistry with immediate implications for fragment-based design. Taken together, these findings provide both a conceptual framework and a practical entry point for the broader adoption of housanes in medicinal chemistry, where their rigid architectures and defined exit vectors promise to expand the reach of next-generation therapeutics.

## Supplementary Material



## Notes

Experimental details, analytical data for compound characterization, reaction optimization, mechanistic studies, and computational studies (PDF)
