# Supplementary material for: Boron-Enabled Stereoselective Synthesis of Polysubstituted Housanes
Source: J Am Chem Soc. 2025 Dec 11;147(51):47730–41. doi: 10.1021/jacs.5c17624 (PMC12752696; doi:10.1021/jacs.5c17624)

Supplementary Information for

"Boron-enabled Stereoselective Synthesis of Polysubstituted Housanes"

Hao Fang,[1,2] Aimara García-Camacho,[3] Ho Seong Hwang,[1] Atthawut Sudsamart,[1,2]
Constantin G. Daniliuc,[4] Oleksandr O. Grygorenko,[5,6]
Ignacio Funes-Ardoiz[3]* and John J. Molloy[1]*

[1]Department of Biomolecular Systems, Max-Planck-Institute of Colloids and Interfaces, 14476 Potsdam, Germany

[2]Department of Chemistry and Biochemistry, Freie Universität Berlin, 14195 Berlin, Germany

[3]Department of Chemistry, Instituto de Investigación Química de la Universidad de La Rioja (IQUR), Universidad de La Rioja, Madre de Dios 53, 26004 Logroño, Spain

[4]Organisch-Chemisches Institut, Universität Münster, 48419 Münster, Germany

[5]Enamine Ltd., Winston Churchill Street 78, 02094 Kyiv, Ukraine

[6]Taras Shevchenko National University of Kyiv, Volodymyrska Street 60, 01601 Kyiv, Ukraine

Content

General Information 3
Synthesis of Starting Materials 4
Experimental Set up for Photoreactions 19
Reaction Optimization of [2 + 2] cycloaddition 20
Substrate scope of [2 + 2] cycloaddition 22
Scale Up Synthesis of **5** 39
Cyclic Voltammetry Studies 40
Reaction Optimization of Housane Formation 41
Substrate Scope of Housanes 42
Control Reactions of Housane Formation 54
Derivatizations 56
X-ray Analysis 64
Exit Vector Analysis 69
Computational Investigation 70
References 95
Spectrum 97

General Information

All chemicals were purchased as reagent grade and used without further purification unless stated otherwise. Commercial styrenes were passed through a pipette containing silica (1 cm) prior to use. Dry solvents were obtained by passing solvents through activated alumina columns and storing them over activated 4 Å molecular sieves 24 h. Solvent refers to bubbling argon through the solvent for a minimum of 15 min. Solvents for purification (extraction and chromatography) were purchased as technical grade and distilled on the rotary evaporator prior to use. For column chromatography SiO_2-(40-63 µm for flash chromatography was used as a stationary phase. For borylated housanes, $B(OH)_3$-SiO_2 was used to avoid degradation. Analytical thin layer chromatography (TLC) was performed on pre-coated TLC sheets ALUGRAM® XtraSIL G/UV_{254} (Macherey Nagel). UV light (254 nm), potassium permanganate ($KMnO_4$), vanillin and *p*-anisaldehyde stain solutions were used for visualization. Concentration under reduced pressure was performed at ~10 mbar and 40 °C, drying at ~10 mbar and ambient temperature. NMR spectra were measured on either a Varian 600 MHz, Bruker Ascend 400 or Bruker Ascend 700 at ambient temperature. The chemical shifts are referenced to the residual solvent peak as internal standard and are reported in ppm. The resonance multiplicity is abbreviated as: s (singlet), d (doublet), t (triplet), q (quadruplet), quint (quintet), m (multiplet) and b (broad). Assignments of unknown compounds are based on APT, DEPT, COSY(HH), HMBC, HSQC and NOESY spectra. Carbon atoms bearing boron were not observed by ^{13}C NMR and are not reported. High-resolution mass spectra were measured by the MS service of Freie Universität Berlin. IR spectra were recorded on a Perkin-Elmer Spectrum 100 FT-IR spectrometer, selected adsorption bands are reported in wavenumbers (cm^{-1}). Photoreactions were performed using Kessil PR-160L lamps (440 nm). Photoreaction set up, including light source emission is comprehensively described (*vide infra*).

Synthesis of Starting Materials

General Procedure A: Wittig Reaction

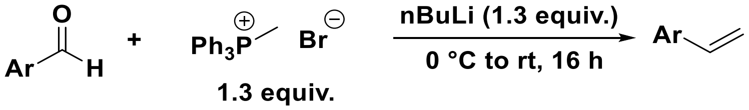

The reaction was performed according to a modified procedure of Koenigs *et al.*[1] To an oven-dried flask, methyltriphenylphosphonium bromide (1.3 equiv., 6.5 mmol, 2.32 g) and dry THF (20 mL, 0.25 M) were added under an argon atmosphere. The flask was cooled to 0 °C in an ice bath and *n*BuLi (2.5 M in hexane, 0.52 mL, 1.3 mmol, 1.3 equiv.) was added dropwise and stirred at 0 °C for 1 h. Then a solution of aldehyde (1.0 equiv., 5 mmol) in dry THF (10 mL) was added dropwise at 0 °C. The reaction vial was allowed to stir at ambient temperature for additional 16 h. Upon completion, the reaction was quenched by addition of H_2O (5 mL). The mixture was then extracted with Et_2O (3 × 50 mL). The combined organic phase was dried over Na_2SO_4, concentrated *in vacuo*, and purified by flash column chromatography (SiO_2, specified combination of solvents).

General Procedure B: Suzuki–Miyaura Cross-Coupling

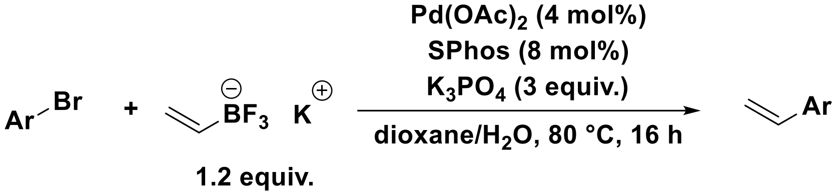

The reaction was performed according to a modified procedure of Molloy *et al.*[2] A two-necked flask equipped with a reflux condenser and a stirring bar was charged with aryl bromide (1 equiv.), potassium vinyltrifluoroborate (1.2 equiv.), $Pd(OAc)_2$ (4 mol%), SPhos (8 mol%), K_3PO_4 (3 equiv.). The flask was sealed and purged with nitrogen before the addition of deoxygenated dioxane/H_2O (10/1, 0.4 M). The reaction mixture was heated to 80 °C for 16 h. After cooling the crude reaction mixture was passed through celite and washed with EtOAc. The mixture was then extracted with EtOAc (3 × 50 mL). The combined organic phase was dried over Na_2SO_4, concentrated *in vacuo*, and purified by flash column chromatography (SiO_2, specified combination of solvents).

General Procedure C: Preparation of Amides

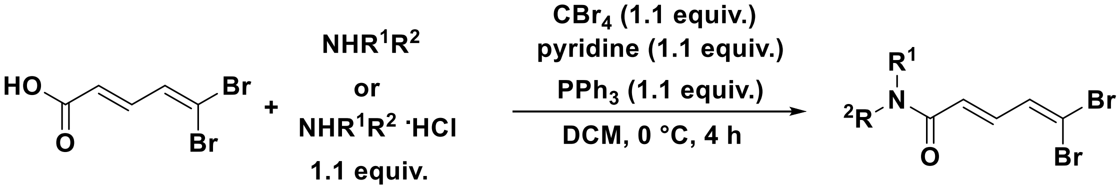

The reaction was performed according to a modified procedure of Gilmour *et al.*[3] To an oven-dried flask with a magnetic stir bar, **S7** (1.28 g, 5.0 mmol, 1.0 equiv.), amine or amine hydrochloride (5.5 mmol, 1.1 equiv.), CBr_4 (1.82 g, 5.5 mmol, 1.1 equiv.) and pyridine (0.44 mL, 5.5 mmol, 1.1 equiv.) were dissolved in dry DCM (20 mL, 0.25 M) under argon atmosphere. The solution was cooled to 0 °C before PPh_3 (1.44 g, 5.5 mmol, 1.1 equiv.) was slowly added. The reaction was stirred at 0 °C for 4 h, the mixture was filtered, concentrated *in vacuo*, and purified by flash column chromatography (SiO_2, specified combination of solvents).

General Procedure D: Preparation of ester

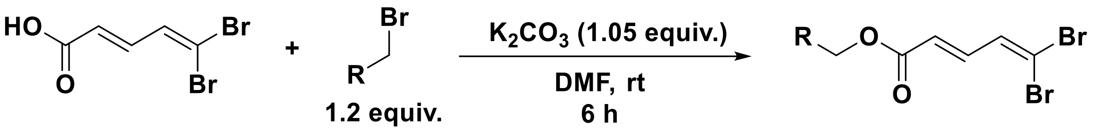

The reaction was performed according to a modified procedure of Gilmour *et al.*[3] To an oven-dried flask with a magnetic stir bar, **S7** (1.28 g, 5.0 mmol, 1.0 equiv.), alkyl bromide (5.5 mmol, 1.1 equiv.), K_2CO_3 (726 mg, 5.25 mmol, 1.05 equiv.) were dissolved in dry DMF (20 mL, 0.25 M) under argon atmosphere. The reaction was stirred at ambient temperature for 6 h. The mixture was filtered, concentrated *in vacuo*, and purified by flash column chromatography (SiO_2, specified combination of solvents).

General Procedure E: Steglich esterification

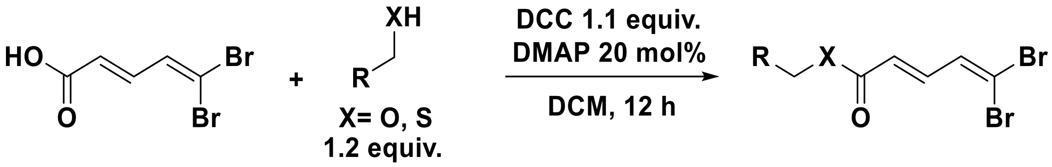

The reaction was performed according to a modified procedure of Song *et al.* [4] **S7** (1.28 g, 5 mmol, 1.0 equiv.) and DMAP (122 mg, 1 mmol, 20 mol%) were added to an oven-dried flask equipped with a magnetic stir bar and dissolved in dry DCM (10 mL, 0.5 M) under an argon atmosphere. The mixture was cooled to 0 °C before the subsequent addition of alcohol or thiol (5 mmol, 1 equiv.) and a solution of DCC (5.25 mL, 1 M in dry DCM, 5.25 mmol, 1.05 equiv.). The reaction was then allowed to gradually warm to ambient temperature and stirred for 12 h. Upon completion, the reaction was filtered over celite, washed with DCM, concentrated *in vacuo*, and purified by flash column chromatography (SiO_2, specified combination of solvents).

General Procedure F: 1, 1-Diborylalkenes formation

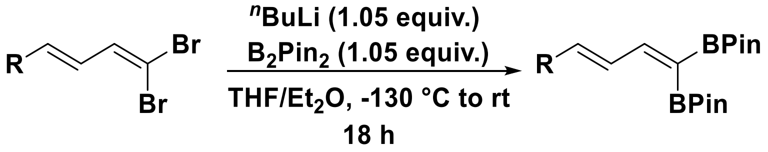

The reaction was performed according to a modified procedure of Meek *et al.*[5] 1, 1-dibromoalkene (1.0 equiv.) was added to an oven-dried flask equipped with a magnetic stir bar and dissolved in THF/Et_2O (2/1, 0.05 M) under an argon atmosphere. The reaction mixture was cooled to -130 ℃ in a liquid nitrogen/pentane bath. The solution was allowed to stir vigorously for 10 minutes before the dropwise addition of *n*BuLi (2.5 M in hexanes, 1.05 equiv.). The reaction was allowed to stir for an additional 10 minutes before the dropwise addition of a solution of B_2pin_2 (1.05 equiv.) in THF (0.3 M). The reaction was then allowed to gradually warm to ambient temperature and stirred for 18 h. Upon completion, the reaction was quenched with a saturated solution of NH_4Cl (30 mL). The mixture was then extracted with Et_2O (3 × 50 mL). The combined organic phase was dried over Na_2SO_4, concentrated *in vacuo*, and purified by flash column chromatography (SiO_2, specified combination of solvents).

3, 4-Dimethoxy-4'-vinyl-1, 1'-biphenyl (S1)

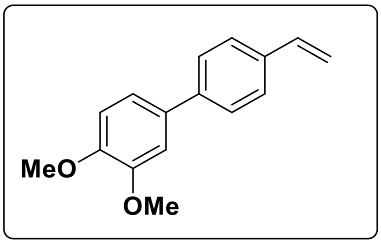

Prepared according to General Procedure **B**, 4'-Bromo-3, 4-dimethoxy-1, 1'-biphenyl (1466 mg, 5 mmol) was converted to **S1** yielding a yellow oil (1021 mg, 85%), after purification by flash column chromatography (SiO_2, 0→10% EtOAc/*n*-hexane).

^{1}H NMR (400 MHz, $CDCl_3$) δ = 7.55 – 7.51 (m, 2H), 7.47 (m, 2H), 7.16 (dd, *J* = 8.2, 2.1 Hz, 1H), 7.12 (d, *J* = 2.1 Hz, 1H), 6.95 (d, *J* = 8.3 Hz, 1H), 6.76 (dd, *J* = 17.6, 10.9 Hz, 1H), 5.79 (dd, *J* = 17.6, 0.9 Hz, 1H), 5.27 (dd, *J* = 10.9, 0.9 Hz, 1H), 3.96 (s, 3H), 3.93 (s, 3H) ppm; analytic data in agreement with literature.[6]

3-Vinylbenzonitrile (S2)

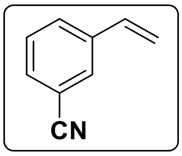

Prepared according to General Procedure **A**, 3-Formylbenzonitrile (656 mg, 5 mmol) was converted to **S2** yielding a yellow oil (490 mg, 76%), after purification by flash column chromatography (SiO_2, 0→5% EtOAc/*n*-hexane).

^{1}H NMR (400 MHz, $CDCl_3$) δ = 7.67 (s, 1H), 7.62 (dt, *J* = 7.8, 1.5 Hz, 1H), 7.53 (dt, *J* = 7.7, 1.4 Hz, 1H), 7.43 (t, *J* = 7.7 Hz, 1H), 6.69 (dd, *J* = 17.6, 10.9 Hz, 1H), 5.82 (d, *J* = 17.6 Hz, 1H), 5.39 (d, *J* = 10.9 Hz, 1H) ppm; analytic data in agreement with literature.[7]

1-Methyl-3-vinyl-1*H*-indole (S3)

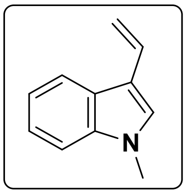

Prepared according to General Procedure **A**, 1-methylindole-3-carboxaldehyde (796 mg, 5 mmol) was converted to **S3** yielding a yellow oil (668 mg, 85%), after purification by flash column chromatography (SiO_2, 0→10% EtOAc/*n*-hexane).

^{1}H NMR (400 MHz, DMSO) δ = 7.83 (dt, *J* = 7.9, 1.0 Hz, 1H), 7.49 (s, 1H), 7.44 (dt, *J* = 8.2, 0.9 Hz, 1H), 7.20 (ddd, *J* = 8.2, 7.0, 1.2 Hz, 1H), 7.11 (ddd, *J* = 8.0, 7.0, 1.1 Hz, 1H), 6.90 – 6.77 (m, 1H), 5.62 (dd, *J* = 17.8, 1.6 Hz, 1H), 5.05 (dd, *J* = 11.3, 1.6 Hz, 1H), 3.77 (s, 3H) ppm; ; analytic data in agreement with literature.[8]

5-Vinylbenzofuran (S4)

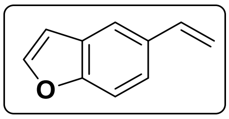

Prepared according to General Procedure **B**, 5-bromobenzofuran (985 mg, 5 mmol) was converted to **S4** yielding a colorless oil (613 mg, 85%), after purification by flash column chromatography (SiO_2, 0→5% EtOAc/*n*-hexane).

^{1}H NMR (400 MHz, $CDCl_3$) δ = 7.62 (dd, *J* = 4.2, 2.0 Hz, 2H), 7.47 (d, *J* = 8.6 Hz, 1H), 7.41 (dd, *J* = 8.5, 1.8 Hz, 1H), 6.83 (dd, *J* = 17.6, 10.9 Hz, 1H), 6.76 (dd, *J* = 2.2, 0.9 Hz, 1H), 5.74 (dd, *J* = 17.5, 0.9 Hz, 1H), 5.23 (dd, *J* = 10.8, 0.9 Hz, 1H) ppm; analytic data in agreement with literature.[2]

Isopropyl 2-methyl-2-(4-(4-vinylbenzoyl)phenoxy)propanoate (S5)

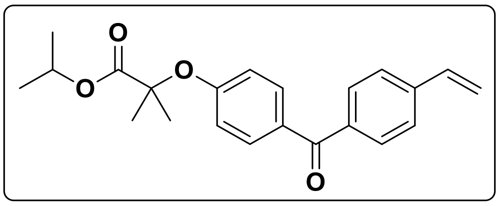

The reaction was performed according to a modified procedure of Echavarren *et al.* [9] Fenofibrate (7.22 g, 20 mmol), potassium vinyltrifluoroborate (4.69 g, 35 mmol, 1.75 equiv.), palladium(II) chloride (178 mg, 5 mol%), RuPhos (560 mg, 6 mol%) and cesium carbonate (19.54 g, 60 mmol, 3 equiv.) were dissolved in deoxygenated dioxane/H_2O (60 mL, 8/1, 0.33 M). The reaction mixture was stirred at 80 °C for 24 h under an argon atmosphere. The reaction mixture was filtered through celite, then extracted with EtOAc (3 × 50 mL). The combined organic phase was dried over Na_2SO_4, concentrated *in vacuo*.

Purification by flash column chromatography (SiO_2, 0→5% EtOAc/*n*-hexane) afforded **S5** as a white solid (6.7 g, 95%).

^{1}H NMR (400 MHz, $CDCl_3$) δ = 7.74 (t, *J* = 8.9 Hz, 4H), 7.49 (d, *J* = 8.2 Hz, 2H), 6.86 (d, *J* = 8.8 Hz, 2H), 6.77 (dd, *J* = 17.6, 10.9 Hz, 1H), 5.88 (dd, *J* = 17.6, 0.8 Hz, 1H), 5.39 (dd, *J* = 10.9, 0.7 Hz, 1H), 5.09 (p, *J* = 6.3 Hz, 1H), 1.66 (s, 6H), 1.20 (d, *J* = 6.3 Hz, 6H) ppm; analytic data in agreement with literature.[9]

Ethyl (*E*)-5, 5-dibromopenta-2, 4-dienoate (S6)

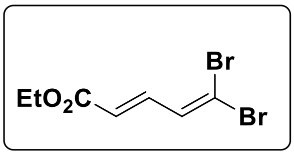

To a solution of PPh_3 (118 g, 450 mmol, 4.5 equiv.) in DCM (500 mL), CBr_4 (82.9 g, 250 mmol, 2.5 equiv.) in DCM (200 mL) was added slowly at 0˚C under an argon atmosphere. After stirring for 30 minutes a solution of ethyl (2*E*)-4-oxo-2-butenoate (100 mmol, 1 equiv.) in DCM (100 mL) was added. The reaction was stirred for 1 h at ambient temperature then filtered through celite, washed with aqueous $NaHCO_3$ (100 mL), brine (100 mL). The combined organic phase was dried over Na_2SO_4, concentrated under reduced pressure. Purification by flash column chromatography (SiO_2, 0→5% EtOAc/ *n*-hexane) afforded **S6** as a yellow solid (3.12 g, 82%).

^{1}H NMR (400 MHz, $CDCl_3$) δ = 7.29 (dd, *J* = 15.4, 10.8 Hz, 1H), 7.09 (dd, *J* = 10.8, 0.8 Hz, 1H), 6.04 (dd, *J* = 15.4, 0.8 Hz, 1H), 4.23 (q, *J* = 7.1 Hz, 2H), 1.31 (t, *J* = 7.1 Hz, 3H) ppm; analytic data in agreement with literature.[10]

(*E*)-5, 5-Dibromopenta-2, 4-dienoic acid (S7)

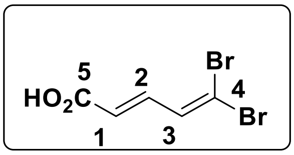

S6 (5.68 g, 20 mmol, 1 equiv.) was dissolved in EtOH (60 mL, 0.33 M). To this, a solution of NaOH in H_2O (18 mL, 2 M, 1.8 equiv.) was slowly added at 0˚C and the mixture was stirred for 1 h at ambient temperature before it was acidified by the addition of 37% aq. HCl to pH =1. The mixture was extracted with EtOAc (3 × 50 mL). The combined organic phase was dried over Na_2SO_4, concentrated *in vacuo* to afford **S7** as a yellow solid (4.66 g, 91%) without further purification.

^{1}H NMR (400 MHz, $CDCl_3$) δ = 7.40 (dd, *J* = 15.4, 10.9 Hz, 1H, H2), 7.13 (d, *J* = 10.9 Hz, 1H, H3), 6.06 (d, *J* = 15.4 Hz, 1H, H1) ppm; **^{13}C NMR** (100 MHz, $CDCl_3$) δ = 171.5 (C5), 142.0 (C2), 134.8 (C3), 123.3 (C1), 102.2 (C4) ppm; **IR** (ATR): ῦ = 696, 812, 880, 905, 947, 976, 1135, 1203, 1223, 1260, 1313, 1490, 1556, 1666, 2375 cm^{-1}; **HRMS** (ESI) $[M-H]^-$ Calculated mass for $C_5H_3Br_2O_2$:252.8505; Mass found:252.8499.

Pent-2-yn-1-yl (*E*)-5, 5-dibromopenta-2, 4-dienoate (S8)

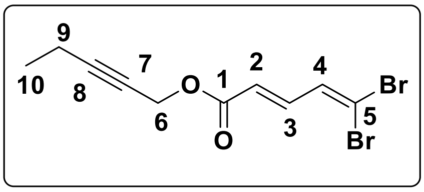

Prepared according to General Procedure **D**, **S7** (1.28 g, 5 mmol) was converted to **S8** yielding a yellow solid (1.58 g, 98%), after purification by flash column chromatography (SiO_2, 0→5% EtOAc/*n*-hexane).

R_f (5% EtOAc/*n*-hexane) = 0.50; **^{1}H NMR** (400 MHz, $CDCl_3$) δ = 7.23 (dd, *J* = 26.9, 16.0 Hz, 1H, H3), 7.01 (d, *J* = 10.6 Hz, 1H, H4), 5.99 (d, *J* = 15.4 Hz, 1H, H2), 4.69 – 4.67 (m, 2H, H6), 2.16 (qt, *J* = 7.6, 2.2 Hz, 2H, H9), 1.07 (t, *J* = 7.5 Hz, 3H, H10) ppm; **^{13}C NMR** (100 MHz, $CDCl_3$) δ = 165.7 (C1), 140.6 (C3), 135.0 (C2), 123.6 (C4), 101.2 (C5), 89.4 (C8), 73.2 (C7), 53.3 (C6), 13.7 (C10), 12.6 (C9) ppm; **IR** (ATR): ῦ = 712, 749, 779, 816, 847, 897, 973, 1027, 1125, 1144, 1177, 1246, 1269, 1307, 1371, 1428, 1508, 1620, 1696, 1712, 2232, 2301, 2375, 2937, 2978, 3026 cm^{-1}; **HRMS** (EI) $[M]^+$ Calculated mass for $C_{10}H_{10}Br_2O_2$:319.9042; Mass found:319.9054.

But-3-en-1-yl (*E*)-5, 5-dibromopenta-2, 4-dienoate (S9)

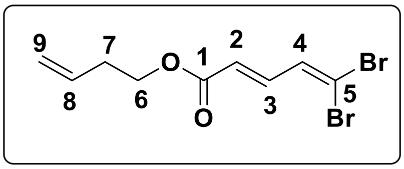

Prepared according to General Procedure **D**, **S7** (1.28 g, 5 mmol) was converted to **S9** yielding a yellow solid (1.33 g, 86%), after purification by flash column chromatography (SiO_2, 0→5% EtOAc/*n*-hexane).

***R**f* (5% EtOAc/*n*-hexane) = 0.50; **^{1}H NMR** (400 MHz, $CDCl_3$) δ = 7.30 (dd, *J* = 15.3, 10.8 Hz, 1H, H3), 7.08 (d, *J* = 10.8 Hz, 1H, H4), 6.04 (d, *J* = 15.4 Hz, 1H, H2), 5.80 (ddt, *J* = 17.0, 10.2, 6.8 Hz, 1H, H8), 5.19 – 5.04 (m, 2H, H9), 4.22 (t, *J* = 6.8 Hz, 2H, H6), 2.43 (q, *J* = 6.7 Hz, 2H, H7) ppm; **^{13}C NMR** (100 MHz, $CDCl_3$) δ = 166.3 (C1), 139.9 (C3), 135.1 (C2), 134.0 (C8), 124.3 (C4), 117.5 (C9), 100.6 (C5), 64.0 (C6), 33.2 (C7) ppm; **IR** (ATR): ῦ = 634, 712, 746, 815, 880, 915, 976, 1025, 1125, 1180, 1220, 1243, 1309, 1379, 1563, 1622, 1640, 1711, 2375, 2955, 3079 cm^{-1}; **HRMS** (EI) $[M]^+$ Calculated mass for $C_9H_{10}Br_2O_2$:307.9042; Mass found:307.9100.

***tert*-Butyl (*E*)-4-((5, 5-dibromopenta-2, 4-dienoyl)oxy)piperidine-1-carboxylate (S10)**

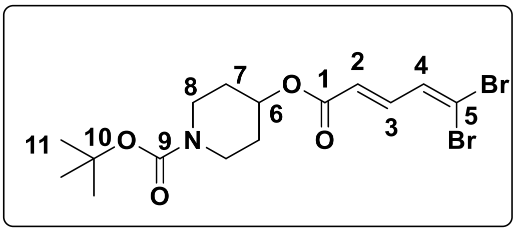

Prepared according to General Procedure **E**, **S7** (1.28 g, 5 mmol) was converted to **S10** yielding a yellow solid (1.80 g, 82%), after purification by flash column chromatography (SiO_2, 0→20% EtOAc/*n*-hexane).

***R**f* (20% EtOAc/*n*-hexane) = 0.45; **^{1}H NMR** (400 MHz, $CDCl_3$) δ = 7.33 – 7.26 (m, 1H, H3), 7.09 (dd, *J* = 10.8, 0.8 Hz, 1H, H4), 6.04 (d, *J* = 14.7 Hz, 1H, H2), 5.01 (tt, *J* = 8.0, 3.8 Hz, 1H, H6), 3.73 (tt, *J* = 8.4, 4.0 Hz, 2H, H8), 3.24 (ddd, *J* = 13.6, 8.7, 3.6 Hz, 2H, H8), 1.92 – 1.83 (m, 2H, H7), 1.64 (m, 2H, H7), 1.46 (s, 9H, H11) ppm; **^{13}C NMR** (100 MHz, $CDCl_3$) δ = 165.6 (C1), 154.9 (C9), 140.0 (C3), 135.0 (C2), 124.4 (C2), 100.9 (C5), 79.9 (C10), 70.4 (C6), 41.1 (C8), 30.7 (C7), 28.6 (C11) ppm; **IR** (ATR): ῦ = 605, 719, 740, 770, 818, 851, 888, 947, 983, 1022, 1091, 1131, 1162, 1237, 1253, 1274, 1307, 1322, 1366, 1415, 1451, 1477, 1616, 1679, 2859, 2971, 3567 cm^{-1}; **HRMS** (ESI) $[M+ACN+Na]^+$ Calculated mass for $C_{17}H_{24}N_2Br_2O_4Na$:500.9995; Mass found:500.9950.

***S*-Phenethyl (*E*)-5, 5-dibromopenta-2, 4-dienethioate (S11)**

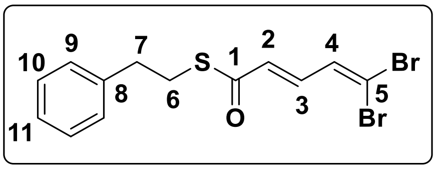

Prepared according to General Procedure **E**, **S7** (1.28 g, 5 mmol) was converted to **S11** yielding a yellow solid (1.84 g, 98%), after purification by flash column chromatography (SiO_2, 0→5% EtOAc/*n*-hexane).

***R**f* (5% EtOAc/*n*-hexane) = 0.40; **^{1}H NMR** (600 MHz, $CDCl_3$) δ = 7.26 – 7.22 (m, 2H, H10), 7.19 – 7.13 (m, 4H, H3+H9+H11), 6.97 (dd, *J* = 10.8, 0.8 Hz, 1H, H4), 6.22 (dd, *J* = 15.1, 0.8 Hz, 1H, H2), 3.18 – 3.14 (m, 2H, H6), 2.85 (dd, *J* = 8.7, 6.7 Hz, 2H, H7) ppm; **^{13}C NMR** (151 MHz, $CDCl_3$) δ = 189.4 (C1), 140.0 (C8), 135.5 (C3), 135.0 (C4), 130.5 (C2), 128.7 (C9), 128.7 (C10), 126.8 (C11), 101.9 (C5), 35.9 (C7), 30.7 (C6) ppm; **IR** (ATR): ῦ = 635, 667, 696, 722, 814, 877, 966, 1029, 1072, 1116, 1159, 1227, 1289, 1339, 1399, 1452, 1495, 1508, 1554, 1599, 1648, 2375, 2928, 3026 cm^{-1}; **HRMS** (EI) $[M]^+$ Calculated mass for $C_{13}H_{12}Br_2SO$:373.8970; Mass found: 373.9012.

(*E*)-5, 5-Dibromo-1-morpholinopenta-2, 4-dien-1-one (S12)

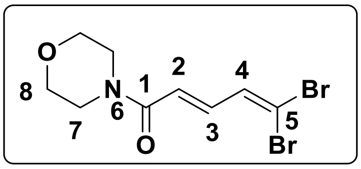

Prepared according to General Procedure **C**, **S7** (1.28 g, 5 mmol) was converted to **S12** yielding a yellow solid (1.30 g, 80%), after purification by flash column chromatography (SiO_2, 0→50% EtOAc/*n*-hexane).

R R_f (50% EtOAc/*n*-hexane) = 0.45; **^{1}H NMR** (400 MHz, $CDCl_3$) δ = 7.32 (dd, *J* = 14.8, 10.8 Hz, 1H, H3), 7.10 (d, *J* = 10.8 Hz, 1H, H4), 6.47 (d, *J* = 14.8 Hz, 1H, H2), 3.69-3.48 (m, 8H, H7+H8) ppm; **^{13}C NMR** (101 MHz, $CDCl_3$) δ = 164.9 (C1), 138.6 (C3), 135.4 (C4), 122.9 (C2), 99.3 (C5), 66.9 (C8), 46.3 (C7), 42.6 (C7) ppm; **IR** (ATR): ῦ = 651, 697, 735, 808, 855, 874, 914, 969, 1036, 1069, 1111, 1193, 1214, 1233, 1243, 1264, 1289, 1297, 1362, 1429, 1508, 1596, 1629 cm^{-1}; **HRMS** (EI) $[M]^+$ Calculated mass for $C_9H_{11}Br_2NO_2$:322.9151; Mass found: 322.9125.

(*E*)-5, 5-Dibromo-*N*-methoxy-*N*-methylpenta-2, 4-dienamide (S13)

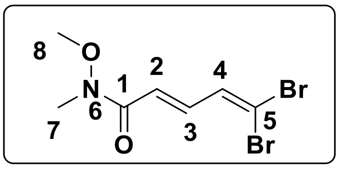

Prepared according to General Procedure **C**, **S7** (1.28 g, 5 mmol) was converted to **S13** yielding a yellow solid (1.16 g, 78%), after purification by flash column chromatography (SiO_2, 0→50% EtOAc/*n*-hexane).

R_f (50% EtOAc/*n*-hexane) = 0.35; **^{1}H NMR** (400 MHz, $CDCl_3$) δ = 7.34 (dd, *J* = 15.1, 10.8 Hz, 1H, H3), 7.15 (dd, *J* = 10.8, 0.8 Hz, 1H, H4), 6.64 (d, *J* = 15.1 Hz, 1H, H2), 3.70 (s, 3H, H8), 3.26 (s, 3H, H7) ppm; **^{13}C NMR** (100 MHz, $CDCl_3$) δ = 166.2 (C1), 138.6 (C3), 135.7 (C4), 122.3 (C2), 99.7 (C5), 62.1 (C8), 32.6 (C7) ppm; **IR** (ATR): ῦ = 625, 686, 712, 808, 821, 842, 894, 953, 976, 994, 1089, 1119, 1151, 1177, 1191, 1223, 1294, 1379, 1416, 1445, 1461, 1508, 1605, 1648, 2375, 2944, 2977, 3014 cm^{-1}; **HRMS** (EI) $[M]^+$ Calculated mass for $C_7H_9Br_2NO_2$:296.8995; Mass found:296.9011.

(*E*)-5, 5-Dibromo-1-(9*H*-carbazol-9-yl)penta-2, 4-dien-1-one (S14)

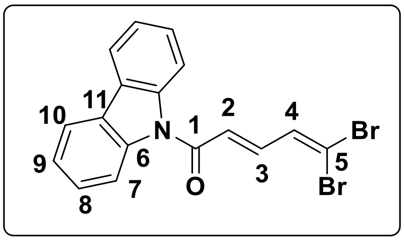

To an oven-dried flask, **S7** (1.28 g, 5 mmol) and dry DCM (10 mL, 0.5 M) were added under an argon atmosphere. The flask was cooled to 0 °C in an ice bath and oxalyl chloride (0.85 mL, 10.0 mmol, 2.0 equiv.) and a catalytic amount of DMF (5 drops) were added. The mixture was allowed to stir at ambient temperature for 4 h, then the reaction mixture was concentrated to give the acyl chloride.

To another oven-dried flask, carbazole (836 mg, 5 mmol, 1 equiv.) and dry THF (10 mL, 0.5 M) were added under an argon atmosphere. The flask was cooled to -78 °C in an ice bath before the dropwise addition of *n*BuLi (2.5 mL, 2.5 M in hexanes, 5.25 mmol, 1.05 equiv.). The mixture was allowed to stir at -78 °C for 1 h.

Then the acyl chloride made above was dissolved in dry THF (10 mL, 0.5 M) at 0 °C under an argon atmosphere before the dropwise addition of the lithiated carbazole solution. The mixture was slowly warmed to ambient temperature and stirred overnight. Purification by flash column chromatography (SiO_2, 0→10% EtOAc/ *n*-hexane) afforded **S14** as a yellow solid (1.56 g, 77%).

R_f (10% EtOAc/*n*-hexane) = 0.40; **^{1}H NMR** (400 MHz, $CDCl_3$) δ = 8.06 (d, *J* = 8.3 Hz, 1H, H7), 8.01 (d, *J* = 7.7 Hz, 1H, H10), 7.63 (dd, *J* = 15.0, 10.9 Hz, 1H, H3), 7.48 (ddd, *J* = 8.4, 7.3, 1.4 Hz, 2H, H8), 7.40 (td, *J* = 7.5, 1.0 Hz, 2H, H9), 7.32 (dd, *J* = 11.0, 0.8 Hz, 1H, H4), 7.03 (dd, *J* = 15.0, 0.8 Hz, 1H, H2) ppm; **^{13}C NMR** (100 MHz, $CDCl_3$) δ = 165.4 (C1), 140.4 (C3), 138.5 (C6),

135.2 (C4), 127.4 (C8), 126.8 (C2), 126.4 (C11), 123.9 (C9), 120.3 (C10), 115.7 (C7), 102.2 (C5) ppm; **IR** (ATR): ῦ = 615, 634, 671, 716, 737, 743, 773, 815, 881, 914, 973, 1099, 1137, 1171, 1210, 1227, 1284, 1362, 1442, 1477, 1490, 1508, 1656, 2312, 2375 cm^{-1}; **HRMS** (EI) $[M]^+$ Calculated mass for $C_{17}H_{11}Br_2NO$:402.9202; Mass found:402.9203.

(*E*)-5, 5-Dibromo-1-(4-(6-fluorobenzo[*d*]isoxazol-3-yl)piperidin-1-yl)penta-2, 4-dien-1-one (S15)

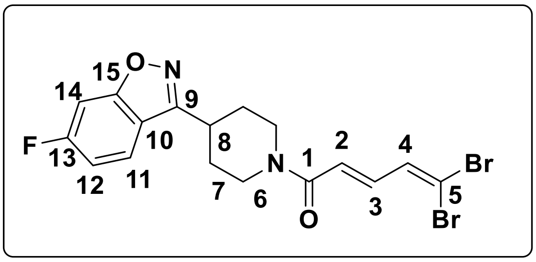

Prepared according to General Procedure **C**, **S7** (1.28 g, 5 mmol) was converted to **S15** yielding a yellow solid (1.33 g, 58%), after purification by flash column chromatography (SiO_2, 0→100% EtOAc/*n*-hexane).

Rf (EtOAc) = 0.45; **^{1}H NMR** (400 MHz, $CDCl_3$) δ = 7.62 (dd, *J* = 8.7, 5.0 Hz, 1H, H11), 7.32 (dd, *J* = 14.8, 10.8 Hz, 1H, H3), 7.26 (dd, *J* = 8.4, 2.1 Hz, 1H, H14), 7.12 (d, *J* = 10.8 Hz, 1H, H4), 7.08 (td, *J* = 9.0, 2.3 Hz, 1H, H12), 6.57 (d, *J* = 14.7 Hz, 1H, H2), 4.68 (m, 1H, H6), 4.15 – 4.03 (m, 1H, H6), 3.37 (m, 2H, H8+H6), 3.02 (m, 1H, H6), 2.22 – 2.12 (m, 2H, H7), 2.02 (m, 2H, H7) ppm; **^{13}C NMR** (100 MHz, $CDCl_3$) δ = 164.9 (C1), 164.4 (d, *J* = 251.4 Hz, C13), 164.1 (d, *J* = 13.6 Hz, C15), 160.2 (C9), 138.3 (C3), 135.6 (C4), 123.6 (C2), 122.3 (d, *J* = 11.1 Hz, C11), 117.2 (C10), 112.8 (d, *J* = 25.4 Hz, C12), 98.9 (C5), 97.8 (d, *J* = 26.8 Hz, C14), 45.8 (C6), 42.2 (C6), 34.4 (C9), 30.8 (C7), 30.2 (C7) ppm; **^{19}F NMR** (376 MHz, $CDCl_3$) δ = -108.97 (td, *J* = 8.8, 5.2 Hz) ppm; **IR** (ATR): ῦ = 608, 623, 648, 706, 762, 811, 881, 979, 1013, 1121, 1141, 1207, 1227, 1269, 1290, 1350, 1416, 1490, 1587, 1609, 1638, 2375, 2868 cm^{-1}; **HRMS** (ESI) $[M+Na]^+$ Calculated mass for $C_{17}H_{15}Br_2FN_2O_2Na$:478.9377; Mass found:478.9375.

5-(2, 5-Dimethylphenoxy)-2, 2-dimethylpentyl (*E*)-5, 5-dibromopenta-2, 4-dienoate (S16)

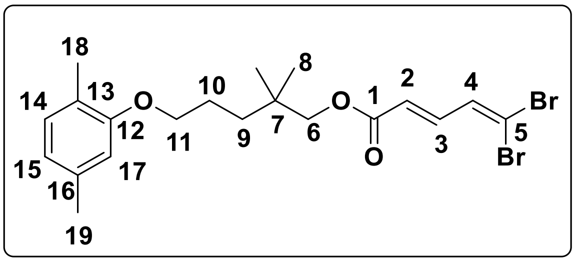

Prepared according to General Procedure **E**, **S7** (1.28 g, 5 mmol) was converted to **S16** yielding a yellow solid (1.97 g, 83%), after purification by flash column chromatography (SiO_2, 0→5% EtOAc/*n*-hexane).

R_f (5% EtOAc/*n*-hexane) = 0.50; **^{1}H NMR** (400 MHz, $CDCl_3$) δ = 7.32 (dd, *J* = 15.4, 10.8 Hz, 1H, H3), 7.09 (dd, *J* = 10.8, 0.8 Hz, 1H, H4), 7.01 (d, *J* = 7.4 Hz, 1H, H14), 6.66 (d, *J* = 7.5 Hz, 1H, H15), 6.61 (d, *J* = 1.6 Hz, 1H, H17), 6.06 (d, *J* = 15.4 Hz, 1H, H2), 3.97 – 3.89 (m, 4H, H11+H6), 2.31 (s, 3H, H19), 2.17 (s, 3H, H18), 1.78 (m, 2H, H10), 1.51 – 1.45 (m, 2H, H9), 0.99 (s, 6H, H8) ppm; **^{13}C NMR** (100 MHz, $CDCl_3$) δ = 166.4 (C1), 157.1 (C12), 139.8 (C3), 136.6 (C16), 135.0 (C4), 130.4 (C14), 124.3 (C2), 123.7 (C13), 120.8 (C15), 112.1 (C17), 100.6 (C5), 72.7 (C6), 68.4 (C11), 35.6 (C9), 33.9 (C7), 24.4 (C8), 24.2 (C10), 21.6 (C19), 15.9 (C18) ppm.**;** **IR** (ATR): ῦ = 696, 712, 816, 848, 880, 976, 1023, 1126, 1157, 1177, 1220, 1244, 1286, 1307, 1371, 1389, 1472, 1508, 1563, 1584, 1620, 1714, 2375 cm^{-1}; **HRMS** (EI) $[M]^+$ Calculated mass for $C_{20}H_{26}Br_2O_3$: 472.0249; Mass found: 472/0223.

(*E*)-5, 5-Dibromo-1-(4-(2-((2, 4-dimethylphenyl)thio)phenyl)piperazin-1-yl)penta-2, 4-dien-1-one (S17)

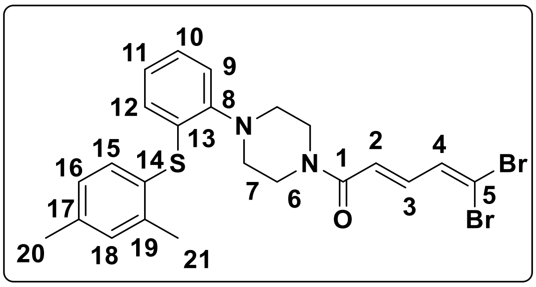

Prepared according to General Procedure **C**, **S7** (1.28 g, 5 mmol) was converted to **S17** yielding a yellow solid (1.93, 72%), after purification by flash column chromatography (SiO_2, 0→30% EtOAc/*n*-hexane).

***R**$_f$* (25% EtOAc/*n*-hexane) = 0.35; **^{1}H NMR** (700 MHz, $CDCl_3$) δ = 7.36 (d, *J* = 7.8 Hz, 1H, H15), 7.33 (dd, *J* = 14.9, 10.8 Hz, 1H, H3), 7.16 (d, *J* = 2.0 Hz, 1H, H18), 7.12 (dd, *J* = 10.8, 0.8 Hz, 1H, H4), 7.09 (td, *J* = 7.5, 1.5 Hz, 1H, H11), 7.04 (dd, *J* = 7.8, 2.0 Hz, 2H, H12+H16), 6.93 – 6.89 (m, 1H, H10), 6.60 – 6.53 (m, 2H, H9+H2), 3.90 (m, 2H, H6/7), 3.74 (m, 2H, H6/7), 3.09 (m, 4H, 6/7), 2.36 (s, 3H, H21), 2.32 (s, 3H, H20) ppm; **^{13}C NMR** (176 MHz, $CDCl_3$) δ = 164.9 (C1), 148.4 (C8), 142.4 (C17), 139.5 (C14), 138.2 (C3), 136.2 (C15), 135.6 (C4), 134.7 (C13), 131.9 (C18), 128.0 (C16), 127.7 (C19), 126.6 (C9), 125.8 (C11), 125.2 (C10), 123.6 (C2), 120.2 (C12), 98.9 (C5), 52.2 (C6/7), 51.4 (C6/7), 46.5 (C6/7), 42.8 (C6/7), 21.3 (C21), 20.7 (C20) ppm; **IR** (ATR): ῦ = 669, 700, 737, 769, 809, 848, 971, 1033, 1109, 1137, 1164, 1214, 1259, 1306, 1339, 1369, 1470, 1508, 1648, 1715, 2375, 2977 cm^{-1}; **HRMS** (ESI) $[M+Na]^+$ Calculated mass for $C_{23}H_{24}Br_2N_2OSNa$: 556.9868; Mass found: 556.9878.

(*E*)-(5-Ethoxy-5-oxopenta-1, 3-diene-1, 1-diyl)diboronic acid, pinacol ester (1)

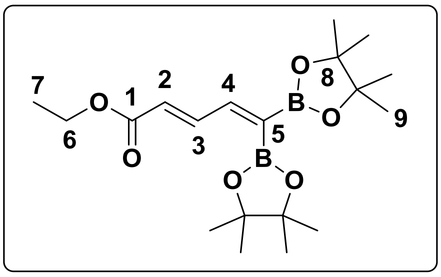

Prepared according to General Procedure **F**, **S6** (2.84 g, 10 mmol) was converted to **1** yielding a yellow solid (2.84 g, 75%), after purification by flash column chromatography (SiO_2, 0→10% EtOAc/*n*-hexane).

***R**$_f$* (10% EtOAc/*n*-hexane) = 0.35; **^{1}H NMR** (400 MHz, $CDCl_3$) δ = 7.63 (dd, *J* = 15.2, 11.4 Hz, 1H, H3), 7.33 (d, *J* = 11.5 Hz, 1H, H4), 6.00 (dd, *J* = 15.2, 0.7 Hz, 1H, H2), 4.19 (q, *J* = 7.1 Hz, 2H, H6), 1.33 (s, 12H, H9), 1.29 (t, *J* = 7.1 Hz, 3H, H7), 1.26 (s, 12H, H9) ppm; **^{13}C NMR** (100 MHz, $CDCl_3$) δ = 167.0 (C1), 153.1 (C4), 144.4 (C3), 125.8 (C2), 84.0 (C8), 83.6 (C8), 60.6 (C6), 24.94 (C9), 24.86 (C9), 14.4 (C7) ppm; **^{11}B NMR** (128 MHz, $CDCl_3$) δ = 30.79 ppm; **IR** (ATR): ῦ = 615, 634, 670, 716, 743, 773, 815, 851, 966, 986, 1009, 1036, 1125, 1181, 1213, 1230, 1254, 1302, 1343, 1371, 1379, 1389, 1508, 1566, 1629, 1648, 1656, 1714, 2374, 2978 cm^{-1}; **HRMS** (ESI) $[M+Na]^+$ Calculated mass for $C_{19}H_{32}B_2O_6Na$: 401.2277; Mass found: 401.2322.

((1*E*,3*E*)-5-Ethoxy-5-oxopenta-1,3-dien-1-yl)boronic acid, pinacol ester (2)

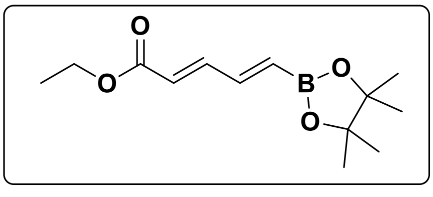

The reaction was performed according to a modified procedure of Morken *et al.*[11] To an oven-dried flask with a magnetic stir bar, 2,2,6,6-tetramethylpiperidine (2.2 mL, 13 mmol, 1.3 equiv.) and THF (30 mL, 0.43 M) were added under an argon atmosphere. The flask was cooled to -78 ℃ in a dry ice/acetone bath and *n*BuLi (2.5 M in hexane, 5.2 mL, 13 mmol, 1.3 equiv.) was added dropwise. The mixture was allowed to warm to 0 ℃ and stirred at 0 ℃ for 1 h. Then a solution of bis(4,4,5,5-tetramethyl1,3,2-dioxaborolan-2-yl)methane (12 mmol, 1.2 equiv.) in THF (10 mL, 1.2 M) was added. The reaction vial was allowed to stir for 5 minutes at 0 ℃. Then the flask was cooled to -78 ℃, and a solution of ethyl (2*E*)-4-oxo-2-butenoate (10 mmol, 1.0 equiv.) in THF (10 mL, 1 M) was added dropwise. The

reaction vial was allowed to stir at -78 ℃ for additional 4 h. Upon completion, the reaction was quenched by addition of H_2O (5 mL). The mixture was then extracted with Et_2O (3 × 10 mL). The combined organic phase was dried over Na_2SO_4, concentrated *in vacuo*. Purification by flash column chromatography (SiO_2, 0→5% EtOAc/ *n*-hexane) afforded **2** as a yellow oil (1.64 g, 65%).

^{1}H NMR (400 MHz, $CDCl_3$) δ = 7.31 – 7.21 (m, 1H), 7.04 (ddd, *J* = 17.7, 10.9, 0.7 Hz, 1H), 6.01 – 5.91 (m, 2H), 4.21 (q, *J* = 7.1 Hz, 2H), 1.29 (m, 15H) ppm; analytic data in agreement with literature.[12]

((1*E*,3*E*)-5-Ethoxy-5-oxopenta-1,3-dien-1-yl)boronic acid, MIDA ester (3)

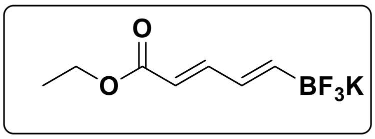

Prepared according to literature procedure of Gilmour *et al.* [13] Aq. KHF_2 (4.5 M, 5.5 mL, 25.0 mmol, 5.0 equiv.) was added dropwise to a stirred solution of **2** (1.26 g, 5.0 mmol, 1.0 equiv.) in MeOH (10 mL, 0.5 M). The reaction was stirred at ambient temperature for 1 h before the reaction mixture was concentrated *in vacuo* and dried at high vacuum overnight. The resulting solid was washed with acetone (3 × 5 mL) and filtered. The filtrate was concentrated to ~ 3 mL *in vacuo* and Et_2O (50 mL) was added to form a precipitate. The solid was filtered to yield **4** as a white solid (905 mg, 78%) without further purification.

^{1}H NMR (400 MHz, CD_3CN) δ = 7.20 (ddt, *J* = 15.4, 10.8, 0.8 Hz, 1H), 6.45 (dd, *J* = 17.4, 10.8 Hz, 1H), 6.25 – 6.14 (m, 1H), 5.73 (dd, *J* = 15.3, 1.0 Hz, 1H), 4.12 (q, *J* = 7.1 Hz, 2H), 1.24 (t, *J* = 7.1 Hz, 3H) ppm.

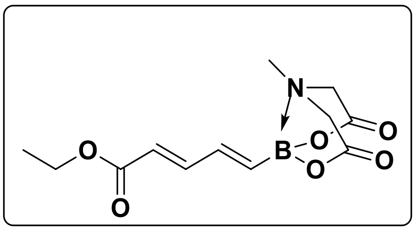

Prepared according to literature procedure of Gilmour *et al.* [13] To a flask containing **4** (696 mg, 3 mmol, 1.0 equiv.) and SiO_2 (223 mg, 3 mmol, 1.0 equiv.) under argon was added H_2O (10 mL, 0.3 M). The reaction mixture was stirred at ambient temperature for 1 h before it was filtered, and the filter cake was thoroughly washed with EtOAc. The aqueous phase was extracted with EtOAc (2 × 30 mL). The combined organic phases were combined, washed with brine (10 mL), dried over Na_2SO_4, and concentrated *in vacuo*. The crude was directly used for next step without further purification. Then to an oven-dried flask, the above-made boronic acid (340 mg, 2 mmol, 1.0 equiv.) and *N*-methyliminodiacetic acid (324 mg, 2.2 mmol, 1.1 equiv.) and dry DMF (8.3 mL, 0.3 M) were added successively under an argon atmosphere. The reaction mixture was stirred at 90 °C for 24 h. The reaction mixture was concentrated *in vacuo* and Et_2O (20 mL) was added to form a precipitate. The solid was filtered to yield **3** as a white solid (565 mg, 67%) over 2 steps.

^{1}H NMR (400 MHz, Acetone) δ = 7.26 (ddd, *J* = 15.4, 10.8, 0.9 Hz, 1H), 6.74 (dd, *J* = 17.4, 10.8 Hz, 1H), 6.22 (d, *J* = 17.4 Hz, 1H), 5.99 (d, *J* = 15.4 Hz, 1H), 4.27 (d, *J* = 16.9 Hz, 2H), 4.16 (q, *J* = 7.1 Hz, 2H), 4.08 (d, *J* = 16.9 Hz, 2H), 1.25 (t, *J* = 7.1 Hz, 3H) ppm; analytic data in agreement with literature.[14]

(*E*)-(5-(*tert*-Butoxy)-5-oxopenta-1,3-diene-1,1-diyl)diboronic acid, pinacol ester (S18)

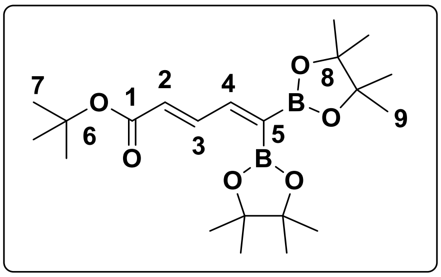

To an oven-dried flask, **S7** (1.28 g, 5 mmol) and *tert*-butyl-2,2,2-trichloroacetimidate (2.18 g, 10 mmol, 2 equiv.) were dissolved in dry DCM (20 mL, 0.25 M) under an argon atmosphere. The mixture was allowed to stir at ambient temperature for 16 h. Purification by flash column chromatography (SiO_2, 5% EtOAc/ n-hexane) afforded the gem-bromo product as a yellow solid (1.37 g, 88%) without further purification.

Prepared according to General Procedure **F**, the above-made crude compound (1.25 g, 4 mmol) was converted to **S18** yielding a yellow solid (1.33 g, 82%), after purification by flash column chromatography (SiO_2, 0→10% EtOAc/*n*-hexane).

R R_f (10% EtOAc/*n*-hexane) = 0.30; **^{1}H NMR** (400 MHz, $CDCl_3$) δ = 7.51 (dd, *J* = 15.2, 11.5 Hz, 1H, H3), 7.31 (d, *J* = 11.5 Hz, 1H, H4), 5.92 (d, *J* = 15.1 Hz, 1H, H2), 1.48 (s, 9H, H7), 1.32 (s, 12H, H9), 1.26 (d, *J* = 1.0 Hz, 12H, H9) ppm; **^{13}C NMR** (100 MHz, $CDCl_3$) δ = 166.2 (C1), 153.5 (C4), 143.2 (C3), 128.2 (C2), 83.9 (C8), 83.6 (C8), 80.5 (C6), 28.3 (C7), 24.92 (C9), 24.88 (C9) ppm; **^{11}B NMR** (128 MHz, $CDCl_3$) δ = 30.39 ppm; **IR** (ATR): ῦ = 679, 741, 850, 936, 962, 986, 1127, 1163, 1256, 1282, 1321, 1349, 1367, 1387, 1459, 1569, 1631, 1707, 2934, 2978 cm^{-1}; **HRMS** (ESI) $[M+Na]^+$ Calculated mass for $C_{21}H_{36}B_2O_6Na$: 429.2590; Mass found: 429.2599.

(*E*)-(5-Oxo-5-(pent-2-yn-1-yloxy)penta-1, 3-diene-1, 1-diyl)diboronic acid, pinacol ester (S19)

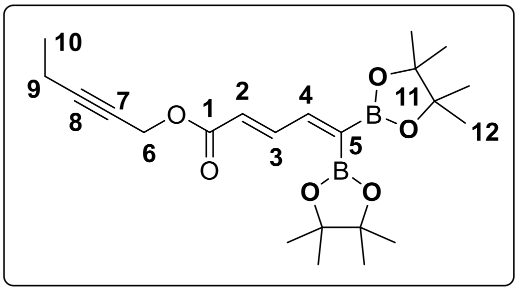

Prepared according to General Procedure **F**, **S8** (644 mg, 2 mmol) was converted to **S19** yielding a yellow oil (599 mg, 72%), after purification by flash column chromatography (SiO_2, 0→10% EtOAc/*n*-hexane).

R_f (10% EtOAc/*n*-hexane) = 0.30; **^{1}H NMR** (400 MHz, $CDCl_3$) δ = 7.67 (dd, *J* = 15.3, 11.5 Hz, 1H, H3), 7.32 (d, *J* = 11.5 Hz, 1H, H4), 6.02 (dd, *J* = 15.3, 0.7 Hz, 1H, H2), 4.72 (t, *J* = 2.2 Hz, 2H, H6), 2.22 (qt, *J* = 7.5, 2.2 Hz, 2H, H9), 1.32 (s, 12H, H12), 1.25 (s, 12H, H12), 1.13 (t, *J* = 7.5 Hz, 3H, H10) ppm; **^{13}C NMR** (100 MHz, $CDCl_3$) δ = 166.3 (C1), 152.8 (C4), 145.2 (C3), 124.8 (C2), 89.0 (C8), 84.0 (C11), 83.6 (C11), 73.4 (C7), 53.0 (C6), 25.1 (C12), 24.9 (C12), 24.8 (C12), 13.7 (C10), 12.6 (C9) ppm; **^{11}B NMR** (128 MHz, $CDCl_3$) δ = 30.82 ppm; **IR** (ATR): ῦ = 663, 743, 848, 959, 984, 1009, 1121, 1172, 1254, 1280, 1340, 1371, 1389, 1566, 1629, 1719, 2933, 2978 cm^{-1}; **HRMS** (ESI) $[M+Na]^+$ Calculated mass for $C_{22}H_{34}B_2O_6Na$: 439.2434; Mass found: 439.2402.

(*E*)-(5-(But-3-en-1-yloxy)-5-oxopenta-1, 3-diene-1, 1-diyl)diboronic acid, pinacol ester (S20)

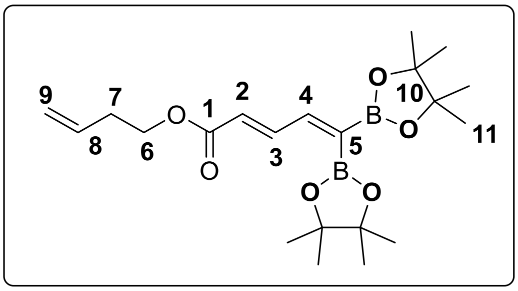

Prepared according to General Procedure **F**, **S9** (620 mg, 2 mmol) was converted to **S20** yielding a yellow solid (550 mg, 68%), after purification by flash column chromatography (SiO_2, 0→10% EtOAc/*n*-hexane).

R_f (10% EtOAc/*n*-hexane) = 0.30; **^{1}H NMR** (400 MHz, $CDCl_3$) δ = 7.63 (dd, *J* = 15.3, 11.4 Hz, 1H, H3), 7.33 (d, *J* = 11.5 Hz, 1H, H4), 6.00 (d, *J* = 15.2 Hz, 1H, H2), 5.91 – 5.70 (m, 1H, H8), 5.18 – 5.01 (m, 2H, H9), 4.19 (t, *J* = 6.7 Hz, 2H, H6), 2.41 (qt, *J* = 6.7, 1.4 Hz, 2H, H7), 1.32 (s, 12H,

H11), 1.25 (s, 12H, H11) ppm; **^{13}C NMR** (100 MHz, $CDCl_3$) δ = 166.9 (C1), 153.1 (C4), 144.4 (C3), 134.1 (C8), 125.3 (C2), 117.3 (C9), 84.0 (C10), 83.6 (C10), 63.7 (C6), 33.1 (C7), 25.1 (C11), 24.9 (C11), 24.8 (C11) ppm; **^{11}B NMR** (128 MHz, $CDCl_3$) δ = 31.16 ppm; **IR** (ATR): ῦ = 742, 849, 918, 960, 984, 1009, 1124, 1174, 1211, 1254, 1282, 1342, 1371, 1389, 1566, 1629, 1714, 2933, 2978 cm^{-1}; **HRMS** (ESI) $[M+Na]^+$ Calculated mass for $C_{21}H_{34}B_2O_6Na$: 427.2434; Mass found: 427.2411.

(*E*)-(5-((1-(*tert*-Butoxycarbonyl)piperidin-4-yl)oxy)-5-oxopenta-1, 3-diene-1, 1-diyl)diboronic acid, pinacol ester (S21)

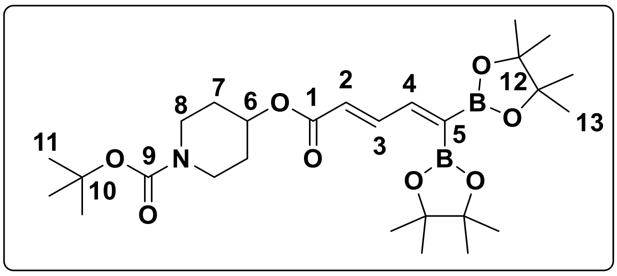

Prepared according to General Procedure **F**, **S10** (878 mg, 2 mmol) was converted to **S21** yielding a yellow solid (715 mg, 67%), after purification by flash column chromatography (SiO_2, 0→30% EtOAc/*n*-hexane).

Rf (25% EtOAc/*n*-hexane) = 0.25; **^{1}H NMR** (400 MHz, $CDCl_3$) δ = 7.65 (dd, *J* = 15.3, 11.5 Hz, 1H, H3), 7.34 (d, *J* = 11.5 Hz, 1H, H4), 5.99 (dd, *J* = 15.3, 0.7 Hz, 1H, H2), 4.99 (tt, *J* = 7.8, 3.7 Hz, 1H, H6), 3.78 – 3.64 (m, 2H, H8), 3.26 (ddd, *J* = 13.5, 8.4, 3.7 Hz, 2H, H8), 1.87 (m, 2H, H7), 1.61 (m, 2H, H7), 1.46 (s, 9H, H11), 1.32 (s, 12H, H13), 1.26 (s, 12H, H13) ppm; **^{13}C NMR** (100 MHz, $CDCl_3$) δ = 166.2 (C1), 154.9 (C9), 153.0 (C4), 144.6 (C3), 125.9 (C2), 84.0 (C12), 83.7 (C12), 79.8 (C10), 69.9 (C6), 41.1 (C8), 30.7 (C7), 28.6 (C11), 24.93 (C13), 24.88 (C13) ppm; **^{11}B NMR** (128 MHz, $CDCl_3$) δ = 31.44 ppm; **IR** (ATR): ῦ = 650, 671, 703, 719, 753, 822, 852, 947, 967, 981, 1013, 1072, 1102, 1138, 1165, 1223, 1267, 1304, 1371, 1389, 1411, 1566, 1735, 2859, 2920, 2977 cm^{-1}; **HRMS** (ESI) $[M+Na]^+$ Calculated mass for $C_{27}H_{45}B_2NO_8Na$: 556.3223; Mass found: 556.3242.

(*E*)-(5-Oxo-5-(phenethylthio)penta-1, 3-diene-1, 1-diyl)diboronic acid, pinacol ester (S22)

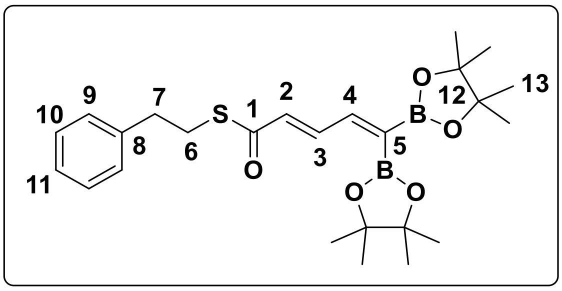

Prepared according to General Procedure **F**, **S11** (752 mg, 2 mmol) was converted to **S22** yielding a yellow solid (357 mg, 38%), after purification by flash column chromatography (SiO_2, 0→10% EtOAc/*n*-hexane).

Rf (10% EtOAc/*n*-hexane) = 0.35; **^{1}H NMR** (400 MHz, $CDCl_3$) δ = 7.62 (dd, *J* = 15.2, 11.3 Hz, 1H, H3), 7.39 – 7.19 (m, 7H, H4+H9+H10+H11), 6.28 (d, *J* = 15.1 Hz, 1H, H2), 3.23 (dd, *J* = 8.7, 6.5 Hz, 2H, H6), 2.93 (dd, *J* = 8.7, 6.6 Hz, 2H, H7), 1.36 (s, 12H, H13), 1.28 (s, 12H, H13) ppm; **^{13}C NMR** (100 MHz, $CDCl_3$) δ = 190.2 (C1), 153.1 (C4), 140.4 (C3), 140.2 (C8), 132.5 (C2), 128.8 (C9), 128.6 (C10), 126.6 (C11), 84.0 (C12), 83.7 (C12), 83.6 (C12), 36.0 (C7), 30.4 (C6), 25.2 (C13), 24.9 (C13), 24.9 (C13) ppm; **^{11}B NMR** (128 MHz, $CDCl_3$) δ = 30.75 ppm; **IR** (ATR): ῦ = 670, 743, 818, 848, 981, 1009, 1138, 1167, 1211, 1279, 1339, 1371, 1389, 1454, 1474, 1508, 1564, 1649, 1666, 2375, 2931, 2977 cm^{-1}; **HRMS** (ESI) $[M+Na]^+$ Calculated mass for $C_{25}H_{36}B_2SO_5Na$: 493.2362; Mass found: 493.2362.

(*E*)-(5-Morpholino-5-oxopenta-1, 3-diene-1, 1-diyl)diboronic acid, pinacol ester (S23)

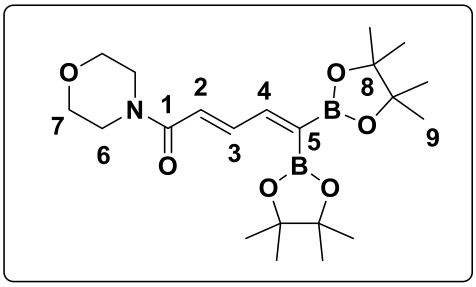

Prepared according to General Procedure **F**, **S12** (650 mg, 2 mmol) was converted to **S23** yielding a yellow solid (377 mg, 45%), after purification by flash column chromatography (SiO_2, 0→50% EtOAc/*n*-hexane).

Rf (50% EtOAc/*n*-hexane) = 0.30; **^{1}H NMR** (400 MHz, $CDCl_3$) δ = 7.60 (dd, *J* = 14.7, 11.5 Hz, 1H, H3), 7.37 (d, *J* = 11.5 Hz, 1H, H4), 6.43 (dd, *J* = 14.7, 0.7 Hz, 1H, H2), 3.61 (m, 8H, H6+H7), 1.32 (s, 12H, H9), 1.25 (s, 12H, H9) ppm; **^{13}C NMR** (100 MHz, $CDCl_3$) δ = 165.6 (C1), 154.0 (C4), 142.8 (C3), 124.9 (C3), 83.9 (C8), 83.5 (C8), 66.9 (C7), 46.3 (C6), 42.5 (C6), 24.94 (C9), 24.89 (C9), 24.7 (C9) ppm; **^{11}B NMR** (128 MHz, $CDCl_3$) δ = 30.92 ppm; **IR** (ATR): ῡ = 673, 697, 740, 802, 851, 925, 951, 984, 1009, 1043, 1066, 1115, 1135, 1223, 1261, 1304, 1343, 1371, 1389, 1401, 1458, 1474, 1521, 1566, 1600, 1640, 2927, 2977 cm^{-1}; **HRMS** (ESI) $[M+K]^+$ Calculated mass for $C_{21}H_{35}B_2NO_6K$: 458.2282; Mass found: 458.2247.

(*E*)-(5-(Methoxy(methyl)amino)-5-oxopenta-1, 3-diene-1, 1-diyl)diboronic acid, pinacol ester (S24)

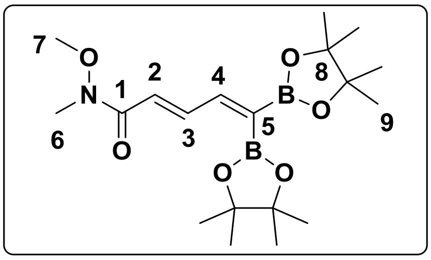

Prepared according to General Procedure **F**, **S13** (598 mg, 2 mmol) was converted to **S24** yielding a yellow solid (385 mg, 49%), after purification by flash column chromatography (SiO_2, 0→50% EtOAc/*n*-hexane).

R_f (50% EtOAc/*n*-hexane) = 0.25; **^{1}H NMR** (400 MHz, $CDCl_3$) δ = 7.65 (dd, *J* = 14.9, 11.5 Hz, 1H, H3), 7.41 (d, *J* = 11.6 Hz, 1H, H4), 6.62 (d, *J* = 14.9 Hz, 1H, H2), 3.67 (s, 3H, H7), 3.24 (s, 3H, H6), 1.32 (s, 12H, H9), 1.25 (s, 12H, H9) ppm; **^{13}C NMR** (100 MHz, $CDCl_3$) δ = 166.9 (C1), 154.0 (C4), 143.1 (C3), 124.0 (C2), 83.9 (C8), 83.5 (C8), 62.0 (C7), 32.5 (C6), 24.9 (C9), 24.8 (C9) ppm; **^{11}B NMR** (128 MHz, $CDCl_3$) δ = 30.02 ppm; **IR** (ATR): ῡ = 851, 984, 1137, 1231, 1261, 1332, 1582, 1619, 1648, 2976 cm^{-1}; **HRMS** (ESI) $[M+Na]^+$ Calculated mass for $C_{19}H_{33}B_2NO_6Na$: 416.2386; Mass found: 416.2394.

(*E*)-(5-(9*H*-Carbazol-9-yl)-5-oxopenta-1, 3-diene-1, 1-diyl)diboronic acid, pinacol ester (S25)

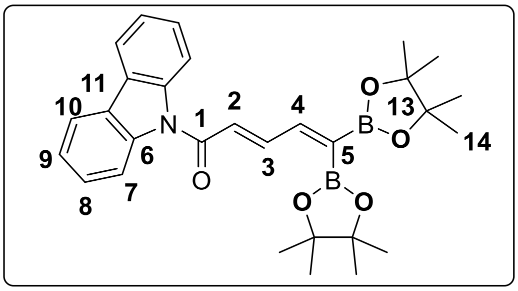

Prepared according to General Procedure **F**, **S14** (810 mg, 2 mmol) was converted to **S25** yielding a yellow solid (699 mg, 70%), after purification by flash column chromatography (SiO_2, 0→10% EtOAc/*n*-hexane).

R_f (10% EtOAc/*n*-hexane) = 0.20; **^{1}H NMR** (400 MHz, $CDCl_3$) δ = 8.10 (d, *J* = 8.3 Hz, 2H, H7), 8.00 (d, *J* = 7.6 Hz, 2H, H10), 7.93 (dd, *J* = 14.9, 11.5 Hz, 1H, H3), 7.55 (d, *J* = 11.5 Hz, 1H, H4), 7.49 – 7.43 (m, 2H, H8), 7.38 (t, *J* = 7.4 Hz, 2H, H9), 7.00 (d, *J* = 14.9 Hz, 1H, H2), 1.36 (s, 12H, H14), 1.30 (s, 12H, H14) ppm; **^{13}C NMR** (100 MHz, $CDCl_3$) δ = 166.2 (C1), 152.9 (C4), 144.9 (C3), 138.6 (C6), 128.8 (C2), 127.2 (C8), 126.2 (C11), 123.7 (C9), 120.1 (C10), 115.9 (C7), 84.2 (C13), 83.8 (C13), 25.0 (C14), 24.9 (C14) ppm; **^{11}B NMR** (128 MHz, $CDCl_3$) δ = 31.05 ppm; **IR** (ATR): ῡ = 631, 670, 723, 753, 835, 851, 911, 983, 1009, 1098, 1135, 1167, 1214, 1259, 1283, 1302, 1322, 1371, 1389, 1444, 1478, 1490, 1672, 2977 cm^{-1}; **HRMS** (ESI) $[M+Na]^+$ Calculated mass for $C_{29}H_{35}B_2NO_5Na$: 522.2593; Mass found: 522.2594.

(*E*)-Buta-1, 3-diene-1, 1, 4-triyltriboronic acid, pinacol ester (S26)

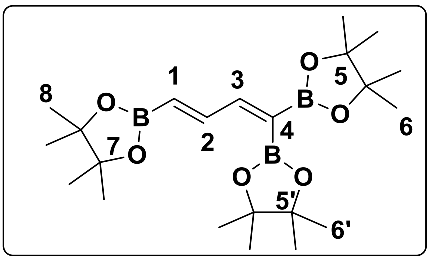

To a solution of *O*-trimethylsilylpropargyl alcohol (7.2 mL, 50 mmol) in DCM (10 mL), pinacolborane (4.6 mL, 31 mmol, 1.55 equiv.) was added dropwise at 0 °C under an argon atmosphere. The reaction mixture was allowed to warm to ambient temperature and stirred for 20 h. The solvent was removed in vacuo and MeOH (10 mL) was added, followed by citric acid (4.19 g, 20 mmol, 1 equiv.). After stirring for 10 min, the mixture was concentrated in vacuo, diluted with diethyl ether (50 mL), and washed with aqueous $NaHCO_3$ (50 mL). The combined organic layers were dried over Na_2SO_4 and concentrated under reduced pressure.

The crude residue was dissolved in DCM (10 mL), and Dess–Martin periodinane (11.9 g, 29 mmol, 2.9 equiv.) was added. The resulting white slurry was stirred for 2 h. The reaction mixture was concentrated in vacuo, filtered through a pad of Celite, and rinsed with hexane/diethyl ether (10/1, 100 mL). The resulting (*E*)-(3-oxoprop-1-en-1-yl)boronic acid pinacol ester was obtained and used in the next step without further purification.

To a solution of PPh_3 (11.8 g, 45 mmol, 4.5 equiv.) in DCM (50 mL), CBr_4 (8.29 g, 25 mmol, 2.5 equiv.) in DCM (20 mL) was added slowly at 0˚C under an argon atmosphere. After stirring for 30 minutes a solution of (*E*)-(3-oxoprop-1-en-1-yl)boronic acid, pinacol ester (1.82 g, 10 mmol, 1 equiv.) in DCM (10 mL) was added. The reaction was stirred for 1 h at ambient temperature then filtered through celite, washed with aqueous $NaHCO_3$ (50 mL), brine (50 mL). The combined organic phase was dried over Na_2SO_4, concentrated under reduced pressure. The crude was passed through a silica plug without further purification.

Prepared according to General Procedure **F**, the above crude was converted to **S26** yielding a yellow oil (2.42 g, 56%), after purification by flash column chromatography (SiO_2, 0→10% EtOAc/n-hexane).

***R**f* (10% EtOAc/*n*-hexane) = 0.25; **^{1}H NMR** (400 MHz, $CDCl_3$) δ = 7.36 – 7.27 (m, 2H, H3+H2), 5.75 (dt, *J* = 16.9, 6.2 Hz, 1H, H1), 1.33 (s, 12H, H6/6'/8), 1.25 (s, 12H, H6/6'/8), 1.25 (s, 12H, H6/6'/8) ppm; **^{13}C NMR** (100 MHz, $CDCl_3$) δ = 157.6 (C2), 150.1 (C3), 83.7 (C5/5'/7), 83.4 (C5/5'/7), 83.3 (C5/5'/7), 25.2 (C6/6'/8), 25.0 (C6/6'/8), 24.8 (C6/6'/8) ppm; **^{11}B NMR** (128 MHz, $CDCl_3$) δ = 30.80 ppm; **IR** (ATR): ṽ = 663, 743, 848, 900, 960, 987, 1010, 1121, 1174, 1210, 1283, 1320, 1371, 1389, 1508, 2375, 2931, 2978, 3567, 3678, 3735, 3747 cm^{-1}; **HRMS** (ESI) $[M+Na]^+$ Calculated mass for $C_{22}H_{39}B_3O_6Na$:455.2918; Mass found:455.2971.

(*E*)-(5-(4-(6-Fluorobenzo[d]isoxazol-3-yl)piperidin-1-yl)-5-oxopenta-1, 3-diene-1, 1-diyl)diboronic acid, pinacol ester (S27)

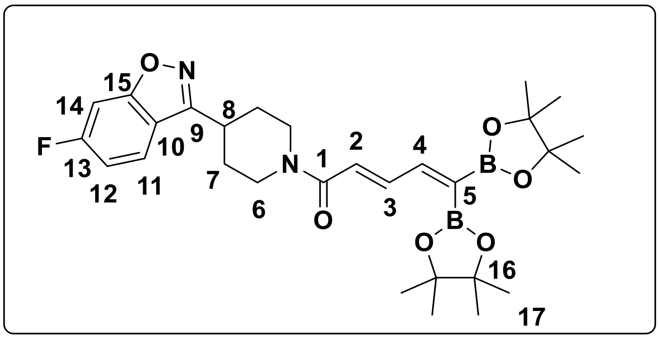

Prepared according to General Procedure **F**, **S15** (916 mg, 2 mmol) was converted to **S27** yielding a yellow solid (508 mg, 46%), after purification by flash column chromatography (SiO_2, 0→100% EtOAc/*n*-hexane).

***R**f* (EtOAc) = 0.35; **^{1}H NMR** (400 MHz, $CDCl_3$) δ = 7.67 – 7.57 (m, 2H, H3+H11), 7.40 (d, *J* = 11.4 Hz, 1H, H4), 7.25 (m, 1H, H14), 7.07 (td, *J* = 8.8, 2.1 Hz, 1H, H12), 6.54 (d, *J* = 14.6 Hz, 1H, H2), 4.69 (m, 1H, H6), 4.12 (m, 1H, H6), 3.35 (m, 2H, H6+H8), 2.97 (m, 1H, H8), 2.13 (m, 2H, H7), 1.98 (m, 2H, H7), 1.33 (s, 12H), 1.25 (s, 12H) ppm; **^{13}C NMR** (100 MHz, $CDCl_3$) δ = 165.5 (C1), 164.3 (d, *J* = 251.0 Hz, C13), 164.1 (d, *J* = 13.5 Hz, C15), 160.3 (C9), 154.2 (C4), 142.6 (C3), 125.6 (C2), 122.4 (d, *J* = 11.1 Hz, C11),

117.2 (d, J = 1.3 Hz, C10), 112.8 (d, J = 25.3 Hz, C12), 97.7 (d, J = 26.8 Hz, C14), 83.9 (C16), 83.5 (C16), 45.8 (C6), 42.0 (C6), 34.5 (C8), 30.8 (C7), 25.0 (C17), 24.9 (C17) ppm; **^{19}F NMR** (376 MHz, $CDCl_3$) δ = -109.14 (td, J = 8.6, 4.9 Hz) ppm; **^{11}B NMR** (128 MHz, $CDCl_3$) δ = 31.69 ppm; **IR** (ATR): ῦ = 660, 742, 848, 959, 986, 1009, 1122, 1174, 1204, 1257, 1284, 1342, 1371, 1508, 1566, 1714, 2931, 2977, 3198 cm^{-1}; **HRMS** (ESI) $[M+Na]^+$ Calculated mass for $C_{29}H_{39}B_2FN_2O_6Na$: 575.2870; Mass found: 575.2907.

(*E*)-(5-((5-(2, 5-Dimethylphenoxy)-2, 2-dimethylpentyl)oxy)-5-oxopenta-1, 3-diene-1, 1-diyl)diboronic acid, pinacol ester (S28)

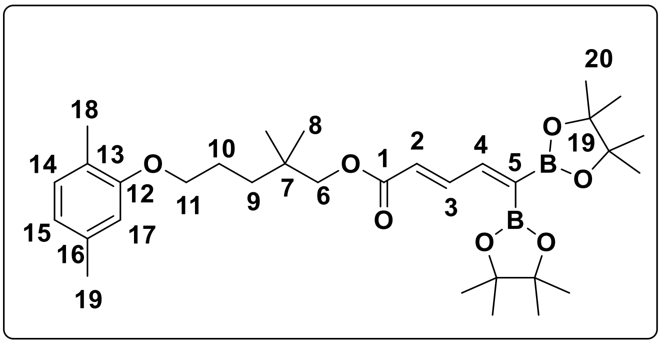

Prepared according to General Procedure **F**, **S16** (948 mg, 2 mmol) was converted to **S28** yielding a yellow solid (921 mg, 81%), after purification by flash column chromatography (SiO_2, 0→5% EtOAc/*n*-hexane).

R$_f$ (10% EtOAc/*n*-hexane) = 0.30; **^{1}H NMR** (400 MHz, $CDCl_3$) δ = 7.67 (dd, J = 15.2, 11.4 Hz, 1H, H3), 7.36 (d, J = 11.4 Hz, 1H, H4), 7.00 (d, J = 7.4 Hz, 1H, H14), 6.65 (d, J = 7.5 Hz, 1H, H15), 6.60 (d, J = 1.7 Hz, 1H, H17), 6.03 (d, J = 15.3 Hz, 1H, H2), 3.96 – 3.87 (m, 4H, H6+H11), 2.30 (s, 3H, H19), 2.16 (s, 3H, H18), 1.77 (ddd, J = 12.1, 8.6, 5.6 Hz, 2H, H10), 1.52 – 1.43 (m, 2H, H9), 1.31 (s, 12H, H20), 1.26 (s, 12H, H20), 0.99 (s, 6H, H8) ppm; **^{13}C NMR** (100 MHz, $CDCl_3$) δ = 167.0 (C1), 157.1 (C12), 153.4 (C4), 144.2 (C3), 136.6 (C16), 130.4 (C14), 126.1 (C2), 123.7 (C13), 120.8 (C15), 112.1 (C17), 83.9 (C19), 83.6 (C19), 72.4 (C6), 68.4 (C11), 35.6 (C9), 33.8 (C7), 24.91 (C20), 24.88 (C20), 24.4 (C8), 24.2 (C10), 21.5 (C19), 15.9 (C18) ppm; **^{11}B NMR** (128 MHz, $CDCl_3$) δ = 30.49 ppm; **IR** (ATR): ῦ =670, 716, 736, 803, 852, 984, 1009, 1128, 1167, 1230, 1253, 1343, 1371, 1389, 1475, 1508, 1566, 1658, 1712, 2312, 2375, 2977 cm^{-1}; **HRMS** (ESI) $[M+Na]^+$ Calculated mass for $C_{32}H_{50}B_2O_7Na$: 591.3635; Mass found: 591.3657.

(*E*)-(5-(4-(2-((2, 4-Dimethylphenyl)thio)phenyl)piperazin-1-yl)-5-oxopenta-1, 3-diene-1, 1-diyl)diboronic acid, pinacol ester (S29)

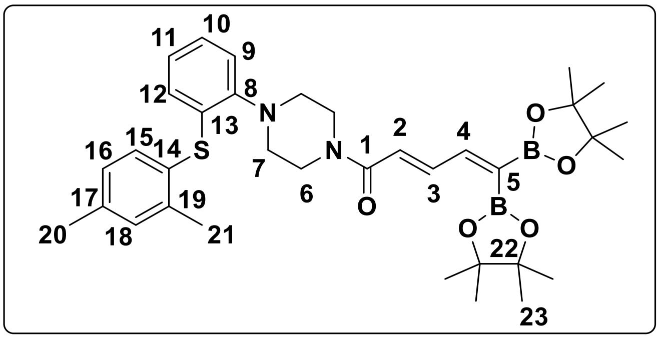

Prepared according to General Procedure **F**, **S17** (1068 mg, 2 mmol) was converted to **S29** yielding a yellow solid (958 mg, 76%), after purification by flash column chromatography (SiO_2, 0→30% EtOAc/*n*-hexane).

R$_f$ (30% EtOAc/*n*-hexane) = 0.35; **^{1}H NMR** (400 MHz, $CDCl_3$) δ = 7.62 (dd, J = 14.6, 11.5 Hz, 1H, H3), 7.40 (d, J = 11.5 Hz, 1H, H4), 7.36 (d, J = 7.8 Hz, 1H, H15), 7.17 – 7.14 (m, 1H, H18), 7.11 – 6.99 (m, 3H, H11+H16+H12), 6.89 (td, J = 7.4, 1.7 Hz, 1H, H10), 6.59 – 6.50 (m, 2H, H2+H9), 3.88 (m, 2H, H6/7), 3.74 (m, 2H, H6/7), 3.07 (t, J = 4.9 Hz, 4H, H6/7), 2.36 (s, 3H, H21), 2.32 (s, 3H, H20), 1.33 (s, 12H, H23), 1.26 (s, 12H, H23) ppm; **^{13}C NMR** (100 MHz, $CDCl_3$) δ = 165.5 (C1), 154.2 (C4), 148.5 (C8), 142.5 (C3), 142.4 (C17), 139.4 (C14), 136.1 (C15), 134.7 (C13), 131.9 (C18), 128.0 (C16), 127.8 (C19), 126.7 (C9), 125.8 (C11), 125.6 (C2), 125.1 (C10), 120.3 (C12), 83.9 (C22), 83.49 (C22), 52.2 (C6/7), 51.5 (C6/7), 46.4 (C6/7), 42.6 (C6/7), 25.0 (C23), 24.9 (C23), 21.3 (C21), 20.7 (C20) ppm; **^{11}B NMR** (128 MHz, $CDCl_3$) δ = 31.05 ppm; **IR** (ATR): ῦ = 618, 674, 684, 729, 760, 815, 851, 917, 983, 1009, 1032, 1137, 1220, 1340, 1371, 1389, 1439,

1470, 1579, 1602, 1639, 1702, 2976 cm^{-1}; **HRMS** (ESI) $[M+Na]^+$ Calculated mass for $C_{35}H_{48}B_2N_2SO_5Na$: 653.3362; Mass found: 653.3428.

Experimental Set Up for Photoreactions

The [2 + 2] photocycloaddition reactions were carried out using a Kessil PR160L-440 (440 mm) LED lamp with the producer´s power settings.[15] The reaction vial was placed in the middle on a stirring plate (Roth Rotilabo-Mini-Magnetrührer M3 Stirring speed 550 rpm). To regulate temperature, a fan was used for cooling. For slow addition, Harvard Pump Model 11 Elite Pump Syringe Pump was used.

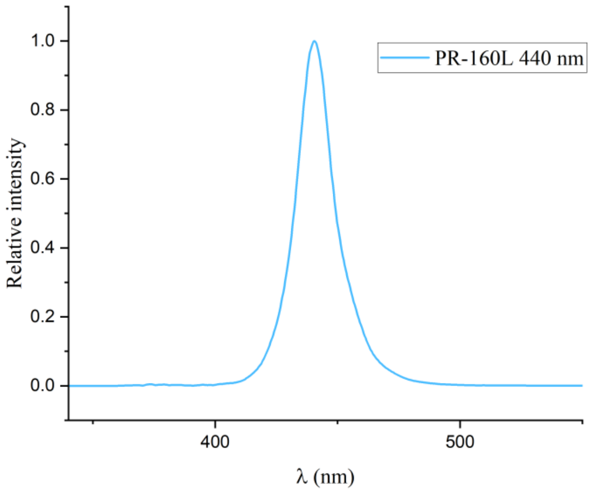

Figure S1: Emission spectra of Kessil PR160L-440.

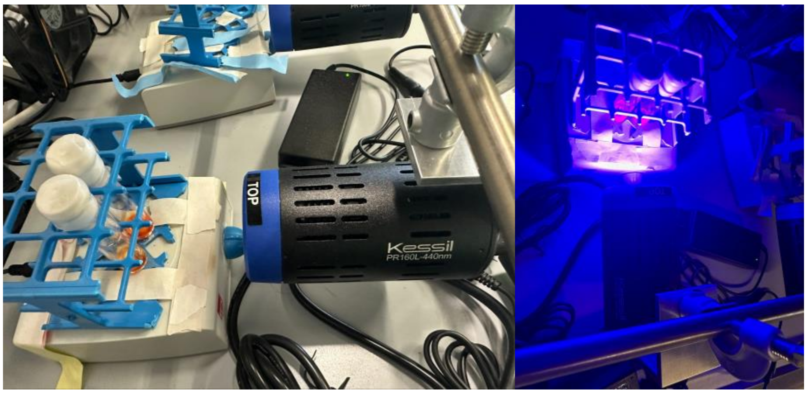

Figure S2: Reaction set up for 2 + 2 cycloaddition reaction; Kessil PR160L-440.

Reaction Optimization of [2 + 2] cycloaddition

Test reaction on 2:

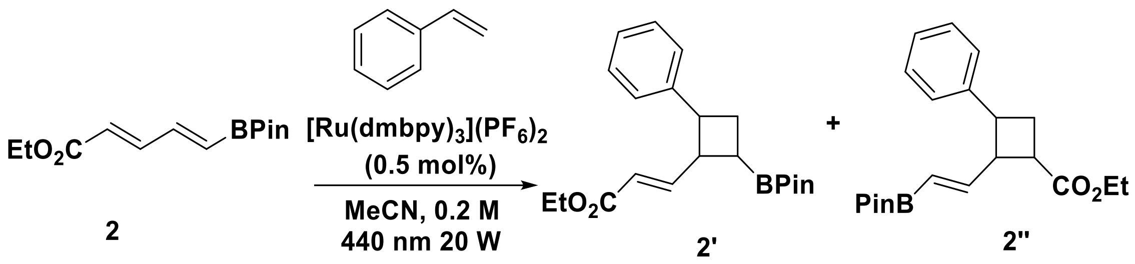

To an oven-dried 5 mL microwave vial, $[Ru(dmbpy)_3](PF_6)_2$ (0.9 mg, 0.5 mol%) was added. The vial was sealed with a septum and purged with nitrogen before the sequential addition of styrene (4 mmol, 20 equiv.) and deoxygenated MeCN (0.2 mL) via syringe. Using a syringe pump, a solution of **2** (0.2 mmol dissolved in 0.8 mL deoxygenated MeCN) was added at a rate of 0.001 mL/min to the styrene and sensitizer solution while under light irradiation with stirring (440 nm, 20 W). After the completion of addition, the reaction mixture was stirred for an additional 1 h under irradiation. Then internal standard (1,3,5-trimethoxybenzene), as a solution in MeCN, was added to record NMR yield. Crude NMR analysis against the internal standard revealed the [2 + 2] cycloaddition occurred to afford a mixture of **2'** and **2''** (NMR yield 70%, rr = 1:1).

Table S1. Photosensitizers screening[a]

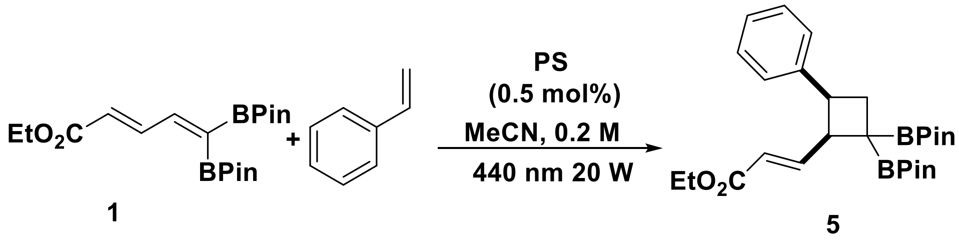

entry	Photosensitizer	E_T[22]	5[b][c]
1	$Ir(ppy)_3$	55.2 kcal/mol	87%
2	$[Ir(ppy)_2(dtbpy)]PF_6$	49.2 kcal/mol	87%
3	$Ir(dmppy)_2(dtbbpy)PF_6$	47.9 kcal/mol	87%
4	$[Ru(phen)_3]Cl_2$	46.8 kcal/mol	87%
5	$[Ru(dmbpy)_3](PF_6)_2$	45.3 kcal/mol	87%
6[d]	$[Ru(dmbpy)_3](PF_6)_2$	45.3 kcal/mol	76%
7[e]	-	-	35%

[a] Standard conditions: **1** (0.1 mmol, 1 equiv.), styrene (5 equiv.), photosensitizer (0.5 mol%), 20 W 440 nm Kessil lamp, MeCN (0.2 M), rt, 16 h; [b] Determined by ^1H NMR spectroscopy against a known internal standard (1, 3, 5-trimethoxybenzene); [c] d.r.= 2.5:1; [d] under air; [e] without photosensitizer, under the irradiation of 40 W 370 nm;

Table S2. Effect of concentration[a]

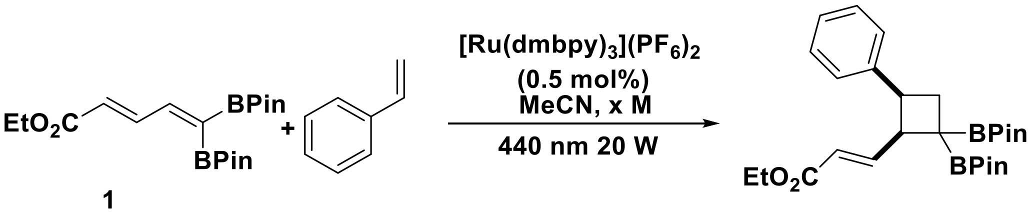

entry	x M	5[b][c]
1	0.05	28%
2	0.1	64%
3	0.2	87%

[a] Standard conditions: **1** (0.1 mmol, 1 equiv.), styrene (5 equiv.), photosensitizer (0.5 mol%), 20 W 440 nm Kessil lamp, MeCN (x M), rt, 16 h; [b] Determined by ^{1}H NMR spectroscopy against a known internal standard (1, 3, 5-trimethoxybenzene); [c] d.r.= 2.5:1.

Table S3. Effect of Lewis acid[a]

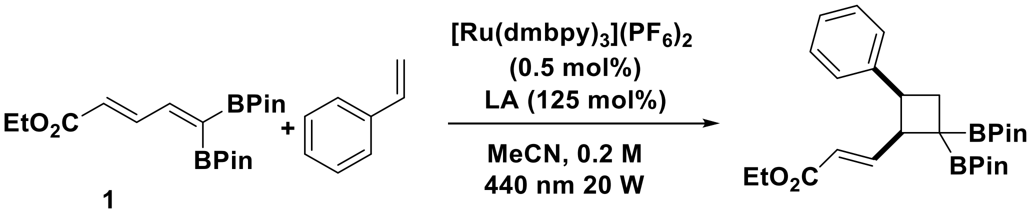

entry	Lewis acid	5[b][c]
1	$Gd(OTf)_3$	20%
2	$La(OTf)_3$	81%
3	$Al(OTf)_3$	43%

[a] Standard conditions: **1** (0.1 mmol, 1 equiv.), styrene (5 equiv.), photosensitizer (0.5 mol%), LA (125 mol%), 20 W 440 nm Kessil lamp, MeCN (0.2 M), rt, 16 h; [b] Determined by ^{1}H NMR spectroscopy against a known internal standard (1, 3, 5-trimethoxybenzene); [c] d.r.= 2.5:1.

Substrate scope of [2 + 2] cycloaddition

General Procedure G: Photosensitized Crossed [2 + 2]-Cycloadditions of dienes and styrenes

To an oven-dried 5 mL microwave vial $[Ru(dmbpy)_3](PF_6)_2$ (0.9 mg, 0.5 mol%) and the specified diene (0.2 mmol, 1 equiv.) were added. The vial was sealed with a septum and purged with nitrogen before the sequential addition of the specified styrene (1 mmol, 5 equiv.) and deoxygenated MeCN (1 mL, 0.2 M) via syringe. The reaction mixture was stirred under light irradiation (440 nm, 20 W) for 16 h. After completion, internal standard (1, 3, 5-trimethoxybenzene), as a solution in MeCN, was added. The crude residue was purified by flash column chromatography (SiO_2, specified combination of solvents) to afford a mixture of diastereomers. Then a second purification was carried out to isolate the major diastereomer. Borylated products purified by SiO_2 were exposed to the minimum amount of SiO_2 for as little time as possible to limit degradation.

Failed examples:

Coupling partners

a) no observed reactivity

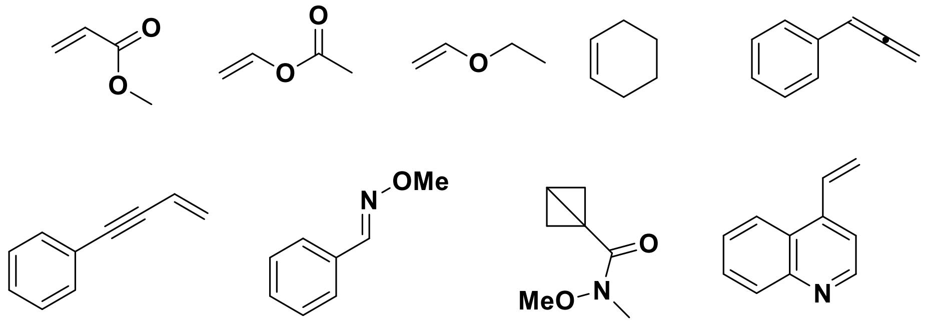

---

b) reactivity observed but with poor site-selectivity

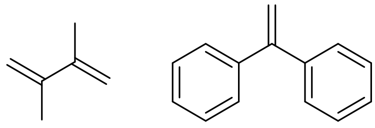

65%, r.r.=2:1 85%, r.r.=3:1

The poor site-selectivity may be due to the poor π–π Stacking Interaction with the diene.

(*E*)-(2-(3-Ethoxy-3-oxoprop-1-en-1-yl)-3-phenylcyclobutane-1, 1-diyl)diboronic acid, pinacol ester (5)

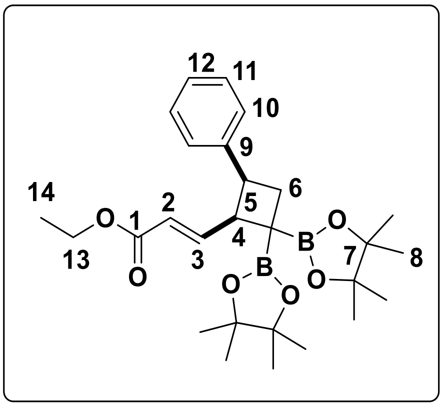

Prepared according to General Procedure **G**, **1** (75.6 mg, 0.2 mmol) and styrene (115 μL, 1 mmol), in the presence of [Ru(dmbpy)$_3$](PF$_6$)$_2$ (0.9 mg, 0.5 mol%), were converted to **5** (NMR yield 87%, d.r.=2.5:1), yielding a white solid (73 mg, 76%, d.r.=2.5:1), after purification by flash column chromatography (SiO$_2$, 10% EtOAc/*n*-hexane).

The major *syn*-diastereomer was isolated by a second column chromatography (SiO$_2$, 0%→10% EtOAc/*n*-hexane) as a white solid (38 mg, 40%).

R*f* (10% EtOAc/*n*-hexane) = 0.30; **^{1}H NMR** (400 MHz, CDCl$_3$) δ = 7.23 (t, *J* = 7.6 Hz, 2H, H11), 7.11 (t, *J* = 7.4 Hz, 1H, H12), 7.06 (d, *J* = 8.1 Hz, 2H, H10), 6.89 (dd, *J* = 15.4, 11.4 Hz, 1H, H3), 5.78 (d, *J* = 15.3 Hz, 1H, H2), 4.04 (q, *J* = 7.1 Hz, 2H, H13), 3.95 (dt, *J* = 11.2, 8.4 Hz, 1H, H5), 3.70 (ddd, *J* = 11.2, 8.2, 2.7 Hz, 1H, H4), 2.95 (t, *J* = 10.9 Hz, 1H, H6), 2.50 (ddd, *J* = 10.8, 8.4, 2.8 Hz, 1H, H6), 1.30 (s, 6H, H8), 1.29 (s, 6H, H8), 1.21 (s, 6H, H8), 1.19 – 1.13 (m, 9H, H8+H14) ppm; **^{13}C NMR** (100 MHz, CDCl$_3$) δ = 166.2 (C1), 149.5 (C3), 141.5 (C9), 128.1 (C11), 127.2 (C10), 125.9 (C12), 122.2 (C2), 83.8 (C7), 83.6 (C7), 59.8 (C13), 49.0 (C4), 43.1 (C5), 28.8 (C6), 24.9 (C8), 24.9 (C8), 24.8 (C8), 24.6 (C8), 14.4 (C14) ppm; **^{11}B NMR** (128 MHz, CDCl$_3$) δ = 32.99 ppm; **IR** (ATR): ṽ = 669, 699, 733, 752, 798, 851, 911, 969, 983, 1036, 1076, 1128, 1137, 1167, 1213, 1231, 1271, 1316, 1339, 1369, 1508, 1602, 1649, 1712, 2375, 2861, 2928, 2980 cm^{-1}; **HRMS** (ESI) [M+Na]$^+$ Calculated mass for $C_{27}H_{40}B_2O_6Na$: 505.2903; Mass found: 505.2932.

(*E*)-(2-(3-Ethoxy-3-oxoprop-1-en-1-yl)-3-(*p*-tolyl)cyclobutane-1, 1-diyl)diboronic acid, pinacol ester (6)

Prepared according to General Procedure **G**, **1** (75.6 mg, 0.2 mmol) and 4-methylstyrene (132 μL, 1 mmol), in the presence of [Ru(dmbpy)$_3$](PF$_6$)$_2$ (0.9 mg, 0.5 mol%), were converted to **6** (NMR yield 70%, d.r.=2.7:1), yielding a white solid (64 mg, 65%, d.r.=2.7:1), after purification by flash column chromatography (SiO$_2$, 10% EtOAc/ *n*-hexane).

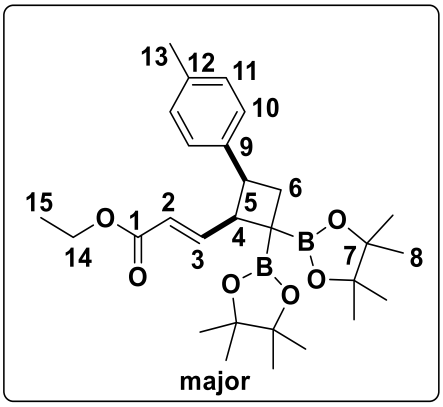

The major *syn*-diastereomer was isolated by a second column chromatography (SiO$_2$, 0%→10% EtOAc/ *n*-hexane) as a white solid (34 mg, 34%).

R*f* (10% EtOAc/*n*-hexane) = 0.30; **^{1}H NMR** (400 MHz, CDCl$_3$) δ = 7.11 – 7.00 (m, 2H, H11), 7.01 – 6.86 (m, 3H, H10+H3), 5.78 (d, *J* = 15.3 Hz, 1H, H2), 4.05 (m, 2H, H14), 3.90 (dt, *J* = 11.3, 8.4 Hz, 1H, H5), 3.67 (ddd, *J* = 11.2, 8.2, 2.7 Hz, 1H, H4), 2.92 (t, *J* = 10.9 Hz, 1H, H6), 2.48 (ddd, *J* = 10.8, 8.4, 2.8 Hz, 1H, H6), 2.27 (s, 3H, H13), 1.29 (s, 6H, H8), 1.29 (s, 6H, H8), 1.21 (s, 6H, H8), 1.17 (m, 9H, H8+H15) ppm; **^{13}C NMR** (100 MHz, CDCl$_3$) δ = 166.3 (C1), 149.6 (C3), 138.4 (C9), 135.3 (C12), 128.8 (C11), 127.1 (C10), 122.2 (C2), 83.8 (C7), 83.6 (C7), 59.8 (C14), 49.1 (C4), 42.9 (C5), 28.9 (C6), 24.9 (C8), 24.85 (C8), 24.80 (C8), 24.6 (C8), 21.2 (C13), 14.4 (C15) ppm; **^{11}B NMR** (128 MHz, CDCl$_3$) δ = 30.72 ppm; **IR** (ATR): ṽ = 670, 699, 811, 849, 981, 1039, 1138, 1164, 1215, 1257, 1306, 1339, 1369, 1607, 1648, 1715, 2375, 2977 cm^{-1}; **HRMS** (ESI) [M+Na]$^+$ Calculated mass for $C_{28}H_{42}B_2O_6Na$: 519.3060; Mass found: 519.3105.

(*E*)-(2-(3-Ethoxy-3-oxoprop-1-en-1-yl)-3-(4-methoxyphenyl)cyclobutane-1, 1-diyl)diboronic acid, pinacol ester (7)

Prepared according to General Procedure **G**, **1** (75.6 mg, 0.2 mmol) and 4-methoxystyrene (135 µL, 1 mmol), in the presence of $[Ru(dmbpy)_3](PF_6)_2$ (0.9 mg, 0.5 mol%), were converted to **7** (NMR yield 88%, d.r.=2.2:1), yielding a white solid (77 mg, 75%, d.r.=2.2:1), after purification by flash column chromatography (SiO_2, 10% EtOAc/*n*-hexane).

The major *syn*-diastereomer was isolated by a second column chromatography (SiO_2, 0%→10% EtOAc/ *n*-hexane) as a white solid (42 mg, 41%).

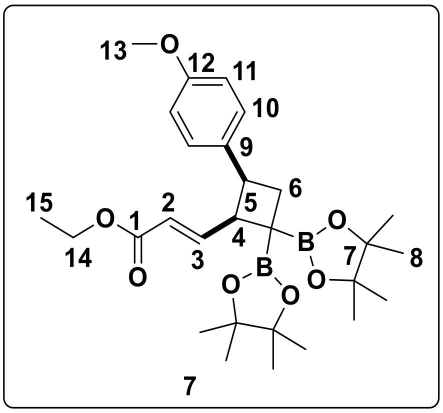

***R**$_f$* (10% EtOAc/*n*-hexane) = 0.25; **^{1}H NMR** (400 MHz, $CDCl_3$) δ = 7.00 – 6.95 (m, 2H, H10), 6.91 (dd, *J* = 15.4, 11.4 Hz, 1H, H3), 6.77 (d, *J* = 8.7 Hz, 2H, H11), 5.76 (d, *J* = 15.4 Hz, 1H, H2), 4.05 (q, *J* = 7.1 Hz, 2H, H14), 3.88 (dt, *J* = 11.2, 8.3 Hz, 1H, H5), 3.75 (s, 3H, H13), 3.65 (ddd, *J* = 11.2, 8.2, 2.7 Hz, 1H, H4), 2.90 (t, *J* = 10.9 Hz, 1H, H6), 2.47 (ddd, *J* = 10.8, 8.4, 2.8 Hz, 1H, H6), 1.29 (s, 6H, H8), 1.28 (s, 6H, H8), 1.21 (s, 6H, H8), 1.19 – 1.14 (m, 9H, H8+H15) ppm; **^{13}C NMR** (100 MHz, $CDCl_3$) δ = 166.3 (C1), 157.8 (C12), 149.6 (C3), 133.6 (C9), 128.2 (C10), 122.2 (C2), 113.5 (C11), 83.8 (C7), 83.6 (C7), 59.8 (C14), 55.3 (C13), 49.1 (C4), 42.6 (C5), 29.0 (C6), 24.9 (C8), 24.85 (C8), 24.78 (C8), 24.6 (C8), 14.4 (C15) ppm; **^{11}B NMR** (128 MHz, $CDCl_3$) δ = 35.89 ppm; **IR** (ATR): ῦ = 669, 693, 730, 795, 826, 848, 970, 981, 1033, 1098, 1138, 1165, 1250, 1303, 1339, 1369, 1513, 1648, 1714, 2375, 2977 cm^{-1};**HRMS** (ESI) $[M+Na]^+$ Calculated mass for $C_{28}H_{42}B_2O_7Na$: 535.3009; Mass found: 535.3019.

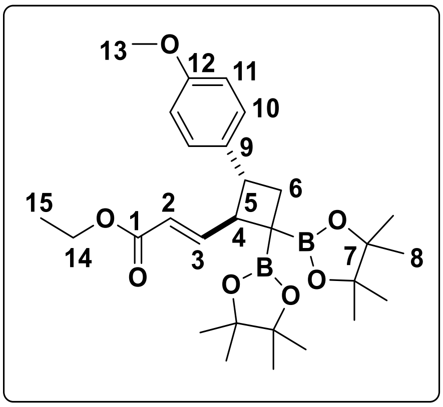

***R**$_f$* (10% EtOAc/*n*-hexane) = 0.27; **^{1}H NMR** (400 MHz, $CDCl_3$) δ = 7.21 (dd, *J* = 15.6, 7.6 Hz, 1H, H3), 7.13 – 7.08 (m, 2H, H10), 6.84 – 6.79 (m, 2H, H11), 5.78 (d, *J* = 16.8 Hz, 1H, H2), 4.16 (m, 2H, H14), 3.77 (s, 3H, H13), 3.53 (q, *J* = 9.3 Hz, 1H, H5), 3.34 (t, *J* = 8.5 Hz, 1H, H4), 2.59 (t, *J* = 9.4 Hz, 1H, H6), 2.14 (t, *J* = 10.0 Hz, 1H, H6), 1.30 – 1.16 (m, 27H, H8+H15) ppm; **^{13}C NMR** (100 MHz, $CDCl_3$) δ = 166.9 (C1), 158.1 (C12), 151.8 (C3), 136.6 (C9), 127.6 (C10), 120.0 (C2), 113.8 (C11), 83.6 (C7), 83.4 (C7), 60.1 (C14), 55.4 (C13), 49.0 (C4), 44.1 (C5), 30.6 (C6), 25.0 (C8), 24.9 (C8), 24.5 (C8), 14.4 (C15) ppm; **^{11}B NMR** (128 MHz, $CDCl_3$) δ = 34.20 ppm; **IR** (ATR): ῦ = 670, 696, 732, 779, 825, 849, 969, 1035, 1137, 1165, 1247, 1304, 1339, 1369, 1513, 1610, 1715, 2311, 2375, 2934, 2977 cm^{-1}; **HRMS** (ESI) $[M+Na]^+$ Calculated mass for $C_{28}H_{42}B_2O_7Na$: 535.3009; Mass found: 535.3052.

(*E*)-(3-(3', 4'-Dimethoxy-[1, 1'-biphenyl]-4-yl)-2-(3-ethoxy-3-oxoprop-1-en-1-yl)cyclobutane-1, 1-diyl)diboronic acid, pinacol ester (8)

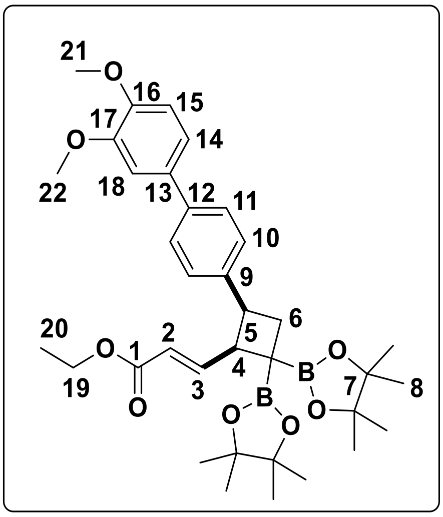

Prepared according to General Procedure **G**, **1** (75.6 mg, 0.2 mmol) and **S1** (240 mg, 1 mmol), in the presence of $[Ru(dmbpy)_3](PF_6)_2$ (0.9 mg, 0.5 mol%), were converted to **8** (NMR yield 68%, d.r.=2.1:1), yielding a white solid (64 mg, 52%, d.r.=2.1:1), after purification by flash column chromatography (SiO_2, 20% EtOAc/ *n*-hexane).

The major *syn*-diastereomer was isolated by a second column chromatography (SiO_2, 0%→20% EtOAc/ *n*-hexane) as a white solid (34 mg, 28%).

$\boldsymbol{R_f}$ (20% EtOAc/*n*-hexane) = 0.30; **^{1}H NMR** (400 MHz, $CDCl_3$) δ = 7.43 (d, *J* = 8.0 Hz, 2H, H11), 7.14 – 7.06 (m, 4H, H10+H14+H18), 7.00 – 6.89 (m, 2H, H3+H15), 5.82 (d, *J* = 15.4 Hz, 1H, H2), 4.05 (q, *J* = 7.1 Hz, 2H, H19), 3.99 – 3.95 (m, 1H, H5), 3.94 (s, 3H, H22), 3.91 (s, 3H, H21), 3.72 (ddd, *J* = 11.2, 8.2, 2.7 Hz, 1H, H4), 2.97 (t, *J* = 10.9 Hz, 1H, H6), 2.53 (ddd, *J* = 10.8, 8.5, 2.8 Hz, 1H, H6), 1.31 (s, 6H, H8), 1.30 (s, 6H, H8), 1.22 (s, 6H, H8), 1.17 (m, 9H, H8+H20) ppm; **^{13}C NMR** (100 MHz, $CDCl_3$) δ = 166.3 (C1), 149.4 (C3), 149.2 (C17), 148.5 (C16), 140.2 (C9), 138.6 (C12), 134.3 (C13), 127.6 (C10), 126.6 (C11), 122.3 (C2), 119.3 (C14), 111.5 (C15), 110.4 (C18), 83.8 (C7), 83.7 (C7), 59.9 (C19), 56.1 (C21/22), 56.0 (C21/22), 49.0 (C4), 42.8 (C5), 28.9 (C6), 24.93 (C8), 24.86 (C8), 24.8 (C8), 24.6 (C8), 14.4 (C20) ppm; **^{11}B NMR** (128 MHz, $CDCl_3$) δ = 34.05 ppm; **IR** (ATR): ῦ = 669, 700, 733, 765, 805, 829, 849, 880, 970, 981, 1026, 1138, 1167, 1215, 1251, 1304, 1339, 1369, 1503, 1526, 1603, 1648, 1656, 1714, 2375, 2977 cm^{-1}; **HRMS** (ESI) $[M+Na]^+$ Calculated mass for $C_{35}H_{48}B_2O_8Na$: 641.3427; Mass found: 641.3454.

(*E*)-(2-(3-Ethoxy-3-oxoprop-1-en-1-yl)-3-(4-fluorophenyl)cyclobutane-1, 1-diyl)diboronic acid, pinacol ester (9)

Prepared according to General Procedure **G**, **1** (75.6 mg, 0.2 mmol) and 4-fluorostyrene (120 μL, 1 mmol), in the presence of $[Ru(dmbpy)_3](PF_6)_2$ (0.9 mg, 0.5 mol%), were converted to **9** (NMR yield 75%, d.r.=2.4:1), yielding a white solid (71 mg, 71%, d.r.=2.4:1), after purification by flash column chromatography (SiO_2, 10% EtOAc/*n*-hexane).

The major *syn*-diastereomer was isolated by a second column chromatography (SiO_2, 0%→10% EtOAc/ *n*-hexane) as a white solid (42 mg, 42%).

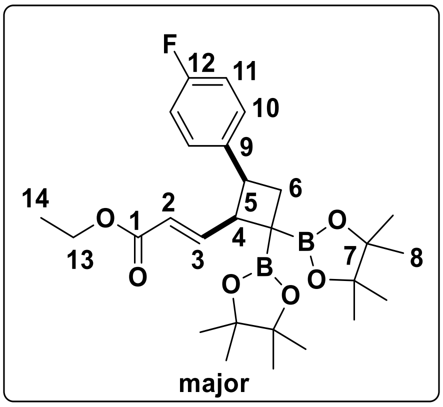

$\boldsymbol{R_f}$ (10% EtOAc/*n*-hexane) = 0.33; **^{1}H NMR** (400 MHz, $CDCl_3$) δ = 7.03 – 6.98 (m, 2H, H10), 6.94 – 6.79 (m, 3H, H11+H3), 5.77 (d, *J* = 15.4 Hz, 1H, H2), 4.05 (q, *J* = 7.1 Hz, 2H, H13), 3.90 (dt, *J* = 11.3, 8.4 Hz, 1H, H5), 3.66 (ddd, *J* = 11.1, 8.2, 2.7 Hz, 1H, H4), 2.90 (t, *J* = 10.9 Hz, 1H, H6), 2.49 (ddd, *J* = 10.8, 8.4, 2.8 Hz, 1H, H6), 1.29 (s, 6H, H8), 1.28 (s, 6H, H8), 1.20 (s, 6H, H8), 1.19 – 1.14 (m, 9H, H8+H14) ppm; **^{13}C NMR** (100 MHz, $CDCl_3$) δ = 166.2 (C1), 161.3 (d, *J* = 243.6 Hz, C12), 149.2 (C3), 137.2 (d, *J* = 3.2 Hz, C9)), 128.6 (d, *J* = 7.8 Hz, C10), 122.4 (C2), 114.9 (d, *J* = 21.1 Hz, C11), 83.8 (C7), 83.7 (C7), 59.9 (C13), 48.8 (C4), 42.40 (C5), 28.9 (C6), 24.9 (C8), 24.85 (C8), 24.76 (C8), 24.6 (C8), 14.4 (C14) ppm; **^{19}F NMR** (376 MHz, $CDCl_3$) δ = -117.48 (td, *J* = 8.9, 4.6 Hz) ppm; **^{11}B NMR** (128 MHz, $CDCl_3$) δ = 34.68 ppm; **IR** (ATR): ῦ = 669, 690, 730, 806, 829, 848, 918, 970, 1042, 1108,

1138, 1217, 1256, 1339, 1371, 1510, 1603, 1649, 1656, 1715, 2375, 2978 cm^{-1}; **HRMS** (ESI) $[M+Na]^+$ Calculated mass for $C_{27}H_{39}B_2FO_6Na$: 523.2809; Mass found: 523.2792.

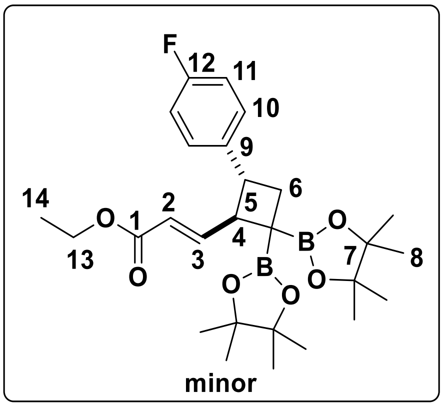

R_f (10% EtOAc/*n*-hexane) = 0.35; **^{1}H NMR** (400 MHz, $CDCl_3$) δ = 7.20 (dd, *J* = 15.6, 7.6 Hz, 1H, H3), 7.15 – 7.09 (m, 2H, H10), 6.99 – 6.91 (m, 2H, H11), 5.78 (dd, *J* = 15.6, 1.3 Hz, 1H, H2), 4.16 (m, 2H, H13), 3.56 (q, *J* = 9.3 Hz, 1H, H5), 3.33 (t, *J* = 8.5 Hz, 1H, H4), 2.61 (t, *J* = 9.4 Hz, 1H, H6), 2.13 (t, *J* = 9.9 Hz, 1H, H6), 1.26 (m, 15H, H8+H14), 1.23 (s, 6H, H8), 1.22 (s, 6H, H8) ppm; **^{13}C NMR** (100 MHz, $CDCl_3$) δ = 166.8 (C1), 161.5 (d, *J* = 243.8 Hz, C12), 151.4 (C3), 140.1 (d, *J* = 3.1 Hz, C9), 128.0 (d, *J* = 7.8 Hz, C10), 120.2 (C2), 115.1 (d, *J* = 21.0 Hz, C11), 83.7 (C7), 83.5 (C7), 60.2 (C13), 48.9 (C4), 44.0 (C5), 30.4 (C6), 24.98 (C8), 24.95 (C8), 24.6 (C8), 14.4 (C14) ppm; **^{19}F NMR** (376 MHz, $CDCl_3$) δ = -117.17 (ddd, *J* = 14.5, 9.1, 5.5 Hz) ppm; **^{11}B NMR** (128 MHz, $CDCl_3$) δ = 34.30 ppm; **IR** (ATR): ṽ = 670, 691, 735, 783, 821, 969, 994, 1022, 1036, 1069, 1095, 1109, 1129, 1138, 1159, 1190, 1233, 1273, 1302, 1327, 1340, 1369, 1511, 1603, 1639, 1711, 2375, 2977 cm^{-1}; **HRMS** (ESI) $[M+Na]^+$ Calculated mass for $C_{27}H_{39}B_2FO_6Na$: 523.2809; Mass found: 523.2844.

(*E*)-(3-(4-Bromophenyl)-2-(3-ethoxy-3-oxoprop-1-en-1-yl)cyclobutane-1, 1-diyl)diboronic acid, pinacol ester (10)

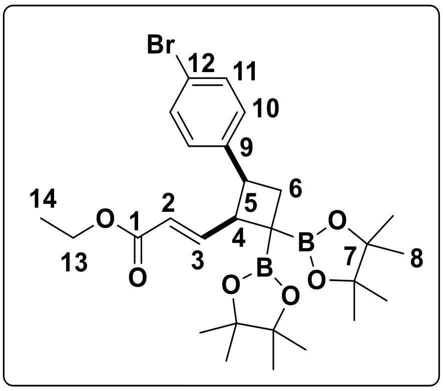

Prepared according to General Procedure **G**, **1** (75.6 mg, 0.2 mmol) and 4-bromostyrene (130 µL, 1 mmol), in the presence of $[Ru(dmbpy)_3](PF_6)_2$ (0.9 mg, 0.5 mol%), were converted to **10** (NMR yield 82%, d.r.=2.7:1), yielding a white solid (82 mg, 73%, d.r.=2.7:1), after purification by flash column chromatography (SiO_2, 10% EtOAc/ *n*-hexane).

The major *syn*-diastereomer was isolated by a second column chromatography (SiO_2, 0%→10% EtOAc/ *n*-hexane) as a white solid (52 mg, 46%).

R_f (10% EtOAc/*n*-hexane) = 0.30; **^{1}H NMR** (400 MHz, $CDCl_3$) δ = 7.34 (d, *J* = 8.5 Hz, 2H, H11), 6.92 (d, *J* = 7.6 Hz, 2H, H10), 6.83 (dd, *J* = 15.4, 11.5 Hz, 1H, H3), 5.78 (d, *J* = 15.4 Hz, 1H, H2), 4.06 (q, *J* = 7.1 Hz, 2H, H13), 3.87 (dt, *J* = 11.3, 8.4 Hz, 1H, H5), 3.67 (ddd, *J* = 11.2, 8.2, 2.7 Hz, 1H, H4), 2.88 (t, *J* = 10.9 Hz, 1H, H6), 2.48 (ddd, *J* = 10.8, 8.4, 2.7 Hz, 1H, H6), 1.29 (s, 6H, H8), 1.29 (s, 6H, H8), 1.20 (s, 6H, H8), 1.20 – 1.15 (m, 9H, H8+H14) ppm; **^{13}C NMR** (100 MHz, $CDCl_3$) δ = 166.1 (C1), 148.9 (C3), 140.6 (C9), 131.2 (C11), 129.0 (C10), 122.6 (C2), 119.8 (C12), 83.9 (C7), 83.7 (C7), 60.0 (C13), 48.7 (C4), 42.5 (C5), 28.7 (C6), 24.93 (C8), 24.86 (C8), 24.79 (C8), 24.7 (C8), 14.4 (C14) ppm; **^{11}B NMR** (128 MHz, $CDCl_3$) δ = 34 ppm; **IR** (ATR): ṽ = 670, 699, 815, 848, 970, 981, 1009, 1042, 1072, 1138, 1164, 1214, 1256, 1306, 1338, 1369, 1648, 1715, 2977 cm^{-1}; **HRMS** (ESI) $[M+Na]^+$ Calculated mass for $C_{27}H_{39}B_2O_6BrNa$: 583.2008; Mass found: 583.2038.

(*E*)-(3-(2-Bromophenyl)-2-(3-ethoxy-3-oxoprop-1-en-1-yl)cyclobutane-1, 1-diyl)diboronic acid, pinacol ester (11)

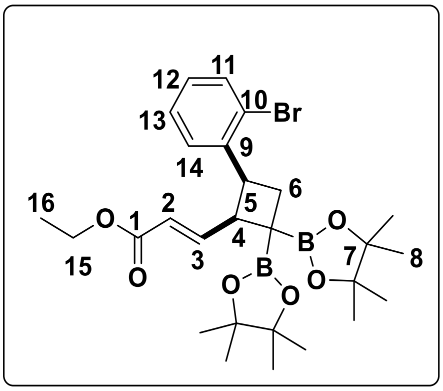

Prepared according to General Procedure **G**, **1** (75.6 mg, 0.2 mmol) and 2-bromostyrene (125 μL, 1 mmol), in the presence of $[Ru(dmbpy)_3](PF_6)_2$ (0.9 mg, 0.5 mol%), were converted to **11** (NMR yield 79%, d.r.=2.4:1), yielding a white solid (81 mg, 72%, d.r.=2.4:1), after purification by flash column chromatography (SiO_2, 10% EtOAc/ *n*-hexane).

The major *syn*-diastereomer was isolated by a second column chromatography (SiO_2, 0%→10% EtOAc/*n*-hexane) as a white solid (48 mg, 43%).

***R**f* (15% EtOAc/*n*-hexane) = 0.30; **^{1}H NMR** (400 MHz, $CDCl_3$) δ = 7.42 (dd, *J* = 7.9, 1.2 Hz, 1H, H11), 7.29 – 7.21 (m, 2H, H13+H14), 7.04 – 6.98 (m, 1H, H12), 6.79 (dd, *J* = 15.3, 11.3 Hz, 1H, H3), 5.81 (d, *J* = 15.3 Hz, 1H, H2), 4.08 – 3.97 (m, 3H, H5+H15), 3.92 (ddd, *J* = 11.1, 8.1, 2.9 Hz, 1H, H4), 3.01 (t, *J* = 10.9 Hz, 1H, H6), 2.42 (ddd, *J* = 10.7, 8.1, 2.9 Hz, 1H, H6), 1.30 (s, 12H, H8), 1.21 (s, 6H, H8), 1.17 (s, 6H, H8), 1.14 (t, *J* = 7.1 Hz, 3H, H16) ppm; **^{13}C NMR** (100 MHz, $CDCl_3$) δ = 166.3 (C1), 148.0 (C3), 140.2 (C9), 132.5 (C11), 128.7 (C14), 127.9 (C12), 127.2 (C13), 124.0 (C10), 122.5 (C2), 83.8 (C7), 83.7 (C7), 59.8 (C15), 48.9 (C4), 44.3 (C5), 27.3 (C6), 24.9 (C8), 24.8 (C8), 24.6 (C8), 14.4 (C16) ppm; **^{11}B NMR** (128 MHz, $CDCl_3$) δ = 34.50 ppm; **IR** (ATR):ῦ = 669, 699, 733, 752, 851, 970, 983, 1036, 1137, 1165, 1214, 1231, 1256, 1270, 1313, 1339, 1369, 1470, 1508, 1566, 1602, 1649, 1712, 2375, 2978 cm^{-1}; **HRMS** (ESI) $[M+Na]^+$ Calculated mass for $C_{27}H_{39}B_2O_6BrNa$: 583.2008; Mass found: 583.2038.

(*E*)-(3-(3-Cyanophenyl)-2-(3-ethoxy-3-oxoprop-1-en-1-yl)cyclobutane-1, 1-diyl)diboronic acid, pinacol ester (12)

Prepared according to General Procedure **G**, **1** (75.6 mg, 0.2 mmol) and **S3** (129 mg, 1 mmol), in the presence of $[Ru(dmbpy)_3](PF_6)_2$ (0.9 mg, 0.5 mol%), were converted to **12** (NMR yield 73%, d.r.=2.5:1), yielding a yellow oil (67 mg, 66%, d.r.=2.5:1), after purification by flash column chromatography (SiO_2, 15% EtOAc/*n*-hexane).

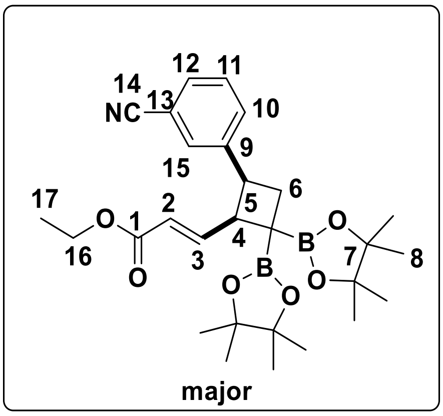

The major *syn*-diastereomer was isolated by a second column chromatography (SiO_2, 0%→15% EtOAc/*n*-hexane) as a yellow oil (40 mg, 39%).

***R**f* (15% EtOAc/*n*-hexane) = 0.25; **^{1}H NMR** (600 MHz, $CDCl_3$) δ = 7.41 (d, *J* = 7.6 Hz, 1H, H12), 7.36 – 7.31 (m, 2H, H15+H11), 7.28 – 7.25 (m, 1H, H10), 6.76 (dd, *J* = 15.4, 11.5 Hz, 1H, H3), 5.79 (d, *J* = 15.3 Hz, 1H, H2), 4.05 (m, 2H, H16), 3.97 – 3.91 (m, 1H, H5), 3.70 (ddd, *J* = 11.2, 8.3, 2.7 Hz, 1H, H4), 2.90 (t, *J* = 10.9 Hz, 1H, H6), 2.51 (ddd, *J* = 10.8, 8.4, 2.7 Hz, 1H, H6), 1.29 (s, 6H, H8), 1.28 (s, 6H, H8), 1.20 (s, 6H, H8), 1.19 – 1.14 (m, 9H, H8+H17) ppm; **^{13}C NMR** (100 MHz, $CDCl_3$) δ = 166.0 (C1), 148.2 (C3), 143.0 (C9), 131.8 (C10), 130.8 (C15), 129.8 (C12), 128.9 (C11), 122.9 (C2), 119.2 (C14), 112.2 (C13), 83.9 (C7), 83.8 (C7), 60.0 (C16), 48.4 (C4), 42.2 (C5), 28.2 (C6), 24.9 (C8), 24.8 (C8), 24.7 (C8), 24.6 (C8), 14.3 (C17) ppm; **^{11}B NMR** (192 MHz, $CDCl_3$) δ = 33.87 ppm; **IR** (ATR): ῦ = 670, 717, 743, 806, 847, 904, 970, 992, 1046, 1086, 1135, 1168, 1194, 1214, 1266, 1303, 1339, 1369, 1508, 1712, 2375, 2931, 2977 cm^{-1}; **HRMS** (ESI) $[M+Na]^+$ Calculated mass for $C_{28}H_{39}B_2NO_6Na$: 530.2856; Mass found: 530.2905.

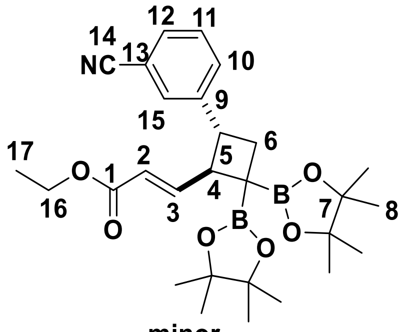

R$_f$ (15% EtOAc/*n*-hexane) = 0.27; **^{1}H NMR** (600 MHz, $CDCl_3$) δ = 7.52 – 7.46 (m, 2H, H12+H15), 7.42 – 7.34 (m, 2H, H10+H11), 7.19 (dd, *J* = 15.6, 7.6 Hz, 1H, H3), 5.78 (dd, *J* = 15.6, 1.3 Hz, 1H, H2), 4.24 – 4.07 (m, 2H, H16), 3.61 (q, *J* = 9.3 Hz, 1H, H5), 3.35 (t, *J* = 8.5 Hz, 1H, H4), 2.64 (t, *J* = 9.5 Hz, 1H, H6), 2.14 (t, *J* = 9.9 Hz, 1H, H6), 1.29 – 1.25 (m, 15H, H8+H17), 1.24 (s, 6H, H8), 1.23 (s, 6H, H8) ppm; **^{13}C NMR** (100 MHz, $CDCl_3$) δ = 166.3 (C1), 150.7 (C3), 145.7 (C9), 131.2 (C10), 130.2 (C15), 130.0 (C12), 129.2 (C11), 120.5 (C2), 119.2 (C14), 112.5 (C13), 83.8 (C7), 83.6 (C7), 60.3 (C16), 48.4 (C4), 43.9 (C5), 30.0 (C6), 25.0 (C8), 24.9 (C8), 24.6 (C8), 14.4 (C17) ppm; **^{11}B NMR** (192 MHz, $CDCl_3$) δ = 33.56 ppm; **IR** (ATR): ṽ = 610, 637, 670, 717, 796, 806, 847, 904, 970, 992, 1046, 1086, 1135, 1170, 1194, 1215, 1266, 1303, 1339, 1369, 1508, 1602, 1712, 2225, 2375, 2931, 2977 cm^{-1}; **HRMS** (ESI) $[M+Na]^+$ Calculated mass for $C_{28}H_{39}B_2NO_6Na$:530.2856; Mass found:530.2899.

(*E*)-(3-(Benzofuran-5-yl)-2-(3-ethoxy-3-oxoprop-1-en-1-yl)cyclobutane-1, 1-diyl)diboronic acid, pinacol ester (13)

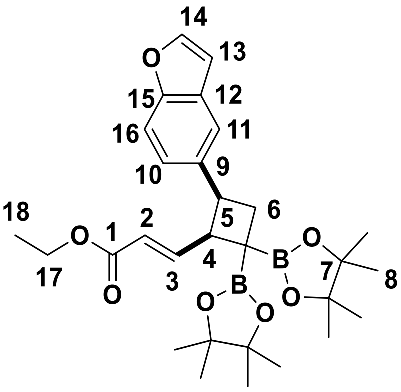

Prepared according to General Procedure **G**, **1** (75.6 mg, 0.2 mmol) and **S4** (144 mg, 1 mmol), in the presence of $[Ru(dmbpy)_3](PF_6)_2$ (0.9 mg, 0.5 mol%), were converted to **13** (NMR yield 84%, d.r.=2.5:1), yielding a white solid (81 mg, 78%, d.r.=2.5:1), after purification by flash column chromatography (SiO_2, 10% EtOAc/ *n*-hexane).

The major *syn*-diastereomer was isolated by a second column chromatography (SiO_2, 0%→10% EtOAc/*n*-hexane) as a white solid (49 mg, 47%).

R$_f$ (10% EtOAc/*n*-hexane) = 0.25; **^{1}H NMR** (400 MHz, $CDCl_3$) δ = 7.55 (d, *J* = 2.2 Hz, 1H, H14), 7.35 (d, *J* = 8.5 Hz, 1H, H16), 7.32 – 7.28 (m, 1H, H11), 6.97 (dd, *J* = 8.6, 1.8 Hz, 1H, H10), 6.90 (dd, *J* = 15.3, 11.4 Hz, 1H, H3), 6.69 (dd, *J* = 2.2, 0.9 Hz, 1H, H13), 5.79 (d, *J* = 15.4 Hz, 1H, H2), 4.01 (q, *J* = 7.1 Hz, 3H, H5+H17), 3.72 (td, *J* = 8.4, 4.2 Hz, 1H, H4), 3.00 (t, *J* = 10.9 Hz, 1H, H6), 2.55 (ddd, *J* = 10.8, 8.4, 2.8 Hz, 1H, H6), 1.31 (s, 6H, H8), 1.30 (s, 6H, H8), 1.21 (s, 6H, H8), 1.18 (s, 6H, H8), 1.13 (t, *J* = 7.1 Hz, 3H, H18) ppm; **^{13}C NMR** (176 MHz, $CDCl_3$) δ = 166.2 (C1), 153.6 (C15), 149.5 (C3), 145.0 (C14), 136.1 (C9), 127.3 (C12), 123.9 (C10), 122.3 (C2), 119.4 (C11), 110.9 (C16), 106.7 (C13), 83.8 (C7), 83.6 (C7), 59.8 (C17), 49.2 (C4), 43.1 (C5), 29.1 (C6), 24.94 (C8), 24.87 (C8), 24.8 (C8), 24.6 (C8), 14.4 (C18) ppm; **^{11}B NMR** (128 MHz, $CDCl_3$) δ = 34.72 ppm; **IR** (ATR): ṽ = 647, 669, 730, 769, 848, 913, 1032, 1108, 1138, 1165, 1214, 1259, 1339, 1371, 1471, 1508, 1648, 1656, 1714, 2375, 2978 cm^{-1}; **HRMS** (ESI) $[M+Na]^+$ Calculated mass for $C_{29}H_{40}B_2O_7Na$: 545.2852; Mass found: 545.2914.

(*E*)-(2-(3-Ethoxy-3-oxoprop-1-en-1-yl)-3-(1-methyl-1*H*-indol-3-yl)cyclobutane-1, 1-diyl)diboronic acid, pinacol ester (14)

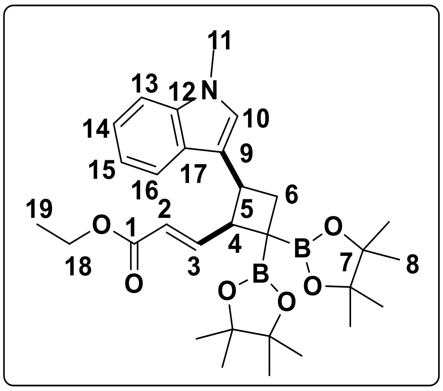

Prepared according to General Procedure **G**, **1** (75.6 mg, 0.2 mmol) and **S3** (157 mg, 1 mmol), in the presence of [Ru(dmbpy)$_3$](PF$_6$)$_2$ (0.9 mg, 0.5 mol%), were converted to **14** (NMR yield 72%, d.r.=2.3:1), yielding a light yellow solid (73 mg, 68%, d.r.=2.3:1), after purification by flash column chromatography (SiO_2, 15% EtOAc/*n*-hexane).

The major *syn*-diastereomer was isolated by a second column chromatography (SiO_2, 0%→10% EtOAc/*n*-hexane) as a yellow solid (40 mg, 37%).

R$_f$ (15% EtOAc/*n*-hexane) = 0.30; **^{1}H NMR** (400 MHz, $CDCl_3$) δ = 7.49 (d, *J* = 7.9 Hz, 1H, H16), 7.22 (d, *J* = 8.2 Hz, 1H, H13), 7.18 – 7.08 (m, 2H, H3+H14), 7.03 (ddd, *J* = 8.0, 6.8, 1.1 Hz, 1H, H15), 6.87 (d, *J* = 1.1 Hz, 1H, H10), 5.74 (d, *J* = 15.4 Hz, 1H, H2), 4.17 – 4.08 (m, 1H, H5), 4.06 – 3.94 (m, 2H, H18), 3.71 (m, 4H, H4+H11), 2.93 (t, *J* = 10.8 Hz, 1H, H6), 2.59 (ddd, *J* = 10.7, 8.4, 2.6 Hz, 1H, H6), 1.32 (s, 12H, H8), 1.22 (s, 6H, H8), 1.18 (s, 6H, H8), 1.13 (t, *J* = 7.1 Hz, 3H, H19) ppm; **^{13}C NMR** (100 MHz, $CDCl_3$) δ = 166.4 (C1), 150.0 (C2), 137.1 (C17), 127.5 (C12), 126.8 (C10), 121.7 (C1), 121.5 (C14), 119.4 (C16), 118.6 (C15), 115.7 (C9), 109.1 (C13), 83.7 (C7), 83.6 (C7), 59.7 (C18), 49.1 (C4), 36.0 (C5), 32.8 (C11), 30.2 (C6), 25.0 (C8), 24.9 (C8), 24.8 (C8), 24.6 (C8), 14.4 (C19) ppm; **^{11}B NMR** (128 MHz, $CDCl_3$) δ = 34.49 ppm; **IR** (ATR): ṽ = 669, 737, 849, 967, 981, 1039, 1138, 1165, 1214, 1270, 1304, 1326, 1371, 1470, 1646, 1715, 2976 cm^{-1}; **HRMS** (ESI) [M+Na]$^+$ Calculated mass for $C_{30}H_{43}B_2NO_6Na$:558.3169; Mass found:558.3202.

(*E*)-(2-(3-Ethoxy-3-oxoprop-1-en-1-yl)-3-vinylcyclobutane-1, 1-diyl)diboronic acid, pinacol ester (15)

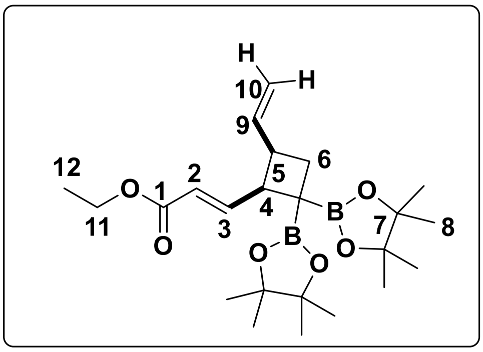

Prepared according to General Procedure **G**, **4** (75.6 mg, 0.2 mmol) and 1, 3-butadiene (0.5 mL, 15 wt% in hexane, 1 mmol), in the presence of [Ru(dmbpy)$_3$](PF$_6$)$_2$ (0.9 mg, 0.5 mol%), were converted to **15** (NMR yield 84%, d.r.=2:1), yielding a colorless oil (66 mg, 76%, d.r.=2:1), after purification by flash column chromatography (SiO_2, 10% EtOAc/ *n*-hexane).

The major *syn*-diastereomer was isolated by a second column chromatography (SiO_2, 0%→10% EtOAc/ *n*-hexane) as a colorless oil (33 mg, 38%).

R$_f$ (10% EtOAc/*n*-hexane) = 0.40; **^{1}H NMR** (400 MHz, $CDCl_3$) δ = 7.14 (dd, *J* = 15.4, 11.2 Hz, 1H, H3), 5.82 (d, *J* = 15.5 Hz, 1H, H2), 5.78 – 5.68 (m, 1H, H9), 5.03 – 4.90 (m, 2H, H10), 4.15 (q, *J* = 7.1 Hz, 2H, H11), 3.43 (ddd, *J* = 11.1, 8.3, 2.4 Hz, 1H, H4), 3.33 – 3.23 (m, 1H, H5), 2.57 (t, *J* = 10.4 Hz, 1H, H6), 2.28 (ddd, *J* = 10.7, 8.3, 2.4 Hz, 1H, H6), 1.27 – 1.18 (m, 27H, H8+H12) ppm; **^{13}C NMR** (100 MHz, $CDCl_3$) δ = 166.5 (C1), 149.8 (C3), 139.0 (C9), 121.8 (C2), 115.1 (C10), 83.7 (C7), 83.6 (C7), 60.0 (C11), 47.8 (C4), 42.5 (C5), 29.0 (C6), 24.91 (C8), 24.87 (C8), 24.8 (C8), 24.6 (C8), 14.5 (C12) ppm; **^{11}B NMR** (128 MHz, $CDCl_3$) δ = 34.49 ppm; **IR** (ATR): ṽ = 669, 697, 849, 911, 980, 1042, 1138, 1165, 1214, 1306, 1339, 1371, 1508, 1648, 1719, 2375, 2930, 2977 cm^{-1}; **HRMS** (ESI) [M+Na]$^+$ Calculated mass for $C_{23}H_{38}B_2O_6Na$: 455.2746; Mass found: 455.2812.

(*E*)-(2-(3-Ethoxy-3-oxoprop-1-en-1-yl)-3-(4-(4-((1-isopropoxy-2-methyl-1-oxopropan-2-yl)oxy)benzoyl)phenyl)cyclobutane-1, 1-diyl)diboronic acid, pinacol ester (16)

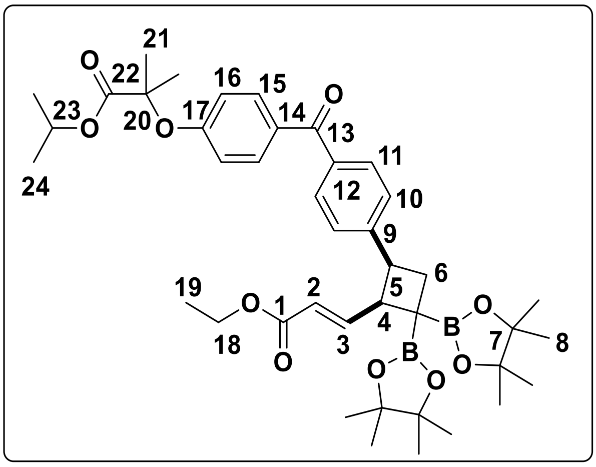

Prepared according to General Procedure **G**, **4** (75.6 mg, 0.2 mmol) and **S5** (352 mg, 1 mmol), in the presence of [Ru(dmbpy)$_3$](PF$_6$)$_2$ (0.9 mg, 0.5 mol%), were converted to **16** (NMR yield 88%, d.r.=2:1), yielding a white solid (120 mg, 82%, d.r.=2:1), after purification by flash column chromatography (SiO$_2$, 20% EtOAc/*n*-hexane).

The major *syn*-diastereomer was isolated by a second column chromatography (SiO$_2$, 0%→10% EtOAc/*n*-hexane) as a white solid (64 mg, 44%).

R$_f$ (15% EtOAc/*n*-hexane) = 0.15; **^{1}H NMR** (400 MHz, CDCl$_3$) δ = 7.75 – 7.69 (m, 2H, H15), 7.64 (d, *J* = 8.2 Hz, 2H, H11), 7.15 (d, *J* = 7.9 Hz, 2H, H10), 6.92 – 6.81 (m, 3H, H16+H3), 5.82 (d, *J* = 15.4 Hz, 1H, H2), 5.08 (p, *J* = 6.3 Hz, 1H, H23), 4.11 – 3.96 (m, 3H, H5+H18), 3.74 (ddd, *J* = 11.2, 8.3, 2.6 Hz, 1H, H4), 2.97 (t, *J* = 10.9 Hz, 1H, H6), 2.54 (ddd, *J* = 10.8, 8.4, 2.7 Hz, 1H, H6), 1.65 (s, 6H, H21), 1.30 (s, 6H, H8), 1.30 (s, 6H, H8), 1.22 – 1.15 (m, 21H, H8+H24+H19) ppm; **^{13}C NMR** (100 MHz, CDCl$_3$) δ = 195.5 (C13), 173.4 (C22), 166.1 (C1), 159.5 (C14), 148.8 (C3), 146.3 (C12), 135.8 (C9), 132.1 (C15), 131.1 (C17), 129.9 (C11), 127.0 (C10), 122.7 (C2), 117.3 (C16), 83.9 (C7), 83.7 (C7), 79.5 (C20), 69.4 (C23), 60.0 (C18), 48.9 (C4), 43.0 (C5), 28.7 (C6), 25.6 (C21), 25.5 (C21), 25.0 (C8), 24.9 (C8), 24.8 (C8), 24.7 (C8), 21.7 (C24), 14.4 (C19) ppm; **^{11}B NMR** (128 MHz, CDCl$_3$) δ = 34.49 ppm; **IR** (ATR): ṽ = 637, 669, 689, 768, 849, 928, 970, 1040, 1101, 1139, 1251, 1274, 1306, 1339, 1371, 1508, 1599, 1649, 1655, 1719, 2978 cm^{-1}; **HRMS** (ESI) [M+Na]$^+$ Calculated mass for $C_{41}H_{56}B_2O_{10}Na$:753.3952; Mass found:753.4041.

(*E*)-(2-(3-(*tert*-Butoxy)-3-oxoprop-1-en-1-yl)-3-(4-methoxyphenyl)cyclobutane-1,1-diyl)diboronic acid, pinacol ester (17)

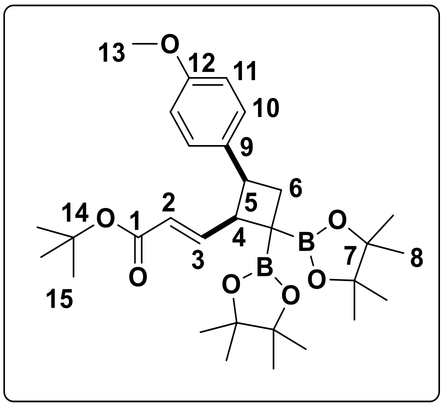

Prepared according to General Procedure **G**, **S18** (81.2 mg, 0.2 mmol) and 4-methoxystyrene (135 μL, 1 mmol), in the presence of [Ru(dmbpy)$_3$](PF$_6$)$_2$ (0.9 mg, 0.5 mol%), were converted to **17** (NMR yield 88%, d.r.=2.3:1), yielding a white solid (89 mg, 82%, d.r.=2.3:1), after purification by flash column chromatography (SiO$_2$, 10% EtOAc/*n*-hexane).

The major *syn*-diastereomer was isolated by a second column chromatography (SiO$_2$, 0%→10% EtOAc/*n*-hexane) as a white solid (49 mg, 45%).

R$_f$ (10% EtOAc/*n*-hexane) = 0.30; **^{1}H NMR** (400 MHz, CDCl$_3$) δ = 6.99 (d, *J* = 8.2 Hz, 2H, H10), 6.87 – 6.72 (m, 3H, H11+H3), 5.69 (d, *J* = 15.4 Hz, 1H, H2), 3.86 (dt, *J* = 11.2, 8.4 Hz, 1H, H4), 3.75 (s, 3H, H13), 3.62 (ddd, *J* = 11.2, 8.1, 2.7 Hz, 1H, H5), 2.90 (t, *J* = 10.9 Hz, 1H, H6), 2.45 (ddd, *J* = 10.8, 8.3, 2.7 Hz, 1H, H6), 1.36 (s, 9H, H15), 1.29 (m, 12H, H8), 1.21 (s, 6H, H8), 1.18 (s, 6H, H8) ppm; **^{13}C NMR** (100 MHz, CDCl$_3$) δ = 165.6 (C1), 157.8 (C12), 148.3 (C3), 133.8 (C9), 128.3 (C10), 123.8 (C2), 113.5 (C11), 83.7 (C7), 83.6 (C7), 79.5 (C14), 55.3 (C13), 49.0 (C5), 42.6 (C4), 29.0 (C6), 28.2 (C15), 24.91 (C8), 24.89 (C8), 24.8 (C8), 24.6 (C8) ppm; **^{11}B NMR** (128 MHz, CDCl$_3$) δ = 34.30 ppm; **IR** (ATR):

ῦ = 604, 637, 673, 712, 749, 823, 837, 856, 920, 968, 992, 1044, 1065, 1103, 1138, 1166, 1215, 1229, 1278, 1302, 1351, 1374, 1416, 1701, 2867, 2929, 2979, 2999 cm^{-1}; **HRMS** (ESI) [M+Na]$^+$ Calculated mass for $C_{30}H_{46}B_2O_7Na$:563.3322; Mass found:563.3388.

(*E*)-(2-(3-Oxo-3-(pent-2-yn-1-yloxy)prop-1-en-1-yl)-3-phenylcyclobutane-1, 1-diyl)diboronic acid, pinacol ester (18)

Prepared according to General Procedure **G**, **S19** (83.2 mg, 0.2 mmol) and styrene (115 μL, 1 mmol), in the presence of $[Ru(dmbpy)_3](PF_6)_2$ (0.9 mg, 0.5 mol%), were converted to **18** (NMR yield 72%, d.r.=2.6:1), yielding a white solid (68 mg, 65%, d.r.=2.6:1), after purification by flash column chromatography (SiO_2, 10% EtOAc/*n*-hexane).

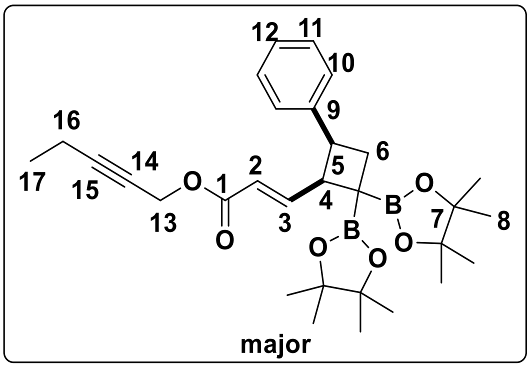

The major *syn*-diastereomer was isolated by a second column chromatography (SiO_2, 0%→10% EtOAc/*n*-hexane) as a white solid (40 mg, 38%).

R_f (10% EtOAc/*n*-hexane) = 0.30; **^{1}H NMR** (400 MHz, $CDCl_3$) δ = 7.22 (t, *J* = 7.5 Hz, 2H, H11), 7.11 (t, *J* = 7.7 Hz, 1H, H12), 7.05 (d, *J* = 7.2 Hz, 1H, H10), 6.95 (dd, *J* = 15.4, 11.5 Hz, 1H, H3), 5.82 (d, *J* = 15.4 Hz, 1H, H2), 4.66 – 4.51 (m, 2H, H13), 3.95 (dt, *J* = 11.4, 8.4 Hz, 1H, H5), 3.70 (ddd, *J* = 11.2, 8.3, 2.7 Hz, 1H, H4), 2.95 (t, *J* = 10.9 Hz, 1H, H6), 2.50 (ddd, *J* = 10.8, 8.4, 2.7 Hz, 1H, H6), 2.18 (qt, *J* = 7.5, 2.2 Hz, 2H, H16), 1.30 (s, 6H, H8), 1.29 (s, 6H, H8), 1.21 (s, 6H, H8), 1.18 (s, 6H, H8), 1.10 (t, *J* = 7.5 Hz, 3H, H17) ppm; **^{13}C NMR** (100 MHz, $CDCl_3$) δ = 165.5 (C1), 150.7 (C3), 141.4 (C9), 128.1 (C11), 127.1 (C10), 126.0 (C12), 121.5 (C2), 88.4 (C15), 83.8 (C7), 83.7 (C7), 73.7 (C14), 52.3 (C13), 49.0 (C4), 43.0 (C5), 28.8 (C6), 24.92 (C8), 24.89 (C8), 24.8 (C8), 24.6 (C8), 13.7 (C17), 12.5 (C16) ppm; **^{11}B NMR** (192 MHz, $CDCl_3$) δ = 33.53 ppm; **IR** (ATR): ῦ = 670, 703, 739, 753, 825, 847, 891, 986, 1039, 1056, 1125, 1161, 1175, 1214, 1282, 1307, 1371, 1445, 1650, 1724, 2977 cm^{-1}; **HRMS** (ESI) [M+Na]$^+$ Calculated mass for $C_{30}H_{42}B_2O_6Na$: 543.3060; Mass found: 543.3012.

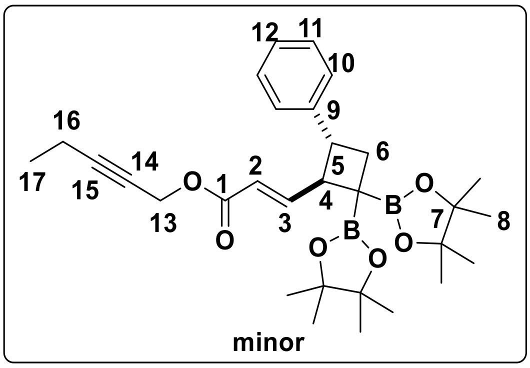

R_f (10% EtOAc/*n*-hexane) = 0.32; **^{1}H NMR** (400 MHz, $CDCl_3$) δ = 7.36 – 7.25 (m, 3H, H3+H11), 7.19 (dt, *J* = 9.7, 2.9 Hz, 3H, H10+H12), 5.85 (dd, *J* = 15.6, 1.3 Hz, 1H, H2), 4.81 – 4.65 (m, 2H, H13), 3.62 (q, *J* = 9.3 Hz, 1H, H5), 3.43 (t, *J* = 8.5 Hz, 1H, H4), 2.64 (t, *J* = 9.4 Hz, 1H, H6), 2.31 – 2.14 (m, 3H, H6+H16), 1.29 (s, 12H, H8), 1.26 (s, 6H, H8), 1.24 (s, 6H, H8), 1.16 (t, *J* = 7.5 Hz, 3H, H17) ppm; **^{13}C NMR** (100 MHz, $CDCl_3$) δ = 166.2 (C1), 152.9 (C3), 144.3 (C9), 128.4 (C11), 126.5 (C10), 126.2 (C12), 119.2 (C2), 88.7 (C15), 83.7 (C7), 83.4 (C7), 73.7 (C14), 52.6 (C13), 48.6 (C4), 44.6 (C5), 30.3 (C6), 25.2 (C8), 25.0 (C8), 24.9 (C8), 24.6 (C8), 13.7 (C17), 12.6 (C16) ppm; **^{11}B NMR** (128 MHz, $CDCl_3$) δ = 35.15 ppm; **IR** (ATR): ῦ = 670, 703, 739, 753, 825, 847, 891, 967, 986, 1039, 1058, 1078, 1125, 1161, 1175, 1214, 1282, 1307, 1336, 1371, 1445, 1650, 1724, 2927, 2977 cm^{-1}; **HRMS** (ESI) [M+Na]$^+$ Calculated mass for $C_{30}H_{42}B_2O_6Na$: 543.3060; Mass found: 543.3104.

(*E*)-(2-(3-(But-3-en-1-yloxy)-3-oxoprop-1-en-1-yl)-3-phenylcyclobutane-1, 1-diyl)diboronic acid, pinacol ester (19)

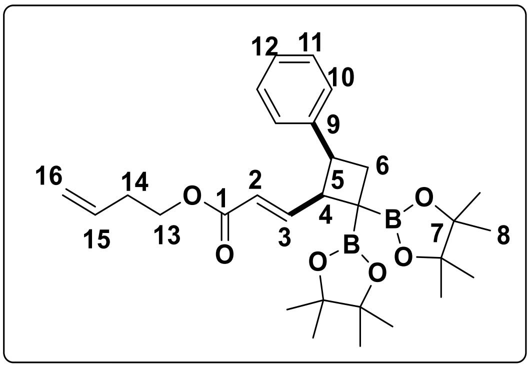

Prepared according to General Procedure **G**, **S20** (80.8 mg, 0.2 mmol) and styrene (115 μL, 1 mmol), in the presence of $[Ru(dmbpy)_3](PF_6)_2$ (0.9 mg, 0.5 mol%), were converted to **19** (NMR yield 89%, d.r.=2.5:1), yielding a colorless oil (84 mg, 83%, d.r.=2.5:1), after purification by flash column chromatography (SiO_2, 10% EtOAc/*n*-hexane).

The major *syn*-diastereomer was isolated by a second column chromatography (SiO_2, 0%→10% EtOAc/*n*-hexane) as a colorless oil (50 mg, 49%).

R$_f$ (10% EtOAc/*n*-hexane) = 0.30; **^{1}H NMR** (400 MHz, $CDCl_3$) δ = 7.22 (t, *J* = 7.5 Hz, 2H, H11), 7.10 (t, *J* = 7.4 Hz, 1H, H12), 7.05 (d, *J* = 7.6 Hz, 2H, H10), 6.90 (dd, *J* = 15.4, 11.4 Hz, 1H, H3), 5.78 (d, *J* = 15.4 Hz, 1H, H2), 5.69 (ddt, *J* = 17.0, 10.2, 6.7 Hz, 1H, H15), 5.05 – 4.97 (m, 2H, H16), 4.09 (dt, *J* = 10.8, 6.9 Hz, 1H, H13), 4.02 – 3.89 (m, 2H, H5+H13), 3.69 (ddd, *J* = 11.2, 8.3, 2.6 Hz, 1H, H4), 2.95 (t, *J* = 10.9 Hz, 1H, H6), 2.49 (ddd, *J* = 10.8, 8.5, 2.7 Hz, 1H, H6), 2.33 – 2.24 (m, 2H, H14), 1.29 (s, 6H, H8), 1.29 (s, 6H, H8), 1.20 (s, 6H, H8), 1.17 (s, 6H, H8) ppm; **^{13}C NMR** (100 MHz, $CDCl_3$) δ = 166.1 (C1), 149.8 (C3), 141.4 (C9), 134.2 (C15), 128.0 (C11), 127.2 (C10), 125.9 (C12), 122.0 (C2), 117.1 (C16), 83.7 (C8), 83.6 (C8), 63.0 (C13), 48.9 (C4), 43.1 (C5), 33.2 (C14), 28.7 (C6), 24.9 (C8), 24.84 (C8), 24.75 (C8), 24.6 (C8) ppm; **^{11}B NMR** (128 MHz, $CDCl_3$) δ = 34.56 ppm; **IR** (ATR): ṽ = 673, 697, 719, 739, 765, 816, 839, 855, 964, 984, 1019, 1052, 1072, 1096, 1138, 1162, 1187, 1211, 1226, 1240, 1269, 1302, 1336, 1371, 1389, 1411, 1424, 1452, 1507, 1602, 1731, 2934, 2977 cm^{-1}; **HRMS** (ESI) $[M+Na]^+$ Calculated mass for $C_{29}H_{42}B_2O_6Na$: 531.3060; Mass found: 531.3074.

(*E*)-(3-([1, 1'-Biphenyl]-4-yl)-2-(3-((1-(*tert*-butoxycarbonyl)piperidin-4-yl)oxy)-3-oxoprop-1-en-1-yl)cyclobutane-1, 1-diyl)diboronic acid, pinacol ester (20)

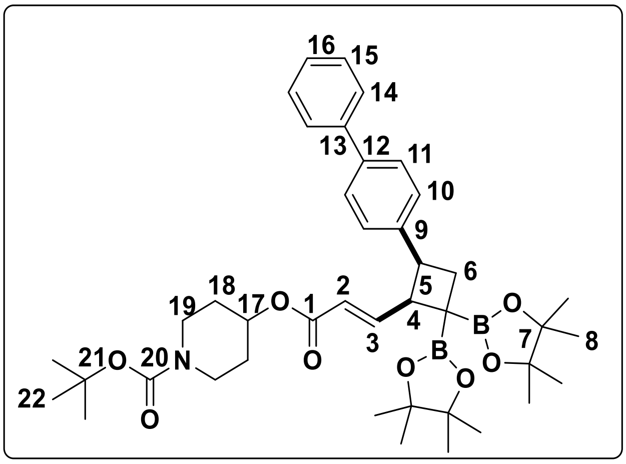

Prepared according to General Procedure **G**, **S21** (106.7 mg, 0.2 mmol) and 4-vinylbiphenyl (180 mg, 1 mmol), in the presence of $[Ru(dmbpy)_3](PF_6)_2$ (0.9 mg, 0.5 mol%), were converted to **20** (NMR yield 94%, d.r.=2:1), yielding a white solid (126 mg, 88%, d.r.=2:1), after purification by flash column chromatography (SiO_2, 30% EtOAc/*n*-hexane).

The major *syn*-diastereomer was isolated by a second column chromatography (SiO_2, 0%→30% EtOAc/*n*-hexane) as a white solid (72 mg, 50%).

R$_f$ (25% EtOAc/*n*-hexane) = 0.25; **^{1}H NMR** (400 MHz, $CDCl_3$) δ = 7.57 – 7.53 (m, 2H, H14), 7.47 (d, *J* = 8.3 Hz, 2H, H11), 7.41 (t, *J* = 7.6 Hz, 2H, H15), 7.33 – 7.28 (m, 1H, H16), 7.14 (d, *J* = 8.1 Hz, 2H, H10), 6.96 (dd, *J* = 15.4, 11.5 Hz, 1H, H3), 5.81 (d, *J* = 15.4 Hz, 1H, H2), 4.84 (tt, *J* = 7.8, 3.7 Hz, 1H, H17), 3.99 (dt, *J* = 11.2, 8.4 Hz, 1H, H5), 3.72 (ddd, *J* = 11.2, 8.2, 2.6 Hz, 1H, H4), 3.63 (ddd, *J* = 13.3, 6.8, 3.9 Hz, 1H, H19), 3.54 (dd, *J* = 13.0, 6.4 Hz, 1H, H19), 3.16 (tdd, *J* = 13.7, 8.4, 3.6 Hz, 2H, H19), 2.99 (t, *J* = 10.9 Hz, 1H), H6, 2.53 (ddd, *J* = 10.8, 8.4, 2.7

Hz, 1H, H6), 1.73 (tdd, J = 15.5, 6.6, 3.1 Hz, 2H, H18), 1.55 – 1.46 (m, 2H, H18), 1.44 (s, 9H, H22), 1.31 (s, 6H, H8), 1.30 (s, 6H, H8), 1.21 (s, 6H, H8), 1.18 (s, 6H, H8) ppm; **^{13}C NMR** (100 MHz, $CDCl_3$) δ = 165.4 (C1), 154.8 (C20), 150.1 (C3), 141.2 (C9), 140.6 (C13), 138.8 (C12), 128.8 (C15), 127.7 (C10), 127.13 (C16), 127.08 (C14), 126.8 (C11), 122.2 (C2), 83.8 (C7), 83.7 (C7), 79.7 (C21), 69.1 (C17), 49.0 (C4), 43.0 (C5), 41.0 (C19), 30.7 (C18), 30.7 (C18), 28.8 (C6), 28.6 (C22), 24.94 (C8), 24.91 (C8), 24.8 (C8), 24.7 (C8) ppm; **^{11}B NMR** (128 MHz, $CDCl_3$) δ = 34.68 ppm; **IR** (ATR): ṽ = 670, 697, 729, 765, 851, 911, 983, 1007, 1025, 1138, 1164, 1336, 1365, 1474, 1691, 2977 cm^{-1}; **HRMS** (ESI) $[M+Na]^+$ Calculated mass for $C_{41}H_{57}B_2NO_8Na$: 736.4162; Mass found: 736.4197.

(*E*)-(2-(3-Oxo-3-(phenethylthio)prop-1-en-1-yl)-3-phenylcyclobutane-1, 1-diyl)diboronic acid, pinacol ester (21)

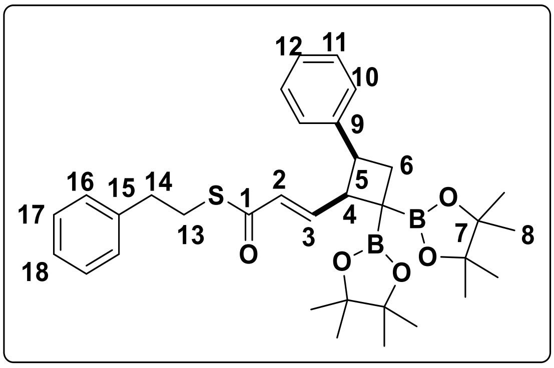

Prepared according to General Procedure **G**, **S22** (94.1 mg, 0.2 mmol) and styrene (115 µL, 1 mmol), in the presence of $[Ru(dmbpy)_3](PF_6)_2$ (0.9 mg, 0.5 mol%), were converted to **21** (NMR yield 52%, d.r.=2.7:1), yielding a light yellow solid (51 mg, 44%, d.r.=2.7:1), after purification by flash column chromatography (SiO_2, 10% EtOAc/ *n*-hexane).

The major *syn*-diastereomer was isolated by a second column chromatography (SiO_2, 0%→10% EtOAc/ *n*-hexane) as a yellow solid (28 mg, 24%).

R_f (10% EtOAc/*n*-hexane) = 0.30; **^{1}H NMR** (600 MHz, $CDCl_3$) δ = 7.31 – 7.25 (m, 4H, H11+H16), 7.22 (t, J = 7.4 Hz, 1H, H18), 7.19 (d, J = 7.8 Hz, 2H, H17), 7.15 (t, J = 7.4 Hz, 1H, H12), 7.09 (d, J = 7.6 Hz, 2H, H10), 6.89 (ddd, J = 15.3, 11.3, 0.9 Hz, 1H, H3), 6.11 (d, J = 15.3 Hz, 1H, H2), 3.99 (dt, J = 11.4, 8.4 Hz, 1H, H5), 3.69 (ddd, J = 11.1, 8.1, 2.6 Hz, 1H, H4), 3.14 – 3.03 (m, 2H, H13), 2.98 (t, J = 10.9 Hz, 1H, H6), 2.80 (ddd, J = 9.5, 7.4, 2.8 Hz, 2H, H14), 2.54 (ddd, J = 10.7, 8.3, 2.7 Hz, 1H, H6), 1.33 (s, 6H, H8), 1.32 (s, 6H, H8), 1.22 (s, 6H, H8), 1.19 (s, 6H, H8) ppm; **^{13}C NMR** (151 MHz, $CDCl_3$) δ = 189.2 (C1), 146.2 (C3), 141.3 (C9), 140.3 (C15), 129.4 (C2), 128.7 (C16), 128.5 (C17), 128.2 (C11), 127.2 (C10), 126.5 (C18), 126.0 (C12), 83.8 (C7), 83.7 (C7), 48.9 (C4), 43.2 (C5), 36.1 (C14), 30.0 (C13), 28.8 (C6), 25.0 (C8), 24.9 (C8), 24.8 (C8), 24.6 (C8) ppm; **^{11}B NMR** (192 MHz, $CDCl_3$) δ = 30.73, 34.30 ppm; **IR** (ATR): ṽ = 660, 699, 742, 779, 793, 847, 959, 1045, 1121, 1172, 1205, 1284, 1340, 1369, 1388, 1508, 1625, 1648, 1671, 2375, 2930, 2977 cm^{-1}; **HRMS** (ESI) $[M+Na]^+$ Calculated mass for $C_{33}H_{44}B_2O_5SNa$: 597.2988; Mass found: 597.3030.

(*E*)-(2-(3-Morpholino-3-oxoprop-1-en-1-yl)-3-phenylcyclobutane-1, 1-diyl)diboronic acid, pinacol ester (22)

Prepared according to General Procedure **G**, **S23** (83.8 mg, 0.2 mmol) and styrene (115 μL, 1 mmol), in the presence of $[Ru(dmbpy)_3](PF_6)_2$ (0.9 mg, 0.5 mol%), were converted to **22** (NMR yield 100%, d.r.=2.2:1), yielding a white solid (94 mg, 90%, d.r.=2.2:1), after purification by flash column chromatography (SiO_2, 50% EtOAc/ *n*-hexane).

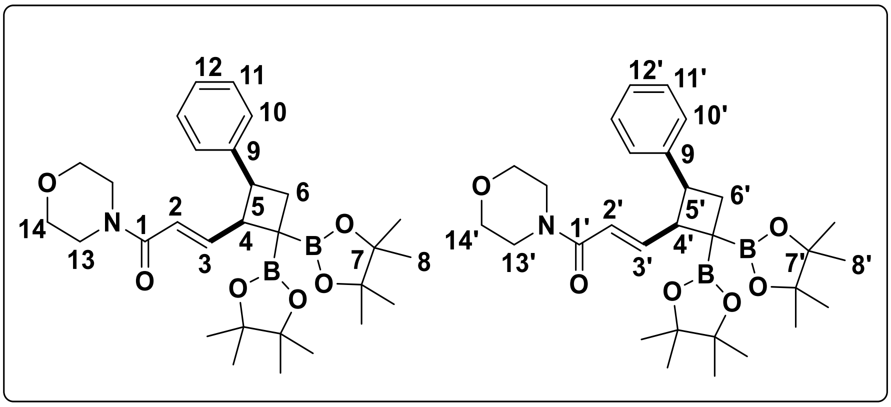

***R**f* (50% EtOAc/*n*-hexane) = 0.30; **^{1}H NMR** (400 MHz, $CDCl_3$) δ = 7.32 – 7.05 (m, 7.4H, Ar+H3'), 6.73 (dd, *J* = 15.1, 11.0 Hz, 1H, H3), 6.19 (dd, *J* = 15.0, 1.2 Hz, 0.4H, H2'), 6.05 (d, *J* = 15.0 Hz, 1H, H2), 3.96 (dt, *J* = 11.3, 8.3 Hz, 1H, H5), 3.77 – 3.13 (m, 13H, H13+ H13' + H14+ H14'+ H5'+ H4+ H4'), 2.98 (t, *J* = 10.9 Hz, 1H, H6), 2.67 – 2.60 (m, 0.4H, H6'), 2.49 (ddd, *J* = 10.7, 8.3, 2.8 Hz, 1H, H6), 2.22 (t, *J* = 10.0 Hz, 0.4H, H6'), 1.34 – 1.18 (m, 33.6H, H8+H8') ppm; **^{13}C NMR** (100 MHz, $CDCl_3$) (only major isomer assigned) δ = 165.5 (C1), 146.1 (C3), 141.6 (C9), 128.1 (C11), 127.3 (C10), 125.8 (C12), 121.1 (C2), 83.7 (C7), 83.6 (C7), 66.9 (C14), 49.4 (C4), 43.6 (C5), 28.6 (C6), 24.89 (C8), 24.86 (C8) ppm; **^{11}B NMR** (128 MHz, $CDCl_3$) δ = 33.76 ppm; **IR** (ATR): ῡ = 671, 699, 733, 753, 849, 924, 981, 1009, 1042, 1069, 1114, 1138, 1165, 1214, 1233, 1269, 1338, 1371, 1508, 1603, 1649, 1714, 2375, 2977 cm^{-1}; **HRMS** (ESI) $[M+Na]^+$ Calculated mass for $C_{29}H_{43}B_2NO_6Na$: 546.3169; Mass found: 546.3106.

(*E*)-(2-(3-(Methoxy(methyl)amino)-3-oxoprop-1-en-1-yl)-3-phenylcyclobutane-1, 1-diyl)diboronic acid, pinacol ester (23)

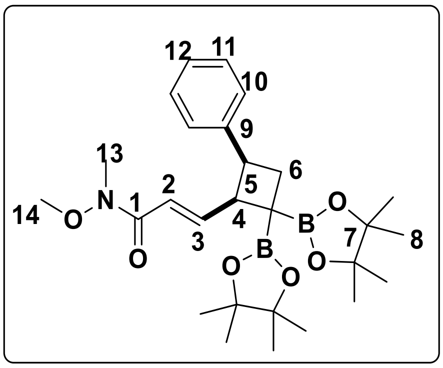

Prepared according to General Procedure **G**, **S24** (78.6 mg, 0.2 mmol) and styrene (115 μL, 1 mmol), in the presence of $[Ru(dmbpy)_3](PF_6)_2$ (0.9 mg, 0.5 mol%), were converted to **23** (NMR yield 92%, d.r.=2.1:1), yielding a white solid (81 mg, 81%, d.r.=2.1:1), after purification by flash column chromatography (SiO_2, 50% EtOAc/*n*-hexane).

The major *syn*-diastereomer was isolated by a second column chromatography (SiO_2, 0%→10% EtOAc/*n*-hexane) as a white solid (44 mg, 44%).

***R**f* (50% EtOAc/*n*-hexane) = 0.25; **^{1}H NMR** (400 MHz, $CDCl_3$) δ = 7.20 (dd, *J* = 8.6, 6.7 Hz, 2H, H11), 7.10 – 7.04 (m, 3H, H10+H12), 6.91 (dd, *J* = 15.2, 11.1 Hz, 1H, H3), 6.31 (d, *J* = 15.2 Hz, 1H, H2), 3.94 (dt, *J* = 11.4, 8.3 Hz, 1H, H5), 3.74 (ddd, *J* = 11.2, 8.2, 2.8 Hz, 1H, H4), 3.51 (s, 3H, H14), 3.10 (s, 3H, H13), 2.98 (t, *J* = 10.9 Hz, 1H, H6), 2.48 (ddd, *J* = 10.8, 8.3, 2.9 Hz, 1H, H6), 1.30 (s, 6H, H8), 1.29 (s, 6H, H8), 1.22 (s, 6H, H8), 1.18 (s, 6H, H8) ppm; **^{13}C NMR** (100 MHz, $CDCl_3$) δ = 166.7 (C1), 147.6 (C3), 141.6 (C9), 128.0 (C11), 127.3 (C10), 125.8 (C12), 120.2 (C2), 83.7 (C7), 83.6 (C7), 61.5 (C14), 49.3 (C4), 43.6 (C5), 32.4 (C13), 28.9 (C6), 25.0 (C8), 24.92 (C8), 24.86 (C8), 24.6 (C8) ppm; **^{11}B NMR** (128 MHz, $CDCl_3$) δ = 35.24 ppm; **IR** (ATR): ῡ = 851, 984, 1137, 1231, 1261, 1332, 1582, 1619, 1648, 2976 cm^{-1}; **HRMS** (ESI) $[M+Na]^+$ Calculated mass for $C_{27}H_{41}B_2NO_6Na$: 520.3012; Mass found: 520.3024.

(*E*)-(2-(3-(9*H*-Carbazol-9-yl)-3-oxoprop-1-en-1-yl)-3-phenylcyclobutane-1, 1-diyl)diboronic acid, pinacol ester (24)

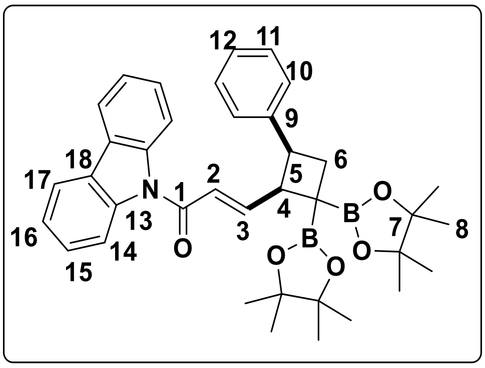

Prepared according to General Procedure **G**, **S25** (75.6 mg, 0.2 mmol) and styrene (115 µL, 1 mmol), in the presence of [Ru(dmbpy)$_3$](PF$_6$)$_2$ (0.9 mg, 0.5 mol%), were converted to **24** (NMR yield 90%, d.r.=2:1), yielding a light yellow solid (104 mg, 86%, d.r.=2:1), after purification by flash column chromatography (SiO$_2$, 15% EtOAc/ *n*-hexane).

The major *syn*-diastereomer was isolated by a second column chromatography (SiO$_2$, 0%→15% EtOAc/ *n*-hexane) as a yellow solid (58 mg, 48%).

R$_f$ (10% EtOAc/*n*-hexane) = 0.20; **^{1}H NMR** (400 MHz, CDCl$_3$) δ = 7.97 – 7.94 (m, 2H, H17), 7.89 (d, *J* = 8.2 Hz, 2H, H14), 7.39 (ddd, *J* = 8.3, 7.3, 1.4 Hz, 2H, H15), 7.35 – 7.26 (m, 5H, H11+H3+H16), 7.25 – 7.20 (m, 2H, H10), 7.16 – 7.10 (m, 1H, H12), 6.68 (d, *J* = 15.0 Hz, 1H, H2), 4.09 (dt, *J* = 11.2, 8.4 Hz, 1H, H5), 3.91 (ddd, *J* = 11.1, 8.2, 2.6 Hz, 1H, H4), 3.10 (t, *J* = 10.9 Hz, 1H, H6), 2.56 (ddd, *J* = 10.8, 8.4, 2.7 Hz, 1H, H6), 1.33 (s, 6H, H8), 1.32 (s, 6H, H8), 1.25 (s, 6H, H8), 1.21 (s, 6H, H8) ppm; **^{13}C NMR** (100 MHz, CDCl$_3$) δ = 165.6 (C1), 151.2 (C3), 141.4 (C9), 138.7 (C13), 128.3 (C11), 127.4 (C10), 126.9 (C15), 126.4 (C12), 125.9 (C18), 125.4 (C2), 123.1 (C16), 119.9 (C17), 115.7 (C14), 83.9 (C7), 83.8 (C7), 49.4 (C4), 43.7 (C5), 28.8 (C6), 24.97 (C8), 24.95 (C8), 24.9 (C8), 24.7 (C8) ppm; **^{11}B NMR** (128 MHz, CDCl$_3$) δ = 34.30 ppm; **IR** (ATR): ῦ = 634, 670, 699, 724, 753, 848, 908, 966, 981, 1096, 1137, 1214, 1284, 1444, 1478, 1630, 1678, 2977 cm^{-1}; **HRMS** (ESI) [M+Na]$^+$ Calculated mass for $C_{37}H_{43}B_2NO_5Na$: 626.3219; Mass found: 626.3229.

(*E*)-(2-(2-Boronovinyl)-3-(4-methoxyphenyl)cyclobutane-1, 1-diyl)diboronic acid, pinacol ester (25)

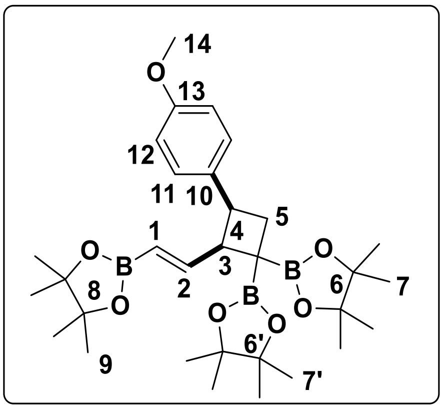

Prepared according to General Procedure **G**, **S26** (113.7 mg, 0.2 mmol) and 4-methoxystyrene (135 µL, 1 mmol), in the presence of [Ru(dmbpy)$_3$](PF$_6$)$_2$ (0.9 mg, 0.5 mol%), were converted to **25** (NMR yield 58%, d.r.=6:1), yielding a light yellow oil (53 mg, 47%, d.r.=6:1), after purification by flash column chromatography (SiO$_2$, 10% EtOAc/ *n*-hexane).

The major *syn*-diastereomer was isolated by a second column chromatography (SiO$_2$, 0%→10% EtOAc/ *n*-hexane) as a yellow oil (36 mg, 32%).

R$_f$ (10% EtOAc/*n*-hexane) = 0.20; **^{1}H NMR** (400 MHz, CDCl$_3$) δ =7.00 – 6.95 (m, 2H), 6.77 – 6.73 (m, 2H), 6.59 (dd, *J* = 17.6, 10.8 Hz, 1H, H2), 5.38 (d, *J* = 17.5 Hz, 1H, H1), 3.84 – 3.79 (m, 1H, H4), 3.75 (s, 3H, H14), 3.58 (ddd, *J* = 11.0, 8.2, 2.8 Hz, 1H, H3), 2.86 (t, *J* = 10.9 Hz, 1H, H5), 2.44 (ddd, *J* = 10.2, 8.4, 2.9 Hz, 1H, H5), 1.28 (s, 6H, H7/7'/9), 1.28 (s, 6H, H7/7'/9), 1.22 (s, 6H, H7/7'/9), 1.20 (s, 6H, H7/7'/9), 1.14 (s, 6H, H7/7'/9), 1.12 (s, 6H, H7/7'/9) ppm; **^{13}C NMR** (100 MHz, CDCl$_3$) δ = 157.5 (C13), 154.5 (C2), 134.5 (C10), 128.5 (C11), 113.2 (C12), 83.5 (C6/6'/8), 83.4 (C6/6'/8), 82.7 (C6/6'/8), 55.3 (C14), 53.0 (C3), 42.8 (C4), 29.5 (C5), 24.94 (C7/7'/9), 24.93(C7/7'/9), 24.90 (C7/7'/9), 24.67 (C7/7'/9), 24.66 (C7/7'/9) ppm; **^{11}B NMR** (128 MHz, CDCl$_3$) δ = 34.05 ppm; **IR** (ATR): ῦ = 669, 828, 849, 970, 1033, 1139, 1213, 1307, 1339, 1369, 1510, 2375, 2977 cm^{-1}; **HRMS** (ESI) [M+Na]$^+$ Calculated mass for $C_{31}H_{49}B_3O_7Na$: 589.3649; Mass found: 589.3605.

(*E*)-(2-(3-(4-(6-Fluorobenzo[*d*]isoxazol-3-yl)piperidin-1-yl)-3-oxoprop-1-en-1-yl)-3-(4-methoxyphenyl)cyclobutane-1, 1-diyl)diboronic acid, pinacol ester (26)

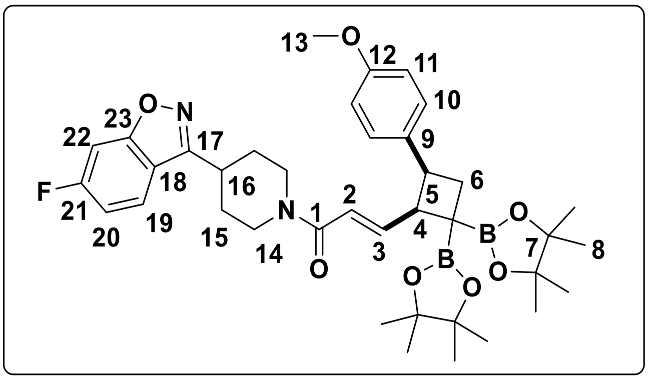

Prepared according to General Procedure **G**, **S27** (110.5 mg, 0.2 mmol) and 4-methoxystyrene (135 µL, 1 mmol), in the presence of $[Ru(dmbpy)_3](PF_6)_2$ (0.9 mg, 0.5 mol%), were converted to **26** (NMR yield 89%, d.r.=2.7:1), yielding a light yellow solid (108 mg, 79%, d.r.=2.7:1), after purification by flash column chromatography (SiO_2, EtOAc).

The major *syn*-diastereomer was isolated by a second column chromatography (SiO_2, 0%→10% EtOAc/*n*-hexane) as a white solid (53 mg, 39%).

***R**f* (EtOAc) = 0.33; **^{1}H NMR** (400 MHz, $CDCl_3$) = δ 7.57 (dd, *J* = 8.9, 5.1 Hz, 1H, H19), 7.27 – 7.22 (m, 1H, H22), 7.07 (td, *J* = 8.8, 2.1 Hz, 1H, H20), 7.01 (d, *J* = 8.5 Hz, 2H, H10), 6.85 – 6.71 (m, 3H, H3+H11), 6.14 (d, *J* = 15.0 Hz, 1H, H2), 4.56 (m, 1H, H14), 3.93 – 3.85 (m, 1H, H5), 3.80 – 3.59 (m, 5H, H13+H4+H14), 3.25 (tt, *J* = 11.4, 3.9 Hz, 1H, H16), 3.03 (m, 1H, H14), 2.93 (t, *J* = 10.9 Hz, 1H, H6), 2.80 (m, 1H, H14), 2.46 (ddd, *J* = 10.7, 8.3, 2.8 Hz, 1H, H6), 2.03 – 1.92 (m, 2H, H15), 1.89 – 1.75 (m, 2H, H15), 1.30 (s, 6H, H8), 1.29 (s, 6H, H8), 1.24 (s, 6H, H8), 1.21 (s, 6H, H8) ppm; **^{13}C NMR** (100 MHz, $CDCl_3$) δ = 164.3 (d, *J* = 250.7 Hz, C21), 164.0 (d, *J* = 13.5 Hz, C23), 160.4 (C17), 145.9 (C3), 157.77 (C12), 133.9 (C9), 128.4 (C10), 122.5 (d, *J* = 11.1 Hz, C19), 121.4 (C2), 117.2 (C18), 113.5 (C11), 112.7 (d, *J* = 25.4 Hz, C20), 97.7 (d, *J* = 26.9 Hz, C22), 83.7 (C7), 55.2 (C13), 49.6 (C4), 43.2 (C5), 34.7 (C16), 30.8 (C15), 29.0 (C6), 25.0 (C8), 24.6 (C8) ppm; **^{19}F NMR** (376 MHz, $CDCl_3$) δ = -109.28 (td, *J* = 8.7, 5.0 Hz) ppm; **^{11}B NMR** (128 MHz, $CDCl_3$) δ = 34.68 ppm; **IR** (ATR): ṽ = 608, 621, 646, 669, 729, 759, 826, 847, 921, 956, 970, 1006, 1035, 1122, 1138, 1167, 1215, 1246, 1270, 1303, 1340, 1371, 1445, 1513, 1610, 1649, 2977 cm^{-1}; **HRMS** (ESI) $[M+Na]^+$ Calculated mass for $C_{38}H_{49}B_2FN_2O_7Na$: 709.3602; Mass found: 709.3662.

(*E*)-(2-(3-((5-(2, 5-Dimethylphenoxy)-2, 2-dimethylpentyl)oxy)-3-oxoprop-1-en-1-yl)-3-phenylcyclobutane-1, 1-diyl)diboronic acid, pinacol ester (27)

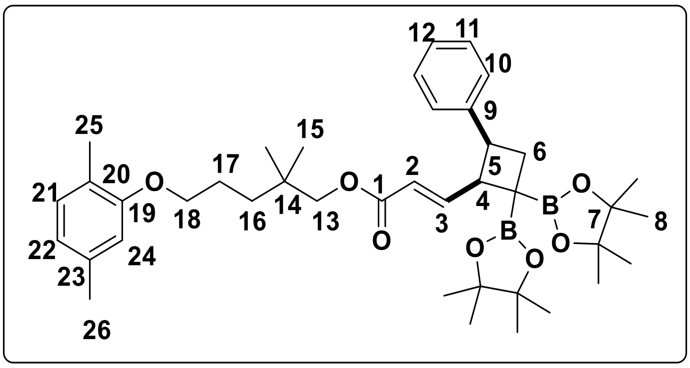

Prepared according to General Procedure **G**, **S28** (113.7 mg, 0.2 mmol) and styrene (115 μL, 1 mmol), in the presence of $[Ru(dmbpy)_3](PF_6)_2$ (0.9 mg, 0.5 mol%), were converted to **27** (NMR yield 77%, d.r.=2:1), yielding a white solid (99 mg, 74%, d.r.=2:1), after purification by flash column chromatography (SiO_2, 10% EtOAc/*n*-hexane).

The major *syn*-diastereomer was isolated by a second column chromatography (SiO_2, 0%→10% EtOAc/*n*-hexane) as a white solid (58 mg, 43%).

$\boldsymbol{R_f}$ (10% EtOAc/*n*-hexane) = 0.30; **^{1}H NMR** (400 MHz, $CDCl_3$) δ = 7.21 (dd, *J* = 8.7, 6.7 Hz, 2H, H11), 7.07 (m, 3H, H10+H12), 7.00 (d, *J* = 7.4 Hz, 1H, H21), 6.91 (dd, *J* = 15.4, 11.4 Hz, 1H, H3), 6.66 (d, *J* = 7.5 Hz, 1H, H22), , 6.61 (d, *J* = 1.6 Hz, 1H, H24), 5.81 (d, *J* = 15.4 Hz, 1H, H2), 4.00 – 3.92 (m, 1H, H5), 3.90 (d, *J* = 10.9 Hz, 1H, H13), 3.86 (t, *J* = 6.5 Hz, 2H, H18), 3.70 (ddd, *J* = 11.2, 8.2, 2.6 Hz, 1H, H4), 3.60 (d, *J* = 10.8 Hz, 1H, H13), 2.96 (t, *J* = 10.9 Hz, 1H, H6), 2.50 (ddd, *J* = 10.8, 8.4, 2.7 Hz, 1H, H6), 2.32 (s, 3H, H26), 2.17 (s, 3H, H25), 1.69 (m, 2H, H17), 1.36 – 1.31 (m, 2H, H16), 1.30 (s, 6H, H8), 1.30 (s, 6H, H8), 1.18 (s, 6H, H8), 1.15 (s, 6H, H8), 0.86 (s, 6H, H15) ppm; **^{13}C NMR** (100 MHz, $CDCl_3$) δ = 166.3 (C1), 157.2 (C19), 149.8 (C3), 141.4 (C9), 136.6 (C23), 130.4 (C21), 128.1 (C11), 127.3 (C10), 126.0 (C12), 123.7 (C20), 122.0 (C2), 120.8 (C22), 112.1 (C24), 83.8 (C7), 83.6 (C7), 71.8 (C13), 68.5 (C18), 48.9 (C4), 43.2 (C5), 35.5 (C16), 33.8 (C14), 28.6 (C6), 25.0 (C8), 24.9 (C8), 24.8 (C8), 24.6 (C8), 24.4 (C15), 24.2 (C15), 24.2 (C17), 21.6 (C26), 16.0 (C25) ppm; **^{11}B NMR** (128 MHz, $CDCl_3$) δ = 34.87 ppm; **IR** (ATR): ṽ = 669, 699, 737, 802, 849, 967, 1035, 1214, 1261, 1306, 1340, 1371, 1508, 1584, 1613, 1648, 1715, 2976 cm^{-1}; **HRMS** (ESI) $[M+Na]^+$ Calculated mass for $C_{40}H_{58}B_2O_7Na$: 695.4261; Mass found: 695.4272.

(*E*)-(2-(3-(4-(2-((2, 4-Dimethylphenyl)thio)phenyl)piperazin-1-yl)-3-oxoprop-1-en-1-yl)-3-phenylcyclobutane-1, 1-diyl)diboronic acid, pinacol ester (28)

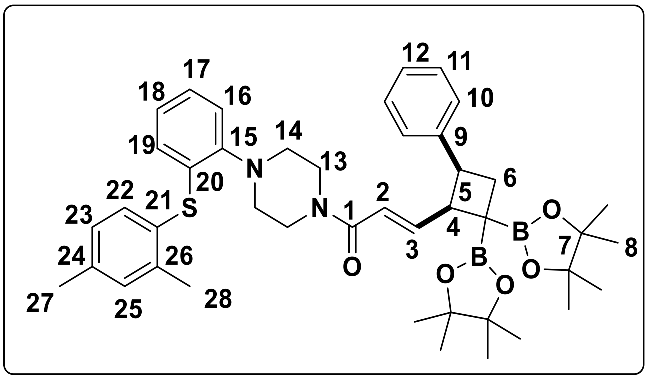

Prepared according to General Procedure **G**, **S29** (113.7 mg, 0.2 mmol) and styrene (115 μL, 1 mmol), in the presence of $[Ru(dmbpy)_3](PF_6)_2$ (0.9 mg, 0.5 mol%), were converted to **28** (NMR yield 76%, d.r.=2.8:1), yielding a light yellow solid (103 mg, 70%, d.r.=2.8:1), after purification by flash column chromatography (SiO_2, 10% EtOAc/*n*-hexane).

The major *syn*-diastereomer was isolated by a second column chromatography (SiO_2, 0%→10% EtOAc/*n*-hexane) as a white solid (57 mg, 39%).

$\boldsymbol{R_f}$ (30% EtOAc/*n*-hexane) = 0.35; **^{1}H NMR** (700 MHz, $CDCl_3$) δ = 7.36 (d, *J* = 7.7 Hz, 1H, H22), 7.24 – 7.20 (m, 2H, H11), 7.15 (d, *J* = 2.0 Hz, 1H, H25), 7.11 – 7.06 (m, 4H, H10+H18+H12),

7.03 (dd, *J* = 7.8, 2.0 Hz, 1H, H23), 6.97 (m, 1H, H19), 6.88 (td, *J* = 7.6, 1.3 Hz, 1H, H17), 6.70 (dd, *J* = 15.0, 11.0 Hz, 1H, H3), 6.53 (dd, *J* = 7.8, 1.4 Hz, 1H, H16), 6.13 (d, *J* = 15.0 Hz, 1H, H2), 3.96 (dt, *J* = 11.6, 8.3 Hz, 1H, H5), 3.71 (m, 3H, H4+H13/H14), 3.41 (m, 2H, H13/H14), 2.98 (t, *J* = 10.9 Hz, 1H, H6), 2.96 – 2.75 (m, 4H, H13/H14+H6), 2.48 (ddd, *J* = 10.7, 8.2, 2.8 Hz, 1H, H6), 2.36 (s, 3H, C28), 2.32 (s, 3H, C27), 1.31 (s, 6H, H8), 1.30 (s, 6H, H8), 1.24 (s, 6H, H8), 1.22 (s, 6H, H8) ppm; **^{13}C NMR** (100 MHz, $CDCl_3$) δ = 165.7 (C1), 148.7 (C15), 145.5 (C3), 142.4 (C9), 141.7 (C24), 139.4 (C21), 136.2 (C22), 134.6 (C20), 131.9 (C25), 128.1 (C11), 128.0 (C23), 127.9 (C18), 127.4 (C10), 126.6 (C16), 125.9 (C26), 125.7 (C12), 124.9 (C17), 121.8 (C2), 120.1 (C19), 83.70 (C7), 83.69, 52.2 (C13/14), 51.6 (C13/14), 49.5 (C4), 46.3 (C13/14), 43.7 (C5), 42.2 (C13/14), 28.6 (C6), 25.0 (C8), 24.9 (C8), 24.7 (C8), 21.3 (C28), 20.7 (C27) ppm; **^{11}B NMR** (128 MHz, $CDCl_3$) δ = 35.07 ppm; **IR** (ATR): ῦ = 617, 669, 699, 730, 759, 815, 847, 967, 1029, 1108, 1138, 1221, 1270, 1371, 1438, 1470, 1508, 1580, 1649, 1655, 2375, 2976 cm^{-1}; **HRMS** (ESI) $[M+Na]^+$ Calculated mass for $C_{43}H_{56}B_2N_2SO_5Na$: 757.3988; Mass found: 757.4078.

Scale Up Synthesis of 5

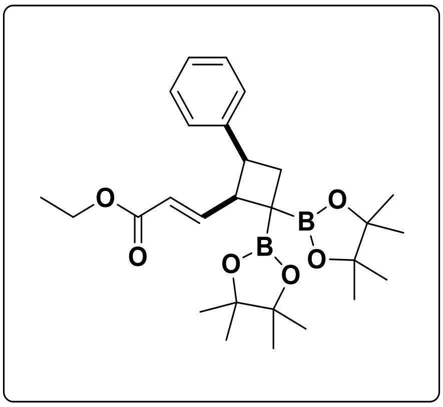

To an oven-dried 100 mL Schlenk flask equipped with a magnetic stirring bar were added $[Ru(dmbpy)_3](PF_6)_2$ (45 mg, 0.5 mol%) and substrate **1** (3.78 g, 10 mmol). The Schlenk flask was sealed with a septum and purged with nitrogen before the sequential addition of the styrene (5.75 mL, 50 mmol, 5 equiv.) and deoxygenated MeCN (50 mL, 0.2 M) via syringe. The reaction mixture was stirred under light irradiation (440 nm, 20 W) for 30 h. After completion, reaction mixture was concentrated in vacuo. The crude residue was purified by flash column chromatography (SiO_2, 10% EtOAc/*n*-hexane) to afford mixture of diastereomers **5** yielding a white solid (74%, 3.56 g, d.r.=2.5:1). The major *syn*-diastereomer **5** was isolated by a second column chromatography (SiO_2, 0%→10% EtOAc/*n*-hexane) as a white solid (2.02 g, 42%).

Cyclic Voltammetry Studies

Cyclic voltammetry was conducted on an Interface 1000 Gamry potentiostat using a 3-electrode cell configuration. A glassy carbon working electrode was employed alongside a platinum wire counter electrode and an Ag/Ag^+ reference electrode. The solution was deoxygenated by bubbling nitrogen prior to measurements. The analysis was carried out on a 5 mM solution in MeCN along with 0.1 M of tetrabutylammonium hexafluorophosphate as supporting electrolyte and with 5 mM of ferrocene as internal standard. It was examined at a scan rate of 0.05 V s^{-1}.

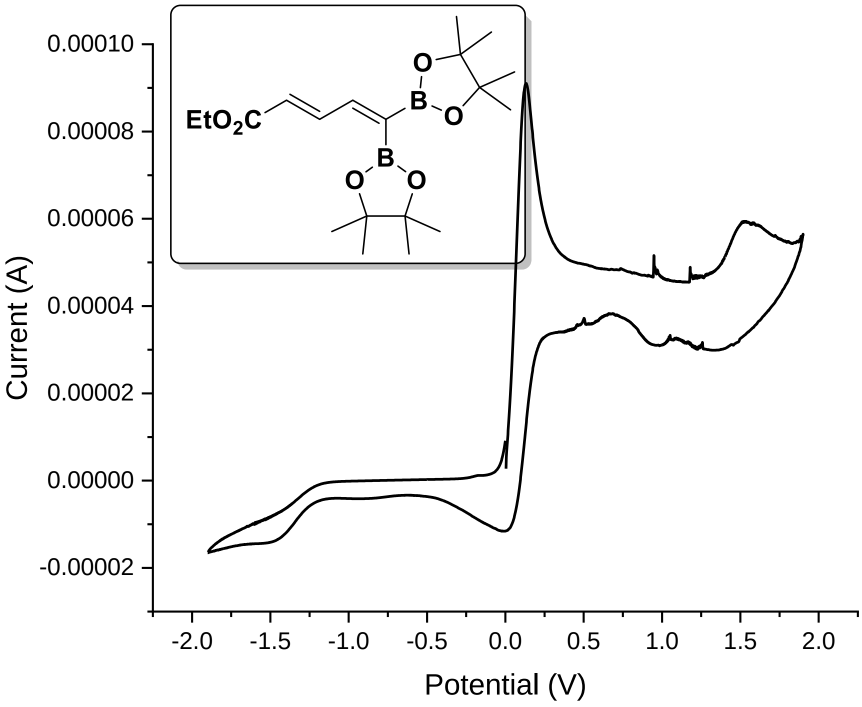

Figure S3: CV of **1.**

Comment: The potential was sweeped between -2 V and 2 V vs SCE. The catalyst used in the reaction, $[Ru(dmbpy)_3](PF_6)_2$, possesses an excited state oxidation potential of 0.22 V vs SCE.[16]

Its excited state reduction potential is not available from experiment data. However, it can be derived from Rehm Weller equation:[17] $E^*(M^*/M^+) = E(M/M^+) - E_{00}$; $E^*(M^*/M^-) = E(M/M^-) + E_{00}$. The excited state reduction potential is at -0.51 V vs SCE by calculation.

The oxidation and reduction potentials of **1** fall outside of the redox range of the photocatalyst, this indicates that single electron transfer between photocatalyst and substrate would not be an inefficient pathway.

Besides, substrate **1** exhibited same reactivity when $[Ru(phen)_3]Cl_2$ was used as the photocatalyst (optimization table), which possesses an excited state oxidation potential of 0.82 V vs SCE, and an excited state reduction potential of -0.87 V vs SCE. The oxidation and reduction potentials of **1** fall outside of the redox range of $[Ru(phen)_3]Cl_2$, further supporting that single electron transfer between photocatalyst and substrate would not be an efficient pathway.

Reaction Optimization of Housane Formation

Table S4. Reaction optimization of stereoselective synthesis of housane

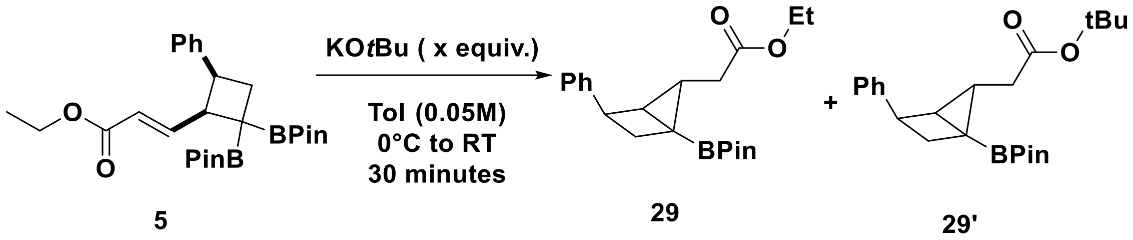

entry	x equiv.	**29**[b][c]	**29'**[b][c]
1	1.5	79%	<5%
2	1.8	71%	<5%
3	2	65%	23%
4	2.2	17%	45%
5	3	22%	27%
6[d]	1.5	63%	<5%
7[e]	1.5	<5%	<5%
8[f]	1.5	52%	<5%
9[g]	1.5	<5%	<5%
10[h]	1.5	<5%	<5%

[a] Standard conditions: **5** (0.1 mmol, 1 equiv.), KO*t*Bu (x equiv., 1 M in THF), Tol (0.05 M), 0 °C to rt, 30 minutes; [b] Determined by ^{1}H NMR spectroscopy against a known internal standard (1, 3, 5-trimethoxybenzene); [c] d.r.=>95:5; [d] THF instead of Tol; [e] KOH instead of KO*t*Bu; [f] KOH instead of KO*t*Bu, DMSO instead of Tol; [g] CsF instead of KO*t*Bu; [h] CsF instead of KO*t*Bu, DMSO instead of Tol.

Substrate Scope of Housanes

General Procedure H:

To an oven-dried microwave vial, the *syn*-isomer of [2 + 2] cycloadduct (1 equiv.) was dissolved in dry toluene (0.05 M) under an argon atmosphere. The reaction mixture was cooled to 0 °C before the dropwise addition of KO*t*Bu (1.5 equiv., 1 M in THF). Then the reaction mixture was allowed to warm to ambient temperature and stirred for another 30 minutes. After completion, the reaction was quenched by addition of H_2O (5 mL). The mixture was then extracted with Et_2O (3 × 10 mL). The combined organic phase was dried over Na_2SO_4, concentrated *in vacuo*, and purified by flash column chromatography ($B(OH)_3$-SiO_2, specified combination of solvents).

General Procedure I:

To an oven-dried microwave vial, the *syn*-isomer of [2 + 2] cycloadduct (1 equiv.) was dissolved in dry toluene (0.05 M) under an argon atmosphere. The reaction mixture was cooled to 0 °C before the dropwise addition of KO*t*Bu (2 equiv., 1 M in THF). Then the reaction mixture was allowed to warm to ambient temperature and stirred for another 1 h. After completion, the reaction was quenched by addition of H_2O (5 mL). The mixture was then extracted with Et_2O (3 × 10 mL). The combined organic phase was dried over Na_2SO_4, concentrated *in vacuo*, and purified by flash column chromatography ($B(OH)_3$-SiO_2, specified combination of solvents).

Failed examples:

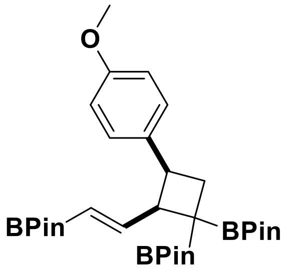

only deborylative product observed

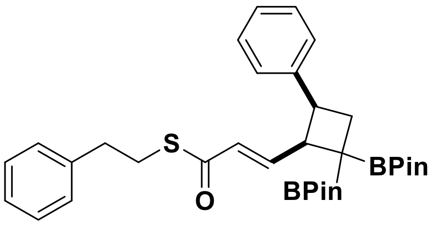

no product observed

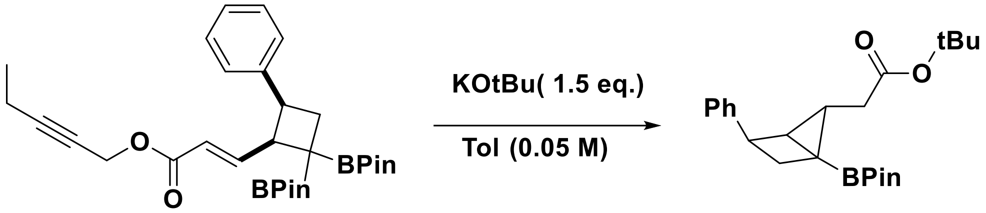

only product obsereved

(5-(2-Ethoxy-2-oxoethyl)-3-phenylbicyclo[2.1.0]pentan-1-yl)boronic acid, pinacol ester (29)

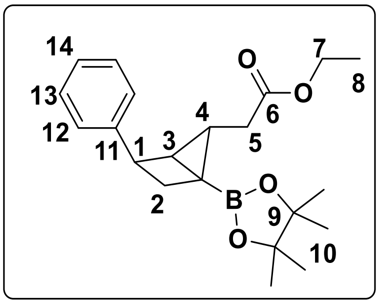

Prepared according to General Procedure **H**, **5** (965 mg, 2 mmol), in the presence of KO*t*Bu (3 mL, 1.5 equiv., 1 M in THF), was converted to **29** yielding a colorless oil as the single diastereomer (513 mg, 72%, d.r.=> 95:5), after purification by flash column chromatography ($B(OH)_3$-SiO_2, 0 → 1% EtOAc/*n*-hexane).

R$_f$ (10% EtOAc/*n*-hexane) = 0.55; **^{1}H NMR** (400 MHz, $CDCl_3$) δ = 7.30 – 7.24 (m, 2H, H13), 7.21 – 7.17 (m, 2H, H12), 7.14 (m, 1H, H14), 4.14 (q, *J* = 7.1 Hz, 2H, H7), 3.72 (dt, *J* = 10.3, 4.9 Hz, 1H, H1), 2.66 (t, *J* = 10.9 Hz, 1H, H2), 2.51 (dd, *J* = 16.9, 7.2 Hz, 1H, H5), 2.44 (dd, *J* = 16.9, 7.6 Hz, 1H, H5), 2.10 (m, 1H, H3), 1.64 (td, *J* = 7.4, 1.9 Hz, 1H, H4), 1.60 – 1.54 (m, 1H, H2), 1.29 – 1.24 (m, 9H, H8+H10), 1.23 (s, 6H, H10) ppm; **^{13}C NMR** (100 MHz, $CDCl_3$) δ = 173.3 (C6), 144.0 (C11), 128.1 (C13), 126.8 (C12), 125.5 (C14), 83.2 (C9), 60.3 (C7), 36.4 (C1), 34.4 (C5), 31.7 (C2), 30.7 (C3), 29.2 (C4), 25.2 (C10), 24.7 (C10), 14.5 (C8) ppm; **^{11}B NMR** (128 MHz, $CDCl_3$) δ = 33.04 ppm; **IR** (ATR): ῦ = 697, 750, 796, 875, 969, 997, 1029, 1126, 1159, 1195, 1270, 1300, 1350, 1369, 1447, 1474, 1491, 1508, 1544, 1602, 1714, 2375, 2978 cm^{-1}; **HRMS** (ESI) $[M+Na]^+$ Calculated mass for $C_{21}H_{29}BO_4Na$: 379.2051; Mass found: 379.2045.

(5-(2-Ethoxy-2-oxoethyl)-3-(*p*-tolyl)bicyclo[2.1.0]pentan-1-yl)boronic acid, pinacol ester (30)

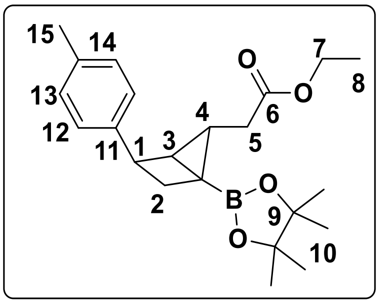

Prepared according to General Procedure **H**, **6** (993 mg, 2 mmol), in the presence of KO*t*Bu (3 mL, 1.5 equiv., 1 M in THF), was converted to **30** yielding a colorless oil as the single diastereomer (563 mg, 76%, d.r.=> 95:5), after purification by flash column chromatography ($B(OH)_3$-SiO_2, 0 → 1% EtOAc/*n*-hexane).

R$_f$ (10% EtOAc/*n*-hexane) = 0.55; **^{1}H NMR** (400 MHz, $CDCl_3$) δ = 7.08 (m, 4H, H12+H13), 4.14 (q, *J* = 7.1 Hz, 2H, H7), 3.68 (dt, *J* = 10.2, 4.9 Hz, 1H, H1), 2.63 (t, *J* = 10.8 Hz, 1H, H2), 2.55 – 2.38 (m, 2H, H5), 2.30 (s, 3H, H15), 2.08 (m, 1H, H3), 1.64 (td, *J* = 7.4, 1.9 Hz, 1H, H4), 1.55 (ddd, *J* = 11.0, 5.0, 1.3 Hz, 1H, H2), 1.27 (t, *J* = 7.2 Hz, 3H, H8), 1.24 (s, 6H, H10), 1.23 (s, 6H, H10) ppm; **^{13}C NMR** (151 MHz, $CDCl_3$) δ = 173.3 (C6), 140.9 (C11), 134.9 (C14), 128.8 (C13), 126.6 (C12), 83.1 (C9), 60.3 (C7), 36.0 (C1), 34.4 (C5), 31.7 (C2), 30.9 (C3), 29.1 (C4), 25.1 (C10), 24.7 (C10), 21.1 (C15), 14.4 (C8) ppm; **^{11}B NMR** (192 MHz, $CDCl_3$) δ = 32.65 ppm; **IR** (ATR): ῦ = 615, 669, 694, 722, 812, 854, 1035, 1135, 1214, 1302, 1339, 1369, 1389, 1516, 1731, 2375, 2927, 2977 cm^{-1}; **HRMS** (ESI) $[M+Na]^+$ Calculated mass for $C_{22}H_{31}BO_4Na$: 393.2207; Mass found: 393.2184.

(5-(2-Ethoxy-2-oxoethyl)-3-(4-methoxyphenyl)bicyclo[2.1.0]pentan-1-yl)boronic acid, pinacol ester (31)

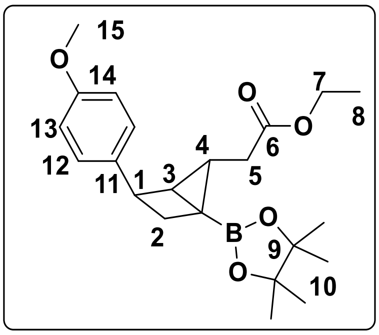

Prepared according to General Procedure **H**, **7** (420 mg, 0.82 mmol), in the presence of KO*t*Bu (1.23 mL, 1.5 equiv., 1 M in THF), was converted to **31** yielding a colorless oil as the single diastereomer (260 mg, 82%, d.r.=> 95:5), after purification by flash column chromatography ($B(OH)_3$-SiO_2, 0 → 3% EtOAc/*n*-hexane).

R

R_f (10% EtOAc/*n*-hexane) = 0.40; **^{1}H NMR** (400 MHz, $CDCl_3$) δ = 7.12 (d, *J* = 7.9 Hz, 2H, H12), 6.82 (d, *J* = 8.8 Hz, 2H, H13), 4.14 (q, *J* = 7.1 Hz, 2H, H7), 3.78 (s, 3H, H15), 3.66 (dt, *J* = 10.1, 4.9 Hz, 1H, H1), 2.62 (t, *J* = 10.8 Hz, 1H, H2), 2.51 (dd, *J* = 16.9, 7.1 Hz, 1H, H5), 2.42 (dd, *J* = 16.9, 7.7 Hz, 1H, H5), 2.07 (m, 1H, H3), 1.65 (td, *J* = 7.4, 1.9 Hz, 1H, H4), 1.52 (ddd, *J* = 11.0, 5.0, 1.3 Hz, 1H, H2), 1.27 (t, *J* = 7.2 Hz, 3H, H8), 1.24 (s, 6H, H10), 1.23 (s, 6H, H10) ppm; **^{13}C NMR** (100 MHz, $CDCl_3$) δ = 173.4 (C6), 157.6 (C14), 136.2 (C11), 127.8 (C12), 113.6 (C13), 83.1 (C9), 60.3 (C7), 55.4 (C15), 35.7 (C1), 34.4 (C5), 31.8 (C2), 30.9 (C3), 29.1 (C4), 25.2 (C10), 24.7 (C10), 14.5 (C8) ppm; **^{11}B NMR** (128 MHz, $CDCl_3$) δ = 33.13 ppm; **IR** (ATR): ῦ = 670, 753, 828, 855, 966, 1035, 1099, 1111, 1137, 1244, 1270, 1299, 1371, 1389, 1511, 1610, 1731, 2931, 2977 cm^{-1}; **HRMS** (ESI) $[M+Na]^+$ Calculated mass for $C_{22}H_{31}BO_5Na$: 409.2157; Mass found: 409.2194.

(3-(3', 4'-Dimethoxy-[1, 1'-biphenyl]-4-yl)-5-(2-ethoxy-2-oxoethyl)bicyclo[2.1.0]pentan-1-yl)boronic acid, pinacol ester (32)

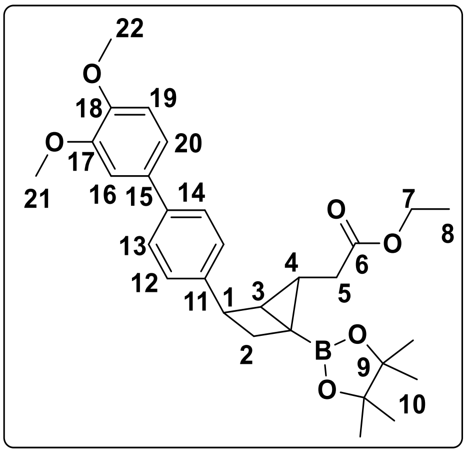

Prepared according to General Procedure **H**, **8** (50 mg, 0.08 mmol), in the presence of KO*t*Bu (0.12 mL, 1.5 equiv., 1 M in THF), was converted to **32** yielding a white solid as the single diastereomer (27 mg, 68%, d.r.=> 95:5), after purification by flash column chromatography ($B(OH)_3$-SiO_2, 0 → 10% EtOAc/*n*-hexane).

R_f (10% EtOAc/*n*-hexane) = 0.15; **^{1}H NMR** (400 MHz, $CDCl_3$) δ = 7.47 (d, *J* = 7.8 Hz, 2H, H12), 7.26 (d, *J* = 8.1 Hz, 2H, H13), 7.12 (dd, *J* = 8.2, 2.2 Hz, 2H, H20), 7.09 (s, 1H, H16), 6.93 (d, *J* = 8.2 Hz, 1H, H19), 4.15 (q, *J* = 7.1 Hz, 2H, H7), 3.94 (s, 3H, H21), 3.92 (s, 3H, H22), 3.75 (dt, *J* = 10.1, 4.8 Hz, 1H, H1), 2.69 (t, *J* = 10.9 Hz, 1H, H2), 2.54 (dd, *J* = 17.0, 7.0 Hz, 1H, H5), 2.45 (dd, *J* = 17.0, 7.8 Hz, 1H, H5), 2.13 (m, 1H, H3), 1.69 (td, *J* = 7.3, 1.9 Hz, 1H, H4), 1.60 (dd, *J* = 11.1, 5.0 Hz, 1H, H2), 1.28 (m, 3H, H8), 1.25 (s, 6H), 1.24 (s, 6H) ppm; **^{13}C NMR** (100 MHz, $CDCl_3$) δ = 173.3 (C6), 149.2 (C17), 148.5 (C18), 142.7 (C11), 138.4 (C14), 134.5 (C15), 127.2 (C13), 126.7 (C12), 119.4 (C20), 111.6 (C19), 110.6 (C16), 83.2 (C9), 60.4 (C7), 56.13 (C22), 56.06 (C21), 36.2 (C1), 34.4 (C5), 31.8 (C2), 30.7 (C3), 29.2 (C4), 25.2 (C10), 24.7 (C10), 14.5 (C8) ppm; **^{11}B NMR** (128 MHz, $CDCl_3$) δ = 33.28 ppm; **IR** (ATR): ῦ = 614, 669, 709, 732, 799, 831, 854, 881, 966, 1025, 1098, 1137, 1167, 1217, 1271, 1299, 1371, 1389, 1412, 1429, 1501, 1526, 1587, 1605, 1731, 2835, 2931, 2977 cm^{-1}; **HRMS** (ESI) $[M+Na]^+$ Calculated mass for $C_{29}H_{37}BO_6Na$: 515.2575; Mass found: 515.2589.

(5-(2-Ethoxy-2-oxoethyl)-3-(4-fluorophenyl)bicyclo[2.1.0]pentan-1-yl)boronic acid, pinacol ester (33)

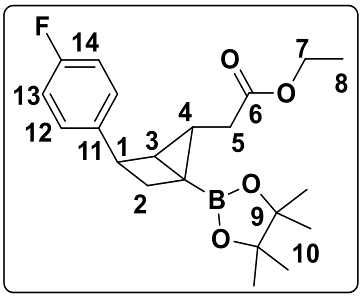

Prepared according to General Procedure **H**, **9** (70 mg, 0.14 mmol), in the presence of KO*t*Bu (0.21 mL, 1.5 equiv., 1 M in THF), was converted to **33** yielding a colorless oil as the single diastereomer (34 mg, 65%, d.r.=> 95:5), after purification by flash column chromatography ($B(OH)_3$-SiO_2, 0→1% EtOAc/*n*-hexane).

R_f (10% EtOAc/*n*-hexane) = 0.55; **^{1}H NMR** (400 MHz, $CDCl_3$) δ = 7.14 (m, 2H, H12), 6.98 – 6.91 (m, 2H, H13), 4.14 (q, *J* = 7.1 Hz, 2H, H7), 3.67 (dt, *J* = 10.3, 4.9 Hz, 1H, H1), 2.64 (t, *J* = 10.8 Hz, 1H, H2), 2.53 (dd, *J* = 17.0, 6.8 Hz, 1H, H5), 2.41 (dd, *J* = 17.0, 8.0 Hz, 1H, H5), 2.08 (m, 1H, H3), 1.60 (ddd, *J* = 8.4, 6.9, 1.9 Hz, 1H, H4), 1.51 (ddd, *J* = 11.1, 5.0, 1.3 Hz, 1H, H2), 1.26 (t, *J* = 7.1 Hz, 3H, H8), 1.24 (s, 6H, H10), 1.23 (s, 6H, H10) ppm; **^{13}C NMR** (100 MHz, $CDCl_3$) δ = 173.3 (C6), 161.1 (d, *J* = 242.7 Hz, C14)), 139.6 (d, *J* = 3.1 Hz, C11), 128.2 (d, *J* = 7.8 Hz, C12), 114.8 (d, *J* = 21.0 Hz, C13), 83.2 (C9), 60.4 (C7), 35.7 (C1), 34.3 (C5), 31.9 (C2), 30.6 (C3), 29.1 (C4), 25.1 (C10), 24.7 (C10), 14.4 (C8) ppm; **^{11}B NMR** (128 MHz, $CDCl_3$) δ = 33.23 ppm; **^{19}F NMR** (376 MHz, $CDCl_3$) δ = -118.26 (td, *J* = 8.9, 4.9 Hz) ppm; **IR** (ATR): ῦ = 673, 691, 719, 765, 815, 855, 964, 1017, 1052, 1070, 1093, 1138, 1149, 1161, 1187, 1207, 1226, 1238, 1270, 1302, 1336, 1372, 1411, 1424, 1452, 1507, 1600, 1731, 2934, 2977 cm^{-1}; **HRMS** (ESI) $[M+Na]^+$ Calculated mass for $C_{21}H_{28}BFO_4Na$: 397.1957; Mass found: 397.1954.

(3-(2-Bromophenyl)-5-(2-ethoxy-2-oxoethyl)bicyclo[2.1.0]pentan-1-yl)boronic acid, pinacol ester (34)

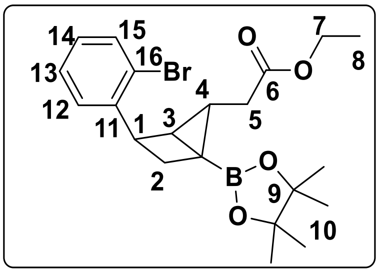

Prepared according to General Procedure **H**, **11** (45 mg, 0.08 mmol), in the presence of KO*t*Bu (mL, 1.5 equiv., 1 M in THF), was converted to **34** yielding a white solid as the single diastereomer (39 mg, 52%, d.r.=> 95:5), after purification by flash column chromatography ($B(OH)_3$-SiO_2, 0 → 1% EtOAc/*n*-hexane).

R_f (10% EtOAc/*n*-hexane) = 0.50; **^{1}H NMR** (400 MHz, $CDCl_3$) δ = 7.64 (dt, *J* = 7.6, 1.2 Hz, 1H, H15), 7.47 (dd, *J* = 8.0, 1.3 Hz, 1H, H12), 7.30 (dd, *J* = 7.9, 6.6 Hz, 1H, H14), 7.05 (td, *J* = 7.6, 1.7 Hz, 1H, H13), 4.20 (q, *J* = 7.1 Hz, 2H, H7), 3.78 (dt, *J* = 10.1, 4.9 Hz, 1H, H1), 2.83 (t, *J* = 10.9 Hz, 1H, H2), 2.63 (dd, *J* = 17.1, 6.5 Hz, 1H, H5), 2.48 (dd, *J* = 17.1, 8.3 Hz, 1H, H5), 2.11 (m, 1H, H3), 1.68 (ddd, *J* = 8.3, 6.5, 1.8 Hz, 1H, H4), 1.50 (m, 1H, H2), 1.31 (t, *J* = 7.1 Hz, 3H, H8), 1.27 (s, 6H, H10), 1.27 (s, 6H, H10) ppm; **^{13}C NMR** (100 MHz, $CDCl_3$) δ = 173.3 (C6), 142.3 (C11), 132.6 (C12), 129.1 (C15), 127.5 (C13), 127.0 (C14), 123.7 (C16), 83.2 (C9), 60.4 (C7), 38.0 (C1), 34.4 (C5), 32.6 (C2), 29.8 (C4), 29.5 (C3), 25.14(C10), 24.7 (C10), 14.5 (C8) ppm; **^{11}B NMR** (128 MHz, $CDCl_3$) δ = 32.79 ppm; **IR** (ATR): ῦ = 658, 673, 753, 792, 835, 855, 967, 1019, 1037, 1101, 1138, 1181, 1223, 1300, 1369, 1378, 1388, 1411, 1425, 1508, 1564, 1734, 2375, 2931, 2977 cm^{-1}; **HRMS** (ESI) $[M+Na]^+$ Calculated mass for $C_{21}H_{28}BBrO_4Na$: 457.1156; Mass found: 457.1191.

(3-(4-Bromophenyl)-5-(2-ethoxy-2-oxoethyl)bicyclo[2.1.0]pentan-1-yl)boronic acid, pinacol ester (35)

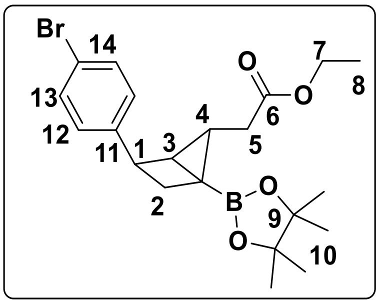

Prepared according to General Procedure **H**, **10** (95 mg, 0.17 mmol), in the presence of KO*t*Bu (0.26 mL, 1.5 equiv., 1 M in THF), was converted to **35** yielding a white solid as the single diastereomer (47 mg, 63%, d.r.=> 95:5), after purification by flash column chromatography ($B(OH)_3$-SiO_2, 0→1% EtOAc/*n*-hexane).

R

R_f (10% EtOAc/*n*-hexane) = 0.50; **^{1}H NMR** (600 MHz, $CDCl_3$) δ = 7.38 (d, *J* = 8.4 Hz, 2H, H13), 7.07 (d, *J* = 7.4 Hz, 2H, H12), 4.14 (q, *J* = 7.2 Hz, 2H, H7), 3.64 (dt, *J* = 10.2, 4.8 Hz, 1H, H1), 2.65 (t, *J* = 10.9 Hz, 1H, H2), 2.52 (dd, *J* = 17.0, 6.8 Hz, 1H, H5), 2.40 (dd, *J* = 17.0, 8.0 Hz, 1H, H5), 2.07 (m, 1H, H3), 1.57 (ddd, *J* = 8.4, 6.8, 1.9 Hz, 1H, H4), 1.51 (ddd, *J* = 11.1, 5.0, 1.3 Hz, 1H, H2), 1.27 (t, *J* = 7.1 Hz, 3H, H8), 1.24 (s, 6H, H10), 1.23 (s, 6H, H10) ppm; **^{13}C NMR** (100 MHz, $CDCl_3$) δ = 173.2 (C6), 143.0 (C11), 131.2 (C13), 128.7 (C12), 119.3 (C14), 83.2 (C9), 60.4 (C7), 35.9 (C1), 34.3 (C5), 31.8 (C2), 30.4 (C3), 29.1 (C4), 25.1 (C10), 24.7 (C10), 14.5 (C8) ppm; **^{11}B NMR** (128 MHz, $CDCl_3$) δ = 33.11 ppm; **IR** (ATR): ῦ = 650, 671, 703, 719, 753, 822, 852, 947, 967, 981, 1013, 1072, 1102, 1138, 1165, 1223, 1267, 1304, 1371, 1389, 1411, 1566, 1735, 2859, 2920, 2977 cm^{-1}; **HRMS** (ESI) $[M+Na]^+$ Calculated mass for $C_{21}H_{28}BBrO_4Na$:457.1156; Mass found:457.1170.

(3-(3-Cyanophenyl)-5-(2-ethoxy-2-oxoethyl)bicyclo[2.1.0]pentan-1-yl)boronic acid, pinacol ester (36)

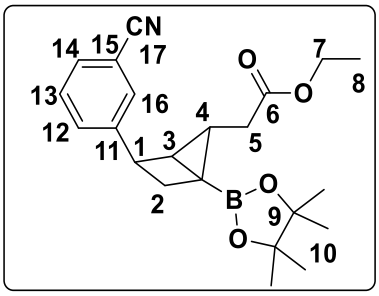

Prepared according to General Procedure **H**, **12** (112 mg, 0.22 mmol), in the presence of KO*t*Bu (0.33 mL, 1.5 equiv., 1 M in THF), was converted to **36** yielding a colorless oil as the single diastereomer (59 mg, 70%, d.r.=> 95:5), after purification by flash column chromatography ($B(OH)_3$-SiO_2, 0 → 10% EtOAc/*n*-hexane).

R_f (15% EtOAc/*n*-hexane) = 0.25; **^{1}H NMR** (400 MHz, $CDCl_3$) δ = 7.49 (m, 1H, H14), 7.46 – 7.41 (m, 2H, H16+H12), 7.39 – 7.34 (m, 1H, H13), 4.17 (q, *J* = 7.1 Hz, 2H, H7), 3.70 (dt, *J* = 10.2, 4.7 Hz, 1H, H1), 2.70 (t, *J* = 10.9 Hz, 1H, H2), 2.55 (dd, *J* = 17.1, 6.7 Hz, 1H, H5), 2.42 (dd, *J* = 17.1, 8.1 Hz, 1H, H5), 2.10 (m, 1H, H3), 1.55 – 1.49 (m, 2H, H4+H2), 1.27 (t, *J* = 7.2 Hz, 3H, H8), 1.24 (s, 6H, H10), 1.23 (s, 6H, H10) ppm; **^{13}C NMR** (100 MHz, $CDCl_3$) δ = 173.1 (C6), 145.4 (C11), 131.5 (C12), 130.5 (C14), 129.4 (C16), 129.0 (C13), 119.4 (C17), 112.2 (C15), 83.4 (C9), 60.5 (C7), 35.9 (C1), 34.2 (C5), 31.8 (C2), 29.9 (C3), 29.2 (C4), 25.1 (C10), 24.7 (C10), 14.5 (C8) ppm; **^{11}B NMR** (128 MHz, $CDCl_3$) δ = 33.03 ppm; **IR** (ATR): ῦ = 644, 669, 694, 740, 773, 782, 798, 852, 941, 966, 1035, 1101, 1137, 1165, 1185, 1223, 1238, 1270, 1302, 1371, 1389, 1415, 1508, 1729, 2229, 2375, 2928, 2978 cm^{-1}; **HRMS** (ESI) $[M+Na]^+$ Calculated mass for $C_{22}H_{28}BNO_4Na$: 404.2003; Mass found: 404.2050.

(5-(2-Ethoxy-2-oxoethyl)-3-(1-methyl-1*H*-indol-3-yl)bicyclo[2.1.0]pentan-1-yl)boronic acid, pinacol ester (37)

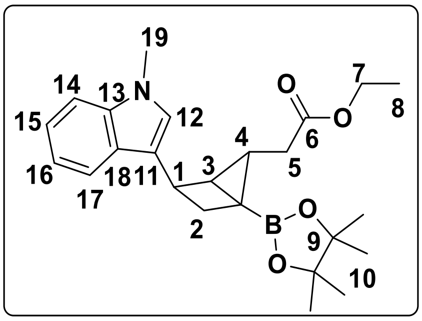

Prepared according to General Procedure **H**, **14** (64 mg, 0.12 mmol), in the presence of KO*t*Bu (0.18 mL, 1.5 equiv., 1 M in THF), was converted to **37** yielding a light-yellow oil as the single diastereomer (37 mg, 75%, d.r.=>95:5), after purification by flash column chromatography (B(OH)$_3$-SiO$_2$, 0→10% EtOAc/*n*-hexane).

***R**$_f$* (15% EtOAc/*n*-hexane) = 0.35; **^{1}H NMR** (400 MHz, CDCl$_3$) δ = 7.46 (dt, *J* = 7.9, 1.0 Hz, 1H, H14), 7.26 (m, 1H, H17), 7.18 (ddd, *J* = 8.2, 6.9, 1.2 Hz, 1H, H15), 7.05 (ddd, *J* = 7.9, 6.9, 1.1 Hz, 1H, H16), 6.99 (d, *J* = 1.2 Hz, 1H, H12), 4.12 (q, *J* = 7.2 Hz, 2H, H7), 3.89 (dt, *J* = 9.9, 4.8 Hz, 1H, H1), 3.75 (s, 3H, H19), 2.67 (t, *J* = 10.6 Hz, 1H, H2), 2.51 (dd, *J* = 17.2, 7.4 Hz, 1H, H2), 2.42 (dd, *J* = 17.1, 7.5 Hz, 1H, H2), 2.13 (m, 1H, H3), 1.88 (td, *J* = 7.5, 1.9 Hz, 1H, H4), 1.61 – 1.56 (m, 1H, H2), 1.25 (s, 6H, H10), 1.25 – 1.20 (m, 9H, H8+H10 ppm); **^{13}C NMR** (100 MHz, CDCl$_3$) δ = 173.6 (C6), 137.2 (C13), 127.3 (C18), 126.4 (C12), 121.5 (C15), 119.2 (C14), 118.6 (C16), 117.3 (C11), 109.2 (C17), 83.1 (C9), 60.3 (C7), 34.3 (C5), 32.8 (C19), 31.5 (C2), 31.4 (C3), 29.4 (C1), 28.6 (C4), 25.2 (C10), 24.8 (C10), 14.4 (C8) ppm; **^{11}B NMR** (128 MHz, CDCl$_3$) δ = 32.86 ppm; **IR** (ATR): ῦ = 644, 669, 737, 782, 854, 941, 966, 983, 1033, 1098, 1137, 1184, 1223, 1238, 1269, 1299, 1327, 1371, 1388, 1471, 1508, 1729, 2375, 2928, 2977 cm^{-1}; **HRMS** (ESI) [M+Na]$^+$ Calculated mass for $C_{24}H_{32}BNO_4Na$: 432.2316; Mass found: 432.2348.

(3-(Benzofuran-5-yl)-5-(2-ethoxy-2-oxoethyl)bicyclo[2.1.0]pentan-1-yl)boronic acid, pinacol ester (38)

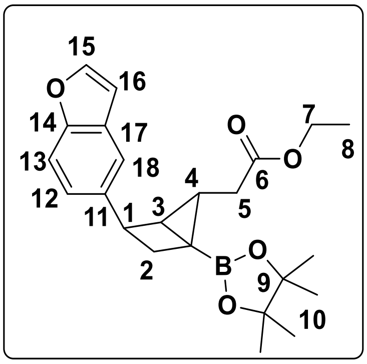

Prepared according to General Procedure **H**, **13** (125 mg, 0.24 mmol), in the presence of KO*t*Bu (0.36 mL, 1.5 equiv., 1 M in THF), was converted to **38** yielding a white solid as the single diastereomer (69 mg, 73%, d.r.=>95:5), after purification by flash column chromatography (B(OH)$_3$-SiO$_2$, 0→2% EtOAc/*n*-hexane).

***R**$_f$* (10% EtOAc/*n*-hexane) = 0.40; **^{1}H NMR** (400 MHz, CDCl$_3$) δ = 7.58 (d, *J* = 2.2 Hz, 1H, H15), 7.42 (dd, *J* = 1.9, 1.0 Hz, 1H, H18), 7.40 (d, *J* = 8.5 Hz, 1H, H13), 7.11 (dd, *J* = 8.5, 1.7 Hz, 1H, H12), 6.71 (dd, *J* = 2.2, 0.9 Hz, 1H, H16), 4.15 (q, *J* = 7.1 Hz, 2H, H7), 3.81 (dt, *J* = 10.2, 4.9 Hz, 1H, H1), 2.70 (t, *J* = 10.8 Hz, 1H, H2), 2.53 (dd, *J* = 16.9, 7.2 Hz, 1H, H5), 2.46 (dd, *J* = 16.9, 7.6 Hz, 1H, H5), 2.15 (m, 1H, H3), 1.67 (td, *J* = 7.4, 1.9 Hz, 1H, H4), 1.60 (ddd, *J* = 11.0, 5.0, 1.3 Hz, 1H, H2), 1.29 – 1.24 (m, 9H, H8+H10), 1.24 (s, 6H, H10) ppm; **^{13}C NMR** (100 MHz, CDCl$_3$) δ = 173.3 (C6), 153.4 (C14), 145.1 (C15), 138.6 (C11), 127.3 (C17), 123.3 (C12), 118.9 (C18), 110.9 (C13), 106.6 (C16), 83.2 (C9), 60.3 (C7), 36.3 (C1), 34.4 (C5), 32.0 (C2), 31.0 (C3), 29.2 (C4), 25.2 (C10), 24.7 (C10), 14.5 (C8) ppm; **^{11}B NMR** (128 MHz, CDCl$_3$) δ = 33.28 ppm; **IR** (ATR): ῦ = 644, 666, 740, 775, 782, 819, 852, 875, 891, 907, 941, 964, 1036, 1076, 1101, 1109, 1132, 1168, 1187, 1221, 1238, 1269, 1302, 1326, 1371, 1388, 1414, 1431, 1474, 1508, 1727, 2375, 2978, 3115 cm^{-1}; **HRMS** (ESI) [M+Na]$^+$ Calculated mass for $C_{23}H_{29}BO_5Na$: 419.2000; Mass found: 419.2023.

(5-(2-Ethoxy-2-oxoethyl)-3-vinylbicyclo[2.1.0]pentan-1-yl)boronic acid, pinacol ester (39)

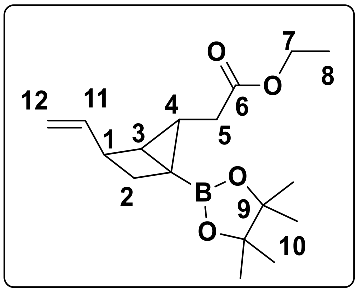

Prepared according to General Procedure **H**, **15** (61 mg, 0.14 mmol), in the presence of KO*t*Bu (0.21 mL, 1.5 equiv., 1 M in THF), was converted to **39** yielding a colorless oil as the single diastereomer (34 mg, 79%, d.r.=> 95:5), after purification by flash column chromatography ($B(OH)_3$-SiO_2, 0→1% EtOAc/*n*-hexane).

***R**$_f$* (10% EtOAc/*n*-hexane) = 0.60; **^{1}H NMR** (400 MHz, $CDCl_3$) δ = 5.76 (ddd, *J* = 17.1, 10.5, 5.5 Hz, 1H, H11), 5.02 – 4.91 (m, 2H, H12), 4.12 (q, *J* = 7.1 Hz, 2H, H7), 3.02 (dq, *J* = 10.2, 4.9 Hz, 1H, H1), 2.41 (dd, *J* = 7.4, 2.0 Hz, 2H, H5), 2.27 (t, *J* = 10.7 Hz, 1H, H2), 1.80 (dd, *J* = 4.6, 1.8 Hz, 1H, H3), 1.71 (td, *J* = 7.4, 1.9 Hz, 1H, H4), 1.35 (ddd, *J* = 11.3, 4.9, 1.4 Hz, 1H, H2), 1.25 (t, *J* = 7.1 Hz, 3H, H8), 1.21 (s, 6H, H10), 1.19 (s, 6H, H10) ppm; **^{13}C NMR** (100 MHz, $CDCl_3$) δ = 173.4 (C6), 139.5 (C11), 114.0 (C12), 83.0 (C9), 60.3 (C7), 35.7 (C1), 34.3 (C5), 30.8 (C3), 29.7 (C2), 28.0 (C4), 25.1 (C10), 24.7 (C10), 14.4 (C8) ppm; **^{11}B NMR** (128 MHz, $CDCl_3$) δ = 32.80 ppm; **IR** (ATR): ṽ = 669, 699, 739, 769, 849, 969, 1032, 1109, 1138, 1164, 1214, 1304, 1339, 1371, 1447, 1471, 1508, 1715, 2375, 2933, 2977 cm^{-1}; **HRMS** (ESI) $[M+Na]^+$ Calculated mass for $C_{17}H_{27}BO_4Na$: 329.1895; Mass found:329.1925.

(5-(2-(But-3-en-1-yloxy)-2-oxoethyl)-3-phenylbicyclo[2.1.0]pentan-1-yl)boronic acid, pinacol ester (40)

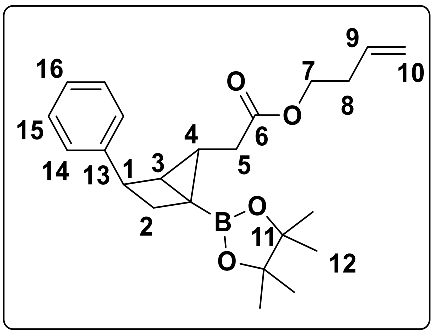

Prepared according to General Procedure **I**, **19** (147 mg, 0.29 mmol), in the presence of KO*t*Bu (0.44 mL, 2 equiv., 1 M in THF), was converted to **40** yielding a colorless oil as the single diastereomer (60 mg, 54%, d.r.=> 95:5), after purification by flash column chromatography ($B(OH)_3$-SiO_2, 0→1% EtOAc/*n*-hexane).

***R**$_f$* (10% EtOAc/*n*-hexane) = 0.60; **^{1}H NMR** (400 MHz, $CDCl_3$) δ = 7.27 (t, *J* = 7.4 Hz, 2H, H15), 7.19 (d, *J* = 8.2 Hz, 2H, H14), 7.14 (t, *J* = 7.3 Hz, 1H, H16), 5.78 (ddt, *J* = 17.0, 10.2, 6.7 Hz, 1H, H9), 5.14 – 5.01 (m, 2H, H10), 4.13 (td, *J* = 6.9, 1.2 Hz, 2H, H7), 3.72 (dt, *J* = 10.2, 4.9 Hz, 1H, H1), 2.66 (t, *J* = 10.9 Hz, 1H, H2), 2.55 – 2.41 (m, 2H, H5), 2.39 (dtd, *J* = 6.8, 5.4, 1.4 Hz, 2H, H8), 2.10 (m, 1H, H3), 1.66 – 1.61 (m, 1H, H4), 1.58 (ddd, *J* = 11.0, 5.0, 1.3 Hz, 1H, H2), 1.24 (s, 6H, H12), 1.23 (s, 6H, H12) ppm; **^{13}C NMR** (100 MHz, $CDCl_3$) δ = 173.2 (C6), 144.0 (C13), 134.2 (C9), 128.1 (C15), 126.4 (C14), 125.5 (C16), 117.3 (C10), 83.2 (C11), 63.5 (C7), 36.4 (C1), 34.4 (C5), 33.2 (C8), 31.6 (C2), 30.7 (C3), 29.1 (C4), 25.2 (C12), 24.7 (C12) ppm; **^{11}B NMR** (128 MHz, $CDCl_3$) δ = 32.91 ppm; **IR** (ATR): ṽ = 669, 699, 753, 789, 816, 855, 888, 918, 966, 984, 1026, 1101, 1135, 1237, 1271, 1300, 1371, 1389, 1416, 1448, 1617, 1735, 2930 cm^{-1}; **HRMS** (ESI) $[M+Na]^+$ Calculated mass for $C_{23}H_{31}BO_4Na$: 405.2207; Mass found: 405.2225.

(3-([1, 1'-Biphenyl]-4-yl)-5-(2-((1-(*tert*-butoxycarbonyl)piperidin-4-yl)oxy)-2-oxoethyl)bicyclo[2.1.0]pentan-1-yl)boronic acid, pinacol ester (41)

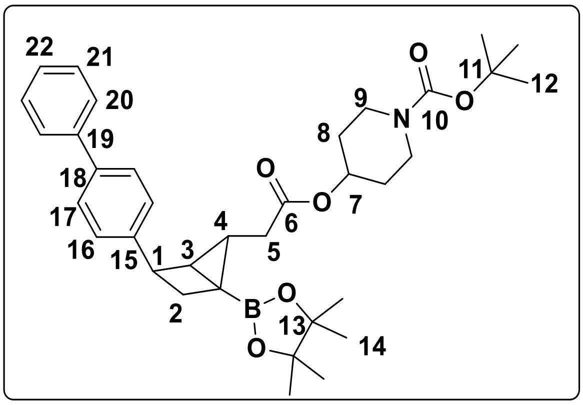

Prepared according to General Procedure **I**, **20** (150 mg, 0.21 mmol), in the presence of KO*t*Bu (0.42 mL, 2 equiv., 1 M in THF), was converted to **41** yielding a white solid as the single diastereomer (101 mg, 82%, d.r.=> 95:5), after purification by flash column chromatography ($B(OH)_3$-SiO_2, 0 → 15% EtOAc/*n*-hexane).

***R**$_f$* (20% EtOAc/*n*-hexane) = 0.20; **^{1}H NMR** (400 MHz, $CDCl_3$) δ = 7.59 – 7.55 (m, 2H, H20), 7.52 – 7.47 (m, 2H, H17), 7.42 (t, *J* = 7.6 Hz, 2H, H21), 7.31 (t, *J* = 7.3 Hz, 1H, H22), 7.26 (d, *J* = 7.9 Hz, 2H, H16), 4.95 (tt, *J* = 8.0, 3.8 Hz, 1H, H7), 3.84 – 3.62 (m, 3H, H1+H9), 3.20 (ddt, *J* = 13.1, 8.3, 3.8 Hz, 2H, H9), 2.69 (t, *J* = 10.9 Hz, 1H, H2), 2.55 (dd, *J* = 16.9, 7.0 Hz, 1H, H5), 2.44 (dd, *J* = 16.9, 7.7 Hz, 1H, H5), 2.16 – 2.10 (m, 1H, H3), 1.85 (d, *J* = 13.0 Hz, 2H, H8), 1.68 (td, *J* = 7.3, 1.9 Hz, 1H, H4), 1.64 – 1.57 (m, 3H, H8+H2), 1.44 (s, 9H, H12), 1.25 (s, 6H, H14), 1.24 (s, 6H, H14) ppm; **^{13}C NMR** (100 MHz, $CDCl_3$) δ = 172.6 (C6), 154.9 (C10), 143.1 (C15), 141.3 (C19), 138.6 (C18), 128.8 (C21), 127.2 (C20/16), 127.2 (C20/16), 127.1 (C22), 127.0 (C17), 83.2 (C13), 79.8 (C11), 69.8 (C7), 41.2 (C9), 36.2 (C1), 34.6 (C5), 31.6 (C2), 30.2 (C8), 30.7 (C3), 29.1 (C4), 28.6 (C12), 25.2 (C14), 24.8 (C14) ppm; **^{11}B NMR** (128 MHz, $CDCl_3$) δ = 33.47 ppm; **IR** (ATR): ṽ = 666, 696, 726, 759, 806, 832, 855, 900, 940, 967, 1023, 1076, 1101, 1134, 1168, 1220, 1236, 1276, 1312, 1363, 1379, 1428, 1448, 1508, 1600, 1685, 1721, 2377, 2864, 2928, 2978 cm^{-1}; **HRMS** (ESI) $[M+Na]^+$ Calculated mass for $C_{35}H_{46}BNO_6Na$: 610.3310; Mass found: 610.3313.

(5-(2-(*tert*-Butoxy)-2-oxoethyl)-3-(4-methoxyphenyl)bicyclo[2.1.0]pentan-1-yl)boronic acid, pinacol ester (42)

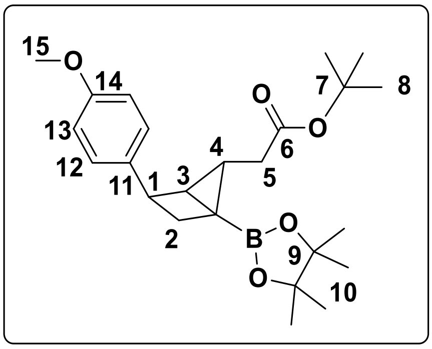

Prepared according to General Procedure **I**, **17** (648 mg, 1.2 mmol), in the presence of KO*t*Bu (2.4 mL, 2 equiv., 1 M in THF), was converted to **42** yielding a colorless oil as the single diastereomer (398 mg, 80%, d.r.=> 95:5), after purification by flash column chromatography ($B(OH)_3$-SiO_2, 0→2% EtOAc/*n*-hexane).

***R**$_f$* (10% EtOAc/*n*-hexane) = 0.48; **^{1}H NMR** (400 MHz, $CDCl_3$) δ = 7.13 – 7.08 (m, 2H, H12), 6.83 – 6.78 (m, 2H, H13), 3.78 (s, 3H, H15), 3.65 (dt, *J* = 10.2, 4.9 Hz, 1H, H1), 2.60 (t, *J* = 10.8 Hz, 1H, H2), 2.44 (dd, *J* = 16.3, 6.9 Hz, 1H, H5), 2.29 (dd, *J* = 16.3, 7.8 Hz, 1H, H5), 2.07 (dt, *J* = 4.8, 1.5 Hz, 1H, H3), 1.61 (td, *J* = 7.3, 1.9 Hz, 1H, H4), 1.52 (ddd, *J* = 11.0, 5.0, 1.3 Hz, 1H, H2), 1.45 (s, 9H, H8), 1.24 (s, 6H, H10), 1.23 (s, 6H, H10) ppm; **^{13}C NMR** (100 MHz, $CDCl_3$) δ = 172.7 (C6), 157.6 (C14), 136.3 (C11), 127.8 (C12), 113.6 (C13), 83.1 (C9), 80.0 (C7), 55.4 (C15), 35.7 (C1), 31.8 (C5), 30.7 (C2), 29.5 (C3), 28.3 (C4), 25.2 (C8), 24.8 (C10) ppm; **^{11}B NMR** (128 MHz, $CDCl_3$) δ = 33.32 ppm; **IR** (ATR): ṽ = 670, 724, 753, 808, 828, 855, 966, 1036, 1099, 1111, 1137, 1244, 1299, 1339, 1368, 1389, 1428, 1511, 1580, 1610, 1729, 2931, 2976 cm^{-1};**HRMS** (ESI) $[M+Na]^+$ Calculated mass for $C_{24}H_{35}BO_5Na$: 437.2470; Mass found: 437.2478.

(5-(2-(Methylamino)-2-oxoethyl)-3-phenylbicyclo[2.1.0]pentan-1-yl)boronic acid, pinacol ester (43)

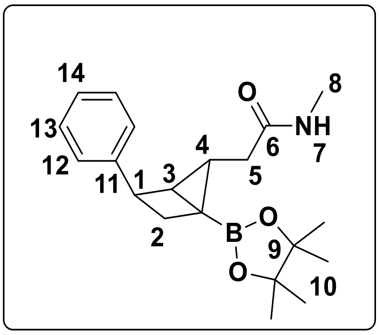

Prepared according to General Procedure **I**, **23** (55 mg, 0.11 mmol), in the presence of KO*t*Bu (0.17 mL, 2 equiv., 1 M in THF), was converted to **43** yielding a white solid as the single diastereomer (29 mg, 77%, d.r.=> 95:5), after purification by flash column chromatography ($B(OH)_3$-SiO_2, 10% → 50% EtOAc/*n*-hexane).

***R**f* (EtOAc) = 0.50; **^{1}H NMR** (400 MHz, $CDCl_3$) δ = 7.31 – 7.25 (m, 2H, H13), 7.18 – 7.09 (m, 3H, H12+H14), 6.17 (s, 1H, H7), 3.73 (dt, *J* = 10.2, 4.9 Hz, 1H, H1), 2.75 (d, *J* = 4.8 Hz, 3H, H8), 2.65 (t, *J* = 10.8 Hz, 1H, H2), 2.51 (dd, *J* = 15.5, 5.5 Hz, 1H, H5), 2.15 (dd, *J* = 15.5, 9.3 Hz, 1H, H5), 2.10 – 2.07 (m, 1H, H3), 1.60 (dtd, *J* = 11.2, 5.3, 1.6 Hz, 2H, H2+H4), 1.26 (s, 6H, H10), 1.25 (s, 6H, H10) ppm; **^{13}C NMR** (100 MHz, $CDCl_3$) δ = 173.4 (C6), 143.8 (C11), 128.3 (C13), 126.5 (C12), 125.7 (C14), 83.4 (C9), 36.5 (C5), 36.2 (C1), 31.3 (C2), 30.9 (C3), 30.0 (C4), 26.2 (C8), 25.0 (C10) ppm; **^{11}B NMR** (128 MHz, $CDCl_3$) δ = 33.13 ppm; **IR** (ATR): ῦ = 668, 700, 752, 791, 812, 848, 920, 967, 1039, 1138, 1165, 1215, 1257, 1312, 1369, 1422, 1517, 1600, 1692, 2977 cm^{-1}; **HRMS** (ESI) $[M+Na]^+$ Calculated mass for $C_{20}H_{28}BNO_3Na$:364.2054; Mass found:364.2061.

(5-(2-(9*H*-Carbazol-9-yl)-2-oxoethyl)-3-phenylbicyclo[2.1.0]pentan-1-yl)boronic acid, pinacol ester (44)

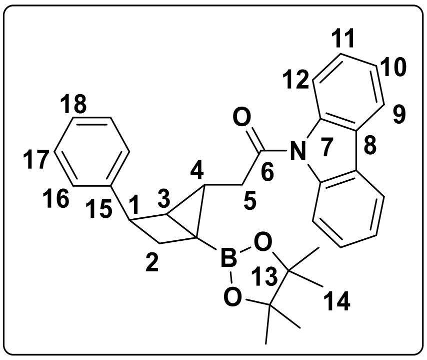

Prepared according to General Procedure **I**, **24** (157 mg, 0.26 mmol), in the presence of KO*t*Bu (0.52 mL, 2 equiv., 1 M in THF). was converted to **44** yielding a white solid as the single diastereomer (50 mg, 40%, d.r.=> 95:5), after purification by flash column chromatography ($B(OH)_3$-SiO_2, 0→2% EtOAc/*n*-hexane).

***R**f* (10% EtOAc/*n*-hexane) = 0.30; **^{1}H NMR** (400 MHz, $CDCl_3$) δ = 8.33 (d, *J* = 8.4 Hz, 2H, H12), 8.01 (ddd, *J* = 7.6, 1.5, 0.7 Hz, 2H, H9), 7.47 (ddd, *J* = 8.6, 7.3, 1.4 Hz, 2H, H11), 7.41 – 7.31 (m, 6H, H9+H16+H17), 7.19 (m, 1H, H18), 3.82 (dt, *J* = 10.2, 4.9 Hz, 1H, H1), 3.41 (dd, *J* = 17.3, 6.9 Hz, 1H, H5), 3.36 (dd, *J* = 17.3, 6.9 Hz, 1H, H5), 2.75 (t, *J* = 10.8 Hz, 1H, H2), 2.25 (m, 1H, H3), 2.04 (td, *J* = 6.9, 1.8 Hz, 1H, H4), 1.71 (ddd, *J* = 11.1, 5.0, 1.3 Hz, 1H, H2), 1.14 (s, 6H, H14), 1.07 (s, 6H, H14) ppm; **^{13}C NMR** (100 MHz, $CDCl_3$) δ = 173.2 (C6), 143.8 (C15), 138.9 (C7), 128.2 (C16/17), 127.3 (C11), 126.9 (C16/17), 126.5 (C8), 125.6 (C18), 123.5 (C10), 119.8 (C9), 116.8 (C12), 83.2 (C13), 40.0 (C5), 36.5 (C1), 31.2 (C2), 31.5 (C3), 28.7 (C4), 24.9 (C14), 24.8 (C14) ppm; **^{11}B NMR** (128 MHz, $CDCl_3$) δ = 32.12 ppm; **IR** (ATR): ῦ = 640, 670, 699, 722, 753, 822, 854, 966, 1037, 1066, 1102, 1147, 1205, 1263, 1282, 1302, 1333, 1371, 1416, 1444, 1478, 1599, 1702, 2933 cm^{-1}; **HRMS** (ESI) $[M+Na]^+$ Calculated mass for $C_{31}H_{32}BNO_3Na$: 500.2367; Mass found: 500.2366.

(5-(2-(4-(6-Fluorobenzo[d]isoxazol-3-yl)piperidin-1-yl)-2-oxoethyl)-3-(4-methoxyphenyl)bicyclo[2.1.0]pentan-1-yl)boronic acid, pinacol ester (45)

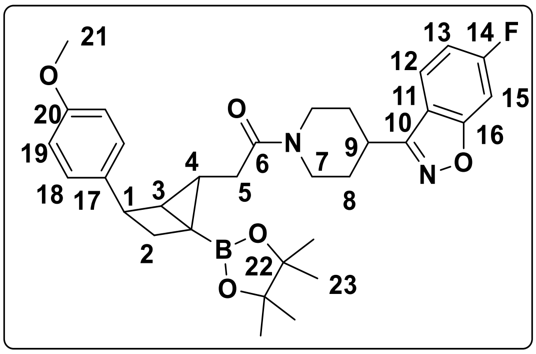

Prepared according to General Procedure **I**, **26** (48 mg, 0.07 mmol), in the presence of KO*t*Bu (0.11 mL, 1.5 equiv., 1 M in THF). was converted to **45** yielding a white solid as the single diastereomer (26 mg, 66%, d.r.=>95:5), after purification by flash column chromatography ($B(OH)_3$-SiO_2, 50% → 100% EtOAc/*n*-hexane).

R

$\boldsymbol{R_f}$ (EtOAc/) = 0.25; **^{1}H NMR** (400 MHz, $CDCl_3$) δ = 7.60 (ddd, *J* = 14.6, 8.6, 5.1 Hz, 1H, H12), 7.26 (m, 1H, H15), 7.15 (d, *J* = 8.5 Hz, 2H, H18), 7.06 (td, *J* = 8.8, 2.1 Hz, 1H, H13), 6.82 (d, *J* = 8.6 Hz, 2H, H19), 4.70 (d, *J* = 13.4 Hz, 1H, H7), 3.99 (d, *J* = 13.6 Hz, 1H, H7), 3.76 (d, *J* = 9.2 Hz, 3H, H21), 3.67 (dt, *J* = 10.2, 4.9 Hz, 1H, H1), 3.36 – 3.15 (m, 2H, H7+H9), 2.88 (q, *J* = 12.2 Hz, 1H, H7), 2.69 – 2.54 (m, 2H, H2+H5), 2.45 (td, *J* = 14.9, 7.3 Hz, 1H, H5), 2.20 – 2.05 (m, 3H, H8+H3), 1.93 (dq, *J* = 25.3, 11.6 Hz, 2H, H8), 1.73 (m, 1H, H4), 1.57 – 1.50 (m, 1H, H2), 1.24 (s, 6H, H23), 1.23 (s, 6H, H23) ppm; **^{13}C NMR** (151 MHz, $CDCl_3$) δ = 171.3 (C6), 164.4 (d, *J* = 251.2 Hz, C14), 160.4 (C16), 157.7 (C20), 127.9 (C18), 122.3 (C12), 117.3 (C11), 113.8 (C19), 112.7 (d, *J* = 25.6 Hz, C13), 97.7 (d, *J* = 26.7 Hz, C15), 83.1 (C22), 55.4 (C21), 45.4 (C7), 41.5 (C7), 36.0 (C1), 34.6 (C9), 33.7 (C5), 31.8 (C2), 31.3 (C3), 30.7 (C8), 30.4 (C8), 29.6 (C4), 25.2 (C23), 24.9 (C23) ppm; **^{11}B NMR** (128 MHz, $CDCl_3$) δ = 31.36 ppm; **^{19}F NMR** (376 MHz, $CDCl_3$) δ = -109.18 (dtd, *J* = 27.1, 8.6, 4.9 Hz) ppm; **IR** (ATR): ṽ = 607, 621, 669, 729, 759, 847, 956, 1006, 1033, 1122, 1138, 1167, 1215, 1247, 1270, 1303, 1340, 1371, 1445, 1513, 1610, 1649, 2976 cm^{-1}; **HRMS** (ESI) $[M+Na]^+$ Calculated mass for $C_{32}H_{38}BFN_2O_5Na$:583.2750; Mass found:583.2753.

(5-(2-((5-(2, 5-Dimethylphenoxy)-2, 2-dimethylpentyl)oxy)-2-oxoethyl)-3-phenylbicyclo[2.1.0]pentan-1-yl)boronic acid, pinacol ester (46)

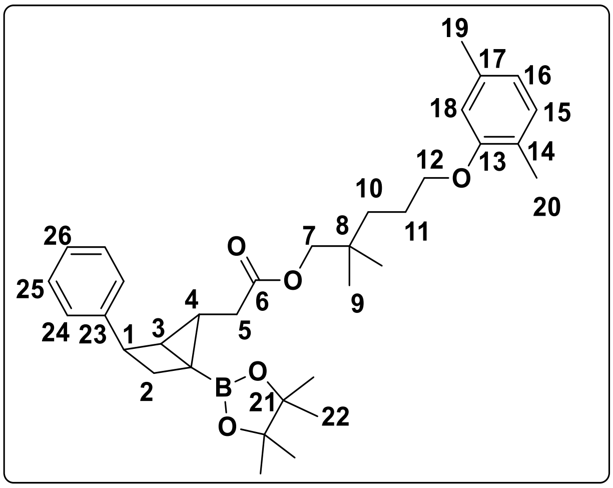

Prepared according to General Procedure **I**, **27** (168 mg, 0.25 mmol), in the presence of KO*t*Bu (0.5 mL, 2 equiv., 1 M in THF). was converted to **46** yielding a white solid as the single diastereomer (102 mg, 75%, d.r.=>95:5), after purification by flash column chromatography ($B(OH)_3$-SiO_2, 0 →2% EtOAc/*n*-hexane).

$\boldsymbol{R_f}$ (10% EtOAc/*n*-hexane) = 0.48; **^{1}H NMR** (400 MHz, $CDCl_3$) δ = 7.30 – 7.25 (m, 2H, H25), 7.21 – 7.17 (m, 2H, H24), 7.14 (t, *J* = 7.0 Hz, 1H, H26), 7.01 (d, *J* = 7.5 Hz, 1H, H15), 6.70 – 6.64 (m, 1H, H16), 6.61 (d, *J* = 1.7 Hz, 1H, H18), 3.90 (t, *J* = 6.4 Hz, 2H, H12), 3.88 – 3.81 (m, 2H, H7), 3.74 (dt, *J* = 10.2, 4.9 Hz, 1H, H1), 2.67 (t, *J* = 10.9 Hz, 1H, H2), 2.52 (dd, *J* = 7.4, 1.7 Hz, 2H, H5), 2.32 (s, 3H, H19), 2.18 (s, 3H, H20), 2.12 (m, 1H, H3), 1.82 – 1.71 (m, 2H, H11), 1.68 – 1.56 (m, 2H, H2+H4), 1.47 – 1.39 (m, 2H, H10), 1.25 (s, 6H), 1.24 (s, 6H), 0.94 (s, 6H, H9) ppm; **^{13}C NMR** (100 MHz, $CDCl_3$) δ = 173.4 (C6), 157.1 (C13), 143.9 (C23), 136.5 (C17), 130.4 (C15), 128.2 (C25), 126.7 (C24), 125.5 (C26), 123.7 (C14), 120.7 (C16), 112.1 (C18), 83.1 (C21), 72.4 (C7), 68.4 (C12), 36.4 (C1), 35.6 (C10), 34.4 (C5), 33.7 (C8), 31.5 (C2), 30.7 (C3), 29.1 (C4), 25.2 (C22), 24.7 (C22), 24.4 (C9), 24.4

(C9), 24.2 (C12), 21.5 (C19), 15.9 (C20) ppm; **^{11}B NMR** (128 MHz, $CDCl_3$) δ = 33.49 ppm; **IR** (ATR): ῦ = 669, 699, 752, 802, 855, 966, 1009, 1030, 1101, 1131, 1155, 1213, 1264, 1300, 1339, 1371, 1389, 1447, 1508, 1584, 1613, 735, 2375, 2973 cm^{-1}; **HRMS** (ESI) [M+Na]$^+$ Calculated mass for $C_{34}H_{47}BO_5Na$: 569.3409; Mass found: 569.3451.

(5-(2-(4-(2-((2, 4-Dimethylphenyl)thio)phenyl)piperazin-1-yl)-2-oxoethyl)-3-phenylbicyclo[2.1.0]pentan-1-yl)boronic acid, pinacol ester (47)

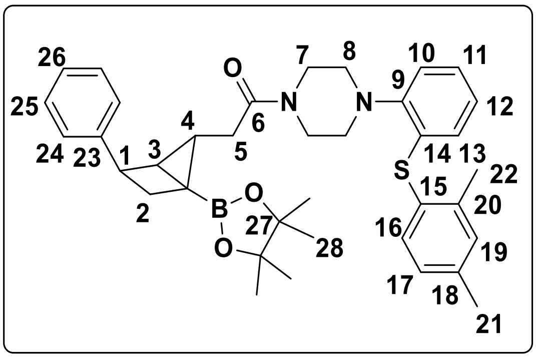

Prepared according to General Procedure **I**, **28** (118 mg, 0.16 mmol), in the presence of KO*t*Bu (0.24 mL, 1.5 equiv., 1 M in THF). was converted to **47** yielding a white solid as the single diastereomer (69 mg, 71%, d.r.=> 95:5), after purification by flash column chromatography ($B(OH)_3$-SiO_2, 5% → 20% EtOAc/*n*-hexane).

R$_f$ (30% EtOAc/*n*-hexane) = 0.45; **^{1}H NMR** (400 MHz, $CDCl_3$) δ = 7.39 (d, *J* = 7.8 Hz, 1H, H16), 7.33 – 7.27 (m, 2H, H25), 7.24 (d, *J* = 7.5 Hz, 2H, H24), 7.20 – 6.98 (m, 5H, H12+H13+H17+H19+H26), 6.92 (t, *J* = 7.6 Hz, 1H, H11), 6.57 (d, *J* = 7.9 Hz, 1H, H10), 3.85 (m, 2H, H7/8), 3.76 (dt, *J* = 10.1, 4.8 Hz, 1H, H1), 3.64 (m, 2H, H7/8), 3.14 – 2.94 (m, 4H, H7/8), 2.74 – 2.60 (m, 2H, H2+H5), 2.50 (dd, *J* = 16.0, 7.3 Hz, 1H, H5), 2.39 (s, 3H, H21), 2.35 (s, 3H, H22), 2.21 (m, 1H, H3), 1.73 (d, *J* = 7.4 Hz, 1H, H4), 1.66 (dd, *J* = 11.1, 5.0 Hz, 1H, H2), 1.28 (s, 12H, H28) ppm; **^{13}C NMR** (100 MHz, $CDCl_3$) δ = 171.3 (C6), 148.6 (C9), 144.1 (C23), 142.4 (C18), 139.4 (C15), 136.2 (C16), 134.7 (C14), 131.8 (C19), 128.2 (C25), 128.0 (C20), 127.8 (C12), 126.8 (C24), 126.5 (C10), 125.7 (C17), 125.5 (C26), 125.0 (C11), 120.2 (C13), 83.1 (C27), 52.0 (C7/8), 51.6 (C7/8), 46.0 (C7/8), 42.0 (C7/8), 36.5 (C1), 33.6 (C5), 31.6 (C2), 30.8 (C3), 29.6 (C4), 25.2 (C28), 24.8 (C28), 21.3 (C21), 20.7 (C22) ppm; **^{11}B NMR** (128 MHz, $CDCl_3$) δ = 32.65 ppm; **IR** (ATR): ῦ = 646, 670, 699, 726, 752, 815, 855, 908, 966, 1026, 1099, 1137, 1221, 1297, 1371, 1389, 1428, 1470, 1580, 2925, 2976 cm^{-1}; **HRMS** (ESI) [M+Na]$^+$ Calculated mass for $C_{37}H_{45}BN_2SO_3Na$: 631.3136; Mass found: 631.3174.

(5-(2-Ethoxy-2-oxoethyl)-3-(4-(4-((1-isopropoxy-2-methyl-1-oxopropan-2-yl)oxy)benzoyl)phenyl)bicyclo[2.1.0]pentan-1-yl)boronic acid, pinacol ester (48)

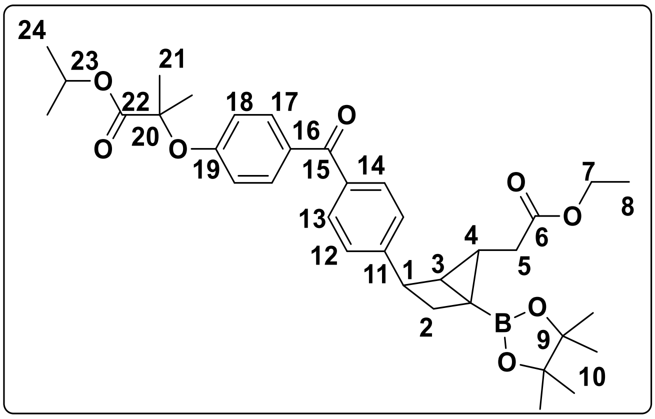

Prepared according to General Procedure **H**, **16** (175 mg, 0.24 mmol), in the presence of KO*t*Bu (0.36 mL, 1.5 equiv., 1 M in THF). was converted to **48** yielding a white solid as the single diastereomer (85 mg, 59%, d.r.=> 95:5), after purification by flash column chromatography ($B(OH)_3$-SiO_2, 0 → 15% EtOAc/*n*-hexane).

R$_f$ (15% EtOAc/*n*-hexane) = 0.20; **^{1}H NMR** (700 MHz, $CDCl_3$) δ = 7.74 (d, *J* = 8.8 Hz, 2H, H17), 7.67 (d, *J* = 8.3 Hz, 2H, H13), 7.29 (d, *J* = 7.6 Hz, 2H, H12), 6.84 (d, *J* = 8.8 Hz, 2H, H18), 5.08 (hept, *J* = 6.2 Hz, 1H, H23), 4.14 (q, *J* = 7.1 Hz, 2H, H7), 3.76 (dt, *J* = 10.3, 4.9 Hz, 1H, H1), 2.71 (t, *J* = 10.9 Hz, 1H, H2), 2.53 (dd, *J* = 17.0, 6.9 Hz, 1H, H5), 2.44 (dd, *J* = 17.0, 7.9 Hz, 1H, H5), 2.13 (m, 1H, H3), 1.65 (s, 6H, H21), 1.63 – 1.56 (m, 2H, H4+H2),

1.26 (t, J = 7.1 Hz, 3H, H8), 1.24 (s, 6H, H10), 1.23 (s, 6H, H10), 1.19 (d, J = 6.2 Hz, 6H, H24) ppm; **^{13}C NMR** (176 MHz, $CDCl_3$) δ = 195.5 (C15), 173.3 (C6), 173.2 (C22), 159.4 (C16), 148.7 (C14), 135.4 (C11), 132.1 (C17), 131.1 (C19), 129.9 (C13), 126.6 (C12), 117.2 (C18), 83.2 (C9), 79.42 (C20), 69.4 (C23), 60.4 (C7), 36.4 (C1), 34.3 (C5), 31.9 (C2), 30.3 (C3), 29.2 (C4), 25.5 (C21), 25.1 (C10), 24.7 (C10), 21.6 (C24), 14.5 (C8) ppm; **^{11}B NMR** (128 MHz, $CDCl_3$) δ = 33.28 ppm; **IR** (ATR): ῦ = 669, 690, 730, 766, 854, 927, 967, 1032, 1099, 1139, 1174, 1246, 1277, 1372, 1416, 1501, 1599, 1649, 1729, 2934, 2980 cm^{-1}; **HRMS** (ESI) $[M+Na]^+$ Calculated mass for $C_{35}H_{45}BO_8Na$: 627.3099; Mass found: 627.3129.

Control Reactions of Housane Formation

Reactions of the *anti*-Isomer

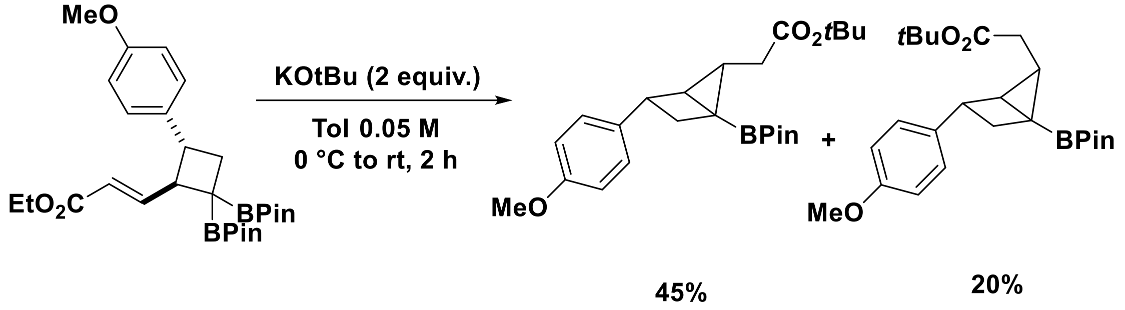

To an oven-dried microwave vial, **7-minor** (102 mg, 0.2 mmol, 1 equiv.) was dissolved in dry toluene (4 mL, 0.05 M) under an argon atmosphere. The reaction mixture was cooled to 0 °C before the dropwise addition of KO*t*Bu (0.4 mL, 2 equiv., 1 M in THF). Then the reaction mixture was allowed to warm to ambient temperature and stirred for another 2 h. After completion, the reaction was quenched by addition of H_2O (5 mL). The mixture was then extracted with Et_2O (3 × 10 mL). The combined organic phase was dried over Na_2SO_4, concentrated *in vacuo*, and purified by flash column chromatography ($B(OH)_3$-SiO_2, 0→2% EtOAc/*n*-hexane) afford **S31** (29 mg, 38%).

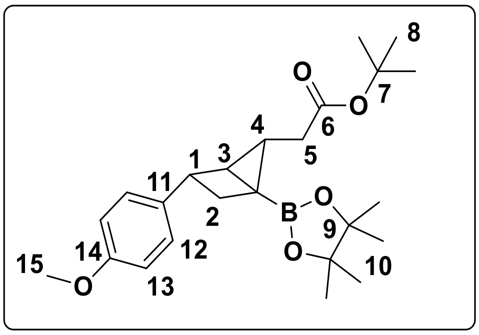

R$_f$ (10% EtOAc/*n*-hexane) = 0.42; **^{1}H NMR** (400 MHz, $CDCl_3$) δ = 7.29 – 7.24 (m, 2H, H12), 6.88 – 6.82 (m, 2H, H13), 3.80 (s, 3H, H15), 2.97 (dd, *J* = 6.2, 3.9 Hz, 1H, H1), 2.45 (dd, *J* = 17.0, 7.4 Hz, 1H, H5), 2.38 (dd, *J* = 17.0, 7.0 Hz, 1H, H5), 1.99 (dd, *J* = 11.4, 3.9 Hz, 1H, H2), 1.92 (ddd, *J* = 11.4, 6.2, 1.3 Hz, 1H, H2), 1.84 (td, *J* = 7.2, 1.7 Hz, 1H, H4), 1.77 (m, 1H, H3), 1.46 (s, 9H, H8), 1.24 (s, 12H, H10) ppm; **^{13}C NMR** (100 MHz, $CDCl_3$) δ = 172.8 (C6), 158.0 (C14), 139.0 (C11), 127.7 (C12), 113.9 (C13), 83.1 (C9), 80.1 (C7), 55.4 (C15), 39.8 (C1), 35.3 (C5), 34.6 (C2), 34.4 (C3), 32.8 (C4), 28.3 (C8), 25.2 (C10), 24.6 (C10) ppm; **^{11}B NMR** (128 MHz, $CDCl_3$) δ = 32.39 ppm; **IR** (ATR): ῦ = 669, 699, 732, 770, 828, 855, 959, 1035, 1081, 1134, 1213, 1243, 1299, 1339, 1368, 1389, 1513, 1583, 1612, 1731, 2375, 2930, 2976 cm^{-1}; **HRMS** (ESI) $[M+Na]^+$ Calculated mass for $C_{24}H_{35}BO_5Na$: 437.2470; Mass found: 437.2490.

Reactions of the Isomer Mixtures

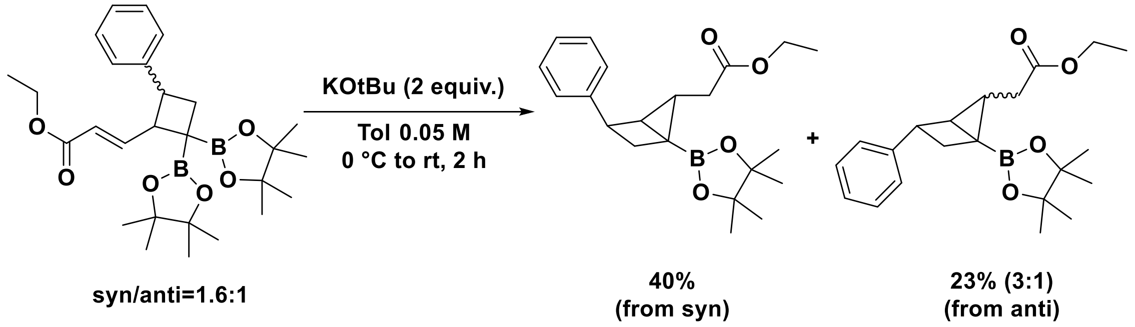

To an oven-dried microwave vial, a mixture of **5** (*syn/anti*=1.6:1, 96 mg, 0.2 mmol, 1 equiv.) was dissolved in dry toluene (4 mL, 0.05 M) under an argon atmosphere. The reaction mixture was cooled to 0 °C before the dropwise addition of KO*t*Bu (0.4 mL, 2 equiv., 1 M in THF). Then the

reaction mixture was allowed to warm to ambient temperature and stirred for another 2 h. After completion, the reaction was quenched by addition of H_2O (5 mL). The mixture was then extracted with Et_2O (3 × 10 mL). The combined organic phase was dried over Na_2SO_4, concentrated *in vacuo*. Crude NMR analysis against the internal standard (1,3,5-trimethoxybenzene) revealed that *syn*-isomer afforded a diastereoselective housane product, while the *anti*-isomer formed a mixture of diastereomers of housanes.

Reactions of *anti*-Isomer at Lower Temperature

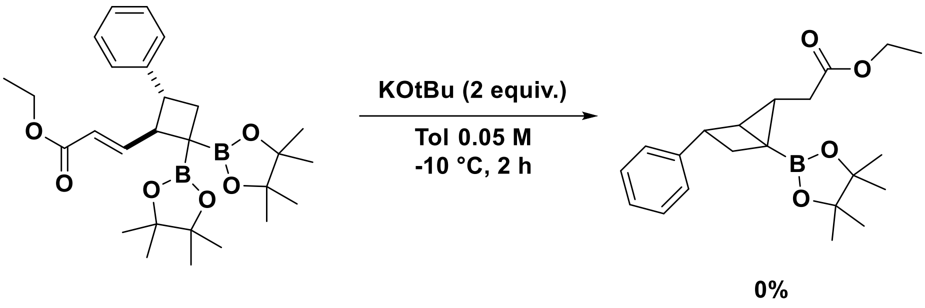

To an oven-dried microwave vial, **5** (96 mg, 0.2 mmol, 1 equiv.) was dissolved in dry toluene (4 mL, 0.05 M) under an argon atmosphere. The reaction mixture was cooled to -10 °C before the dropwise addition of KO*t*Bu (0.4 mL, 2 equiv., 1 M in THF). Then the reaction mixture was stirred for another 2 h. After completion, the reaction was quenched by addition of H_2O (5 mL). The mixture was then extracted with Et_2O (3 × 10 mL). The combined organic phase was dried over Na_2SO_4, concentrated *in vacuo*. Crude NMR analysis against the internal standard (1,3,5-trimethoxybenzene) revealed that desired housane product was not detected and instead protodeboronation had occurred. This indicates at lower temperature, the intramolecular Michael addition is inefficient and deborylation competes.

Derivatizations

8-(*p*-Tolyl)-3-oxa-2-boratricyclo[4.3.0.0^{1,7}]nonan-2-ol (49)

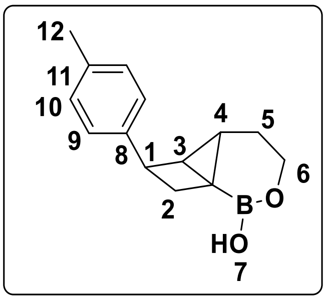

In an oven-dried flask, **30** (74 mg, 0.2 mmol) was dissolved in THF (4 mL, 0.05 M). $LiAlH_4$ (19 mg, 0.5 mmol, 2.5 equiv.) was added portion-wise over 5 minutes to the solution. The reaction mixture was stirred for 2 h at 0 °C. The resulting solution was acidified to pH 7 using 2 M of HCl solution and poured into water. The organic phase was extracted with DCM (3 × 20 mL) and washed with brine. The combined organic phase was dried over Na_2SO_4, filtered, and concentrated *in vacuo*. Purification by flash column chromatography (SiO_2, 0→10% EtOAc/*n*-hexane) to afford **49** as a white solid (19 mg, 42%).

R_f (10% EtOAc/*n*-hexane) = 0.15; **^{1}H NMR** (400 MHz, $CDCl_3$) δ = 7.10 (d, *J* = 8.0 Hz, 2H, H10), 7.04 (d, *J* = 8.2 Hz, 1H, H9), 4.44 (s, 1H, H7), 3.79 – 3.67 (m, 2H, H6), 3.43 (ddd, *J* = 13.3, 11.5, 2.9 Hz, 1H, H1), 2.69 – 2.60 (m, 2H, H2+H3), 2.32 (s, 3H, H12), 2.15 – 2.05 (m, 1H, H5), 1.88 (m, 1H, H5), 1.60 (m, 2H, H2+H4) ppm; **^{13}C NMR** (100 MHz, $CDCl_3$) δ = 140.8 (C8), 135.1 (C11), 128.9 (C10), 126.6 (C9), 60.2 (C6), 35.3 (C1), 29.0 (C2), 27.3 (C3), 26.6 (C4), 26.1 (C5), 21.2 (C12) ppm; **^{11}B NMR** (128 MHz, $CDCl_3$) δ = 30.60 ppm; **IR** (ATR): ῦ = 614, 671, 694, 717, 733, 759, 782, 802, 816, 839, 864, 880, 915, 938, 979, 1014, 1043, 1079, 1101, 1121, 1154, 1178, 1226, 1241, 1274, 1309, 1325, 1396, 1411, 1452, 1478, 1510, 2375, 2864, 2894, 2960, 3326 cm^{-1}; **HRMS** (ESI) $[M+Na]^+$ Calculated mass for $C_{14}H_{17}BO_2Na$: 251.1214; Mass found: 251.1265.

2-(1-Borono-3-vinylbicyclo[2.1.0]pentan-5-yl)acetate, pyridin-1-ium-2,3,4,5,6-*d*$_5$ salt (50)

To a round-bottom flask, **39** (30.6 mg, 0.1 mmol) and lithium hydroxide (7.2 mg, 0.3 mmol, 3 equiv.) were dissolved in THF/H_2O (2 mL, 0.05 M, 9/1). The reaction mixture was stirred for 16 h at ambient temperature. The resulting solution was maintained to pH 2 using 1 M HCl solution and poured into water. The organic phase was extracted with DCM (20 mL × 3) and washed with brine. The combined organic phase was dried over Na_2SO_4, filtered, and concentrated *in vacuo* without further purification to afford **50** as a colorless solid (36 mg, quant.).

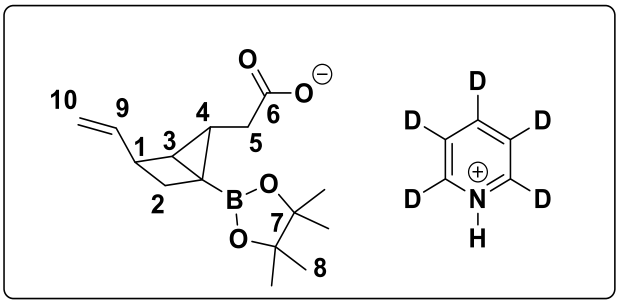

^{1}H NMR (400 MHz, Pyr) δ = 5.87 (ddd, *J* = 16.5, 10.4, 5.5 Hz, 1H, H9), 5.14 (dt, *J* = 17.3, 2.0 Hz, 1H, H10), 5.02 (dt, *J* = 10.4, 1.9 Hz, 1H, H10), 3.09 (dq, *J* = 10.7, 5.2 Hz, 1H, H1), 2.86 (d, *J* = 7.3 Hz, 2H, H5), 2.57 (t, *J* = 10.7 Hz, 1H, H2), 2.12 (td, *J* = 7.4, 1.9 Hz, 1H, H4), 2.07 (m, 1H, H3), 1.54 (dd, *J* = 11.0, 4.8 Hz, 1H, H2), 1.25 (s, 6H, H8), 1.24 (s, 6H, H8) ppm; **^{13}C NMR** (100 MHz, Pyr) δ = 176.0 (C6), 140.3 (C9), 114.7 (C10), 83.8 (C7), 36.6 (C1), 35.3 (C5), 31.7 (C3), 30.8 (C2), 29.6 (C4), 25.6 (C8), 25.2 (C8) ppm; **^{11}B NMR** (128 MHz, Pyr) δ = 33.27 ppm. **IR** (ATR): ῦ = 603, 637, 748, 823, 854, 920, 968, 991, 1037, 1104, 1215, 1229, 1302, 1347, 1372, 1390, 1416, 1513, 1612, 1644, 1703, 2326, 2347, 2930, 2979 cm^{-1}; **HRMS** (ESI) $[M+H+Na]^+$ Calculated mass for $C_{15}H_{23}BO_4Na$: 301.1582; Mass found: 301.1561.

Comment: The carboxylic acid obtained after hydrolysis is not stable for long-time characterization due to the intramolecular reaction between the proton and the strained sigma bond of housane core. Therefore, pyridine-*d*$_5$ was used as the solvent for NMR characterization.

Potassium, (5-(2-ethoxy-2-oxoethyl)-3-(*p*-tolyl)bicyclo[2.1.0]pentan-1-yl)trifluoroborate (51)

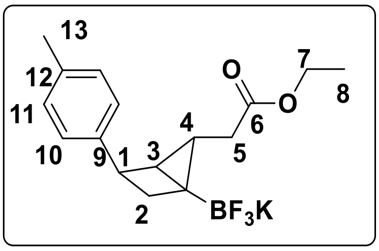

The reaction was performed according to a modified procedure of Masarwa *et al.*[18] In an open flask, **30** (370 mg, 1 mmol) was dissolved in acetonitrile/methanol (8 mL, 1/1, 0.125 M). To the above mixture, a solution of potassium fluoride (4 mmol, 232 mg in 0.6 mL H_2O) was added dropwise, and the mixture was stirred at ambient temperature for 5 minutes before the solution of *L*-(+)-tartaric acid (2.04 mmol, 308 mg in 2 mL THF) was added dropwise to the rapidly stirring clear solution. The reaction mixture was stirred for another 30 minutes, then the mixture was filtered to remove the white precipitate and washed thoroughly with excess acetonitrile (10 mL). The filtrate was concentrated and precipitated with pentane to afford **51** as a white solid (305 mg, 87%).

^{1}H NMR (400 MHz, Acetone-*d*6) δ = 7.08 (d, *J* = 7.9 Hz, 2H), 7.01 (d, *J* = 7.9 Hz, 2H), 4.09 (q, *J* = 7.1 Hz, 2H, H7), 3.47 (dt, *J* = 9.9, 4.6 Hz, 1H, H1), 2.55 (t, *J* = 10.4 Hz, 1H, H2), 2.45 (dd, *J* = 15.9, 6.3 Hz, 1H, H5), 2.29 – 2.19 (m, 4H, H13+H5), 1.42 M, 1H, H3), 1.24 – 1.18 (m, 4H, H8+H2), 1.06 – 1.00 (m, 1H, H4) ppm; **^{13}C NMR** (100 MHz, Acetone) δ = 175.2 (C6), 143.7 (C9), 134.4, 129.1, 127.5, 60.2 (C7), 36.7 (C1), 35.3 (C5), 33.6 (C2), 26.3 (C4), 25.1 (C3), 21.0 (C13), 14.6 (C8) ppm; **^{19}F NMR** (376 MHz, Acetone) δ = -139.54 ppm; **^{11}B NMR** (128 MHz, Acetone) δ = 4.39 ppm; **IR** (ATR): ῦ = 635, 697, 750, 796, 875, 974, 997, 1027, 1055, 1124, 1159, 1197, 1299, 1352, 1369, 1447, 1475, 1491, 1508, 1544, 1602, 1714, 2375, 2978 cm^{-1}; **HRMS** (ESI) $[M+K]^+$ Calculated mass for $C_{16}H_{19}BF_3O_2K_2$: 389.0699; Mass found: 389.0761.

Ethyl 2-(2-(*p*-tolyl)bicyclo[2.1.0]pentan-5-yl)acetate (52)

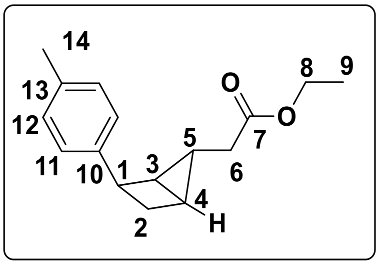

The reaction was performed according to a modified procedure of Qin *et al.*[19] To an oven-dried flask, **51** (70 mg, 0.2 mmol, 1.0 equiv.), *t*-butylcatechol (200 mg, 1.2 mmol, 6.0 equiv.) were added. The vial was sealed with a septum and purged with nitrogen before the addition of dry toluene (1 mL, 0.2 M). After stirring at 80 °C for 5 h, the reaction mixture was cooled to ambient temperature, filtered through celite, washed with hexane, and concentrated *in vacuo*. Purification by flash column chromatography (SiO_2, 0→2% EtOAc/*n*-hexane) to afford a mixture of **52** as a colorless oil (34 mg, 70%).

R_f (5% EtOAc/*n*-hexane) = 0.60; **^{1}H NMR** (400 MHz, $CDCl_3$) δ = 7.09 (m, 4H), 4.16 (q, *J* = 7.2 Hz, 2H, H8), 3.71 (dt, *J* = 9.9, 4.5 Hz, 1H, H1), 2.63 (td, *J* = 10.8, 4.5 Hz, 1H, H2), 2.32 (s, 3H, H14), 2.22 (dd, *J* = 15.8, 6.9 Hz, 1H, H6), 2.07 (dd, *J* = 15.9, 7.7 Hz, 1H, H6), 1.84 (td, *J* = 4.6, 2.3 Hz, 1H, H3), 1.62 – 1.53 (m, 2H, H2+H4), 1.28 (m, 4H, H9+H5) ppm; **^{13}C NMR** (100 MHz, $CDCl_3$) δ = 173.0 (C7), 140.5 (C10), 134.9 (C13), 128.8 (C12), 126.8 (C11), 60.5 (C8), 36.4 (C1), 36.2 (C6), 30.0 (C2), 23.6 (C3), 21.3 (C14), 21.1 (C5), 17.9 (C4), 14.4 (C9) ppm; **IR** (ATR): ῦ = 699, 775, 811, 839, 930, 1030, 1093, 1124, 1178, 1250, 1339, 1368, 1475, 1513, 1732, 2375, 2927, 2978 cm^{-1}; **HRMS** (EI) $[M]^+$ Calculated mass for $C_{16}H_{20}O_2$:244.1458; Mass found:244.1478.

Ethyl 2-(1-(1*H*-indazol-1-yl)-3-(*p*-tolyl)bicyclo[2.1.0]pentan-5-yl)acetate (53)

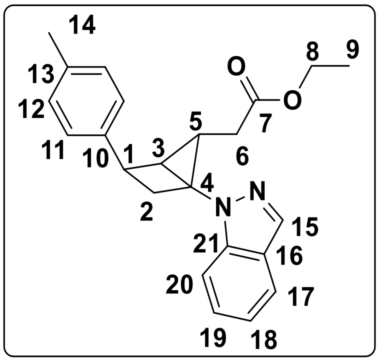

The reaction was performed according to a modified procedure of Londregan *et al.*[20] To an oven-dried 5 mL microwave vial, phenanthroline (43 mg, 0.24 mmol, 1.2 equiv.), $Cu(OAc)_2$ (44 mg, 0.24 mmol, 1.2 equiv.) and DCE (2 mL) were added and stirred at 60 °C for 20 minutes. Meanwhile in a separate oven-dried 5 mL vial, **51** (70 mg, 0.2 mmol), indazole (47 mg, 0.4 mmol, 2 equiv.), K_3PO_4 (0.6 mL, 1 M in H_2O, 0.6 mmol, 3.0 equiv.) were added. The vial was sealed with a septum and purged with nitrogen before the sequential addition of DCE (1 mL, 0.2 M) and the solution of copper catalyst. The reaction was heated to 80 °C for 4 h. After completion, the reaction mixture was cooled to ambient temperature and quenched by saturated aqueous NH_4Cl solution (2 mL). The organic phase was extracted with DCM (10 mL × 3) and washed with brine. The combined organic phase was dried over Na_2SO_4, filtered, and concentrated *in vacuo*. Purification by flash column chromatography (SiO_2, 0→10% EtOAc/*n*-hexane) to afford **53** as a white solid (40 mg, 56%).

R*f* (10% EtOAc/*n*-hexane) = 0.25; **^{1}H NMR** (400 MHz, $CDCl_3$) δ = 8.01 (s, 1H, H15), 7.74 (d, *J* = 8.1 Hz, 1H, H17), 7.59 (d, *J* = 8.5 Hz, 1H, H20), 7.41 (t, *J* = 7.7 Hz, 1H, H19), 7.22 – 7.11 (m, 5H, H18+H11+H12), 4.15 (dt, *J* = 10.1, 4.7 Hz, 1H, H1), 4.05 (q, *J* = 7.1 Hz, 2H, H8), 3.00 (t, *J* = 10.9 Hz, 1H, H2), 2.92 (dt, *J* = 5.0, 1.4 Hz, 1H, H3), 2.48 (dd, *J* = 16.8, 5.4 Hz, 1H, H6), 2.39 – 2.33 (m, 4H, H14+H2), 2.03 (ddd, *J* = 9.1, 5.4, 1.4 Hz, 1H, H5), 1.75 (dd, *J* = 16.8, 9.2 Hz, 1H, H6), 1.17 (t, *J* = 7.1 Hz, 3H, H9) ppm; **^{13}C NMR** (100 MHz, $CDCl_3$) δ = 172.3 (C7), 140.9 (C21), 138.1 (C10), 135.6 (C13), 134.3 (C15), 129.0 (C12), 127.3 (C11), 126.8 (C19), 124.5 (C16), 121.4 (C17/18), 121.3 (C17/18), 109.8 (C20), 60.6 (C8), 41.9 (C4), 36.2 (C2), 34.4 (C1), 32.8 (C6), 31.3 (C3), 26.1 (C5), 21.2 (C14), 14.3 (C9) ppm; **IR** (ATR): ṽ = 631, 648, 742, 768, 812, 908, 941, 1006, 1023, 1098, 1164, 1238, 1299, 1366, 1432, 1470, 1497, 1514, 1615, 1731, 2375, 2928, 2977 cm^{-1}; **HRMS** (ESI) $[M+Na]^+$ Calculated mass for $C_{23}H_{24}N_2O_2Na$:383.1730; Mass found:383.1768.

(5-(2-(Methylamino)-2-oxoethyl)-3-phenylbicyclo[2.1.0]pentan-1-yl)boronic acid (54)

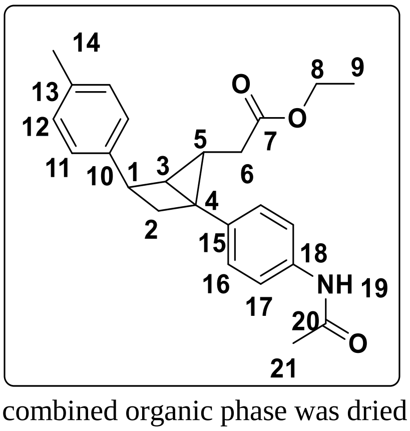

The reaction was performed according to a modified procedure of Londregan *et al.*[20] To an oven-dried 5 mL microwave vial, **51** (70 mg, 0.2 mmol), 4-bromoacetanilide (86 mg, 0.4 mmol, 2 equiv.), Pd(dppf)Cl_2 (14.6 mg, 10 mol %), and Cs_2CO_3 (195 mg, 0.6 mmol, 3 equiv.) were added. The vial was sealed with a septum and purged with nitrogen before the sequential addition of the deoxygenated Tol/H_2O (3 mL, 10/1, 0.067 M) via syringe. The reaction was then heated to 90 °C for 18 h. After completion, the reaction mixture was cooled to ambient temperature and quenched by saturated aqueous NH_4Cl solution (2 mL). The organic phase was extracted with DCM and washed with brine. The combined organic phase was dried over Na_2SO_4, filtered, and concentrated *in vacuo*. Purification by flash column chromatography (SiO_2, 0→50% EtOAc/*n*-hexane) to afford **54** as a white solid (55 mg, 73%).

R*f* (50% EtOAc/*n*-hexane) = 0.35; **^{1}H NMR** (400 MHz, $CDCl_3$) δ = 7.48 (s, 1H, H19), 7.44 (d, *J* = 8.1 Hz, 2H, H17), 7.13 (q, *J* = 5.5 Hz, 6H, H11+H12+H16), 4.06 (q, *J* = 7.1 Hz, 2H, H8), 3.82 (dt, *J* = 10.1, 4.5 Hz, 1H, H1), 2.71 (t, *J* = 10.7 Hz, 1H, H2), 2.31 (m, 4H, H14+H3), 2.18 – 2.08

(m, 5H, H21+H6+H2), 1.97 (dd, J = 16.5, 8.3 Hz, 1H, H6), 1.64 (t, J = 7.5 Hz, 1H, H5), 1.19 (t, J = 7.1 Hz, 3H, H9) ppm; **^{13}C NMR** (100 MHz, $CDCl_3$) δ = 172.9 (C7), 168.5 (C20), 140.0 (C10), 136.2 (C18), 135.6 (C15), 135.1 (C13), 128.9 (C12), 128.8 (C16), 127.0 (C11), 119.8 (C17), 60.5 (C8), 36.8 (C2), 35.0 (C1), 33.8 (C6), 31.0 (C4), 28.0 (C3), 27.1 (C5), 24.6 (C21), 21.2 (C14), 14.3 (C9) ppm; **IR** (ATR): ῡ = 638, 670, 706, 811, 832, 881, 980, 1010, 1030, 1144, 1167, 1246, 1339, 1371, 1442, 1513, 1609, 1638, 2377, 2977 cm^{-1}; **HRMS** (ESI) $[M+Na]^+$ Calculated mass for $C_{24}H_{27}NO_3Na$:400.1883; Mass found:400.1886.

Ethyl 2-(1-(hydroxymethyl)-3-(4-methoxyphenyl)bicyclo[2.1.0]pentan-5-yl)acetate (55)

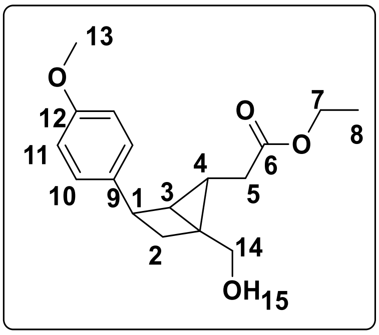

The reaction was performed according to a modified procedure of Qin *et al.*[19] To an oven-dried 5 mL microwave vial, **31** (77 mg, 0.2 mmol, 1.0 equiv.) and dibromomethane (87 mg, 0.5 mmol, 2.5 equiv.) were dissolved in dry THF (2 mL, 0.1 M) and cooled to –78 °C under an argon atmosphere. To this mixture, *n*BuLi (2.5 M in hexane, 112 µL, 0.28 mmol, 1.4 equiv.) was added dropwise before the solution was stirred for 10 minutes at –78 °C. The reaction was then warmed up to ambient temperature and stirred overnight. The reaction mixture was quenched with saturated NH_4Cl solution (5 mL) and the aqueous phase was extracted with ethyl acetate (20 mL × 3). The combined organic phase was dried over Na_2SO_4, filtered and the solvent removed *in vacuo*.

To a solution of the crude in THF (2 mL, 0.1 M) and H_2O (1 mL, KH_2PO_4-NaOH buffer pH 7) kept at 0 °C was added $NaBO_3•H_2O$ (100 mg, 1 mmol, 5 equiv.). The reaction was allowed to stir for 1 h at 0 °C, before being allowed to reach ambient temperature. It was stirred for an additional 16 h before the reaction was quenched by saturated aqueous $Na_2S_2O_3$ solution (4 mL). The aqueous phase was extracted with EtOAc (20 mL × 3). The combined organic phase was dried over Na_2SO_4, filtered and the solvent removed *in vacuo*. Purification by flash column chromatography (SiO_2, 0→30% EtOAc/*n*-hexane) to afford **55** as a colorless oil (42 mg, 73%).

R_f (25% EtOAc/*n*-hexane) = 0.30; **^{1}H NMR** (400 MHz, $CDCl_3$) δ = 7.06 (d, J = 8.8 Hz, 2H, H10), 6.82 (d, J = 8.8 Hz, 2H, H11), 4.15 (q, J = 7.1 Hz, 2H, H7), 4.00 – 3.91 (m, 1H, H15), 3.78 (s, 3H, H13), 3.66 (m, 2H, H15+H1), 2.90 (d, J = 8.0 Hz, 1H, H16), 2.70 – 2.57 (m, 2H, H2+H5), 2.00 (dd, J = 16.6, 10.3 Hz, 1H, H5), 1.87 – 1.78 (m, 2H, H2+H3), 1.33 (ddd, J = 10.3, 4.9, 1.1 Hz, 1H, H4), 1.25 (t, J = 7.2 Hz, 3H, H8) ppm; **^{13}C NMR** (100 MHz, $CDCl_3$) δ = 174.3 (C6), 157.7 (C12), 135.2 (C9), 127.8 (C10), 113.6 (C11), 62.6 (C15), 61.1 (C7), 55.4 (C13), 34.4 (C1), 33.2 (C2), 33.0 (C5), 29.0 (C14), 27.0 (C3), 24.5 (C4), 14.2 (C8) ppm; **IR** (ATR): ῡ = 669, 687, 747, 803, 818, 872, 904, 950, 967, 1006, 1020, 1032, 1063, 1082, 1108, 1172, 1188, 1215, 1243, 1296, 1319, 1371, 1414, 1437, 1457, 1511, 1580, 1610, 1725, 2833, 2912, 3567 cm^{-1}; **HRMS** (ESI) $[M+Na]^+$ Calculated mass for $C_{17}H_{22}O_4Na$:313.1410; Mass found:313.1430.

5-(2-Ethoxy-2-oxoethyl)-3-(4-methoxyphenyl)bicyclo[2.1.0]pentane-1-carboxylic acid (56)

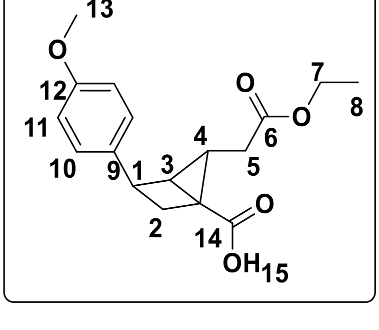

The reaction was performed according to a modified procedure of Stephenson *et al.*[21] To a solution of **55** (29 mg, 0.1 mmol, 1.0 equiv.) in CH_3CN/DCM (1 mL, 1/1, 0.1 M) was slowly added a solution of $NaIO_4$ (4 equiv.) and $RuCl_3$ hydrate (0.05 equiv.) in 0.5 mL H_2O at 0 °C. The mixture was stirred at ambient temperature for 1 h. After completion, the suspension was filtered through a pad of Celite and rinsed with EtOAc. The filtrates were dried over Na_2SO_4 and concentrated

in vacuo. Purification by flash column chromatography (SiO_2, 0→50% EtOAc/*n*-hexane) to afford **56** as a white solid (17 mg, 56%).

***R**$_f$* (50% EtOAc/*n*-hexane) = 0.35; **^{1}H NMR** (400 MHz, $CDCl_3$) δ = 7.10 (d, *J* = 8.3 Hz, 2H, H10), 6.85 (d, *J* = 8.7 Hz, 2H, H11), 4.15 (q, *J* = 7.2 Hz, 2H, H7), 3.79 (m, 4H, H1+H13), 3.00 (t, *J* = 11.0 Hz, 1H, H2), 2.82 – 2.68 (m, 2H, H5), 2.62 (dd, *J* = 4.9, 2.7 Hz, 1H, H3), 1.99 (td, *J* = 7.5, 2.7 Hz, 1H, H4), 1.86 (dd, *J* = 11.4, 4.8 Hz, 1H, H2), 1.25 (t, *J* = 7.1 Hz, 3H, H8) ppm; **^{13}C NMR** (100 MHz, $CDCl_3$) δ = 179.0 (C15), 172.4 (C6), 158.0 (C12), 133.8 (C9), 127.9 (C10), 113.8 (C11), 60.7 (C7), 55.4 (C13), 36.9 (C3), 34.5 (C1), 32.5 (C5), 31.8 (C2), 31.8 (C4), 26.3 (C14), 14.3 (C8) ppm; **IR** (ATR): ῦ = 6698699, 735, 772, 830, 959, 1033, 1081, 1110, 1213, 1243, 1299, 1339, 1368, 1389, 1513, 1583, 1612, 1731, 2930, 3568 cm^{-1}; **HRMS** (ESI) [M+Na]$^+$ Calculated mass for $C_{17}H_{20}O_5Na$: 327.1203; Mass found: 327.1193.

Ethyl 2-(2-oxo-4-(*p*-tolyl)cyclopentyl)acetate (57)

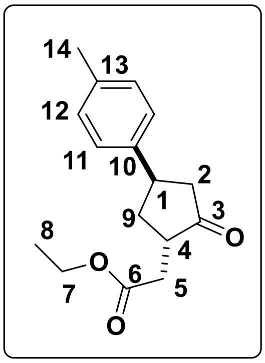

To a solution of **30** (74 mg, 0.2 mmol, 1 equiv.) in THF (2 mL) and H_2O (1 mL, KH_2PO_4-NaOH buffer pH 7) kept at 0 °C was added $NaBO_3$•H_2O (100 mg, 1 mmol, 5 equiv.). The reaction was allowed to stir for 1 h at 0 °C, before being allowed to reach ambient temperature. It was stirred for an additional 16 h before the reaction was quenched by saturated aqueous $Na_2S_2O_3$ solution (4 mL). The aqueous phase was extracted with EtOAc (20 mL × 3). The combined organic phase was dried over Na_2SO_4, filtered and the solvent removed *in vacuo*. Purification by flash column chromatography (SiO_2, 0→5% EtOAc/*n*-hexane) to afford **57** as a colorless oil (48 mg, 93%)

***R**$_f$* (5% EtOAc/*n*-hexane) = 0.55; **^{1}H NMR** (400 MHz, $CDCl_3$) δ = 7.16 – 7.09 (m, 4H, H10+H11), 4.14 (q, *J* = 7.1 Hz, 2H, H7), 3.57 (p, *J* = 6.5 Hz, 1H, H1), 2.80 – 2.50 (m, 5H, H4+H5+H2), 2.33 (s, 4H, H13+H9), 2.26 – 2.16 (m, 1H, H9), 1.26 (t, *J* = 7.1 Hz, 3H, H8) ppm; **^{13}C NMR** (100 MHz, $CDCl_3$) δ = 219.0 (C3), 172.0 (C6), 140.9 (C10), 136.3 (C13), 129.5 (C12), 126.7 (C11), 60.9 (C7), 45.0 (C2), 43.0 (C4), 38.1, 36.6 (C9), 34.7 (C5), 21.1 (C13), 14.3 (C8) ppm; **IR** (ATR): ῦ = 719, 816, 940, 1030, 1177, 1238, 1350, 1373, 1490, 1516, 1729, 2375, 2978 cm^{-1}; **HRMS** (EI) [M]$^+$ Calculated mass for $C_{16}H_{20}O_3$:260.1407; Mass found:260.1399.

(3-(2-Boronoethyl)-5-(2-ethoxy-2-oxoethyl)bicyclo[2.1.0]pentan-1-yl)boronic acid, pinacol ester (58)

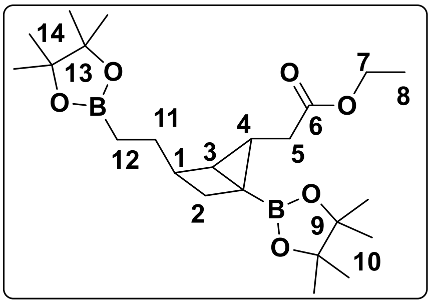

The reaction was performed according to a modified procedure of Morken *et al.*[22] To an oven-dried 5 mL microwave vial, $[Ir(cod)Cl]_2$ (0.7 mg, 1 mol%), 1, 1-bis(diphenylphosphino)methane (0.8 mg, dppm, 2 mol%) and **39** (30.6 mg, 0.1 mmol, 1 equiv.), were added. The vial was sealed with a septum and purged with nitrogen before the sequential addition of dry DCM (0.15 mL, 0.67 M) and pinacolborane (15 µL, 0.1 mml, 1 equiv.). The reaction was allowed to stir at ambient temperature for 18 h. Upon completion, the reaction was concentrated *in vacuo*. Purification by flash column chromatography ($B(OH)_3$-SiO_2, 0→5% EtOAc/n-hexane) to afford **58** as a white solid (40 mg, 92%).

***R**$_f$* (10% EtOAc/*n*-hexane) = 0.30; **^{1}H NMR** (400 MHz, $CDCl_3$) δ = 4.12 (q, *J* = 7.1 Hz, 2H, H7), 2.46 – 2.33 (m, 2H, H5), 2.28 (dtd, *J* = 12.4, 8.0, 4.7 Hz, 1H, H1), 2.15 (t, *J* = 10.5 Hz, 1H, H2), 1.75 – 1.68 (m, 2H, H3+H4), 1.40 – 1.27 (m, 2H, H11), 1.27 – 1.23 (m, 3H, H8), 1.22 (s, 12H,

H10/14), 1.20 (s, 6H, H10/14), 1.18 (s, 6H, H10/14), 1.06 (ddd, *J* = 11.0, 4.8, 1.1 Hz, 1H, H2), 0.77 – 0.51 (m, 2H, H12) ppm; **^{13}C NMR** (100 MHz, $CDCl_3$) δ = 173.6 (C6), 83.0 (C9/13), 82.9 (C9/13), 60.2 (C7), 35.4 (C1), 34.5 (C5), 31.2 (C3), 29.7 (C2), 27.6 (C4), 26.2 (C11), 25.1 (C10/14), 24.98 (C10/14), 24.95 (C10/14), 24.7 (C10/14), 14.4 (C8) ppm; **^{11}B NMR** (128 MHz, $CDCl_3$) δ = 34.29 ppm; **IR** (ATR): ῦ = 670, 733, 851, 966, 1032, 1129, 1144, 1213, 1234, 1270, 1297, 1320, 1371, 1448, 1508, 1737, 2375, 2927, 2977 cm^{-1}; **HRMS** (ESI) $[M+Na]^+$ Calculated mass for $C_{23}H_{40}B_2O_6Na$:457.2903; Mass found:457.2958.

(*E*)-(5-(2-Ethoxy-2-oxoethyl)-3-(3, 3, 4, 4, 5, 5, 6, 6, 6-nonafluorohex-1-en-1-yl)bicyclo[2.1.0]pentan-1-yl)boronic acid, pinacol ester (59)

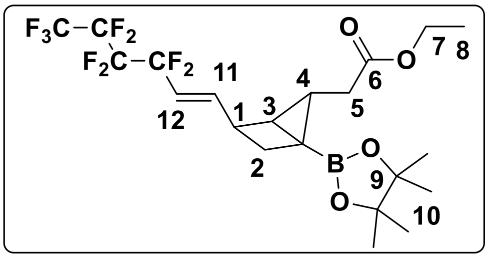

The reaction was performed according to a modified procedure of Zhou *et al.*[23] To an oven-dried 5 mL microwave vial, **39** (30.6 mg, 0.1 mmol, 1 equiv.), nonafluoro iodobutane (52 mg, 0.15 mmol, 1.5 equiv.) were added. The vial was sealed with a septum and purged with nitrogen before the sequential addition of DBU (23 mg, 0.15 mmol, 1.5 equiv.) and deoxygenated MeCN (0.3 mL, 0.33 M). The reaction mixture was stirred at ambient temperature under the irradiation of 40 W 390 nm purple LED for 18 h. Upon completion, the mixture was concentrated *in vacuo*. Purification by flash column chromatography ($B(OH)_3$-SiO_2, 0→1% EtOAc/*n*-hexane) to afford **59** as a yellow oil (35 mg, 70%, *E/Z*=8:1).

R_f (10% EtOAc/*n*-hexane) = 0.60; **^{1}H NMR** (400 MHz, $CDCl_3$) δ = 6.30 (ddt, *J* = 15.8, 4.8, 2.3 Hz, 1H, H11), 5.77 – 5.58 (m, 1H, H12), 4.14 (q, *J* = 7.1 Hz, 2H, H7), 3.25 – 3.09 (m, 1H, H1), 2.49 (dd, *J* = 17.1, 7.0 Hz, 1H, H5), 2.43 – 2.34 (m, 2H, H5+H2), 1.86 (dt, *J* = 4.5, 1.5 Hz, 1H, H3), 1.63 (ddd, *J* = 8.4, 7.0, 1.8 Hz, 1H, H4), 1.38 (ddd, *J* = 11.4, 4.9, 1.3 Hz, 1H, H2), 1.26 (t, *J* = 7.2 Hz, 3H, H8), 1.22 (s, 6H, H10), 1.21 (s, 6H, H10) ppm; **^{13}C NMR** (100 MHz, $CDCl_3$) δ = 172.9 (C6), 143.3 (t, *J* = 8.7 Hz, C11), 116.6 (t, *J* = 23.0 Hz, C12), 83.2 (C9), 60.3 (C7), 34.0 (C1), 34.0 (C5), 29.8 (C2), 29.2 (C3), 28.1 (C4), 24.9 (C10), 24.6 (C10), 14.2 (C8) ppm; **^{11}B NMR** (128 MHz, $CDCl_3$) δ = 32.84 ppm; **^{19}F NMR** (376 MHz, $CDCl_3$) δ = -81.02 – -81.12 (m), -111.38 (q, *J* = 13.1 Hz), -124.11 – -124.42 (m), -125.74 (td, *J* = 11.4, 3.3 Hz) ppm; **IR** (ATR): ῦ = 631, 669, 740, 768, 812, 854, 881, 910, 940, 966, 1029, 1098, 1131, 1165, 1184, 1231, 1302, 1371, 1391, 1431, 1470, 1497, 1516, 1613, 1735, 2375, 2933, 2980 cm^{-1}; **HRMS** (ESI) $[M+Na]^+$ Calculated mass for $C_{21}H_{26}BF_9O_4Na$:547.1672; Mass found:547.1723.

(2-(2-Ethoxy-2-oxoethyl)-1, 3-diiodo-4-phenylcyclopentyl)boronic acid, pinacol ester (61)

To an oven-dried 5 mL microwave vial, **29** (71 mg, 0.2 mmol, 1.0 equiv.) was dissolved in dry DCM (2 mL, 0.1 M) and cooled to 0 °C. A solution of iodine in dry DCM (1 mL, 0.3 M, 0.3 mmol, 1.5 equiv.) was added dropwise and the solution was stirred for 4 h at 0 °C, The reaction mixture was quenched with saturated $Na_2S_2O_3$ solution (2 mL) and the aqueous phase was extracted with DCM (10 mL × 3). The combined organic phase was dried over Na_2SO_4, filtered and the solvent removed *in vacuo*. Purification by flash column chromatography ($B(OH)_3$-SiO_2, 0→1% EtOAc/*n*-hexane) to afford **61** as a yellow oil (113 mg, 93%, d.r.=4.5:1).

R $_f$ (5% EtOAc/*n*-hexane) = 0.60; **^{1}H NMR** (600 MHz, $CDCl_3$) δ = 7.36 – 7.31 (m, 4H, H11+H12), 7.26 (m, 1H, H13), 4.61 (dd, *J* = 7.9, 5.6 Hz, 1H, H5), 4.17 (m, H8), 3.79 – 3.72 (m, 1H, H1), 3.14 (dd, *J* = 18.1, 3.1 Hz, 1H, H6), 3.05 – 2.96 (m, 2H, H2+H6), 2.47 – 2.39 (m, 2H, H2+H4), 1.33 (s, 6H, H15), 1.32 (s, 6H, H15), 1.28 (t, *J* = 7.1 Hz, 3H, H9) ppm; **^{13}C NMR** (151 MHz, $CDCl_3$) δ = 172.6 (C7), 142.4 (C10), 128.9 (C11), 127.2 (C13), 127.1 (C12), 84.4 (C14), 60.8 (C8), 58.9 (C1), 54.1 (C4), 50.0 (C2), 40.5 (C5), 39.3 (C6), 24.7 (C15), 24.6 (C15), 14.4 (C9) ppm; **^{11}B NMR** (192 MHz, $CDCl_3$) δ = 31.32 ppm; **IR** (ATR): ῡ = 608, 669, 696, 724, 752, 847, 911, 947, 1063, 1096, 1134, 1168, 1193, 1211, 1251, 1303, 1343, 1372, 1389, 1444, 1495, 1600, 1702, 1757, 2931, 2977 cm^{-1}; **HRMS** (ESI) $[M+Na]^+$ Calculated mass for $C_{21}H_{29}BI_2O_4Na$: 633.0140; Mass found: 633.0117.

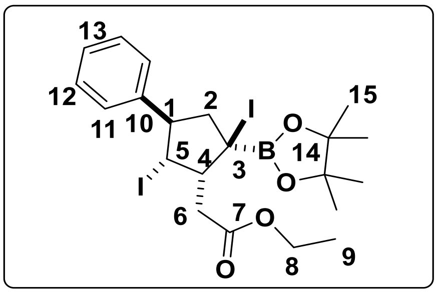

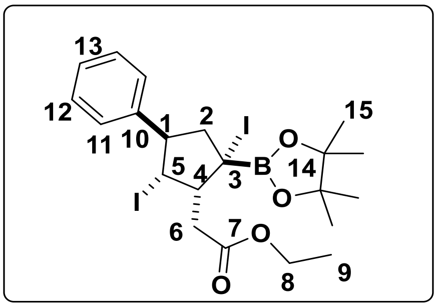

R $_f$ (5% EtOAc/*n*-hexane) = 0.58; **^{1}H NMR** (600 MHz, $CDCl_3$) δ = 7.35 – 7.32 (m, 2H, H12), 7.29 – 7.26 (m, 1H, H13), 7.16 – 7.13 (m, 2H, H11), 4.45 (t, *J* = 8.0 Hz, 1H, H5), 4.24 – 4.15 (m, 2H, H8), 3.45 (td, *J* = 8.2, 4.2 Hz, 1H, H1), 3.03 – 2.94 (m, 2H, H2+H6), 2.87 (ddd, *J* = 11.5, 8.3, 5.8 Hz, 1H, H4), 2.80 (t, *J* = 11.6 Hz, 1H, H6), 2.62 (dd, *J* = 16.1, 8.3 Hz, 1H, H2), 1.31 (t, *J* = 7.1 Hz, 3H, H9), 1.26 (s, 12H, H15) ppm; **^{13}C NMR** (151 MHz, $CDCl_3$) δ = 172.3 (C7), 144.1 (C10), 128.6 (C11), 128.2 (C12), 127.4 (C13), 84.6 (C14), 60.9 (C8), 60.8 (C1), 49.3 (C4), 46.9 (C6), 36.8 (C5), 35.7 (C2), 24.5 (C15), 24.4 (C15), 14.4 (C9) ppm; **^{11}B NMR** (192 MHz, $CDCl_3$) δ = 31.72 ppm; **IR** (ATR): ῡ = 669, 696, 729, 752, 847, 910, 947, 1063, 1096, 1134, 1168, 1193, 1211, 1251, 1343, 1372, 1444, 1495, 1600, 1702, 1758, 2931, 2977 cm^{-1}; **HRMS** (ESI) $[M+Na]^+$ Calculated mass for $C_{21}H_{29}BI_2O_4Na$: 633.0140; Mass found: 633.0110.

(6-(4-Methoxyphenyl)-2-oxohexahydro-2*H*-cyclopenta[b]furan-4-yl)boronic acid, pinacol ester (62)

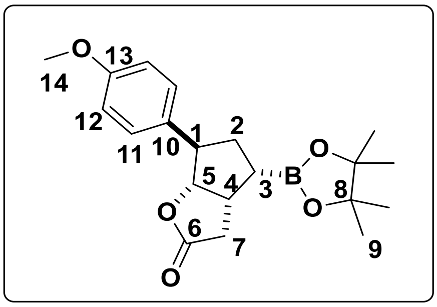

To an oven-dried 5 mL microwave vial, **42** (83 mg, 0.2 mmol, 1.0 equiv.) was dissolved in dry DCM (2 mL, 0.1 M) and cooled to -10 °C. TFA (153 μL, 2 mmol, 10 equiv.) was added dropwise and the solution was warmed up slowly to 0 °C. The reaction mixture was stirred for another 2 h before quenched with saturated $NaHCO_3$ solution (2 mL). The aqueous phase was extracted with DCM (10 mL × 3). The combined organic phase was dried over Na_2SO_4, filtered and the solvent removed *in vacuo*. Purification by flash column chromatography ($B(OH)_3$-SiO_2, 0→20% EtOAc/*n*-hexane) to afford **62** as a light yellow solid (34 mg, 47%).

R $_f$ (25% EtOAc/*n*-hexane) = 0.45; **^{1}H NMR** (400 MHz, $CDCl_3$) δ = 7.12 – 7.05 (m, 2H, H11), 6.88 – 6.82 (m, 2H, H12), 5.03 (dd, *J* = 7.3, 2.4 Hz, 1H, H5), 3.79 (s, 3H, H14), 3.43 (td, *J* = 5.8, 2.4 Hz, 1H, H1), 3.24 – 3.15 (m, 1H, H4), 2.75 (dd, *J* = 18.7, 10.3 Hz, 1H, H7), 2.62 (dd, *J* = 18.7, 4.6 Hz, 1H, H7), 2.25 – 2.14 (m, 1H, H2), 1.89 (ddd, *J* = 13.0, 6.9, 4.4 Hz, 1H, H2), 1.80 (ddd, *J* = 10.4, 8.7, 6.8 Hz, 1H, H3), 1.24 (s, 12H, H9) ppm; **^{13}C NMR** (100 MHz, $CDCl_3$) δ = 177.9 (C6), 158.3 (C13), 134.1 (C10), 128.2 (C11), 114.1 (C12), 92.0 (C5), 83.8 (C8), 55.4 (C14), 50.4 (C1), 40.4 (C4), 33.6 (C7), 33.3 (C2), 25.0 (C9), 24.9 (C9) ppm; **^{11}B NMR** (128 MHz, $CDCl_3$) δ = 33.48 ppm; **IR** (ATR): ῡ = 775, 828, 947, 987, 1036, 1141, 1243, 1293, 1339, 1366, 1391, 1454, 1511, 1583, 1610, 1727, 2838, 2928, 2977 cm^{-1}; **HRMS** (ESI) $[M+Na]^+$ Calculated mass for $C_{20}H_{27}BO_5Na$: 381.1844; Mass found: 381.1851.

Ethyl 2-(4-phenylcyclopent-1-en-1-yl)acetate (S31)

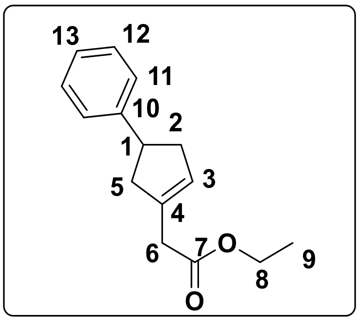

To an oven-dried 5 mL microwave vial, **31** (71 mg, 0.2 mmol, 1.0 equiv.) was dissolved in dry DCM (2 mL, 0.1 M) and cooled to 0 °C under an argon atmosphere. TFA (153 µL, 2 mmol, 10 equiv.) was added dropwise and the solution was stirred for 1 h at 0 °C. The reaction mixture was quenched with saturated $NaHCO_3$ solution (2 mL) and the aqueous phase was extracted with DCM (10 mL × 3). The combined organic phase was dried over Na_2SO_4, filtered and the solvent removed *in vacuo*. Purification by flash column chromatography (SiO_2, 0→10% EtOAc/*n*-hexane) to afford **S31** as a colorless oil (36 mg, 78%).

R_f (5% EtOAc/*n*-hexane) = 0.60; **^{1}H NMR** (400 MHz, $CDCl_3$) δ = 7.31 – 7.23 (m, 4H, H11+H12), 7.20 – 7.15 (m, 1H, H13), 5.61 – 5.57 (m, *J* = 1.4 Hz, 1H, H3), 4.16 (q, *J* = 7.1 Hz, 2H, H8), 3.54 (tt, *J* = 9.0, 6.9 Hz, 1H, H1), 3.16 (s, 2H, H6), 2.88 – 2.74 (m, 2H, H2+H5), 2.55 – 2.42 (m, 2H, H2+H5), 1.27 (t, *J* = 7.2 Hz, 3H, H9) ppm; **^{13}C NMR** (100 MHz, $CDCl_3$) δ = 171.4 (C7), 147.4 (C10), 135.9 (C4), 128.5 (C12), 127.4 (C3), 127.0 (C11), 126.0 (C13), 60.8 (C8), 43.8 (C1), 43.7 (C5), 41.5 (C2), 37.2 (C6), 14.4 (C9) ppm; **IR** (ATR): ῦ = 669, 699, 758, 812, 984, 1030, 1141, 1246, 1297, 1339, 1368, 1491, 1508, 1603, 1655, 1731, 2375, 2848, 2924 cm^{-1}; **HRMS** (EI) $[M]^+$ Calculated mass for $C_{15}H_{18}O_2$:230.1301; Mass found: 230.1311.

(2-(2-Ethoxy-2-oxoethyl)-4-phenylcyclopent-1-en-1-yl)boronic acid, pinacol ester (S32)

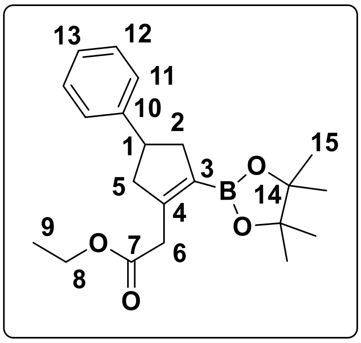

To an oven-dried 5 mL microwave vial, **31** (71 mg, 0.2 mmol, 1.0 equiv.) was dissolved in dry THF (2 mL, 0.1 M) and cooled to 0 °C under an argon atmosphere. A solution of *N*-bromosuccinimide in dry THF (1 mL, 0.2 M, 0.2 mmol, 1 equiv.) was added dropwise and the solution was stirred for 1 h at 0 °C. The reaction was then warmed up to ambient temperature and stirred overnight. Purification by flash column chromatography ($B(OH)_3$-SiO_2, 0→1% EtOAc/*n*-hexane) to afford **S32** as a light yellow oil (33 mg, 47%).

R_f (5% EtOAc/*n*-hexane) = 0.60; **^{1}H NMR** (600 MHz, $CDCl_3$) δ = 7.28 – 7.23 (m, 4H), 7.16 (tt, *J* = 6.2, 2.0 Hz, 1H, H13), 4.14 (q, *J* = 7.1 Hz, 2H, H8), 3.59 (d, *J* = 4.1 Hz, 2H, H6), 3.52 – 3.45 (m, 1H, H1), 2.99 (dd, *J* = 16.3, 8.8 Hz, 1H, H2), 2.91 (dd, *J* = 16.6, 8.2 Hz, 1H, H5), 2.68 – 2.56 (m, 2H, H5+H2), 1.29 – 1.23 (m, 15H, H15+H9) ppm; **^{13}C NMR** (151 MHz, $CDCl_3$) δ = 171.6 (C7), 151.9 (C4), 146.9 (C10), 128.4 (C12), 127.1 (C11), 125.8 (C13), 83.1 (C14), 60.6 (C8), 46.3 (C5), 44.2 (C2), 43.9 (C1), 37.0 (C6), 25.0 (C15), 14.4 (C9) ppm; **^{11}B NMR** (128 MHz, $CDCl_3$) δ = 29.42 ppm; **IR** (ATR): ῦ = 674, 699, 758, 819, 854, 981, 1030, 1066, 1147, 1204, 1271, 1309, 1412, 1457, 1602, 1731, 2934, 2978 cm^{-1}; **HRMS** (ESI) $[M+Na]^+$ Calculated mass for $C_{21}H_{29}BO_4Na$:379.2051; Mass found:379.2051.

X-ray Analysis

X-ray diffraction: Data sets for compounds **35** and **54** were collected with a Bruker D8 Venture Photon III Diffractometer. Programs used: data collection: *APEX4* Version 2021.4-0 [24] (Bruker AXS Inc., **2021**); cell refinement: *SAINT* Version 8.40B (Bruker AXS Inc., **2021**); data reduction: *SAINT* Version 8.40B (Bruker AXS Inc., **2021**); absorption correction, *SADABS* Version 2016/2 (Bruker AXS Inc., **2021**); structure solution *SHELXT*-Version 2018-3 [25] (Sheldrick, G. M. *Acta Cryst.,* **2015**, *A71*, 3-8); structure refinement *SHELXL*- Version 2018-3 [26] (Sheldrick, G. M. *Acta Cryst.*, **2015**, *C71* (1), 3-8) and graphics, *XP* [27] (Version 5.1, Bruker AXS Inc., Madison, Wisconsin, USA, **1998**). *R*-values are given for observed reflections, and wR^2 values are given for all reflections.

Exceptions and special features: For compound **35** the Bpin group and the ethoxy carbonyl group and for compound **54** two ethoxy carbonyl groups are disordered over two positions. Several restraints (SADI, SAME, ISOR, SIMU and EADP) were used in order to improve refinement stability.

X-ray crystal structure analysis of 35 (dan10754): A colourless, prism shaped specimen of $C_{21}H_{28}BBrO_4$, approximate dimensions 0.056 × 0.081 × 0.091 mm^3, was used for the X-ray crystallographic analysis. The crystals were crystallised from pentane and diethyl ether. The X-ray intensity data of **35** were measured on a Bruker D8 VENTURE KAPPA diffractometer system equipped with a microfocus sealed tube (λ = 0.71073 Å) and a multilayer mirror monochromator. The specimen was held at 100(2) K during the measurement with an Oxford Cryostream 1000 low temperature device. A total of 1333 frames were collected. The total exposure time was 4.96 hours. The frames were integrated with the SAINT V8.41 package using a narrow-frame algorithm. The integration of the data using a triclinic unit cell yielded a total of 27060 reflections to a maximum θ angle of 27.52° (0.77 Å resolution), of which 4739 were independent (average redundancy 5.71, completeness = 99.9%, Rint = 4.69%, Rsig = 3.11%) and 4346 (91.7%) were greater than $2\sigma(F^2)$. The final cell constants of a = 9.3323(4) Å, b = 10.2017(4) Å, c = 11.5883(5) Å, volume = 1036.48(8) $Å^3$, are based upon the refinement of the XYZ-centroids of 9973 reflections above 20 $\sigma(I)$ with 2.36° < 2θ < 27.51°. Data were corrected for absorption effects using the Multi-Scan method in SADABS 2016/2. The calculated minimum and maximum transmission coefficients (based on crystal size) are 0.839 and 0.896. The structure was solved by SHELXT 2018/2 and refined using the SHELXL-2019/2 Software, in the space group *P*-1 (2), with Z = 2 for the formula unit $C_{21}H_{28}BBrO_4$. The final anisotropic full-matrix least-squares refinement on F^2 with 304 variables against 4739 data points 232 and 232 restraints converged at R_1 = 2.52%, for the observed data and wR_2 = 6.35% for all data. The goodness-of-fit on F^2 was 1.05. The largest peak in the final difference electron density synthesis was 0.40 $e^-/Å^3$ and the deepest hole was −0.23 $e^-/Å^3$ with an RMS deviation of 0.058 $e^-/Å^3$. On the basis of the final model, the calculated density was 1.39 g/cm^3 and *F*(000), 452 e^-. CCDC Nr.: 2486407.

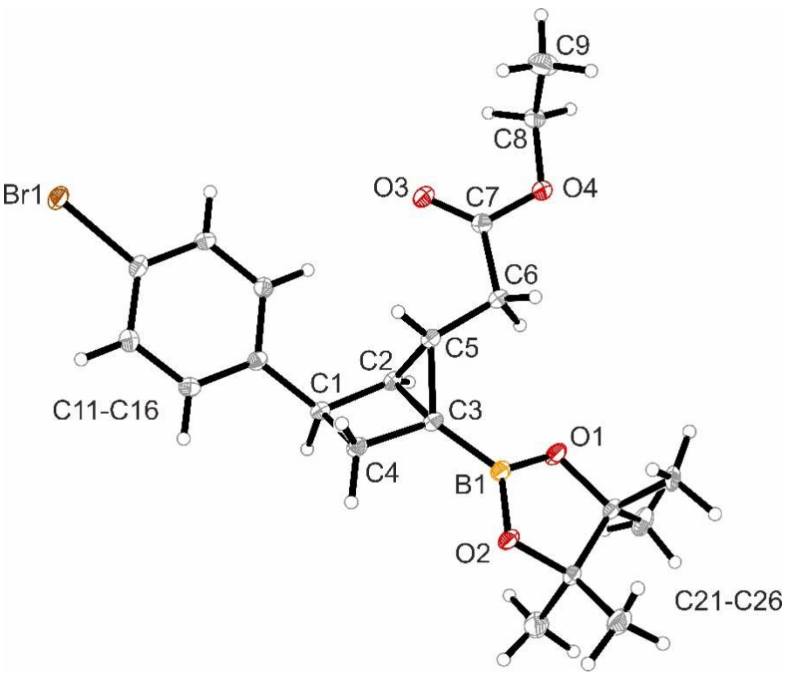

Figure S4a: Crystal structure of compound **35** representing the independent molecule found in the asymmetric unit (chiral centres: C1(*R*), C2(*R*), C5(*S*)). Thermal ellipsoids are shown at 50% probability.

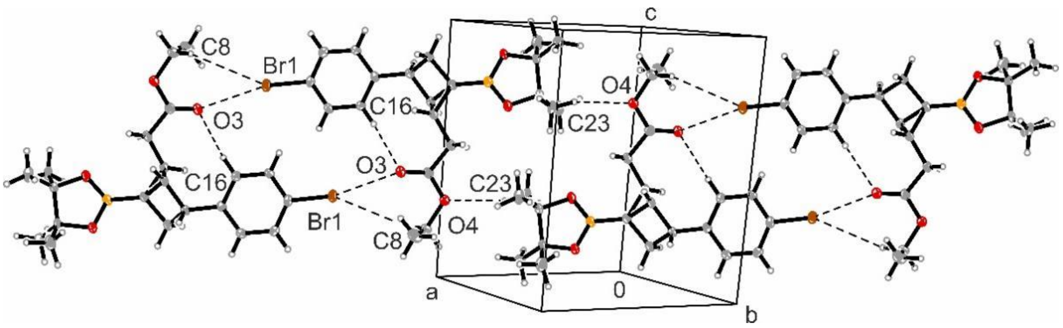

Figure S4b: Excerpt of the packing diagram of compound **35** representing the formation of the linear chains along the *ab*-diagonal trough Br···O, C-H···Br and C-H···O interactions.

Table S5. Non-covalent intermolecular Br···O, C-H···Br and C-H···O interactions in compound **35** (Å and deg).

D-H···*A*	*d*(*D*-H)	*d*(H···*A*)	(*D*··*A*)	∠(*DHA*)
C8A-H8AA···Br1[#1]	0.98	2.882	3.803	155.1
Br1···O3A[#1]			3.080	
C16-H16···O3	0.98	2.580	3.449	152.2
C23B-H23H···O4[#2]	0.98	2.731	3.595	146.6

Symmetry transformations used to generate equivalent atoms: [#1] -x, 1-y, 1-z; [#2] 2-x, -y, 1-z.

X-ray crystal structure analysis of 54 (dan10712): A colorless, prism-like specimen of $C_{25}H_{28.50}N_{1.50}O_3$, approximate dimensions 0.074 mm x 0.101 mm x 0.144 mm, was used for the X-ray crystallographic analysis. The X-ray intensity data were measured on a single crystal diffractometer Bruker D8 Venture Photon III system equipped with a micro focus tube Cu ImS (CuKα, λ = 1.54178 Å) and a MX mirror monochromator. A total of 1694 frames were collected. The total exposure time was 11.63 hours. The frames were integrated with the Bruker SAINT software package using a wide-frame algorithm. The integration of the data using an orthorhombic unit cell yielded a total of 37606 reflections to a maximum θ angle of 67.04° (0.84 Å resolution), of which 7731 were independent (average redundancy 4.864, completeness = 99.1%, R_{int} = 6.11%, R_{sig} = 5.04%) and 7251 (93.79%) were greater than 2σ(F^2). The final cell constants of a = 9.1873(4) Å, b = 18.8274(8) Å, c = 25.4046(12) Å, volume = 4394.3(3) Å^3, are based upon the refinement of the XYZ-centroids of 9242 reflections above 20 σ(I) with 5.842° < 2θ < 133.4°. Data were corrected for absorption effects using the multi-scan method (SADABS). The ratio of minimum to maximum apparent transmission was 0.873. The calculated minimum and maximum transmission coefficients (based on crystal size) are 0.9150 and 0.9550. The structure was solved and refined using the Bruker SHELXTL Software Package, using the space group $P2_12_12_1$, with Z = 8 for the formula unit, $C_{25}H_{28.50}N_{1.50}O_3$. The final anisotropic full-matrix least-squares refinement on F^2 with 616 variables converged at R1 = 5.94%, for the observed data and wR2 = 15.94% for all data. The goodness-of-fit was 1.021. The largest peak in the final difference electron density synthesis was 0.336 e^-/Å^3 and the largest hole was -0.284 e^-/Å^3 with an RMS deviation of 0.057 e^-/Å^3. On the basis of the final model, the calculated density was 1.203 g/cm^3 and F(000), 1704 e^-. The hydrogen atoms at N1A and N1B were refined freely, but with N-H distance restraint (DFIX and U-fixed value). CCDC Nr.: 2486408.

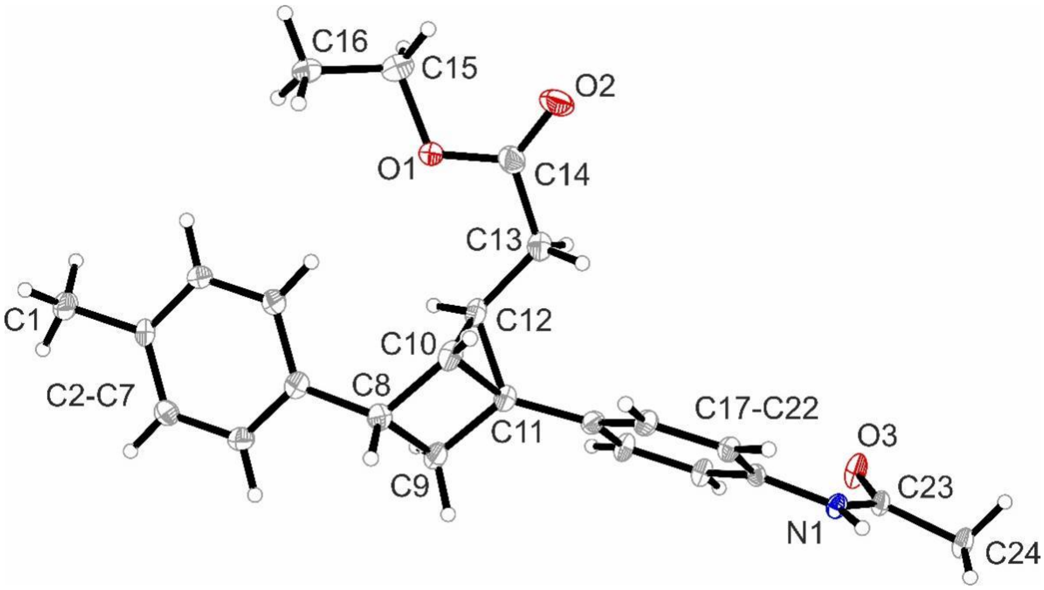

Figure S5a: Crystal structure of compound **54**.
Thermal ellipsoids are shown at 30% probability.
Independent molecule (molecule with suffix “A”) of two
found in the asymmetric unit is shown (chiral centres: C8(*S*), C10(*R*), C12(*R*)).

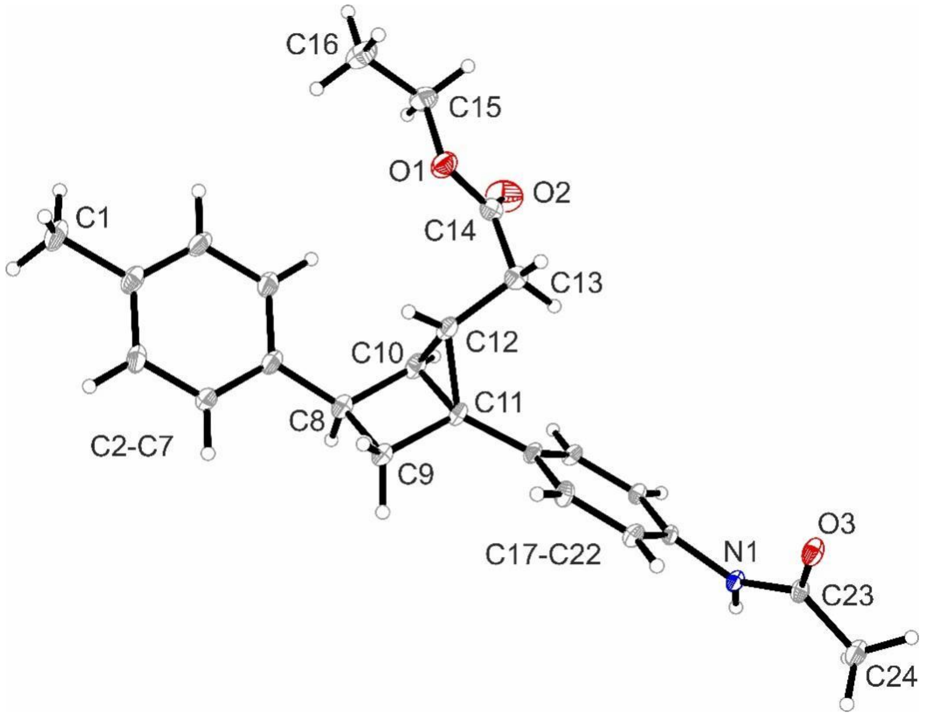

Figure S5b: Independent molecule (molecule with suffix “B”) of two
found in the asymmetric unit is shown (chiral centres: C8(*R*), C10(*S*), C12(*S*)).

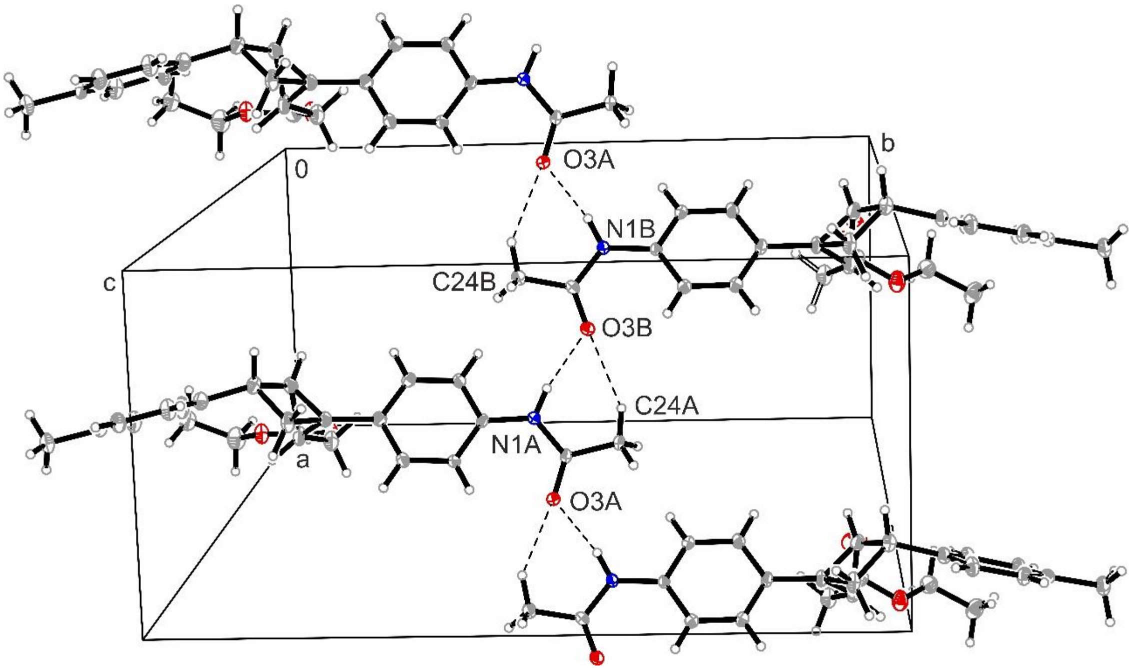

Figure S5c: Excerpt of the packing diagram of compound **54** representing the formation of the linear chains along the *a*-axis trough NH···O and CH···O interactions.

Table S6. Non-covalent intermolecular N-H···O and C-H···O interactions in compound **54** (Å and deg).

D-H···*A*	*d*(*D*-H)	*d*(H···*A*)	(*D*···*A*)	∠(*D*H*A*)
N1A-H1A···O3B[#1]	0.88(3)	2.00(3)	2.859(4)	166(5)
N1B-H1B···O3A	0.89(3)	1.94(3)	2.821(4)	170(5)
C24A-H24B···O3B[#1]	0.98	2.329	3.200	147.7
C24B-H24E···O3A	0.98	2.325	3.151	141.4

Symmetry transformations used to generate equivalent atoms: [#1] -1+x, y, z.

Exit Vector Analysis

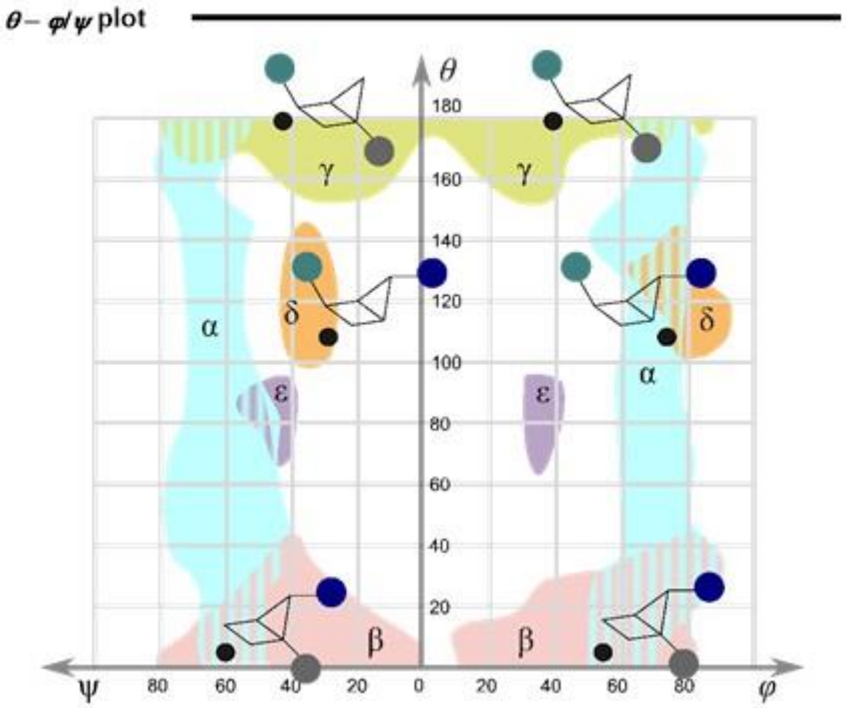

Figure S6. $\theta - \varphi / \psi$ coordinates (two plots at the same graph).

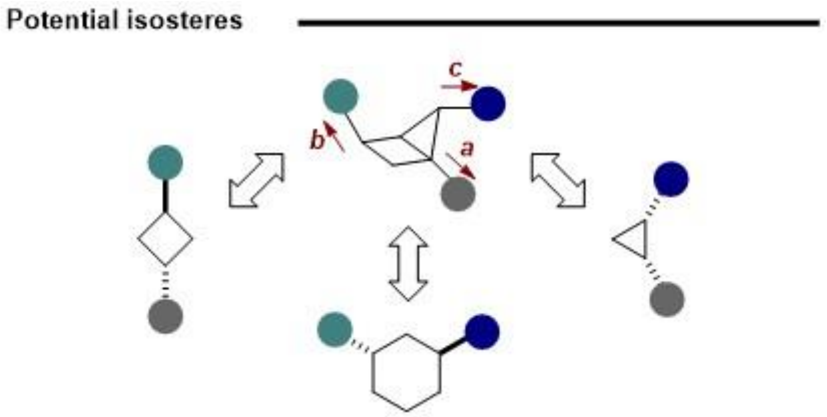

Figure S7. Summary of isosteric replacement potential provided by polysubstituted housanes.

Computational Investigation

1. Computational Details

DFT calculations were carried out using Gaussian16 program package (v C.02) [28] at the ωB97xD level of theory.[29] All the structures were optimized in solution (acetonitrile ε = 38.8) using the SMD implicit solvation method.[30] For optimizations and frequency calculations, Def2SVP was used as the basis set.[31] [32] All the stationary points were characterized as a minima (zero imaginary frequencies) or transition states (one imaginary frequency) by frequency analysis and IRC calculations when needed. Potential energies were further refined by single point energy calculations at the ωB97Xd/Def2TZVPP [31] [32] level of theory, including quasi-harmonic corrections with the Goodvibes program (cut-off of freq = 100 cm^{-1}, method: Grimme, [33] Tº 298.15 K).[34] Final free energies were computed by the addition of thermodynamic free energy corrections at low-theory level to the single point energies at high-theory level, including the 1M standard state correction by adding 1.89 kcal/mol when needed. All the reported energies in the manuscript are free energies in solution calculated at 298 K and 1 atm in kcal/mol.

3D images were created by CYLview software.[35]

2. Energy Profiles

2.1. Complete free energy profile of 5_{syn}

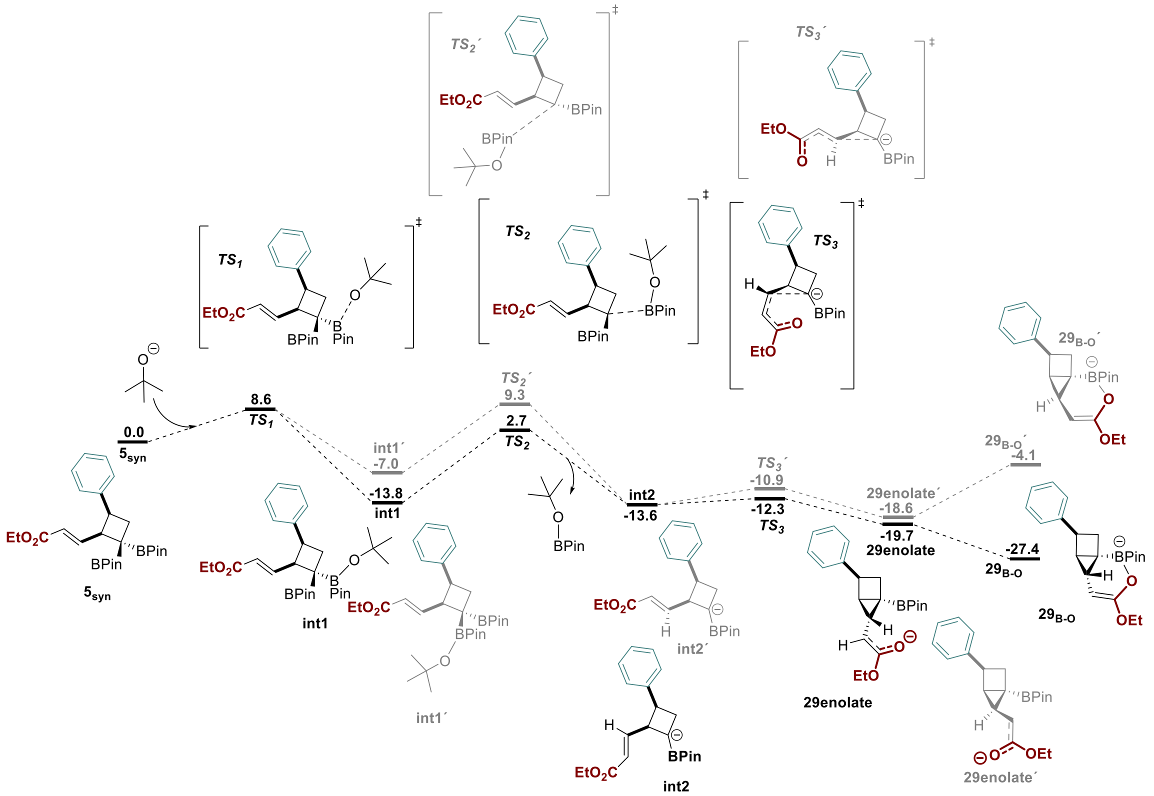

Figure S8. Comparison of free energy profiles (SMD(CH_3CN); ωB97xD/Def2TZVPP//ωB97xD/Def2SVP) of housane formation using *syn*-configuration of initial cyclobutane (**5**), Main pathway is shown in black and secondary pathway in grey. Values in kcal/mol.

2.2. Complete free energy profile of 5_{anti}

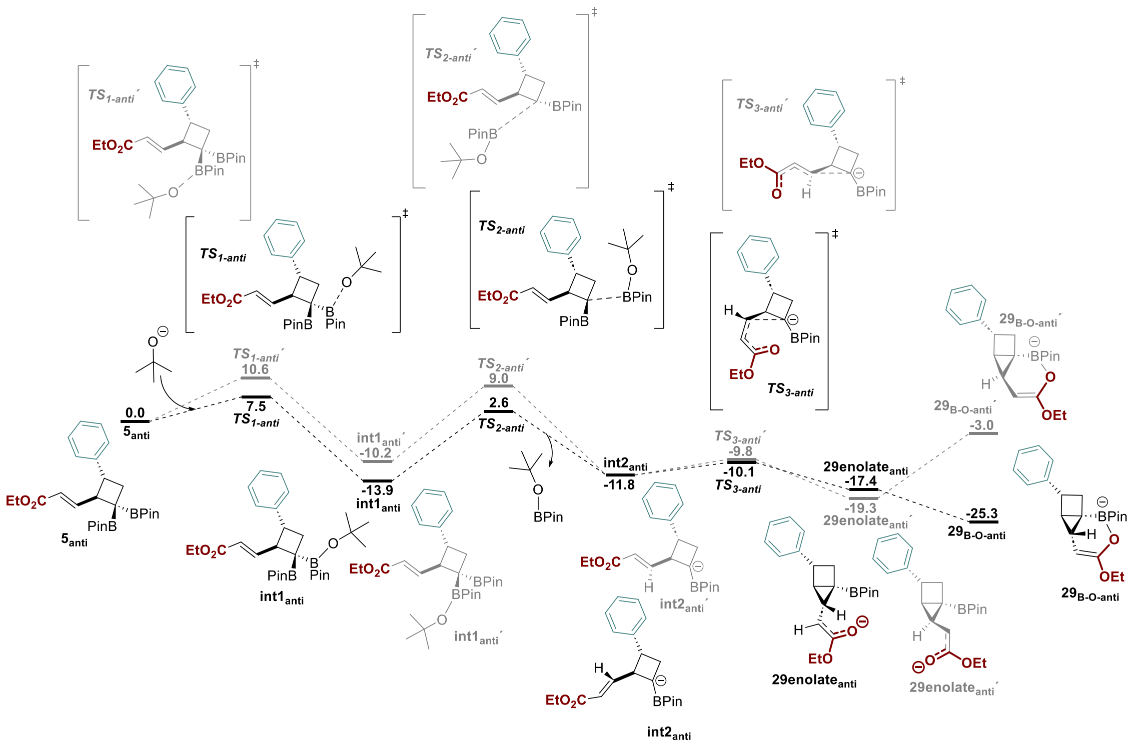

Figure S9. Comparison of free energy profiles (SMD(CH_3CN); ωB97xD/Def2TZVPP//ωB97xD/Def2SVP) of housane formation using *anti*-configuration of initial cyclobutane (**5**$_{anti}$).

2.3 Diastereoselectivity in the initial [2 + 2] mechanism to form borylated cyclobutanes

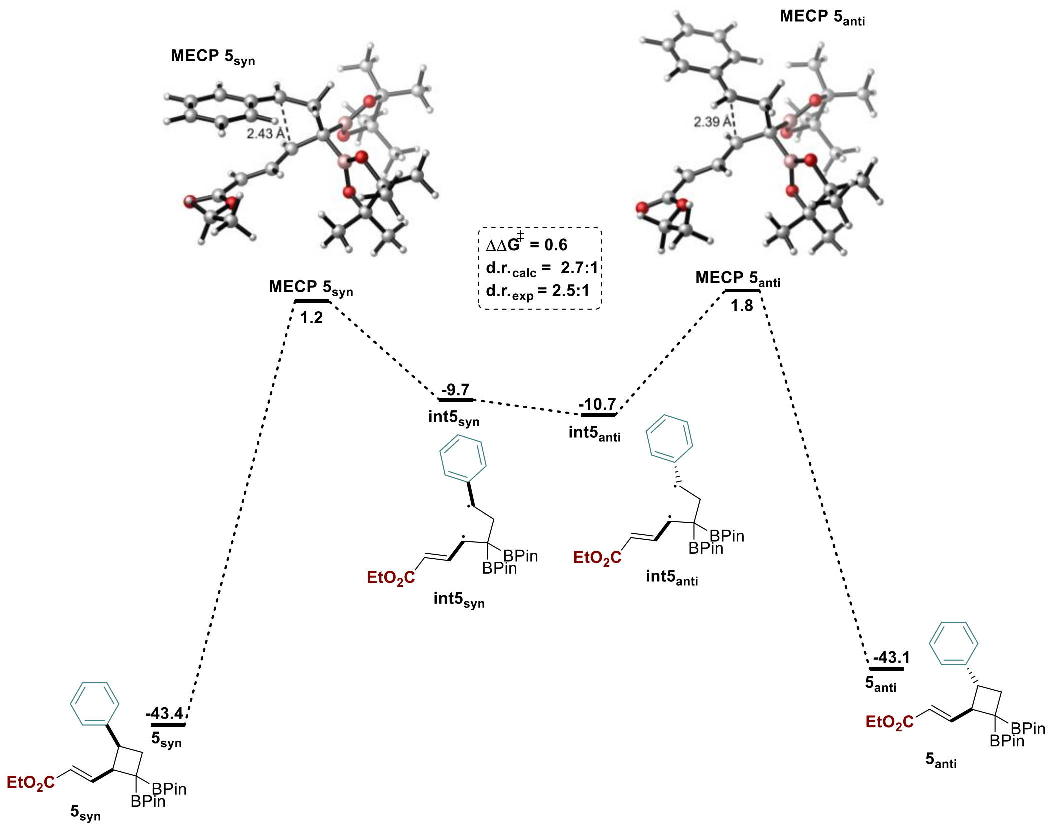

Figure S10: Free energy profile (SMD(CH_3CN); wB97xD/Def2TZVPP//wB97xD/Def2SVP) of borylated cyclobutanes formation via [2 + 2]. Values in kcal/mol.

3.XYZ Coordinates and Energies of the Calculated Species

Final free energies are calculated as the sum of E (basis set employed: ωB97xD) + G_{corr}

5_{syn}

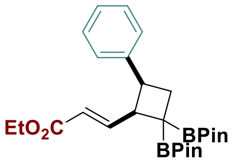

E= -1554.39020975
G_{Corr}= 0.582989

0 1

C	-2.71926800	-0.03913100	0.19820700
H	-3.73270300	0.04257600	0.60066400
C	-1.88239800	1.00532700	0.17281200
H	-0.88437900	0.84687500	-0.25009300
C	-2.30453000	-1.35445100	-0.33806200
O	-3.27767400	-2.25826500	-0.21314100
O	-1.22807300	-1.60642500	-0.83432000
C	-3.01506100	-3.57707200	-0.70186400
H	-2.75814000	-3.51892000	-1.77128100
H	-2.14034600	-3.98837000	-0.17391600
C	-4.24725600	-4.41731700	-0.47227300
H	-5.11359300	-4.00135000	-1.00806600
H	-4.07077600	-5.43808600	-0.84154000
H	-4.49148300	-4.47520900	0.59901300
C	-2.20893100	2.37131800	0.66173600
C	-1.19331700	3.07917700	1.63501400
C	-2.04172900	3.57752400	-0.32554900
H	-3.21367000	2.36776500	1.10774200
C	-1.31040500	4.37032100	0.79321100
H	-1.60577200	3.16867000	2.64979200
H	-0.36697400	4.85854100	0.51111700
H	-1.94721200	5.12007200	1.28468600
C	0.15483500	2.40469900	1.72899800
C	0.36411100	1.45594500	2.74111000
C	1.18579700	2.62156200	0.80513300
C	1.55932200	0.74414800	2.82839700
H	-0.42859800	1.27191400	3.47215700
C	2.38390300	1.90898800	0.88880900
H	1.05758400	3.34294900	-0.00433700
C	2.57631100	0.96668000	1.89836000
H	1.69766200	0.01157500	3.62759800
H	3.17224500	2.09335200	0.15444200
H	3.51423200	0.40983600	1.96249800
C	-0.42332900	2.23751200	-3.46267700
C	0.24012900	3.65797300	-3.34458800
C	-5.46650000	4.25545700	-1.76008000
C	-4.84236600	5.69923000	-1.71888900
B	-3.37711300	4.17891400	-0.88593000
B	-1.19755500	3.30859300	-1.62170000
O	-1.52414900	2.34484500	-2.53572200
O	-0.10133400	4.03721400	-1.99284400
O	-4.57015000	3.51342800	-0.90465400
O	-3.44995400	5.41886200	-1.45884100
C	1.75142200	3.66656800	-3.49607400
H	2.03845300	3.28857700	-4.48874700
H	2.12954200	4.69521500	-3.40024700
H	2.24128600	3.04962900	-2.73129400
C	-0.39428800	4.69367100	-4.26947800
H	-1.49083300	4.69553600	-4.17974900
H	-0.02891300	5.69301900	-3.99070500
H	-0.12849400	4.51025700	-5.32027000
C	-0.96401900	1.89990100	-4.84110100
H	-0.15348400	1.91371000	-5.58503100
H	-1.40079500	0.89024900	-4.83096100
H	-1.74307300	2.60658800	-5.15622000
C	0.47592500	1.11331900	-2.95221000
H	-0.12361200	0.19857500	-2.83810500
H	1.29827200	0.90677700	-3.65208300
H	0.90709900	1.35636400	-1.96889900
C	-5.39068300	3.61036500	-3.14241400
H	-5.65040200	2.54523200	-3.05337800
H	-6.09380900	4.07958600	-3.84513700
H	-4.37620400	3.67756200	-3.56288000
C	-6.87857000	4.15814500	-1.20894000
H	-7.56164700	4.79439400	-1.79140900
H	-7.23513100	3.11984700	-1.28069500
H	-6.92622200	4.46362800	-0.15548200
C	-5.35298900	6.53965500	-0.55096000
H	-4.73276000	7.44399700	-0.46422400
H	-6.39579300	6.85153200	-0.70550500
H	-5.28921700	5.98990700	0.40018300
C	-4.95843200	6.47664900	-3.01914400
H	-6.01579700	6.61623900	-3.28967500
H	-4.50265400	7.47086700	-2.90025600
H	-4.44876500	5.96417600	-3.84562000

tBuO-

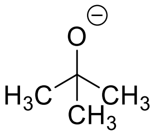

E= -233.173504025

G_{Corr}= 0.091955

-1 1

C	-0.60674600	-0.43084300	0.00693900
C	0.95635600	-0.37730400	-0.08616900
H	-1.00266000	0.60014300	-0.00086000
H	-1.00098100	-0.93524100	-0.89298800
H	-1.00850800	-0.94925700	0.89873000
C	1.45800300	-1.86022600	-0.02875800
C	1.45781000	0.31734600	1.22514400
H	2.56174500	-1.86610400	-0.06541500
H	1.13748600	-2.43394000	0.86210800
H	1.10270600	-2.39188700	-0.92921100
H	1.13622800	-0.16455400	2.16842800
H	2.56154300	0.35112500	1.21261000
H	1.10351800	1.36336400	1.23194200
O	1.37430000	0.25043800	-1.17715300
C	-2.71926800	-0.03913100	0.19820700
H	-3.73270300	0.04257600	0.60066400
C	-1.88239800	1.00532700	0.17281200
H	-0.88437900	0.84687500	-0.25009300

tBuOBPin

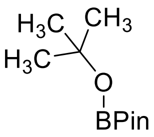

E= -644.480064757

G_{Corr}= 0.269636

0 1

C	-8.96842000	3.30616500	-0.22180500
C	-9.23243800	2.53058200	-1.56560000
B	-8.67220700	4.68029800	-1.99703100
O	-8.99052300	4.67841500	-0.66005100
O	-8.66961800	3.42233200	-2.54563800
C	-7.57947800	3.04004800	0.35544900
H	-7.38614100	3.75692900	1.16717700
H	-7.50231000	2.02431200	0.76894500
H	-6.79578100	3.16764200	-0.40666000
C	-10.02968500	3.10551700	0.84652200
H	-10.10229300	2.04231200	1.12043100
H	-9.76059200	3.67275000	1.75007700
H	-11.01593200	3.45001100	0.50876100
C	-10.71991000	2.38707500	-1.88309900
H	-10.83102700	2.03112800	-2.91799300
H	-11.20731700	1.66211900	-1.21560700
H	-11.24436300	3.35060200	-1.79548000
C	-8.54210800	1.18034800	-1.66208300
H	-8.88046500	0.51670200	-0.85223900
H	-8.79157700	0.70222300	-2.62114500
H	-7.44997900	1.27780300	-1.60427900
C	-7.90098200	7.94149800	-3.49743900
C	-8.26606000	7.11155100	-2.27077000
H	-6.95382600	7.58798100	-3.93256700
H	-8.68610100	7.85988800	-4.26449400
H	-7.78619200	9.00184500	-3.22824900
C	-9.60475500	7.57125100	-1.69429700
C	-7.15463800	7.18800500	-1.22417600
H	-9.87805000	6.97683900	-0.81120600
H	-9.54843700	8.62918000	-1.39712000
H	-10.40077300	7.46716700	-2.44781200
H	-7.00343100	8.22968400	-0.90381400
H	-7.40594500	6.58552300	-0.33997000
H	-6.20759800	6.81596200	-1.64494800
O	-8.38478300	5.76184800	-2.75265600
C	-0.60674600	-0.43084300	0.00693900
C	0.95635600	-0.37730400	-0.08616900
H	-1.00266000	0.60014300	-0.00086000
H	-1.00098100	-0.93524100	-0.89298800

int1

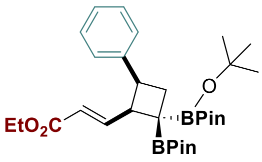

E= -1787.61228798

G_{Corr}= 0.704553

-1 1

C	-2.81181900	-0.07204200	0.23438900
H	-3.75625500	-0.07972000	0.78556300
C	-1.96839600	0.96866600	0.29732000
H	-1.04607400	0.90499300	-0.28886400
C	-2.51609200	-1.24596400	-0.60917500
O	-3.49949400	-2.15023300	-0.54508500
O	-1.52359100	-1.40555000	-1.28769900
C	-3.34562100	-3.34098600	-1.31977700
H	-3.23166100	-3.06926700	-2.38119000
H	-2.42198400	-3.85609400	-1.01140200
C	-4.56196100	-4.20664000	-1.09788100
H	-5.47858900	-3.68713800	-1.41511200
H	-4.46882900	-5.13191700	-1.68507000
H	-4.66322600	-4.47997500	-0.03682900
C	-2.19130600	2.20472500	1.08919000
C	-1.02750500	2.66401400	2.04602500
C	-2.12537800	3.60075500	0.37484800
H	-3.12726100	2.09841000	1.65498700
C	-1.17502000	4.09658700	1.49618100
H	-1.31314100	2.55180300	3.10396800
H	-0.24006700	4.58286800	1.17690800
H	-1.67471700	4.77030300	2.20528900
C	0.28796000	1.95370900	1.84124200
C	0.58031400	0.81525000	2.60811600
C	1.21613300	2.34334000	0.86607700
C	1.75453100	0.09022800	2.41062700
H	-0.13060900	0.49233600	3.37454800
C	2.39362500	1.62009800	0.66478600
H	1.01325500	3.21541800	0.24026200
C	2.66913800	0.49039200	1.43447700
H	1.95822200	-0.79156000	3.02368400
H	3.09970700	1.94369600	-0.10458200
H	3.59126800	-0.07435200	1.27679700
C	-0.69204100	2.90988600	-3.06965100
C	-0.15173300	4.35535000	-2.76181900
C	-5.39137400	3.40856400	-1.05624600
C	-4.88425700	4.70681900	-1.79947200
B	-3.61029900	4.39856400	0.17575200
B	-1.46529900	3.62663400	-1.03566400
O	-1.71047500	2.75029200	-2.07721300
O	-0.43727600	4.49390300	-1.36457500
O	-4.77366400	3.48657400	0.20367800
O	-3.63489900	4.94798700	-1.20689100
C	1.34253300	4.53082300	-2.98637700
H	1.60072700	4.34487200	-4.04015700
H	1.63731600	5.56241900	-2.74124800
H	1.93295000	3.85101300	-2.35727700
C	-0.92761300	5.45178100	-3.49033200
H	-2.00473800	5.35127000	-3.29876400
H	-0.60677300	6.43011300	-3.10128500
H	-0.74228600	5.43665700	-4.57433900
C	-1.29894200	2.74282500	-4.45445100
H	-0.53886800	2.91355300	-5.23215400
H	-1.67985500	1.71674100	-4.57062300
H	-2.13158400	3.43702400	-4.62356000
C	0.34565900	1.81226700	-2.82965400
H	-0.16004600	0.83542000	-2.85068000
H	1.13001000	1.81366600	-3.60071800
H	0.82262100	1.92020400	-1.84514600
C	-4.93700400	2.11668500	-1.75123900
H	-5.16757400	1.26475900	-1.09207300
H	-5.45524000	1.95021600	-2.70922200
H	-3.85346300	2.13010100	-1.93093300
C	-6.90931000	3.34927900	-0.87906700
H	-7.42649600	3.34036300	-1.85225300
H	-7.18424200	2.42560900	-0.34478000
H	-7.28897800	4.19848200	-0.29504200
C	-5.79515700	5.92079700	-1.56573900
H	-5.28189300	6.81861200	-1.94492300
H	-6.76062600	5.83335900	-2.08909600
H	-5.98325900	6.06996900	-0.49584200
C	-4.70818400	4.51520100	-3.30443300
H	-5.65031100	4.20211100	-3.78293400
H	-4.39430800	5.46191300	-3.77289400
H	-3.93786700	3.76208800	-3.51094400
C	-3.80343600	6.61020900	3.22395400
C	-4.43129300	5.49789000	2.37384800
H	-2.74507500	6.38598700	3.43272200
H	-3.84561500	7.56762200	2.68064300
H	-4.32510700	6.73456800	4.18665300
C	-5.91899500	5.81789000	2.15401200

C	-4.32973500	4.16554900	3.13495800
H	-6.37217500	5.03884900	1.52617300
H	-6.46910200	5.86981400	3.10812600
H	-6.03000900	6.78763000	1.64319100
H	-4.86380100	4.22770600	4.09695800
H	-4.77151800	3.35810900	2.53577200
H	-3.28161600	3.91045200	3.34956600
O	-3.74407900	5.49897600	1.15274300

int2

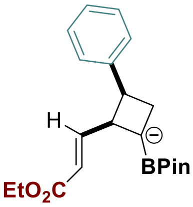

E= -1143.10438
G_{Corr}= 0.404301

-1 1

C	-1.36704300	0.11551900	-0.60449500
H	-2.45439600	0.21297500	-0.55657100
C	-0.55527700	0.90181500	0.14527800
H	0.52748600	0.75683800	0.04754800
C	-0.81635600	-0.77465700	-1.61903300
O	-1.78701200	-1.35971700	-2.35079100
O	0.36252500	-0.98920600	-1.83387000
C	-1.37221700	-2.20910700	-3.41575400
H	-0.70492400	-1.64920900	-4.09101400
H	-0.79026700	-3.05214900	-3.00882000
C	-2.60571600	-2.69280900	-4.14046700
H	-3.17258600	-1.84812500	-4.56062700
H	-2.31499400	-3.35766300	-4.96718800
H	-3.26690100	-3.25398100	-3.46291000
C	-0.99209700	2.05441800	0.96397700
C	0.00127900	2.51638800	2.06056100
C	-0.48840200	3.22717000	0.10201200
H	-2.05413100	1.97238900	1.25987400
C	0.70529500	3.44010100	1.03263700
H	-0.56192500	3.14617500	2.76979900
H	1.67287100	3.02021000	0.68349600
H	0.92545300	4.46137100	1.39907400
C	0.78060000	1.49850800	2.85301100
C	0.17895800	0.29903100	3.26211000
C	2.10998700	1.73181200	3.22810800
C	0.88114300	-0.63498100	4.02310700
H	-0.85578300	0.09181900	2.97448800
C	2.81621600	0.80063000	3.99214700
H	2.60196600	2.65792600	2.91838200
C	2.20493600	-0.38777000	4.39236600
H	0.39232500	-1.56404700	4.32796100
H	3.85272400	1.00465600	4.27330300
H	2.75765100	-1.11980600	4.98637000
C	-1.66164300	3.18743700	-3.40737600
C	-0.69447100	4.42616100	-3.43346100
B	-0.78163200	3.51672000	-1.31987600
O	-1.91127500	3.03494700	-2.01666300
O	0.00449700	4.29121200	-2.20024700
C	0.30477900	4.42313100	-4.58265300
H	-0.21278900	4.41263900	-5.55457700
H	0.92875100	5.32938100	-4.54041400
H	0.97224400	3.55221000	-4.53117900
C	-1.44879100	5.75835000	-3.40757100
H	-2.20565000	5.77033900	-2.60864600
H	-0.73181300	6.56926400	-3.20742200
H	-1.94810900	5.97127900	-4.36478400
C	-2.97916500	3.39472500	-4.14190600
H	-2.80626800	3.61971000	-5.20586600
H	-3.58636600	2.47810400	-4.08366300

H	-3.56302800	4.21381700	-3.70051300
C	-0.99183100	1.90316300	-3.91014200
H	-1.64936300	1.05078400	-3.68201000
H	-0.81753100	1.92254300	-4.99649800
H	-0.03005200	1.72885300	-3.40398000

29enolate

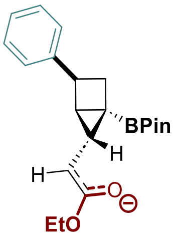

E= -1143.11520975
G_{Corr}= 0.40552

-1 1

C	-1.37250400	-0.30438500	-0.28338400
H	-2.27858400	-0.59946400	0.25531500
C	-0.40188000	0.61168300	0.36501500
H	0.54857700	0.17659600	0.72783400
C	-1.17492600	-0.76914200	-1.57643300
O	-2.20264400	-1.60642000	-2.02471300
O	-0.22708600	-0.53515400	-2.35409400
C	-2.04000800	-2.18877100	-3.29238200
H	-1.97729500	-1.41616800	-4.07995400
H	-1.09419000	-2.75838300	-3.34510900
C	-3.22221400	-3.10025200	-3.54947500
H	-4.16603400	-2.53300500	-3.52669300
H	-3.13238500	-3.57878600	-4.53680900
H	-3.28158100	-3.89214700	-2.78640100
C	-0.92291300	1.74552200	1.18798800
C	0.18082400	2.44997500	1.97491800
C	-0.20921900	2.05413300	-0.13327400
H	-1.99317000	1.83756000	1.39732500
C	0.96727000	2.66384000	0.64496700
H	-0.18052300	3.42688500	2.33546100
H	1.91809900	2.10888200	0.56770200
H	1.16025800	3.72260000	0.42247600
C	0.84714300	1.73312500	3.12370800
C	0.22452600	0.66341600	3.77763600
C	2.10865500	2.14545300	3.57586900
C	0.83968600	0.02670200	4.85717000
H	-0.75386600	0.31933200	3.43126500
C	2.72795000	1.51113100	4.65230500
H	2.61325400	2.97698100	3.07485900
C	2.09419500	0.44776400	5.29883900
H	0.33623800	-0.80708100	5.35338200
H	3.71237000	1.84736900	4.98817600
H	2.57850400	-0.05219200	6.14123900
C	-2.09414000	2.95090800	-3.27571300
C	-0.71825700	3.68966700	-3.47485900
B	-0.74166800	2.56309100	-1.49345200
O	-2.05743300	2.61621800	-1.87724100
O	0.09309700	3.11282500	-2.43820400
C	-0.05752400	3.44098700	-4.82176500
H	-0.70379400	3.79108800	-5.64096000
H	0.89317300	3.99243400	-4.87853900
H	0.15703600	2.37514900	-4.97478600
C	-0.79768200	5.19067300	-3.19887600
H	-1.28437600	5.39527700	-2.23344100
H	0.22264700	5.60057400	-3.15850200
H	-1.34984400	5.72064400	-3.98850900
C	-3.31892700	3.80873300	-3.55146100
H	-3.32213400	4.15237200	-4.59707900
H	-4.23132300	3.21630000	-3.38576100
H	-3.36019600	4.68711100	-2.89364400
C	-2.18405900	1.63473700	-4.04655600

H	-3.09069600	1.10118000	-3.72254800
H	-2.25220500	1.80192000	-5.13156400
H	-1.32369500	0.98686600	-3.81895100
C	-0.60674600	-0.43084300	0.00693900

29 B-O

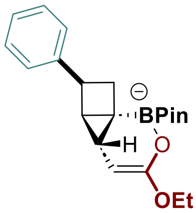

E= -1143.12903431

G_{Corr}= 0.407047

-1 1

C	-0.07282300	-0.97969300	-0.47423700
H	0.35201600	-1.94436700	-0.18882500
C	0.19779900	0.23567500	0.34187100
H	1.19779500	0.28565400	0.79841000
C	-0.93817800	-0.94173100	-1.52008700
O	-1.26956500	-2.10441000	-2.13893500
O	-1.54236400	0.11920600	-1.98977900
C	-1.77490600	-2.02956700	-3.46214000
H	-2.72558400	-1.47308500	-3.47932500
H	-1.06526000	-1.48220300	-4.10784600
C	-1.97372900	-3.44135600	-3.96518300
H	-2.68815800	-3.98589600	-3.32877300
H	-2.37051700	-3.42291700	-4.99116000
H	-1.02302700	-3.99602000	-3.97123600
C	-0.94474500	0.84246900	1.12464500
C	-0.48395800	2.03709100	1.95486200
C	-0.44126800	1.50639100	-0.16665700
H	-1.87249300	0.28167900	1.28442700
C	0.08603800	2.68722000	0.65583500
H	-1.35381000	2.62113900	2.29873600
H	1.17926200	2.84106200	0.63451300
H	-0.40497900	3.64120200	0.41550900
C	0.43115900	1.81499500	3.13551600
C	0.47496400	0.58473100	3.80265700
C	1.24684500	2.85389800	3.60654800
C	1.30296200	0.39724400	4.91090700
H	-0.14554800	-0.24077900	3.44311800
C	2.07717800	2.67116600	4.71216100
H	1.23079200	3.82205700	3.09719800
C	2.10832100	1.43987900	5.37024100
H	1.32156600	-0.57184100	5.41644400
H	2.70593000	3.49440000	5.06144400
H	2.76020900	1.29348600	6.23500700
C	-1.98887700	3.03516400	-3.16548400
C	-0.42952700	3.02643500	-3.38345400
B	-1.03233400	1.53450700	-1.66344600
O	-2.11648200	2.48161400	-1.87507100
O	-0.01742200	1.88757400	-2.66810000
C	0.00042200	2.89872900	-4.84328000
H	-0.39198800	3.72803100	-5.45360000
H	1.09981200	2.92041200	-4.91244500
H	-0.34227100	1.95044700	-5.27983600
C	0.24521300	4.26370900	-2.77380600
H	-0.09446600	4.42790300	-1.74091100
H	1.33313100	4.09457900	-2.74839800
H	0.05413700	5.18030100	-3.35364000
C	-2.62141400	4.42372300	-3.18285500
H	-2.43687700	4.93704500	-4.14004100
H	-3.71195900	4.34031500	-3.05019200
H	-2.23383300	5.05150600	-2.36866600
C	-2.71972300	2.14247800	-4.17983800
H	-3.76107900	2.01044900	-3.84611700
H	-2.73757400	2.58338700	-5.18877500

H	-2.25706000	1.14722800	-4.23273300
C	-0.60674600	-0.43084300	0.00693900

TS1

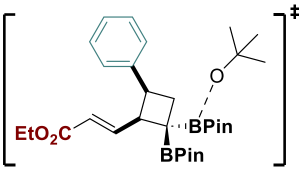

E= -1787.57269736

G_{Corr}= 0.700616

-1 1

C	-2.65903100	-0.13322800	0.26365900
H	-3.66627200	-0.09605800	0.68787900
C	-1.86132600	0.94270700	0.24365600
H	-0.86542700	0.82541900	-0.19761100
C	-2.20685800	-1.42318700	-0.29961700
O	-3.13080400	-2.37372200	-0.14002700
O	-1.14156300	-1.62461200	-0.84196000
C	-2.82402300	-3.67756100	-0.64078700
H	-2.61935500	-3.60945000	-1.72092700
H	-1.90635200	-4.04349900	-0.15380500
C	-3.99997500	-4.57922900	-0.35518700
H	-4.90998600	-4.20931400	-0.85085600
H	-3.78874600	-5.59080900	-0.73150500
H	-4.19200300	-4.64628800	0.72617400
C	-2.22623800	2.28583300	0.76425200
C	-1.22286600	3.00420600	1.74099800
C	-2.11686300	3.51837100	-0.19221300
H	-3.22287600	2.24658200	1.22262300
C	-1.42051700	4.31318100	0.94615000
H	-1.61742000	3.03841300	2.76837700
H	-0.50765600	4.86809900	0.68199000
H	-2.16041900	4.95975500	1.44479100
C	0.15328400	2.38507200	1.78153400
C	0.43222400	1.40877400	2.75018200
C	1.15152800	2.68451100	0.84433700
C	1.66062600	0.75048800	2.78211800
H	-0.33322700	1.15998700	3.49120400
C	2.38272700	2.02588800	0.87175300
H	0.96807700	3.43084400	0.06878600
C	2.64352900	1.05544600	1.83861700
H	1.85194800	-0.00484100	3.54867000
H	3.14347500	2.27523500	0.12730200
H	3.60736000	0.54100000	1.85952400
C	-0.49747800	2.33848900	-3.39434500
C	0.06995900	3.79773700	-3.26340800
C	-5.54867000	4.02306800	-1.69477400
C	-4.91709100	5.47043900	-1.82528300
B	-3.48853600	4.07918700	-0.72660800
B	-1.29054200	3.31996400	-1.50605800
O	-1.57205500	2.35091300	-2.43618600
O	-0.24561400	4.11942700	-1.89411200
O	-4.63862000	3.34328100	-0.81856300
O	-3.54785500	5.24429300	-1.45272400
C	1.57026000	3.91630000	-3.47090400
H	1.84559500	3.58301100	-4.48289000
H	1.87977200	4.96645100	-3.36189900
H	2.13079400	3.31799400	-2.74041000
C	-0.67048200	4.80617400	-4.13938900
H	-1.75946400	4.72972400	-4.00334000
H	-0.36450000	5.82249700	-3.85025100
H	-0.43629300	4.66530400	-5.20431900
C	-1.05353400	1.99187400	-4.76504700
H	-0.26847500	2.07553400	-5.53158800
H	-1.42109000	0.95481600	-4.76500800
H	-1.88768000	2.64949300	-5.04326400
C	0.49353500	1.26907400	-2.93716200
H	-0.03794200	0.31251700	-2.82740800

H	1.30706800	1.13449800	-3.66459000
H	0.93576800	1.52133100	-1.96118900
C	-5.55294800	3.24046600	-3.00794000
H	-5.85029200	2.20133500	-2.80114500
H	-6.26097900	3.66259800	-3.73584700
H	-4.55108100	3.21858200	-3.46139100
C	-6.93549100	4.00097400	-1.07001800
H	-7.65281500	4.56581600	-1.68466100
H	-7.29282200	2.96256700	-0.99590800
H	-6.91893800	4.43098900	-0.06025400
C	-5.48100900	6.48036300	-0.82703100
H	-4.95812700	7.44003000	-0.97055400
H	-6.55567000	6.65905300	-0.98208800
H	-5.26769600	6.12797400	0.20136000
C	-4.95185500	6.04107800	-3.23656500
H	-5.99099700	6.18412900	-3.57071000
H	-4.45446100	7.02262500	-3.24761900
H	-4.43860600	5.39389900	-3.96041600
C	-3.98472800	5.59302100	4.08993700
C	-4.80454100	4.97173900	2.92456800
H	-2.97769500	5.14189000	4.11819500
H	-3.85764700	6.67353600	3.90622400
H	-4.44405600	5.46243300	5.08719500
C	-6.23479300	5.57811200	2.98874200
C	-4.93557500	3.45057900	3.21393800
H	-6.86274400	5.13862300	2.19481700
H	-6.74786900	5.41773600	3.95485000
H	-6.18296100	6.66533500	2.80615000
H	-5.42403000	3.22217100	4.17909800
H	-5.51940000	2.96688700	2.41292300
H	-3.93496200	2.98697100	3.22394200
O	-4.21713200	5.21925100	1.73250300
C	-0.60674600	-0.43084300	0.00693900

TS2

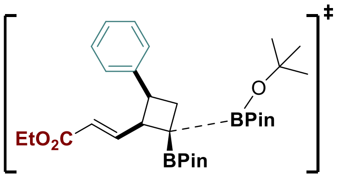

E= -1787.58269362

G_{Corr}= 0.701172

-1 1

C	-2.67084300	-0.20894900	0.06285900
H	-3.68235900	-0.19731700	0.47783100
C	-1.81905700	0.81407600	0.24463300
H	-0.82491800	0.72439400	-0.20738100
C	-2.28200500	-1.39419100	-0.72135700
O	-3.25873200	-2.31037700	-0.73819900
O	-1.22486300	-1.55692500	-1.29412500
C	-3.01523200	-3.51301500	-1.46867000
H	-2.79243700	-3.26109000	-2.51772000
H	-2.12528500	-4.01421100	-1.05570300
C	-4.24156300	-4.38637000	-1.35883400
H	-5.12342600	-3.88279900	-1.78258900
H	-4.07957400	-5.32270200	-1.91265600
H	-4.45309700	-4.63871200	-0.30886600
C	-2.10371100	2.08306300	0.96474100
C	-1.09852700	2.48019600	2.12076500
C	-1.71457100	3.39417500	0.26731200
H	-3.14049800	2.04698000	1.35661600
C	-0.98551800	3.89402100	1.50717500
H	-1.57226700	2.43209800	3.11564700
H	0.05195800	4.26445900	1.39125800
H	-1.52936800	4.65498000	2.09852900
C	0.15959900	1.65005700	2.15458600
C	0.27838600	0.59570300	3.07089200
C	1.20765800	1.84800700	1.24210300
C	1.39997800	-0.23462500	3.07995500
H	-0.52729000	0.42192300	3.79052800
C	2.32891900	1.01790200	1.24444600
H	1.13462500	2.65177300	0.50582000
C	2.43152400	-0.02778800	2.16344500
H	1.46790500	-1.04765600	3.80757200
H	3.12918000	1.18937500	0.51965400
H	3.31113500	-0.67630400	2.16609100
C	-0.74889600	3.10880100	-3.30774800
C	-0.24969200	4.55628300	-2.95742900
C	-5.52588800	3.46045600	-0.95721400
C	-5.15239400	4.70511200	-1.85190200
B	-4.02707300	4.81155000	0.13258500
B	-1.28804500	3.60329400	-1.13308600
O	-1.69047500	2.86151700	-2.27193800
O	-0.33587900	4.56746200	-1.53906200
O	-4.98297100	3.81891300	0.31506100
O	-4.00726900	5.24652100	-1.19224800
C	1.18427300	4.84701600	-3.38113800
H	1.30313300	4.73878400	-4.47066800
H	1.45200600	5.88075500	-3.11266400
H	1.89694900	4.17579700	-2.88267200
C	-1.18248100	5.64542000	-3.49284400
H	-2.21779800	5.45159900	-3.17930300
H	-0.87826500	6.61229700	-3.06258600
H	-1.14110900	5.73194700	-4.58929100
C	-1.42222500	2.97941200	-4.66670600
H	-0.71616300	3.22276000	-5.47597700
H	-1.76531000	1.94378400	-4.81599200
H	-2.29322200	3.64091700	-4.75705700
C	0.36119300	2.05839700	-3.18693200
H	-0.09528700	1.05744700	-3.21818100
H	1.09434100	2.12899300	-4.00445300
H	0.89640200	2.15283500	-2.22963700
C	-4.86149100	2.16685000	-1.42166000
H	-5.00642300	1.40132000	-0.64573000
H	-5.31714200	1.79542400	-2.35204300
H	-3.78428800	2.30203500	-1.58850200
C	-7.02370100	3.23076500	-0.79595900
H	-7.49760300	3.04448100	-1.77186900
H	-7.19539700	2.34708900	-0.16235800
H	-7.52224400	4.08755700	-0.32309700
C	-6.23093200	5.79154200	-1.84438700
H	-5.82729000	6.69246200	-2.33069000
H	-7.13353600	5.48136100	-2.39141700
H	-6.51975700	6.06330400	-0.81791200
C	-4.79387800	4.34118600	-3.28411900
H	-5.62881300	3.81790600	-3.77532700
H	-4.57912900	5.25249700	-3.86343600
H	-3.90357100	3.70135200	-3.30196300
C	-3.40860700	6.80005900	3.17901400
C	-4.23458500	5.76273300	2.42080100
H	-2.37308600	6.44830900	3.30609000
H	-3.38345900	7.75028500	2.62372200
H	-3.83441700	6.98997800	4.17583300
C	-5.66745300	6.27134700	2.23093600
C	-4.23712100	4.43662000	3.18630600
H	-6.26838400	5.53236800	1.68167000
H	-6.14923700	6.45666000	3.20330800
H	-5.66524800	7.21399800	1.66162800
H	-4.68200700	4.57682000	4.18371400
H	-4.81699400	3.67680600	2.64614700
H	-3.21127100	4.06456500	3.31969800
O	-3.60663200	5.63626800	1.14598400
C	-0.60674600	-0.43084300	0.00693900

TS3

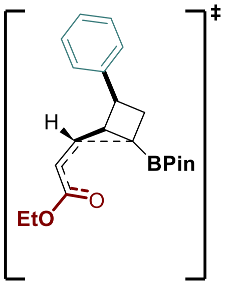

E= -1143.102301

G_{Corr}= 0.404358

-1 1

C	-1.32976500	0.14037400	-0.63925600
H	-2.41755100	0.22053100	-0.57867300
C	-0.51412200	0.93931200	0.15029700
H	0.56241100	0.73873700	0.14096200
C	-0.77102400	-0.64404700	-1.69866300
O	-1.72598800	-1.30222900	-2.41871200
O	0.41485300	-0.76130600	-1.99052800
C	-1.28168900	-2.06098900	-3.52975500
H	-0.70679600	-1.41772400	-4.21734300
H	-0.59792100	-2.85856200	-3.19312300
C	-2.49433200	-2.64272500	-4.21989000
H	-3.16248100	-1.84564900	-4.58080400
H	-2.18206500	-3.24748600	-5.08439100
H	-3.06506300	-3.28887700	-3.53538800
C	-0.98342400	2.00519000	1.03816800
C	0.04789400	2.50958600	2.06484600
C	-0.40368600	2.99669500	0.02442500
H	-2.04851800	1.96347200	1.31660500
C	0.79613200	3.26409100	0.92861300
H	-0.45039000	3.25311200	2.70907100
H	1.74305500	2.76757600	0.63362700
H	1.04365400	4.31578600	1.15727800
C	0.78110100	1.52750200	2.94152600
C	0.18920900	0.31621300	3.32439800
C	2.06722000	1.81638700	3.41766700
C	0.85867900	-0.57705100	4.16157400
H	-0.80844900	0.06387200	2.95448700
C	2.73998900	0.92636000	4.25565900
H	2.55030000	2.75369500	3.12687700
C	2.13766600	-0.27538700	4.63158900
H	0.37894000	-1.51747000	4.44516000
H	3.74292000	1.17172000	4.61467900
H	2.66462200	-0.97569600	5.28433700
C	-1.76701800	3.17762700	-3.42079200
C	-0.67575900	4.30795100	-3.45819000
B	-0.76576500	3.34740900	-1.37726900
O	-1.95797600	2.99579200	-2.01832900
O	0.06086600	4.05623100	-2.26148900
C	0.26567600	4.23816700	-4.65216600
H	-0.29318500	4.31803200	-5.59742200
H	0.98489800	5.07067700	-4.61150800
H	0.83578400	3.29947100	-4.65923800
C	-1.27754800	5.71106800	-3.35241700
H	-1.98854400	5.77735700	-2.51500300
H	-0.46748000	6.43285200	-3.16747900
H	-1.79691200	6.00758700	-4.27586500
C	-3.09255800	3.55207600	-4.06766400
H	-2.95232700	3.80421200	-5.13015900
H	-3.78852200	2.70110000	-4.01019700
H	-3.56258400	4.40707900	-3.56321100
C	-1.26442900	1.85204000	-4.00077600
H	-1.98613700	1.05969800	-3.75384700
H	-1.15676100	1.89579900	-5.09495400
H	-0.29721600	1.56405400	-3.56272300

int1´

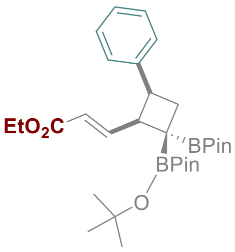

E= -1787.60020831

G_{Corr}= 0.703268

-1 1

C	-1.78474800	-0.48903800	-0.86197100
H	-2.77567100	-0.93602600	-0.73613600
C	-1.51618000	0.75505200	-0.43448400
H	-0.52215100	1.17553600	-0.60371800
C	-0.76026500	-1.32021200	-1.52140600
O	-1.26902000	-2.49251500	-1.92121600
O	0.40158900	-1.02006800	-1.69405100
C	-0.38131800	-3.40143400	-2.57426600
H	0.03768700	-2.91652400	-3.47031500
H	0.46050600	-3.63142400	-1.90191800
C	-1.15959200	-4.64445100	-2.93151600
H	-1.99264200	-4.40660900	-3.60995100
H	-0.49744900	-5.36270200	-3.43681000
H	-1.56813900	-5.12633500	-2.03057100
C	-2.47299100	1.60313900	0.32168600
C	-2.03719500	1.92191000	1.80292400
C	-2.65654300	3.14573400	0.07189800
H	-3.44704700	1.08520200	0.34747500
C	-2.57765900	3.34942600	1.61280700
H	-2.57972900	1.30225400	2.53391800
H	-1.92854000	4.16791700	1.95125000
H	-3.56053400	3.47545600	2.09875000
C	-0.55847700	1.78647200	2.07745000
C	-0.06630000	0.60793600	2.65715000
C	0.36157000	2.78583700	1.72757400
C	1.29862200	0.42679200	2.88255400
H	-0.76837100	-0.18370400	2.93624100
C	1.72705100	2.60695900	1.95198600
H	0.00605000	3.70112200	1.24795000
C	2.20348700	1.42895700	2.52959300
H	1.65646000	-0.50053800	3.33766800
H	2.42718000	3.39691700	1.66626700
H	3.27374900	1.29240300	2.70435700
C	-1.68780400	3.70295300	-3.23669500
C	-0.22383700	3.40297000	-2.73464100
C	-6.01893800	4.72154700	-0.51754300
C	-6.00800900	3.63219300	-1.65035000
B	-4.04151000	3.57738000	-0.49177200
B	-1.59246500	4.07338800	-0.88112600
O	-2.24623000	4.43208800	-2.17535000
O	-0.36786100	3.36060400	-1.33745300
O	-4.64339500	4.77101700	-0.13377200
O	-4.89484200	2.81231600	-1.26449000
C	0.34895300	2.07719600	-3.23931200
H	0.41039400	2.06388100	-4.33971100
H	1.36869100	1.94114200	-2.84462700
H	-0.24150300	1.21381000	-2.91291200
C	0.77131400	4.50321200	-3.13254700
H	0.39394300	5.50336200	-2.88913500
H	1.71045100	4.34962200	-2.57802600
H	1.00555300	4.47862300	-4.20833400
C	-1.75764500	4.53995900	-4.51419600
H	-1.24497500	4.04022100	-5.35189800
H	-2.81056700	4.68530700	-4.80475200
H	-1.30943400	5.53341300	-4.37615200
C	-2.48470900	2.41142000	-3.46735600
H	-3.53973200	2.66316000	-3.62849400

H	-2.12003900	1.85234600	-4.34341000
H	-2.43760700	1.75286000	-2.59133700
C	-6.81925800	4.29107100	0.71296500
H	-6.61264700	4.99286400	1.53503300
H	-7.90231700	4.29610300	0.52077900
H	-6.52806600	3.28356800	1.04689500
C	-6.46508300	6.10551700	-0.96571400
H	-7.49231200	6.07789400	-1.36049900
H	-6.44917100	6.79612900	-0.10880500
H	-5.80124300	6.51212500	-1.74026900
C	-5.70197600	4.22559700	-3.02536500
H	-5.58775300	3.41013200	-3.75505400
H	-6.51612700	4.87694100	-3.37493500
H	-4.76067000	4.79364100	-2.99827000
C	-7.26060000	2.77221800	-1.71981500
H	-8.14655900	3.39164100	-1.92775700
H	-7.16162000	2.03708400	-2.53288300
H	-7.42947600	2.22161200	-0.78472100
C	-1.44345700	7.31861600	1.02126300
C	-0.89859800	6.53751400	-0.18523900
H	-2.54434700	7.28452200	1.02359400
H	-1.08835200	6.86211300	1.95897000
H	-1.12537600	8.37377100	1.00853200
C	0.63695600	6.59129800	-0.14925800
C	-1.42962700	7.20130600	-1.46354000
H	1.06749000	5.99047300	-0.95982000
H	1.01243400	7.62369300	-0.24285300
H	1.00374400	6.18029800	0.80528100
H	-1.13226100	8.26241300	-1.49597100
H	-1.05686800	6.70352600	-2.36601400
H	-2.52760800	7.14344800	-1.49307300
O	-1.33781500	5.22488900	-0.01340300

int2 ´

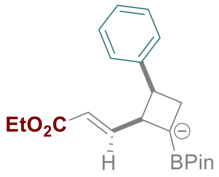

E= -1143.100321
G_{Corr}= 0.403935

-1 1

C	1.39771900	0.82075400	-0.40953800
H	2.28189800	0.46089700	0.11676400
C	0.51351800	-0.01843600	-1.02715300
H	-0.30162300	0.45484300	-1.58735200
C	1.16579300	2.25617500	-0.41231400
O	2.15891200	2.92373300	0.21508500
O	0.22186600	2.84531000	-0.91158900
C	2.05166400	4.34103000	0.27377200
H	2.00736000	4.75114700	-0.74853300
H	1.10986800	4.61978500	0.77427300
C	3.24884300	4.87382000	1.02540900
H	4.18618800	4.60343300	0.51593600
H	3.19351100	5.97079900	1.08628100
H	3.28107400	4.47267400	2.04976400
C	0.40487300	-1.49356400	-0.98902300
C	1.31875600	-2.26966000	-0.00947000
C	-0.68599300	-1.54281600	0.09605100
H	0.24060100	-1.94356700	-1.98400900
C	0.35731500	-1.90039200	1.14736300
H	1.17087700	-3.33921100	-0.23659000
H	0.73861300	-1.04188700	1.74450400
H	0.13974800	-2.71472400	1.86211900
C	2.80235900	-2.01944800	0.06478400
C	3.55572200	-1.86881600	-1.10834800
C	3.47040300	-1.95618300	1.29376400
C	4.93216600	-1.65450100	-1.05554700

H	3.05107700	-1.90877000	-2.07806300
C	4.84924400	-1.74298800	1.35152600
H	2.90321700	-2.06867900	2.22169700
C	5.58591600	-1.58893600	0.17703400
H	5.49911100	-1.53346200	-1.98234100
H	5.35002500	-1.69340100	2.32199500
H	6.66413700	-1.41650200	0.22075200
C	-4.21232100	-0.52592100	0.76580500
C	-3.92996100	0.09072000	-0.65125200
B	-2.07484800	-1.03015000	0.09019100
O	-2.90160300	-0.85457000	1.21744800
O	-2.77944300	-0.63573700	-1.06851700
C	-5.04990400	-0.10465200	-1.66366600
H	-5.98459400	0.35803100	-1.31063500
H	-4.77678700	0.36926500	-2.61917300
H	-5.23695800	-1.16950800	-1.85761600
C	-3.55068200	1.57296300	-0.57887800
H	-2.75705500	1.74591900	0.16347600
H	-3.16494300	1.89025500	-1.55969700
H	-4.41164200	2.20986200	-0.32653100
C	-4.86279000	0.43121000	1.75526000
H	-5.83063100	0.79283700	1.37455800
H	-5.04553200	-0.08435400	2.71078300
H	-4.21908200	1.29776500	1.95831800
C	-5.02494900	-1.82127300	0.68822200
H	-4.99992000	-2.31622700	1.67102200
H	-6.07696000	-1.63498700	0.42500000
H	-4.59740900	-2.51493500	-0.05162600

29enolate ´

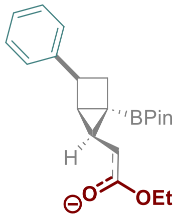

E= -1143.113531
G_{Corr}= 0.405592

-1 1

C	1.37604800	0.81166400	-0.45322300
H	2.40535000	0.51777100	-0.24361300
C	0.37507400	-0.16168200	-0.93423600
H	-0.31166300	0.23583900	-1.69413100
C	0.95737200	2.10632400	-0.14920300
O	1.98688800	2.92169400	0.32152000
O	-0.18072800	2.60274400	-0.25995400
C	1.64801900	4.24744400	0.64240400
H	1.25306400	4.77761100	-0.24349400
H	0.84302700	4.27449000	1.39895400
C	2.88872500	4.94188300	1.16354100
H	3.68724700	4.94064100	0.40515200
H	2.66319700	5.98725700	1.42442700
H	3.27171400	4.43690100	2.06411700
C	0.51116100	-1.63551800	-1.08779900
C	1.35912100	-2.29643100	0.00552600
C	-0.45809400	-1.12334400	-0.01342700
H	0.24445900	-2.14317800	-2.02073400
C	0.46375900	-1.63461200	1.09302400
H	1.14860100	-3.37907500	-0.00034000
H	0.95580900	-0.83951100	1.67920100
H	0.00405500	-2.35602100	1.78300500
C	2.85593300	-2.11375700	0.01822700
C	3.57186000	-1.96740700	-1.17666600
C	3.56822600	-2.11624300	1.22426800
C	4.95970200	-1.82691600	-1.16842300
H	3.03166100	-1.94983700	-2.12733500
C	4.95641000	-1.97418300	1.23745900

H	3.02789200	-2.22547500	2.16890500
C	5.65850500	-1.82830400	0.04012200
H	5.49894900	-1.70929000	-2.11198200
H	5.49294300	-1.97403400	2.18987200
H	6.74512200	-1.71278000	0.04876400
C	-4.04394300	-0.28781000	0.72183400
C	-3.90663200	-0.00847800	-0.82040900
B	-1.96584800	-0.85350800	-0.01764100
O	-2.67830800	-0.52454400	1.11055700
O	-2.73049600	-0.76559300	-1.15927300
C	-5.07821300	-0.49244900	-1.65857800
H	-6.00615600	0.01385800	-1.35225100
H	-4.89807500	-0.26014400	-2.71890300
H	-5.22387200	-1.57702600	-1.56834700
C	-3.59525800	1.45486200	-1.13906900
H	-2.71343700	1.81290500	-0.58451000
H	-3.36597800	1.53983800	-2.21223700
H	-4.45066600	2.11052900	-0.92093100
C	-4.59150600	0.87967500	1.52720900
H	-5.59792600	1.15395000	1.17630600
H	-4.66519500	0.59936800	2.58878300
H	-3.94010900	1.76048700	1.45205900
C	-4.83169600	-1.55927800	1.03206200
H	-4.71046400	-1.80290200	2.09811400
H	-5.90474900	-1.43019500	0.82928800
H	-4.46413500	-2.41324100	0.44350400

29 B-O´

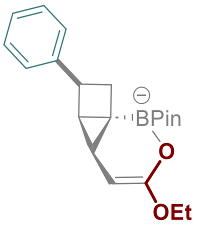

E= -1143.09171617

G_{Corr}= 0.406865

-1 1

C	0.82723200	-0.23820500	-0.06196900
H	1.11623000	-1.09674800	0.54806000
C	-0.43685900	0.53680100	0.00204700
H	-1.21272000	0.17994100	-0.69364200
C	1.60790800	0.16583200	-1.11236900
O	2.77648700	-0.48151800	-1.35027600
O	1.34607100	1.15344800	-1.92602600
C	3.54616500	-0.10439100	-2.47861600
H	2.95448900	-0.22489400	-3.40199500
H	3.82693700	0.95962800	-2.41291600
C	4.77732300	-0.98149000	-2.51469300
H	4.50159100	-2.04390900	-2.59843500
H	5.40178600	-0.71740600	-3.38118100
H	5.37949200	-0.85105000	-1.60244800
C	-1.08290900	1.52628400	0.92150400
C	-0.11412900	2.13821300	1.92430900
C	-0.23628500	2.02694400	-0.27000100
H	-2.17176300	1.51155700	1.05725700
C	0.79202100	2.56942800	0.73937600
H	-0.56023200	3.02944400	2.39565400
H	1.79692000	2.11617200	0.71586300
H	0.89864600	3.66240000	0.66772700
C	0.45679100	1.26234900	3.01310500
C	1.65596500	1.61639000	3.64765700
C	-0.19746400	0.09933000	3.43756100
C	2.18607300	0.83285100	4.67244700
H	2.18459200	2.52007400	3.33000000
C	0.32807400	-0.68746000	4.46385300
H	-1.12895600	-0.20092400	2.95027400
C	1.52323600	-0.32464600	5.08557700

H	3.12429500	1.12628600	5.15067900
H	-0.19879300	-1.59274400	4.77678300
H	1.93824800	-0.94188200	5.88620200
C	-0.38510200	3.69846600	-3.49892300
C	-0.93963100	2.29700700	-3.94394800
B	0.04470400	2.11139400	-1.85147600
O	0.48633300	3.35960500	-2.44267300
O	-0.99815300	1.60037400	-2.71732700
C	-2.32942900	2.33419100	-4.57089400
H	-2.34596100	2.97299600	-5.46826900
H	-2.63121200	1.31903100	-4.87412400
H	-3.08064600	2.70770300	-3.86143500
C	0.02341400	1.57051500	-4.89315400
H	1.04691900	1.57867900	-4.49302900
H	-0.29482200	0.51990400	-4.98188200
H	0.02846600	2.01238000	-5.90178900
C	0.38737900	4.44680200	-4.58041900
H	-0.24170100	4.63236700	-5.46563800
H	0.72289800	5.42257300	-4.19455100
H	1.27926500	3.88785700	-4.89542700
C	-1.49433100	4.60654600	-2.94970500
H	-1.02811800	5.46718500	-2.44515700
H	-2.15675500	4.99092800	-3.74079900
H	-2.10795200	4.07229700	-2.20880400

TS2 ´

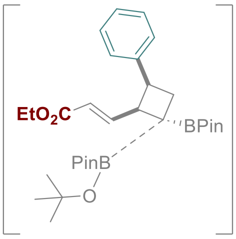

E= -1787.57195388

G_{Corr}= 0.701005

-1 1

C	-2.11561800	-0.43220900	-0.64157800
H	-3.08086500	-0.86621100	-0.36445200
C	-1.68075300	0.71304400	-0.08757200
H	-0.71621400	1.11308500	-0.41693700
C	-1.33578100	-1.14279200	-1.66803600
O	-1.97840800	-2.23251300	-2.10492100
O	-0.24584500	-0.81727300	-2.09144400
C	-1.33262500	-3.01317200	-3.11189200
H	-1.13524000	-2.37852500	-3.99048900
H	-0.35827200	-3.35989800	-2.73238200
C	-2.23510300	-4.17220000	-3.45915600
H	-3.20199800	-3.81666600	-3.84583800
H	-1.76001100	-4.79110100	-4.23433400
H	-2.42131900	-4.80488000	-2.57833800
C	-2.45015400	1.52986300	0.88465800
C	-1.69141500	2.07745200	2.15092200
C	-2.75808400	2.99423700	0.48140900
H	-3.33337800	0.93442800	1.18988900
C	-2.07774700	3.52267600	1.74248400
H	-2.20242500	1.73995100	3.06500500
H	-1.20725600	4.18664900	1.59118500
H	-2.72102500	4.02777400	2.49266900
C	-0.22700200	1.74461800	2.28723400
C	0.20893500	0.84705100	3.27181700
C	0.73546000	2.29228300	1.42215400
C	1.55698300	0.50437600	3.39630700
H	-0.52313000	0.40937100	3.95724400
C	2.08212100	1.94957900	1.54282800
H	0.42432800	2.97713900	0.62699700
C	2.50130200	1.05558800	2.53064100
H	1.86970500	-0.19559300	4.17569500

H	2.81293200	2.38501300	0.85572400
H	3.55756400	0.79092900	2.62448900
C	-1.29057700	3.62236500	-3.32184300
C	0.11906200	3.66263500	-2.60879600
C	-6.01593800	4.47907000	-0.61206900
C	-5.89195400	3.20931900	-1.53105800
B	-4.04744900	3.37921600	-0.15973100
B	-1.49944700	4.53318000	-1.21311900
O	-2.08956100	4.44788100	-2.47872800
O	-0.21979300	3.95725800	-1.25478400
O	-4.69684300	4.62523200	-0.10844600
O	-4.85111400	2.47825400	-0.89417800
C	0.90120000	2.35843400	-2.67318500
H	1.14794500	2.11091100	-3.71765100
H	1.84913900	2.47117100	-2.12421700
H	0.35609000	1.50837500	-2.24487300
C	1.01106500	4.79820800	-3.11941600
H	0.48769600	5.76457400	-3.10212500
H	1.89388000	4.87897400	-2.46724100
H	1.36172400	4.61195400	-4.14511500
C	-1.29903600	4.21232000	-4.72724500
H	-0.61625000	3.65826500	-5.38955700
H	-2.31254200	4.14141200	-5.15103600
H	-1.00653700	5.27082900	-4.72775900
C	-1.89891300	2.22254400	-3.36150800
H	-2.92976400	2.28869900	-3.73610500
H	-1.33321700	1.55307100	-4.02552200
H	-1.93259400	1.77829300	-2.36085100
C	-6.95107500	4.25294900	0.58036900
H	-6.83170200	5.08783300	1.28780900
H	-8.00842400	4.20751500	0.27876100
H	-6.69917700	3.32245700	1.11167900
C	-6.41972100	5.75446800	-1.34127700
H	-7.39834900	5.63834800	-1.83282700
H	-6.49783800	6.58589300	-0.62344800
H	-5.67611900	6.03428100	-2.09971500
C	-5.43320300	3.55532500	-2.95010800
H	-5.22756800	2.61961900	-3.49277900
H	-6.20272700	4.10748000	-3.51046600
H	-4.50301200	4.14190900	-2.92493200
C	-7.14753100	2.34864700	-1.59537300
H	-7.99814100	2.92124300	-1.99712200
H	-6.97505800	1.48786300	-2.25997300
H	-7.42409000	1.95869800	-0.60641400
C	-2.06950800	7.53601900	0.85705100
C	-1.31142700	6.84650400	-0.27727800
H	-3.15064900	7.51715900	0.65389200
H	-1.89212400	7.01009400	1.80792800
H	-1.74984900	8.58293900	0.97331500
C	0.18631000	6.81858400	0.04250600
C	-1.56729500	7.58239300	-1.59605100
H	0.75585500	6.34130300	-0.76573800
H	0.57161600	7.84029500	0.18564000
H	0.36664900	6.24801900	0.96695000
H	-1.21644800	8.62461200	-1.53918800
H	-1.04724700	7.08976700	-2.43061400
H	-2.64393900	7.58853200	-1.82520400
O	-1.84112100	5.53618200	-0.33738300

TS3´

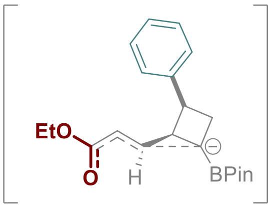

E= -1143.100212

G_{Corr}= 0.404542

-1 1

C	1.39418400	0.81168100	-0.42428600
H	2.28685400	0.45888300	0.09221800
C	0.50598000	-0.03872300	-1.02962400
H	-0.31140800	0.42537100	-1.59367100
C	1.14815900	2.24126900	-0.42441100
O	2.14236100	2.92178400	0.19165500
O	0.19249200	2.82301900	-0.91274400
C	2.01870200	4.33682600	0.25110300
H	1.95934600	4.74730800	-0.77042200
H	1.07885100	4.60551300	0.76093100
C	3.21685000	4.88437600	0.99087500
H	4.15235400	4.62519300	0.47221200
H	3.14922100	5.98064800	1.05266600
H	3.26422600	4.48345100	2.01477100
C	0.41158100	-1.51275400	-0.98644600
C	1.32407200	-2.26995400	0.00793000
C	-0.67261700	-1.51984400	0.10401100
H	0.24032500	-1.97889800	-1.97221400
C	0.36905900	-1.86614100	1.15945500
H	1.16981300	-3.34409300	-0.19153700
H	0.75906500	-0.99904500	1.73797500
H	0.14272800	-2.66354600	1.88984100
C	2.80899300	-2.02455800	0.07056800
C	3.55661700	-1.89013200	-1.10803900
C	3.48363000	-1.95044400	1.29536000
C	4.93436100	-1.68163600	-1.06476100
H	3.04673400	-1.93830600	-2.07461500
C	4.86363100	-1.74286300	1.34357200
H	2.92064600	-2.04999000	2.22735000
C	5.59482900	-1.60543800	0.16354400
H	5.49695400	-1.57340700	-1.99577400
H	5.36970800	-1.68455700	2.31079600
H	6.67402900	-1.43746400	0.19972000
C	-4.20522900	-0.51580100	0.76029100
C	-3.93258700	0.06787800	-0.67262700
B	-2.06781800	-1.02106200	0.08849500
O	-2.89037100	-0.82688400	1.21385700
O	-2.77690700	-0.65965600	-1.07623500
C	-5.05409400	-0.16181200	-1.67588800
H	-5.99081800	0.30329700	-1.33163200
H	-4.78736600	0.28919500	-2.64416700
H	-5.23430100	-1.23259900	-1.84155300
C	-3.56450600	1.55413700	-0.63862100
H	-2.76823000	1.75155800	0.09465600
H	-3.18651100	1.85032500	-1.62899900
H	-4.42928000	2.19004100	-0.39721300
C	-4.85572900	0.46187300	1.72934500
H	-5.82698900	0.80948800	1.34441600
H	-5.03163900	-0.03134800	2.69783000
H	-4.21527700	1.33607500	1.90835600
C	-5.01092800	-1.81696000	0.71885500
H	-4.97845600	-2.28706400	1.71354300
H	-6.06511300	-1.64254900	0.45635100
H	-4.58343600	-2.52669100	-0.00557200

5_{anti}

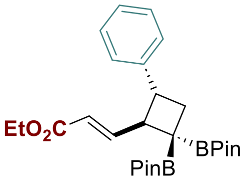

E= -1554.39184043

G_{Corr}= 0.58223

0 1

C	-2.64259800	-0.02775400	0.22564500
H	-3.64056200	0.06242100	0.66329700
C	-1.79917700	1.01056400	0.17502100

H	-0.81546900	0.85086600	-0.28294000
C	-2.25843800	-1.34465300	-0.32911700
O	-3.23175200	-2.24234100	-0.16797100
O	-1.20410900	-1.60156900	-0.86833300
C	-2.99669800	-3.56168300	-0.66931400
H	-2.78141500	-3.50267000	-1.74783400
H	-2.10448600	-3.97937700	-0.17684000
C	-4.22395300	-4.39510100	-0.39328600
H	-5.10797500	-3.97325000	-0.89445700
H	-4.06787300	-5.41622000	-0.77068500
H	-4.42677700	-4.45342200	0.68658000
C	-2.09190400	2.37800100	0.67677800
C	-0.99924400	3.07305500	1.54200500
C	-1.99749900	3.58864700	-0.31507000
H	-3.06172300	2.38777600	1.19755600
C	-1.28492800	4.39994800	0.80216400
H	-0.01388500	2.69323000	1.22941700
H	-0.40533700	4.99266700	0.51358200
H	-1.97764800	5.04677800	1.36218400
C	-1.11193100	2.98935000	3.03953600
C	-0.08697900	2.41990000	3.80507600
C	-2.25361900	3.46183600	3.70396700
C	-0.19429000	2.32536500	5.19387300
H	0.80998400	2.04478900	3.30400900
C	-2.36509200	3.36966700	5.09020600
H	-3.06896700	3.90835700	3.12757100
C	-1.33432300	2.80045800	5.84143700
H	0.61804800	1.87750300	5.77181000
H	-3.26286400	3.74412000	5.58848500
H	-1.42110800	2.72793400	6.92830600
C	-0.42843400	2.31574300	-3.50448900
C	0.29187500	3.70123200	-3.32398000
C	-5.46819100	4.14693600	-1.68739800
C	-4.87392000	5.60345000	-1.70500100
B	-3.35810200	4.13862600	-0.86290600
B	-1.14031800	3.33290000	-1.60568700
O	-1.50975000	2.41824500	-2.55539700
O	-0.00918900	4.01905600	-1.94622600
O	-4.53808600	3.45112500	-0.82900200
O	-3.47038700	5.35893200	-1.47177300
C	1.79855300	3.66415400	-3.51103100
H	2.04940600	3.32383600	-4.52686200
H	2.21573700	4.67328200	-3.37693000
H	2.28277200	2.99579500	-2.78706800
C	-0.32319000	4.80815000	-4.17767200
H	-1.41729600	4.84231500	-4.06537200
H	0.08215100	5.77748100	-3.85212300
H	-0.08356800	4.67462600	-5.24222100
C	-1.00255200	2.07202500	-4.88950300
H	-0.20363100	2.09419700	-5.64575200
H	-1.47816100	1.08059000	-4.92459900
H	-1.75842600	2.82301500	-5.15437900
C	0.43454900	1.13249400	-3.07162800
H	-0.19838400	0.23680300	-2.99164800
H	1.23356200	0.93069200	-3.79941300
H	0.89597000	1.30762800	-2.08799400
C	-5.40704400	3.46032300	-3.05032700
H	-5.64031700	2.39302700	-2.92230200
H	-6.13564200	3.89107200	-3.75175700
H	-4.40323300	3.53678900	-3.49413400
C	-6.86562300	4.03799100	-1.10241600
H	-7.57452100	4.64168600	-1.68867600
H	-7.20154500	2.99085000	-1.13412800
H	-6.89682200	4.37512900	-0.05800100
C	-5.37402400	6.46787500	-0.55008000
H	-4.77138500	7.38703300	-0.50528800
H	-6.42673800	6.75316700	-0.68790200
H	-5.27562800	5.94824300	0.41497800
C	-5.03780500	6.33905700	-3.02431000
H	-6.10399600	6.44669200	-3.27402100
H	-4.60269500	7.34641000	-2.94501300
H	-4.53505300	5.81411800	-3.84721200

int1$_{anti}$

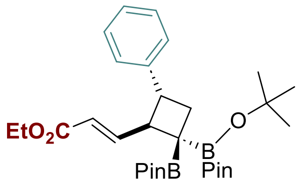

E= -1787.61371275

G_{Corr}= 0.703342

-1 1

C	-2.83753900	-0.06431800	0.23804400
H	-3.78372700	-0.01305200	0.78367500
C	-1.93418800	0.92508000	0.30839200
H	-1.01211600	0.81255800	-0.27318400
C	-2.61556200	-1.24658900	-0.61517100
O	-3.67182000	-2.06698000	-0.59166800
O	-1.62231600	-1.47694300	-1.27222700
C	-3.59985300	-3.25037400	-1.38918400
H	-3.43270300	-2.96763000	-2.44061400
H	-2.73338800	-3.85034800	-1.06857500
C	-4.89259600	-4.01070900	-1.21972000
H	-5.75116600	-3.40629400	-1.54880800
H	-4.86387700	-4.92802900	-1.82576800
H	-5.04739700	-4.29626300	-0.16839800
C	-2.08381200	2.17331000	1.09710500
C	-0.84699200	2.62147200	1.92361000
C	-2.04755300	3.56636400	0.36538700
H	-2.97784400	2.09509500	1.73215900
C	-1.05787400	4.07050400	1.45078700
H	0.06293200	2.20506700	1.45497600
H	-0.15496200	4.59867200	1.10631400
H	-1.55548900	4.69727000	2.20368800
C	-0.81508700	2.30315100	3.39594600
C	-0.32938700	3.23183100	4.32597500
C	-1.27221100	1.06600000	3.87222300
C	-0.29756900	2.93436000	5.68933200
H	0.02520200	4.20614600	3.97876100
C	-1.24378100	0.76526600	5.23387900
H	-1.66091300	0.32854000	3.16446100
C	-0.75638900	1.69965000	6.14968400
H	0.08435000	3.67479000	6.39707100
H	-1.60838700	-0.20432200	5.58302600
H	-0.73724800	1.46730700	7.21730500
C	-0.67068700	2.80561700	-3.07976400
C	-0.13781900	4.26252500	-2.82374000
C	-5.34758700	3.40997900	-1.00947800
C	-4.84214700	4.71594200	-1.74033900
B	-3.53219300	4.36467900	0.20237900
B	-1.41530600	3.57217800	-1.05774700
O	-1.68200500	2.67463000	-2.07659000
O	-0.39681700	4.43313600	-1.42500300
O	-4.69940500	3.45637800	0.23688900
O	-3.57595100	4.92960200	-1.17392800
C	1.34952700	4.44628000	-3.08520700
H	1.58851900	4.22504700	-4.13672700
H	1.63699600	5.48941800	-2.88401500
H	1.96001000	3.79726500	-2.44323100
C	-0.93764500	5.33081500	-3.56765000
H	-2.01017100	5.22495700	-3.35677900
H	-0.62131200	6.32257300	-3.21023900
H	-0.76933300	5.28831200	-4.65375600
C	-1.28591900	2.58568400	-4.45290200
H	-0.53758900	2.74799700	-5.24369400
H	-1.64932700	1.55001300	-4.53556600
H	-2.13383100	3.25942000	-4.63151300
C	0.37785100	1.72670700	-2.80216900
H	-0.11961400	0.74556700	-2.78334400
H	1.15930700	1.70425700	-3.57582400
H	0.85899200	1.87933600	-1.82404900
C	-4.92680000	2.12716600	-1.74156300

H	-5.15961200	1.26286100	-1.09956300
H	-5.46479000	1.99148100	-2.69347300
H	-3.84631200	2.12718400	-1.93975000
C	-6.86158000	3.36549700	-0.79738100
H	-7.40184300	3.38092700	-1.75789200
H	-7.13503200	2.43534000	-0.27367900
H	-7.21679900	4.20804700	-0.18886700
C	-5.73195600	5.93617000	-1.46133700
H	-5.21774600	6.83489200	-1.83714600
H	-6.71142900	5.86977200	-1.96122200
H	-5.89005700	6.06740900	-0.38439700
C	-4.70593100	4.55232300	-3.25294700
H	-5.66326800	4.25896600	-3.71336100
H	-4.39374300	5.50500100	-3.71012000
H	-3.94957500	3.79532600	-3.49541200
C	-3.63939400	6.52659900	3.28917400
C	-4.30479300	5.44067100	2.43314700
H	-2.58247000	6.27682600	3.47571700
H	-3.67177200	7.49379400	2.76274400
H	-4.14072600	6.64486200	4.26339000
C	-5.79203700	5.78657200	2.25388600
C	-4.20585200	4.09510100	3.17031400
H	-6.27057100	5.02567800	1.62231200
H	-6.31991700	5.82777100	3.22097700
H	-5.90109200	6.76722100	1.76380700
H	-4.72142600	4.14877600	4.14289400
H	-4.66734500	3.30177600	2.56706400
H	-3.15791400	3.82429100	3.36284700
O	-3.64811800	5.45461900	1.19494800

C	-1.30758400	2.64861900	5.46197700
H	-1.52586900	1.52489000	3.63629300
C	-0.67852400	3.71867500	6.10123100
H	0.73410800	5.33842400	5.87946400
H	-2.02431300	2.02964500	6.00808700
H	-0.89973200	3.94373200	7.14746200
C	-1.58839700	2.81296000	-3.46744300
C	-0.69962500	4.09559000	-3.65932800
B	-0.75280800	3.47265500	-1.43775100
O	-1.85026200	2.84094100	-2.07410600
O	-0.01107000	4.17871000	-2.41789700
C	0.31263400	3.99733700	-4.79332700
H	-0.18982300	3.82003400	-5.75715200
H	0.87911000	4.93818300	-4.87446700
H	1.03242100	3.18599300	-4.61852200
C	-1.53803400	5.36660700	-3.82428400
H	-2.30548500	5.43905700	-3.03875900
H	-0.87708700	6.24228300	-3.73390500
H	-2.03598100	5.41319300	-4.80460900
C	-2.90401200	2.83038100	-4.23455300
H	-2.72864100	2.91222700	-5.31863600
H	-3.45530100	1.89515400	-4.04993200
H	-3.54454800	3.66506600	-3.91892900
C	-0.83507400	1.51408700	-3.77770800
H	-1.45211900	0.66581400	-3.44389900
H	-0.63559300	1.39077000	-4.85286600
H	0.12224600	1.46802900	-3.23595500

int2$_{anti}$

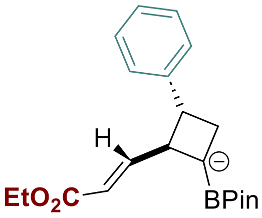

E= -1143.10261961
G_{Corr}= 0.403153

-1 1

C	-1.82569900	0.27637600	-0.11169000
H	-2.86481100	0.60981700	-0.05710500
C	-0.82489900	0.99800900	0.43210000
H	0.19970100	0.62615000	0.30121100
C	-1.54276900	-0.91084300	-0.92335500
O	-2.65351900	-1.37541500	-1.52139000
O	-0.45787400	-1.43503100	-1.07923100
C	-2.51015900	-2.51277400	-2.36946400
H	-1.75219000	-2.29987900	-3.14004800
H	-2.14285700	-3.36634600	-1.77682000
C	-3.85507200	-2.81041100	-2.98783700
H	-4.20813900	-1.95935700	-3.58971100
H	-3.77553300	-3.68848500	-3.64546800
H	-4.60648600	-3.02614700	-2.21334300
C	-0.95393500	2.34908900	1.03230300
C	0.21035600	2.80161900	1.96387100
C	-0.44707900	3.36888500	0.00161200
H	-1.96329000	2.49733000	1.46800000
C	0.59271900	3.92664600	0.96664600
H	0.99297000	2.02333800	1.97933600
H	1.65994600	3.92877200	0.66613900
H	0.38541200	4.94139300	1.36726000
C	-0.11413500	3.14355600	3.39644700
C	0.51123100	4.21262800	4.05215400
C	-1.02681600	2.36624000	4.12539300
C	0.23404100	4.49838700	5.39026300
H	1.22861200	4.83325700	3.50866200

29enolate$_{anti}$

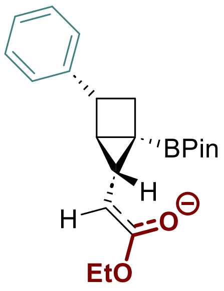

E= -1143.11424256
G_{Corr}= 0.405819

-1 1

C	-0.87586900	-0.67497700	-0.15202600
H	-1.51596500	-1.33370600	0.44393900
C	0.00494700	0.30236200	0.53779600
H	0.99542900	-0.05039700	0.88598100
C	-0.87802900	-0.81290800	-1.53295500
O	-1.76477200	-1.79335000	-1.98496800
O	-0.21336400	-0.18360000	-2.38451000
C	-1.78198800	-2.05559000	-3.36542100
H	-2.15898900	-1.18360400	-3.93033600
H	-0.76134200	-2.24841000	-3.74103000
C	-2.67424800	-3.25531600	-3.60827300
H	-3.69909800	-3.05950300	-3.25567400
H	-2.71951900	-3.48996800	-4.68286000
H	-2.29377100	-4.14175700	-3.07700100
C	-0.61143000	1.35527500	1.41209500

C	0.43443700	2.15299300	2.19850700
C	0.02535200	1.75233100	0.07672600
H	-1.67935500	1.33338700	1.65233000
C	1.14126600	2.48189400	0.84282300
H	1.03752800	1.55632700	2.90316900
H	2.15549000	2.06498100	0.72202300
H	1.17976300	3.56292400	0.64512500
C	-0.10312300	3.36813100	2.91098300
C	0.34912300	3.70892500	4.19135800
C	-1.05449000	4.20278600	2.30112500
C	-0.12442700	4.84958600	4.84366300
H	1.08569200	3.06947500	4.68639800
C	-1.52916700	5.34173200	2.94874400
H	-1.42692600	3.95512100	1.30315900
C	-1.06517900	5.67157900	4.22447400
H	0.24453000	5.09496300	5.84290800
H	-2.26915700	5.97646200	2.45439000
H	-1.43825400	6.56398000	4.73297600
C	-2.24420100	2.95504400	-2.69744200
C	-0.92018100	3.77965900	-2.92874000
B	-0.65279400	2.31650500	-1.20085400
O	-2.00942600	2.35569600	-1.41309300
O	0.03400800	3.07318900	-2.12236000
C	-0.43515700	3.80338900	-4.37026400
H	-1.18716700	4.26768600	-5.02620200
H	0.49047700	4.39443000	-4.44180800
H	-0.22271200	2.79194200	-4.74060800
C	-0.99537600	5.20517200	-2.38095400
H	-1.34279800	5.21561300	-1.33685000
H	0.01097400	5.64947800	-2.40652900
H	-1.66599000	5.83849000	-2.97971500
C	-3.50780500	3.79948300	-2.62433000
H	-3.66689700	4.34188200	-3.56879700
H	-4.37881400	3.14830300	-2.45603600
H	-3.46644600	4.52840200	-1.80392200
C	-2.42824200	1.81868100	-3.70153300
H	-3.28494500	1.20517600	-3.38189000
H	-2.63705700	2.19798500	-4.71265000
H	-1.54192000	1.16784300	-3.71089400

29 **B-O-anti**

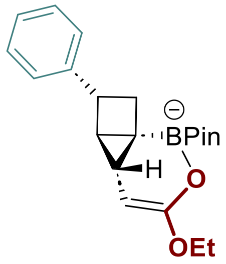

E= -1143.12917868

G_{Corr}= 0.408092

-1 1

C	-0.11617800	-0.98015200	-0.26481300
H	-0.06797700	-1.96878700	0.19731400
C	0.58518400	0.17985600	0.34623700
H	1.61974100	-0.00026800	0.68000900
C	-0.87995700	-0.83328400	-1.38021200
O	-1.49606300	-1.93114500	-1.88715700
O	-1.09276400	0.26452700	-2.05227200
C	-2.42125000	-1.76193000	-2.94809300
H	-3.21335900	-1.05226400	-2.65505400
H	-1.91777100	-1.33707300	-3.83212500
C	-3.00957900	-3.11704400	-3.27023400
H	-3.53129700	-3.53770000	-2.39702900
H	-3.73284400	-3.02580700	-4.09423700
H	-2.22348400	-3.82363700	-3.57773200
C	-0.23136700	1.15975600	1.15493800

C	0.61502800	2.27733600	1.77134100
C	0.24297400	1.51729600	-0.25931000
H	-1.25020800	0.91159400	1.47409700
C	1.18437000	2.56747800	0.34542300
H	1.35584600	1.95589100	2.52398400
H	2.26319200	2.37312000	0.21538300
H	0.97779700	3.59542300	0.01751100
C	-0.20211600	3.41558400	2.33163600
C	0.08244200	3.96075900	3.58878900
C	-1.26811900	3.96117400	1.59322400
C	-0.66628200	5.02455900	4.09984100
H	0.90510400	3.54649800	4.17912000
C	-2.01505700	5.02154000	2.10305700
H	-1.51019400	3.54633000	0.60702300
C	-1.71840700	5.55976800	3.35861300
H	-0.42407400	5.43466600	5.08385000
H	-2.84034600	5.43254000	1.51498500
H	-2.30662300	6.39080400	3.75593000
C	-2.06619600	3.21579200	-2.60534300
C	-0.61374800	3.42286800	-3.17658600
B	-0.63297100	1.66849500	-1.60331300
O	-1.81688000	2.51175600	-1.41020900
O	0.06947000	2.27156200	-2.73406900
C	-0.54154700	3.50423800	-4.69946800
H	-1.15126200	4.33610200	-5.08706100
H	0.49995400	3.67406900	-5.01618200
H	-0.88485900	2.57114500	-5.16689900
C	0.06237600	4.66470400	-2.57969900
H	-0.05824100	4.69152000	-1.48706600
H	1.14026000	4.62254500	-2.80122000
H	-0.33779400	5.60241900	-2.99574300
C	-2.81034800	4.50862200	-2.28249300
H	-2.93160400	5.13565900	-3.18017200
H	-3.81528200	4.27369600	-1.89707600
H	-2.28787700	5.09671200	-1.51556000
C	-2.94048400	2.35991100	-3.53446200
H	-3.85121600	2.06640100	-2.98880000
H	-3.24632600	2.90807400	-4.43919600
H	-2.41862600	1.44034200	-3.83244700

TS1$_{anti}$

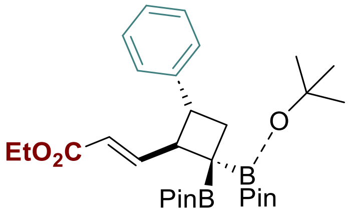

E= -1787.57576676

G_{Corr}= 0.699544

-1 1

C	-2.79068000	-0.00096100	0.35969800
H	-3.75678100	0.16887300	0.84310500
C	-1.86142800	0.96273200	0.29248300
H	-0.91447000	0.72606300	-0.20831000
C	-2.55049100	-1.33431600	-0.23030700
O	-3.58856800	-2.14981300	-0.02816800
O	-1.55201300	-1.67526000	-0.82753600
C	-3.49240800	-3.47655700	-0.55241500
H	-3.32779300	-3.42318200	-1.64019400
H	-2.61546400	-3.97573400	-0.11061900
C	-4.77080700	-4.20664300	-0.22037800
H	-5.63973600	-3.70577300	-0.67286900
H	-4.72147700	-5.23318400	-0.61196600
H	-4.92369200	-4.25934600	0.86799300
C	-2.02172100	2.33803500	0.82578900
C	-0.84010700	2.97951900	1.59559500
C	-1.95651500	3.55793500	-0.15687000

H	-2.94132300	2.44052000	1.41674300
C	-1.17548700	4.33370300	0.93721100
H	0.10679000	2.59209100	1.17579600
H	-0.32939900	4.96100200	0.61973000
H	-1.89182100	4.88871800	1.56139200
C	-0.79963500	2.87272100	3.09603100
C	-0.22394100	3.89433800	3.86318300
C	-1.34428900	1.76809500	3.76309400
C	-0.19630200	3.81794400	5.25540000
H	0.19804000	4.77001700	3.36202300
C	-1.32132800	1.68863800	5.15618500
H	-1.80338100	0.96179100	3.18517400
C	-0.74954000	2.71508200	5.90868500
H	0.25226700	4.62893900	5.83495600
H	-1.75971800	0.82146300	5.65673700
H	-0.73665500	2.65724200	6.99983800
C	-0.47240300	2.28365000	-3.39091900
C	0.24942700	3.67014200	-3.22973400
C	-5.35411000	4.17110200	-1.68689100
C	-4.79464200	5.64173600	-1.52252600
B	-3.34188800	4.14086000	-0.62251700
B	-1.15519800	3.30582400	-1.47933400
O	-1.53540300	2.38875500	-2.42718500
O	-0.02909300	3.99391500	-1.85264000
O	-4.45566700	3.38825700	-0.89170300
O	-3.42614800	5.40765200	-1.15642200
C	1.75347400	3.63288100	-3.44190400
H	1.98925900	3.28719400	-4.45966900
H	2.17254500	4.64285400	-3.31959200
H	2.24966700	2.96901000	-2.72179700
C	-0.37812600	4.77123600	-4.08260700
H	-1.47024000	4.80544300	-3.95368300
H	0.03072300	5.74298600	-3.76851800
H	-0.15476000	4.63215800	-5.15010800
C	-1.06677000	2.03031400	-4.76627300
H	-0.27945500	2.04450100	-5.53498200
H	-1.54561400	1.03989400	-4.78677100
H	-1.82502900	2.78058200	-5.02633700
C	0.40035600	1.10319900	-2.96717500
H	-0.23062700	0.20693800	-2.87706000
H	1.19049400	0.89934100	-3.70419700
H	0.87351500	1.28242800	-1.98978100
C	-5.24851500	3.64131400	-3.11739000
H	-5.48804000	2.56725600	-3.11635700
H	-5.94725100	4.14939800	-3.79797900
H	-4.22708900	3.75606600	-3.50882800
C	-6.76992800	3.97796500	-1.16457900
H	-7.48114800	4.61600700	-1.71124600
H	-7.07665600	2.92981200	-1.30206200
H	-6.83334600	4.21527100	-0.09471400
C	-5.42598000	6.41081100	-0.36332500
H	-4.94798700	7.40185500	-0.30030700
H	-6.50616100	6.56682900	-0.50310400
H	-5.20776000	5.86830300	0.57514600
C	-4.83105800	6.47132400	-2.79837300
H	-5.86910500	6.61993800	-3.13376000
H	-4.39255300	7.46219300	-2.60615300
H	-4.26411200	6.00337800	-3.61433900
C	-4.01295800	4.67607200	4.28459400
C	-4.79683100	4.32048200	2.99095300
H	-3.05949600	4.12386400	4.29846000
H	-3.77247900	5.75316100	4.27861900
H	-4.55728300	4.44798200	5.21977400
C	-6.15522900	5.07697000	3.05215800
C	-5.12179200	2.80081800	3.05410800
H	-6.77341600	4.81511800	2.17664900
H	-6.74556000	4.85699400	3.96050600
H	-5.97645000	6.16549300	3.01833700
H	-5.75452000	2.51884800	3.91574200
H	-5.64202100	2.49316800	2.13120200
H	-4.18881400	2.21721500	3.12089000
O	-4.08549100	4.65036900	1.88809600

TS2$_{anti}$

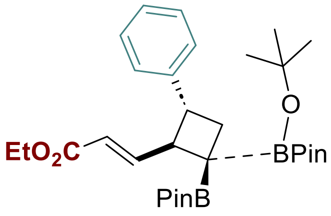

E= -1787.58431417
G_{Corr}= 0.700324

-1 1

C	-2.58865600	-0.34208600	0.02168300
H	-3.62362300	-0.29511700	0.37007600
C	-1.71115400	0.64342500	0.27965200
H	-0.68995100	0.52411800	-0.10642300
C	-2.19650200	-1.52992500	-0.75543300
O	-3.20758800	-2.40257900	-0.85762200
O	-1.10946000	-1.72993700	-1.25644600
C	-2.96440100	-3.60214000	-1.59308800
H	-2.65750100	-3.34256500	-2.61879900
H	-2.12796800	-4.14875000	-1.12903200
C	-4.23131600	-4.42285200	-1.58671600
H	-5.05889500	-3.87417400	-2.06092900
H	-4.07032700	-5.35582500	-2.14648100
H	-4.52702800	-4.68359300	-0.55937600
C	-1.97537800	1.91243600	1.00454400
C	-0.92850900	2.25532700	2.11067300
C	-1.55046400	3.21404300	0.29750700
H	-3.01255500	1.91128200	1.39537900
C	-0.75241900	3.67110300	1.51530200
H	-0.01392100	1.66436800	1.92778000
H	0.29890000	3.98731000	1.37124300
H	-1.24551300	4.45327900	2.12392200
C	-1.30530900	2.09146000	3.56065900
C	-0.73500000	2.92285200	4.53554900
C	-2.22885400	1.12350500	3.97615500
C	-1.08298900	2.79962100	5.88021000
H	-0.01647300	3.68957200	4.23196100
C	-2.58162100	0.99739100	5.32093300
H	-2.68634600	0.46262900	3.23527300
C	-2.01340100	1.83777700	6.27892500
H	-0.63024900	3.46331300	6.62159500
H	-3.30974200	0.23935000	5.62139900
H	-2.29399200	1.74440100	7.33094900
C	-0.81668900	2.90817700	-3.33170800
C	-0.24066600	4.33352900	-3.01294100
C	-5.41432200	3.47387800	-0.67575000
C	-5.01914900	4.66768700	-1.62917000
B	-3.76298100	4.74997500	0.28061500
B	-1.19107500	3.41258500	-1.12515100
O	-1.69676300	2.69298300	-2.23596800
O	-0.22851600	4.33783100	-1.59170000
O	-4.76640500	3.82395700	0.54736700
O	-3.80571100	5.15340200	-1.05523500
C	1.17042500	4.57366500	-3.53401000
H	1.20778200	4.47370400	-4.63017000
H	1.49602900	5.59339800	-3.27601700
H	1.89015300	3.87003200	-3.09387600
C	-1.16614000	5.46006500	-3.47858100
H	-2.18281900	5.30370100	-3.09185500
H	-0.79567900	6.41238200	-3.06805000
H	-1.19812800	5.55076600	-4.57502800
C	-1.57978700	2.80838000	-4.64490500
H	-0.91656400	3.02424600	-5.49710700
H	-1.97307000	1.78760200	-4.77124600
H	-2.42715500	3.50454200	-4.68161600
C	0.25958700	1.81758800	-3.28407700
H	-0.23388900	0.83397600	-3.27770600
H	0.93562900	1.85901700	-4.15130200

H	0.86383200	1.89626600	-2.36732000
C	-4.87210900	2.12577400	-1.14398800
H	-5.01931300	1.39210100	-0.33811400
H	-5.41148200	1.76406700	-2.03236600
H	-3.80132900	2.17964700	-1.38202400
C	-6.91040900	3.35145500	-0.41147400
H	-7.46011200	3.17440500	-1.34856800
H	-7.09833800	2.49758500	0.25745200
H	-7.31722400	4.25185700	0.06816400
C	-6.02660400	5.81999600	-1.59567300
H	-5.59775700	6.67845100	-2.13446400
H	-6.97735500	5.55136100	-2.07961100
H	-6.23704900	6.14040500	-0.56454900
C	-4.77199500	4.24096300	-3.06778700
H	-5.66472100	3.75399400	-3.48988600
H	-4.54214600	5.12037200	-3.68914300
H	-3.92204100	3.55022900	-3.11711900
C	-2.88801000	6.81113100	3.21612700
C	-3.83702800	5.83789200	2.51935100
H	-1.90007600	6.34608300	3.35867500
H	-2.75685600	7.72043000	2.60954400
H	-3.27782300	7.10316100	4.20296200
C	-5.20790400	6.49054000	2.31719400
C	-3.97197400	4.56151300	3.35298800
H	-5.89244400	5.79418700	1.81102400
H	-5.65415100	6.77235900	3.28336600
H	-5.11374200	7.39882000	1.70167000
H	-4.35927900	4.80255200	4.35534000
H	-4.65492400	3.84954400	2.87193000
H	-2.99377700	4.07783400	3.47387600
O	-3.24636700	5.58075300	1.24619700

TS3$_{anti}$

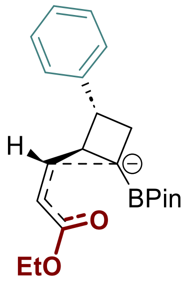

E= -1143.10153474
G_{Corr}= 0.404786

-1 1

C	-1.33654500	-0.05248500	-0.23467200
H	-2.40168800	0.06539100	-0.02292600
C	-0.39648100	0.76613200	0.38251300
H	0.66359500	0.53018900	0.23992900
C	-0.95683500	-0.90777700	-1.31588600
O	-2.02124300	-1.57980400	-1.84728400
O	0.16994100	-1.07464600	-1.77360200
C	-1.76022400	-2.41445800	-2.96147700
H	-1.27792500	-1.83053300	-3.76372300
H	-1.05213100	-3.21190600	-2.67874300
C	-3.07263600	-2.99882900	-3.43226900
H	-3.76910200	-2.20464500	-3.74251800
H	-2.90240200	-3.66273400	-4.29303600
H	-3.55135100	-3.58600100	-2.63360800
C	-0.70590600	1.89524600	1.26056100
C	0.49522800	2.41985100	2.08673800
C	-0.28103600	2.79302600	0.09856300
H	-1.71047200	1.90821100	1.71457400
C	1.02248300	3.14136600	0.80581800
H	1.15183500	1.62520400	2.47304800
H	1.94708900	2.68740900	0.39747900
H	1.23326800	4.21589600	0.96069900

C	0.11237800	3.33187500	3.22298700
C	0.59041600	3.09994300	4.51877700
C	-0.73724200	4.43194100	3.01935700
C	0.23614800	3.93615700	5.58001000
H	1.25213200	2.24810200	4.70042400
C	-1.09584900	5.26651100	4.07623700
H	-1.12203100	4.63083200	2.01484400
C	-0.60969100	5.02302000	5.36313600
H	0.62412200	3.73448000	6.58184500
H	-1.76012000	6.11536500	3.89431900
H	-0.88971400	5.67837100	6.19157200
C	-2.15295200	2.83358500	-3.10547800
C	-1.07086100	3.94369000	-3.36131800
B	-0.85376400	3.08301900	-1.24784200
O	-2.13162900	2.72328800	-1.68244400
O	-0.16268500	3.73424100	-2.27870200
C	-0.32321700	3.80558700	-4.67966200
H	-1.01970900	3.85046200	-5.53116800
H	0.40003100	4.62837100	-4.78881300
H	0.23187300	2.85920900	-4.72937800
C	-1.63774300	5.35895400	-3.23007300
H	-2.21250800	5.47274400	-2.29846200
H	-0.80324400	6.07602600	-3.20380400
H	-2.28915500	5.62175300	-4.07680100
C	-3.55800400	3.19632400	-3.56292300
H	-3.57854900	3.39329900	-4.64588600
H	-4.24375700	2.36018600	-3.35685100
H	-3.93882400	4.08251900	-3.03753100
C	-1.75362700	1.47333200	-3.68569800
H	-2.43712800	0.70598500	-3.29343200
H	-1.81102500	1.46160700	-4.78442600
H	-0.73391100	1.19129600	-3.38282800

int1 ′$_{anti}$

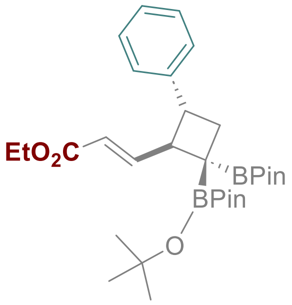

E= -1787.60763187
G_{Corr}= 0.70321

-1 1

C	-2.20829300	-0.42558000	-0.85396600
H	-3.19859900	-0.74367500	-0.51444100
C	-1.66488100	0.73470400	-0.45360800
H	-0.67887200	1.02777900	-0.82258800
C	-1.50335200	-1.32023000	-1.79051400
O	-2.20102900	-2.43886600	-2.01604500
O	-0.43054600	-1.10386200	-2.31427000
C	-1.63618400	-3.39346500	-2.91695300
H	-1.46793000	-2.91261100	-3.89363500
H	-0.65393700	-3.71403100	-2.53493000
C	-2.59193200	-4.55617200	-3.02989900
H	-3.56737700	-4.22806200	-3.41926600
H	-2.17991500	-5.30712000	-3.71977400
H	-2.74799700	-5.03497100	-2.05155800
C	-2.31146500	1.68625700	0.48066700
C	-1.51652300	2.07068500	1.75679500
C	-2.47545000	3.22844300	0.18376000
H	-3.28695800	1.26801400	0.78598400
C	-2.17043900	3.46517900	1.69095200
H	-0.45485500	2.16503900	1.48249600
H	-1.51137300	4.30980100	1.92824000

H	-3.07094700	3.52841800	2.32758300
C	-1.64385700	1.19507800	2.97309700
C	-0.52117000	0.56337200	3.52433200
C	-2.89079500	0.96545300	3.57525500
C	-0.63469500	-0.26832100	4.64011900
H	0.45999400	0.72738600	3.06923900
C	-3.01021600	0.13592900	4.68920800
H	-3.78442700	1.44358700	3.16397300
C	-1.88083100	-0.48506500	5.22775700
H	0.25643800	-0.74905800	5.05200000
H	-3.99193600	-0.02755300	5.14133100
H	-1.97325400	-1.13522500	6.10125500
C	-1.12570500	3.61475700	-3.18717500
C	0.23957600	3.38908100	-2.42299400
C	-5.91123300	4.58640000	-0.65793500
C	-5.75552000	3.38588100	-1.66038700
B	-3.88737800	3.55830900	-0.36784100
B	-1.38428600	3.99960800	-0.84540800
O	-1.96870200	4.15751500	-2.20454700
O	-0.17400100	3.18772800	-1.09479000
O	-4.57444100	4.73822900	-0.16293400
O	-4.64993400	2.66763400	-1.09670600
C	1.02699500	2.16824900	-2.89959200
H	1.29752600	2.25969200	-3.96419200
H	1.96336200	2.08657600	-2.32367100
H	0.46900500	1.23200200	-2.76192200
C	1.16432900	4.61502300	-2.50140200
H	0.63025700	5.52845700	-2.21291200
H	1.99086100	4.47480800	-1.78659100
H	1.59909900	4.74764800	-3.50477500
C	-1.02204900	4.57937200	-4.37049400
H	-0.31846600	4.20861200	-5.13373600
H	-2.00887100	4.68812900	-4.84884500
H	-0.69323700	5.57825300	-4.05353200
C	-1.73858100	2.30152000	-3.69282000
H	-2.76039800	2.50144100	-4.04944100
H	-1.16742900	1.86045500	-4.52499800
H	-1.80509700	1.56097000	-2.88536400
C	-6.80048100	4.25400500	0.53986600
H	-6.69897900	5.05084800	1.29188300
H	-7.86030800	4.18408400	0.25458500
H	-6.50056800	3.30466200	1.00891100
C	-6.35987300	5.89300200	-1.29405700
H	-7.35276800	5.77918600	-1.75547100
H	-6.42988200	6.67689400	-0.52462600
H	-5.65333700	6.23363900	-2.06227200
C	-5.33756000	3.83462900	-3.06088500
H	-5.10353400	2.94319400	-3.66214800
H	-6.14023600	4.38823400	-3.56948100
H	-4.42890800	4.45271700	-3.01372300
C	-6.96427900	2.46677500	-1.74182500
H	-7.85094000	3.02097500	-2.08582200
H	-6.76830600	1.65824100	-2.46221300
H	-7.19284700	2.00704200	-0.77102400
C	-2.30957100	6.78067400	1.04572600
C	-1.47061500	6.57849100	-0.22903900
H	-3.16771500	6.09515300	1.04699300
H	-1.69827800	6.59219500	1.94276700
H	-2.69278600	7.81310700	1.10901100
C	-0.27480300	7.54333200	-0.18167900
C	-2.33860000	6.92952400	-1.44675600
H	0.31455800	7.47520300	-1.10992000
H	-0.59561200	8.59024700	-0.05551500
H	0.38662100	7.27993500	0.65918900
H	-2.67177100	7.97913600	-1.38167000
H	-1.77637200	6.79624600	-2.38145800
H	-3.22070400	6.27902200	-1.48710100
O	-0.92717400	5.29104000	-0.27139800

int2 ´anti

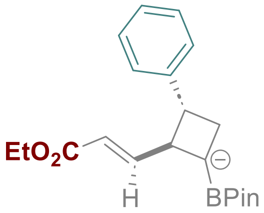

E= -1143.10174959

G_{Corr}= 0.40335

-1 1

C	1.75794800	0.41559700	-0.37002400
H	2.32525200	0.67382100	0.52715500
C	0.47044000	0.78961600	-0.55050400
H	-0.01994700	0.45817800	-1.47406900
C	2.46443000	-0.34573000	-1.40330900
O	3.71571000	-0.65387200	-1.01951100
O	2.01904300	-0.68514400	-2.48239200
C	4.51494600	-1.39631300	-1.93740600
H	4.02121100	-2.35634200	-2.15881100
H	4.59052800	-0.84260400	-2.88711800
C	5.87418400	-1.60794200	-1.31461400
H	5.79286900	-2.16928000	-0.37156000
H	6.51284500	-2.18089000	-2.00289600
H	6.36655400	-0.64585400	-1.10724700
C	-0.36442600	1.70646800	0.26728400
C	0.34191500	2.45763400	1.43271000
C	-0.35831000	3.01375400	-0.54314200
H	-1.33112400	1.23574800	0.53826500
C	0.55314300	3.67612500	0.48226200
H	1.27000400	1.98625400	1.78868500
H	1.61891100	3.80649700	0.19579800
H	0.23000700	4.63948600	0.92520500
C	-0.54194000	2.71699300	2.62517100
C	-1.79957600	3.32553100	2.47849400
C	-0.13593700	2.34964000	3.91448100
C	-2.61770400	3.55891600	3.58208700
H	-2.13269600	3.61669200	1.47728600
C	-0.95256100	2.58220300	5.02375700
H	0.83879400	1.87250700	4.05263500
C	-2.19782000	3.18817300	4.86219900
H	-3.59247300	4.03391400	3.44294700
H	-0.61166600	2.28648200	6.01944400
H	-2.83931200	3.37130300	5.72779900
C	-1.17258400	4.31957100	-3.94210700
C	-1.43071200	2.77622600	-4.08349600
B	-0.75498900	3.25896200	-1.94405800
O	-0.43723100	4.39177700	-2.72587400
O	-1.52760700	2.36481200	-2.72658200
C	-2.71305800	2.41184700	-4.81938000
H	-2.70828000	2.81555300	-5.84385900
H	-2.80669600	1.31686800	-4.88704900
H	-3.60025300	2.79329500	-4.29591800
C	-0.24563800	2.04095600	-4.71778500
H	0.69987200	2.30467000	-4.21967900
H	-0.39348200	0.95674200	-4.59880800
H	-0.14773000	2.25780700	-5.79213100
C	-0.34907300	4.93089200	-5.06774100
H	-0.84458500	4.78910100	-6.04084600
H	-0.23191500	6.01304900	-4.90144100
H	0.65482600	4.48775600	-5.11714000
C	-2.46970900	5.11343200	-3.76094500
H	-2.21842600	6.13875100	-3.44911000
H	-3.05427200	5.17163100	-4.69142400
H	-3.10224900	4.66679500	-2.97874600

29enolate ´anti

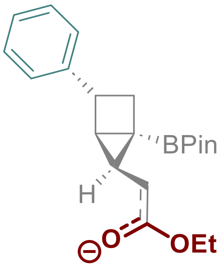

E= -1143.11597393
G_{Corr}= 0.404553

-1 1

C	1.83523100	0.13757100	-0.35558400
H	2.19884500	0.00741000	0.66706400
C	0.48000900	0.66014300	-0.62293900
H	-0.04923200	0.16298300	-1.44755200
C	2.67266000	-0.15712300	-1.42962000
O	3.91375700	-0.66559700	-1.04007200
O	2.43945500	-0.03649400	-2.64643900
C	4.81213800	-0.99086600	-2.07069900
H	4.38473400	-1.76390300	-2.73541200
H	5.00878900	-0.11257100	-2.71190700
C	6.09809500	-1.48672800	-1.44325100
H	5.91492400	-2.37923400	-0.82462300
H	6.82843800	-1.75245900	-2.22286100
H	6.54584800	-0.71182600	-0.80149100
C	-0.47601500	1.18459400	0.38399800
C	0.22147800	1.95137300	1.52039200
C	0.08238900	2.17404100	-0.65107800
H	-1.51029000	0.83072400	0.45697300
C	0.85681800	2.89629900	0.45076800
H	0.94652100	1.35527600	2.09661100
H	1.95534500	2.83489500	0.36988900
H	0.56433800	3.94726500	0.58921900
C	-0.71856800	2.63702500	2.47888200
C	-1.82245500	3.37119200	2.01520000
C	-0.50785600	2.56534900	3.86121600
C	-2.68153400	4.01339500	2.90491100
H	-2.01023300	3.44214500	0.93995000
C	-1.36597200	3.20817200	4.75623900
H	0.34365300	1.99490700	4.24321300
C	-2.45674700	3.93524000	4.28153000
H	-3.53453400	4.57911100	2.52132100
H	-1.17959800	3.13788600	5.83100700
H	-3.13081900	4.43792400	4.97942400
C	-1.07567000	4.16240000	-3.62135600
C	-1.65027700	2.72774100	-3.91276100
B	-0.56293400	2.74080500	-1.92160600
O	-0.17311000	3.91002200	-2.52826800
O	-1.55973000	2.10020600	-2.62096600
C	-3.09529900	2.70623500	-4.38308300
H	-3.20319100	3.26814200	-5.32316600
H	-3.41108900	1.66862800	-4.56837400
H	-3.77277700	3.13904600	-3.63521500
C	-0.77277100	1.91521600	-4.86486300
H	0.27706000	1.90663800	-4.53503500
H	-1.12902900	0.87435800	-4.87468600
H	-0.81772700	2.30373400	-5.89244600
C	-0.30200900	4.78079300	-4.77438900
H	-0.94741500	4.88643200	-5.65951400
H	0.05364900	5.78262800	-4.49036800
H	0.57163900	4.17376100	-5.04628600
C	-2.13670800	5.13770300	-3.11410900
H	-1.63804900	6.04237100	-2.73559400
H	-2.82826300	5.43603700	-3.91523900
H	-2.72195700	4.70153600	-2.29051800

$29_{B\text{-}O\text{-}anti}$´

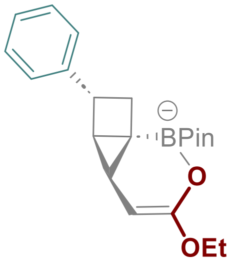

E= -1143.09200061
G_{Corr}= 0.406425

-1 1

C	1.09383400	-0.36945200	-0.27756500
H	1.45993900	-1.23663300	0.27676600
C	-0.25428900	0.24719400	-0.21379300
H	-0.96466700	-0.14963500	-0.95674600
C	1.84987900	0.18596900	-1.27670200
O	3.08933800	-0.31017700	-1.51440700
O	1.49693400	1.18451900	-2.04110100
C	3.84095300	0.21744800	-2.59398000
H	3.29218500	0.08455600	-3.54169000
H	3.99565600	1.30065100	-2.46002400
C	5.16487200	-0.51174200	-2.63659100
H	5.01470400	-1.59170400	-2.78782700
H	5.77831700	-0.12887800	-3.46578000
H	5.72321500	-0.36773000	-1.69887900
C	-1.02781200	1.09657700	0.74249200
C	-0.14903900	1.72498400	1.82470900
C	-0.21606400	1.76507800	-0.38894500
H	-2.11164600	0.95841600	0.85316100
C	0.70672700	2.35700700	0.68976500
H	0.38949400	0.98350900	2.43686000
H	1.76881000	2.06091200	0.66506200
H	0.64899700	3.45659100	0.70319300
C	-0.84041900	2.70673700	2.73433900
C	-1.70195000	3.68879700	2.21874700
C	-0.63271000	2.67371900	4.11883400
C	-2.32985000	4.60581100	3.05960700
H	-1.88148200	3.73497700	1.14079600
C	-1.25792600	3.59243400	4.96490500
H	0.03165700	1.91441300	4.54154200
C	-2.10982700	4.56265700	4.43854000
H	-2.99692600	5.36096900	2.63575000
H	-1.07764400	3.54727100	6.04210000
H	-2.60154100	5.28203900	5.09801100
C	-0.49377700	3.66933400	-3.43792600
C	-0.88125700	2.26804200	-4.03321100
B	0.10265600	1.99054300	-1.94786600
O	0.42331900	3.32247300	-2.42459400
O	-0.85514900	1.44334900	-2.88724200
C	-2.26685200	2.20896400	-4.66759300
H	-2.36033700	2.93388200	-5.49186500
H	-2.44794800	1.20377400	-5.08038500
H	-3.05512500	2.41351900	-3.93000000
C	0.15969000	1.76367500	-5.04178200
H	1.17532700	1.85047400	-4.63060000
H	-0.03219400	0.69822900	-5.24395100
H	0.11328400	2.30707100	-5.99843600
C	0.17226800	4.61782100	-4.42894300
H	-0.48328800	4.82072300	-5.29080200
H	0.39198300	5.57934500	-3.93826800
H	1.12160100	4.20749500	-4.79969700
C	-1.69809600	4.37203100	-2.79471900
H	-1.33240700	5.22053000	-2.19555200
H	-2.40925800	4.76025100	-3.54027400
H	-2.23670700	3.68924300	-2.12050200

TS1 ´$_{anti}$

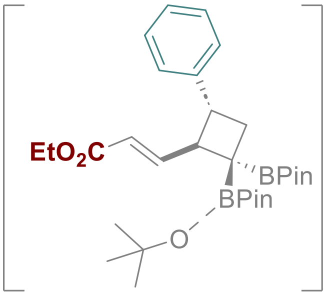

E= -1787.57090802

G_{Corr}= 0.699683

-1 1

C	-2.46747700	-0.60358300	-0.80898000
H	-3.47582000	-0.82036000	-0.44513300
C	-1.79170400	0.48436800	-0.41600400
H	-0.78826200	0.64652200	-0.82304100
C	-1.87533200	-1.55589900	-1.77330900
O	-2.68538500	-2.59447800	-1.99049900
O	-0.79840100	-1.43448400	-2.31580000
C	-2.23987500	-3.59238300	-2.91311100
H	-2.05856300	-3.12147000	-3.89221500
H	-1.28040100	-4.00420500	-2.56258400
C	-3.30275200	-4.66017000	-2.99975500
H	-4.25483100	-4.24106700	-3.35830900
H	-2.98285100	-5.44199900	-3.70397500
H	-3.47247400	-5.12808800	-2.01846100
C	-2.31403100	1.52767600	0.50433500
C	-1.38291400	2.07929000	1.61960000
C	-2.49205300	3.00742800	-0.01270200
H	-3.26352100	1.18493300	0.94672900
C	-1.97783400	3.48643200	1.37937400
H	-0.34614000	2.05862800	1.25137000
H	-1.28879100	4.34648200	1.33172600
H	-2.80848300	3.70363500	2.07131600
C	-1.44031000	1.43825900	2.97936200
C	-0.29858500	0.85987600	3.54892600
C	-2.63938600	1.38793000	3.70651100
C	-0.34805300	0.25105600	4.80439900
H	0.64569600	0.88880300	2.99763600
C	-2.69432100	0.78120300	4.96033300
H	-3.54622400	1.83090700	3.28496500
C	-1.54709200	0.20987600	5.51550400
H	0.55635200	-0.19255400	5.22865800
H	-3.63906500	0.75448400	5.50937800
H	-1.58905700	-0.26500300	6.49881300
C	-0.87423500	3.82344500	-3.32904600
C	0.39340800	3.44651600	-2.46539700
C	-5.94303100	4.39468000	-0.75017200
C	-5.82386800	3.18515800	-1.74886100
B	-3.93801200	3.34550100	-0.49520100
B	-1.48875200	3.38652000	-1.17852800
O	-1.89644900	4.00163500	-2.33563300
O	-0.20327600	2.91702400	-1.26698300
O	-4.58911400	4.53181100	-0.28113900
O	-4.70457800	2.45856500	-1.20945200
C	1.27235300	2.37037900	-3.08772900
H	1.69023600	2.71978800	-4.04439900
H	2.11254700	2.14660700	-2.41333900
H	0.72390500	1.43592300	-3.26548500
C	1.23424200	4.65075100	-2.04829100
H	0.62904300	5.33793600	-1.42151300
H	2.06851700	4.28951500	-1.42467900
H	1.66946900	5.16461600	-2.91878300
C	-0.73015600	5.10499800	-4.13298100
H	0.08580300	5.01295200	-4.86590800
H	-1.66196800	5.30518100	-4.68367500
H	-0.52468700	5.96453000	-3.48307900
C	-1.34785500	2.68613700	-4.23480700
H	-2.32428100	2.95555100	-4.66449500
H	-0.64717700	2.50346600	-5.06237500
H	-1.47446800	1.75087800	-3.66870400
C	-6.80915500	4.08251100	0.46853200
H	-6.68074400	4.88307000	1.21210200
H	-7.87490600	4.02665300	0.20387600
H	-6.51430400	3.13182700	0.93787800
C	-6.38215700	5.70318400	-1.38678900
H	-7.37664800	5.59498600	-1.84540800
H	-6.44440300	6.48852400	-0.61856500
H	-5.67339800	6.03601200	-2.15654400
C	-5.43313700	3.61543500	-3.16228600
H	-5.18486100	2.71861200	-3.74934000
H	-6.25537500	4.14124400	-3.66859300
H	-4.54539700	4.26446700	-3.14711100
C	-7.04002000	2.27502200	-1.79170400
H	-7.93105600	2.83661000	-2.11065900
H	-6.87173500	1.46354900	-2.51552600
H	-7.24297300	1.82072800	-0.81286400
C	-2.46281300	7.05004900	1.10449400
C	-1.21992500	7.11330300	0.17275200
H	-3.16971800	6.28975500	0.73543600
H	-2.15128200	6.75580400	2.12201200
H	-3.00384200	8.01151500	1.18301600
C	-0.29313200	8.23924700	0.71143900
C	-1.72307400	7.54904200	-1.23094500
H	0.58514000	8.34414000	0.05114000
H	-0.78039800	9.22864300	0.79317300
H	0.07966100	7.95527400	1.71055800
H	-2.28933500	8.49909000	-1.23021400
H	-0.86402800	7.66977900	-1.91392600
H	-2.36652300	6.75700800	-1.64655200
O	-0.56086200	5.93350000	0.12140500

TS2 ´anti

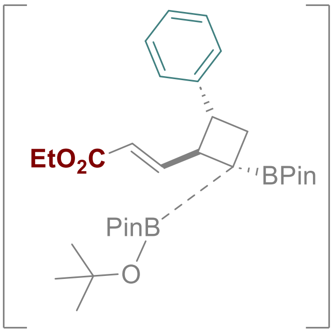

E= -1787.5744566

G_{Corr}= 0.700705

-1 1

C	-2.36025700	-0.54145900	-0.77596000
H	-3.29926200	-0.95213900	-0.39283400
C	-1.85312100	0.60238300	-0.28390300
H	-0.92172800	1.00201800	-0.69889100
C	-1.70888200	-1.28660100	-1.86532800
O	-2.42230100	-2.36376000	-2.21464800
O	-0.66136300	-0.99834100	-2.40656200
C	-1.90713400	-3.17681100	-3.27012000
H	-1.78613300	-2.56061900	-4.17522000
H	-0.90813400	-3.54584800	-2.98840400
C	-2.87194300	-4.31443900	-3.50108800
H	-3.86429200	-3.93701600	-3.79021500
H	-2.49787600	-4.95741100	-4.31120800
H	-2.98109300	-4.92922600	-2.59503400
C	-2.49274400	1.42487700	0.77093700
C	-1.55239500	1.97084100	1.88641500
C	-2.87950300	2.89105900	0.46831500
H	-3.31856700	0.83190400	1.22000200

C	-2.30477900	3.32132000	1.82055100
H	-0.54942700	2.10433100	1.45385800
H	-1.65434700	4.20999400	1.85443200
H	-3.04581300	3.42260600	2.64361100
C	-1.43860300	1.20372300	3.17548400
C	-0.19921100	0.72508800	3.62211100
C	-2.56935500	0.93031900	3.96191400
C	-0.08742600	0.00095600	4.81086300
H	0.69548100	0.92549800	3.02515800
C	-2.46436200	0.20710000	5.14904300
H	-3.54989500	1.29027400	3.63762800
C	-1.22094500	-0.26174400	5.57981100
H	0.89160000	-0.36007100	5.13681900
H	-3.35972400	0.00676800	5.74342400
H	-1.13764600	-0.82852000	6.51047400
C	-0.93479200	3.57228900	-3.19888400
C	0.36736500	3.31743800	-2.35273800
C	-5.96553800	4.55522800	-0.79589800
C	-5.77116500	3.39949300	-1.83993700
B	-4.07768800	3.34405400	-0.27902800
B	-1.18990300	4.30838100	-1.03617100
O	-1.69146900	4.42146000	-2.33618900
O	-0.12130000	3.39099100	-1.01287200
O	-4.68470400	4.61848300	-0.18155000
O	-4.82595700	2.56069300	-1.18570400
C	1.03311800	1.97239200	-2.59619900
H	1.38343100	1.90337700	-3.63826300
H	1.91217800	1.87255300	-1.94020400
H	0.36350100	1.12438900	-2.40426200
C	1.40414200	4.43396400	-2.52821300
H	0.95255500	5.42847600	-2.39335200
H	2.18547300	4.31268600	-1.76276600
H	1.88429100	4.40143900	-3.51740700
C	-0.69510500	4.30644100	-4.51303000
H	-0.02482100	3.72816800	-5.16774100
H	-1.65096000	4.44166600	-5.04228300
H	-0.25583000	5.29990500	-4.35133800
C	-1.72940900	2.29790700	-3.47272400
H	-2.69435700	2.56736200	-3.92360200
H	-1.19845900	1.63432500	-4.17066200
H	-1.93112900	1.74378300	-2.54874100
C	-6.98762000	4.20061500	0.28923500
H	-6.91843500	4.94378900	1.09827700
H	-8.01925000	4.20559500	-0.09342700
H	-6.78066000	3.20976600	0.72150800
C	-6.31711600	5.90843600	-1.39948300
H	-7.28025100	5.85957600	-1.93119900
H	-6.40861300	6.66083100	-0.60059900
H	-5.54976700	6.25603600	-2.10306300
C	-5.14724000	3.89495500	-3.14719200
H	-4.90200700	3.02367600	-3.77333900
H	-5.83587300	4.53629400	-3.71756900
H	-4.21259400	4.44206600	-2.95070600
C	-7.03243200	2.60059300	-2.14083000
H	-7.81943700	3.24884200	-2.55693700
H	-6.81039300	1.81823500	-2.88300700
H	-7.42406100	2.10776900	-1.24060600
C	-2.53131000	6.84252600	1.11541500
C	-1.71672800	6.65791300	-0.16650800
H	-3.38949200	6.15505300	1.10577800
H	-1.91086600	6.63510200	2.00112800
H	-2.90465900	7.87590100	1.19304100
C	-0.51695400	7.60901900	-0.17583700
C	-2.61124100	6.92866800	-1.37624900
H	0.07846900	7.46694100	-1.09164900
H	-0.84308900	8.66011200	-0.13662900
H	0.13406400	7.41471500	0.69081200
H	-3.05104500	7.93436700	-1.28340900
H	-2.04775200	6.87771100	-2.31696400
H	-3.41976800	6.18798200	-1.40637500
O	-1.17972400	5.34156100	-0.13685200

TS3´$_{anti}$

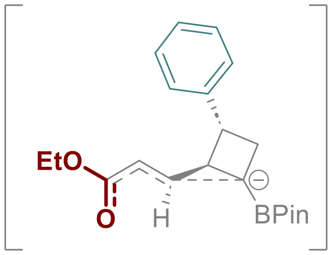

E= -1143.1007619
G_{Corr}= 0.404442

-1 1

C	1.74296100	0.35049400	-0.37690600
H	2.27124300	0.51257700	0.56493000
C	0.42479800	0.70738300	-0.54922600
H	-0.05899400	0.40660500	-1.48481400
C	2.49240200	-0.22298100	-1.47049800
O	3.75383900	-0.55793000	-1.10047900
O	2.09741800	-0.42316500	-2.61009400
C	4.58643700	-1.13909000	-2.09373100
H	4.12173100	-2.06308600	-2.47600500
H	4.68139700	-0.45028400	-2.94954000
C	5.93401700	-1.42418000	-1.47218100
H	5.83810700	-2.12373300	-0.62782000
H	6.60360000	-1.87545600	-2.21938500
H	6.40175000	-0.49812800	-1.10459100
C	-0.44573000	1.49118600	0.34454000
C	0.27496900	2.20555300	1.51766800
C	-0.25908200	2.74839000	-0.50825400
H	-1.44296100	1.06167100	0.54858700
C	0.69396500	3.33194900	0.52095900
H	1.10864400	1.64169600	1.96253200
H	1.77464300	3.28842600	0.26549700
H	0.48974700	4.35446000	0.88888800
C	-0.64267400	2.65170100	2.62678700
C	-1.83014900	3.35223200	2.35754300
C	-0.33022000	2.37772100	3.96454900
C	-2.67074300	3.76377600	3.39016600
H	-2.08972700	3.57846900	1.31901900
C	-1.16925000	2.78944800	5.00253900
H	0.58830900	1.83129800	4.19785900
C	-2.34424800	3.48456700	4.71951900
H	-3.58955900	4.30779300	3.15582800
H	-0.90137200	2.56358100	6.03798100
H	-3.00341200	3.80759700	5.52910500
C	-1.06140700	4.22373600	-3.83970700
C	-1.52488100	2.72988300	-3.99289500
B	-0.70138200	3.07516200	-1.88707800
O	-0.27831800	4.17635300	-2.64809200
O	-1.60859800	2.30264000	-2.63559100
C	-2.87820400	2.55028200	-4.66572900
H	-2.86927900	2.97001200	-5.68355400
H	-3.11704100	1.47838300	-4.74281500
H	-3.68137300	3.03393600	-4.09358100
C	-0.47788300	1.86026400	-4.69447500
H	0.51505800	1.98141200	-4.23606000
H	-0.76620900	0.80302500	-4.59164000
H	-0.39967300	2.09124900	-5.76724400
C	-0.20347100	4.74119800	-4.98578600
H	-0.74768700	4.68283600	-5.94112200
H	0.06087400	5.79528000	-4.80955700
H	0.73031600	4.17023800	-5.07767000
C	-2.23218400	5.17885900	-3.59511300
H	-1.83452100	6.15629400	-3.28229200
H	-2.83844700	5.32918000	-4.50082700
H	-2.88809500	4.80728900	-2.79320000

int5$_{syn}$

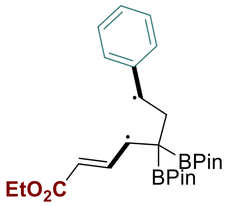

E= -1554.33207333
G_{Corr}= 0.576644

0 3

C	-1.87539300	0.40426900	-0.17187700
H	-2.65936300	0.20352300	-0.90673100
C	-1.04765000	1.51366100	-0.27857800
H	-0.29564400	1.64218200	0.50547100
C	-1.11132000	2.47547900	-1.27533300
C	-0.27987400	3.72697800	-1.23540300
C	3.37060600	3.80220100	-0.19541700
B	1.21459500	3.42399100	-0.80824200
O	2.03001100	4.33870500	-0.20357600
O	1.85140500	2.24535900	-1.07293900
C	4.09935800	4.41824800	-1.38642300
H	4.06305800	5.51413600	-1.29981300
H	5.15341900	4.10825100	-1.41795400
H	3.62175000	4.13593700	-2.33640500
C	4.05213800	4.19104100	1.10523800
H	5.04173900	3.71528400	1.17538300
H	4.19414700	5.28141400	1.13974500
H	3.45810400	3.89499100	1.97984300
C	3.10693600	2.26077100	-0.36123300
C	2.86481800	1.54473800	0.96672300
H	2.47693200	0.53686900	0.75887000
H	3.79316200	1.44543200	1.54715800
H	2.12161400	2.07470600	1.58186400
C	4.15301200	1.52260300	-1.17828200
H	5.14168400	1.61062000	-0.70367500
H	3.89510000	0.45468900	-1.23474800
H	4.21830100	1.91308600	-2.20235900
C	-0.93288500	4.83427000	-0.30588300
C	-1.63482600	4.28978800	0.89646100
H	-1.67133800	5.39655200	-0.89749500
H	-0.14327000	5.54812500	-0.02699300
C	-1.05850100	3.94822400	2.14895400
C	-1.85362100	3.28293400	3.12654500
C	0.29774000	4.21384700	2.48515200
C	-1.32502300	2.90463100	4.35112800
H	-2.89847100	3.06100100	2.89182300
C	0.81621700	3.83063700	3.71601700
H	0.94560200	4.71022600	1.76176300
C	0.01590900	3.17441500	4.65821000
H	-1.95758900	2.38925800	5.07840600
H	1.86345400	4.04379200	3.94665200
H	0.43196600	2.87356800	5.62238800
C	0.28320200	5.83803000	-4.33573800
C	0.31281500	4.37626000	-4.91779500
B	-0.07212700	4.32408700	-2.68403200
O	-0.25047900	3.60623800	-3.83200700
O	0.35580500	5.59954300	-2.91324400
C	1.72846600	3.84946000	-5.14171900
H	2.35699000	3.99722400	-4.25081200
H	1.67983500	2.77025000	-5.34815900
H	2.21154800	4.34332900	-5.99663000
C	-0.53014500	4.17138500	-6.16451400
H	-0.17576300	4.82293300	-6.97719900
H	-0.44596500	3.12794000	-6.50268900
H	-1.59071800	4.38596500	-5.97896500
C	1.45608200	6.70957800	-4.75204000
H	1.48173100	6.82144400	-5.84628600
H	1.35052200	7.71101400	-4.30907000
H	2.41344900	6.28820600	-4.41843800
C	-1.03522800	6.56149900	-4.59805400
H	-1.06201900	7.48300900	-3.99801500
H	-1.13807900	6.83743000	-5.65719900
H	-1.89906600	5.94280400	-4.31200200
H	-1.85857400	2.35532400	-2.06865600
H	-2.69078600	4.03001000	0.76910100
C	-1.79238400	-0.54234600	0.94193800
O	-2.54902900	-1.47891400	1.10158000
O	-0.78918200	-0.26518200	1.78630300
C	-0.64298900	-1.08348400	2.94531200
H	-0.56374300	-2.13872100	2.63997900
H	-1.54565800	-0.98670000	3.57040800
C	0.59109300	-0.62373800	3.68338600
H	0.71783600	-1.21781400	4.60026600
H	1.49126800	-0.74892300	3.06305200
H	0.50586000	0.43636600	3.96604800

int5$_{anti}$

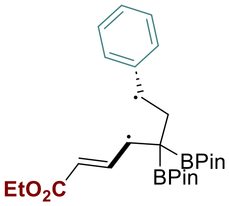

E= -1554.3338405
G_{Corr}= 0.576868

0 3

C	-2.12505400	0.63622600	-0.41163100
H	-1.63358600	-0.15905500	0.15499900
C	-1.43003300	1.77954700	-0.79191100
H	-1.98180600	2.54018600	-1.35083500
C	-0.11897800	2.04623500	-0.44048700
C	0.54456100	3.38023800	-0.64170600
C	4.29807600	3.85933700	-0.63550500
B	2.11848100	3.24165400	-0.65596900
O	2.97564600	4.17207900	-0.14377500
O	2.76120300	2.19591700	-1.25403000
C	4.52240900	4.72471400	-1.87345000
H	4.39246200	5.78185400	-1.59917000
H	5.53598800	4.59422600	-2.27810100
H	3.79589600	4.48762700	-2.66498900
C	5.32095900	4.19378000	0.43608800
H	6.32489300	3.87354300	0.11965700
H	5.34558300	5.28129100	0.60026700
H	5.08234100	3.70919500	1.39188400
C	4.17158600	2.32705700	-0.96973000
C	4.47032800	1.42567400	0.22551900
H	4.16818000	0.39670800	-0.01922200
H	5.54295900	1.42119700	0.46622400
H	3.91194800	1.74197800	1.11937800
C	4.96565800	1.87527700	-2.18372800
H	6.03910900	2.06224400	-2.03100200
H	4.82586300	0.79513100	-2.33849800
H	4.64417000	2.39485600	-3.09592600
C	0.09797200	4.39715100	0.47273700
C	-1.33006300	4.81729700	0.34639100
H	0.74081500	5.28600300	0.38192200
H	0.30475400	3.95644200	1.46014000
C	-2.44579700	4.26512400	1.02463700
C	-3.76139100	4.68272500	0.66841200
C	-2.33444400	3.25886900	2.02626600
C	-4.88304600	4.11225200	1.25141500
H	-3.87754200	5.45604000	-0.09635300
C	-3.46336000	2.69565000	2.60398500
H	-1.34629900	2.90611500	2.32864100
C	-4.74606200	3.10927000	2.22039700
H	-5.87927700	4.44380900	0.94752700
H	-3.34819700	1.91608200	3.36141400
H	-5.63022500	2.65578400	2.67414200
C	-0.05055800	5.56175100	-3.68284100

C	0.03766300	4.12943000	-4.32641100
B	0.26789700	3.99420500	-2.07530400
O	-0.10013800	3.27169800	-3.17251200
O	0.44388700	5.32216100	-2.34728700
C	1.40477500	3.83136800	-4.93722800
H	2.21800700	4.06797300	-4.23473800
H	1.46335100	2.75930800	-5.17587600
H	1.56510400	4.40110900	-5.86343000
C	-1.06756000	3.80828200	-5.31753000
H	-1.04495400	4.51599800	-6.15965400
H	-0.92312600	2.79454500	-5.71960000
H	-2.05913600	3.85105500	-4.84790300
C	0.81743000	6.61314300	-4.35254500
H	0.52346700	6.73932000	-5.40525400
H	0.68914300	7.58018700	-3.84402200
H	1.88155800	6.34558000	-4.31408400
C	-1.48505900	6.06597400	-3.54202200
H	-1.48759800	6.96672300	-2.91056400
H	-1.91455200	6.33081700	-4.51870800
H	-2.13318100	5.31359800	-3.06743200
H	0.42352500	1.27604900	0.12191300
H	-1.54765400	5.56514000	-0.42361600
C	-3.56227700	0.46292600	-0.61858700
O	-4.20600100	-0.46951900	-0.17957200
O	-4.11362100	1.45496900	-1.33029900
C	-5.53093900	1.44724500	-1.48779100
H	-6.00371600	1.45466500	-0.49219700
H	-5.83759100	0.51688700	-1.99187400
C	-5.91653300	2.66508400	-2.29181000
H	-7.00733500	2.68778600	-2.43053100
H	-5.61375800	3.58880500	-1.77622000
H	-5.44241600	2.64701000	-3.28478000

MECP 5$_{syn}$

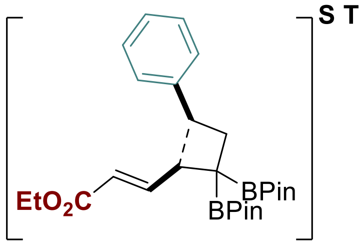

E_S = -1554.31719173
$G_{Corr}S$ = 0.580558
E_T = -1554.31635342
$G_{Corr}T$ = 0.576990

0 1-3

C	-1.35466300	0.68426100	0.59198200
H	-2.23663500	0.47090700	-0.01774300
C	-0.48301200	1.70090300	0.25195500
H	0.36687200	1.86577700	0.92392600
C	-0.67280700	2.61072100	-0.78557600
C	0.23264000	3.81418500	-0.99164900
C	3.98680900	3.55428500	-0.54588300
B	1.75451000	3.40153400	-0.90313600
O	2.69445200	4.10694400	-0.20806200
O	2.26665100	2.29926800	-1.52598900
C	4.55933300	4.42138400	-1.66461500
H	4.60922500	5.46409000	-1.31882400
H	5.57346900	4.10234700	-1.94360900
H	3.92307000	4.39120100	-2.56212700
C	4.88581600	3.60429100	0.67734400
H	5.83968700	3.09543900	0.47226400
H	5.10586300	4.65088600	0.93579900
H	4.41399400	3.12937300	1.54749700
C	3.60627900	2.10608600	-1.02450400
C	3.50891900	1.10268700	0.12326200
H	3.02830800	0.18458200	-0.24555200
H	4.50253400	0.83943800	0.51303100
H	2.90054500	1.49292500	0.95302900
C	4.48119400	1.55563800	-2.13694900
H	5.52964800	1.50047200	-1.80803700
H	4.15213200	0.53975400	-2.40170700
H	4.42753000	2.17803700	-3.03967000
C	-0.16281000	4.84116900	0.11461700
C	-1.54811300	4.44973900	0.53791600
H	-0.13987100	5.87612400	-0.26333700
H	0.53675700	4.79661000	0.95868300
C	-1.93103700	3.92165900	1.80241500
C	-3.26396700	3.46737600	1.99184200
C	-1.01058300	3.70193800	2.86112400
C	-3.64648000	2.82352100	3.16072400
H	-3.98993400	3.61420600	1.18720300
C	-1.40393300	3.06432500	4.02954000
H	0.02720200	4.02423300	2.75745300
C	-2.71948200	2.61525500	4.18846200
H	-4.67514200	2.47306900	3.27444100
H	-0.67395100	2.90413600	4.82669900
H	-3.01999900	2.10555600	5.10680600
C	0.17547600	5.91487500	-4.13995400
C	0.12780200	4.44931900	-4.70719100
B	0.12892900	4.40782000	-2.44515500
O	-0.25950300	3.68995200	-3.54037900
O	0.50488500	5.68582600	-2.75236300
C	1.49982900	3.92991500	-5.13388000
H	2.25717000	4.10395700	-4.35435400
H	1.43280000	2.84518600	-5.30427900
H	1.83910900	4.40614000	-6.06470100
C	-0.88979800	4.22595400	-5.81188000
H	-0.67110300	4.87698400	-6.67145300
H	-0.84307700	3.18167400	-6.15493700
H	-1.91299400	4.42795800	-5.46875900
C	1.23776000	6.80589500	-4.76163800
H	1.07284800	6.90359100	-5.84493700
H	1.18512000	7.81161700	-4.31840800
H	2.24878900	6.41163500	-4.59384800
C	-1.18323300	6.61109500	-4.16441200
H	-1.12155000	7.53410900	-3.56911500
H	-1.47889000	6.88206700	-5.18787500
H	-1.96991100	5.97645600	-3.72931900
H	-1.49159000	2.43015000	-1.49040600
H	-2.35000100	4.63631500	-0.18399700
C	-1.24297900	-0.09389600	1.82149600
O	-2.07576400	-0.89348000	2.20504900
O	-0.12464200	0.16988100	2.51759000
C	0.01716400	-0.44802300	3.79324800
H	0.07242500	-1.54132400	3.66569800
H	-0.87544800	-0.23194100	4.40183100
C	1.26944400	0.09394000	4.43978100
H	1.41637200	-0.37755900	5.42281000
H	2.15486700	-0.11724500	3.82168000
H	1.19770400	1.18222000	4.58556600

MECP 5$_{anti}$

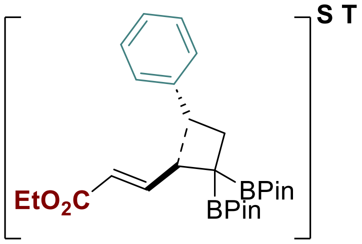

E_S = -1554.3145665
$G_{Corr}S$ = 0.578508
E_T = -1554.31399464
$G_{Corr}T$ = 0.576151

0 1-3

C	-1.42013500	0.67042200	0.67345600
H	-2.29211400	0.43343100	0.05804000
C	-0.62886600	1.76626200	0.39397800
H	0.24048400	1.92994800	1.04121100
C	-0.87487700	2.70571200	-0.61181000
C	0.05495100	3.87426200	-0.90150400
C	3.81563800	3.53186300	-0.54039200
B	1.57008500	3.42359800	-0.83518200
O	2.54561100	4.12705200	-0.18938300
O	2.03977900	2.29140700	-1.43722400
C	4.38578500	4.35272700	-1.69412800
H	4.45875000	5.40484500	-1.38174100
H	5.39047000	4.00660400	-1.97513600
H	3.73757000	4.30320800	-2.58181700
C	4.74268200	3.59523100	0.66109300
H	5.68184600	3.06287500	0.44913500
H	4.98967700	4.64393100	0.88351700
H	4.28245200	3.15450800	1.55508200
C	3.38872700	2.08024500	-0.96929700
C	3.30146300	1.10885100	0.20637400
H	2.79535800	0.19049400	-0.12654700
H	4.29842200	0.83608100	0.58049000
H	2.72098100	1.53357700	1.03900200
C	4.21707100	1.48007300	-2.09242000
H	5.27379700	1.40826500	-1.79493800
H	3.85793800	0.46500600	-2.31824000
H	4.15033300	2.07870800	-3.01024400
C	-0.29953000	4.96408900	0.15524900
C	-1.59132100	4.51438800	0.78237000
H	-0.38569200	5.96425200	-0.29720200
H	0.47308400	5.02958700	0.93390300
C	-2.89641100	4.82029800	0.29621600
C	-4.03686300	4.37721700	1.01660200
C	-3.11341200	5.53072800	-0.91308400
C	-5.31978300	4.62998500	0.55179900
H	-3.89160500	3.83011800	1.95226400
C	-4.40115200	5.77714000	-1.37040400
H	-2.26006000	5.89023400	-1.49269000
C	-5.51145200	5.32977700	-0.64520700
H	-6.18341500	4.28311600	1.12535200
H	-4.54540100	6.32940200	-2.30230600
H	-6.52200100	5.53044200	-1.00846200
C	0.06910700	5.83552900	-4.14591100
C	0.03774800	4.34248600	-4.64609800
B	-0.05044400	4.39976200	-2.38377100
O	-0.38621200	3.63069300	-3.46141100
O	0.29930800	5.67536600	-2.72941500
C	1.42194500	3.80730200	-5.00873400
H	2.14495100	3.98634000	-4.19942500
H	1.35671200	2.72160200	-5.17056400
H	1.80458600	4.27101800	-5.92902200
C	-0.94435200	4.07106400	-5.77218200
H	-0.69477300	4.68216500	-6.65259400
H	-0.89176400	3.01243100	-6.06618800
H	-1.97716900	4.29231100	-5.47330500
C	1.19322500	6.67677900	-4.72906600
H	1.10980900	6.72899200	-5.82470200
H	1.13184600	7.70178200	-4.33419100
H	2.18001700	6.27153000	-4.46951800
C	-1.26698600	6.55378300	-4.31527300
H	-1.23216000	7.50930700	-3.77110400
H	-1.47025000	6.77046600	-5.37400600
H	-2.10182500	5.95950000	-3.91473300
H	-1.73066400	2.53042300	-1.27312000
H	-1.53945900	4.03579600	1.76376800
C	-1.17482700	-0.23925600	1.78800300
O	-1.84910000	-1.21988400	2.03665000
O	-0.11780800	0.11699200	2.53739500
C	0.19687000	-0.69366900	3.66673800
H	0.44674800	-1.71210400	3.32808800
H	-0.68874400	-0.77153700	4.31761100
C	1.35758100	-0.05301600	4.38976400
H	1.61737200	-0.64805600	5.27708200
H	2.24448300	0.00400700	3.74105900
H	1.09923100	0.96452900	4.71988700

References

[1] H. Fang, C. Empel, I. Atodiresei, R. M. Koenigs, *ACS Catal.* **2023**, *13,* 6445-6451.
[2] A. Marotta, H. Fang, C. E. Adams, K. Sun Marcus, C. G. Daniliuc, J. J. Molloy, *Angew. Chem. Int. Ed.* **2023**, *62*, e202307540.
[3] M. Wienhold, B. Kweon, C. McLaughlin, M. Schmitz, T. J. Zähringer, C. G. Daniliuc, C. Kerzig, R. Gilmour, *Angew. Chem. Int. Ed.* **2023**, *135*, e202304150.
[4] S. Chen, Y. N. Wang, J. Xie, W. Li, M. Ye, X. Ma, K. Yang, S. Li, Y. Lan, Q. Song, *Nat. Commun.* **2024**, *15*, 5479.
[5] M. Z. Liang, S. J. Meek, *Angew. Chem., Int. Ed.* **2019**, *131*, 14372-14377.
[6] N. R. Vautravers, P. André, A. M. Z. Slawin, D. J. Cole-Hamilton, *Org. Biomol. Chem.* **2009**, *7,* 717-724.
[7] F. Scheidt, J. Neufeld, M. Schäfer, C. Thiehoff, R. Gilmour, *Org. Lett.* **2018**, *20,* 8073-8076.
[8] M. Ratushnyy, M. Kamenova, V. Gevorgyan, *Chem. Sci.* **2018**, *9*, 7193-7197.
[9] M. Mato, B. Herlé, A. M. Echavarren, *Org. Lett.* **2018**, *20*, 4341-4345.
[10] O. Saku, H. Ishida, E. Atsumi, Y. Sugimoto, H. Kodaira, Y. Kato, S. Shirakura, Y. Nakasato, *J. Med. Chem.* **2012**, *55,* 3436-3451.
[11] J. R. Coombs, L. Zhang, J. P. Morken, *Org. Lett.* **2015**, *17*, 1708–171.
[12] I. T. Crouch, T. Dreier, D. E. Frantz, *Angew. Chem. Int. Ed.* **2011**, *50*, 6128-6132.
[13] J. J. Molloy, J. B. Metternich, C. G. Daniliuc, A. J. Watson, R. Gilmour, *Angew. Chem., Int. Ed.* **2018**, *57*, 3168-3172.
[14] J. Cornil, P. G. Echeverria, P. Phansavath, V. Ratovelomanana-Vidal, A. Guérinot, J. Cossy, *Org. Lett.* **2015**, *17*, 948-951.
[15] Kessil Photoscience products overview https://kessil.com/products/science_main.php (accessed April 2025).
[16] K. Teegardin, J. I. Day, J. Chan, J. Weaver, *Organic process research & development* **2016**, *20,* 1156-1163.
[17] L. Buzzetti, G. E. Crisenza, P. Melchiorre, *Angew. Chem., Int. Ed.* **2019**, *58,* 3730-3747.
[18] N. Kumar, R. R. Reddy, A. Masarwa, *Chem. Eur. J.* **2019**, *25,* 8008-8012.
[19] Y. Yang, J. Tsien, J. M. Hughes, B. K. Peters, R. R. Merchant, T. Qin, *Nat. Chem.* **2021**, *13,* 950-955.
[20] M. R. Harris, Q. Li, Y. Lian, J. Xiao, A. T. Londregan, *Org. Lett.* **2017**, *19*, 2450-2453.
[21] J. Y. Son, S. Aikonen, N. Morgan, A. S. Harmata, J. J. Sabatini, R. C. Sausa, E. F. C. Byrd, D. H. Ess, R. S. Paton, C. R. Stephenson, *J. Am. Chem. Soc.* **2023**, *145,* 16355-16364.
[22] M. D. Aparece, C. Gao, G. J. Lovinger, J. P. Morken, *Angew. Chem. Int. Ed.* **2019**, *131,* 602-605.
[23] L. Tang, G. Lv, Y. Fu, X. P. Chang, R. Cheng, L. Wang, Q. Zhou, *J. Org. Chem.* **2022**, *87,* 14763-14777.
[24] Bruker AXS (**2021**) *APEX4 Version 2021.4-0*, *SAINT Version 8.40B* and *SADABS Bruker AXS area detector scaling and absorption correction Version 2016/2*, Bruker AXS Inc., Madison, Wisconsin, USA.
[25] G. M. Sheldrick, *SHELXT – Integrated space-group and crystal-structure determination*, *Acta Cryst.*, **2015**, *A71*, 3-8.
[26] G. M. Sheldrick, *Crystal structure refinement with SHELXL*, *Acta Cryst.*, **2015**, *C71 (1),* 3-8.
[27] Bruker AXS (**1998**) *XP – Interactive molecular graphics, Version 5.1,* Bruker AXS Inc., Madison, Wisconsin, USA.
[28] Gaussian 16, Revision C.01, M. J. Frisch, G. W. Trucks, H. B. Schlegel, G. E. Scuseria, M. A. Robb, J. R. Cheeseman, G. Scalmani, V. Barone, G. A. Petersson, H. Nakatsuji, X. Li, M. Caricato, A. V. Marenich, J. Bloino, B. G. Janesko, R. Gomperts, B. Mennucci, H. P. Hratchian, J. V. Ortiz, A. F. Izmaylov, J. L. Sonnenberg, D. Williams-Young, F. Ding, F. Lipparini, F. Egidi, J. Goings, B. Peng, A. Petrone, T. Henderson, D. Ranasinghe, V. G. Zakrzewski, J. Gao, N. Rega, G. Zheng, W. Liang, M. Hada, M. Ehara, K. Toyota, R. Fukuda, J. Hasegawa, M. Ishida, T. Nakajima, Y. Honda, O. Kitao, H. Nakai, T. Vreven, K. Throssell, J. A. Montgomery, Jr., J. E. Peralta, F. Ogliaro, M. J. Bearpark, J. J. Heyd, E. N. Brothers, K. N. Kudin, V. N. Staroverov, T. A. Keith, R. Kobayashi, J. Normand, K.

Raghavachari, A. P. Rendell, J. C. Burant, S. S. Iyengar, J. Tomasi, M. Cossi, J. M. Millam, M. Klene, C. Adamo, R. Cammi, J. W. Ochterski, R. L. Martin, K. Morokuma, O. Farkas, J. B. Foresman, and D. J. Fox, Gaussian, Inc., Wallingford CT, **2016**.
[29] J. D. Chai, M. Head-Gordon, *Chem. Phys.* **2008**, *10*, 6615.
[30] A. V. Marenich, C. J. Cramer, D. G. Truhlar, *J. Phys. Chem. B.* **2009**, *113*, 6378.
[31] F. Weigend, *Phys. Chem. Chem.* **2006**, *8*, 1057.
[32] F. Weigend, R. Ahlrichs, *Phys. Chem. Chem. Phys.* **2005**, *7*, 3297.
[33] S. Grimme, *Chem. Eur. J.* **2012**, *18*, 9955.
[34] G. Luchini, J. V. Alegre-Requena, I. Funes-Ardoiz, R. S. Paton, *F1000Research.* **2020**, 9, 291.
[35] C. Y. Legauklt, *CYLview*, 1.0b, Université de Sherbrooke, **2009**. http://www.cylview.org (accessed **2024**).

Spectrum

^{1}H-NMR of S1 (400 MHz, $CDCl_3$)

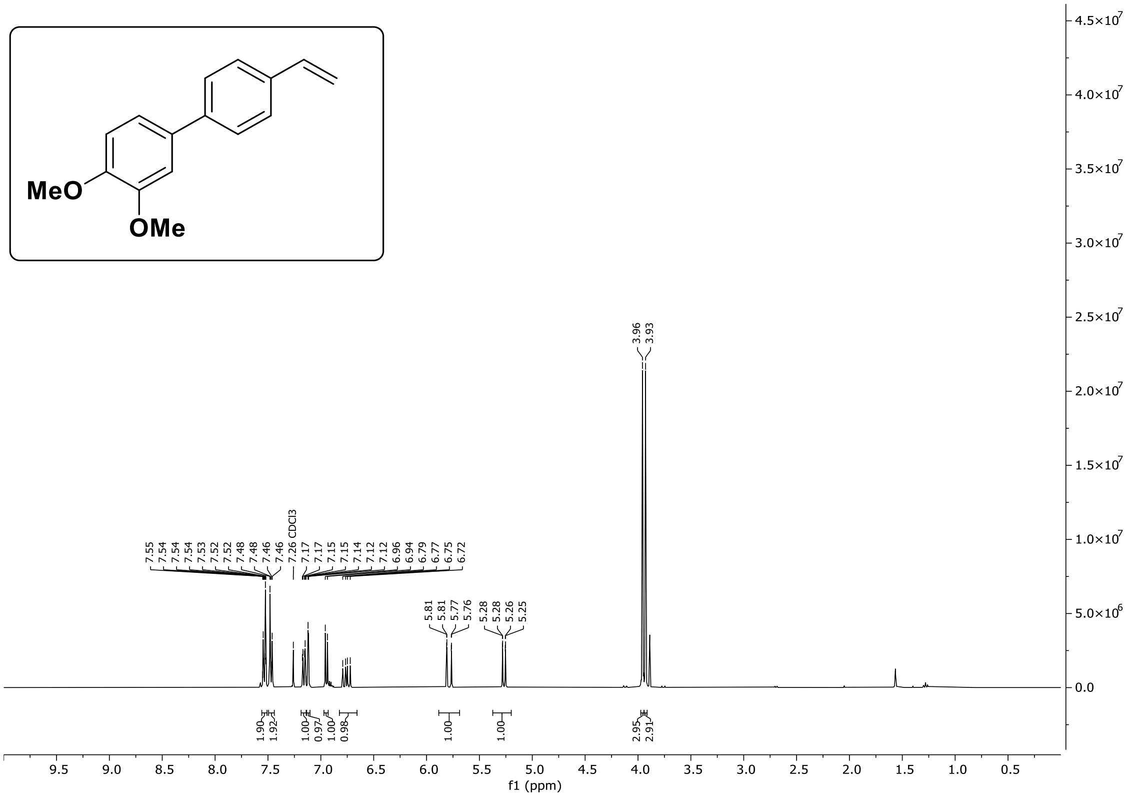

^{1}H-NMR of S2 (400 MHz, $CDCl_3$)

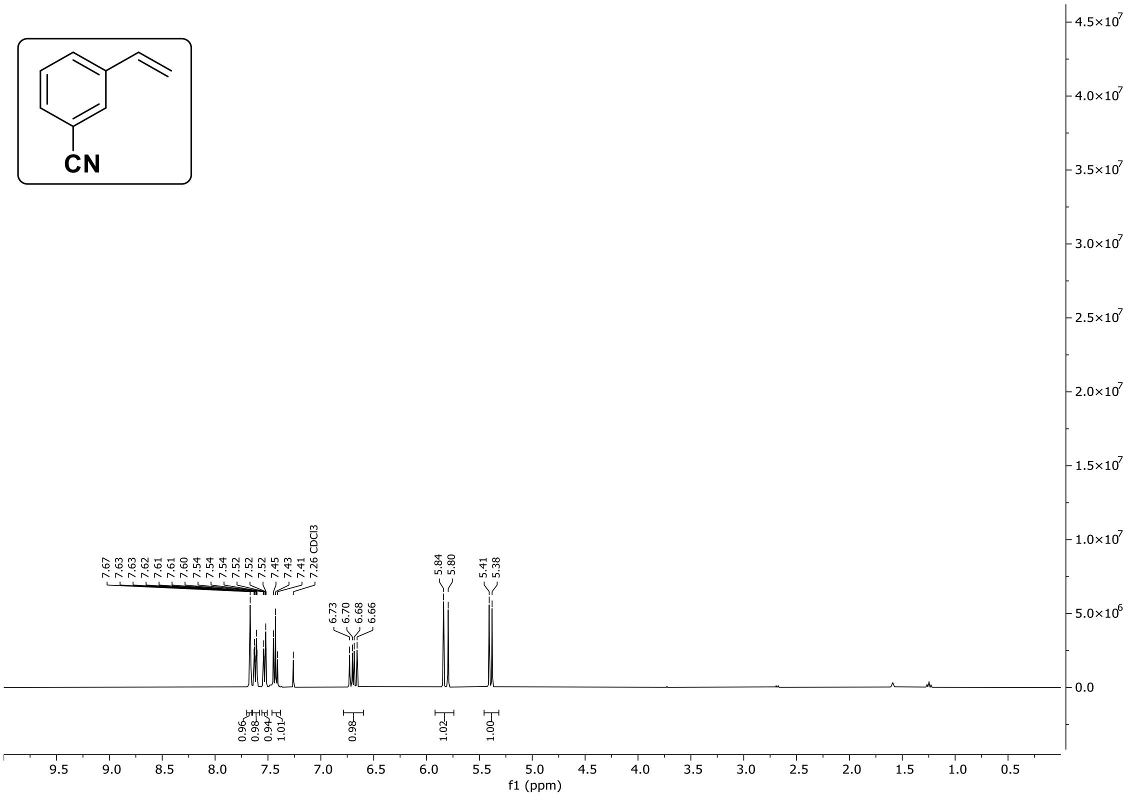

^{1}H-NMR of S3 (400 MHz, DMSO)

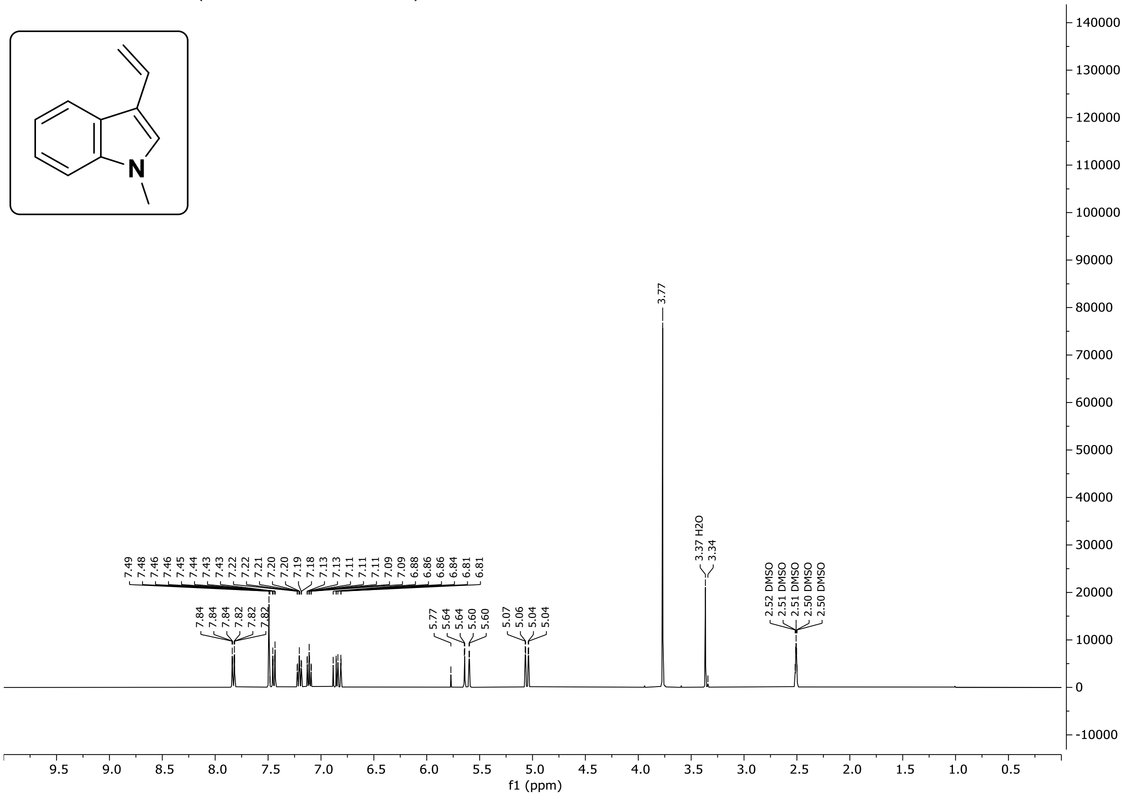

^{1}H-NMR of S4 (400 MHz, $CDCl_3$)

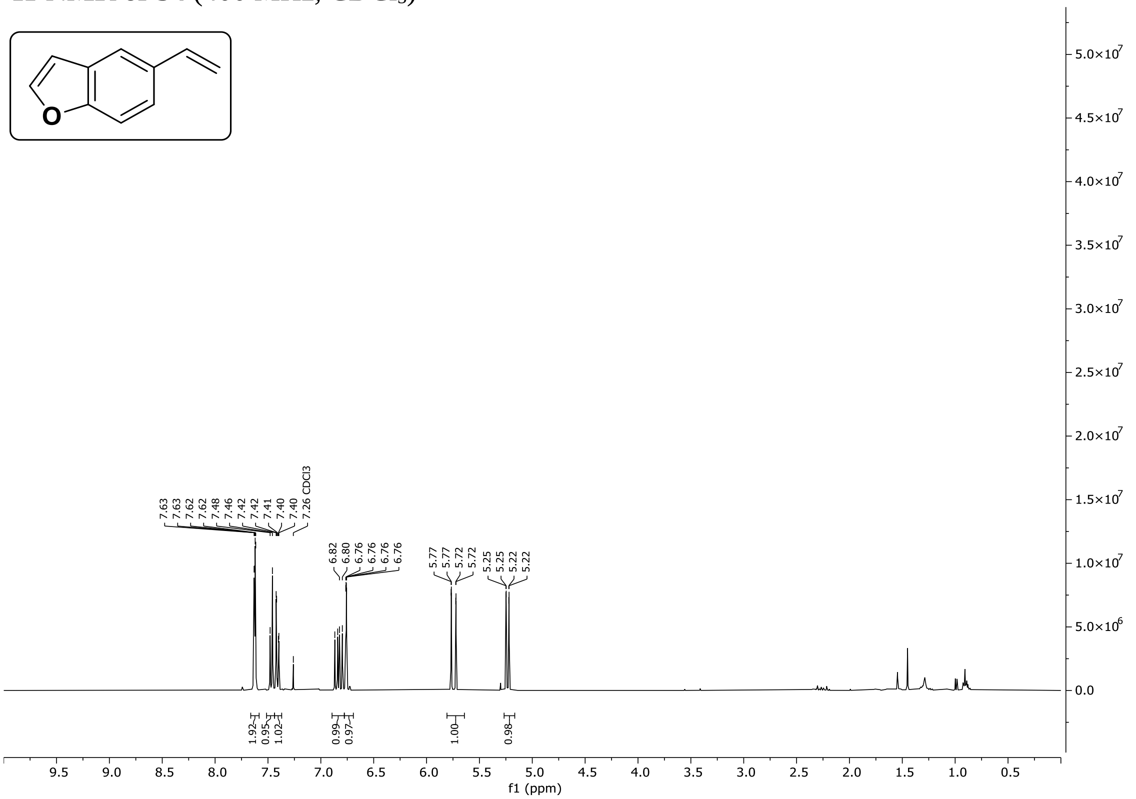

^{1}H-NMR of S5 (400 MHz, $CDCl_3$)

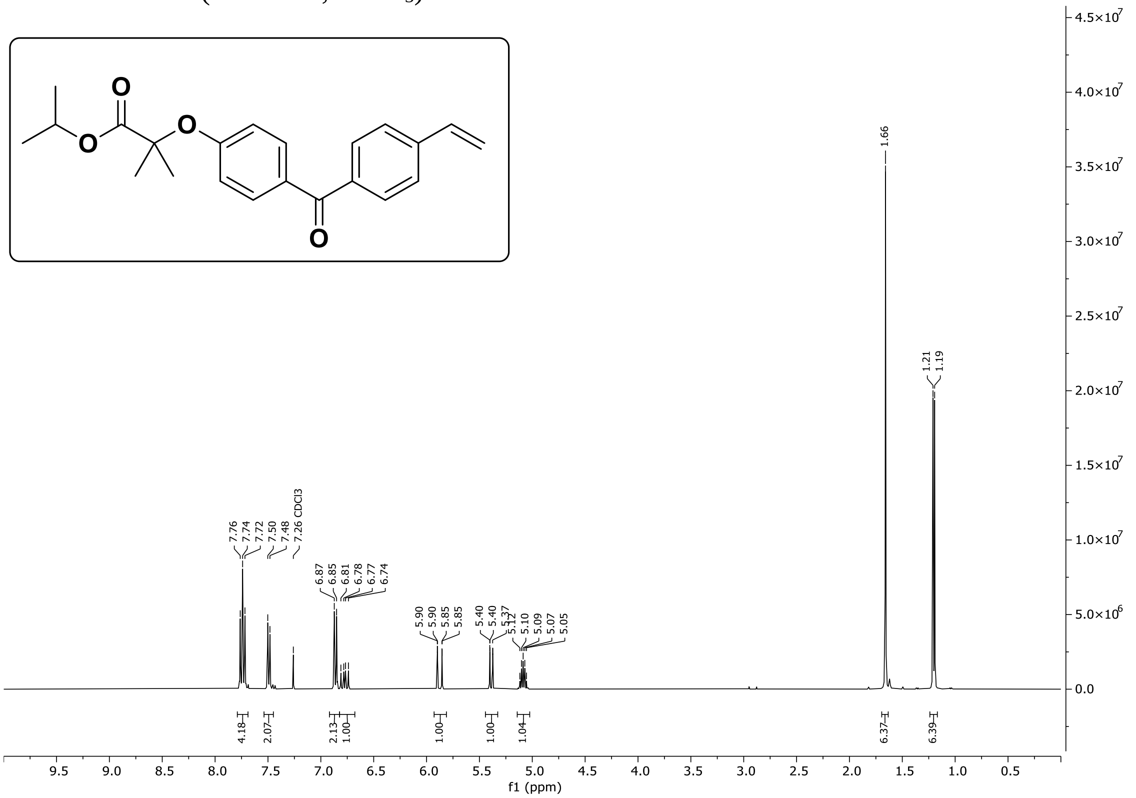

^{1}H-NMR of S6 (400 MHz, $CDCl_3$)

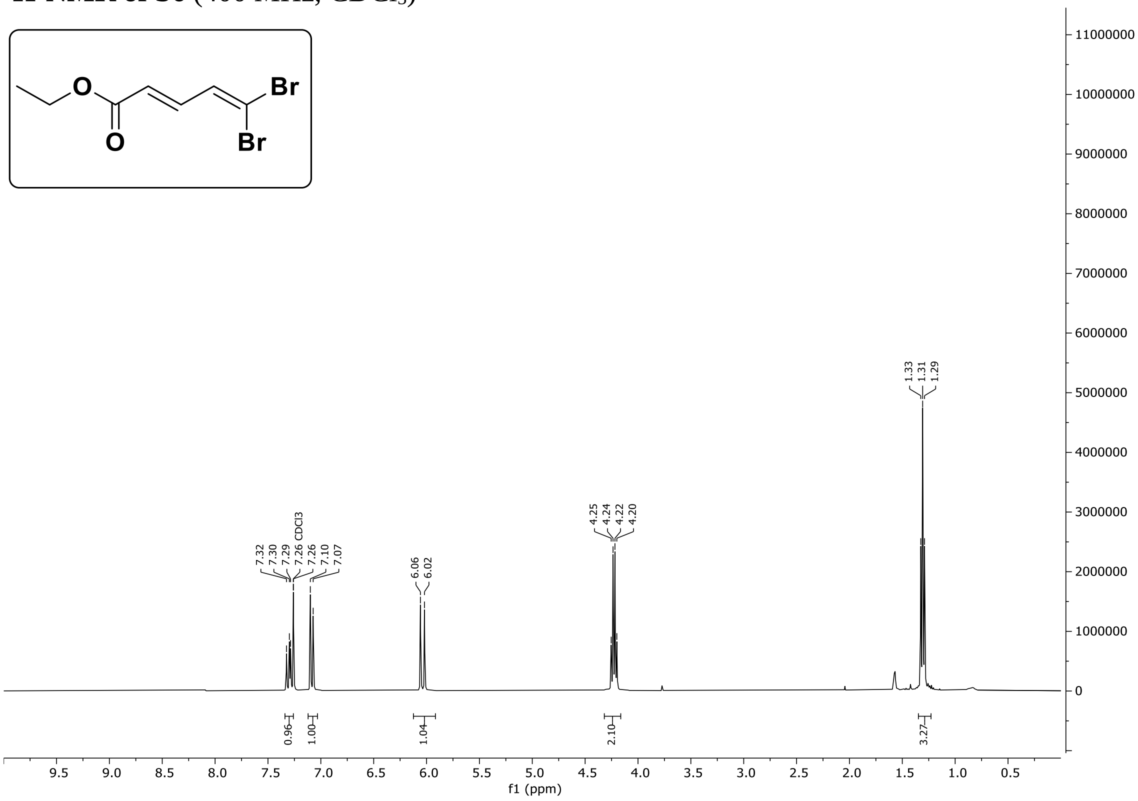

^{1}H-NMR of S7 (400 MHz, $CDCl_3$)

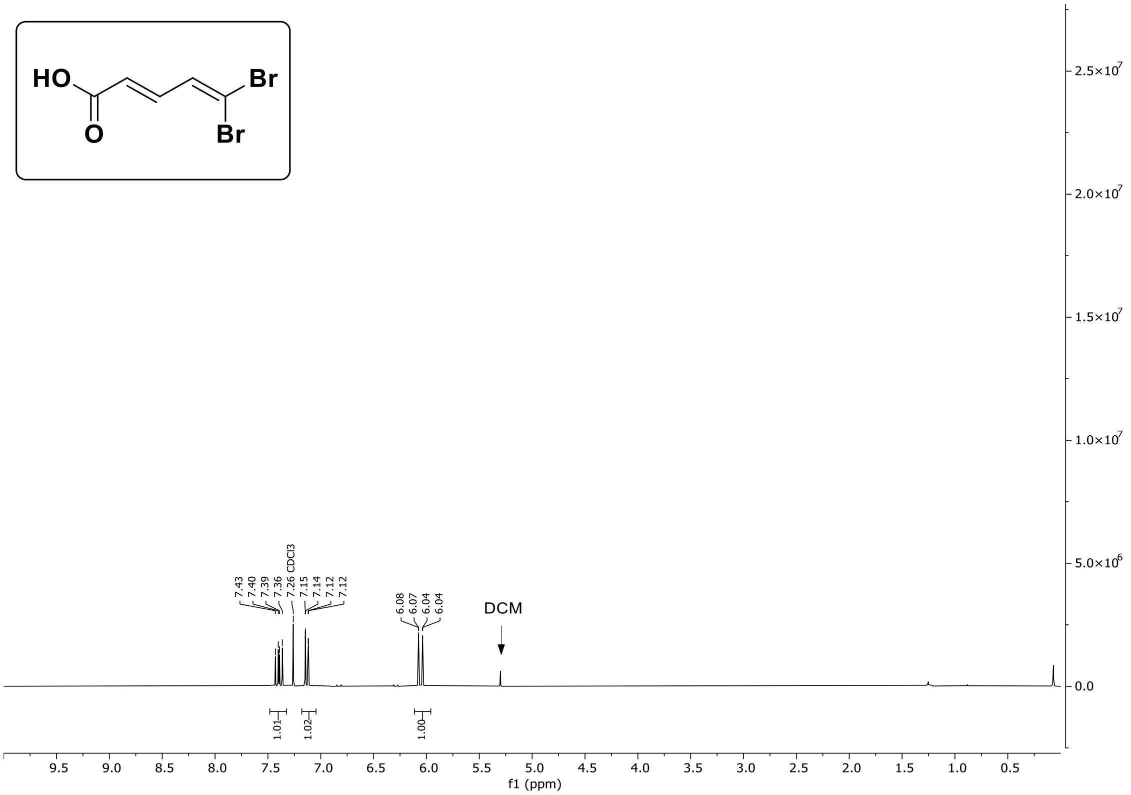

^{13}C-NMR of S7 (100 MHz, $CDCl_3$)

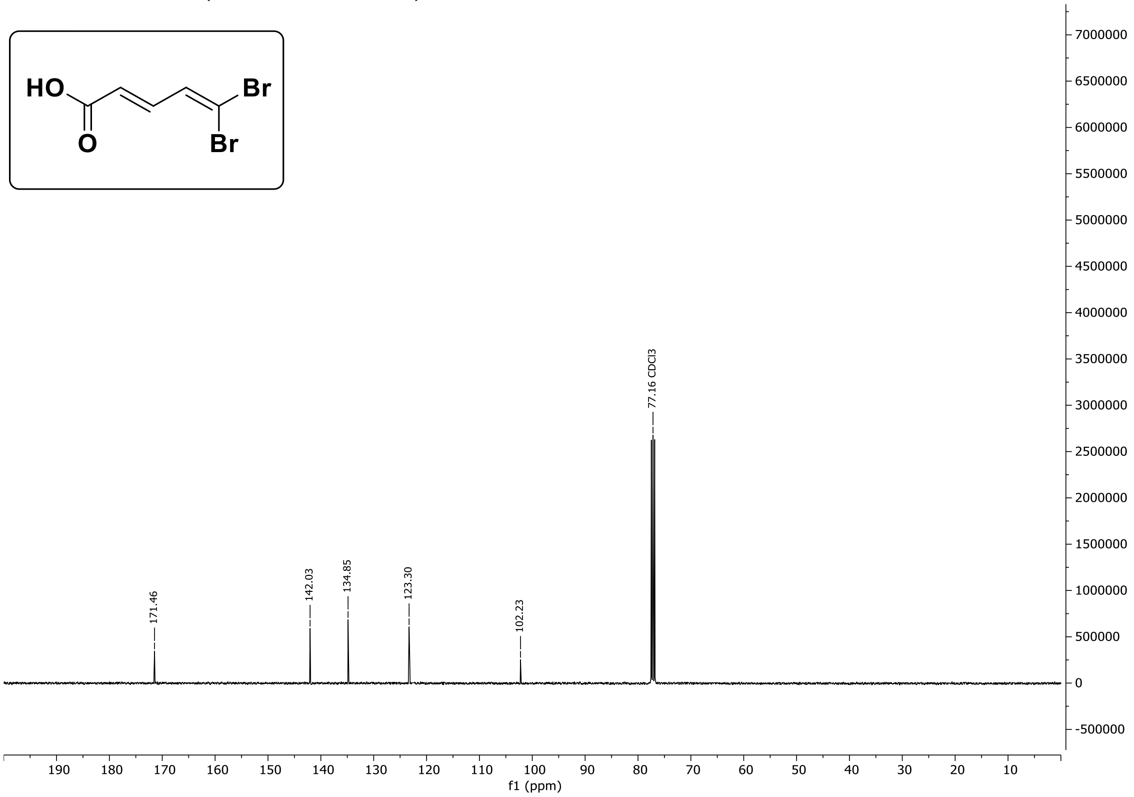

^{1}H-NMR of S8 (400 MHz, $CDCl_3$)

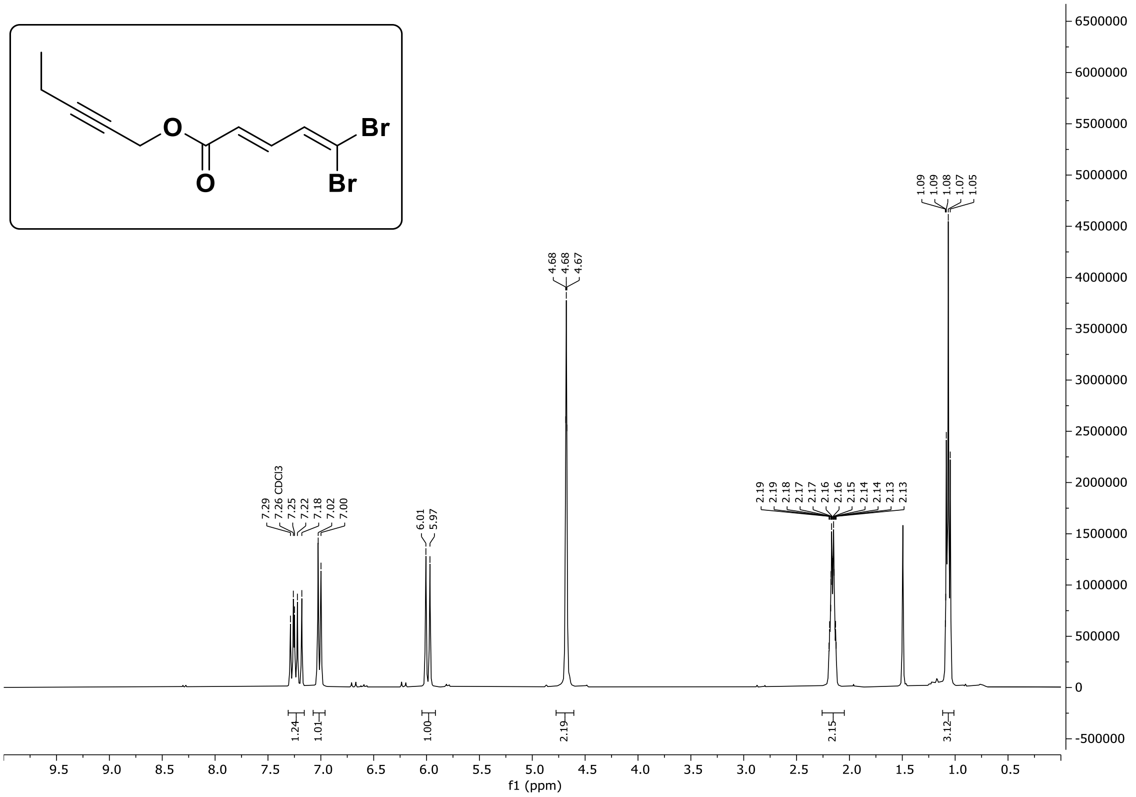

^{13}C-NMR of S8 (100 MHz, $CDCl_3$)

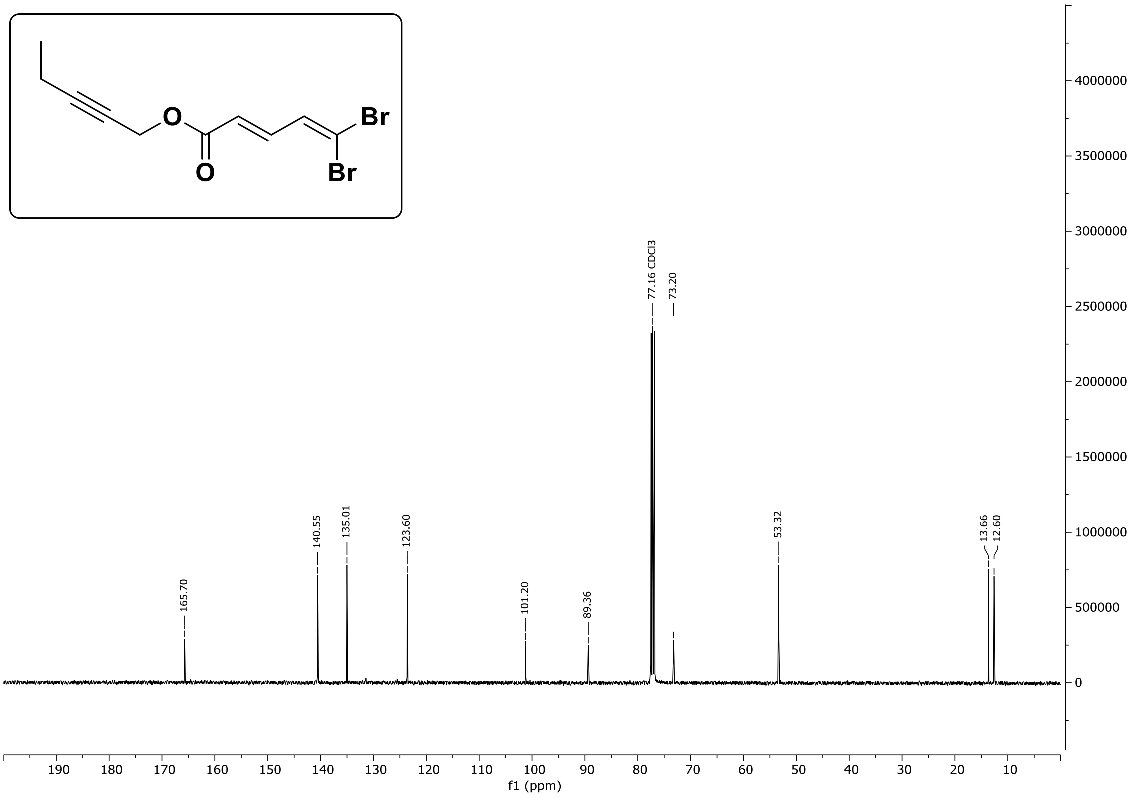

^{1}H-NMR of S9 (400 MHz, $CDCl_3$)

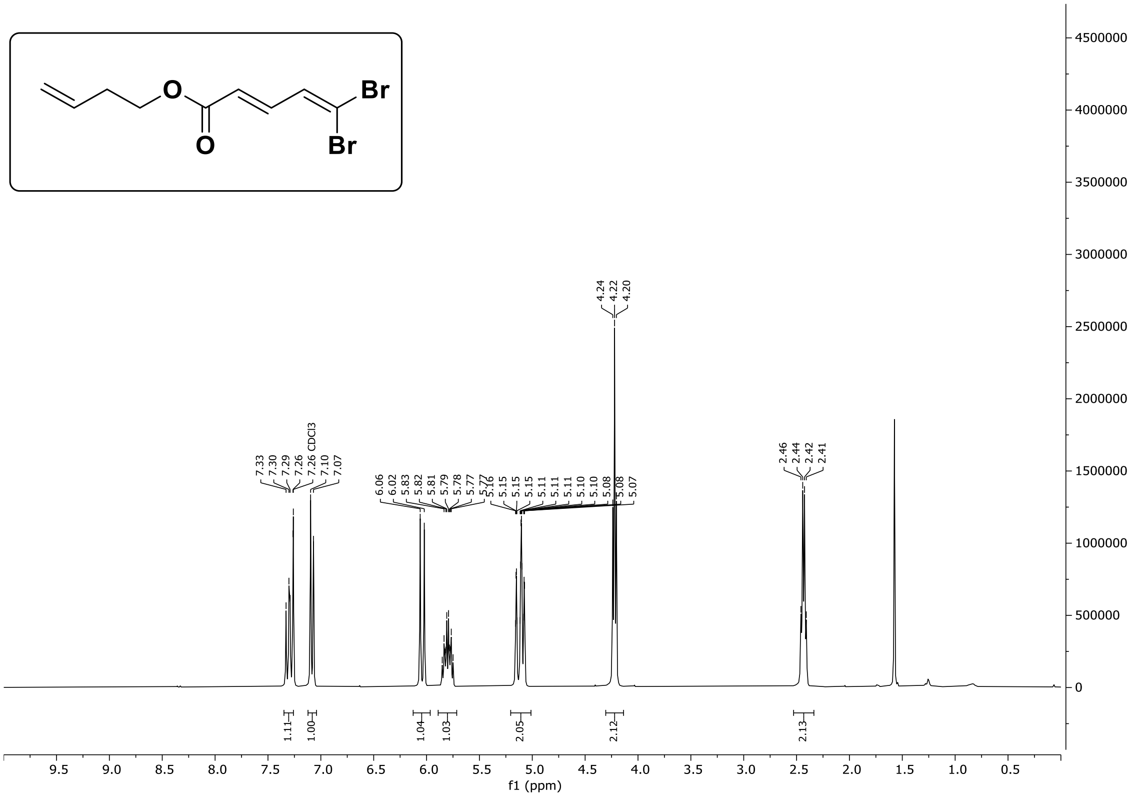

^{13}C-NMR of S9 (100 MHz, $CDCl_3$)

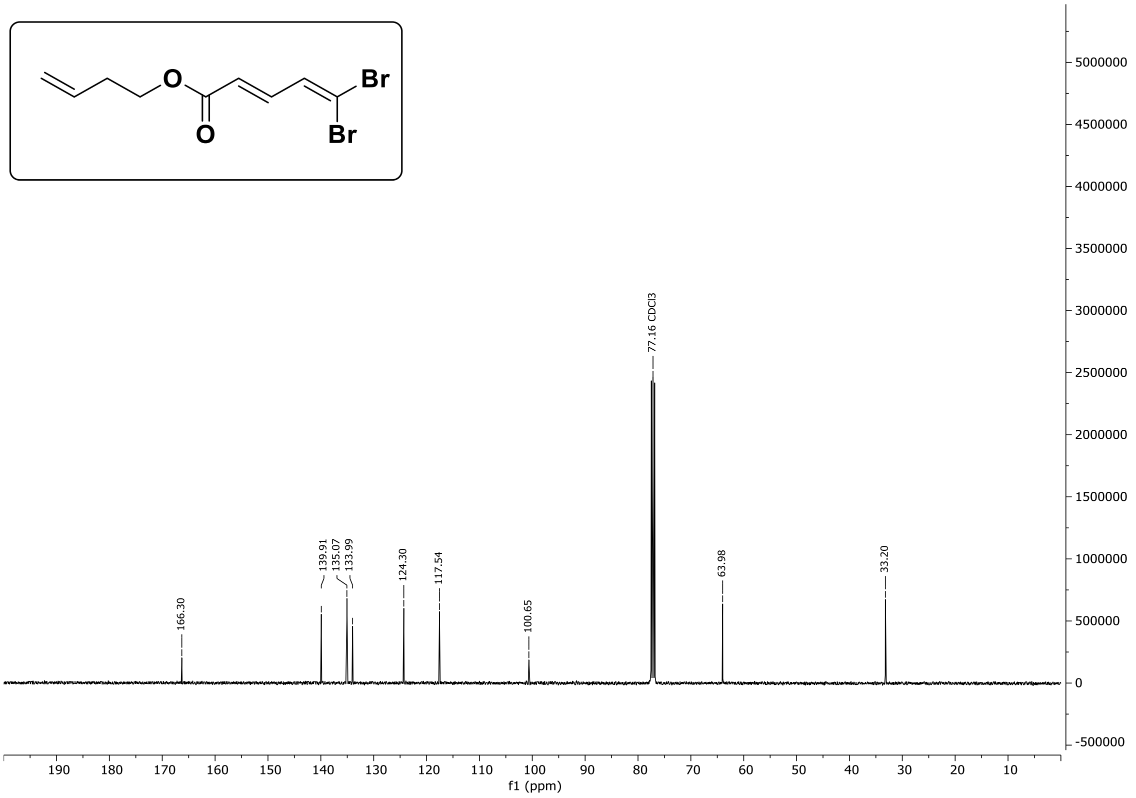

^{1}H-NMR of S10 (400 MHz, $CDCl_3$)

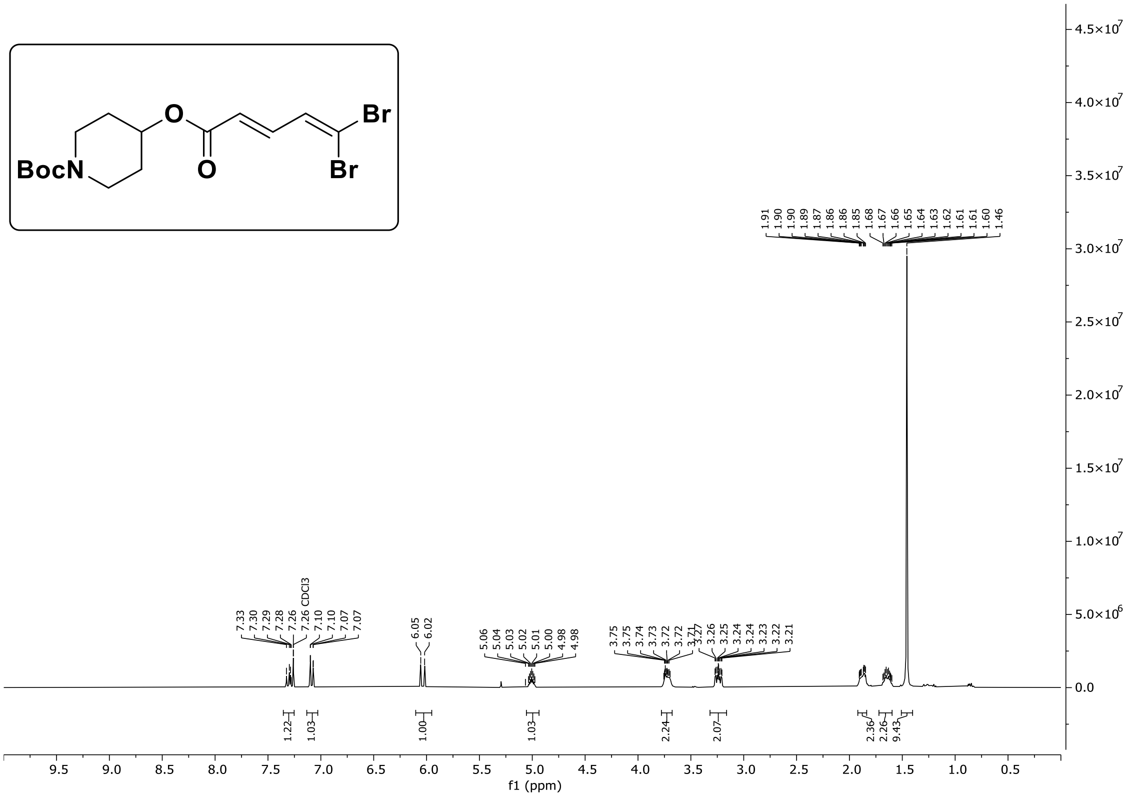

^{13}C-NMR of S10 (100 MHz, $CDCl_3$)

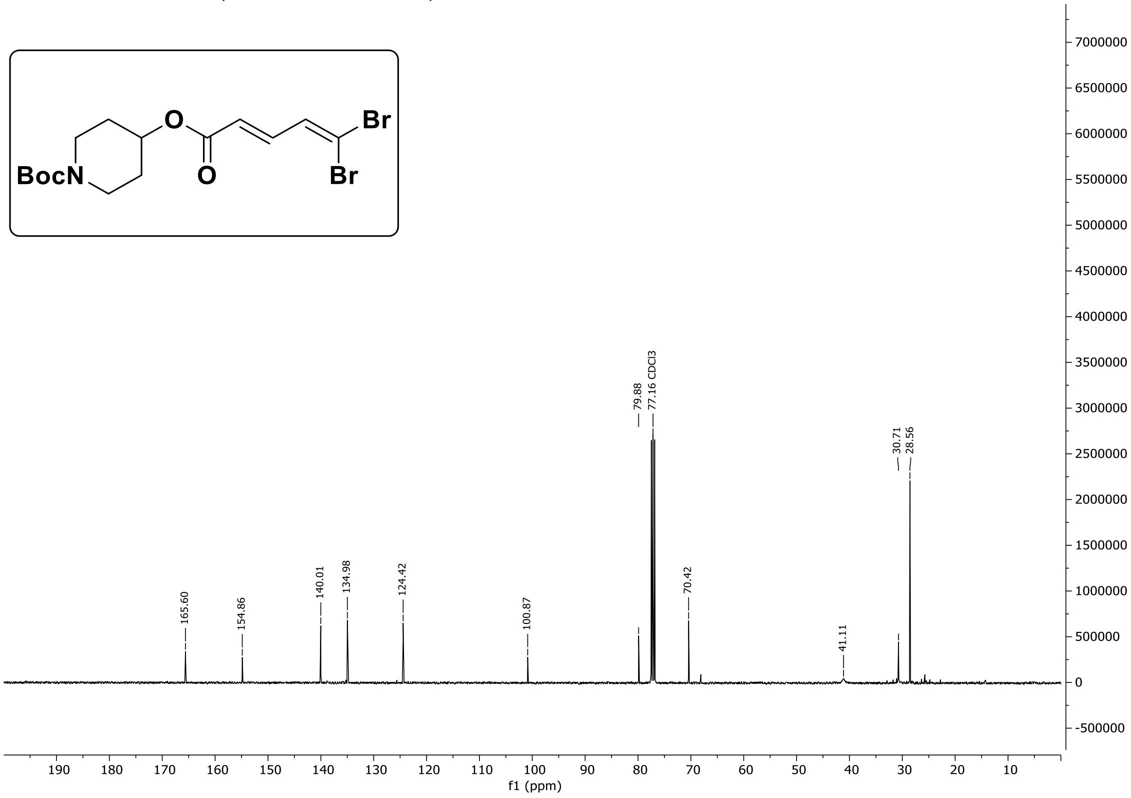

¹H-NMR of S11 (600 MHz, $CDCl_3$)

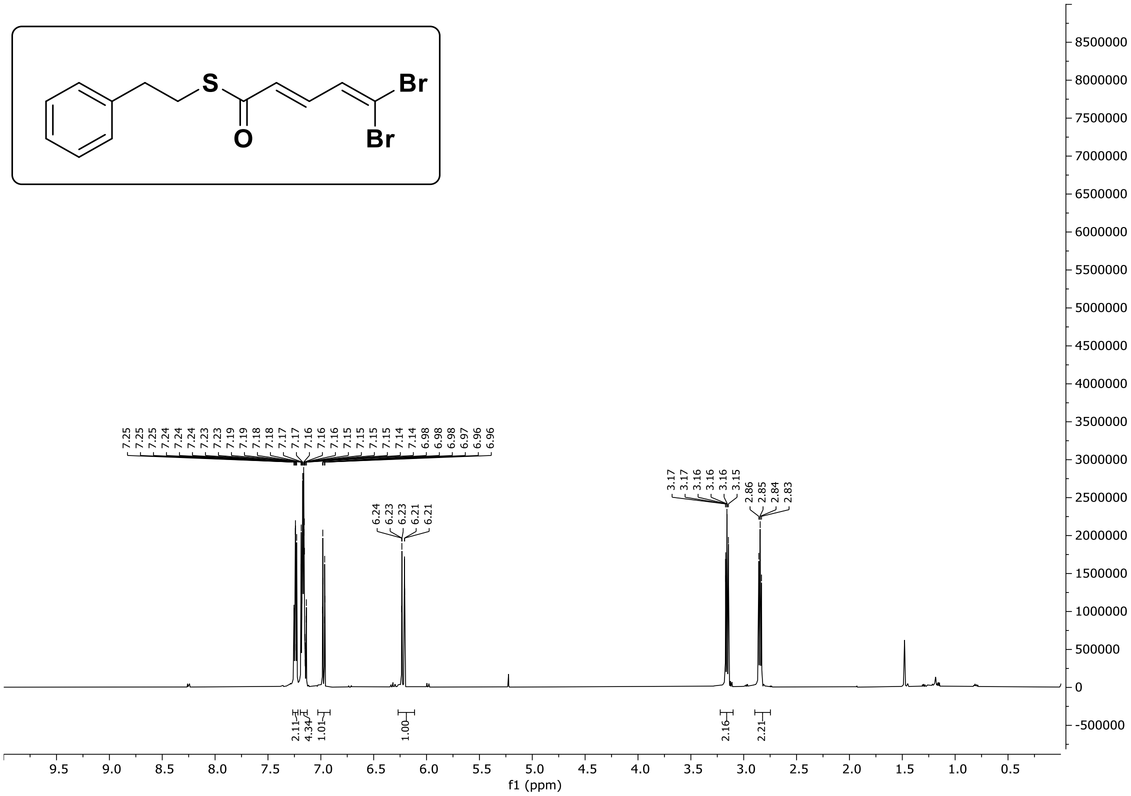

¹³C-NMR of S11 (151 MHz, $CDCl_3$)

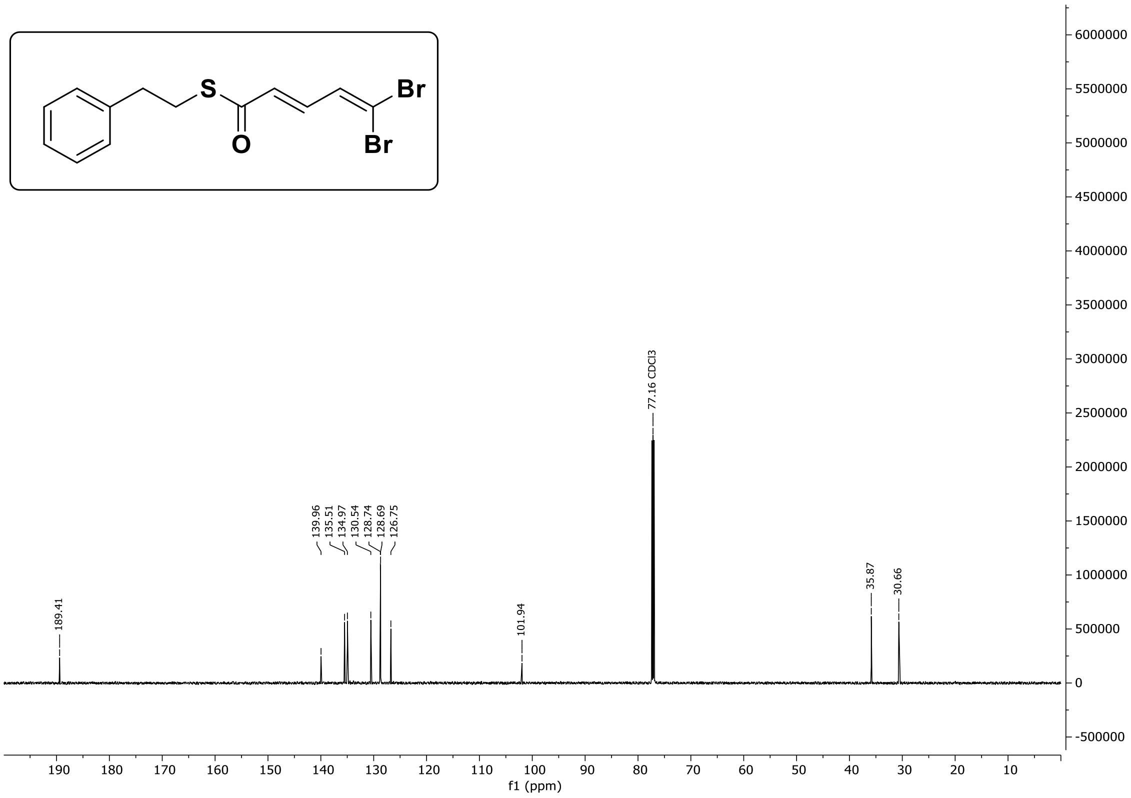

^{1}H-NMR of S12 (400 MHz, $CDCl_3$)

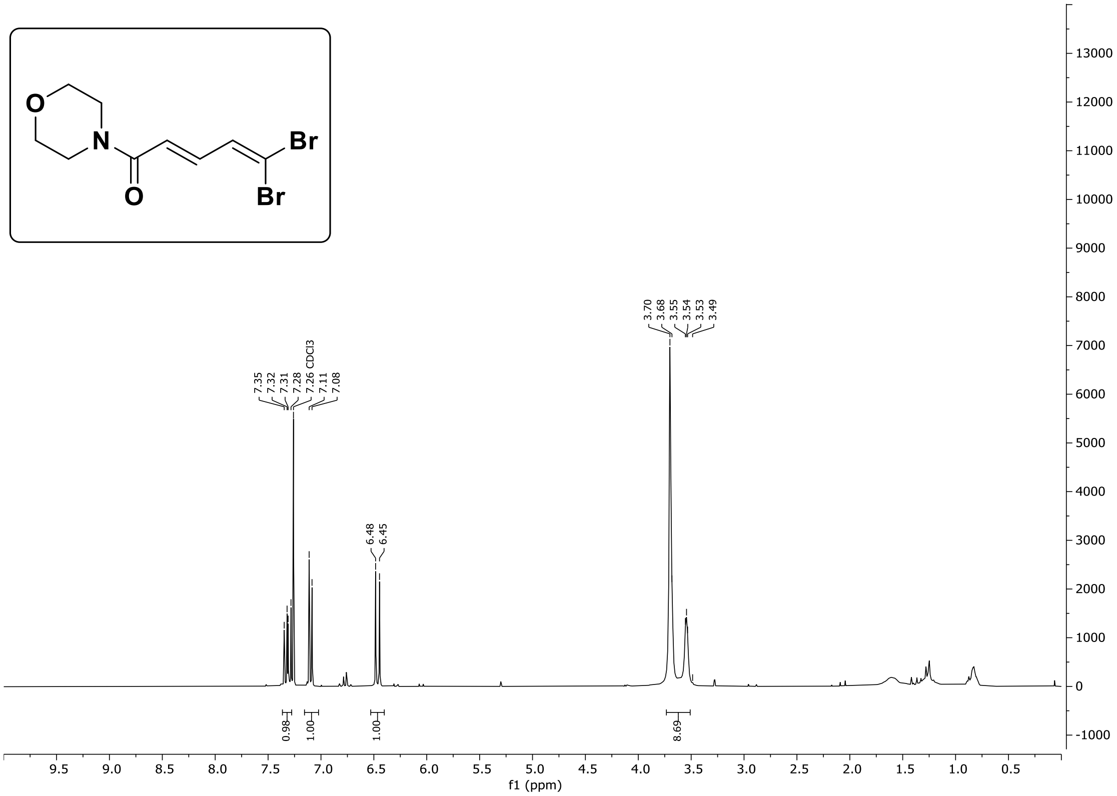

^{13}C-NMR of S12 (101 MHz, $CDCl_3$)

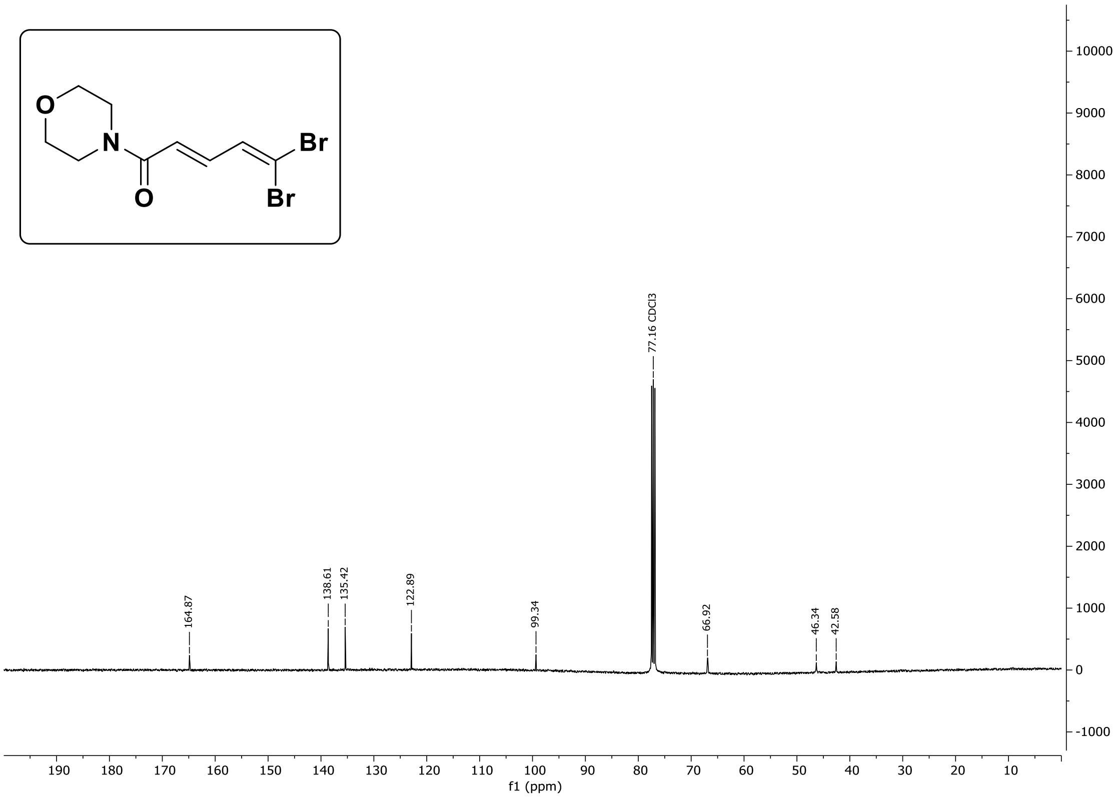

^{1}H-NMR of S13 (400 MHz, $CDCl_3$)

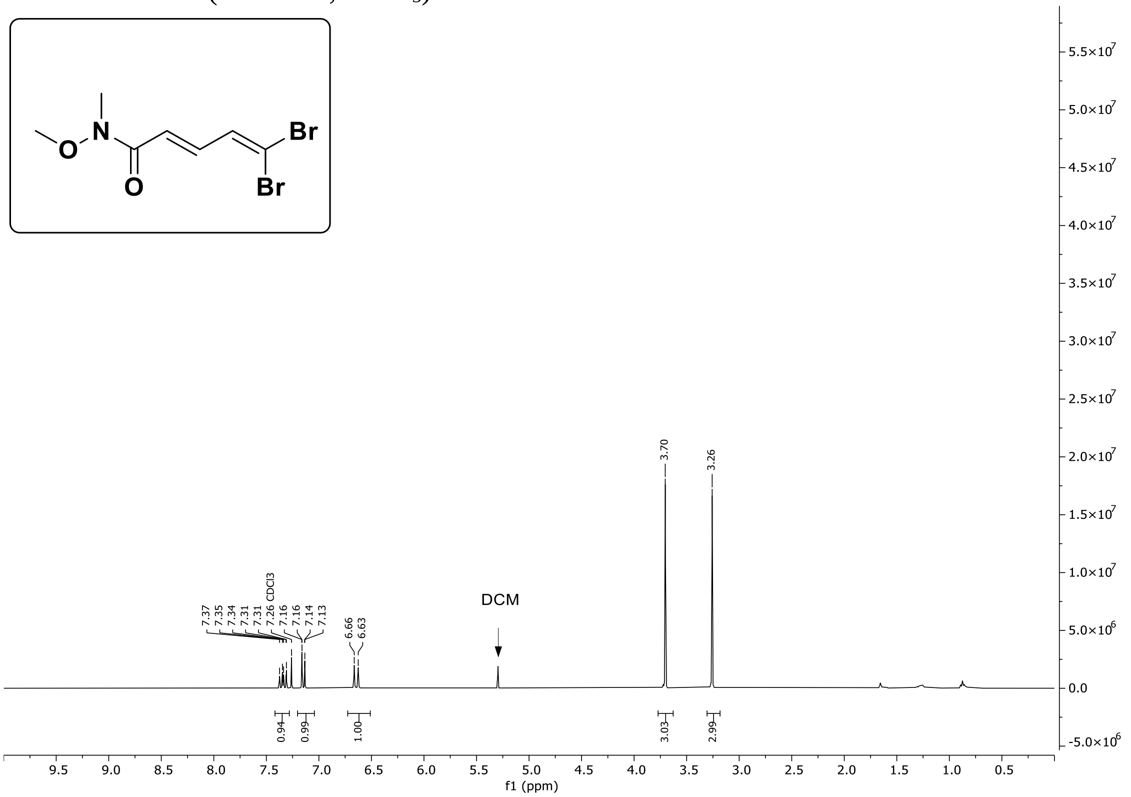

^{13}C-NMR of S13 (100 MHz, $CDCl_3$)

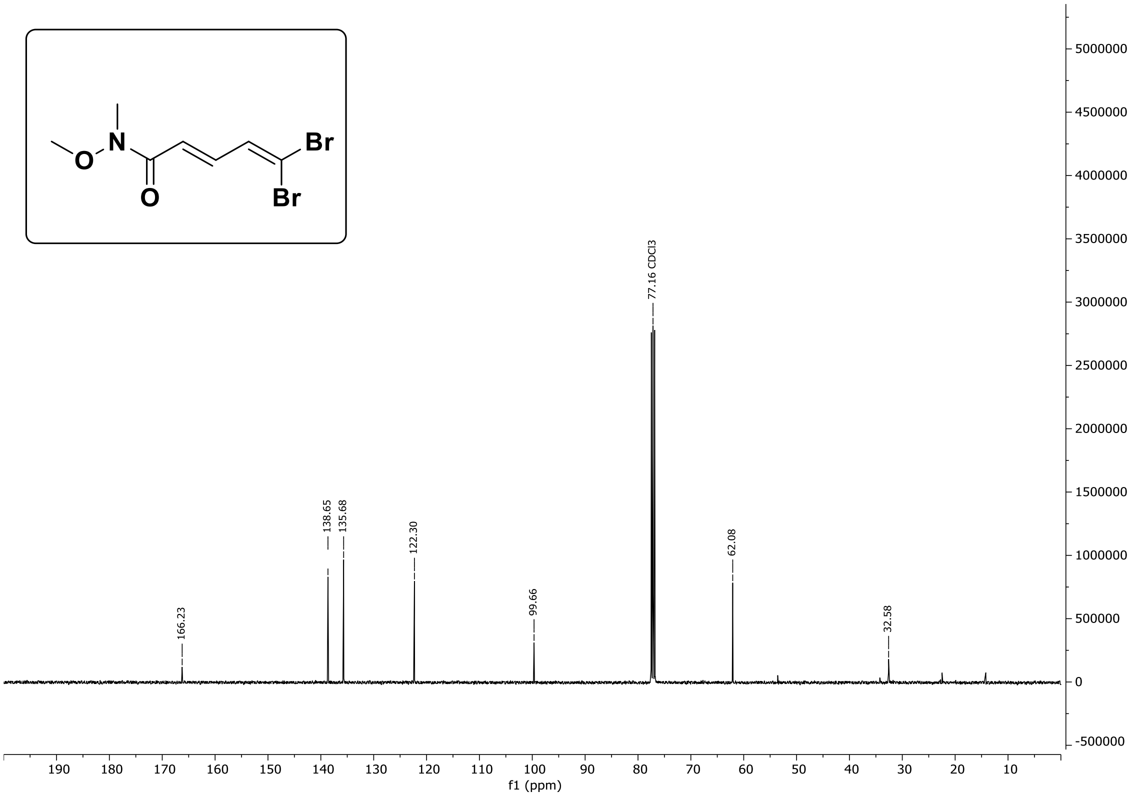

^{1}H-NMR of S14 (400 MHz, $CDCl_3$)

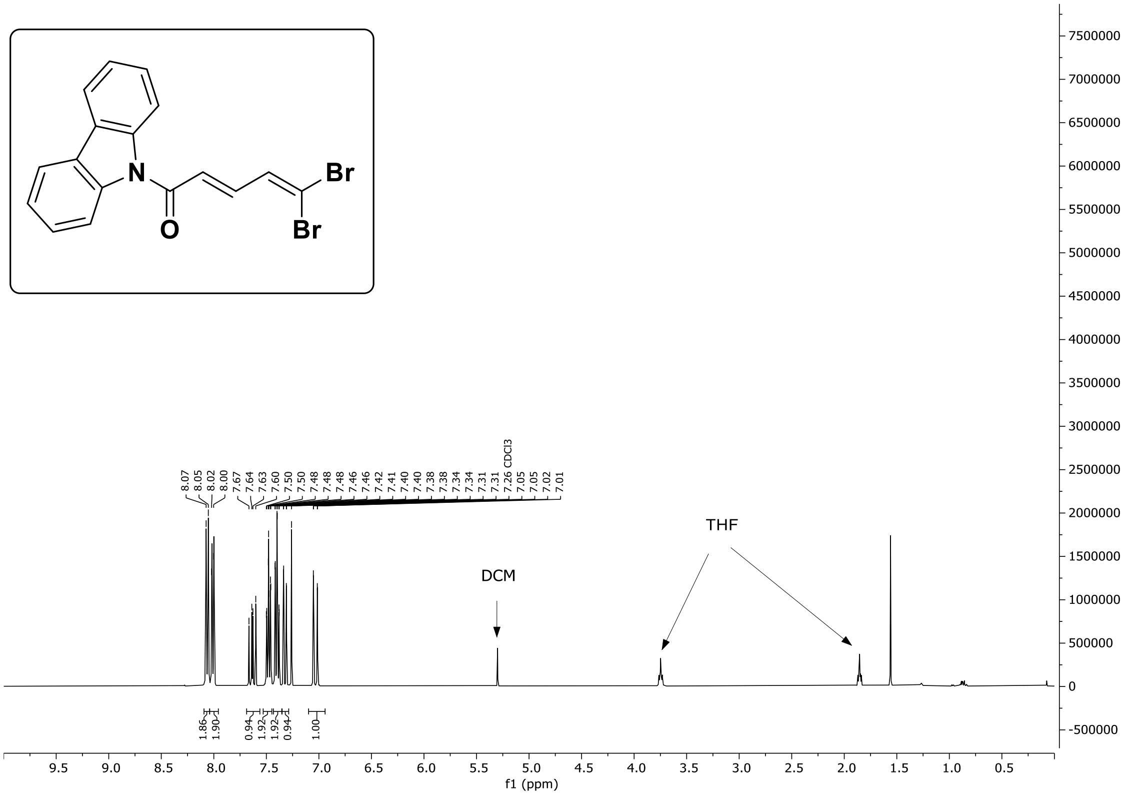

^{13}C-NMR of S14 (100 MHz, $CDCl_3$)

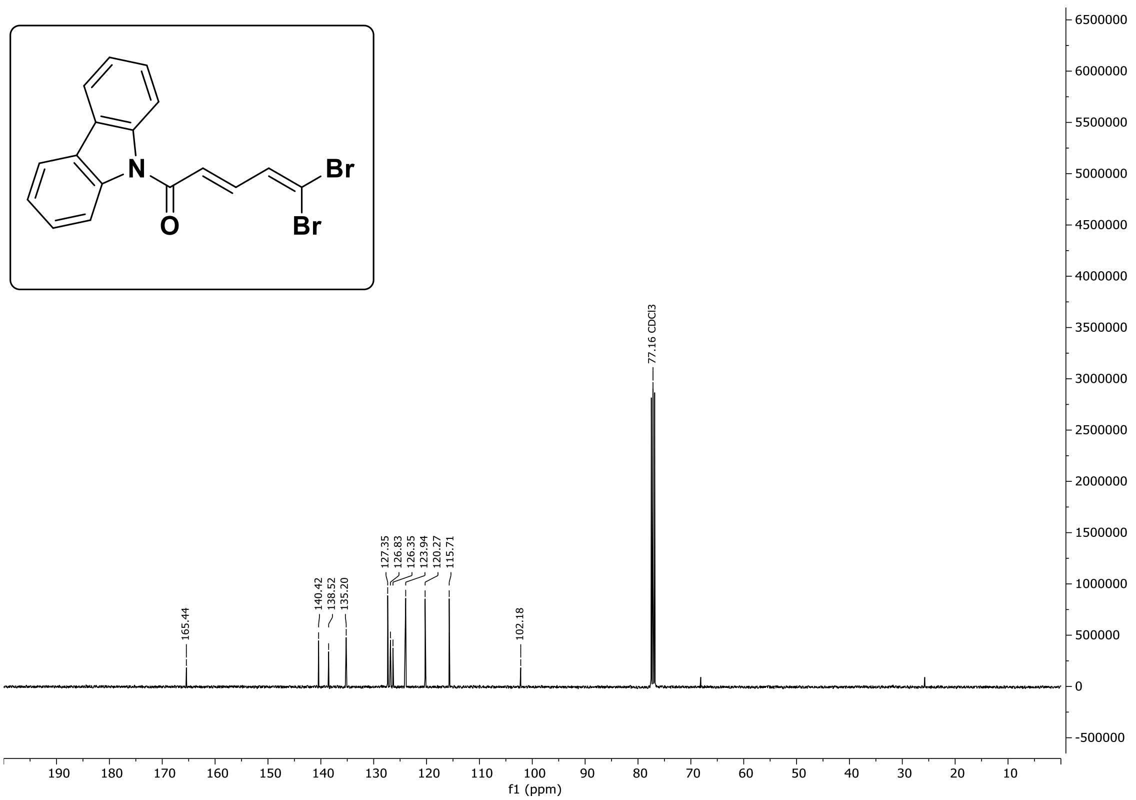

^{1}H-NMR of S15 (400 MHz, $CDCl_3$)

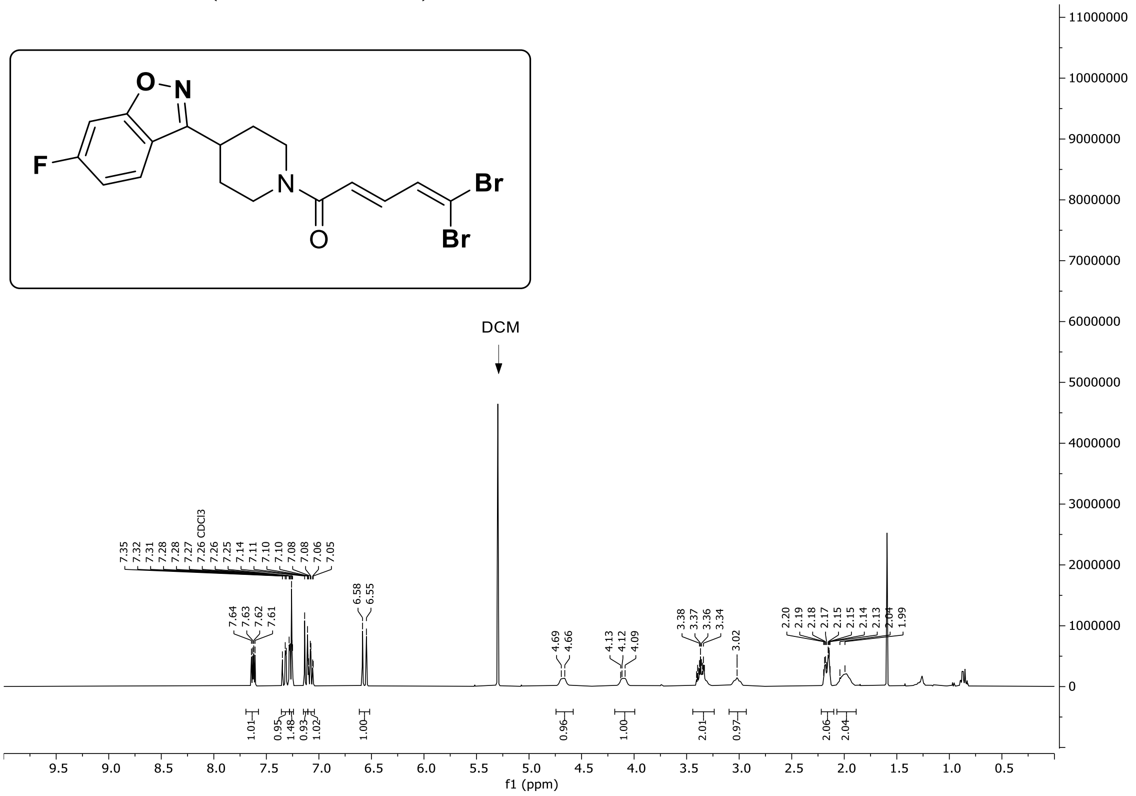

^{13}C-NMR of S15 (100 MHz, $CDCl_3$)

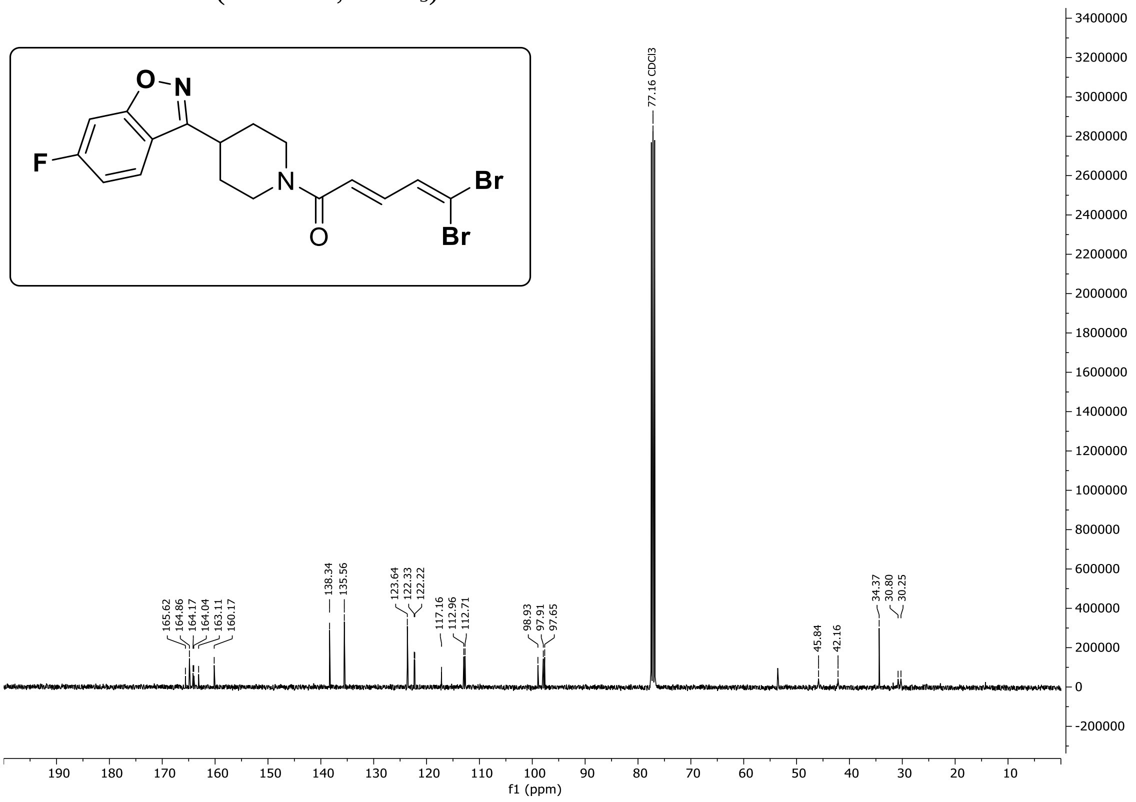

^{19}F-NMR of S15 (376 MHz, $CDCl_3$)

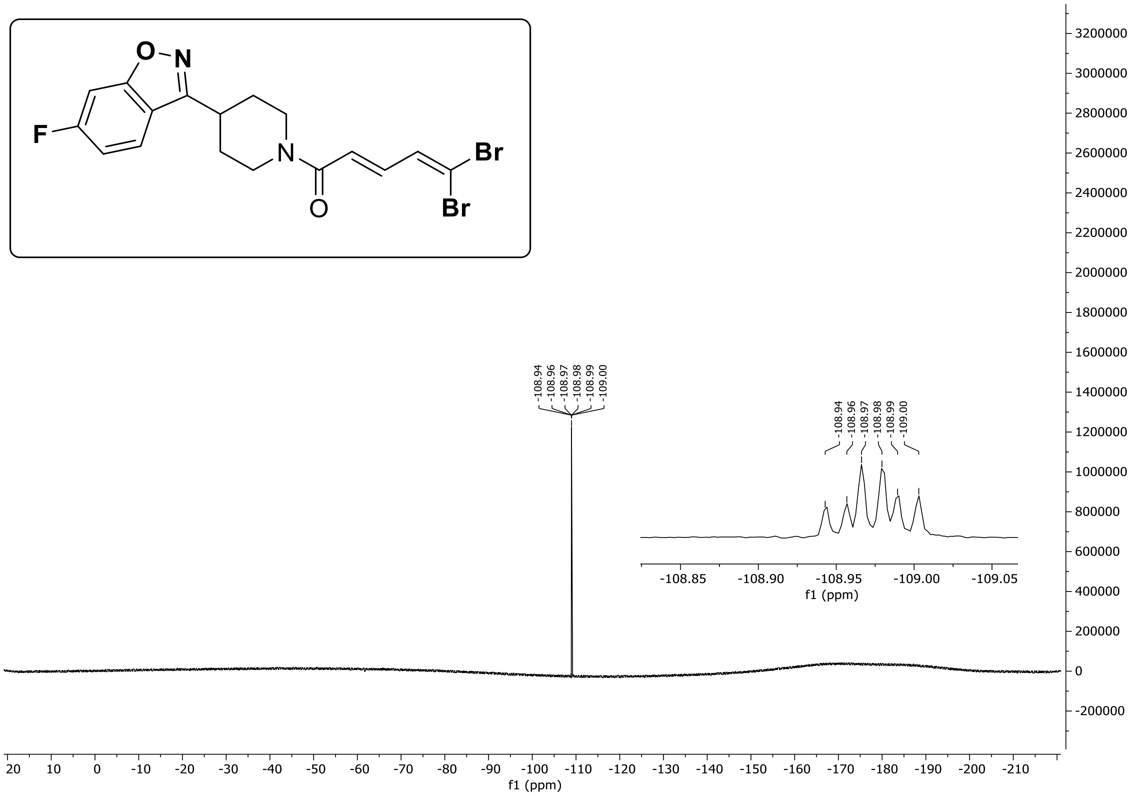

^{1}H-NMR of S16 (400 MHz, $CDCl_3$)

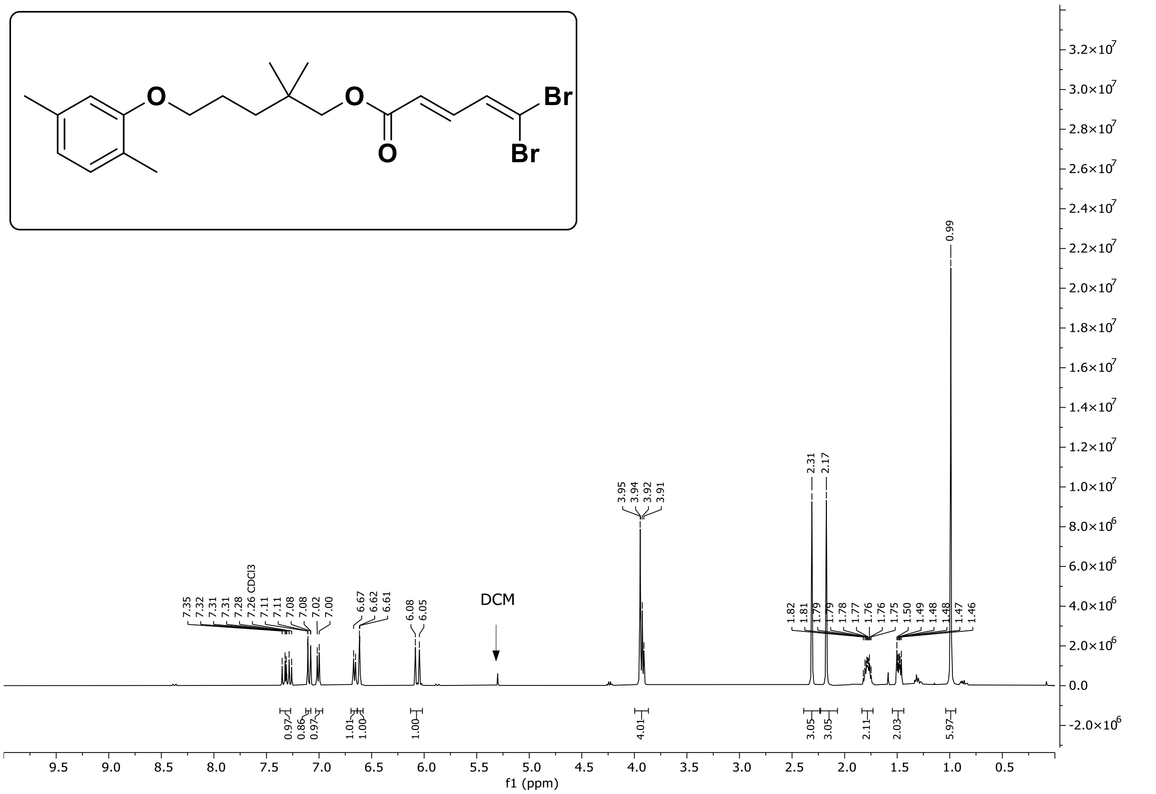

^{13}**C-NMR of S16** (100 MHz, $CDCl_3$)

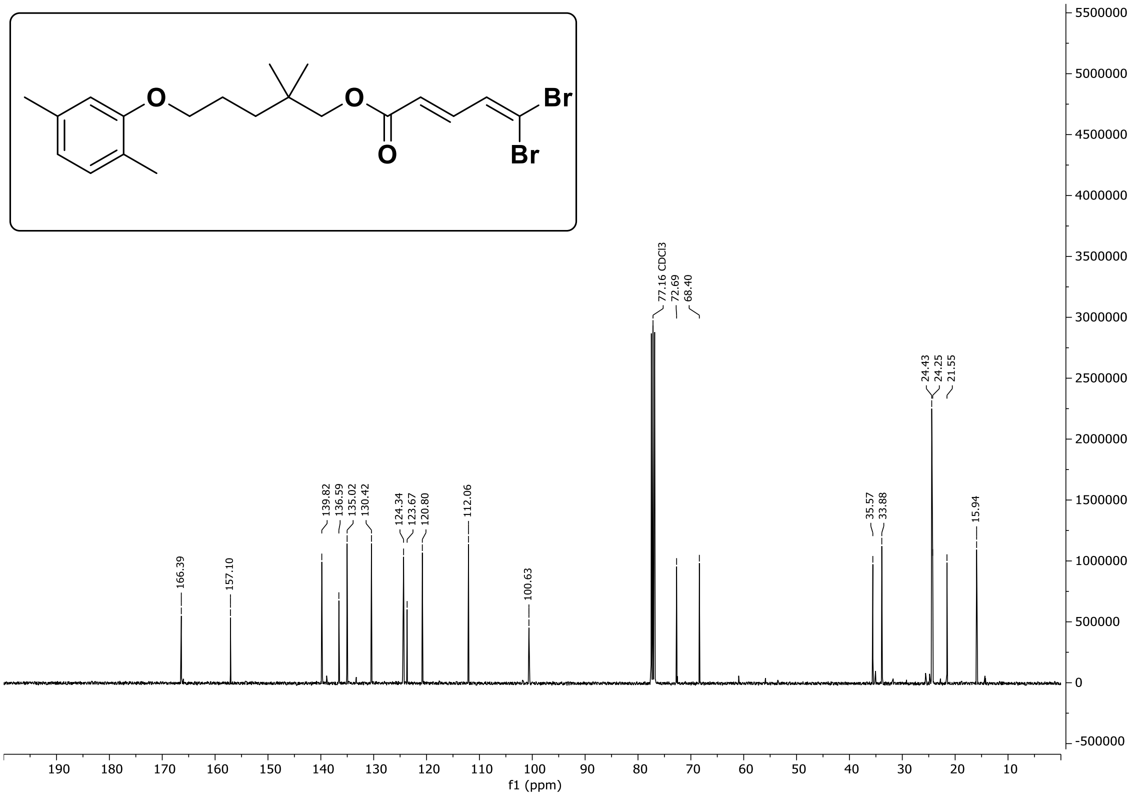

^{1}**H-NMR of S17** (700 MHz, $CDCl_3$)

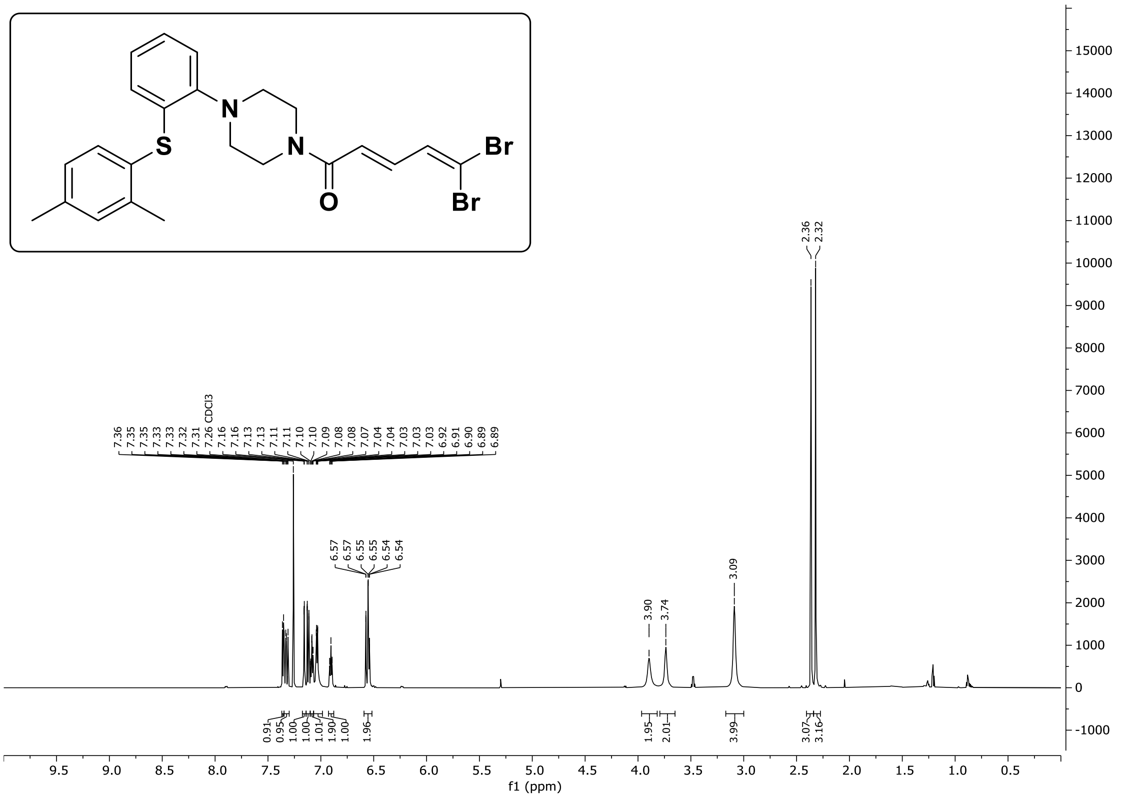

^{13}C-NMR of S17 (176 MHz, $CDCl_3$)

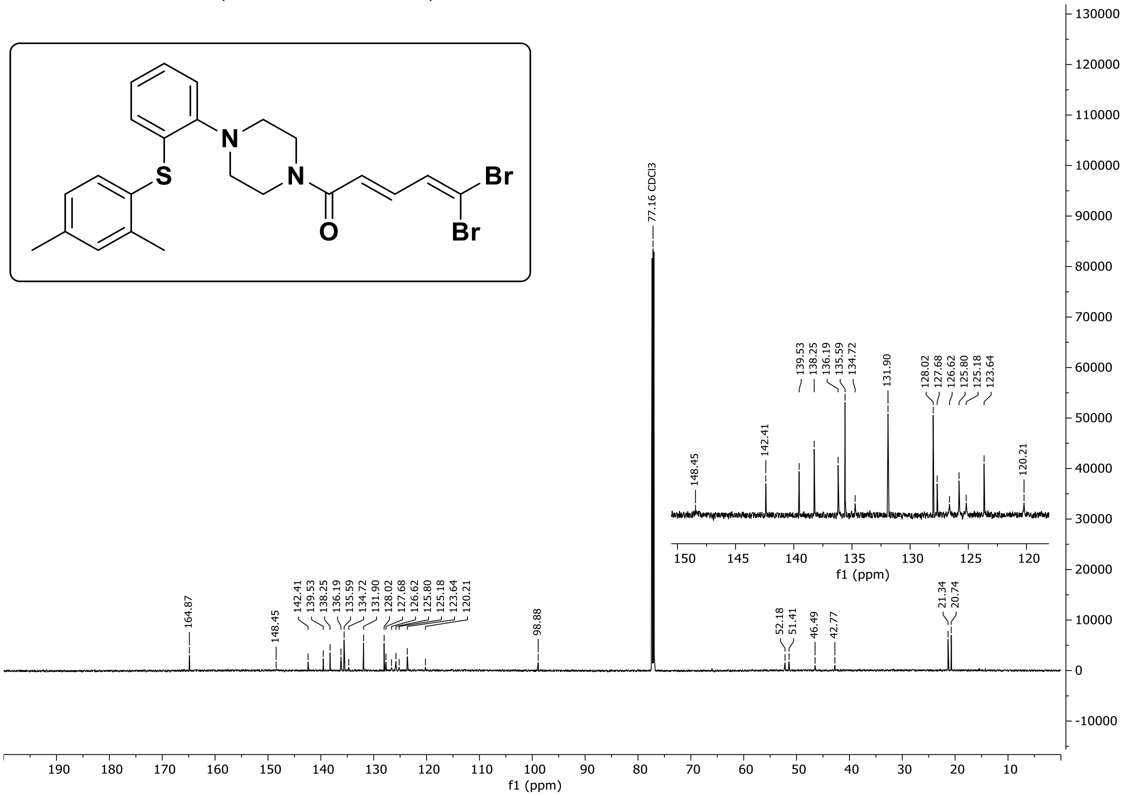

^{1}H-NMR of 1 (400 MHz, $CDCl_3$)

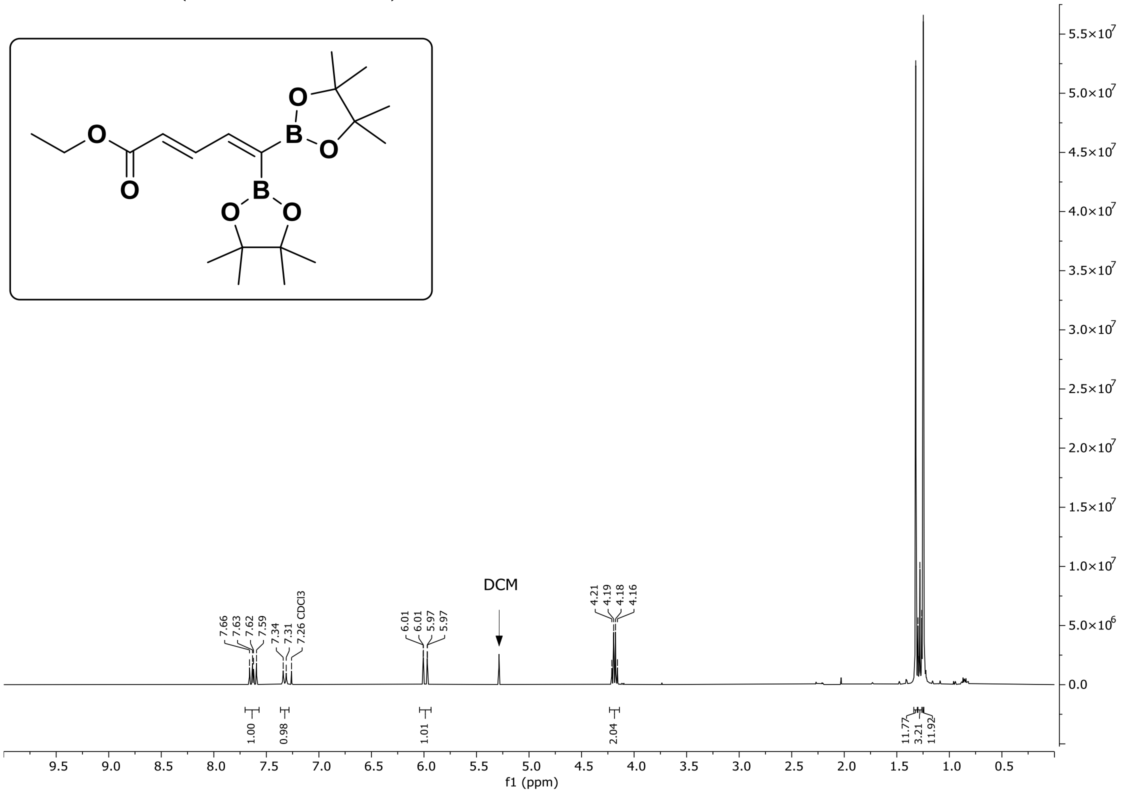

^{13}C-NMR of 1 (100 MHz, $CDCl_3$)

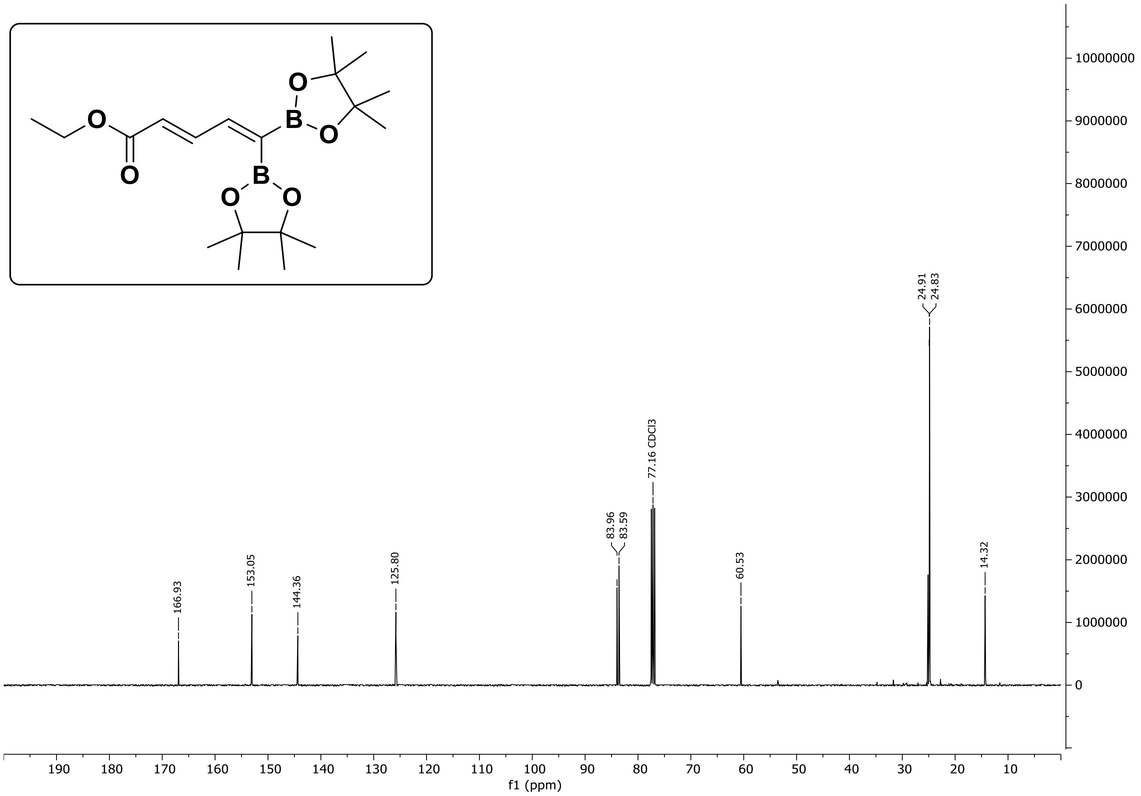

^{11}B NMR of 1 (128 MHz, $CDCl_3$)

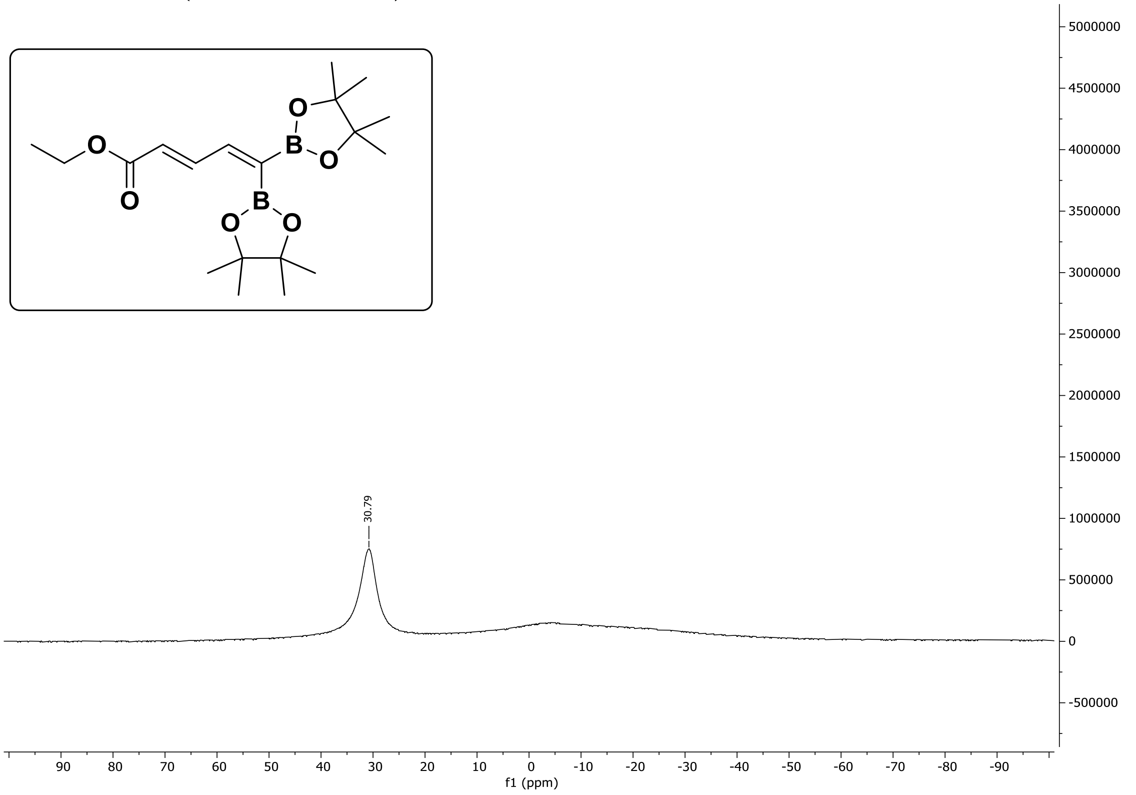

^{1}H-NMR of 2 (400 MHz, $CDCl_3$)

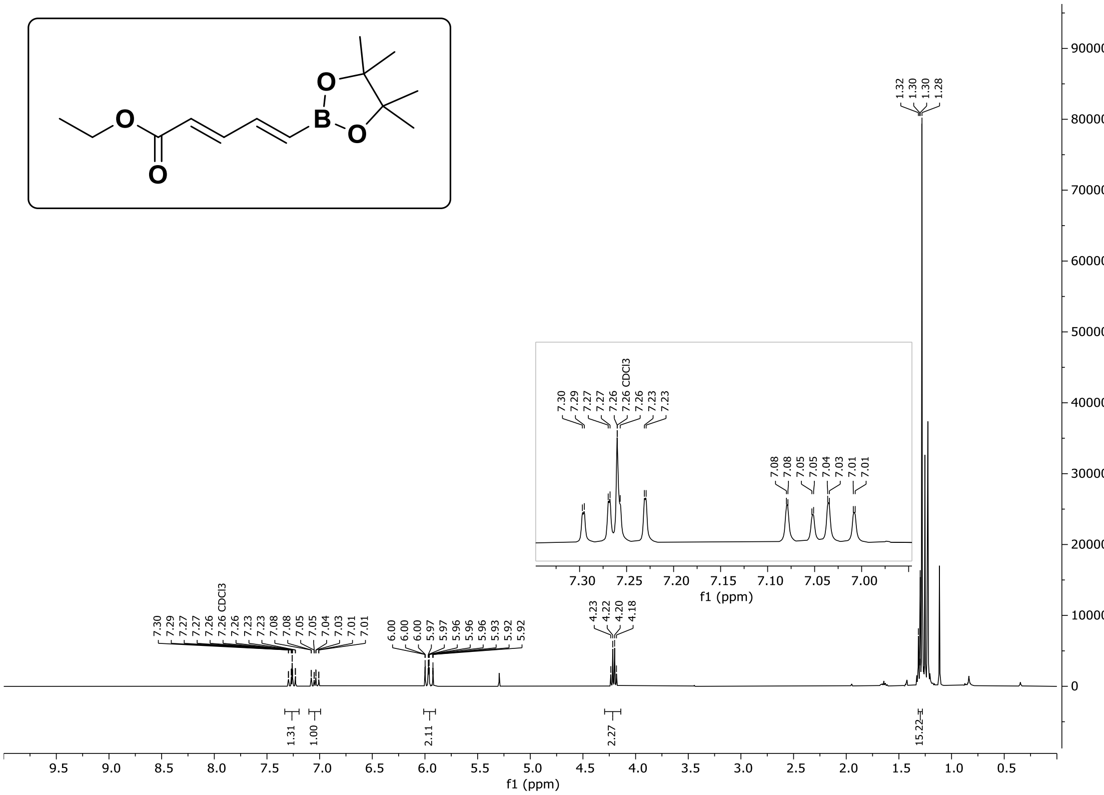

^{1}H-NMR of 3 (400 MHz, CD_3CN)

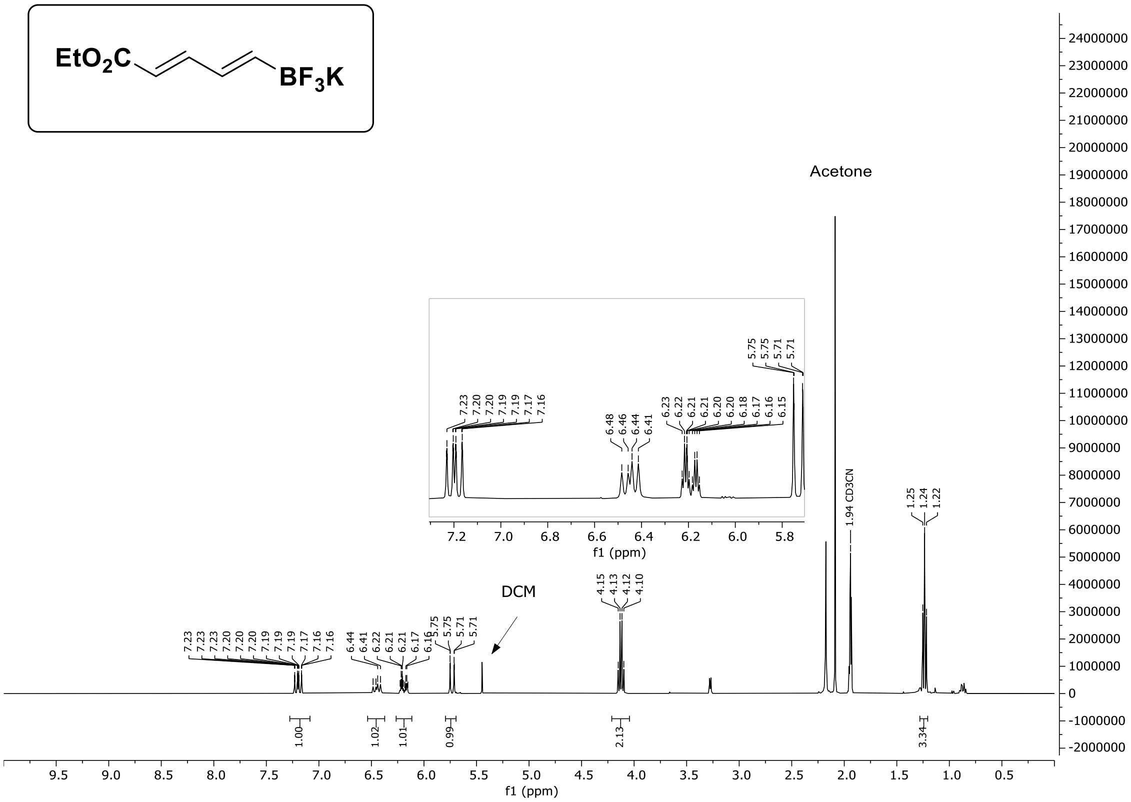

^{1}H-NMR of 3 (400 MHz, Acetone)

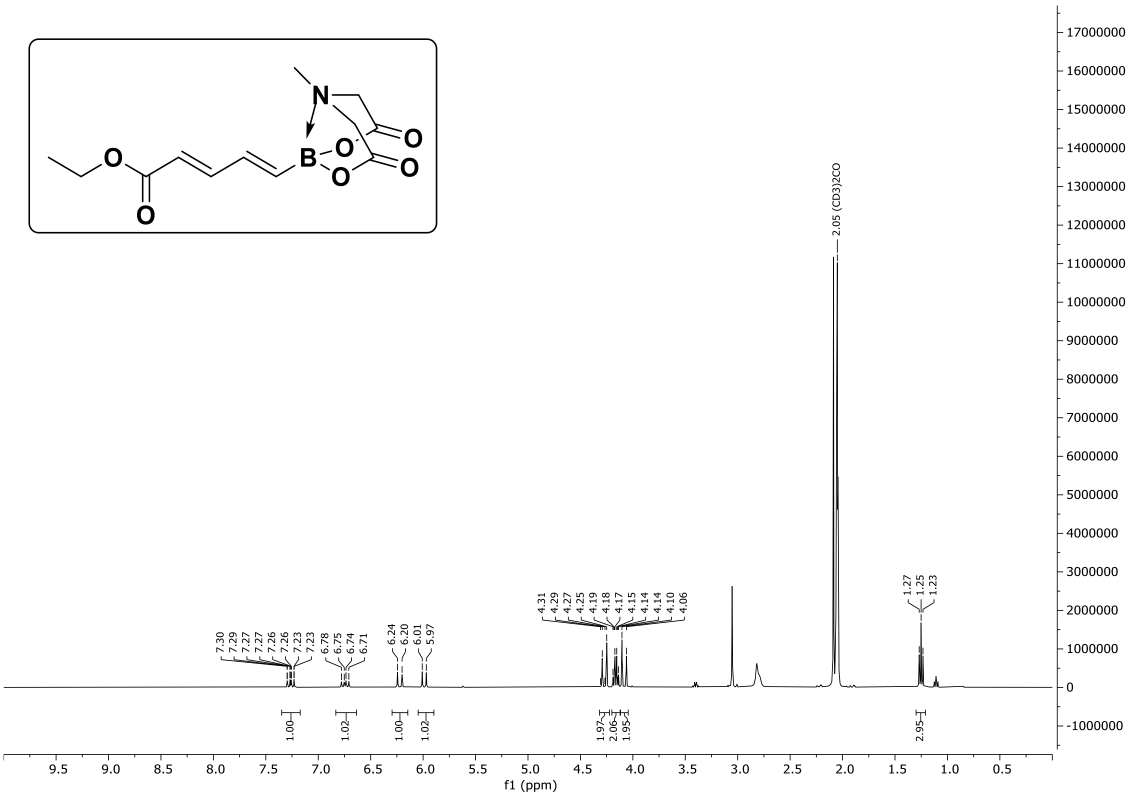

^{1}H-NMR of S18 (400 MHz, $CDCl_3$)

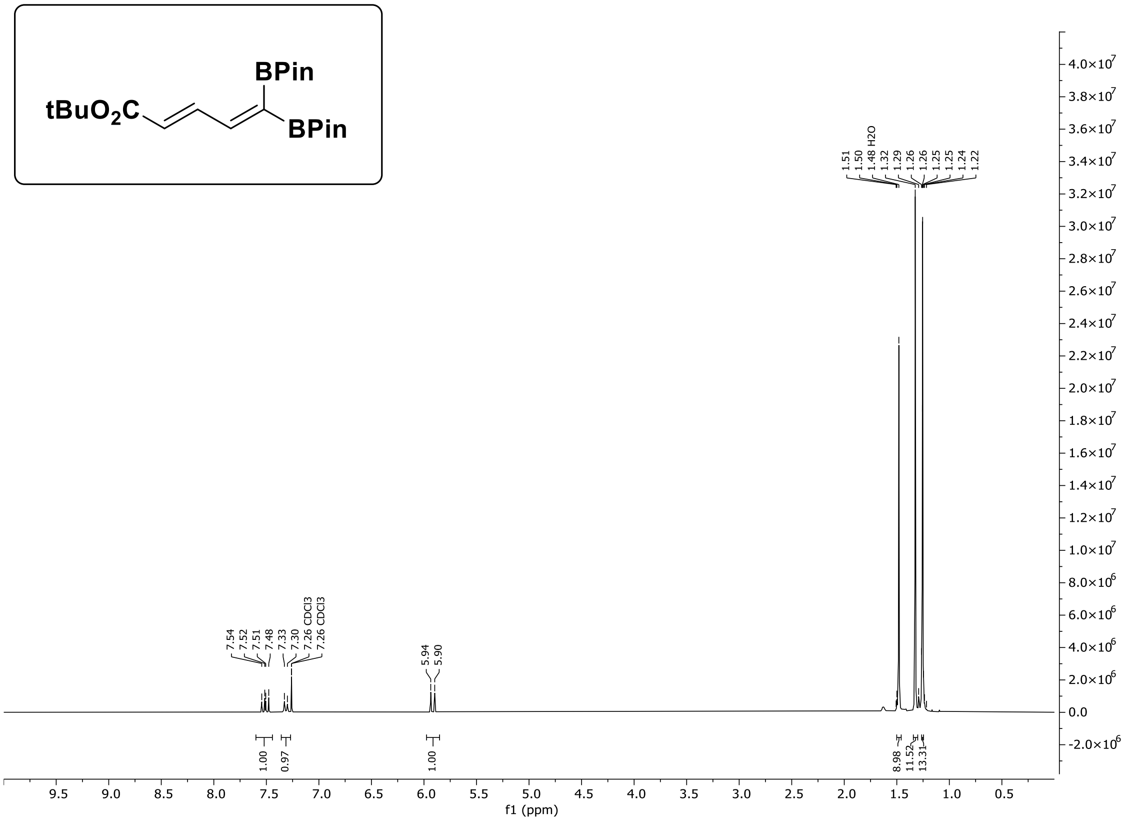

^{13}C-NMR of S18 (151 MHz, $CDCl_3$)

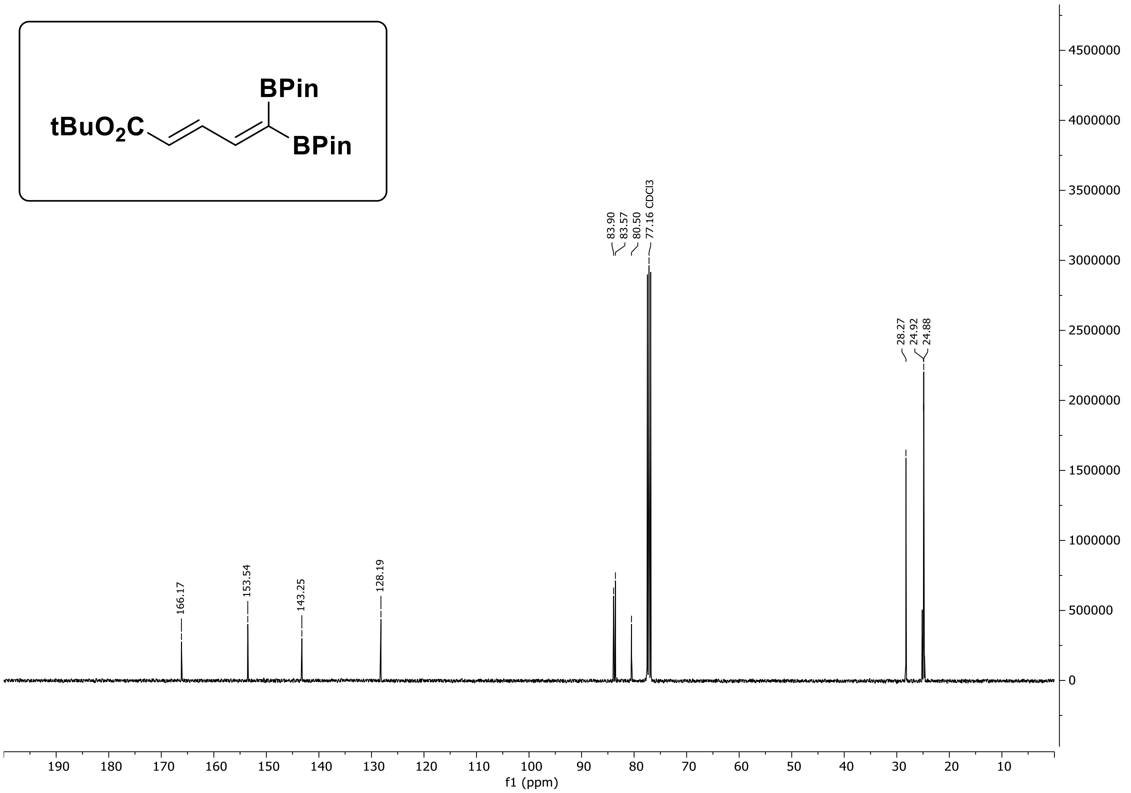

^{11}B NMR of S18 (128 MHz, $CDCl_3$)

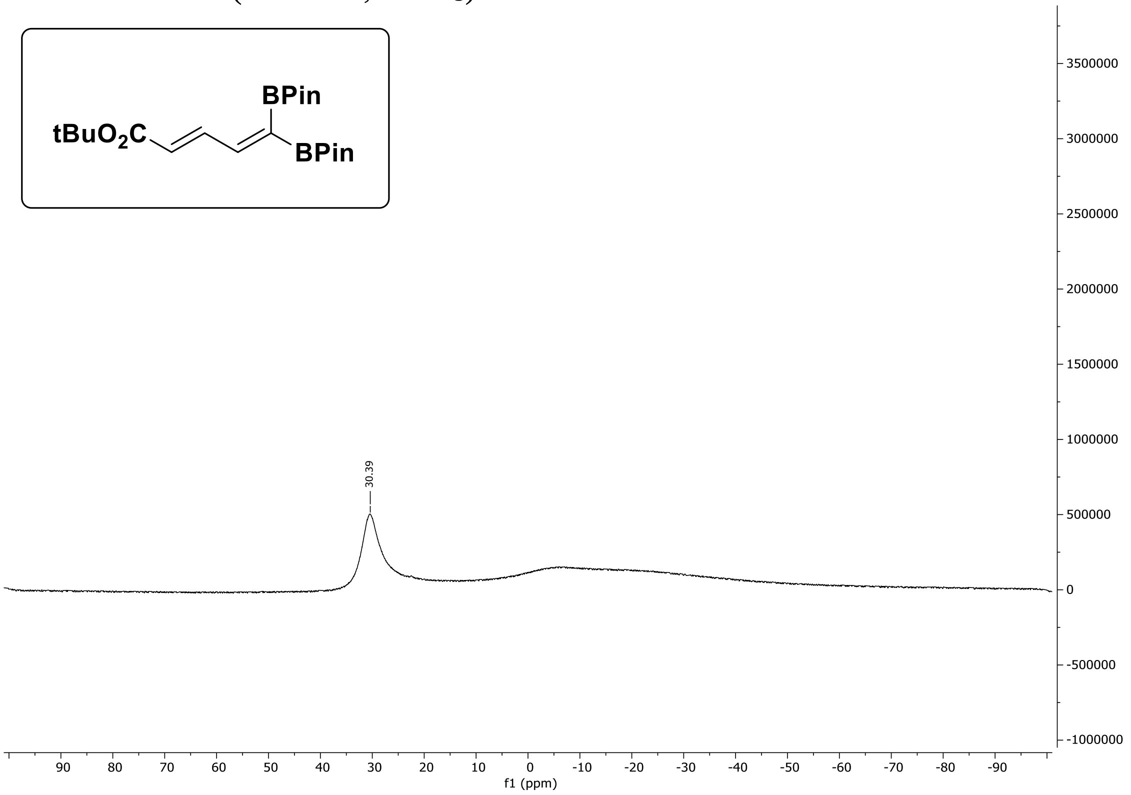

^{1}H-NMR of S19 (400 MHz, $CDCl_3$)

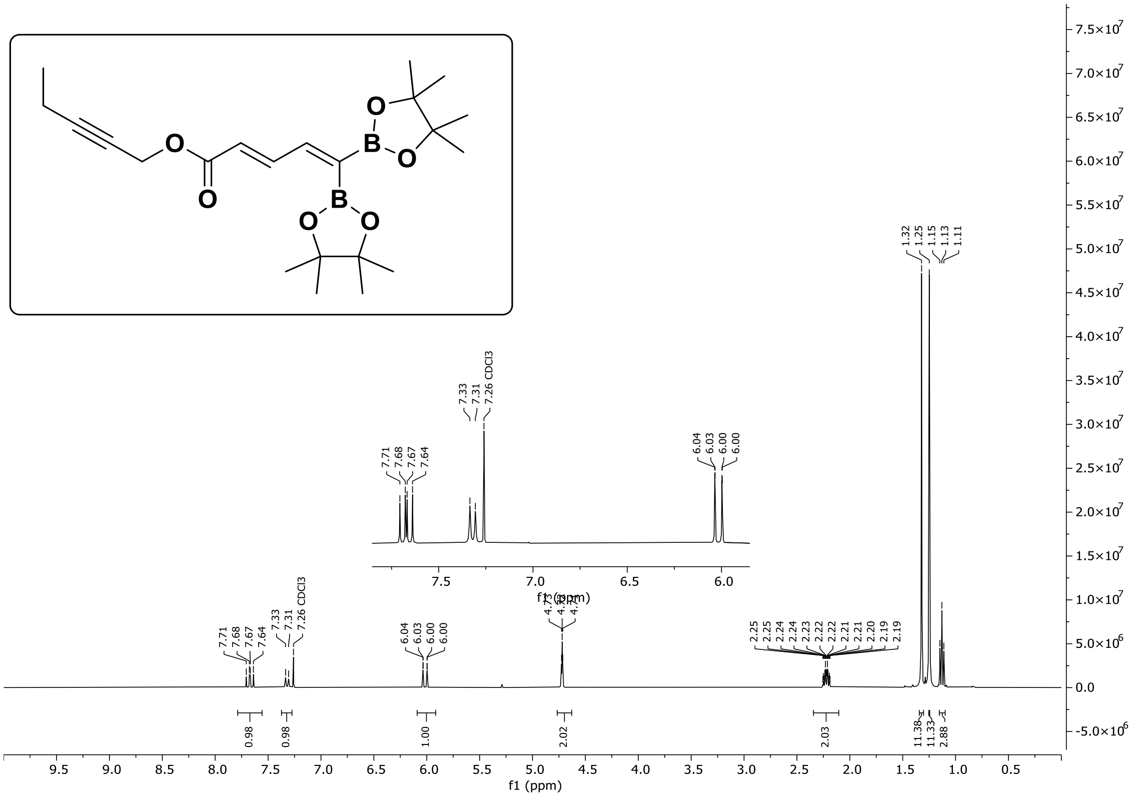

^{13}C-NMR of S19 (100 MHz, $CDCl_3$)

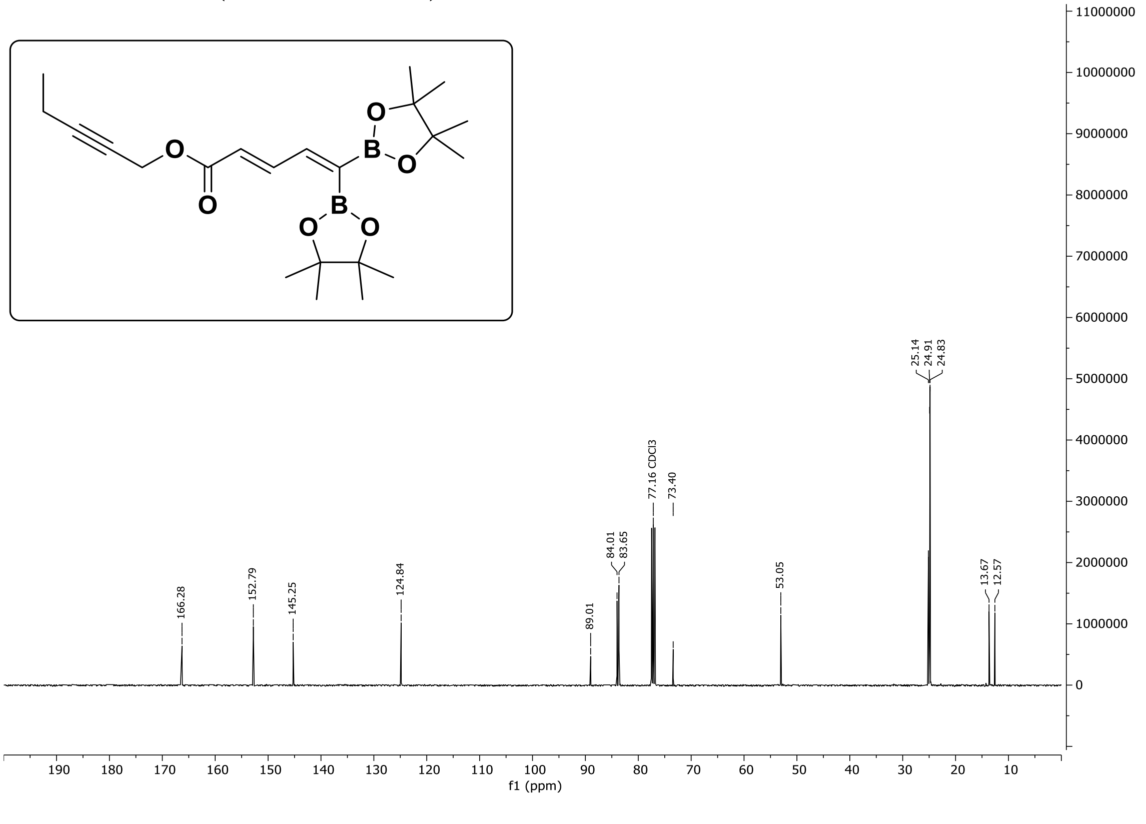

^{11}B NMR of S19 (128 MHz, $CDCl_3$)

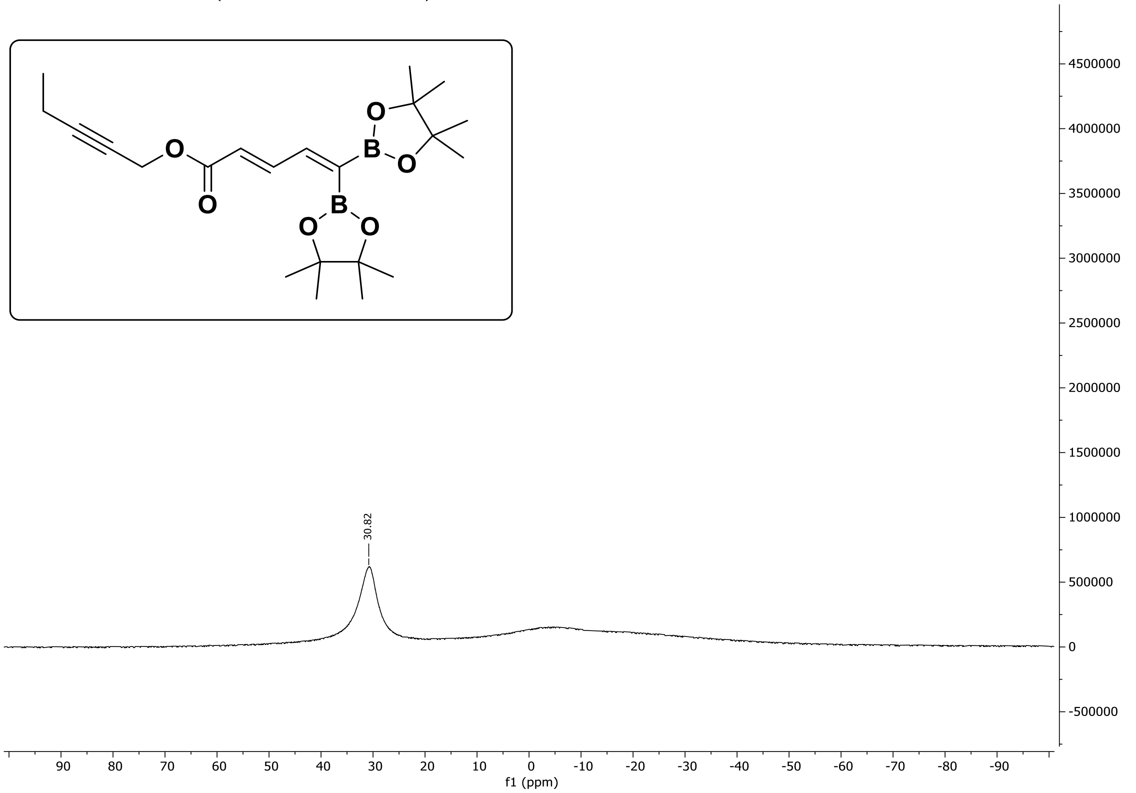

^{1}H-NMR of S20 (400 MHz, $CDCl_3$)

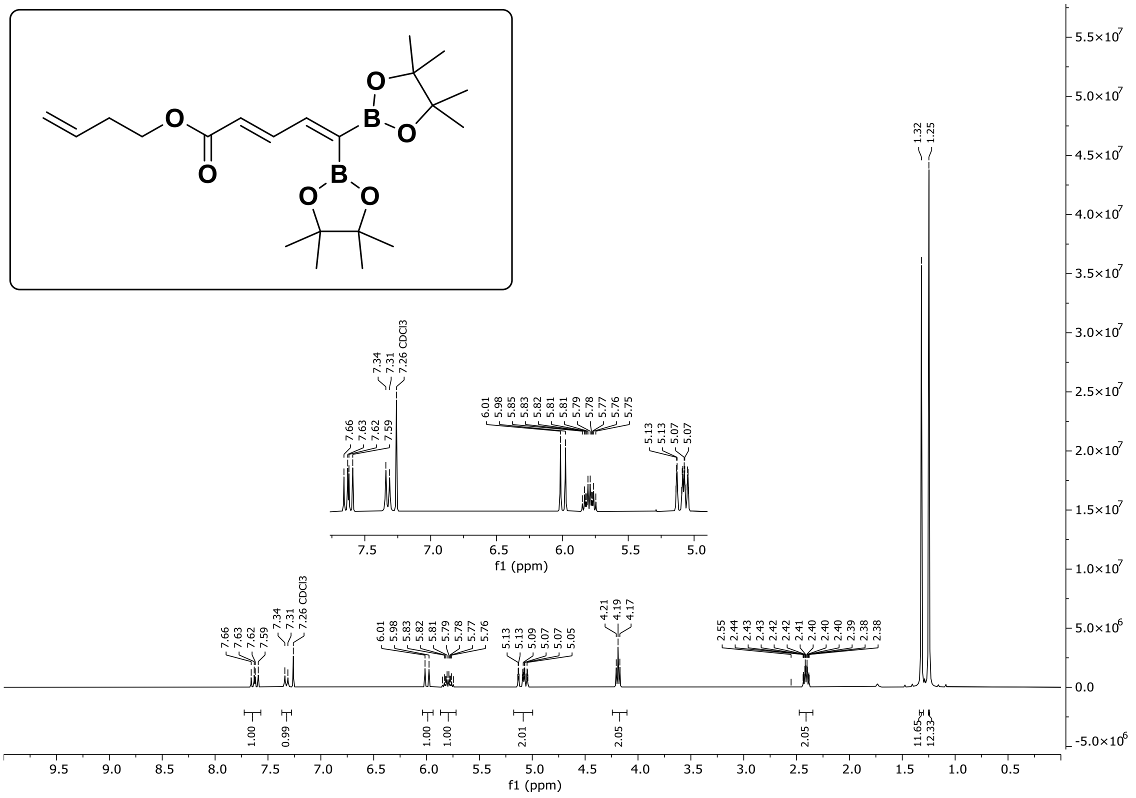

^{13}C-NMR of S20 (100 MHz, $CDCl_3$)

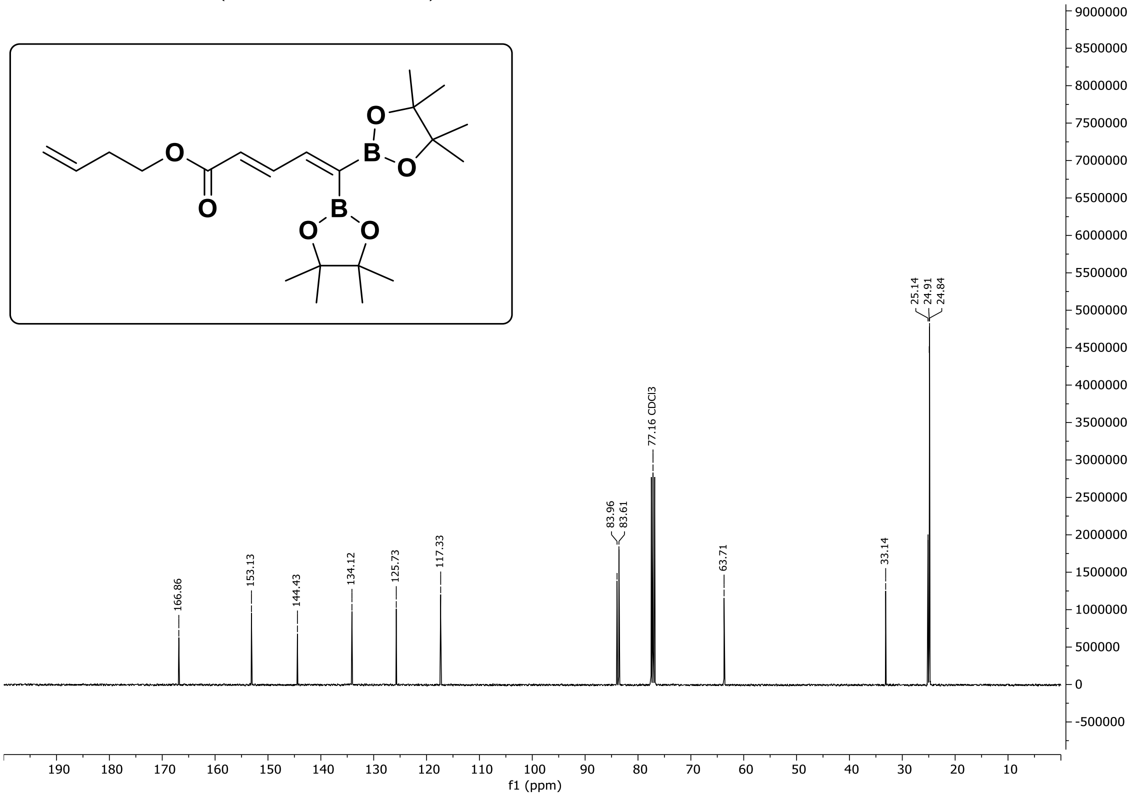

^{11}B NMR of S20 (128 MHz, $CDCl_3$)

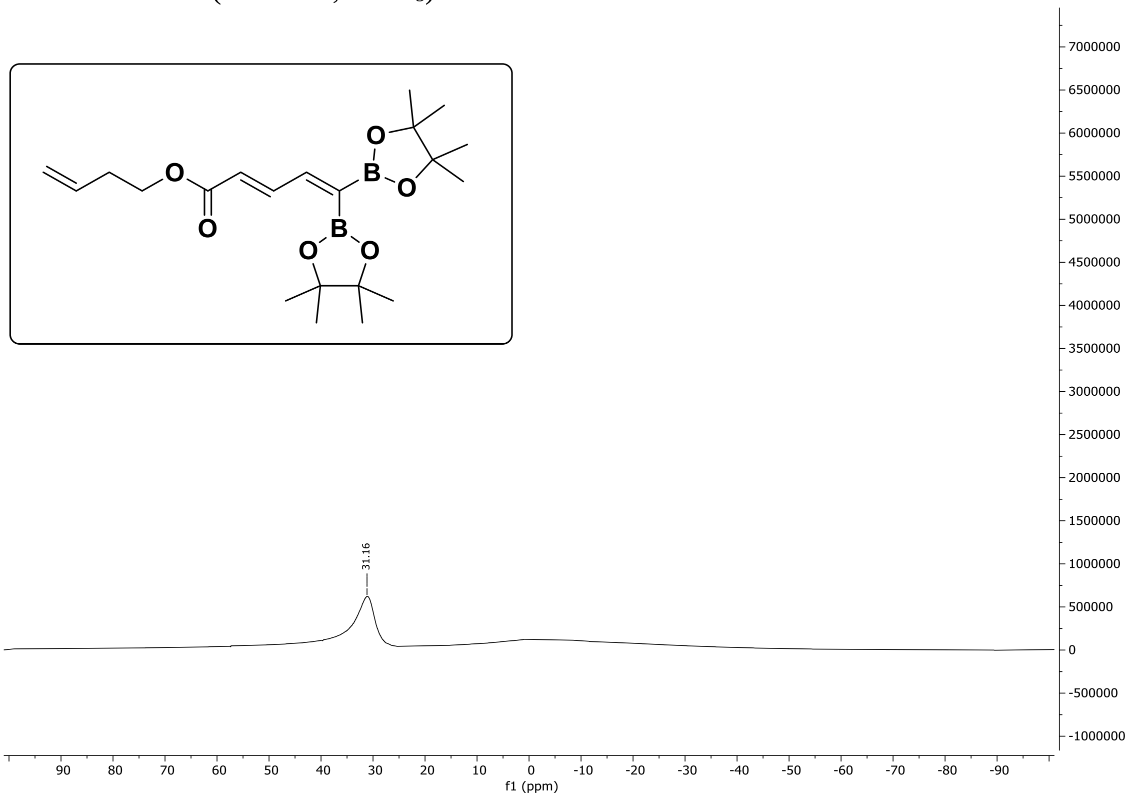

^{1}H-NMR of S21 (400 MHz, $CDCl_3$)

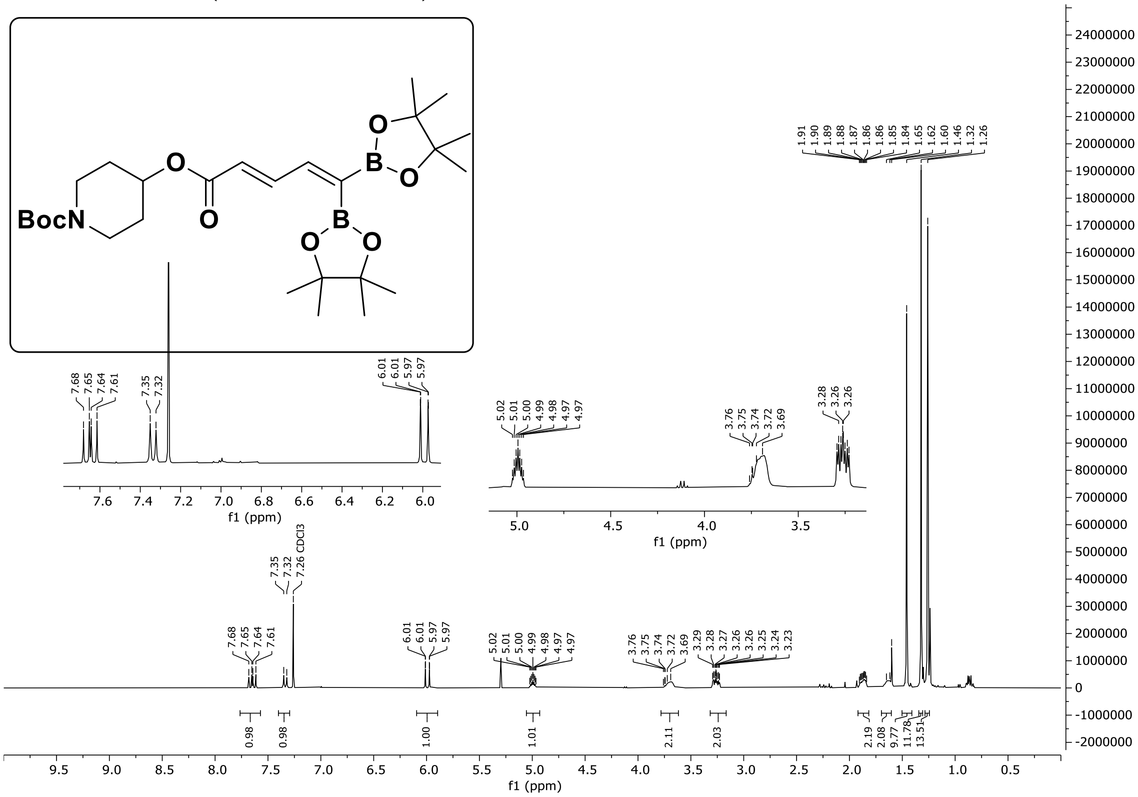

^{13}C-NMR of S21 (100 MHz, $CDCl_3$)

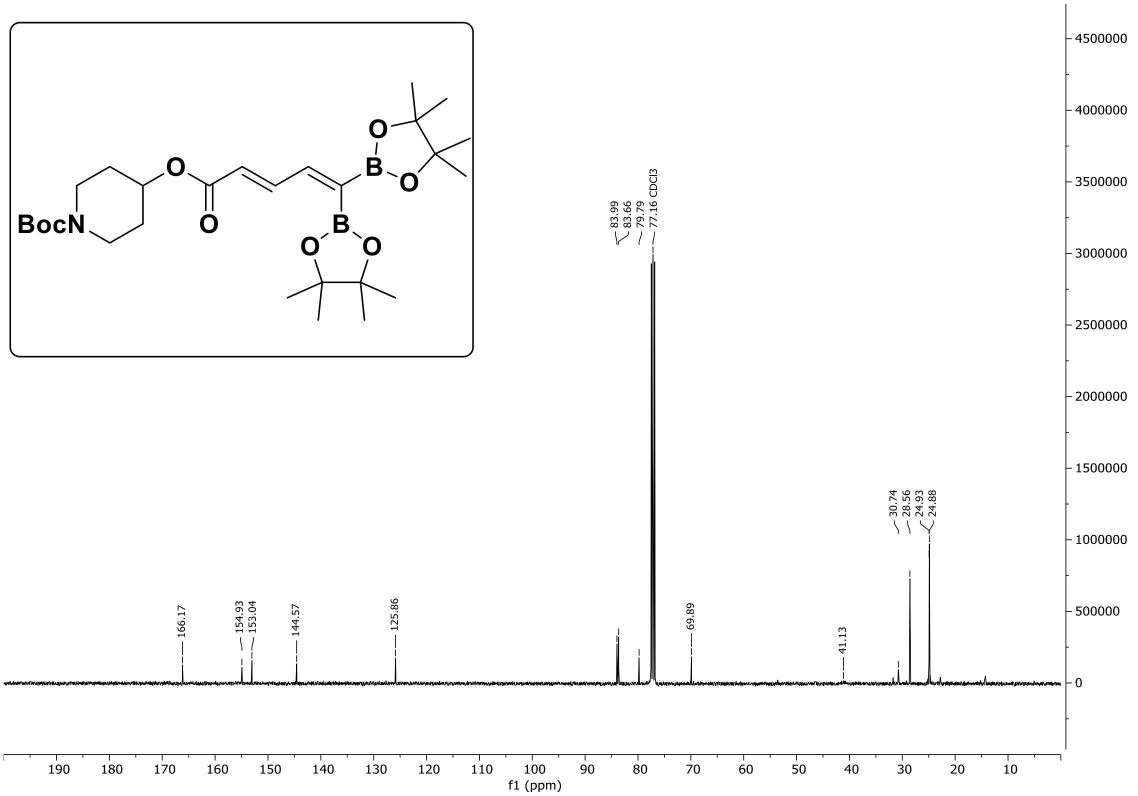

^{11}B NMR of S21 (128 MHz, $CDCl_3$)

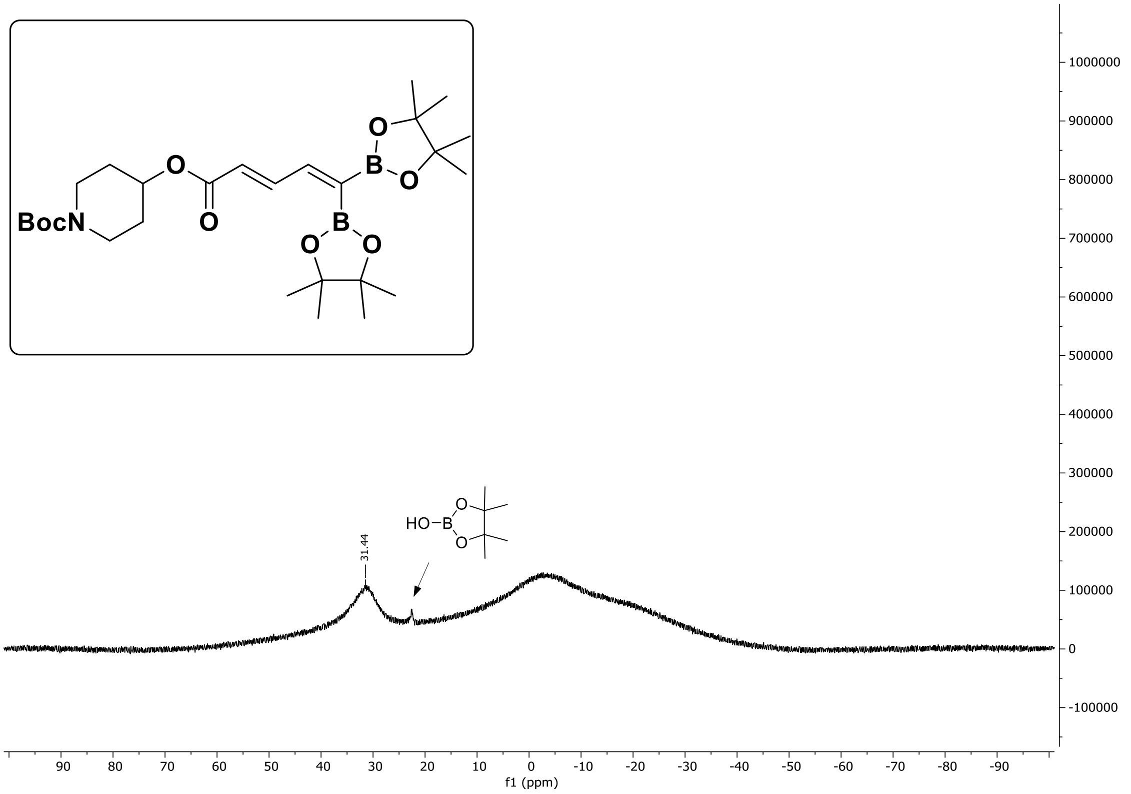

^{1}H-NMR of S22 (400 MHz, $CDCl_3$)

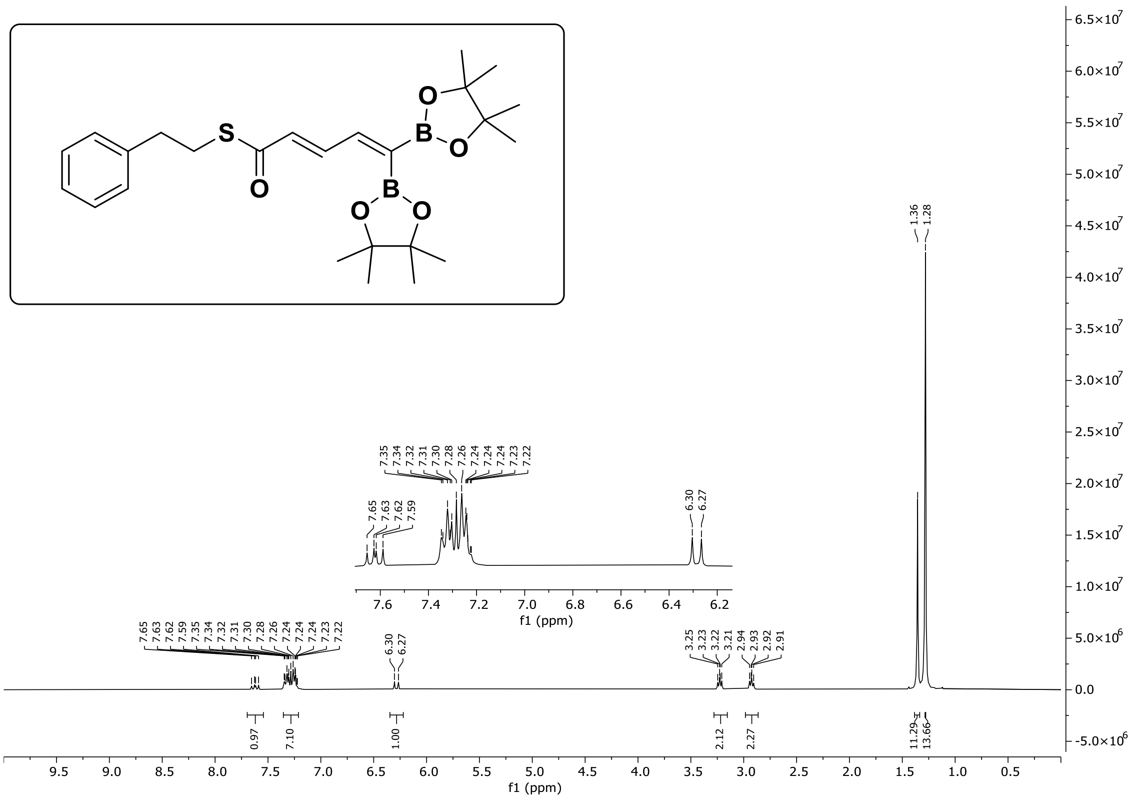

^{13}C-NMR of S22 (100 MHz, $CDCl_3$)

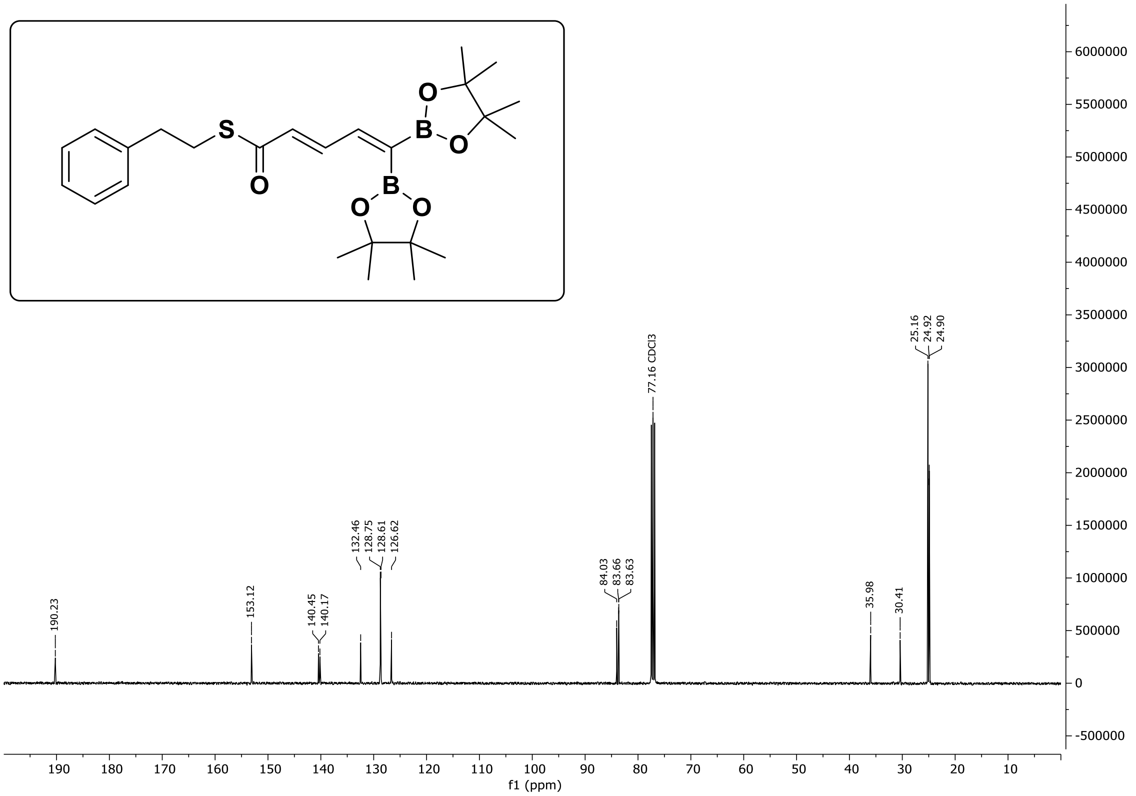

^{11}B NMR of S22 (128 MHz, $CDCl_3$)

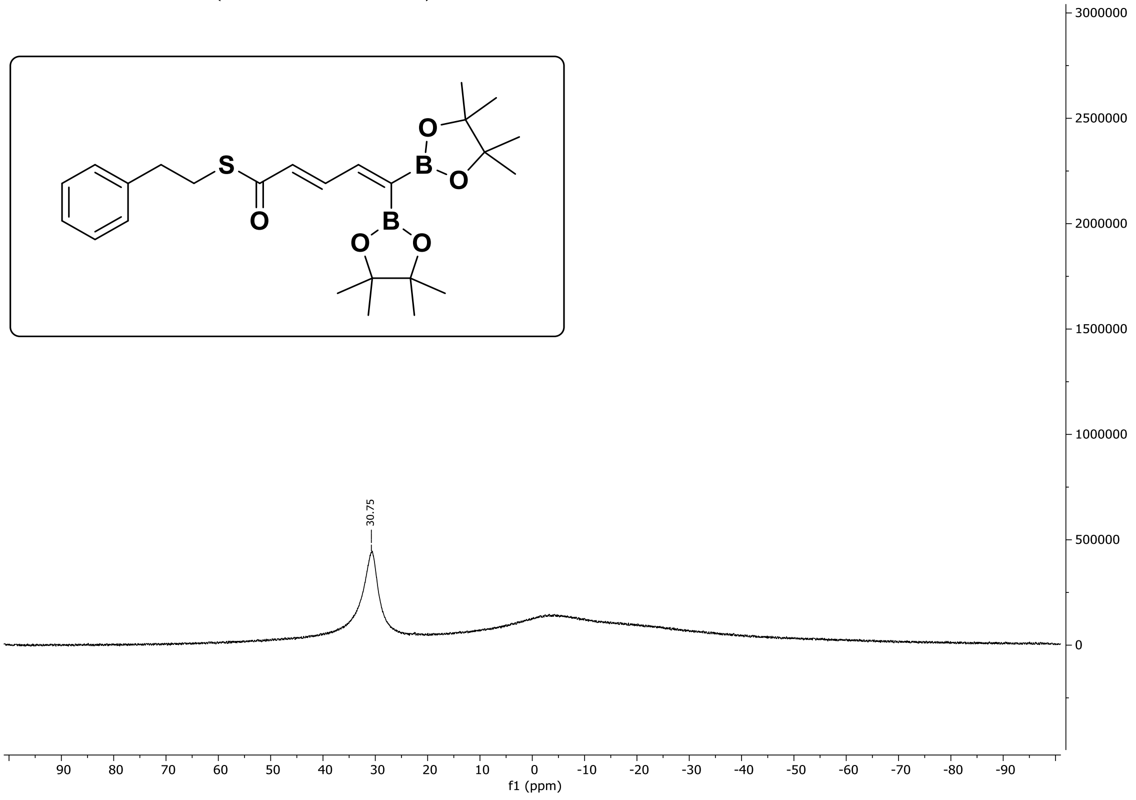

^{1}H-NMR of S23 (400 MHz, $CDCl_3$)

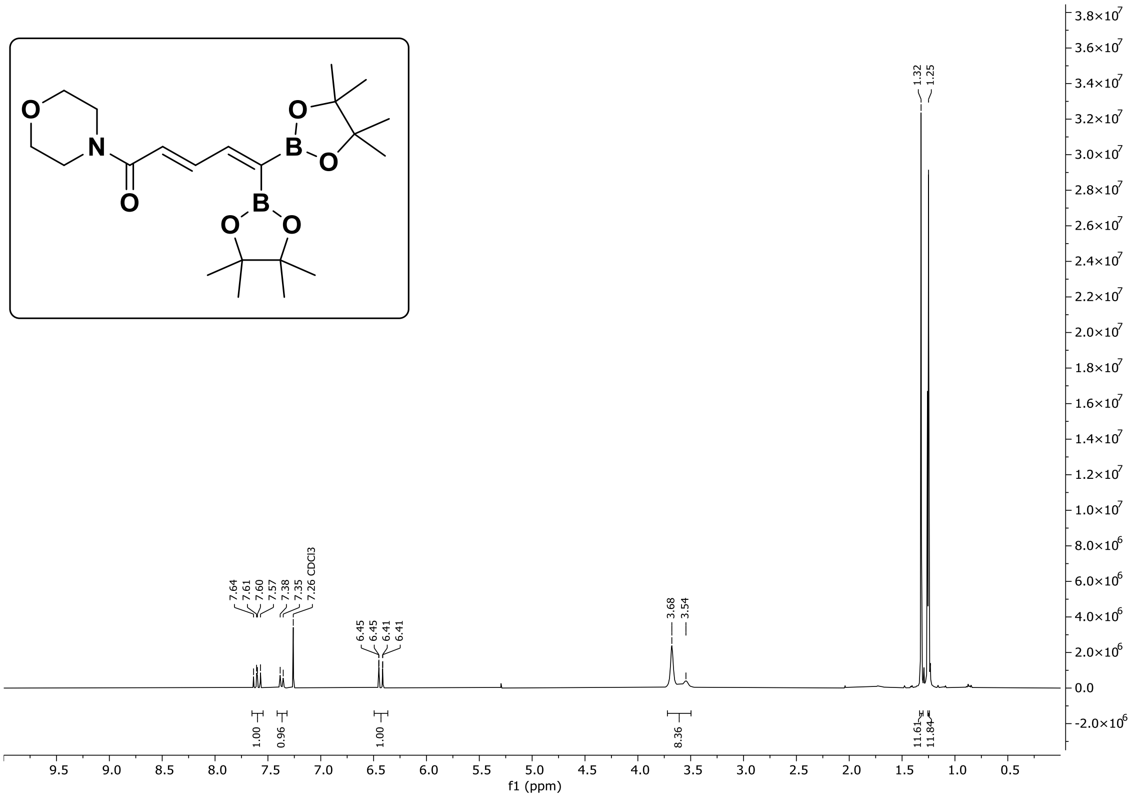

^{13}C-NMR of S23 (100 MHz, $CDCl_3$)

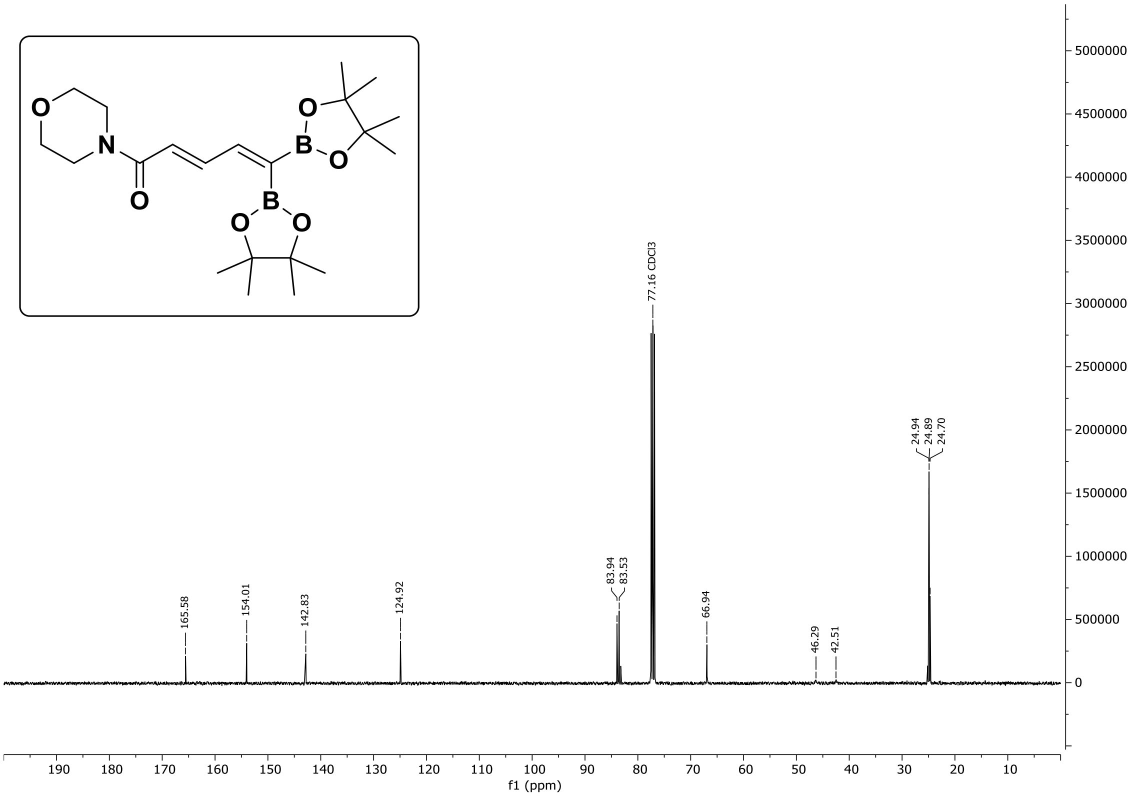

^{11}B NMR of S23 (128 MHz, $CDCl_3$)

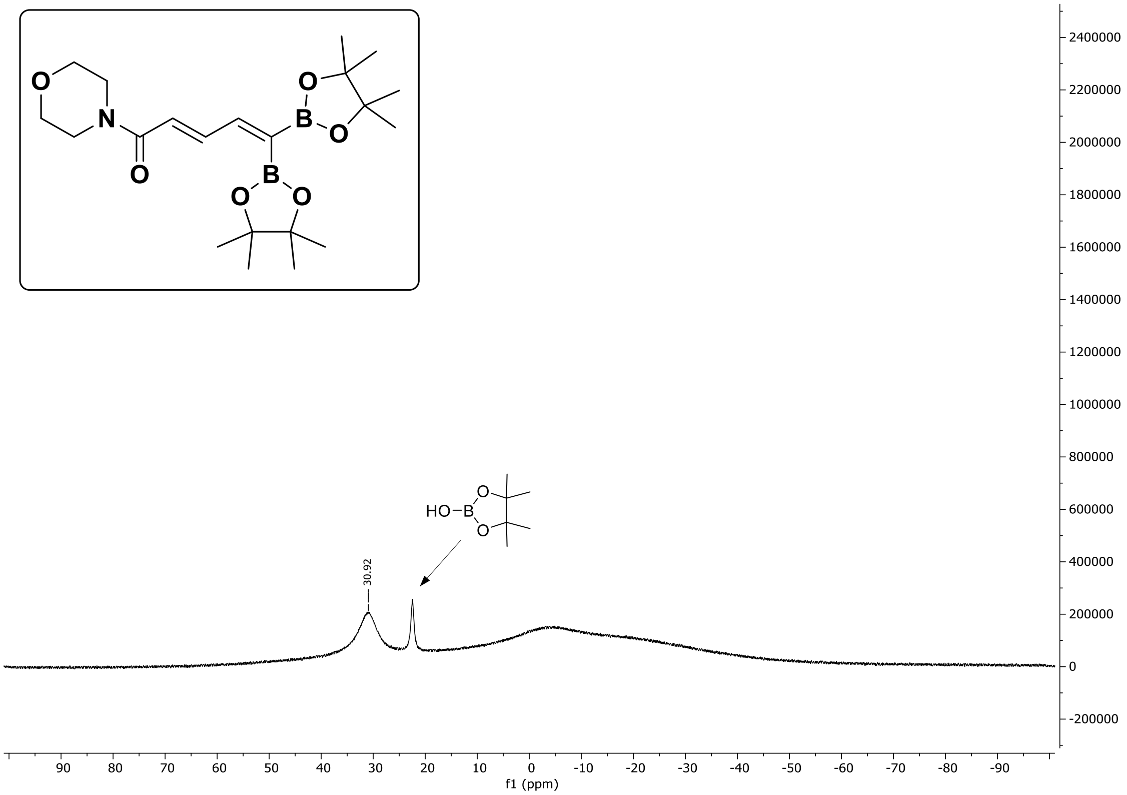

^{1}H-NMR of S24 (400 MHz, $CDCl_3$)

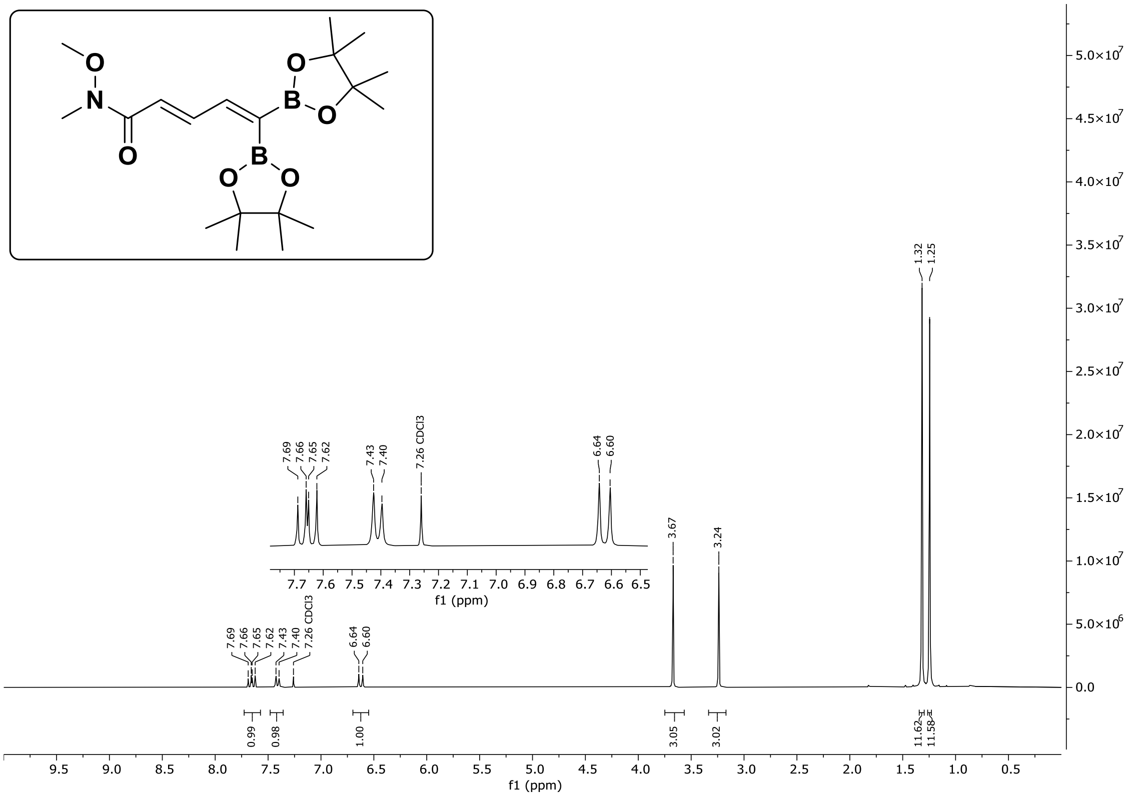

^{13}C-NMR of S24 (100 MHz, $CDCl_3$)

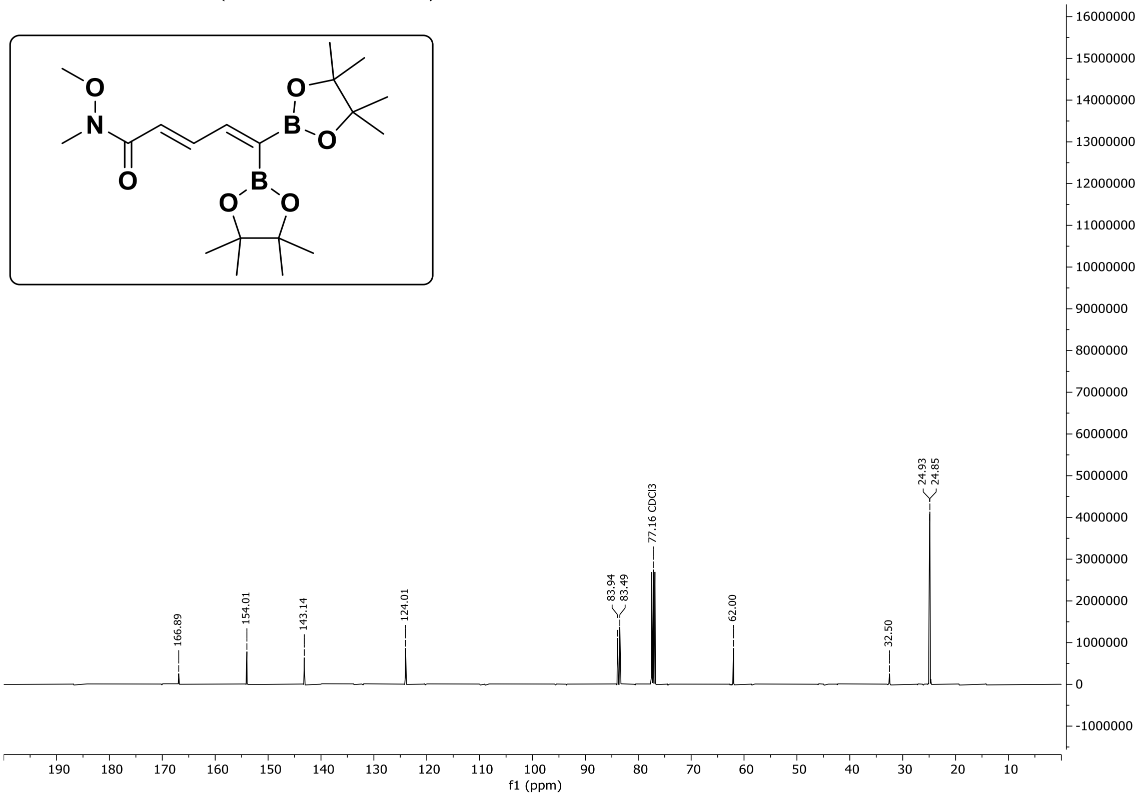

^{11}B NMR of S24 (128 MHz, $CDCl_3$)

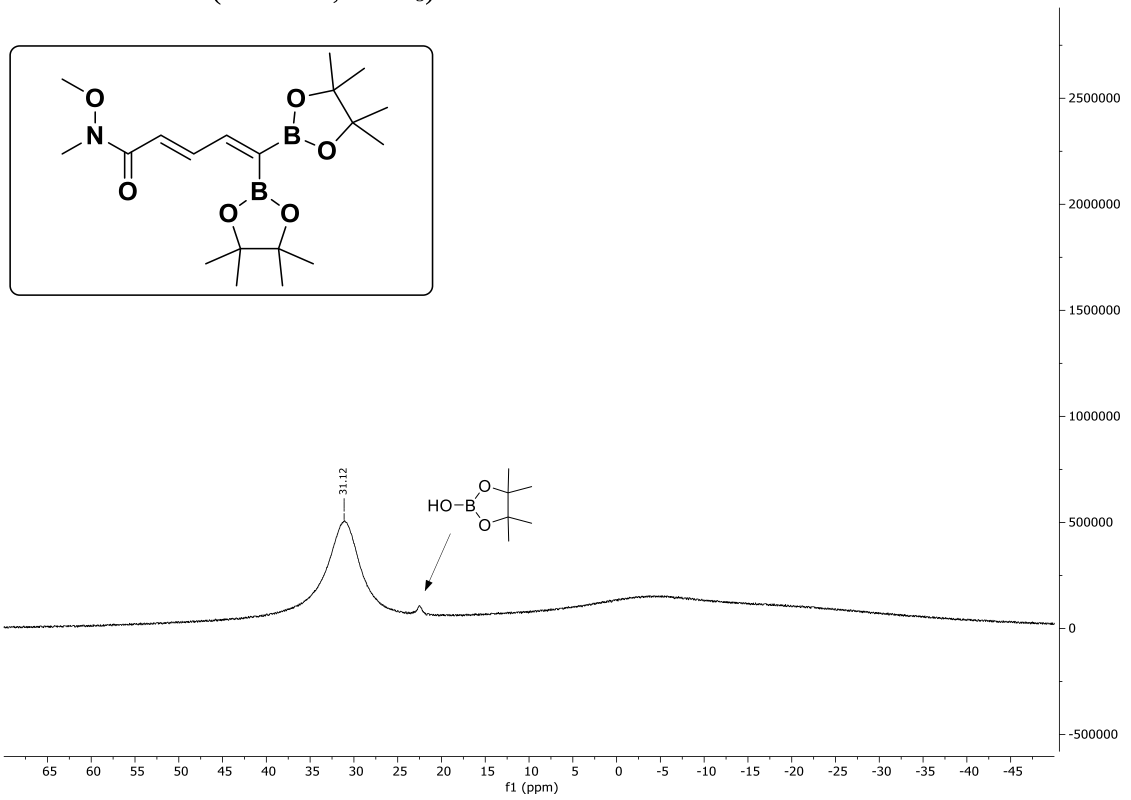

^{1}H-NMR of S25 (400 MHz, $CDCl_3$)

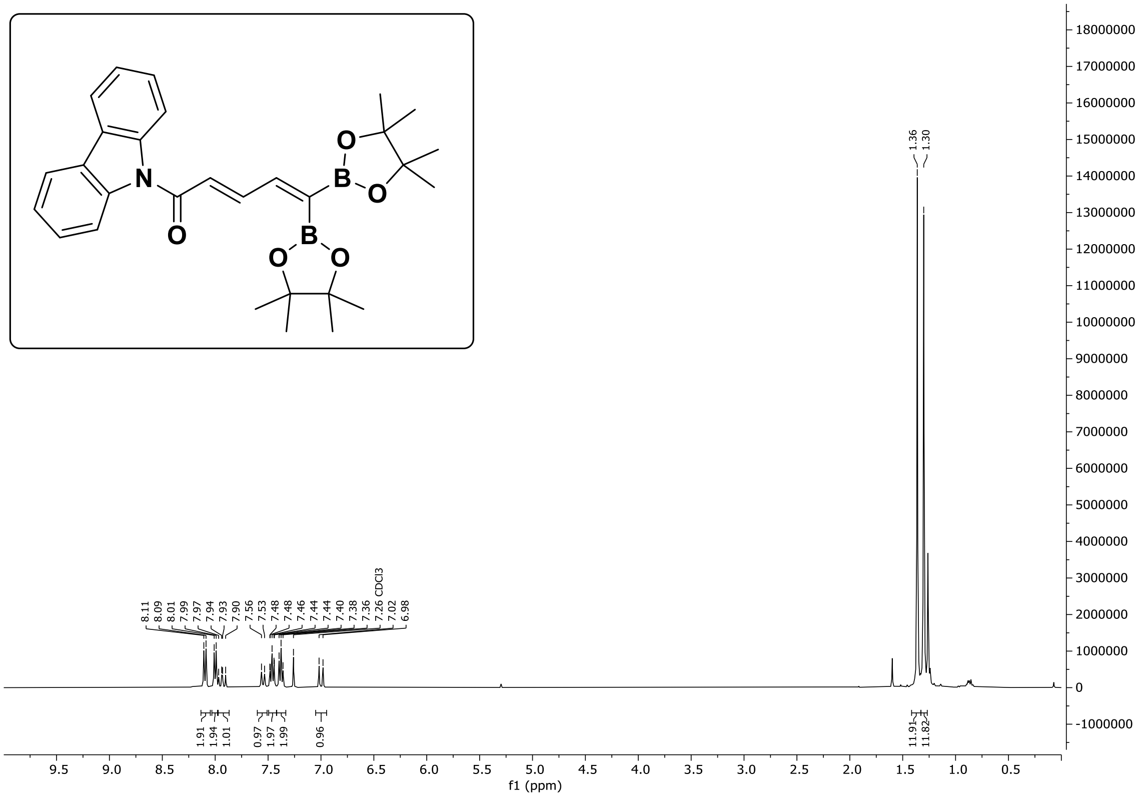

^{13}C-NMR of S25 (100 MHz, $CDCl_3$)

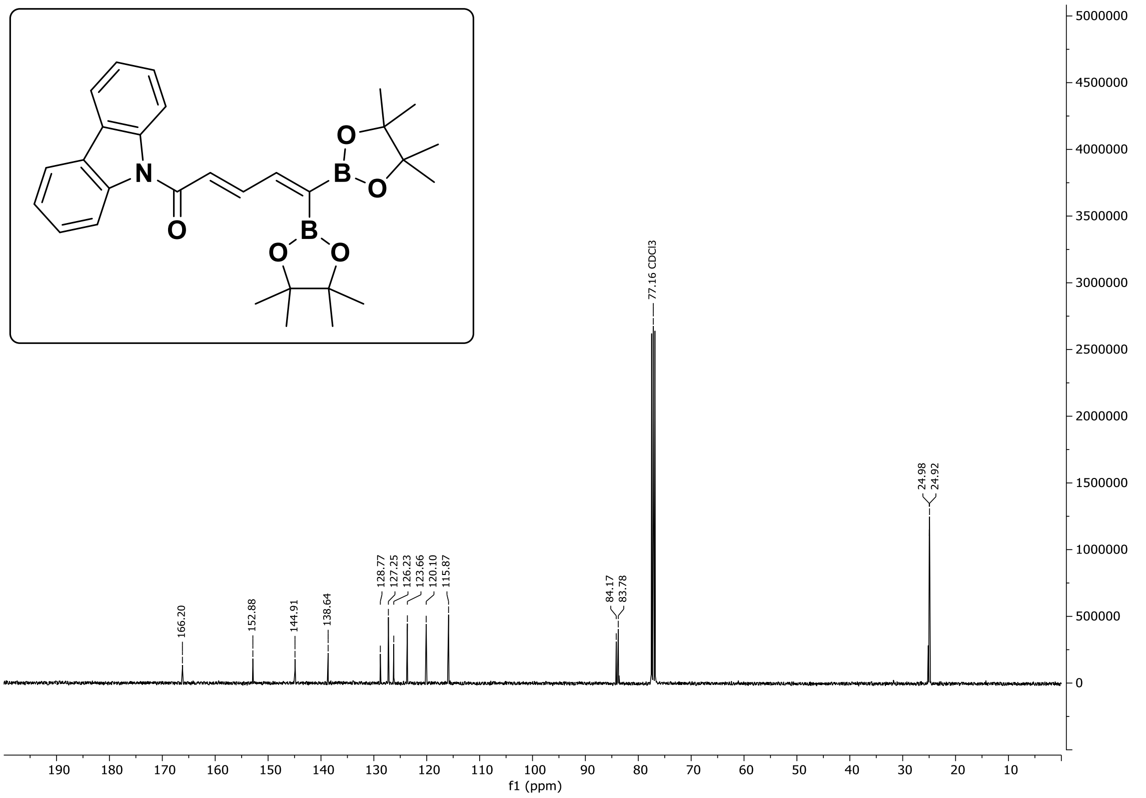

^{11}B NMR of S25 (128 MHz, $CDCl_3$)

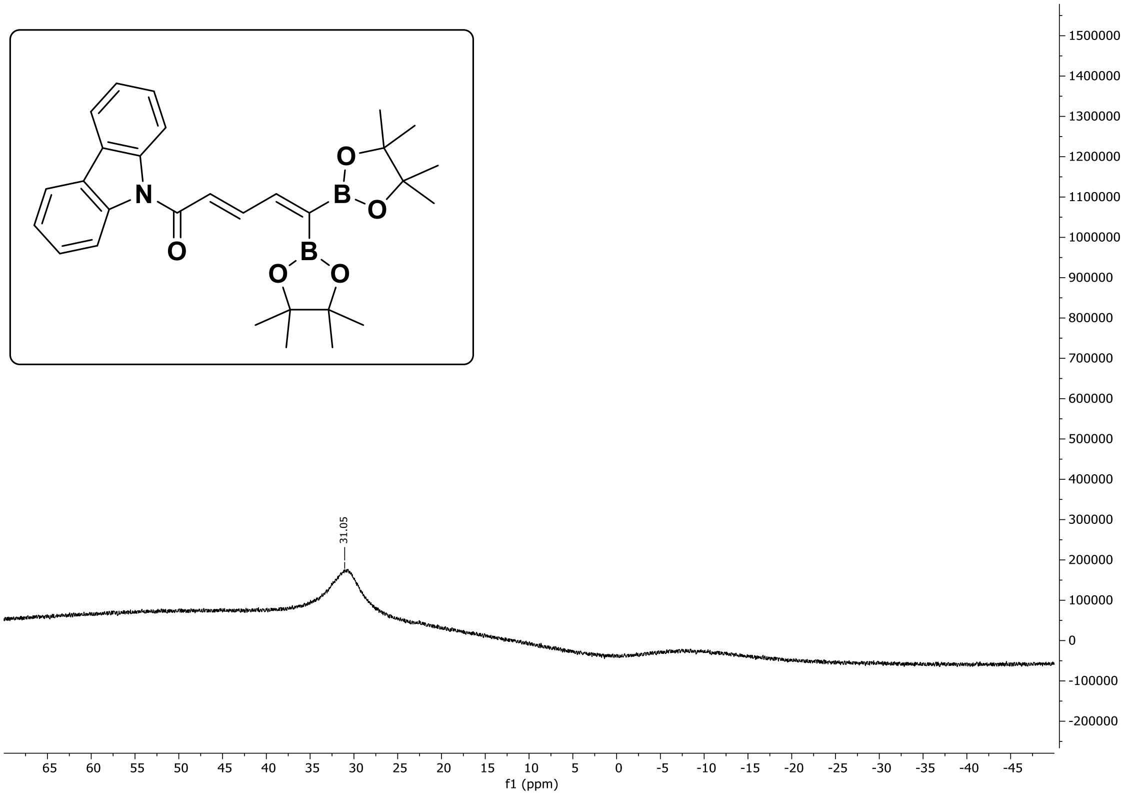

^{1}H-NMR of S26 (400 MHz, $CDCl_3$)

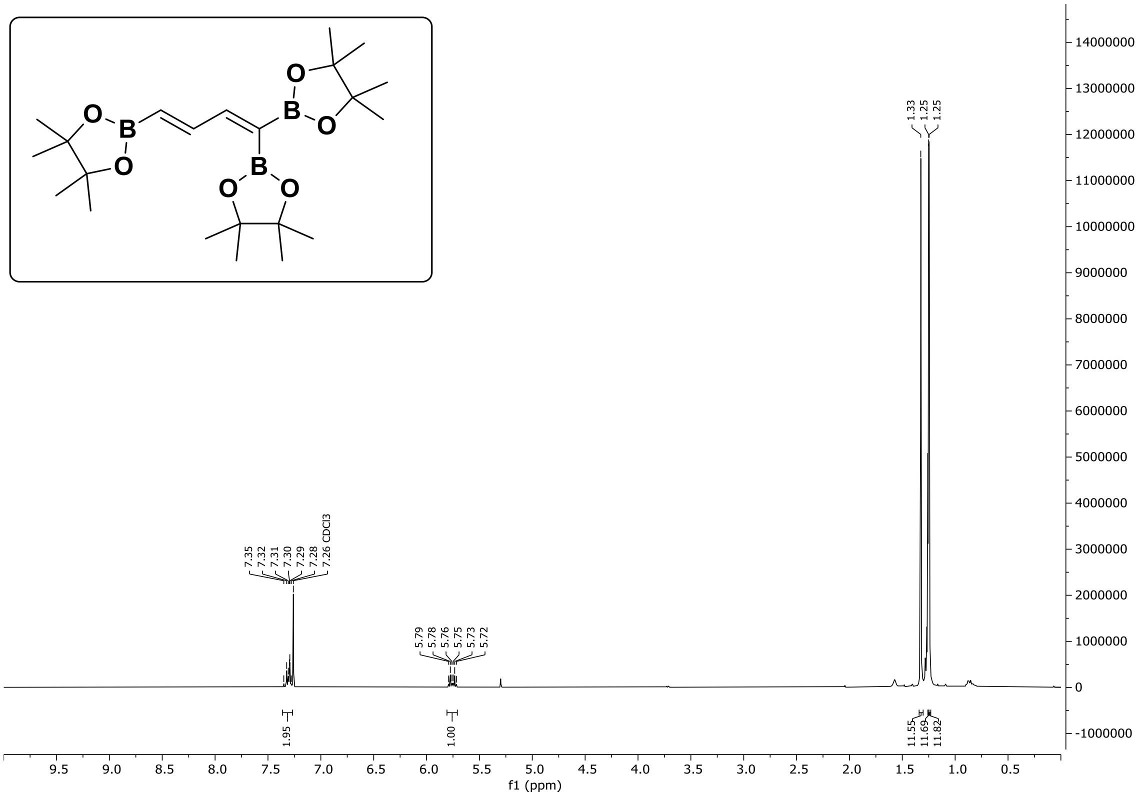

^{13}C-NMR of S26 (100 MHz, $CDCl_3$)

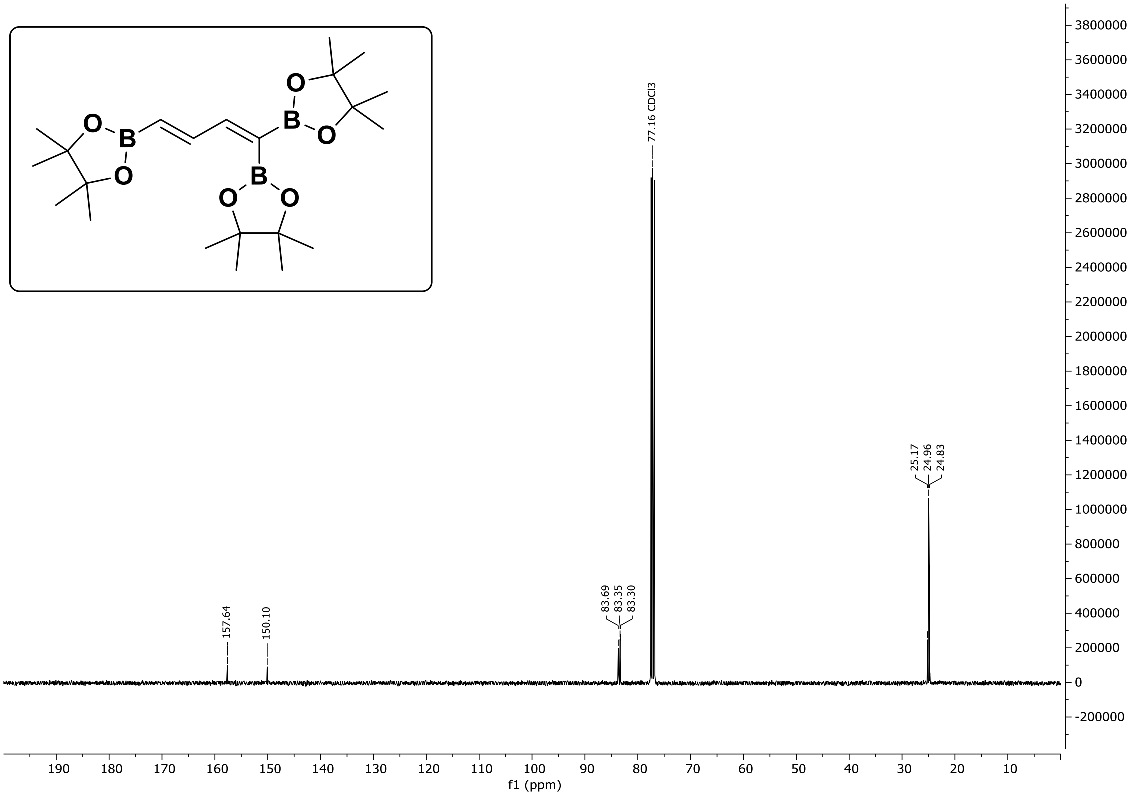

^{11}B NMR of S26 (128 MHz, $CDCl_3$)

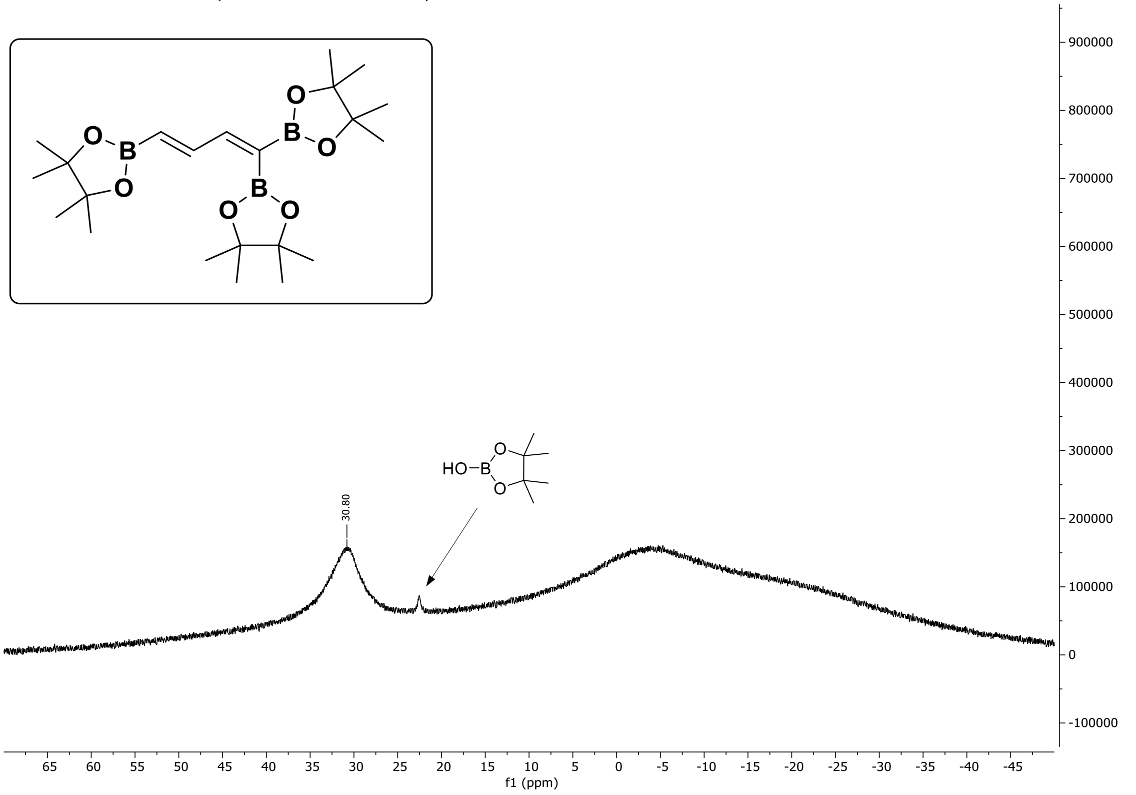

^{1}H-NMR of S27 (400 MHz, $CDCl_3$)

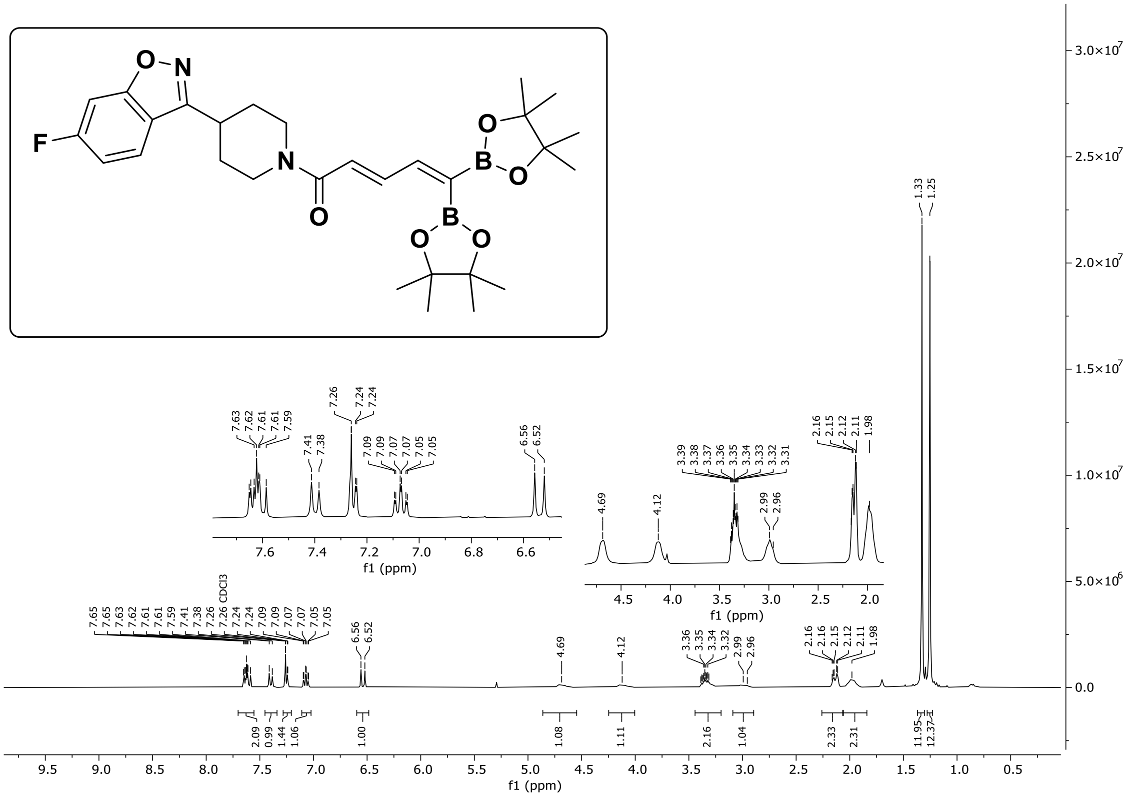

^{13}C-NMR of S27 (100 MHz, $CDCl_3$)

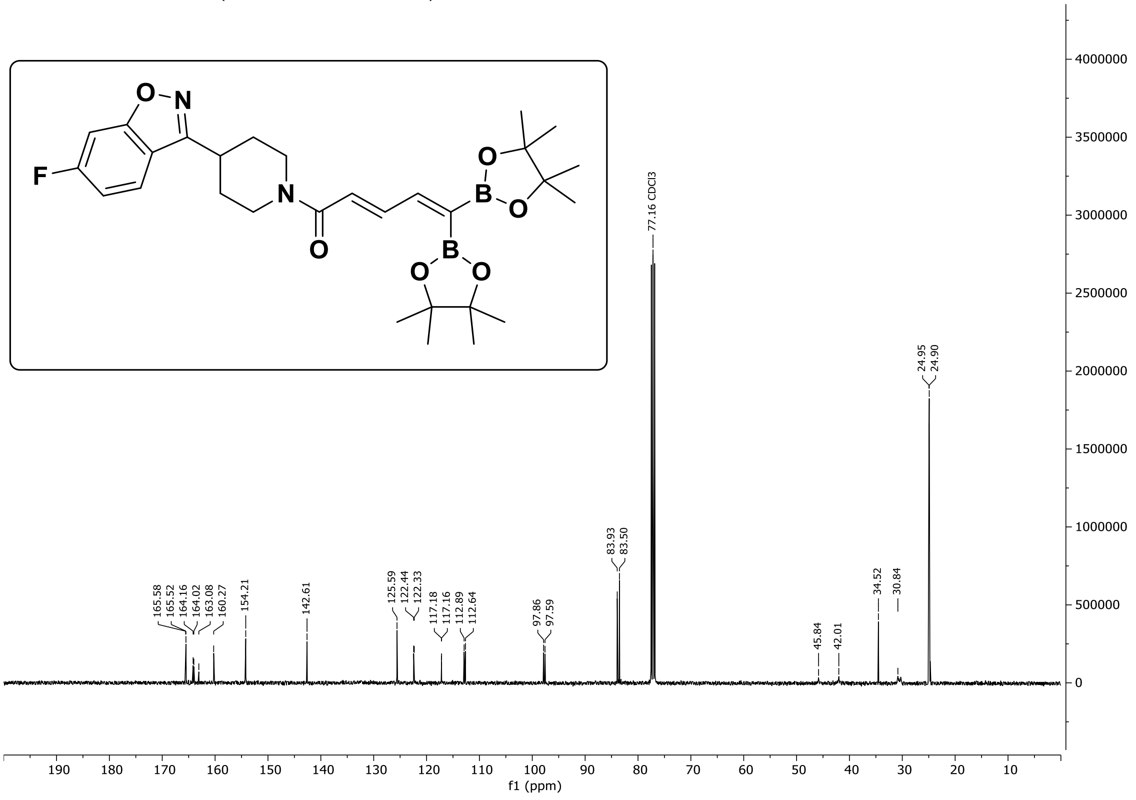

^{19}F-NMR of S27 (376 MHz, $CDCl_3$)

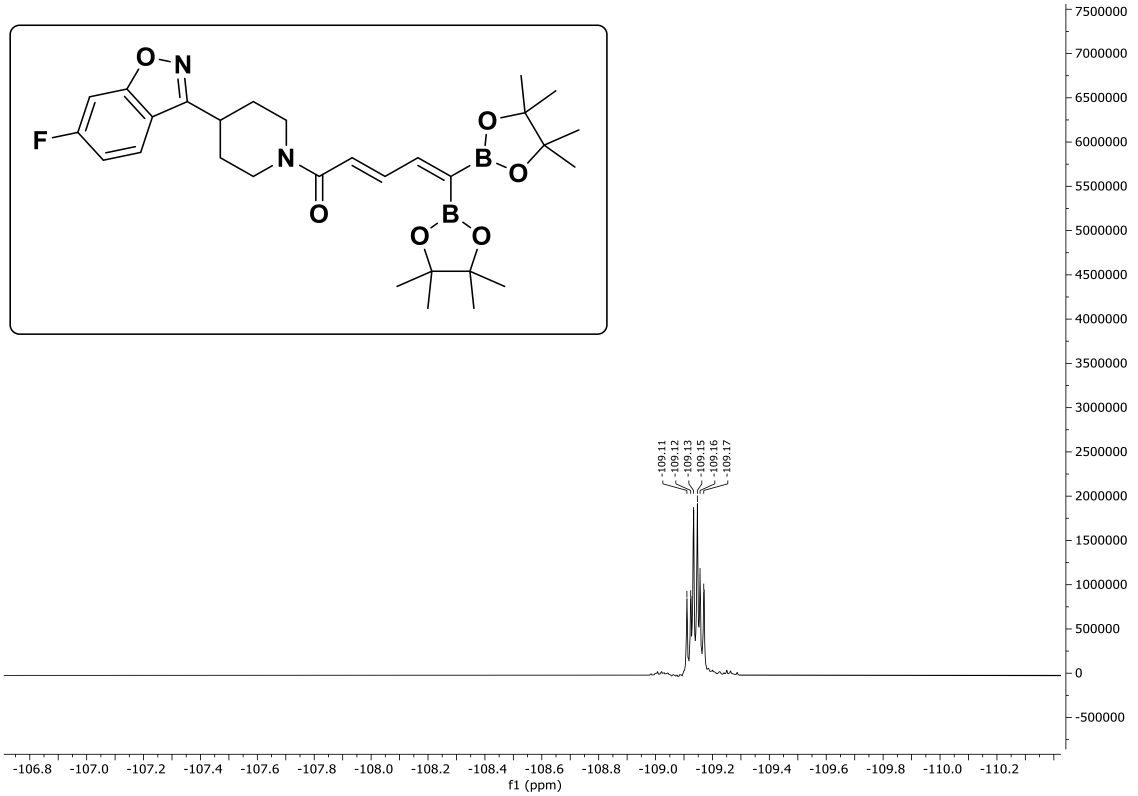

^{11}B NMR of S27 (128 MHz, $CDCl_3$)

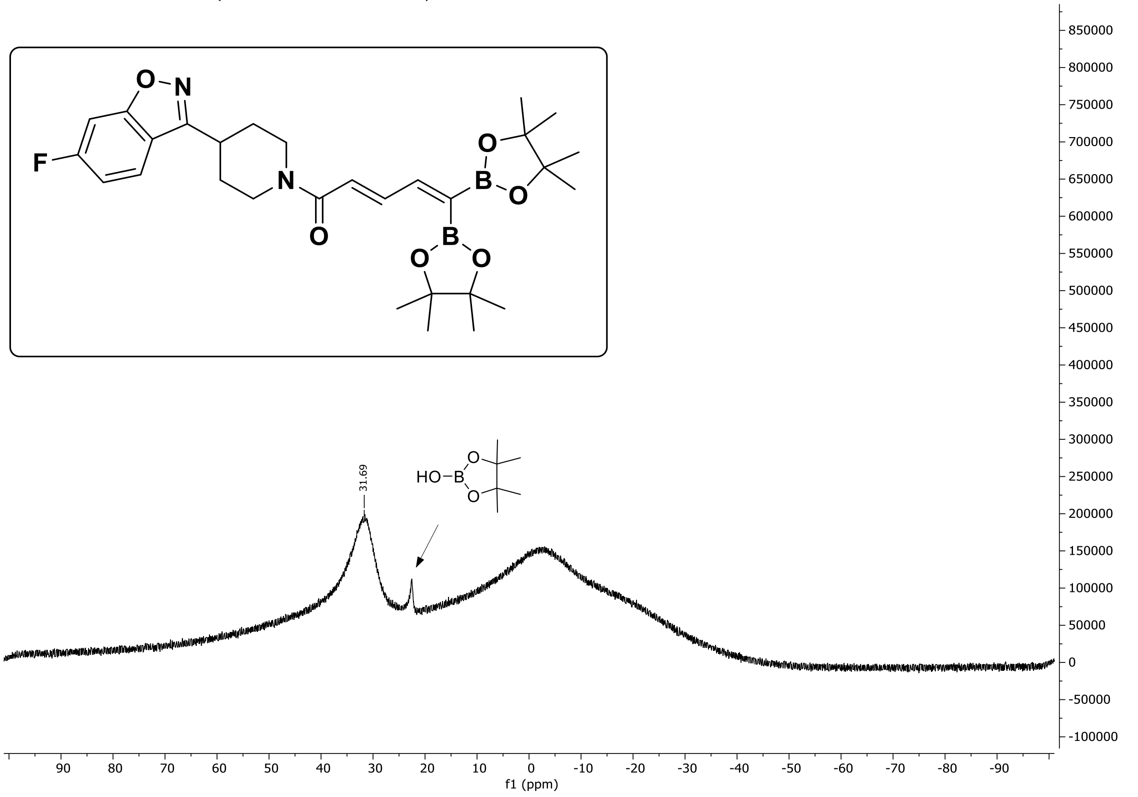

^{1}H-NMR of S28 (400 MHz, $CDCl_3$)

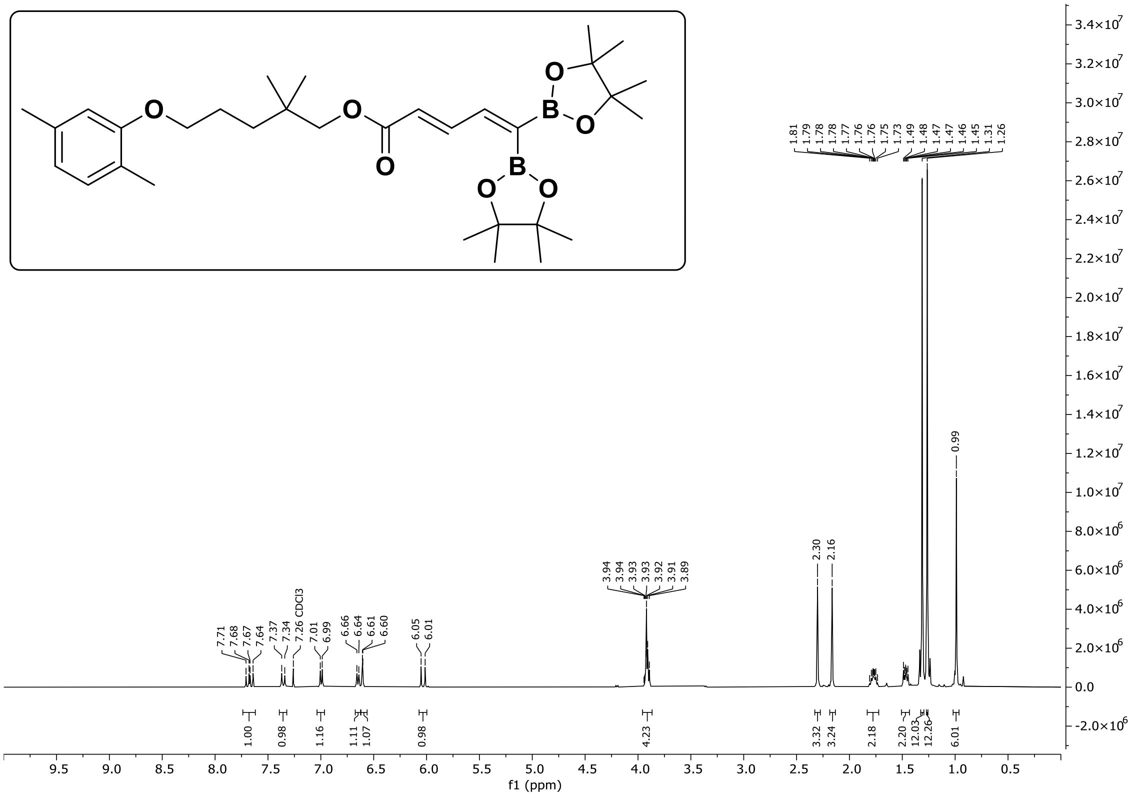

^{13}C-NMR of S28 (100 MHz, $CDCl_3$)

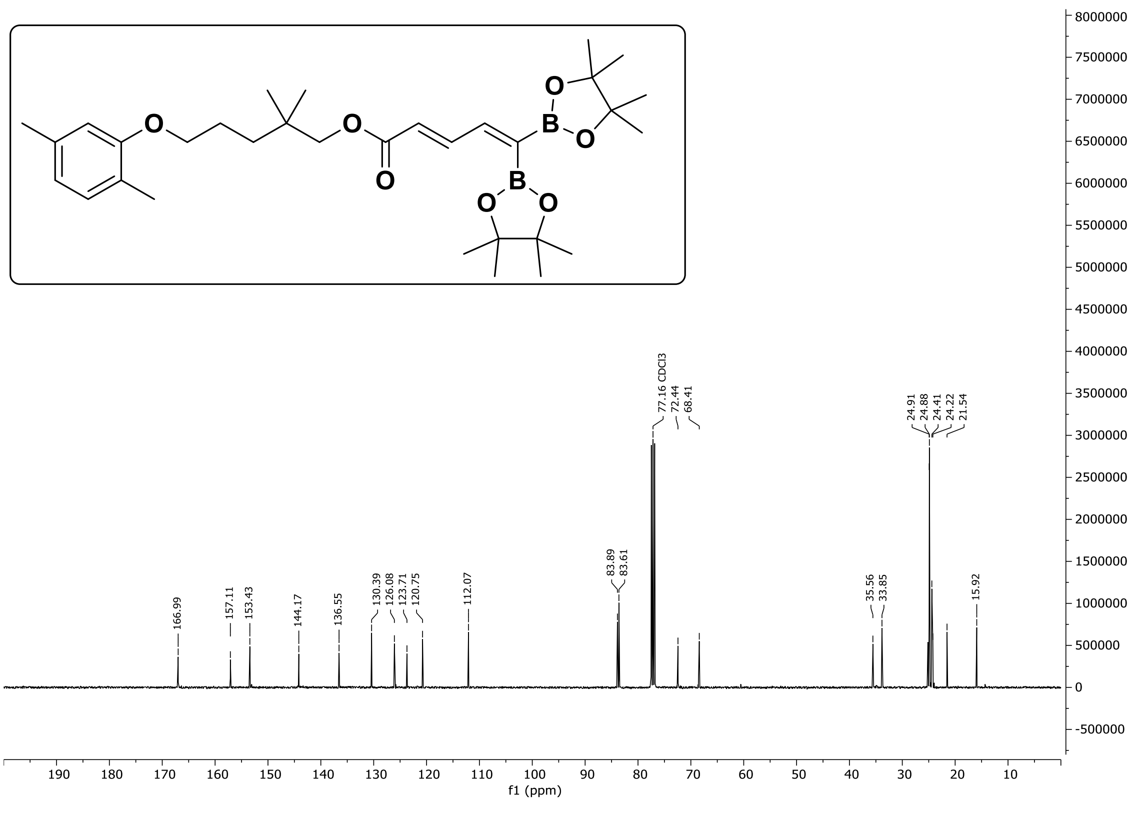

^{11}B NMR of S28 (128 MHz, $CDCl_3$)

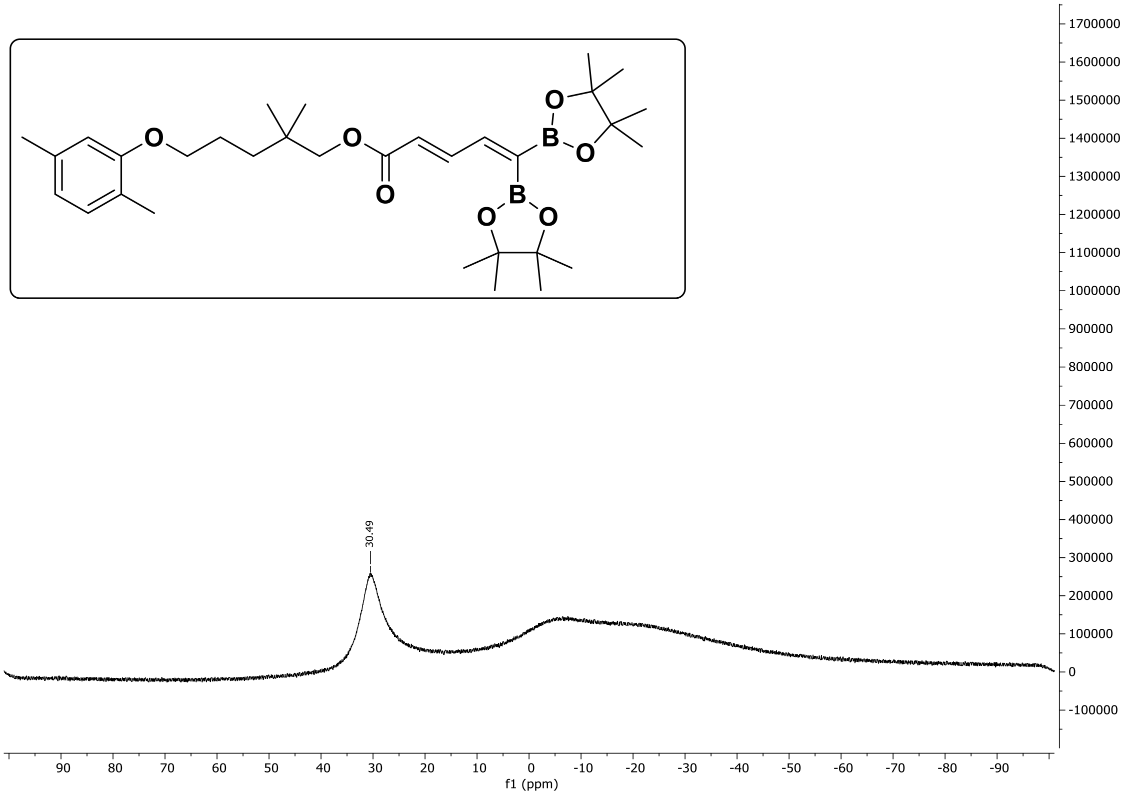

^{1}H-NMR of S29 (400 MHz, $CDCl_3$)

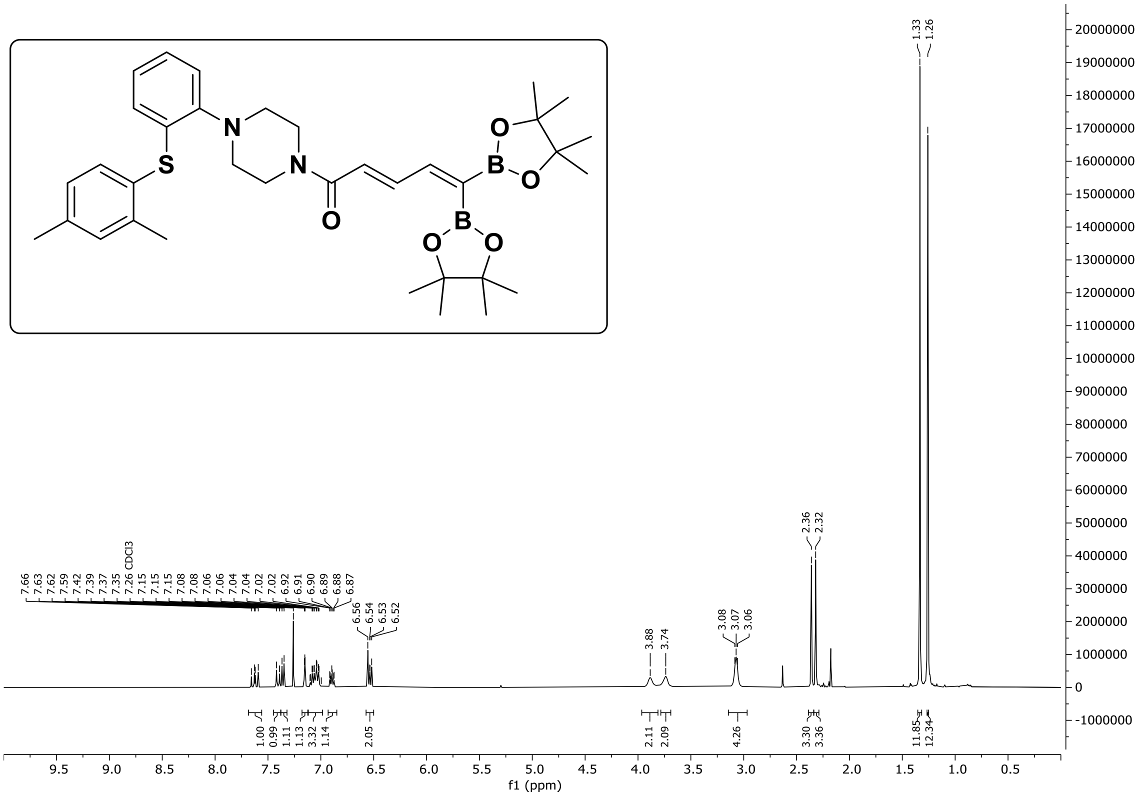

^{13}**C-NMR of S29** (100 MHz, $CDCl_3$)

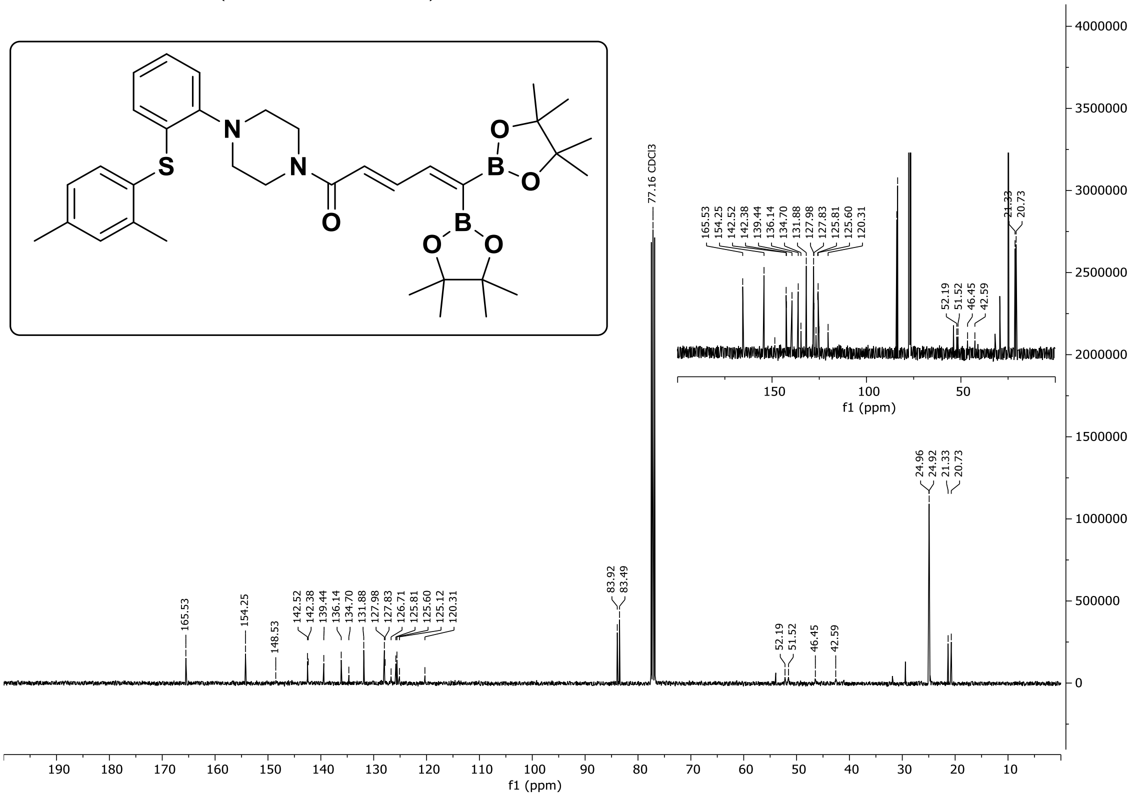

^{11}**B NMR of S29** (128 MHz, $CDCl_3$)

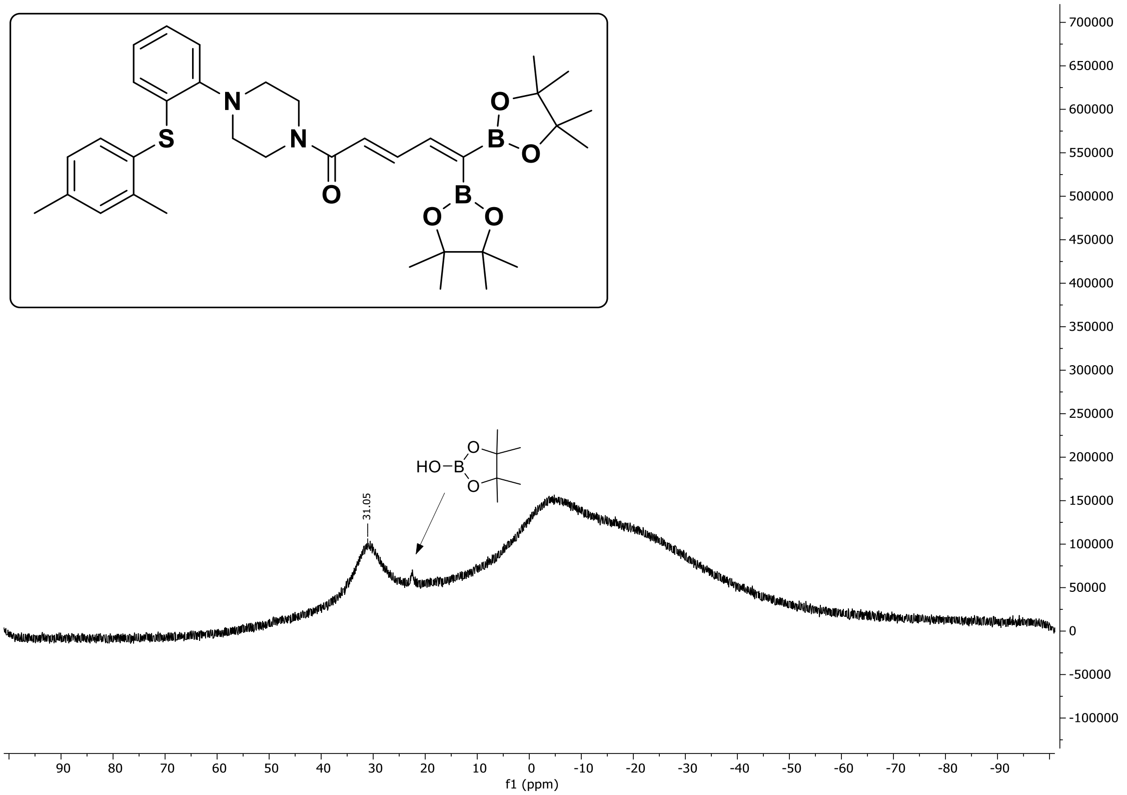

^{1}H-NMR of 5 (400 MHz, $CDCl_3$)

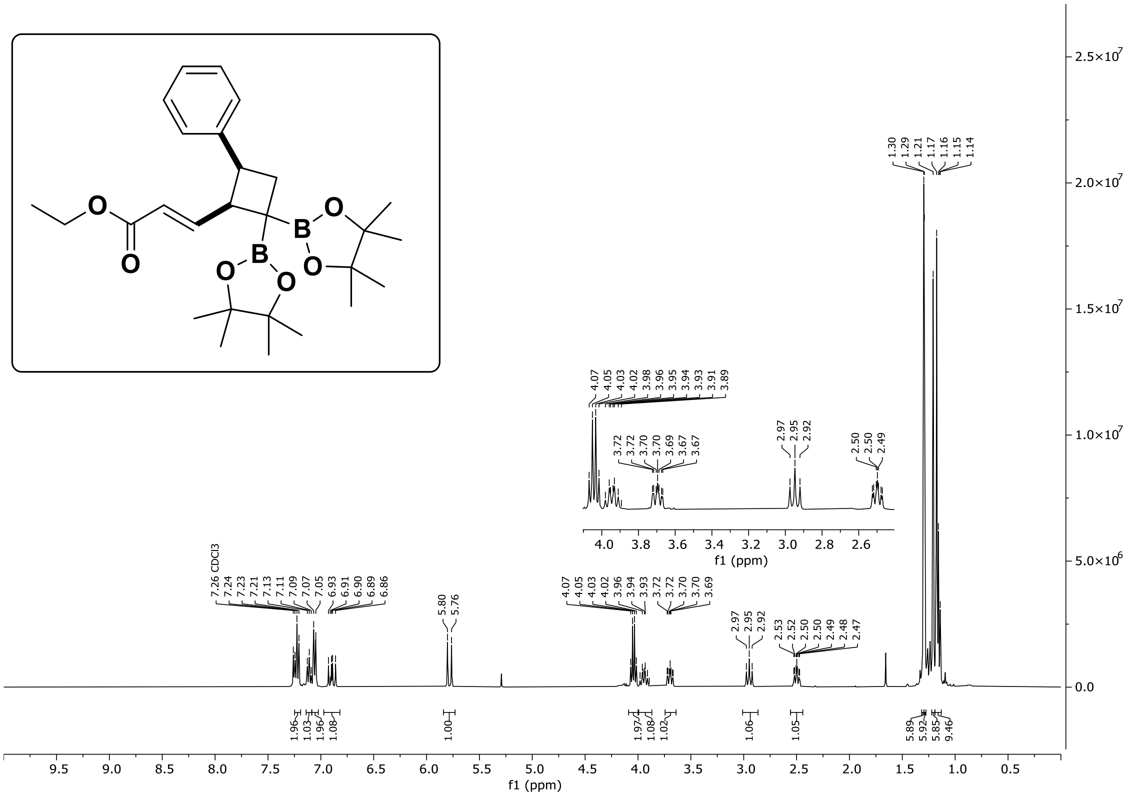

^{13}C-NMR of 5 (100 MHz, $CDCl_3$)

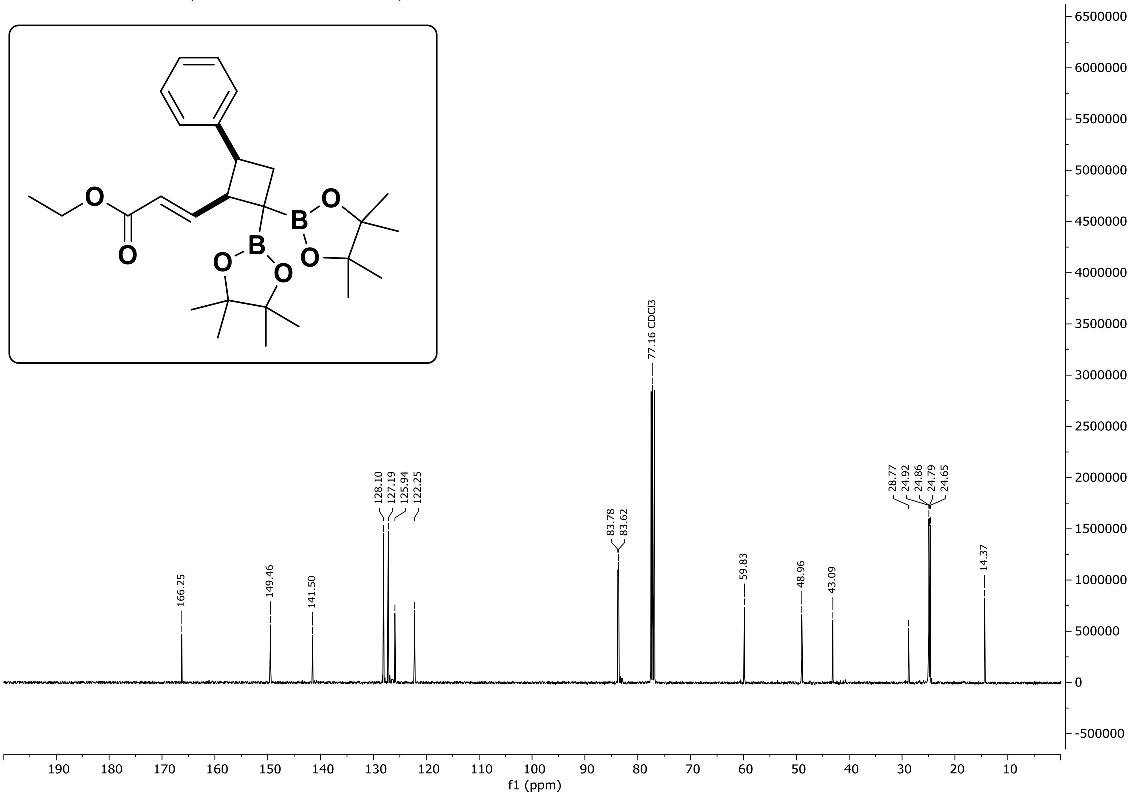

^{11}B NMR of 5 (128 MHz, $CDCl_3$)

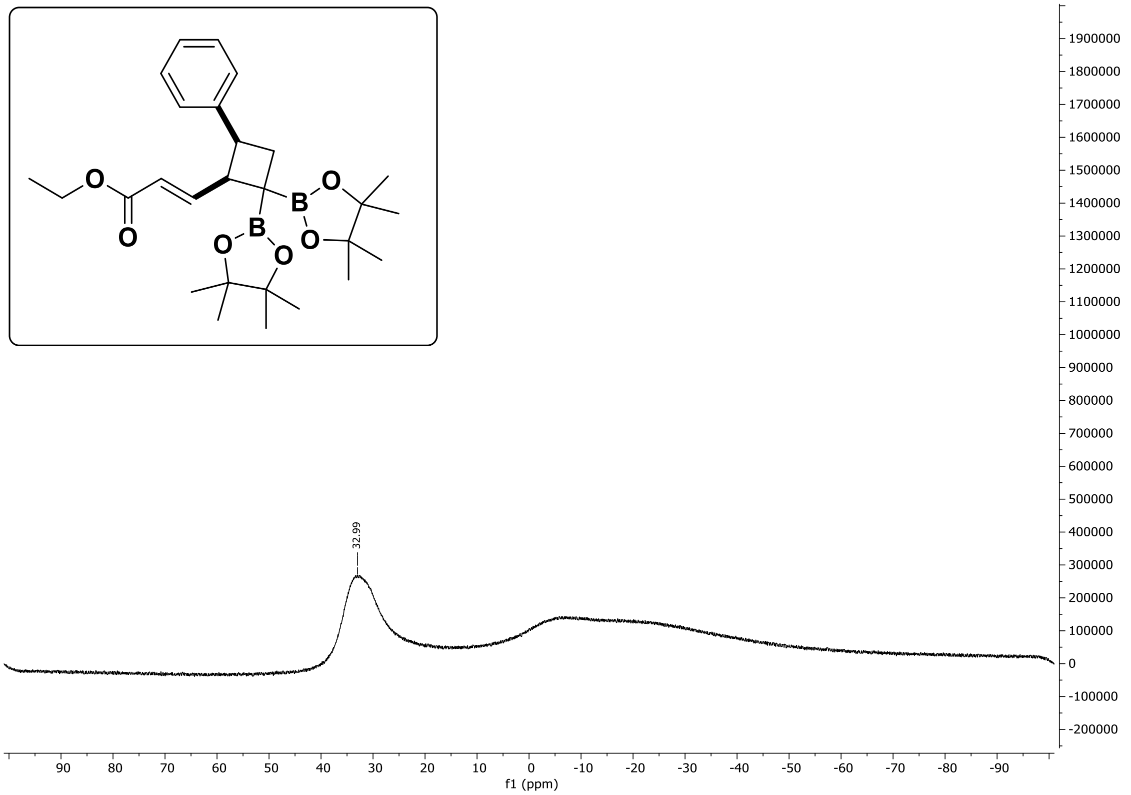

^{1}H-NMR of 6 (400 MHz, $CDCl_3$)

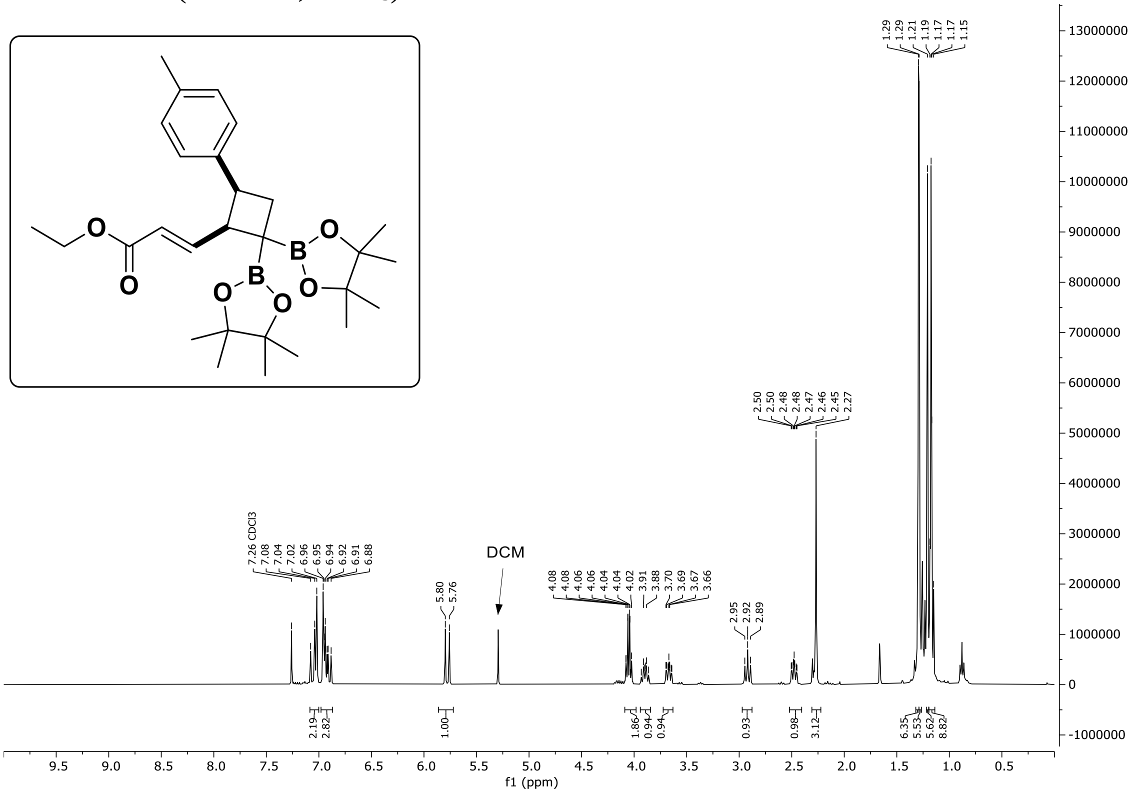

^{13}C-NMR of 6 (100 MHz, $CDCl_3$)

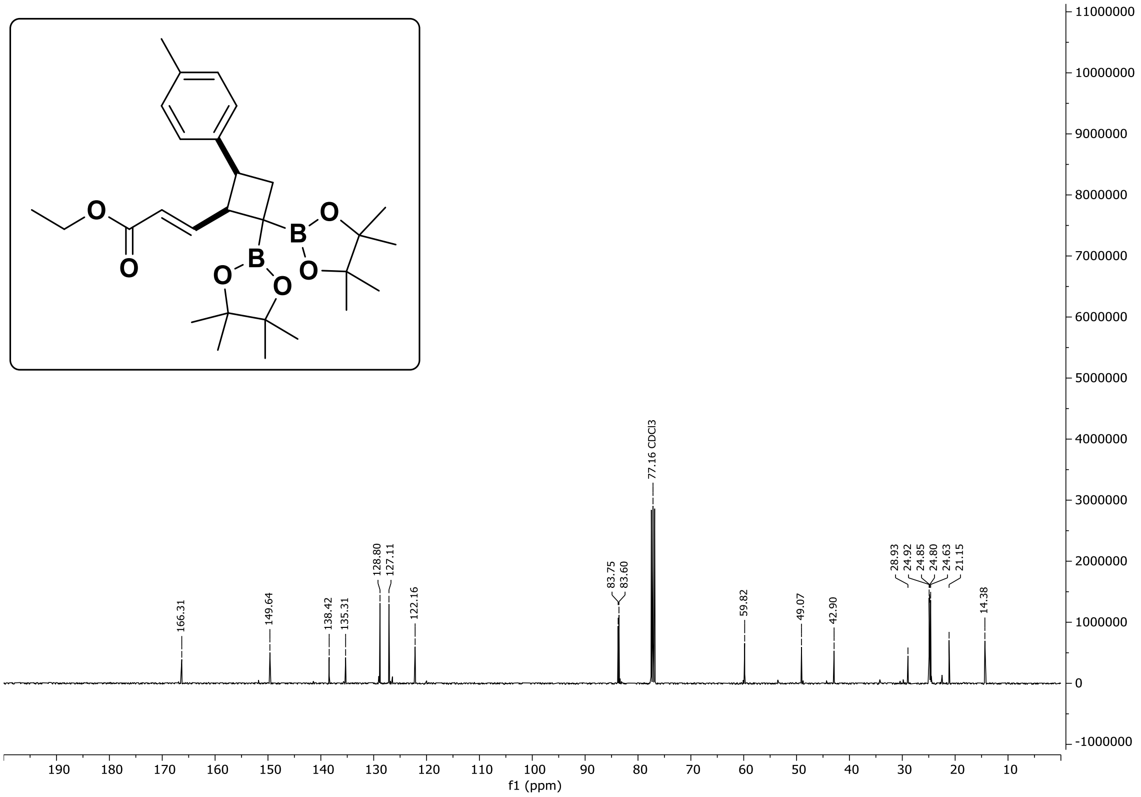

^{11}B NMR of 6 (128 MHz, $CDCl_3$)

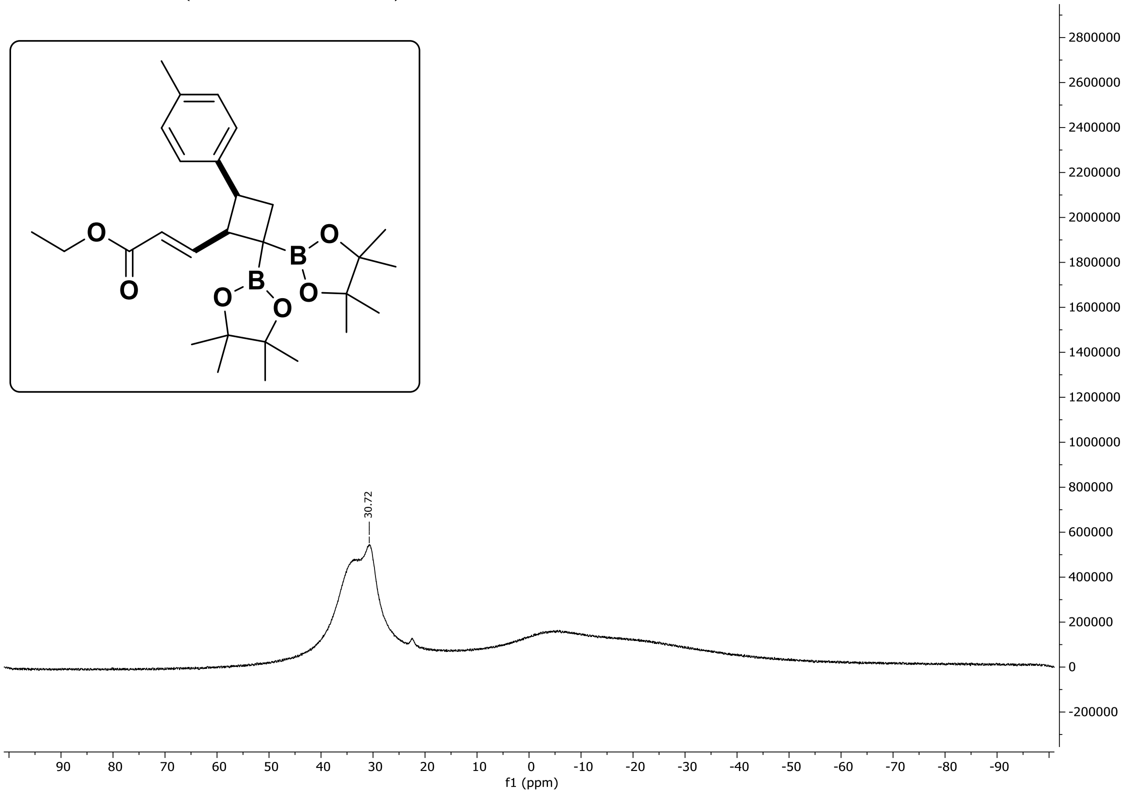

^{1}H-NMR of 7-major (400 MHz, $CDCl_3$)

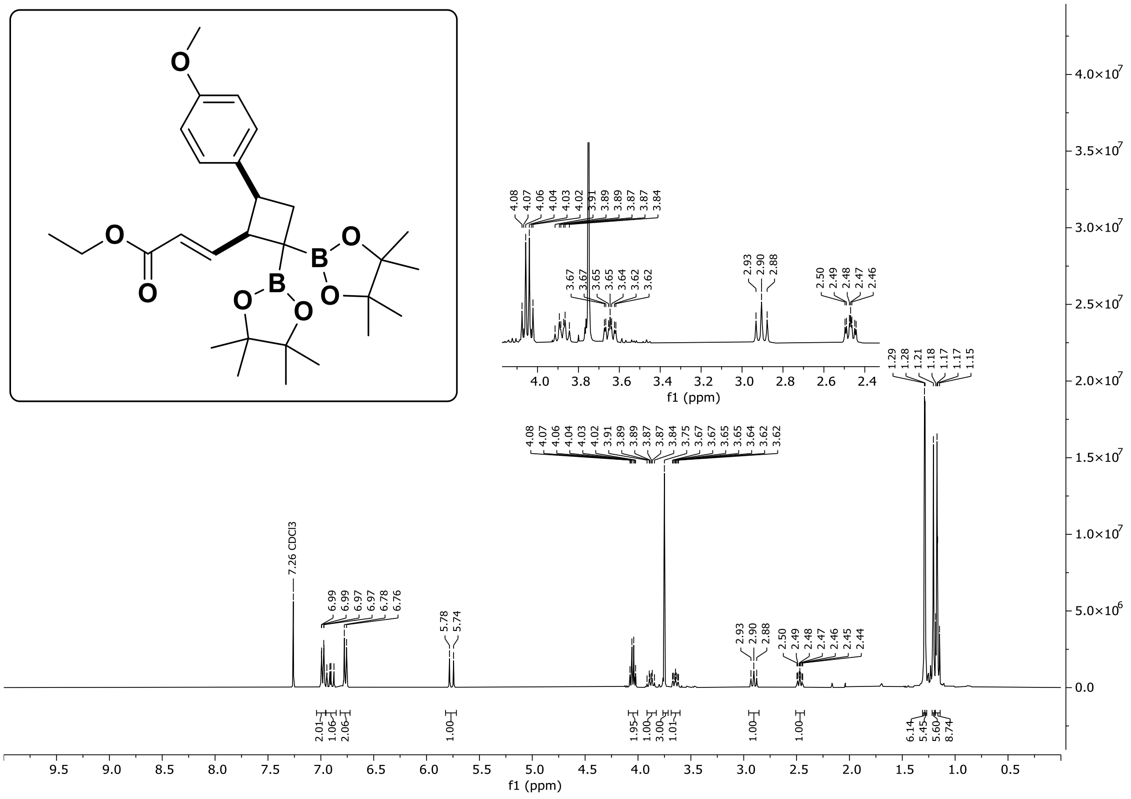

^{13}C-NMR of 7-major (100 MHz, $CDCl_3$)

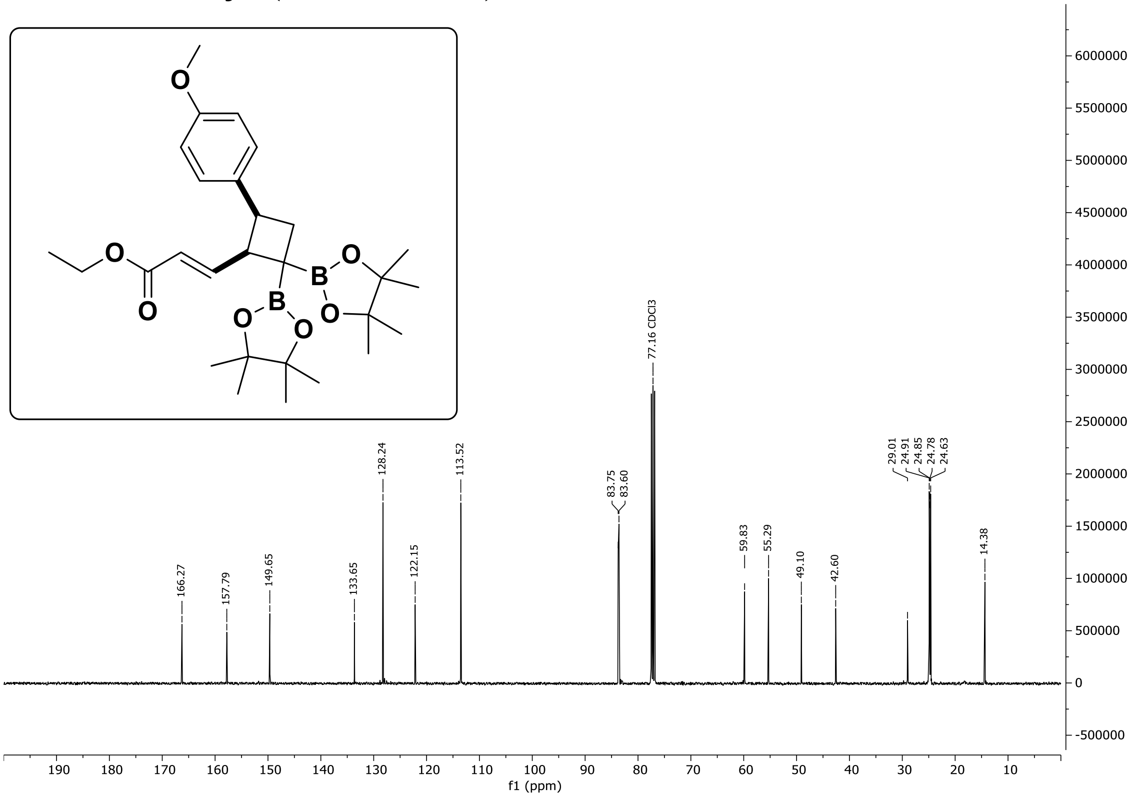

^{11}B NMR of 7-major (128 MHz, $CDCl_3$)

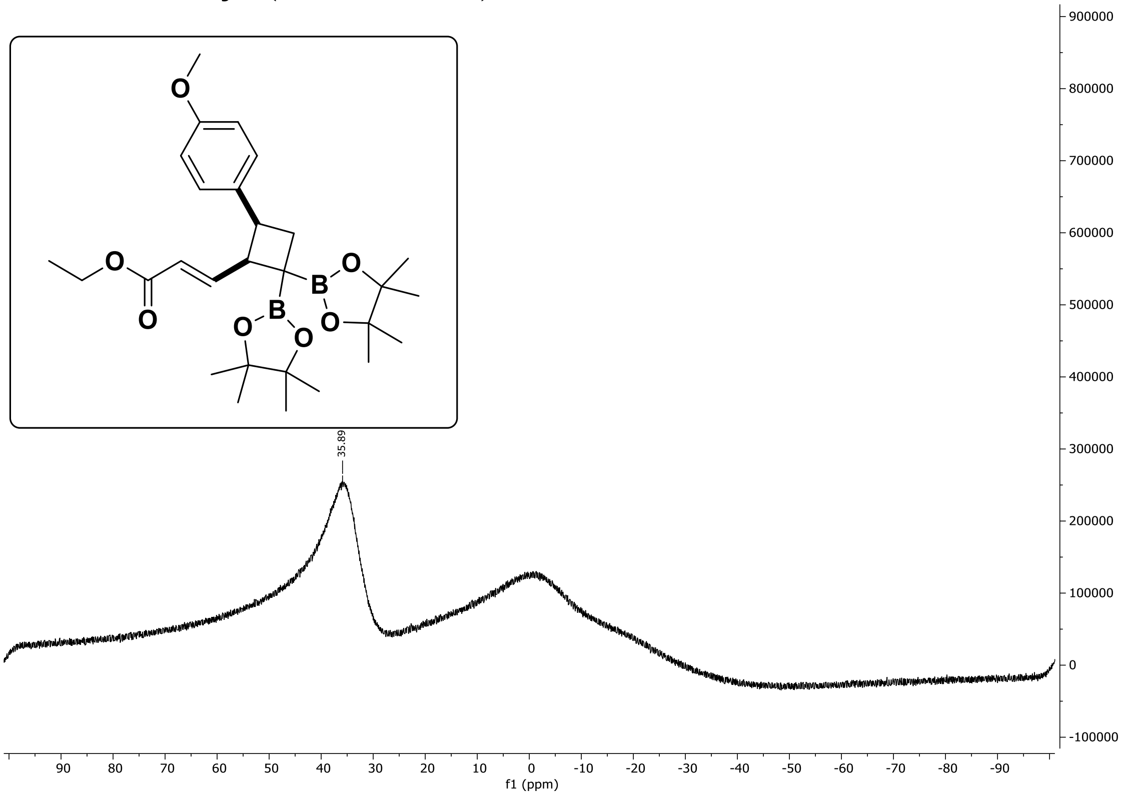

NOESY of 7-major:

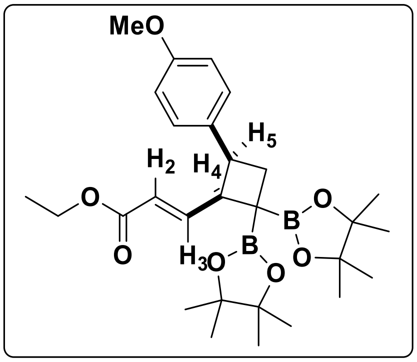

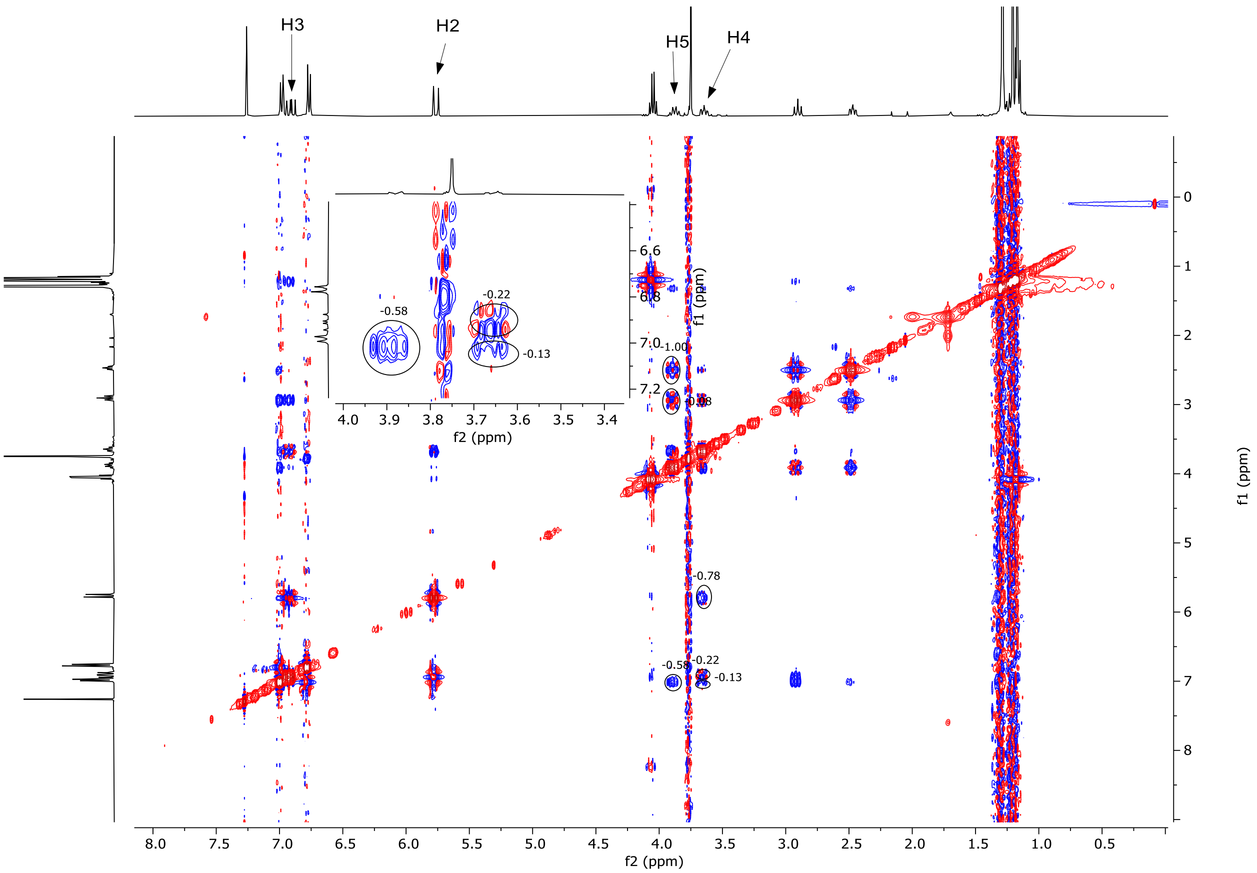

H4 has good nOe with H2 and H3, but H5 has no nOe with H2 and H3.

^{1}H-NMR of 7-minor (400 MHz, $CDCl_3$)

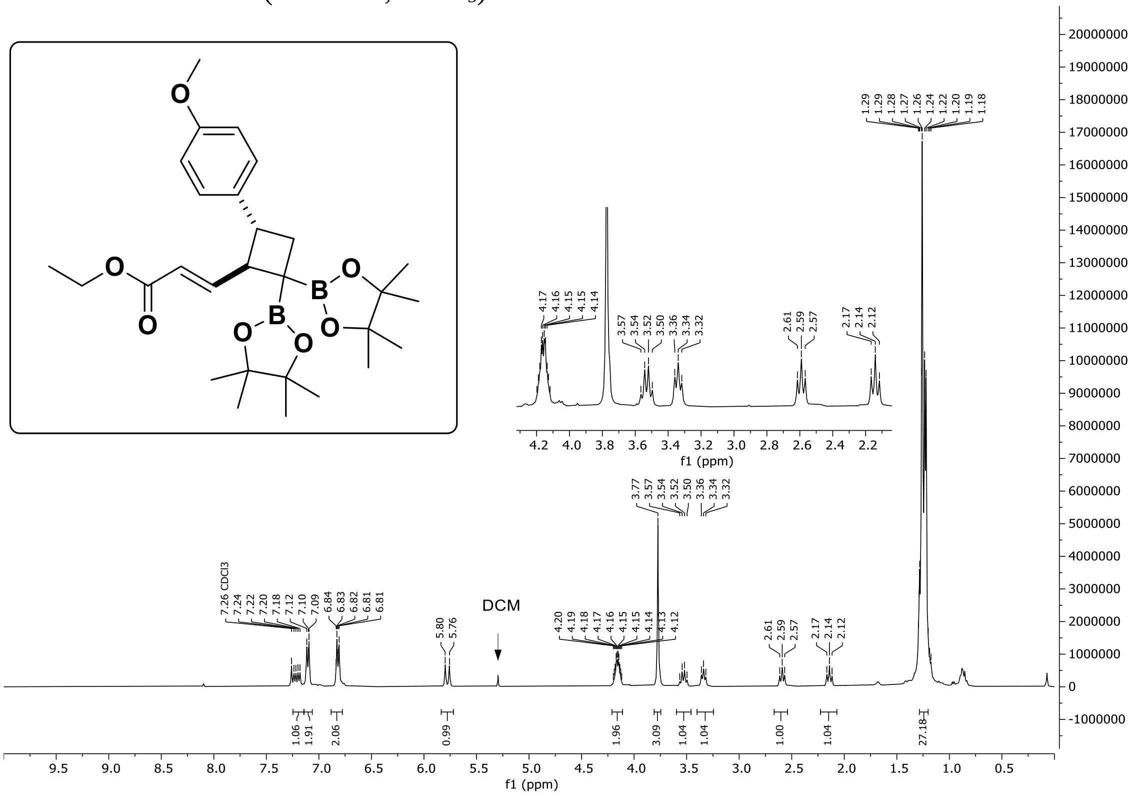

^{13}C-NMR of 7-minor (100 MHz, $CDCl_3$)

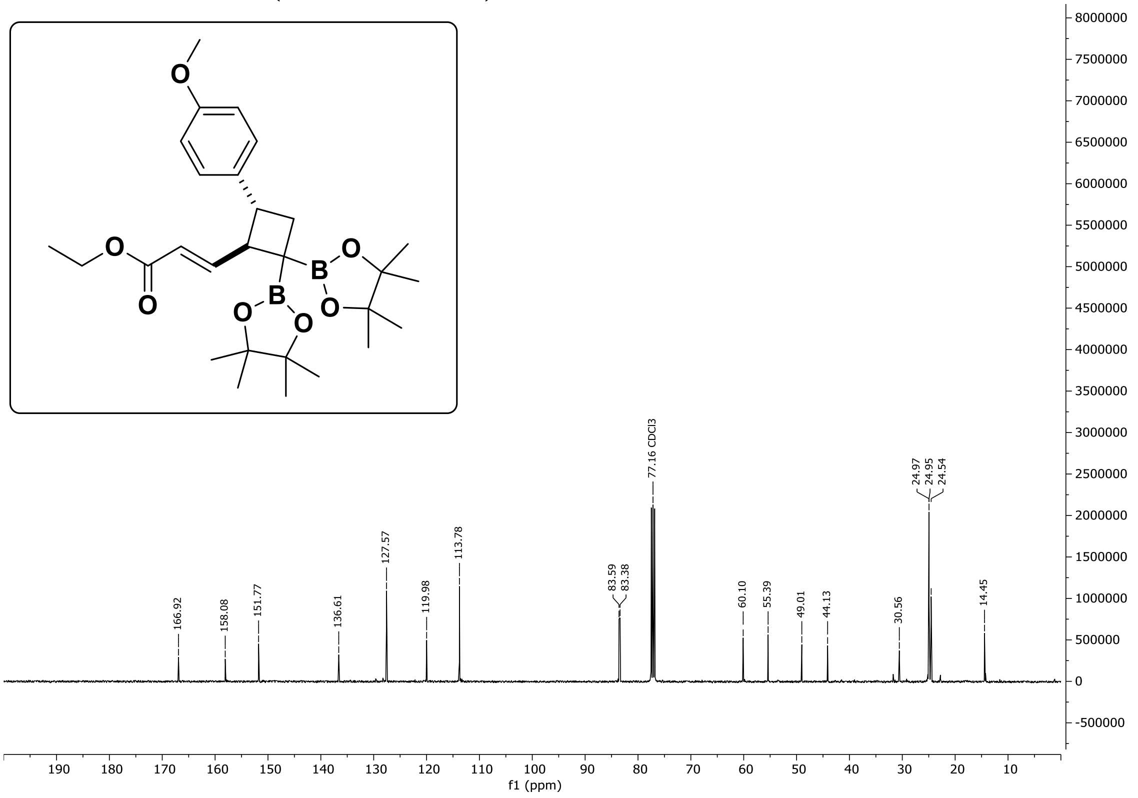

^{11}B NMR of 7-minor (128 MHz, $CDCl_3$)

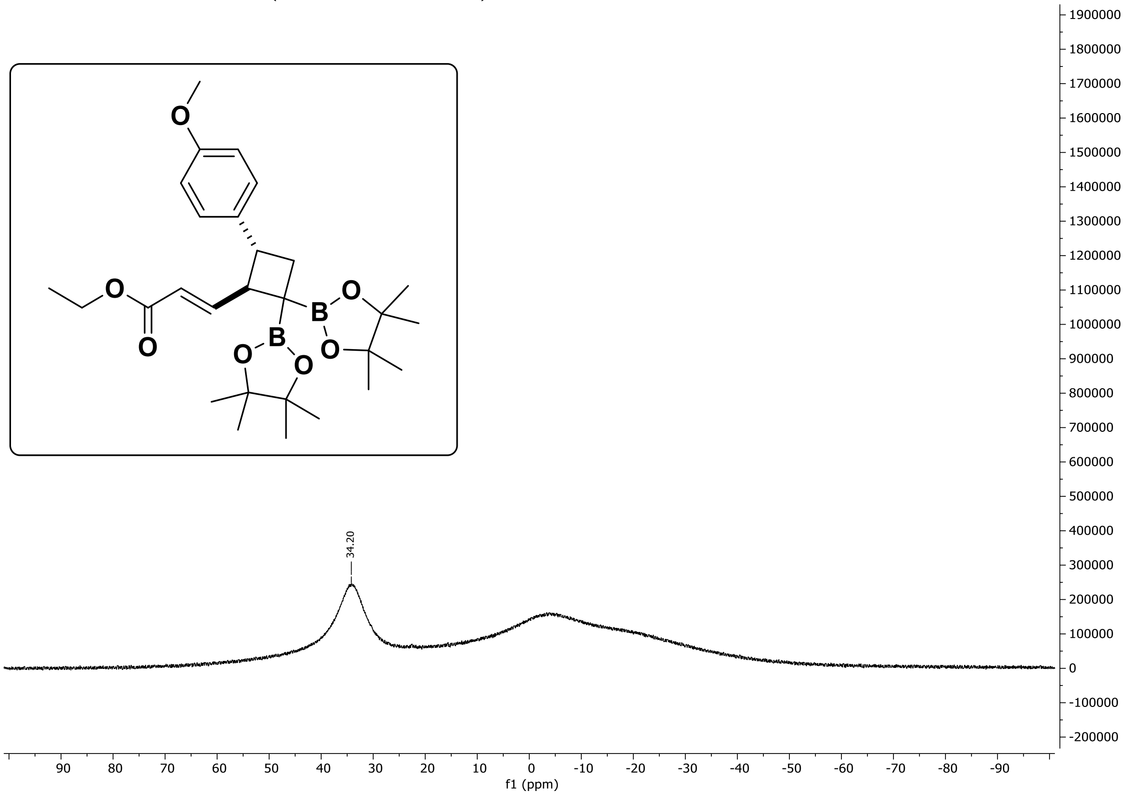

NOESY of **7-minor**:

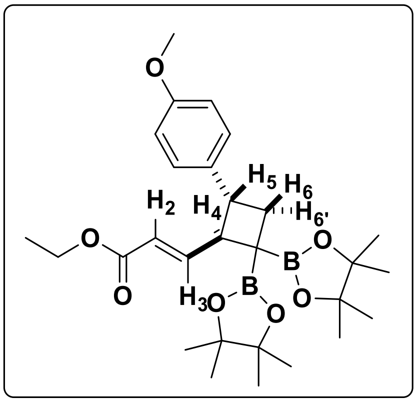

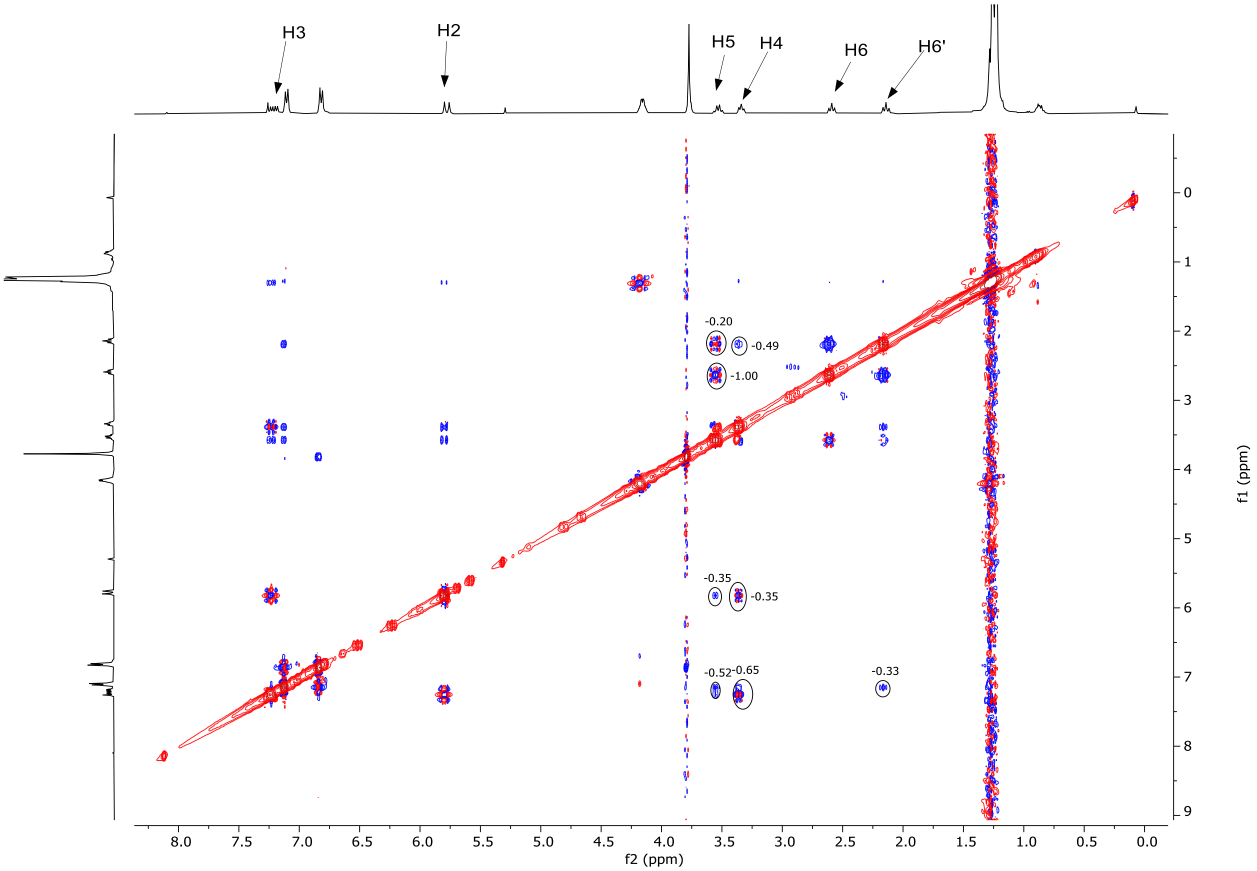

Both H4 and H5 have good nOe with H2 and H3.

H5 has good nOe with H6, but weak nOe with H6'; H4 has good nOe with H6'.

^{1}H-NMR of 8 (400 MHz, $CDCl_3$)

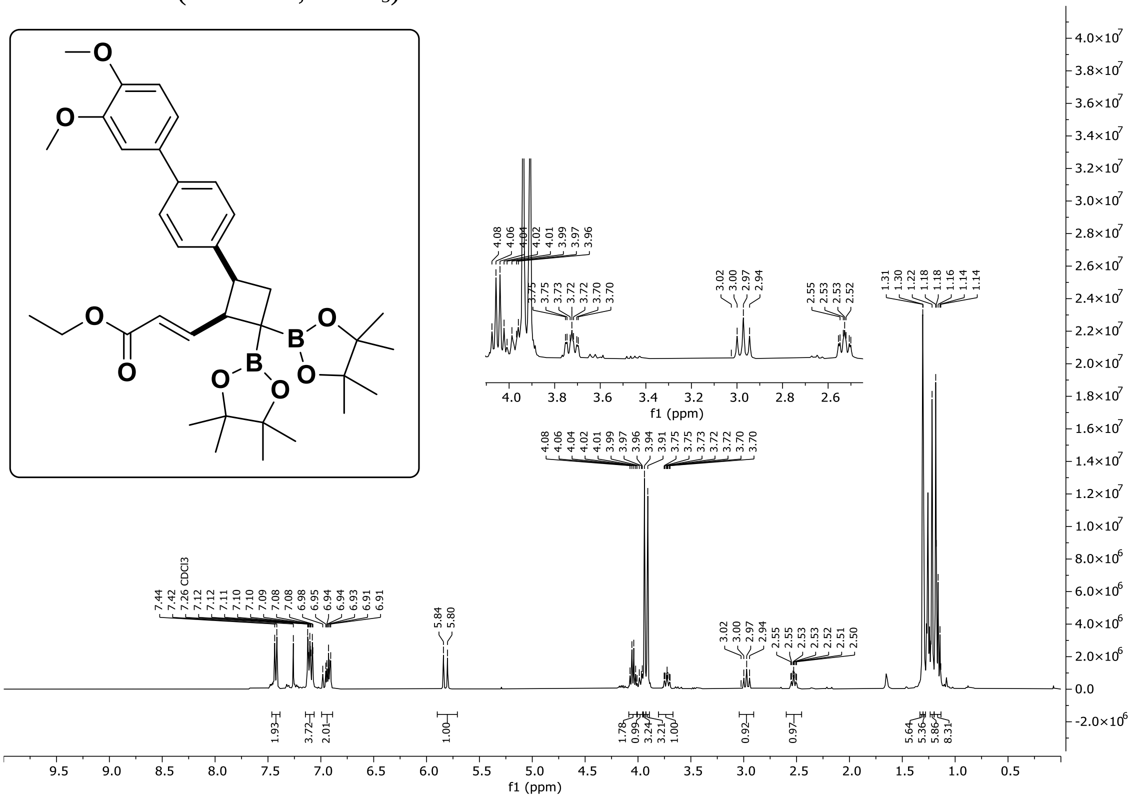

^{13}C-NMR of 8 (100 MHz, $CDCl_3$)

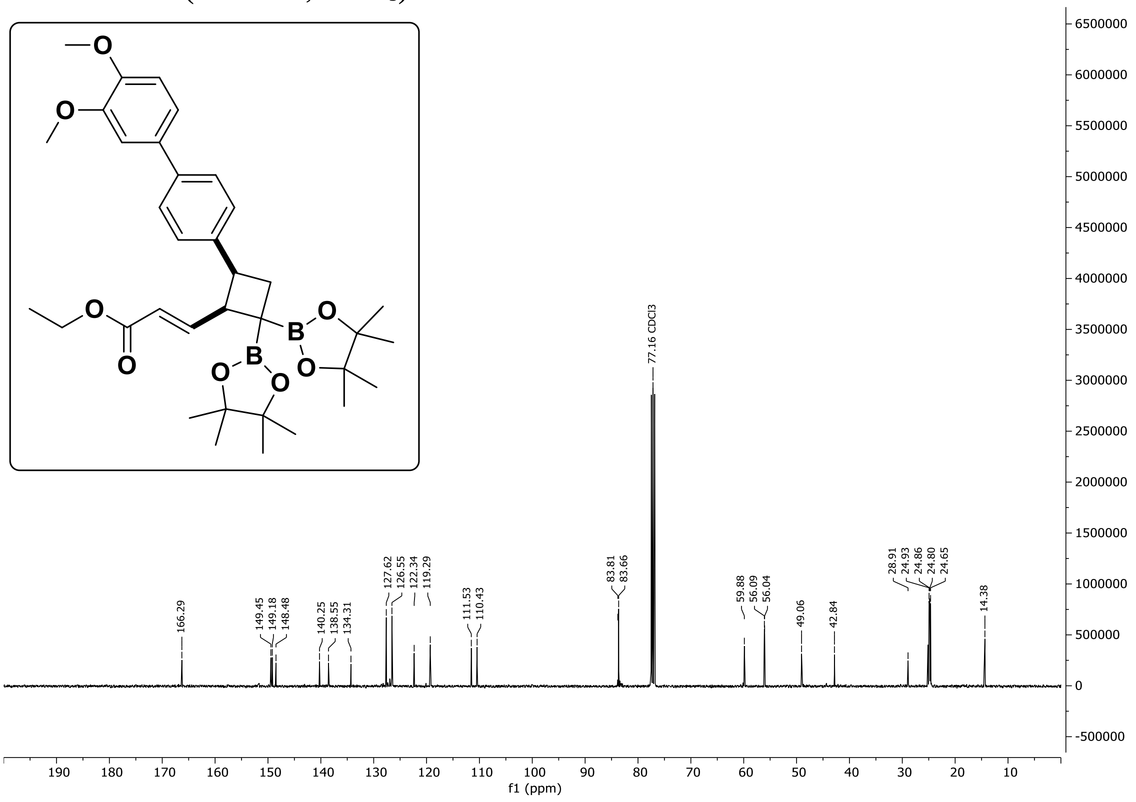

^{11}**B NMR of 8** (128 MHz, $CDCl_3$)

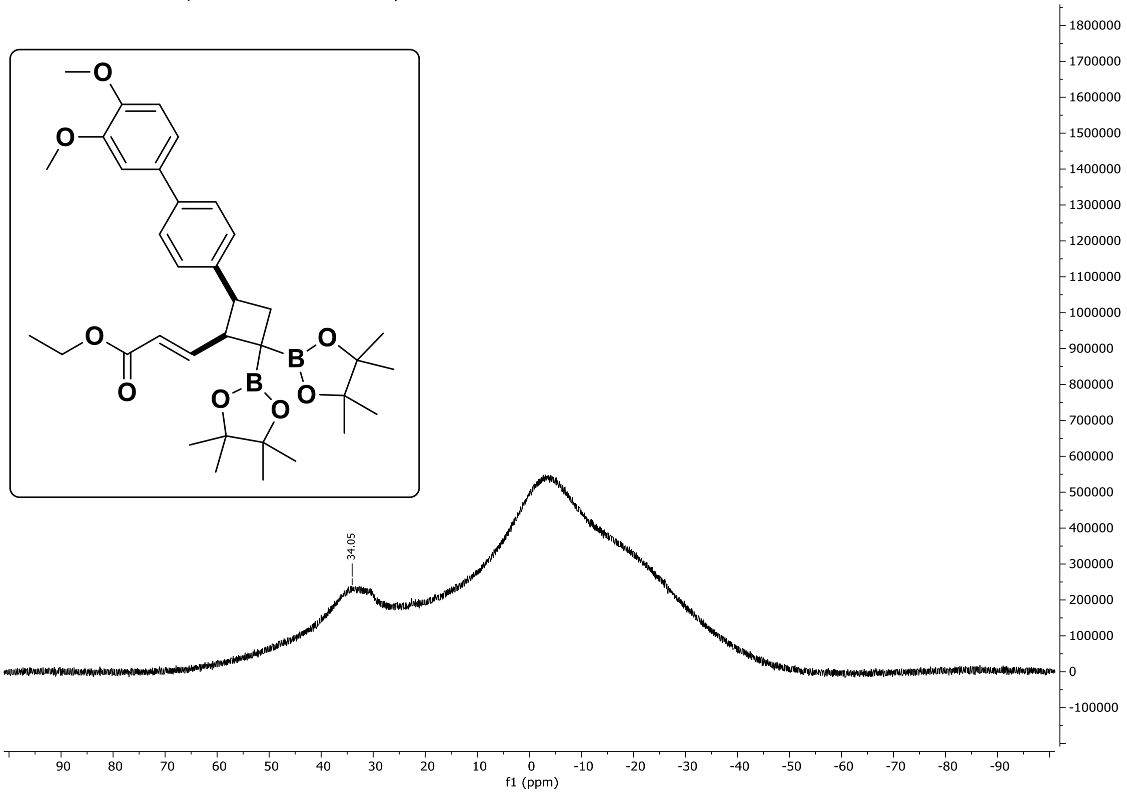

^{1}**H-NMR of 9-major** (400 MHz, $CDCl_3$)

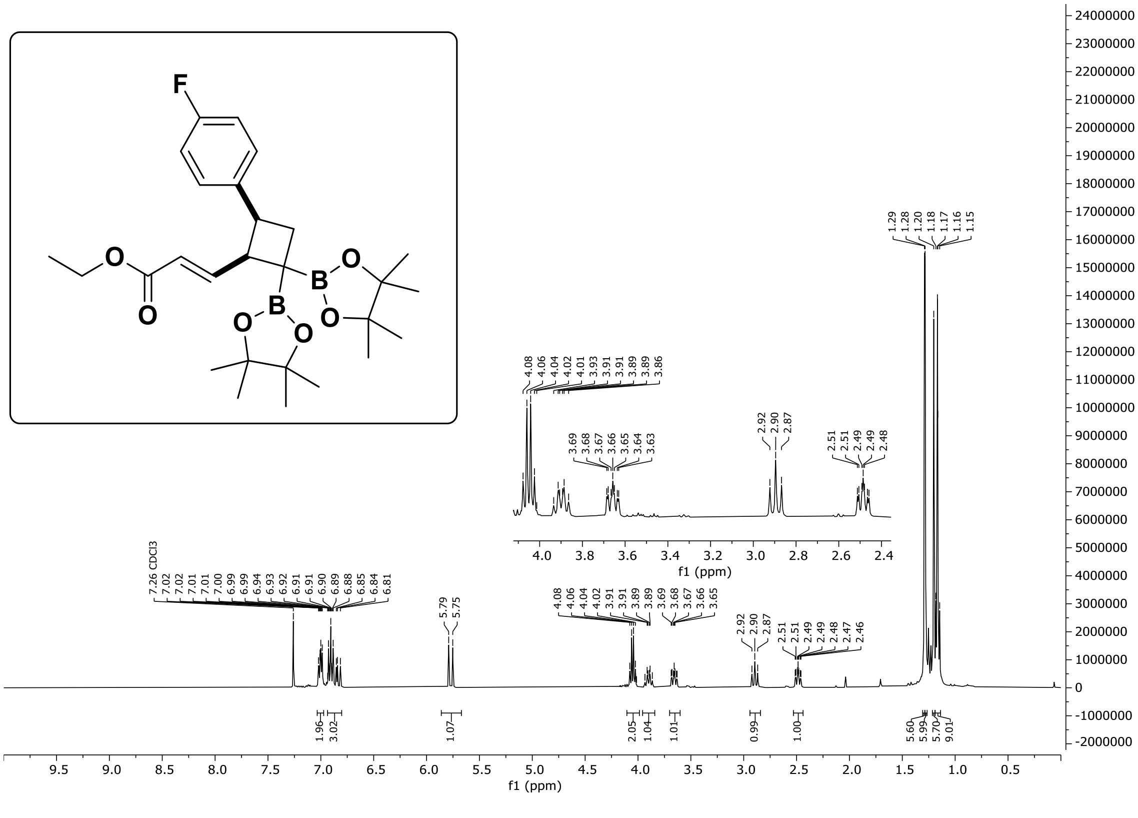

^{13}C-NMR of 9-major (100 MHz, $CDCl_3$)

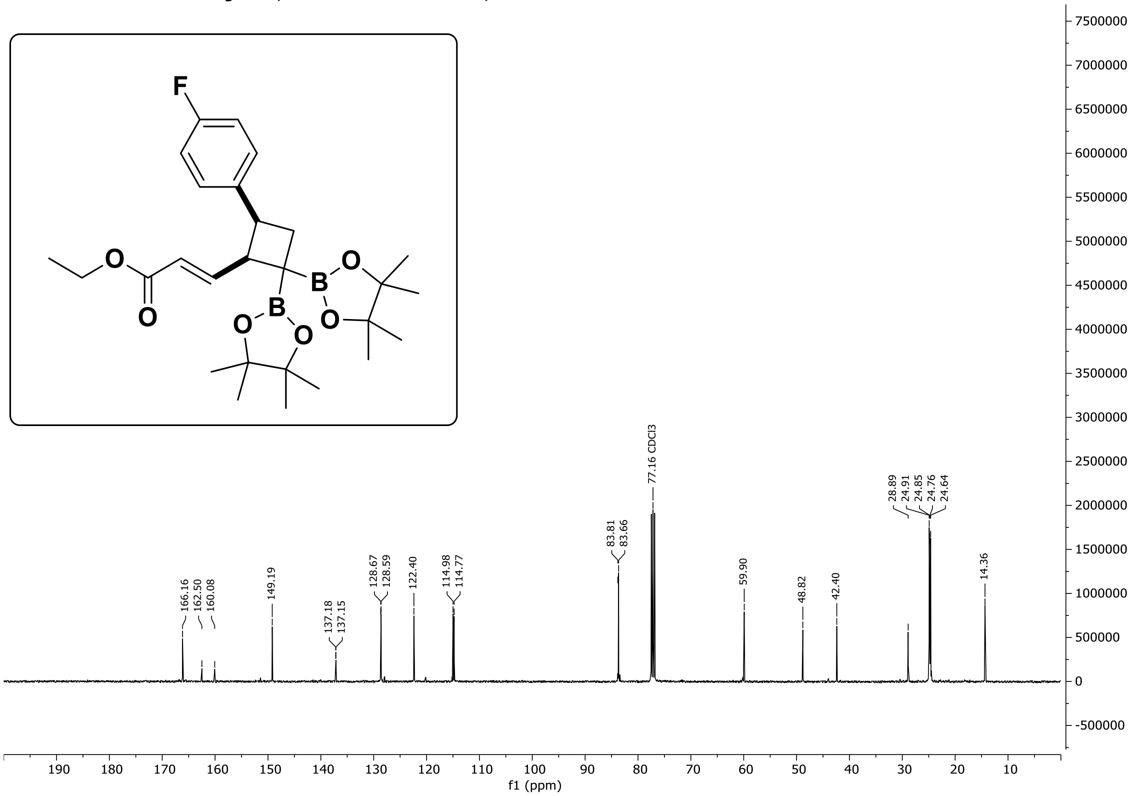

^{19}F-NMR of 9-major (376 MHz, $CDCl_3$)

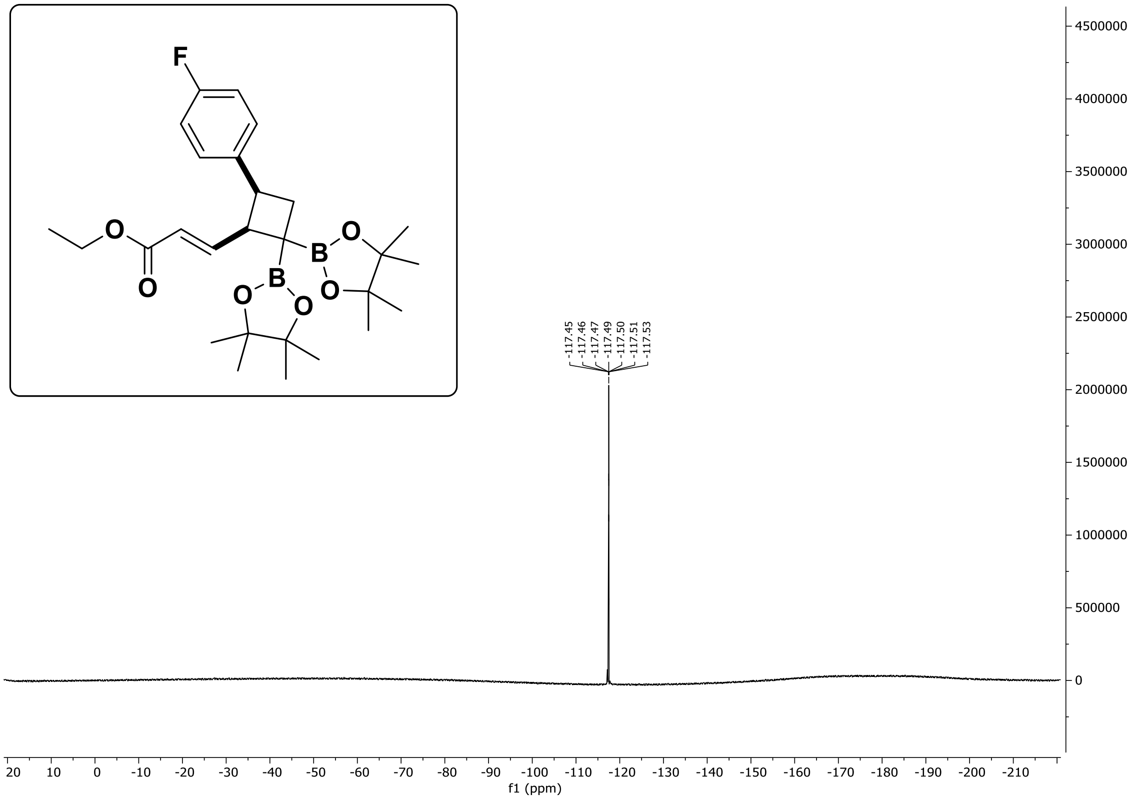

^{11}B NMR of 9-major (128 MHz, $CDCl_3$)

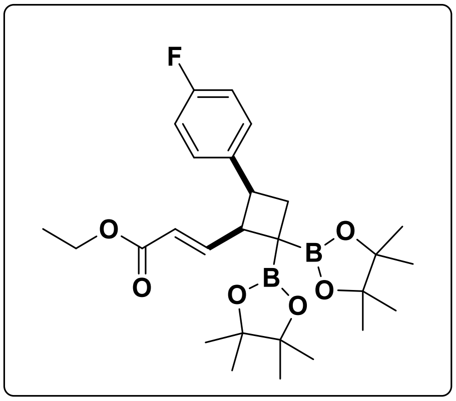

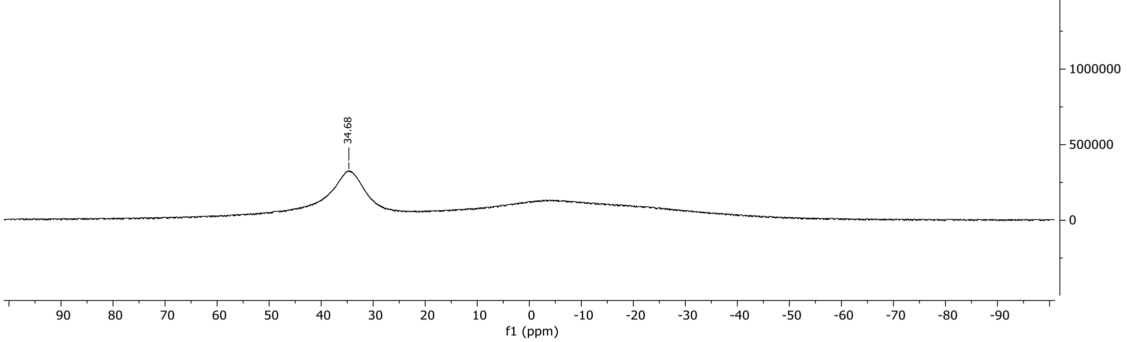

NOESY of 9-major isomer:

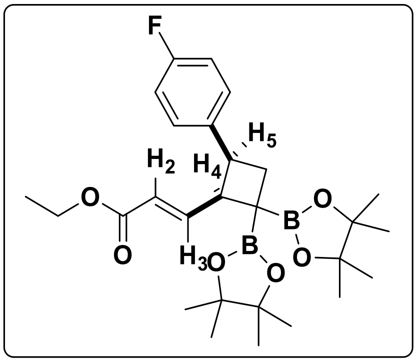

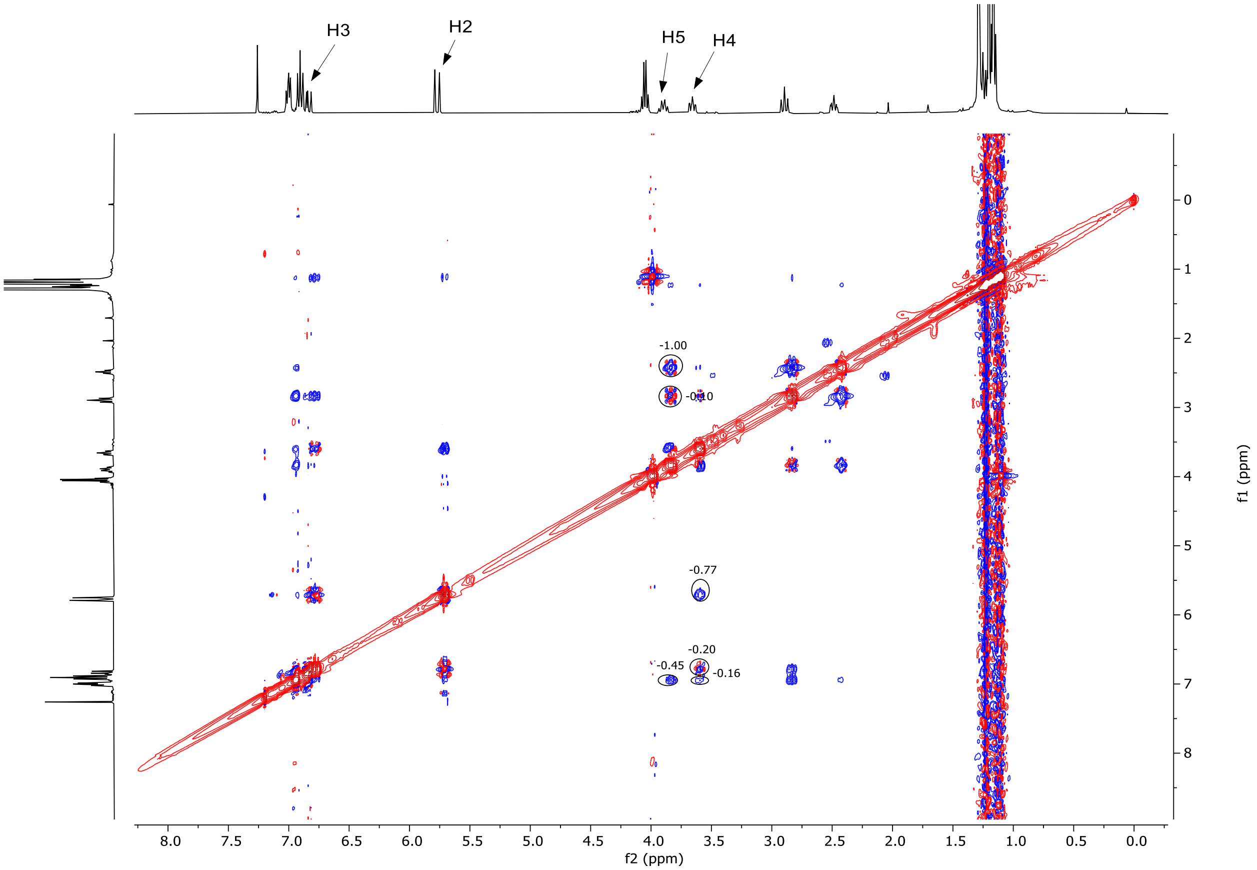

H4 has good nOe with H2 and H3, but H5 has no nOe with H2 and H3.

^{1}H-NMR of 9-minor (400 MHz, $CDCl_3$)

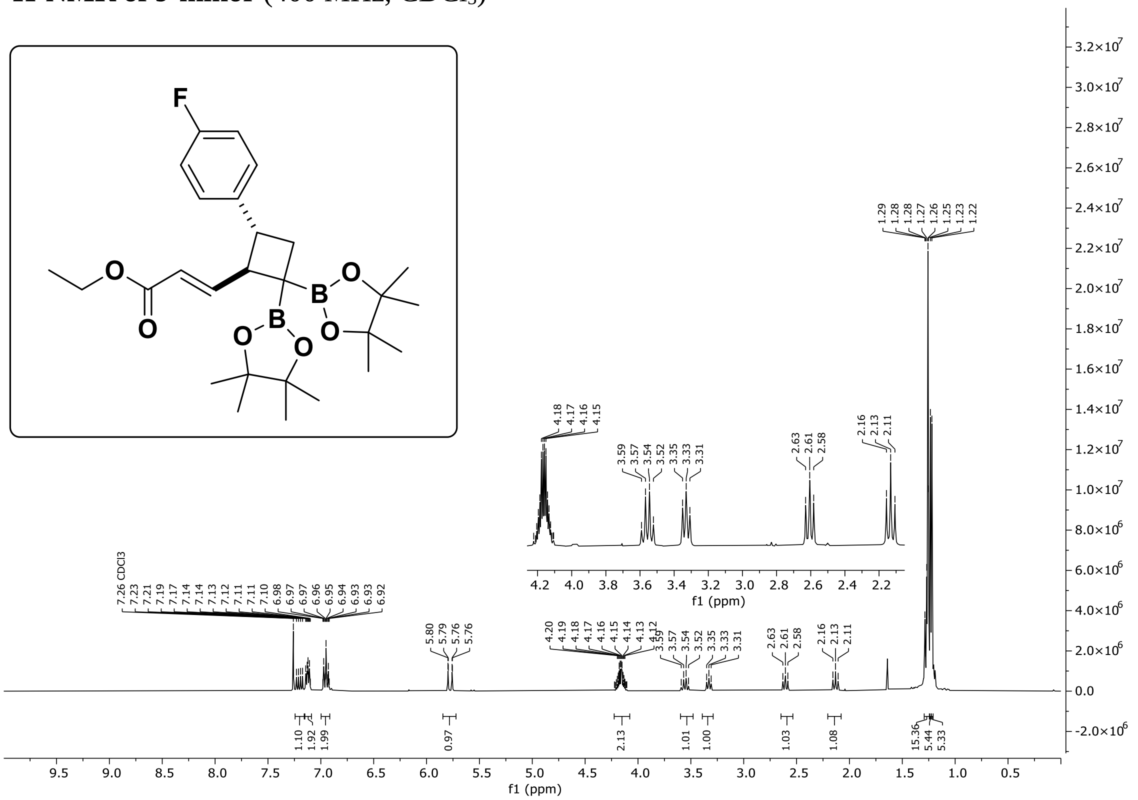

^{13}C-NMR of 9-minor (100 MHz, $CDCl_3$)

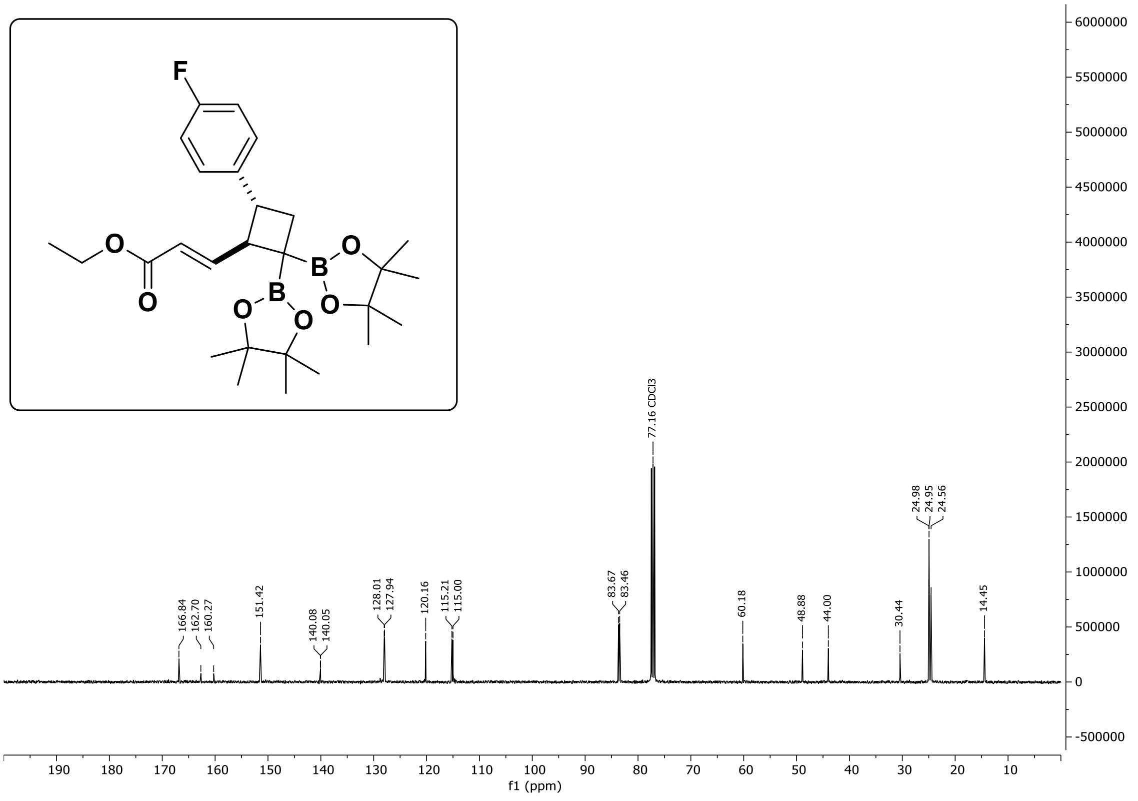

^{19}F-NMR of 9-minor (376 MHz, $CDCl_3$)

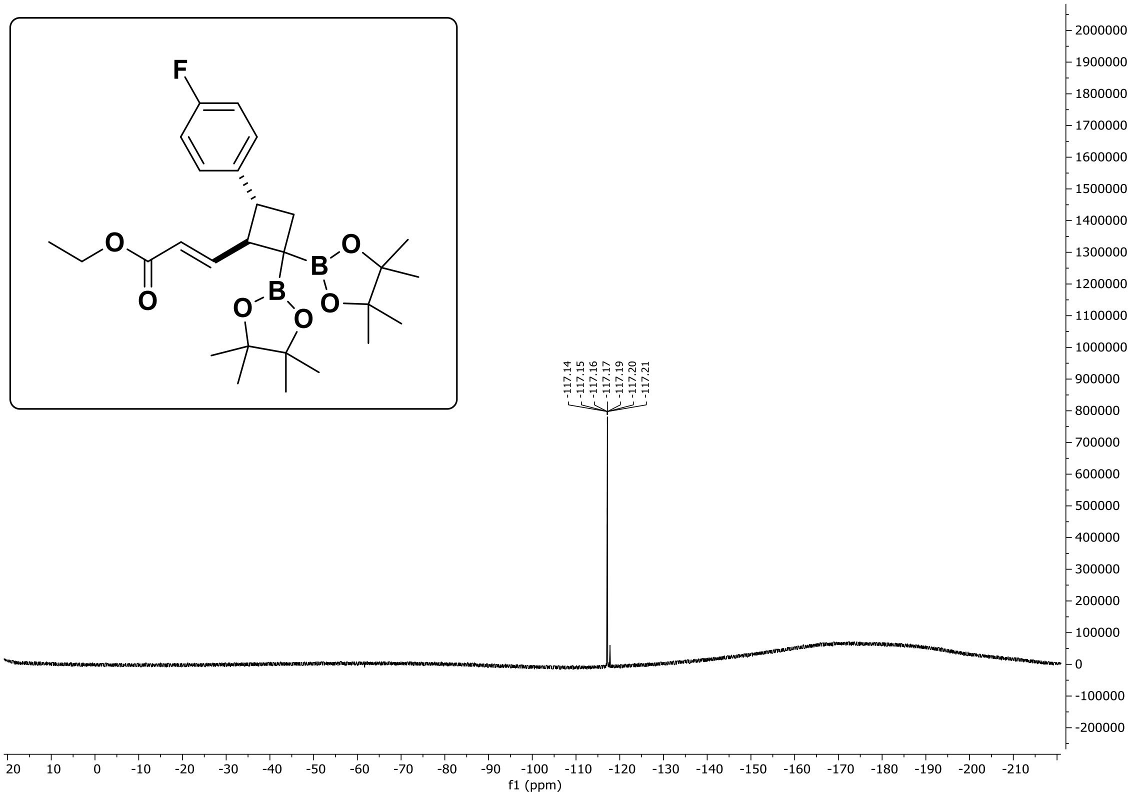

^{11}B NMR of 9-minor (128 MHz, $CDCl_3$)

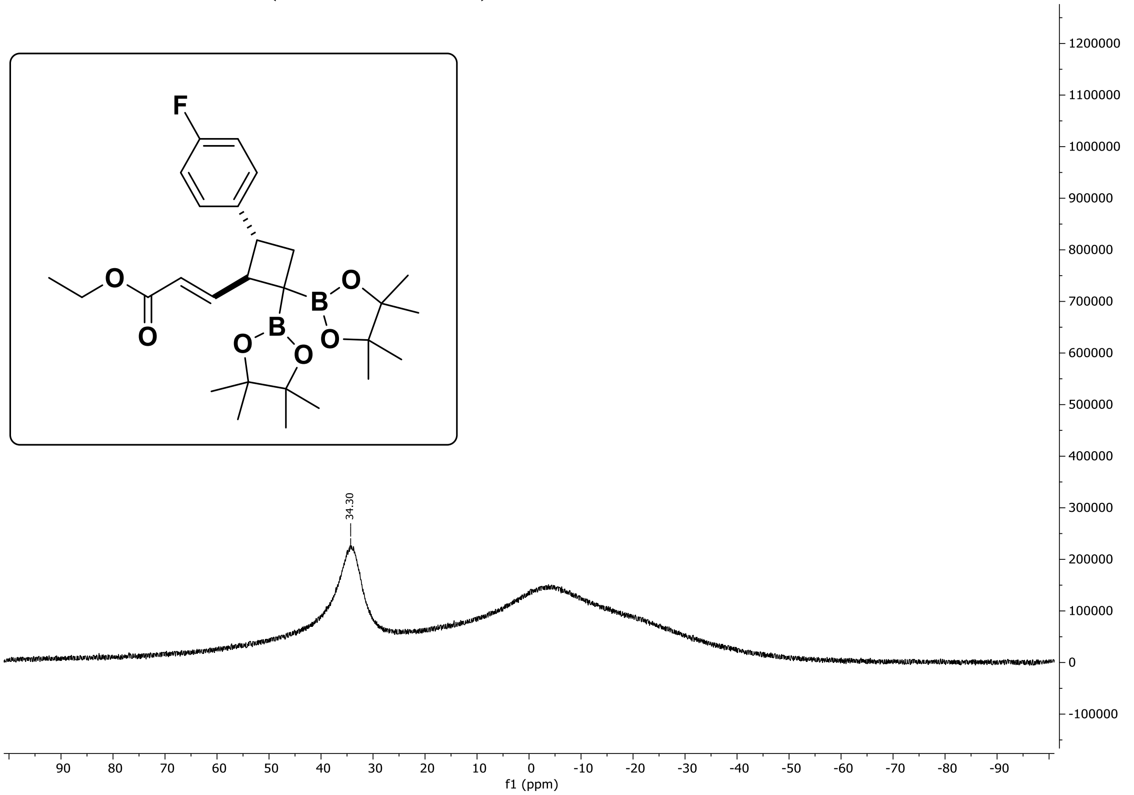

NOESY of 9-minor isomer:

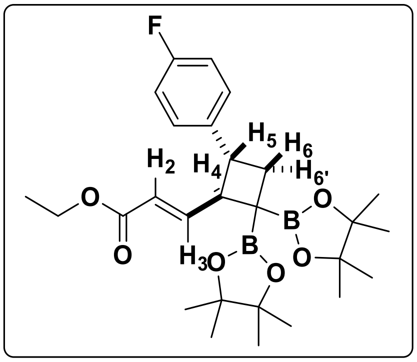

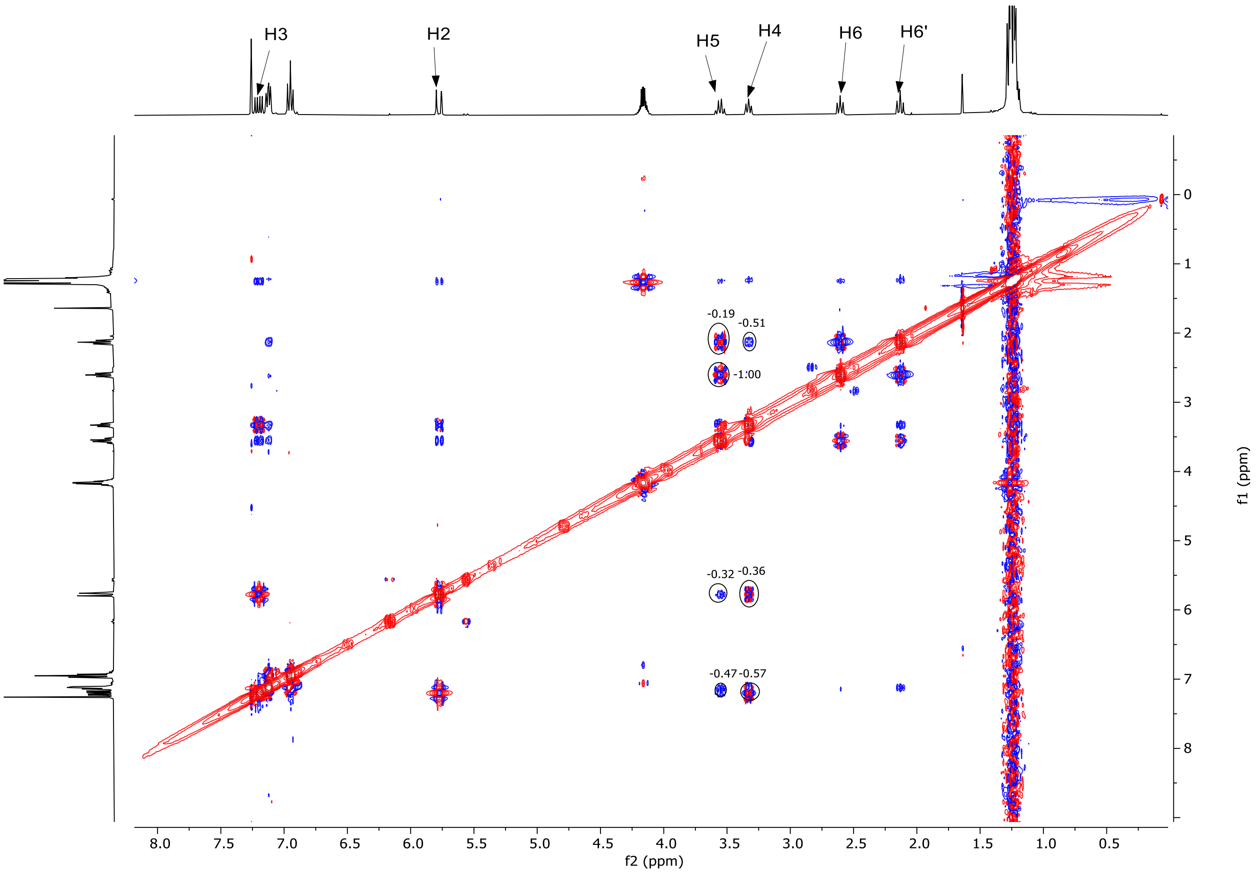

Both H4 and H5 have good nOe with H2 and H3.

H5 has good nOe with H6, but weak nOe with H6'; H4 has good nOe with H6'.

^{1}H-NMR of 10 (400 MHz, $CDCl_3$)

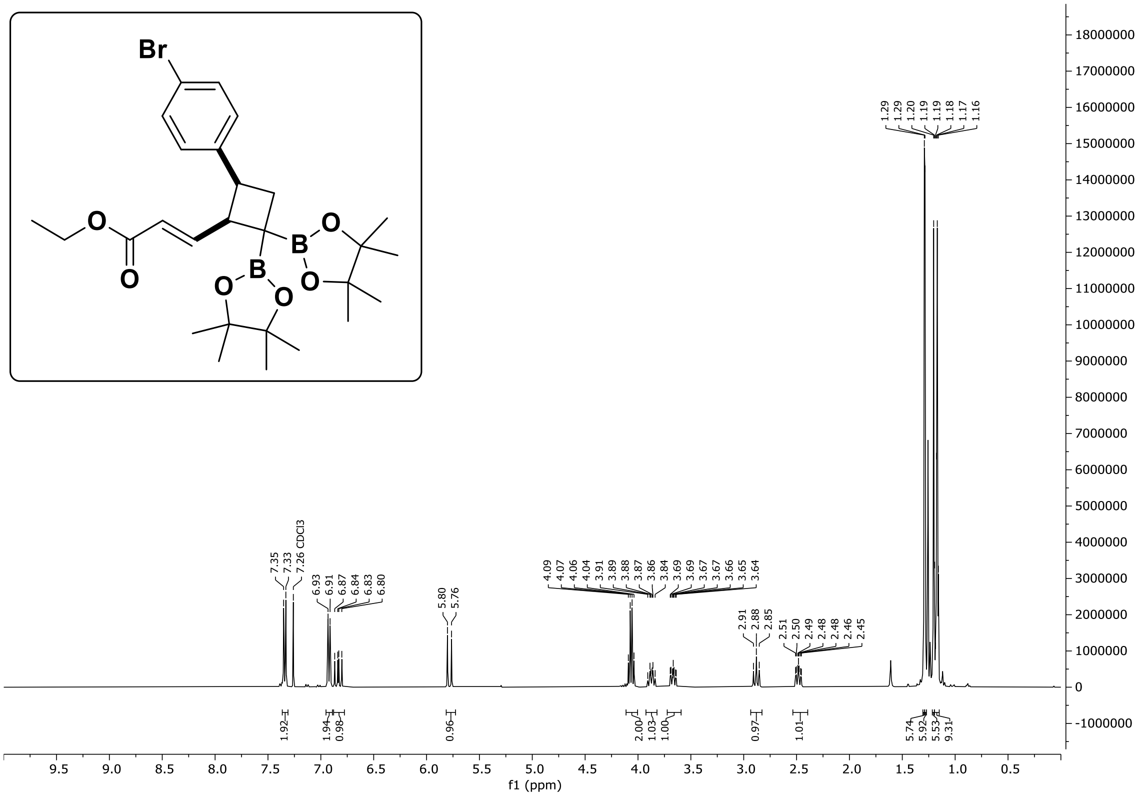

^{13}C-NMR of 10 (100 MHz, $CDCl_3$)

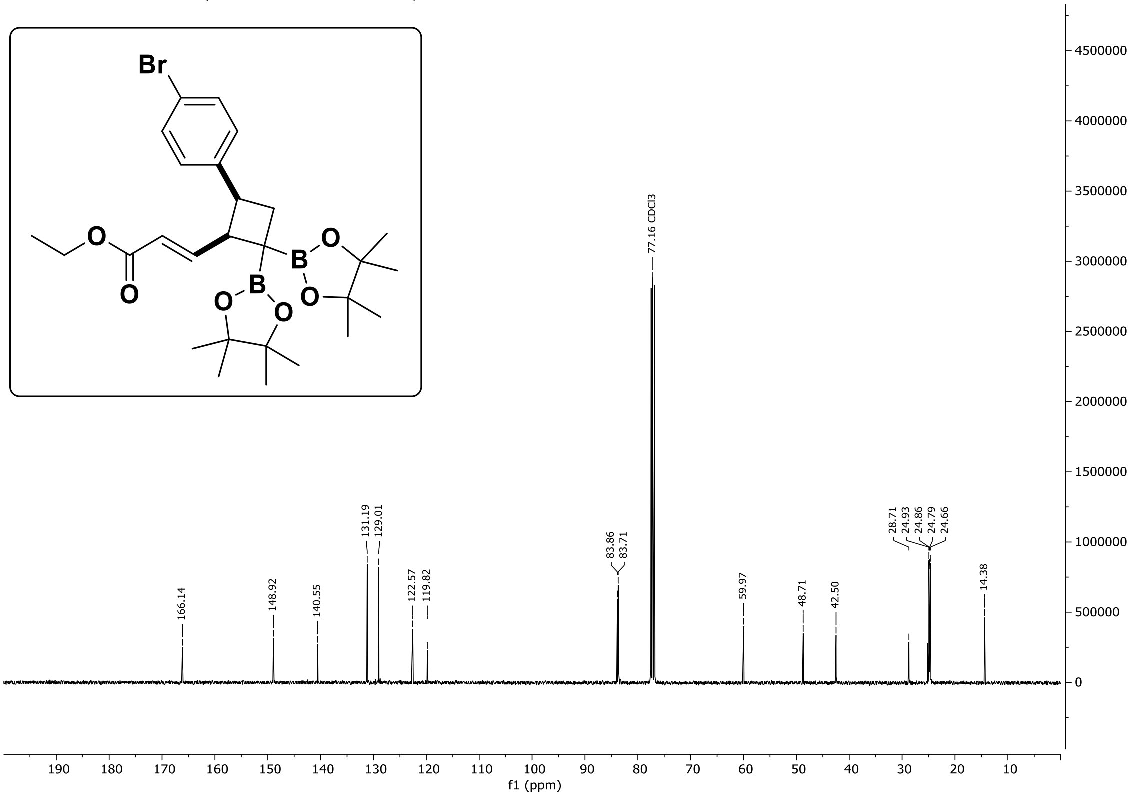

^{11}B NMR of 10 (128 MHz, $CDCl_3$)

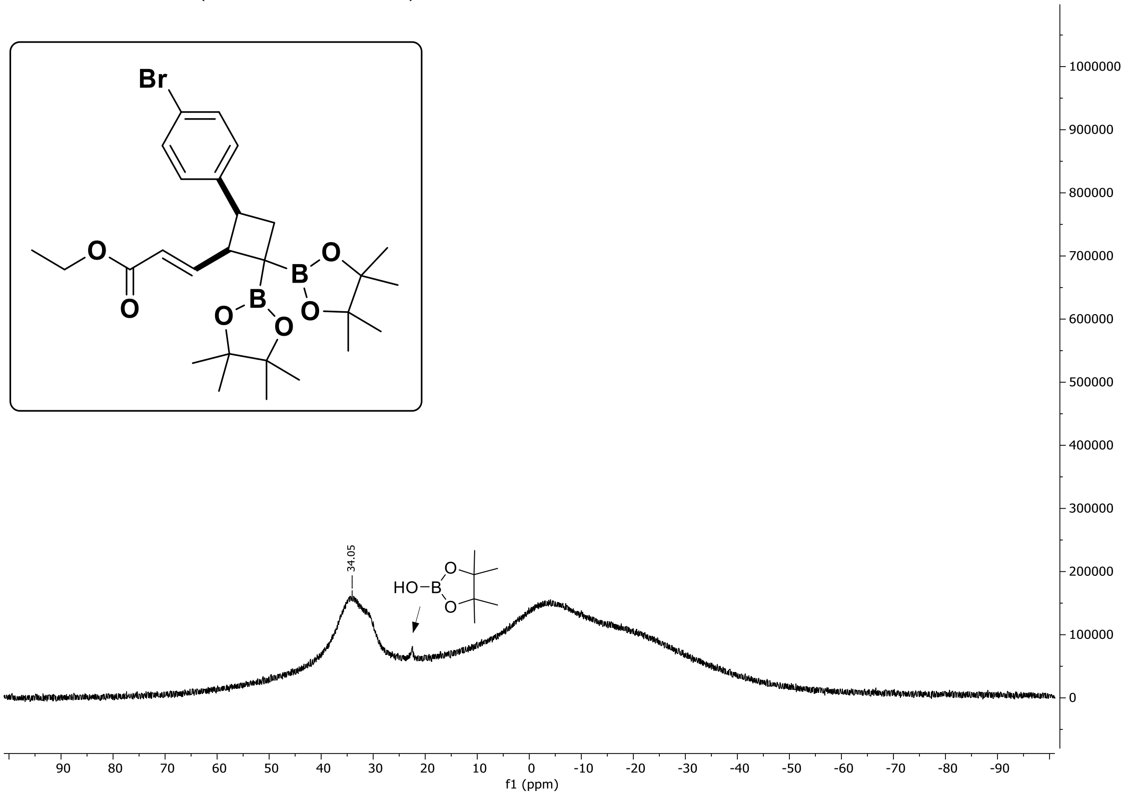

^{1}H-NMR of 11 (400 MHz, $CDCl_3$)

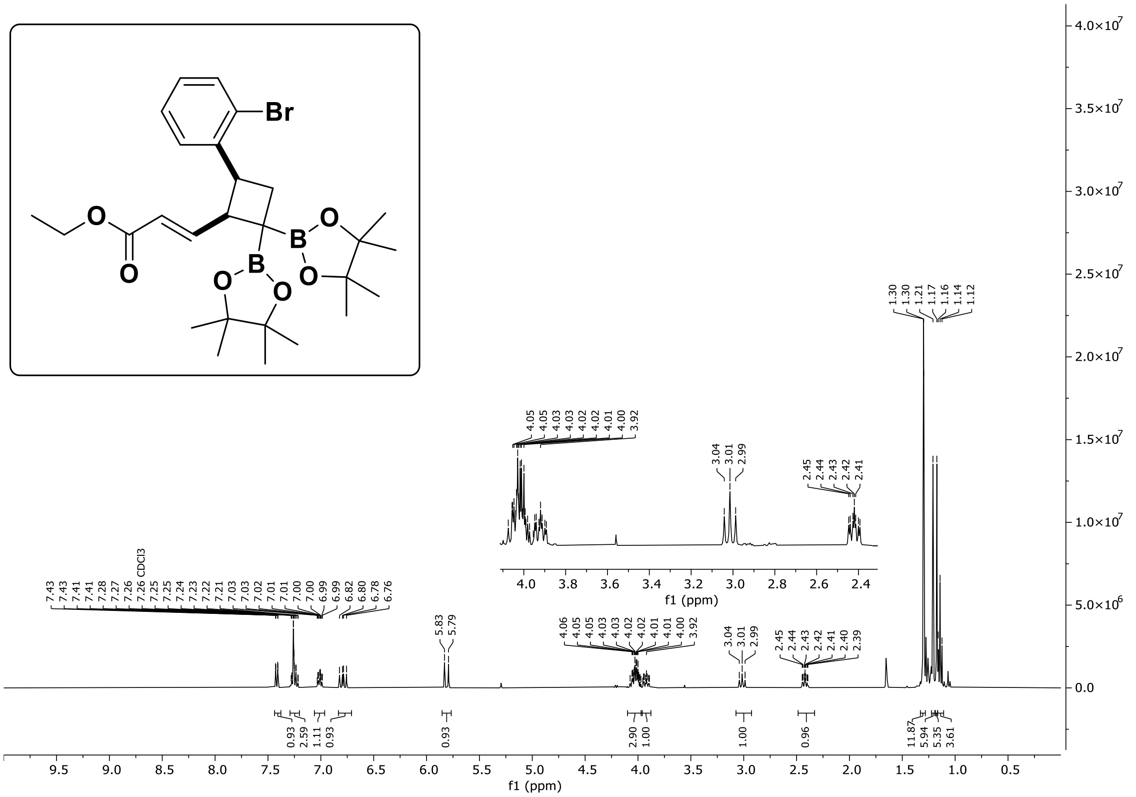

^{13}C-NMR of 11 (100 MHz, $CDCl_3$)

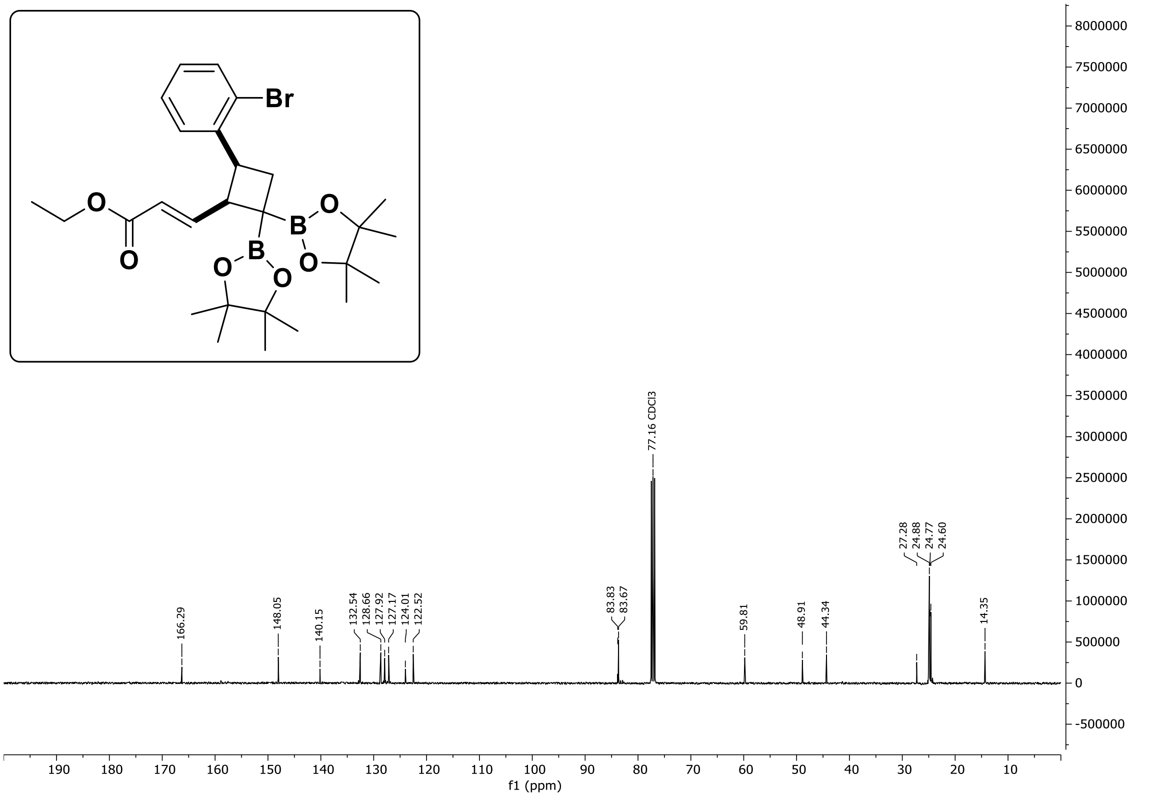

^{11}B NMR of 11 (128 MHz, $CDCl_3$)

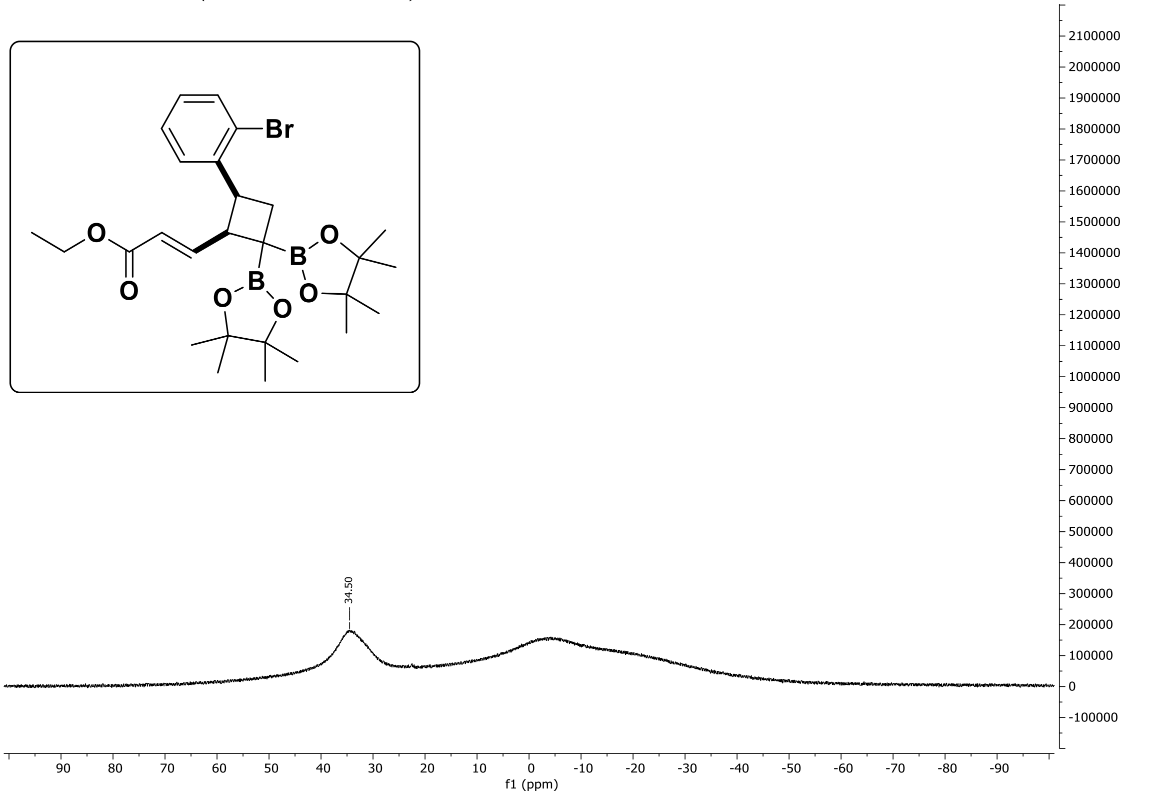

^{1}H-NMR of 12-major (400 MHz, $CDCl_3$)

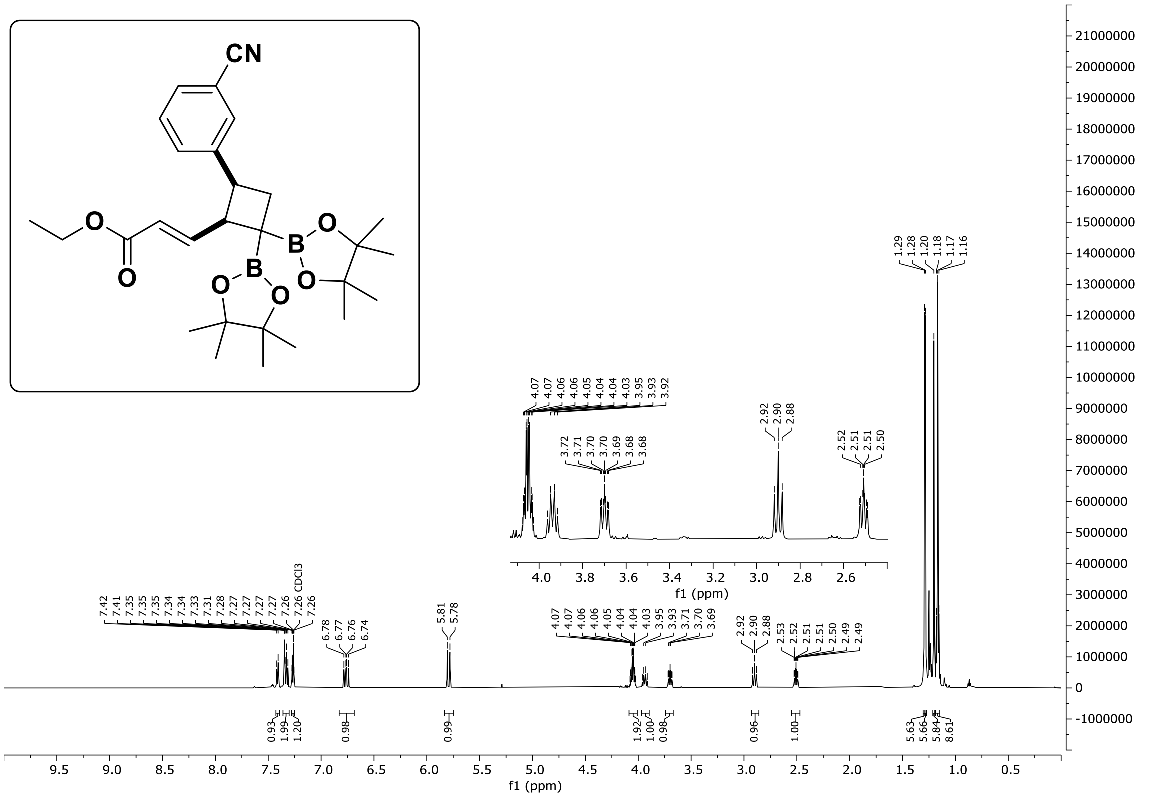

^{13}C-NMR of 12-major (100 MHz, $CDCl_3$)

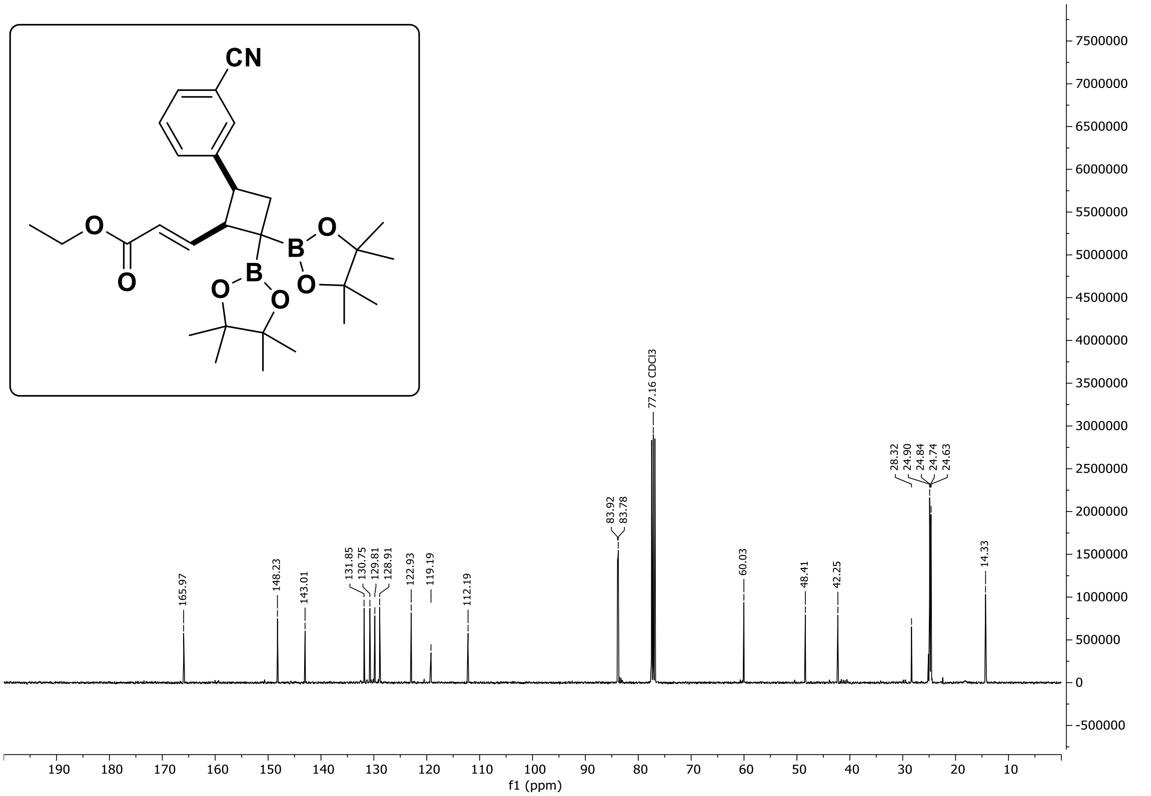

^{11}B NMR of 12-major (128 MHz, $CDCl_3$)

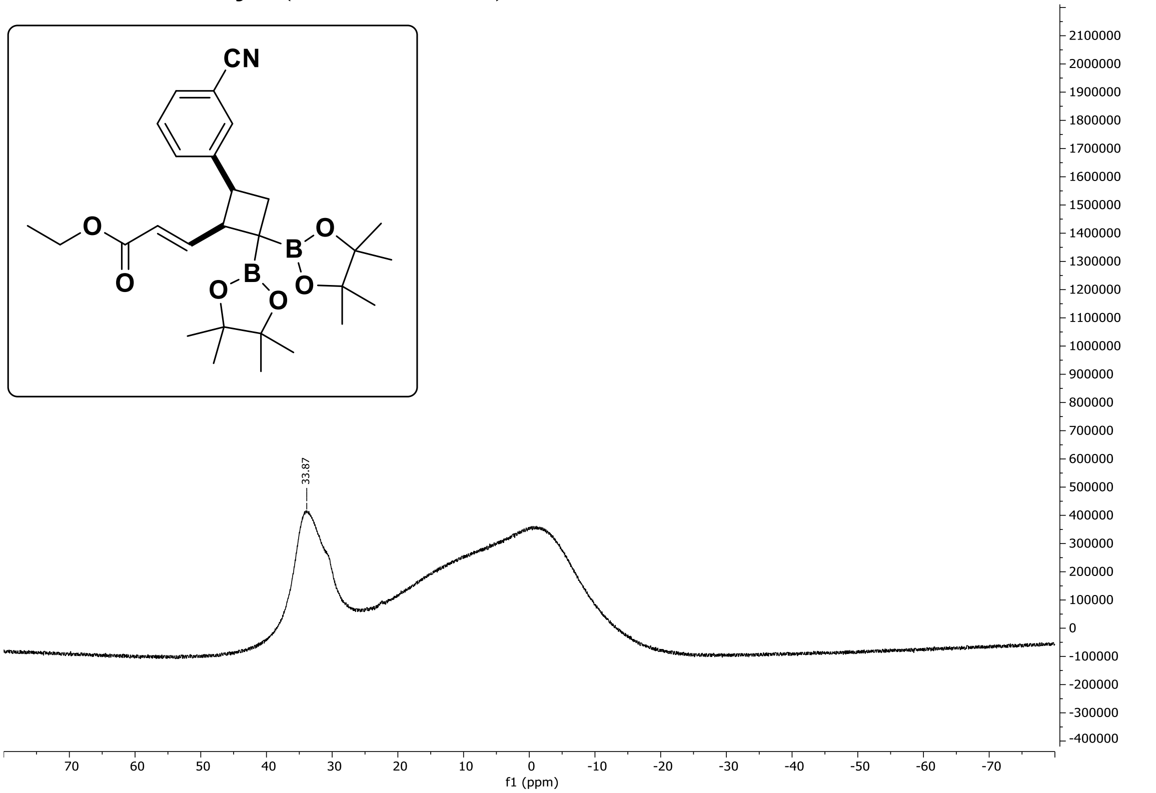

^{1}H-NMR of 12-minor (400 MHz, $CDCl_3$)

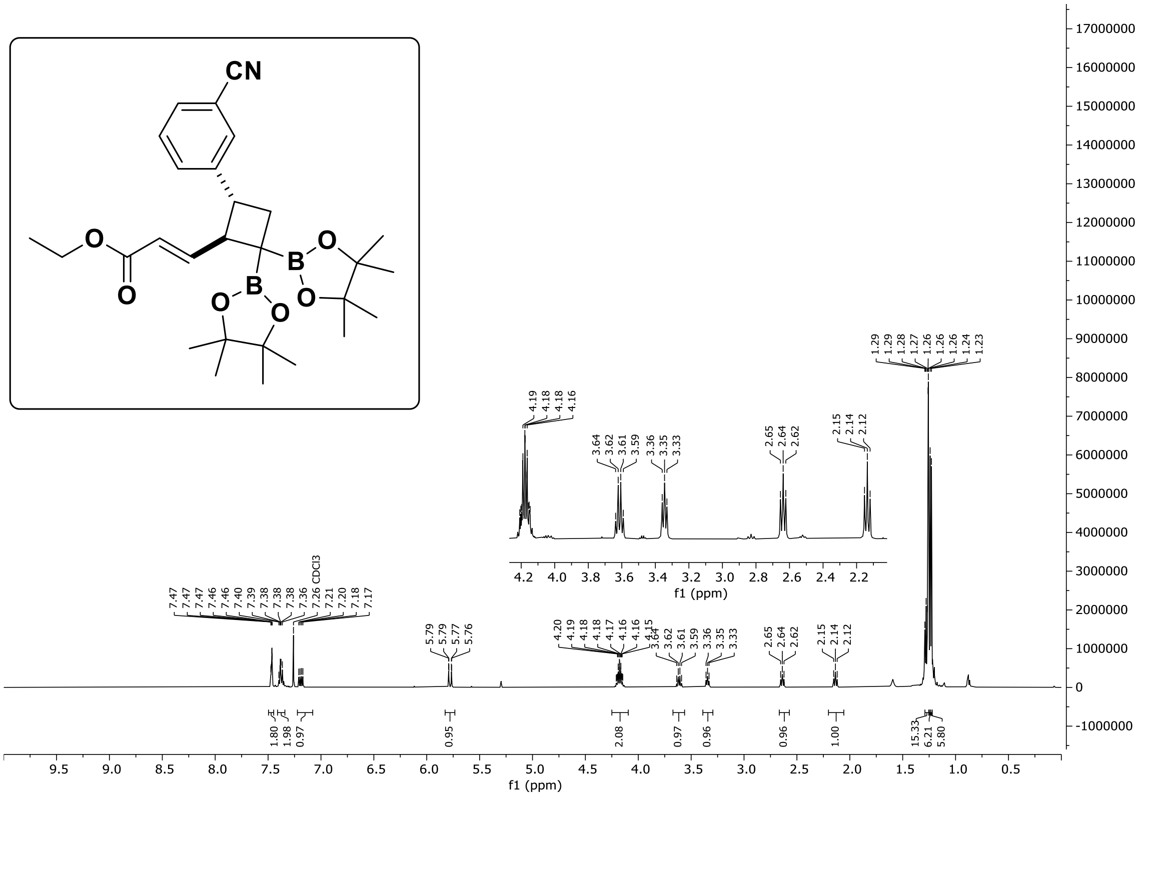

^{13}C-NMR of 12-minor (151 MHz, $CDCl_3$)

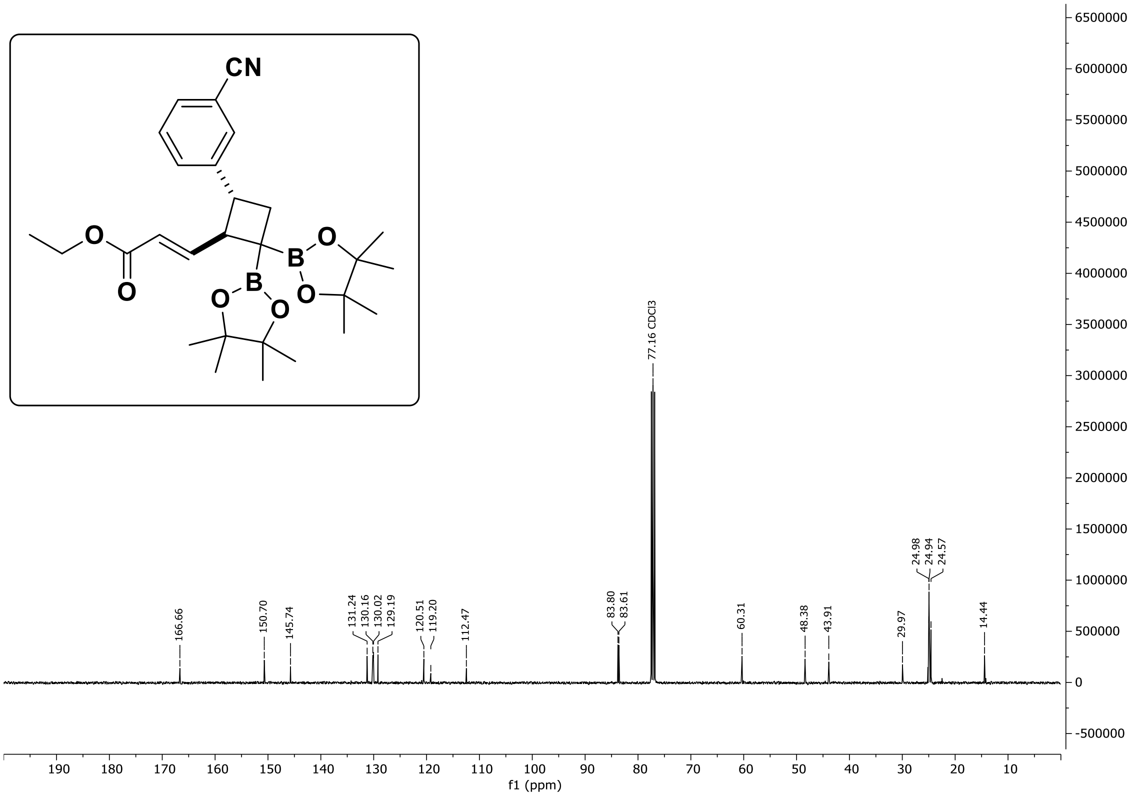

^{11}B NMR of 12-minor (128 MHz, $CDCl_3$)

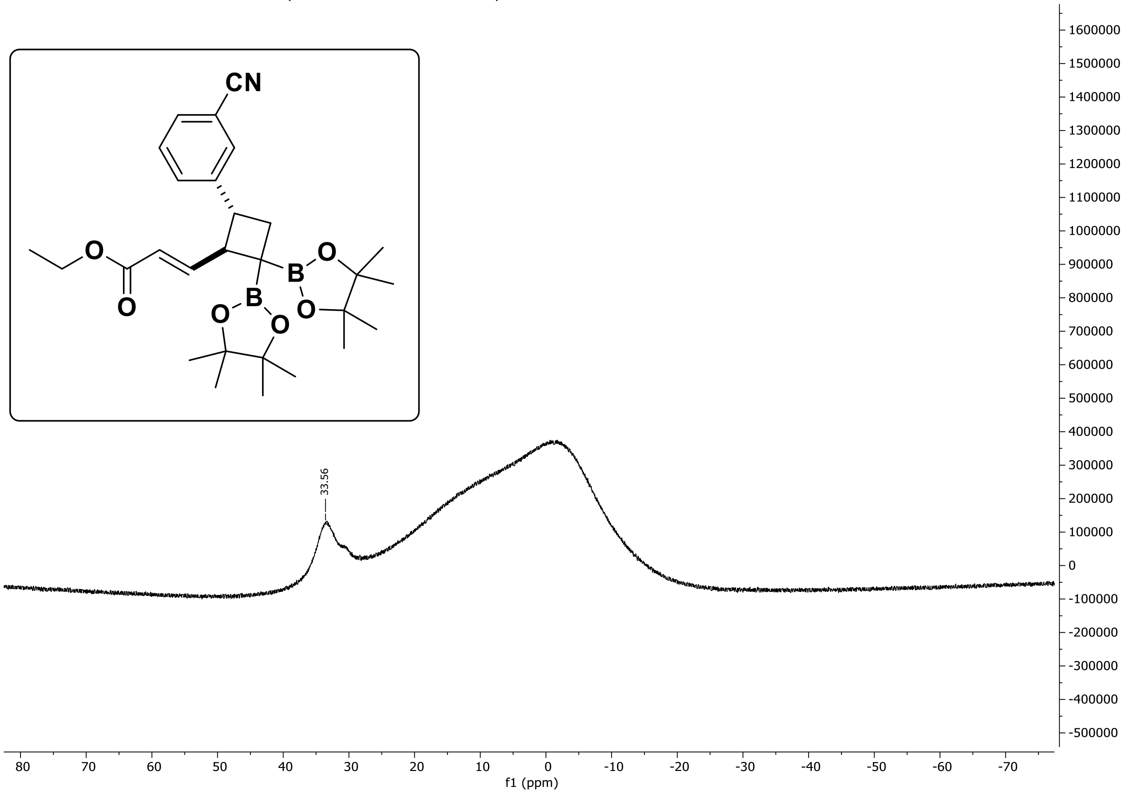

^{1}H-NMR of 13 (400 MHz, $CDCl_3$)

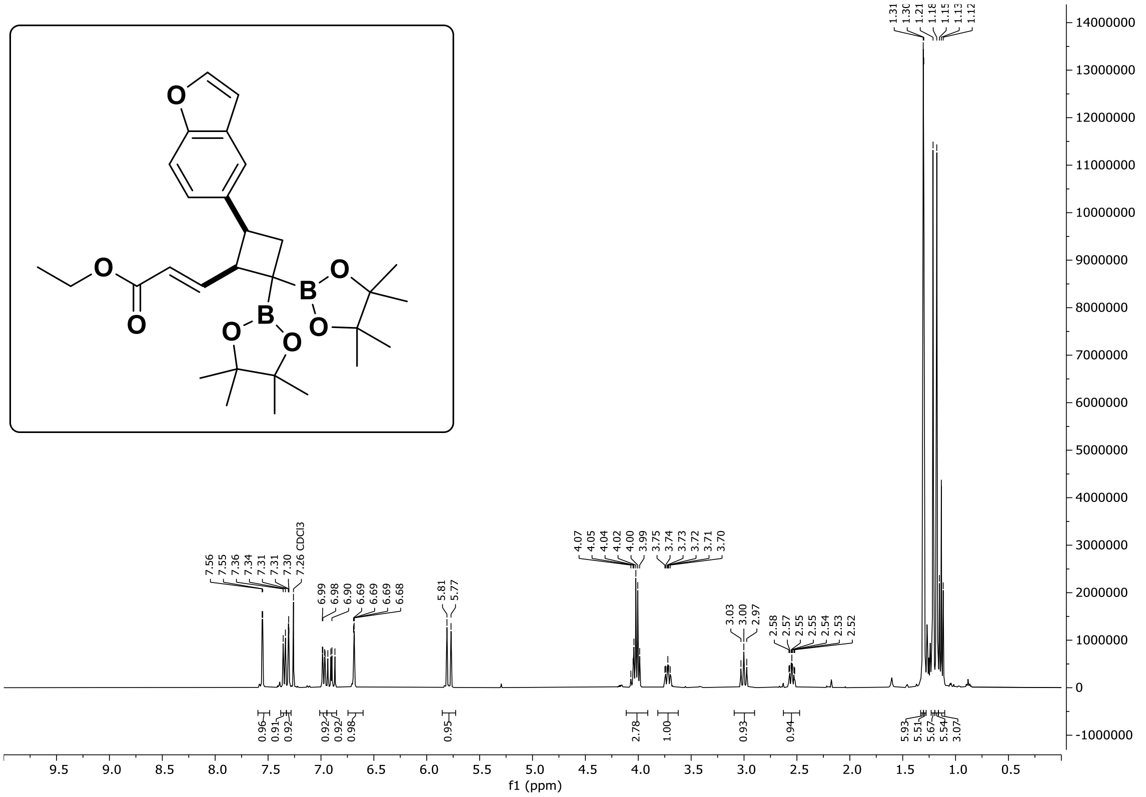

^{13}C-NMR of 13 (100 MHz, $CDCl_3$)

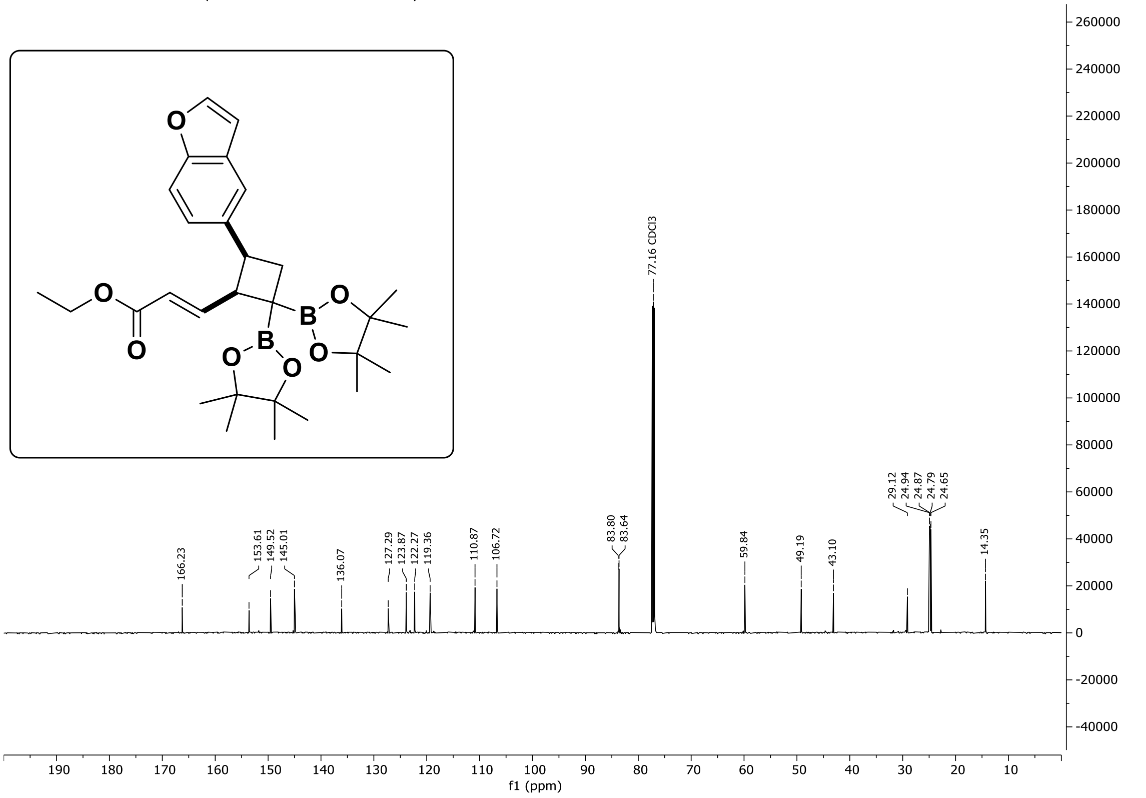

^{11}B NMR of 13 (128 MHz, $CDCl_3$)

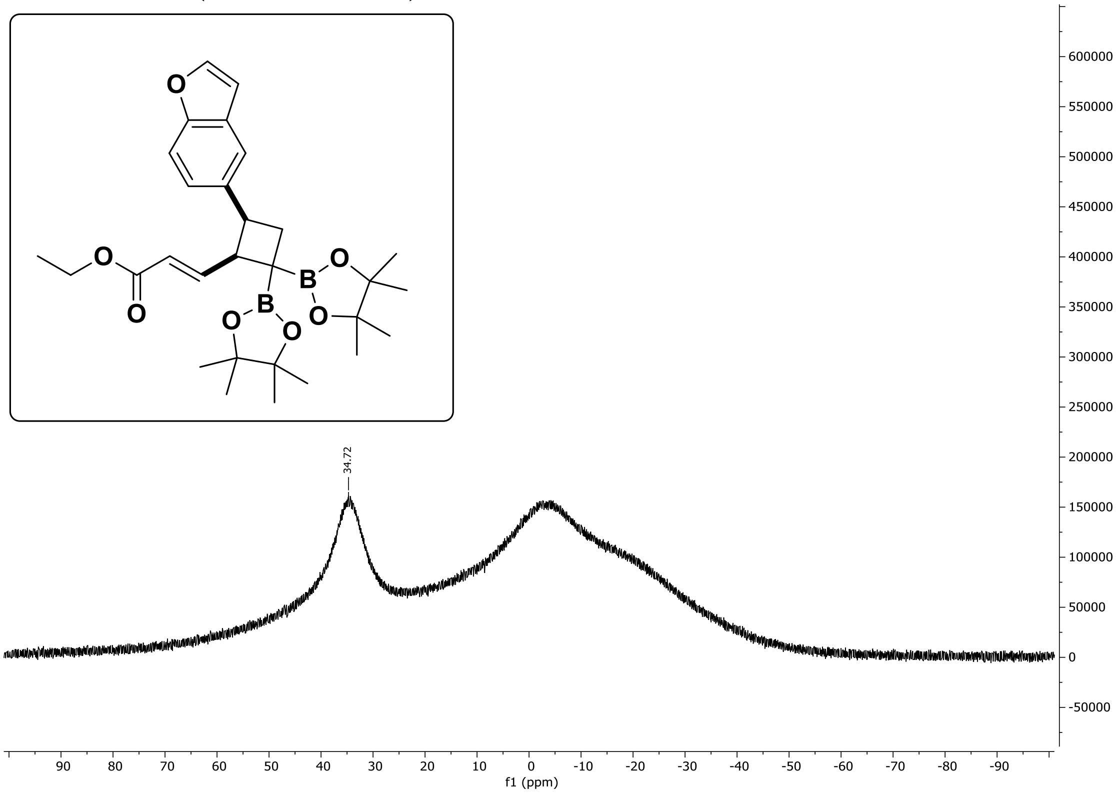

^{1}H-NMR of 14 (400 MHz, $CDCl_3$)

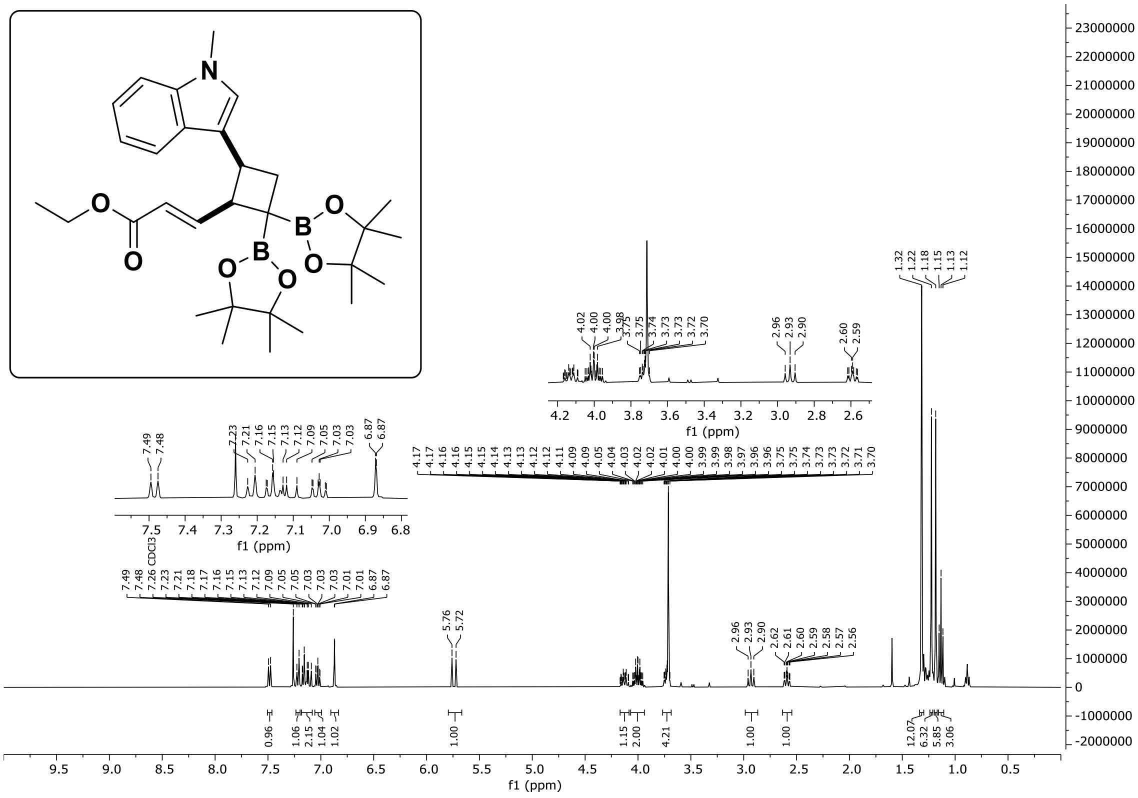

^{13}C-NMR of 14 (100 MHz, $CDCl_3$)

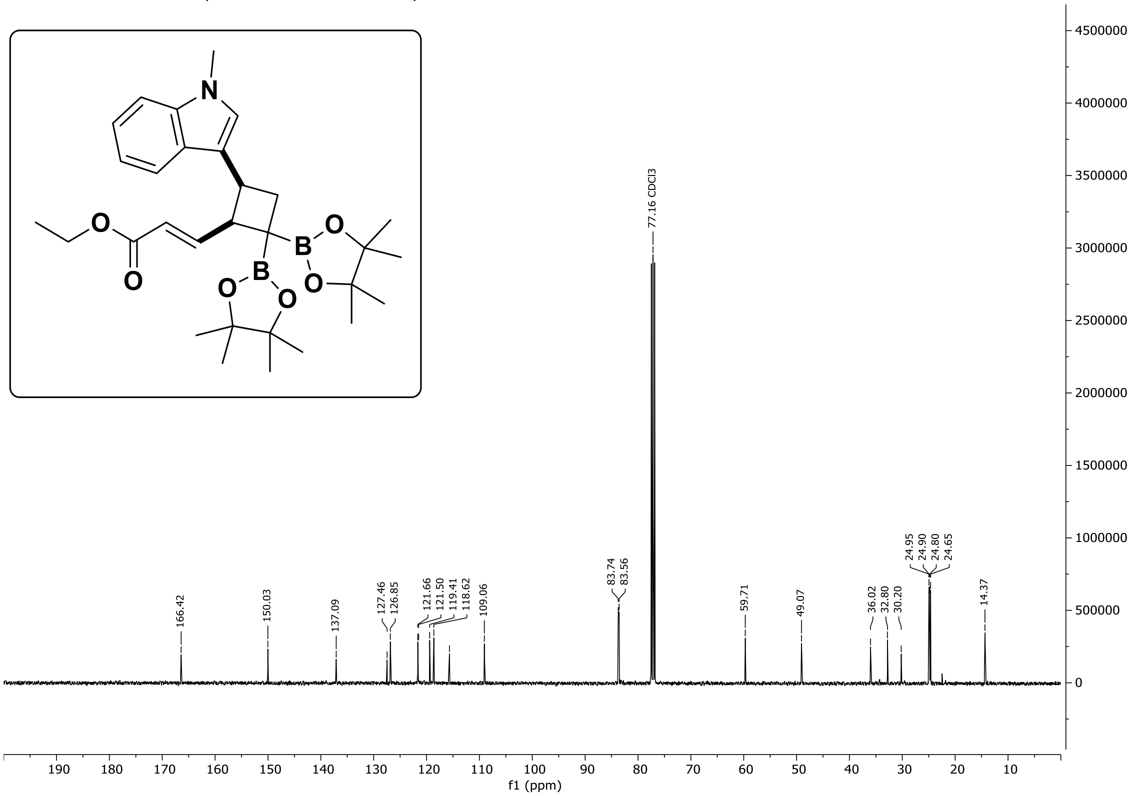

^{11}B NMR of 14 (128 MHz, $CDCl_3$)

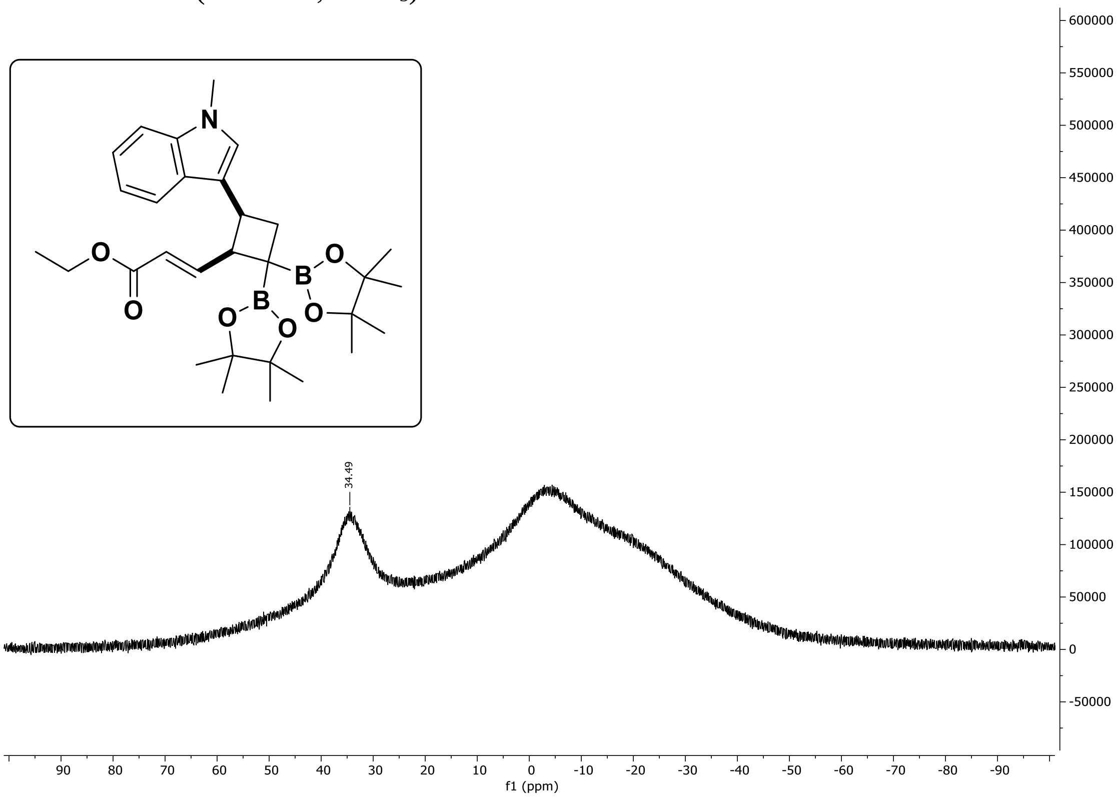

^{1}H-NMR of 15 (400 MHz, $CDCl_3$)

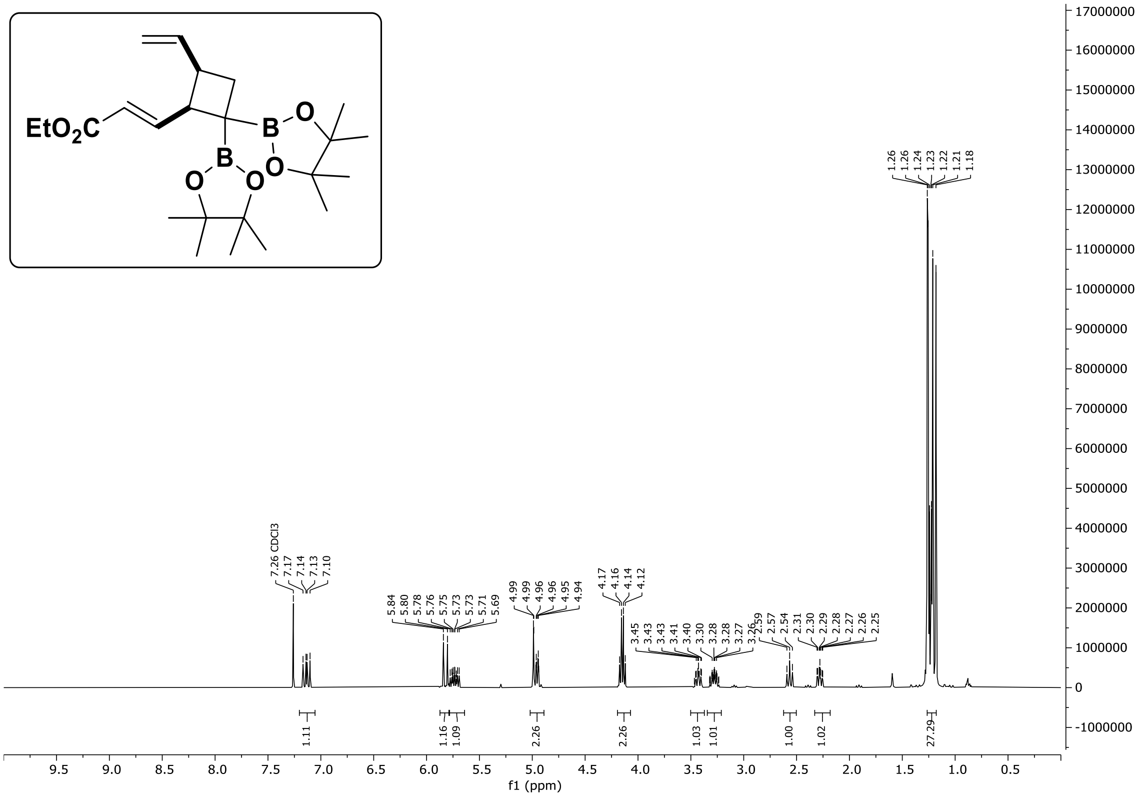

^{13}C-NMR of 15 (100 MHz, $CDCl_3$)

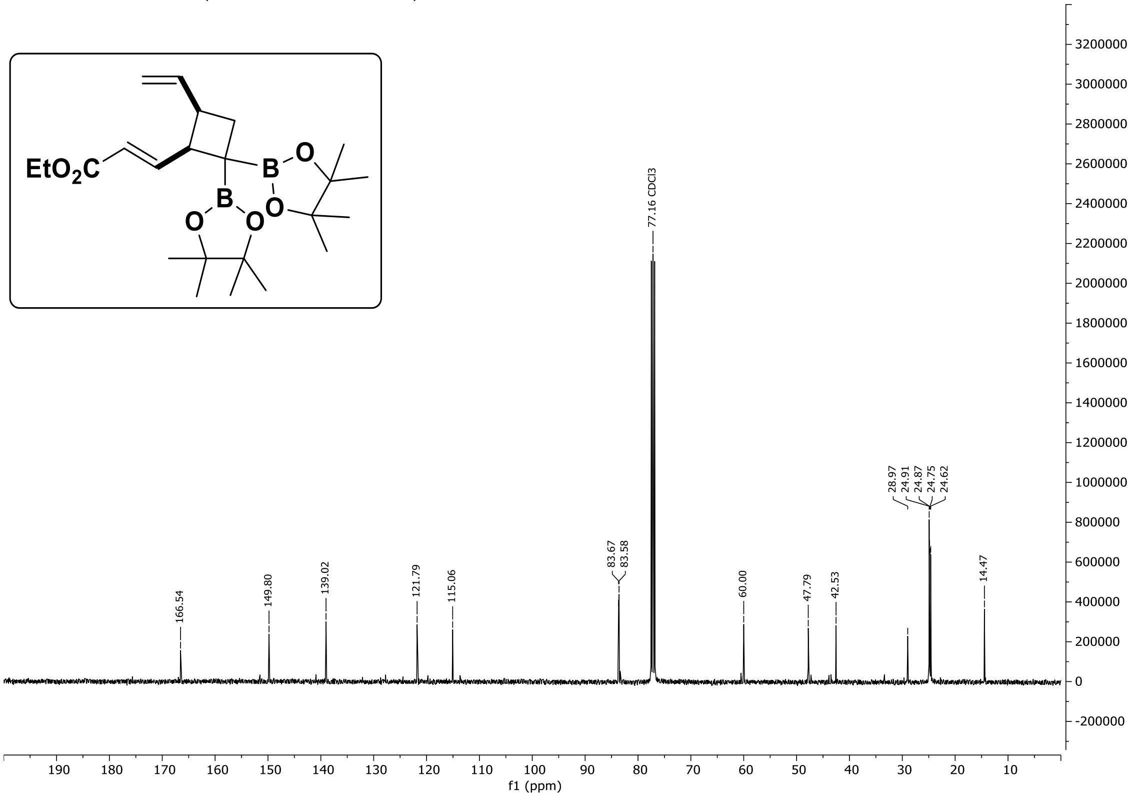

^{11}B NMR of 15 (128 MHz, $CDCl_3$)

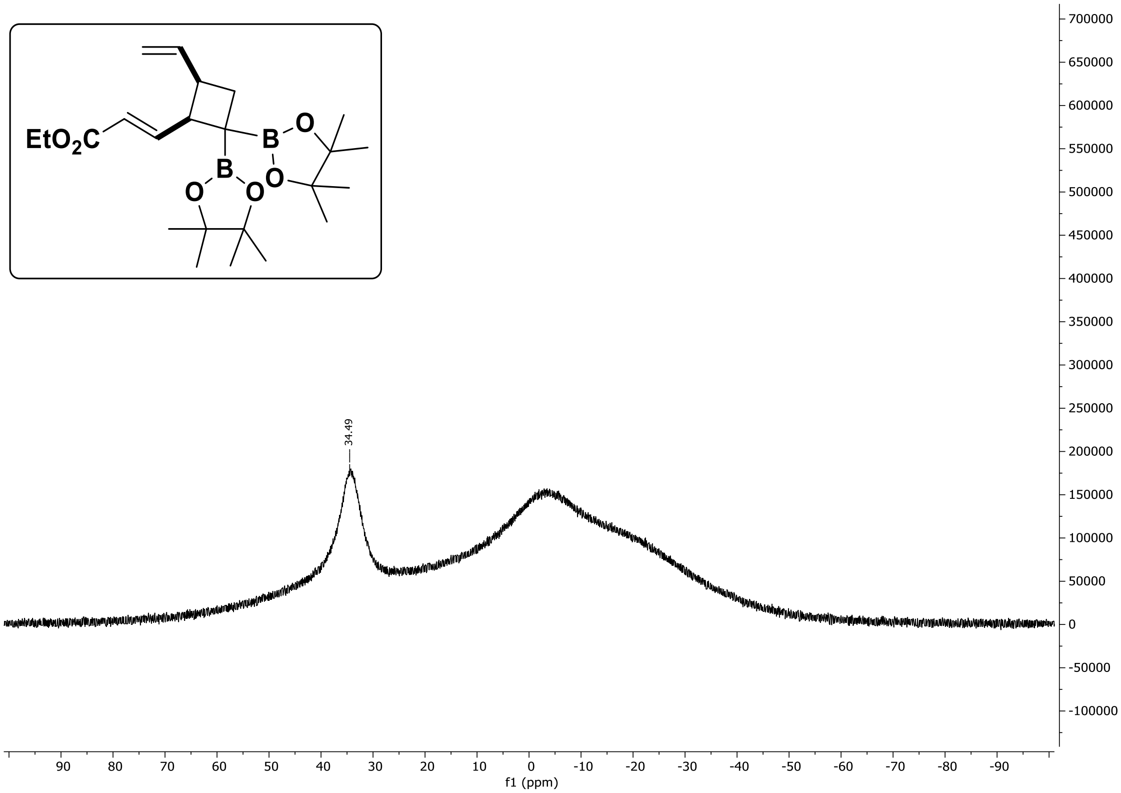

^{1}H-NMR of 16 (400 MHz, $CDCl_3$)

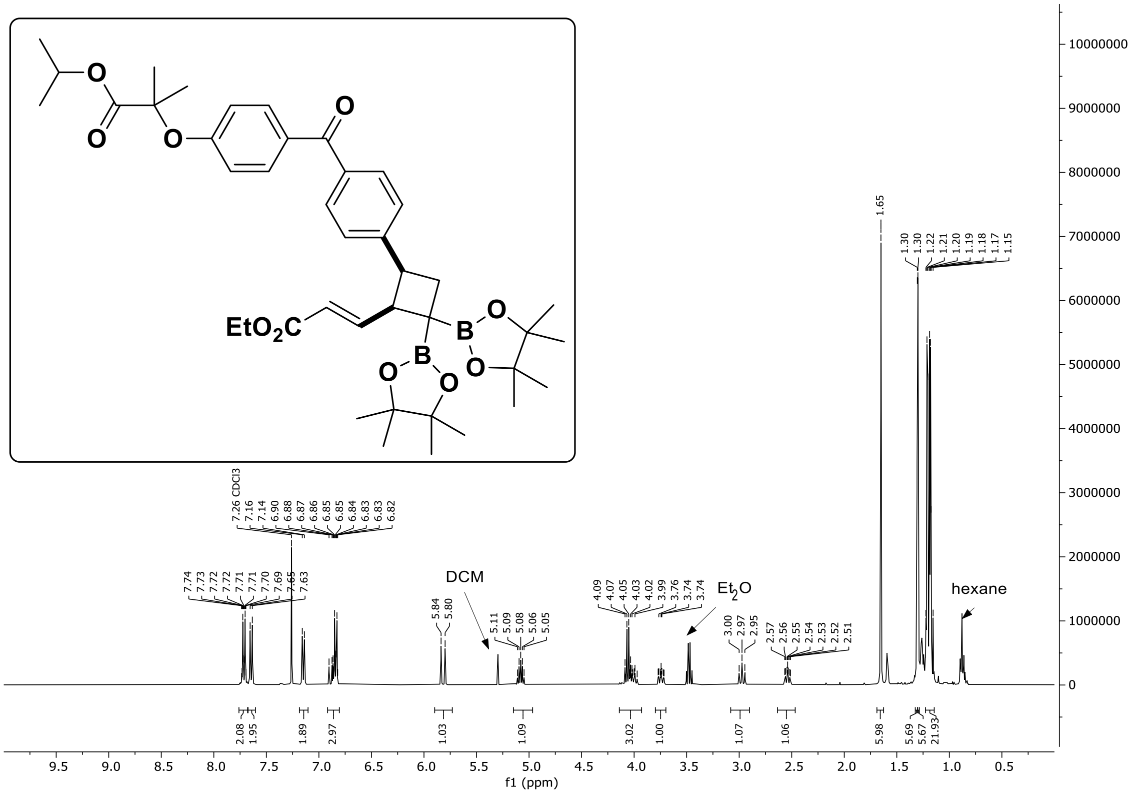

^{13}C-NMR of 16 (100 MHz, $CDCl_3$)

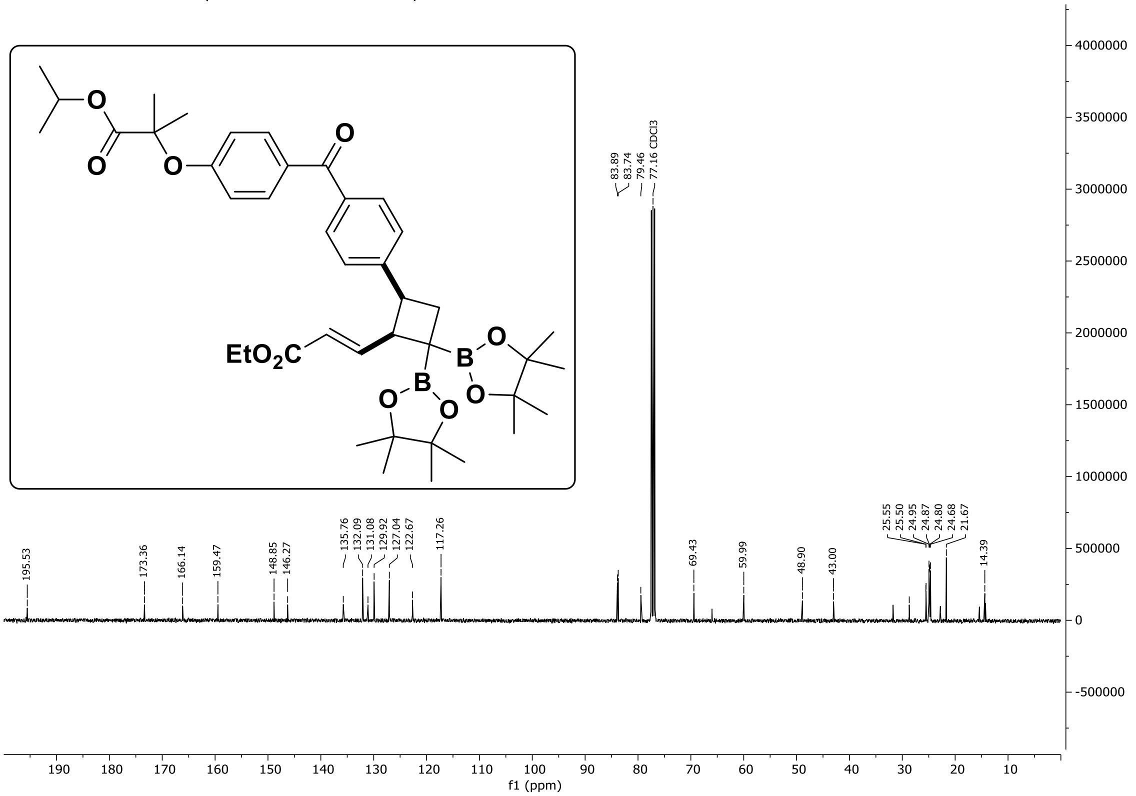

^{11}B NMR of 16 (128 MHz, $CDCl_3$)

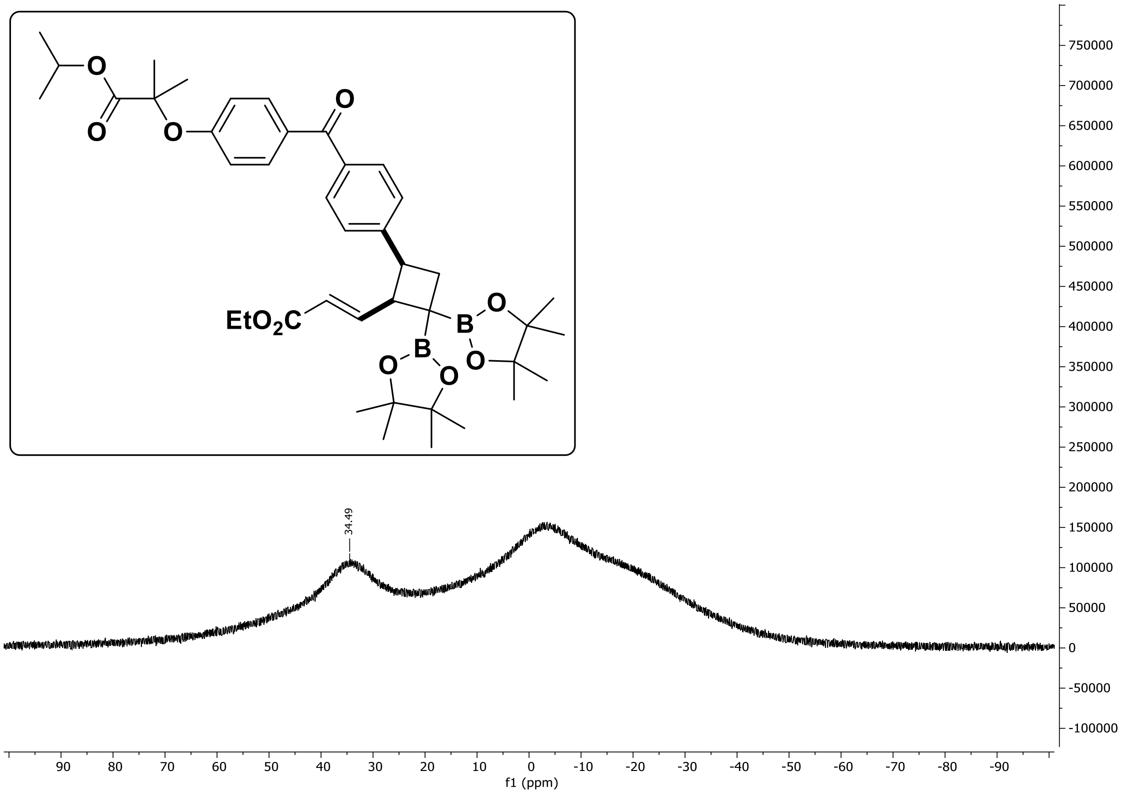

^{1}H-NMR of 17 (400 MHz, $CDCl_3$)

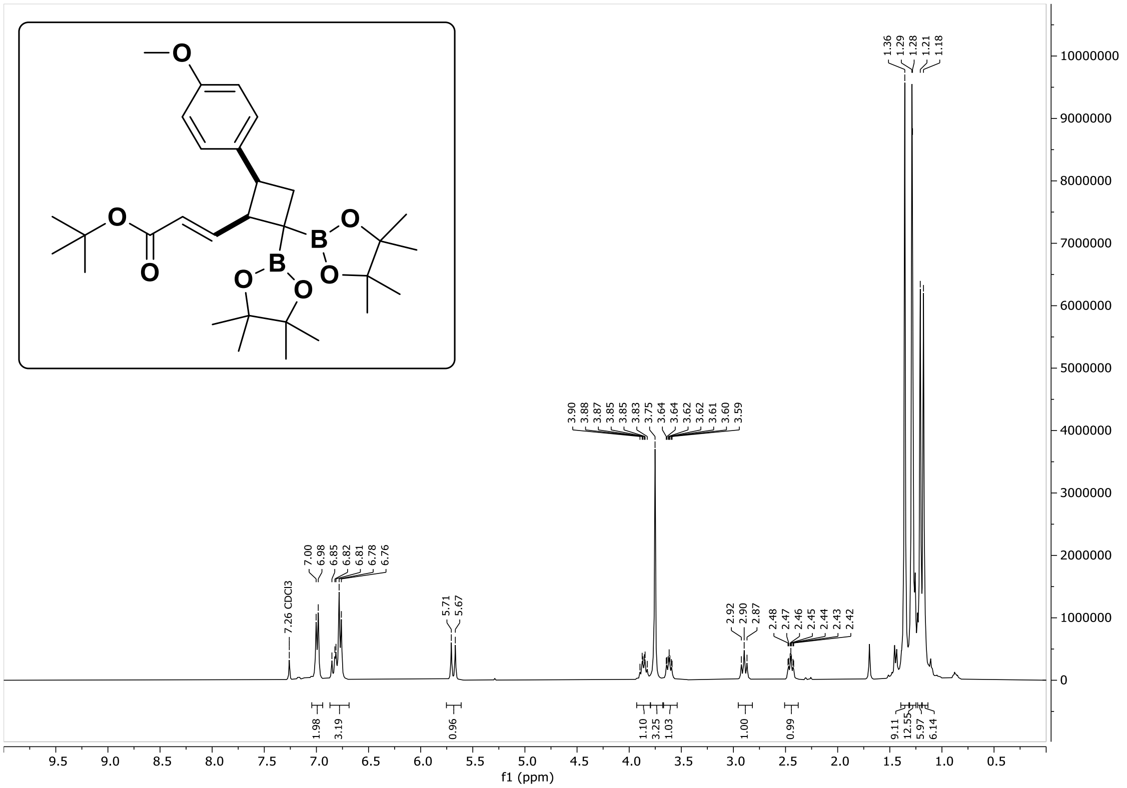

^{13}C-NMR of 17 (100 MHz, $CDCl_3$)

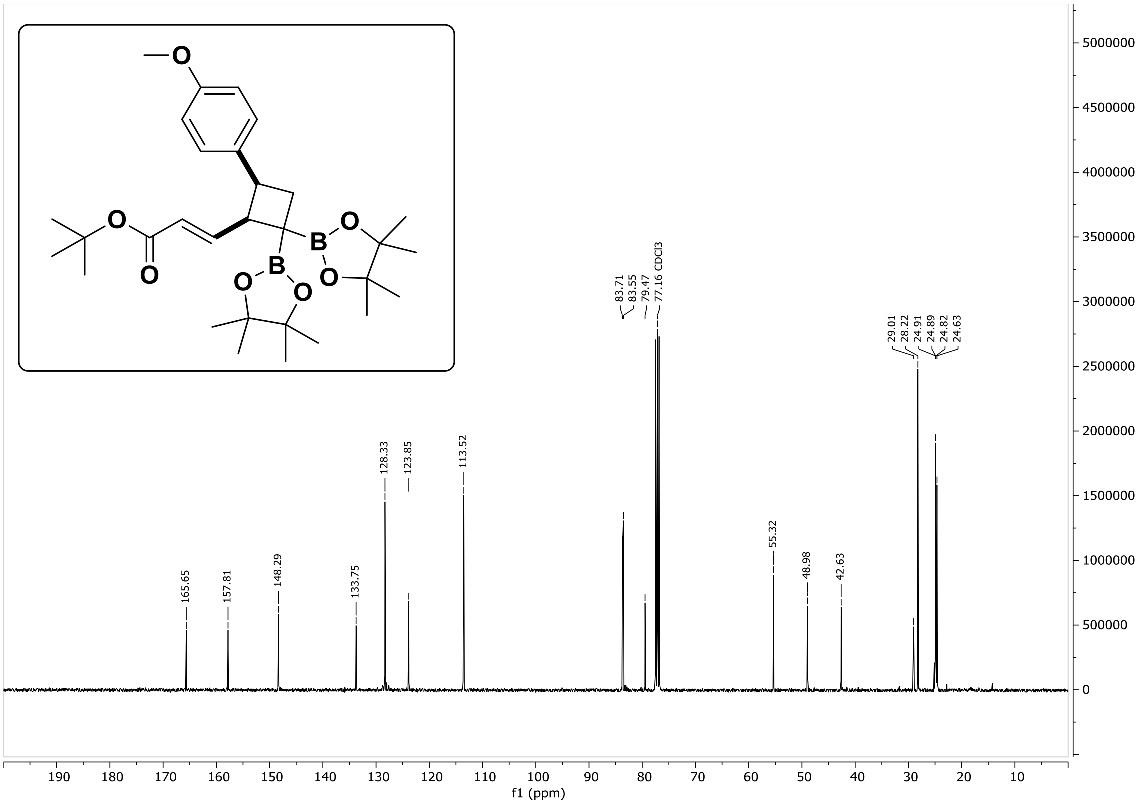

^{11}B NMR of 17 (128 MHz, $CDCl_3$)

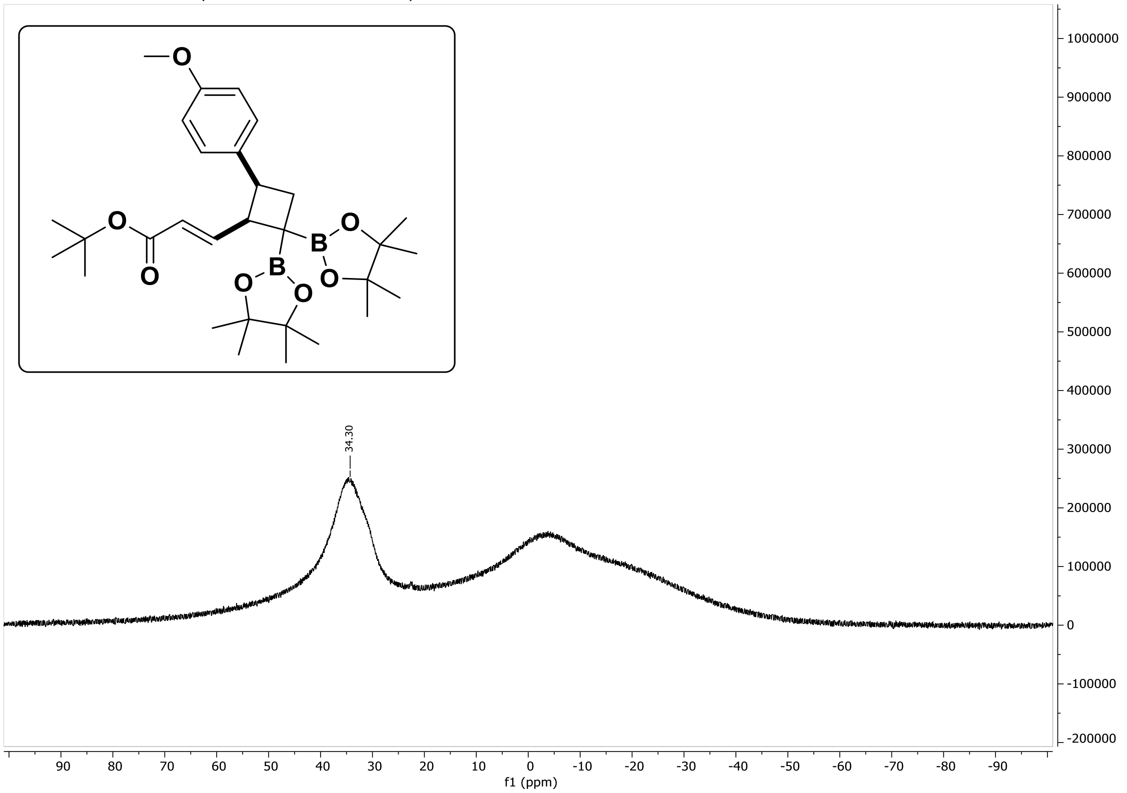

^{1}H-NMR of 18-major (400 MHz, $CDCl_3$)

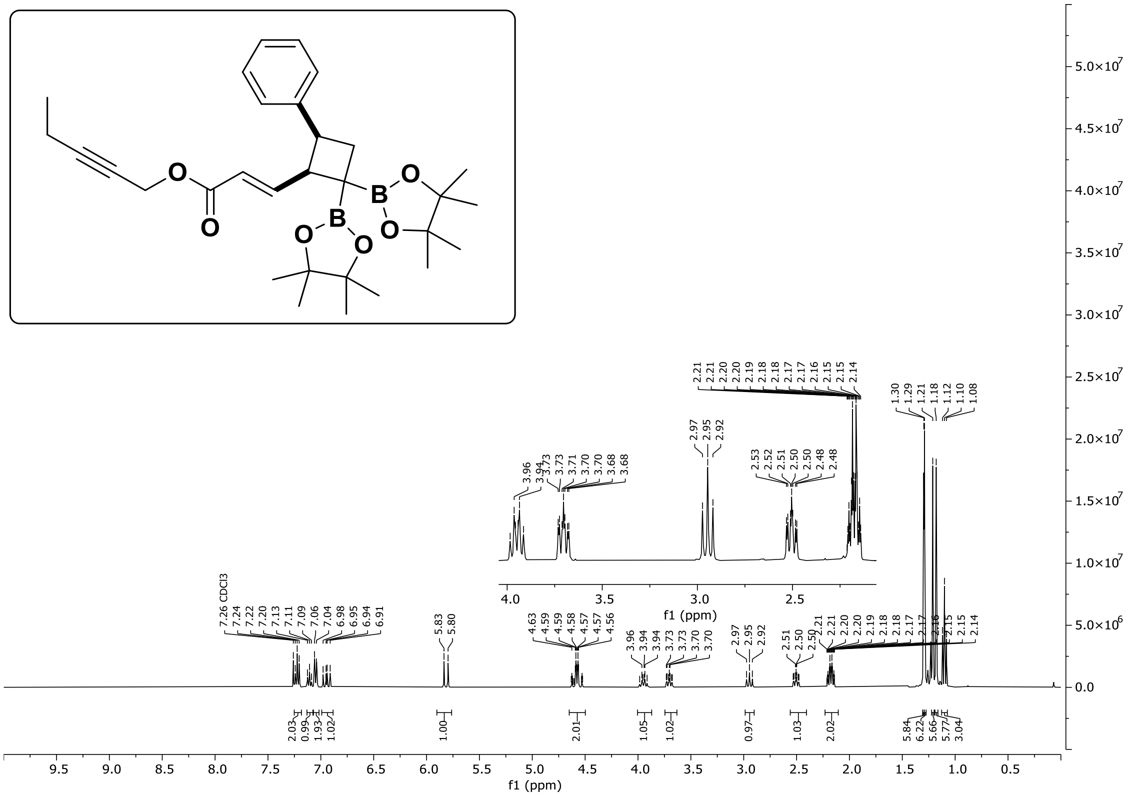

^{13}C-NMR of 18-major (100 MHz, $CDCl_3$)

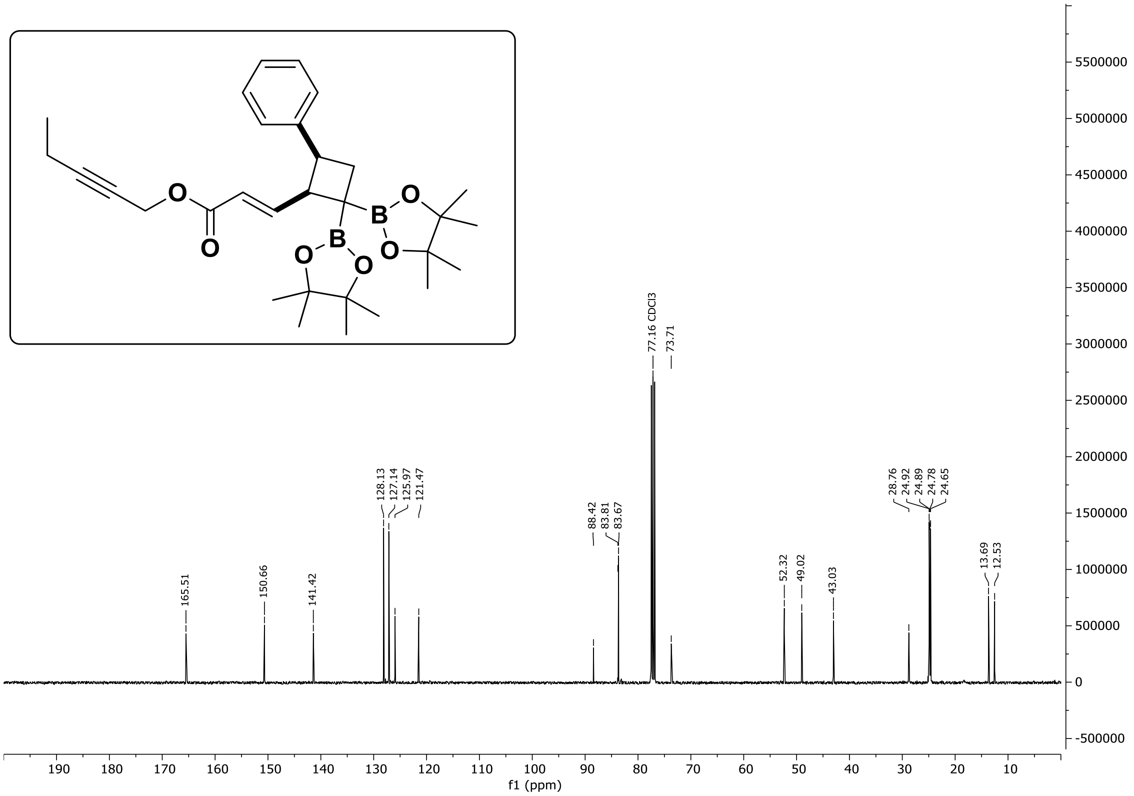

^{11}B NMR of 18-major (192 MHz, $CDCl_3$)

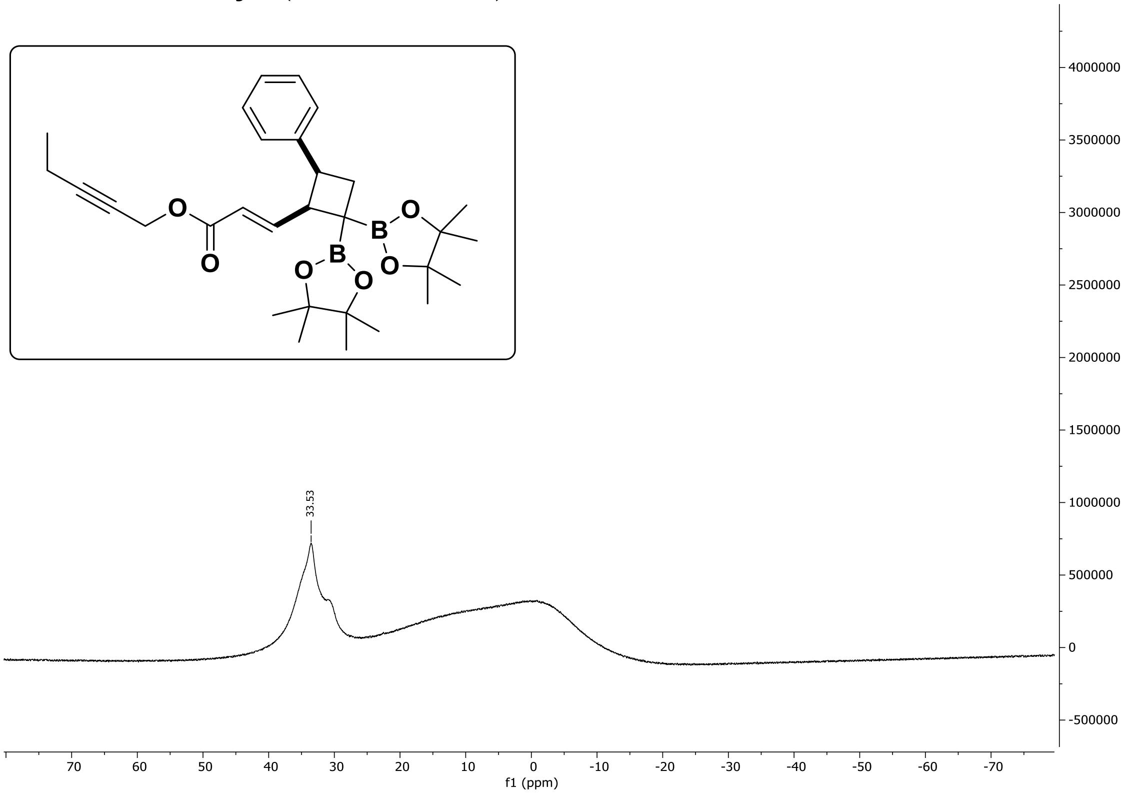

NOESY of 18-major:

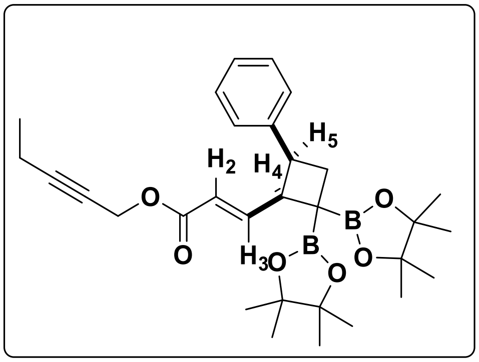

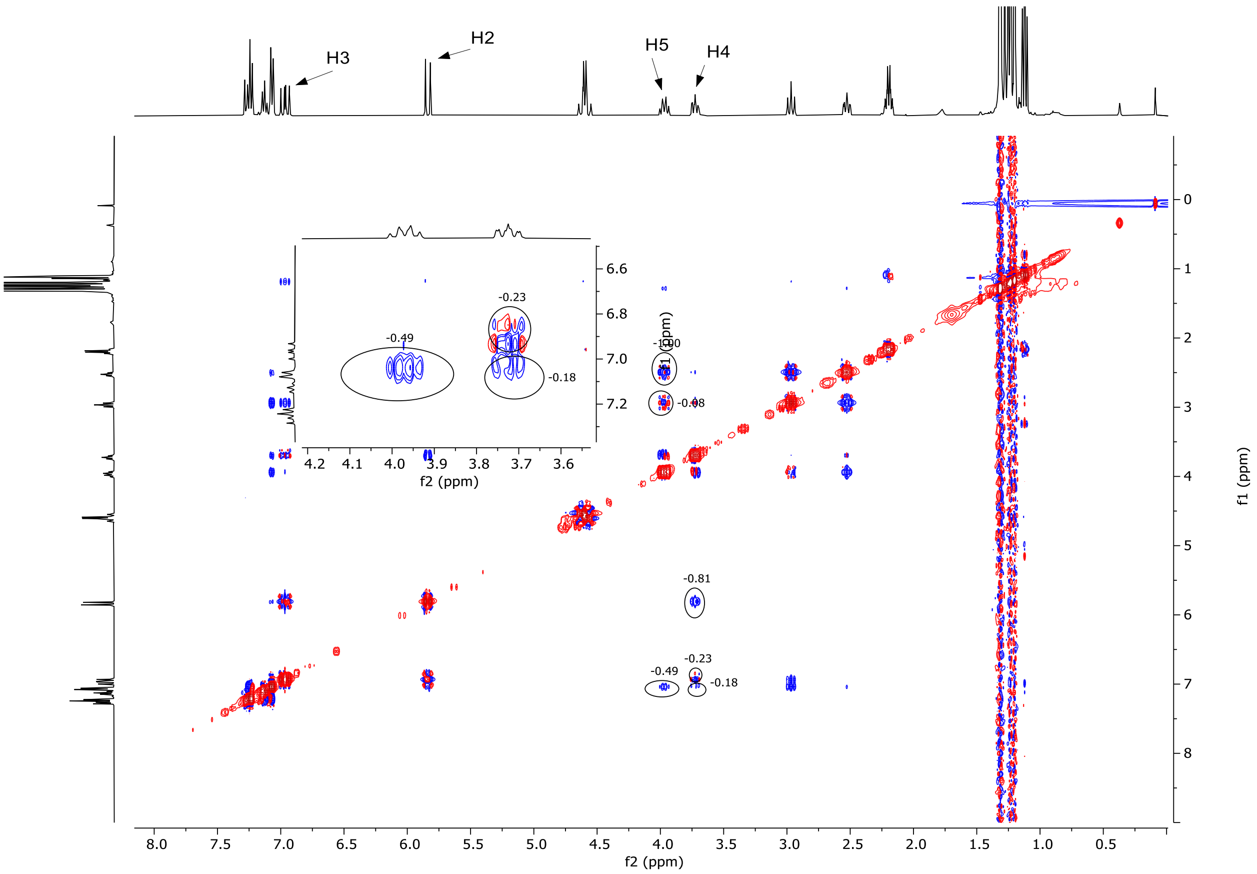

H4 has good nOe with H2 and H3, but H5 has no nOe with H2 and H3.

^{1}H-NMR of 18-minor (400 MHz, $CDCl_3$)

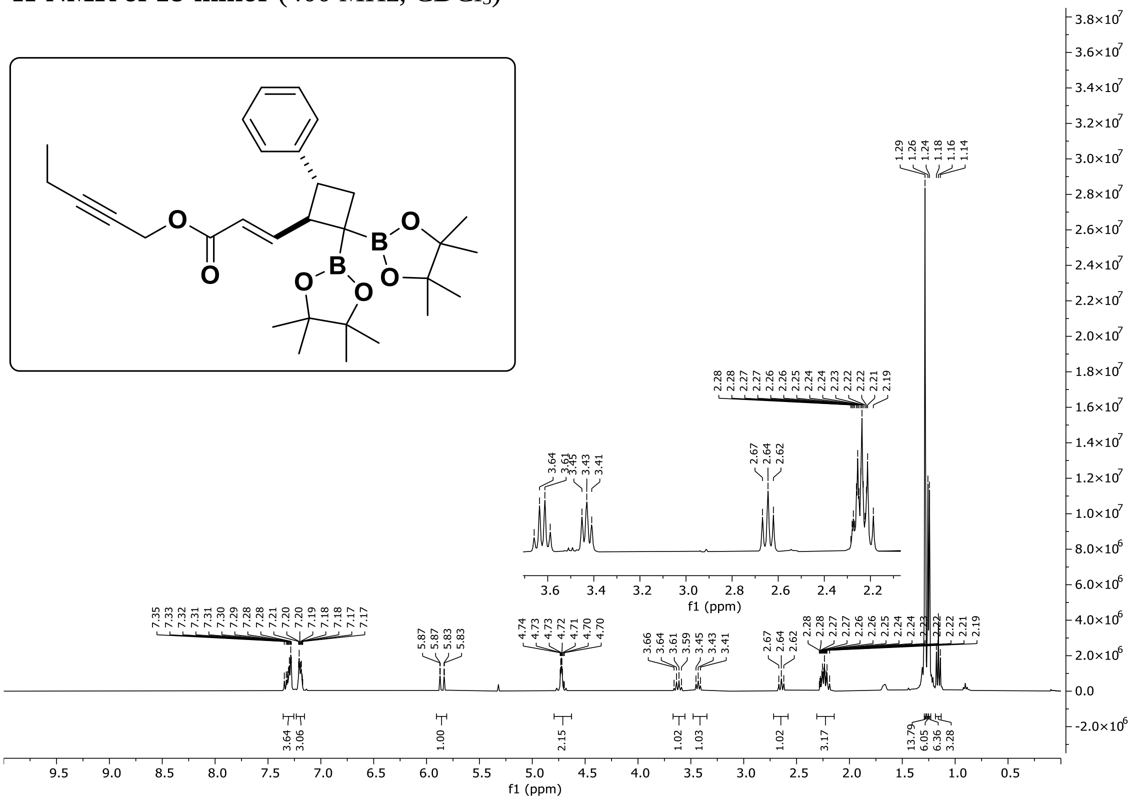

^{13}C-NMR of 18-minor (100 MHz, $CDCl_3$)

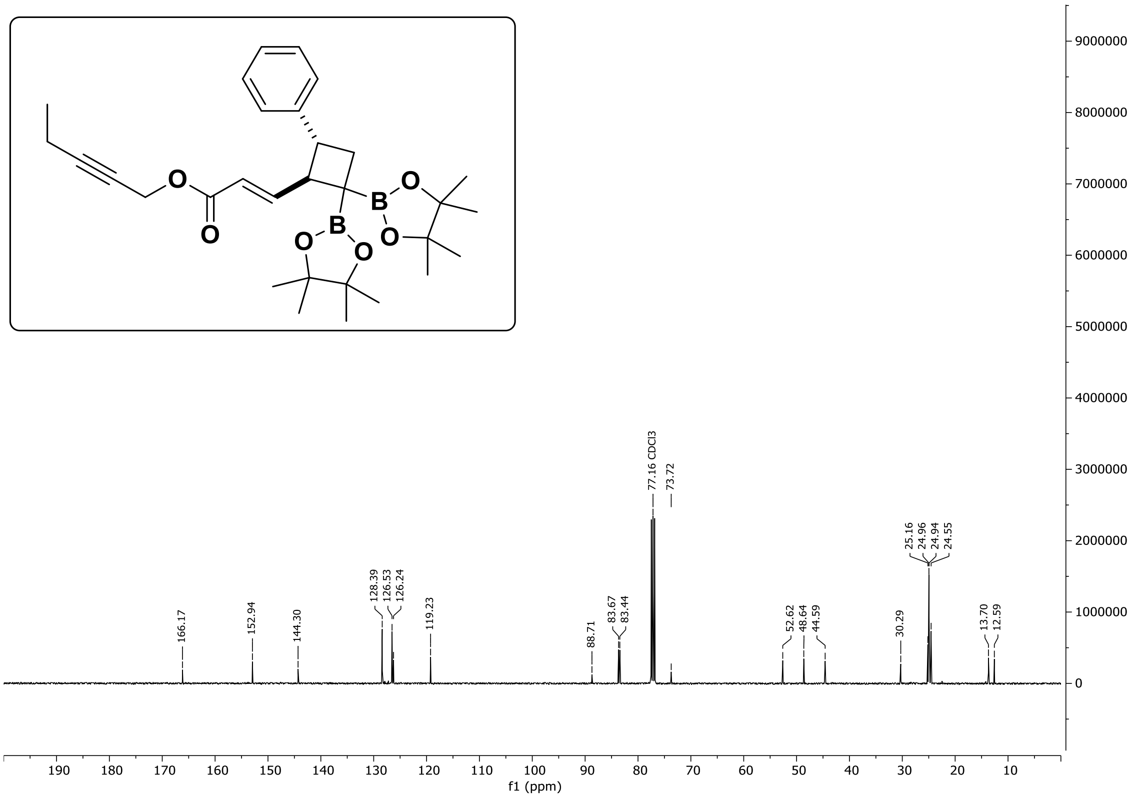

^{11}B NMR of 18-minor (128 MHz, $CDCl_3$)

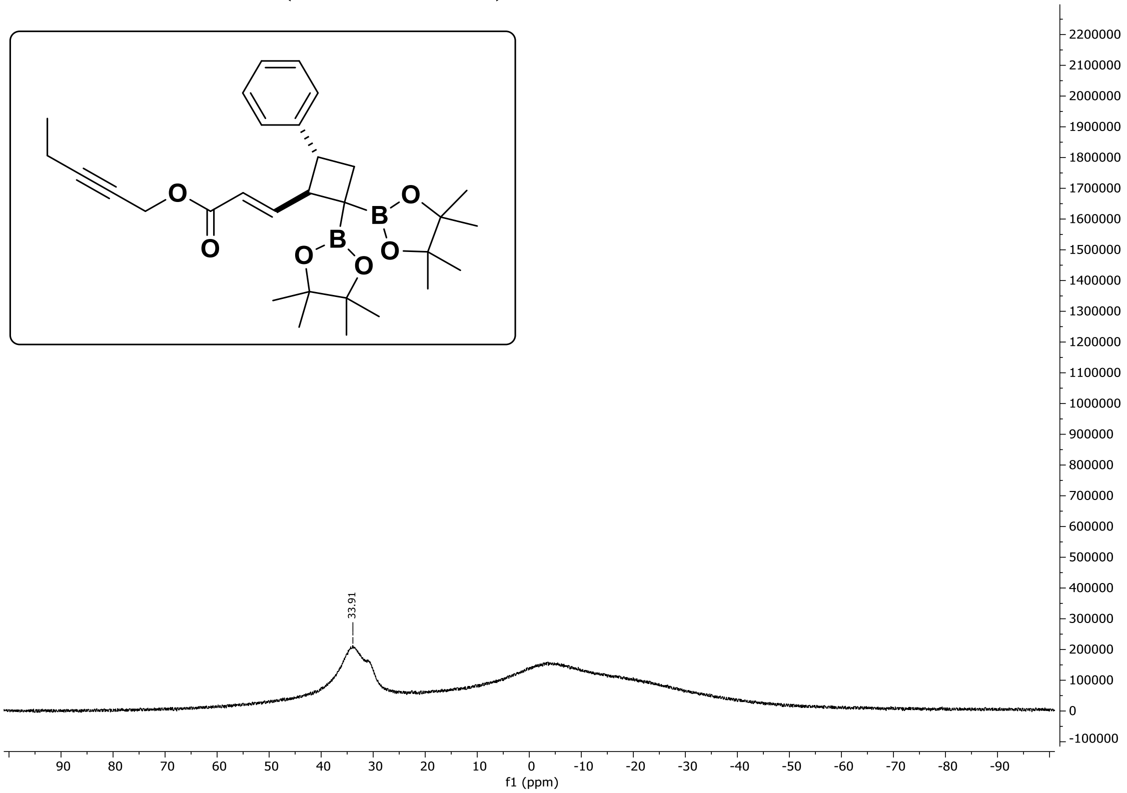

NOESY of 18-minor:

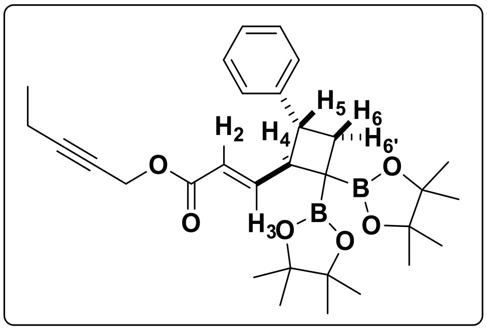

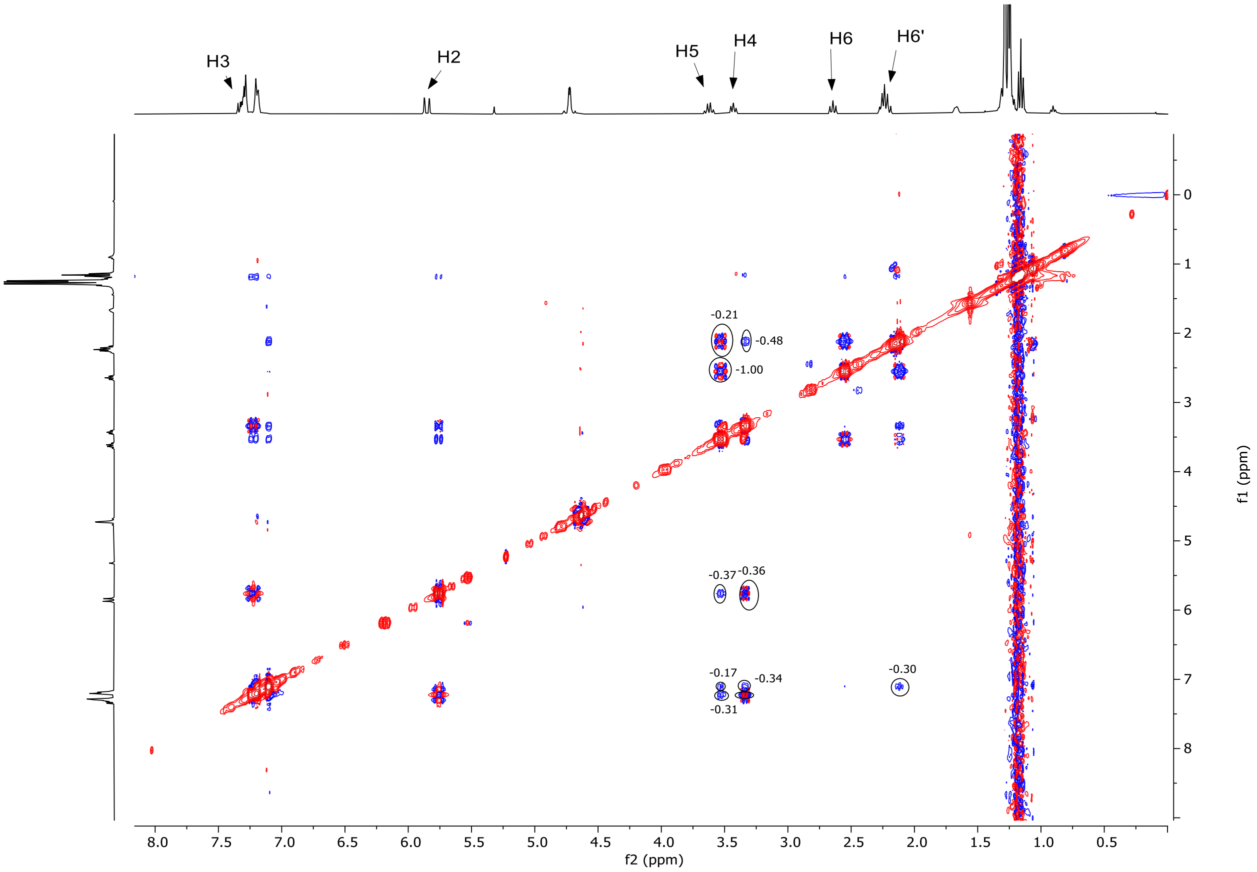

Both H4 and H5 have good nOe with H2 and H3.

H5 has good nOe with H6, but weak nOe with H6'; H4 has good nOe with H6'.

^{1}H-NMR of 19 (400 MHz, $CDCl_3$)

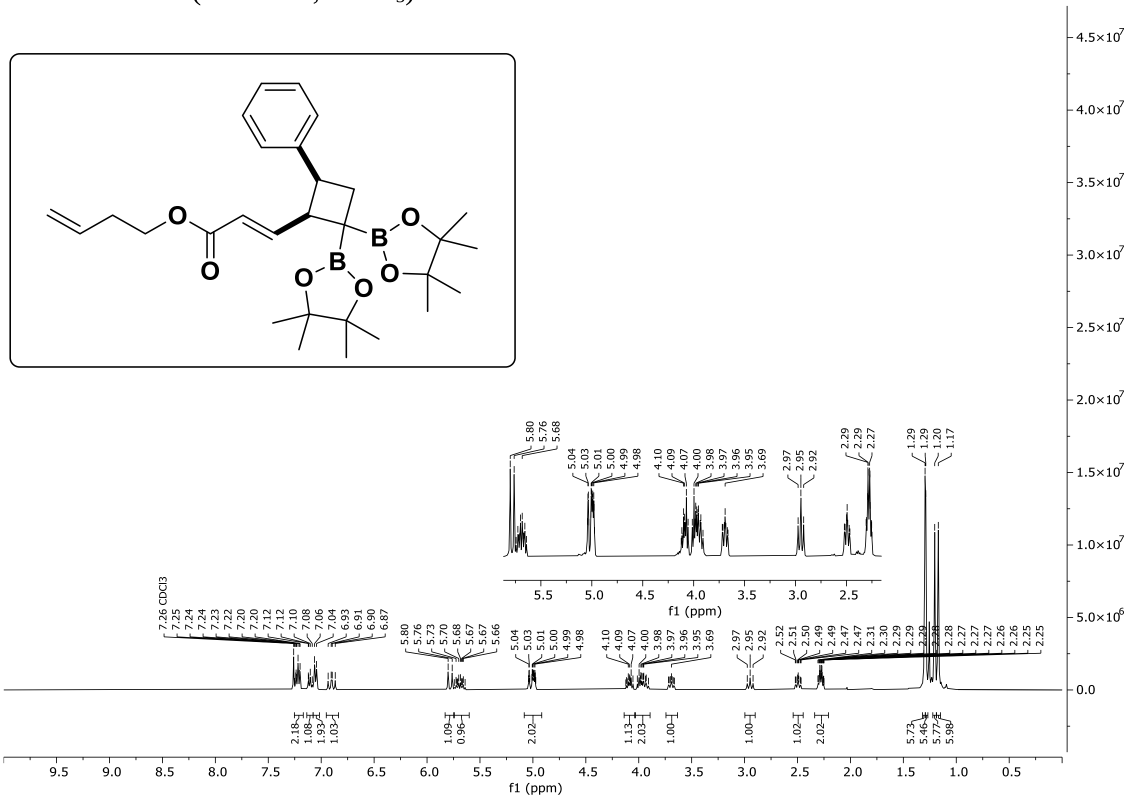

^{13}C-NMR of 19 (100 MHz, $CDCl_3$)

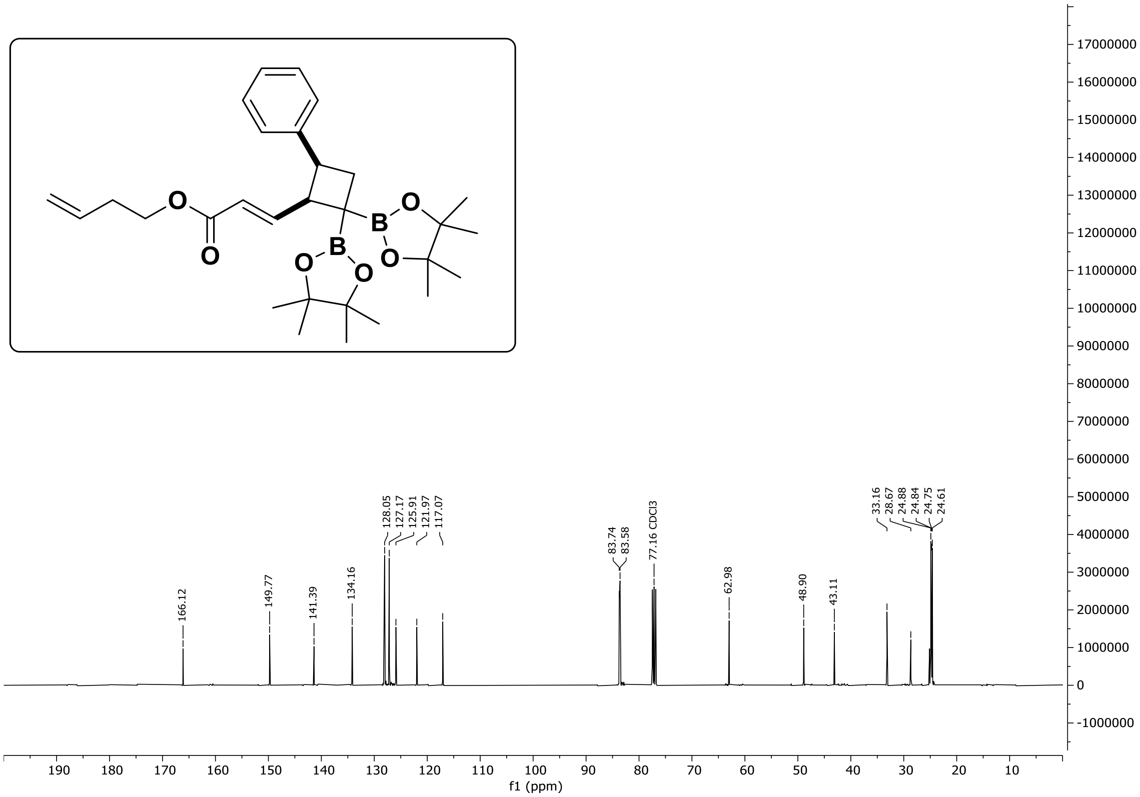

^{11}B NMR of 19 (128 MHz, $CDCl_3$)

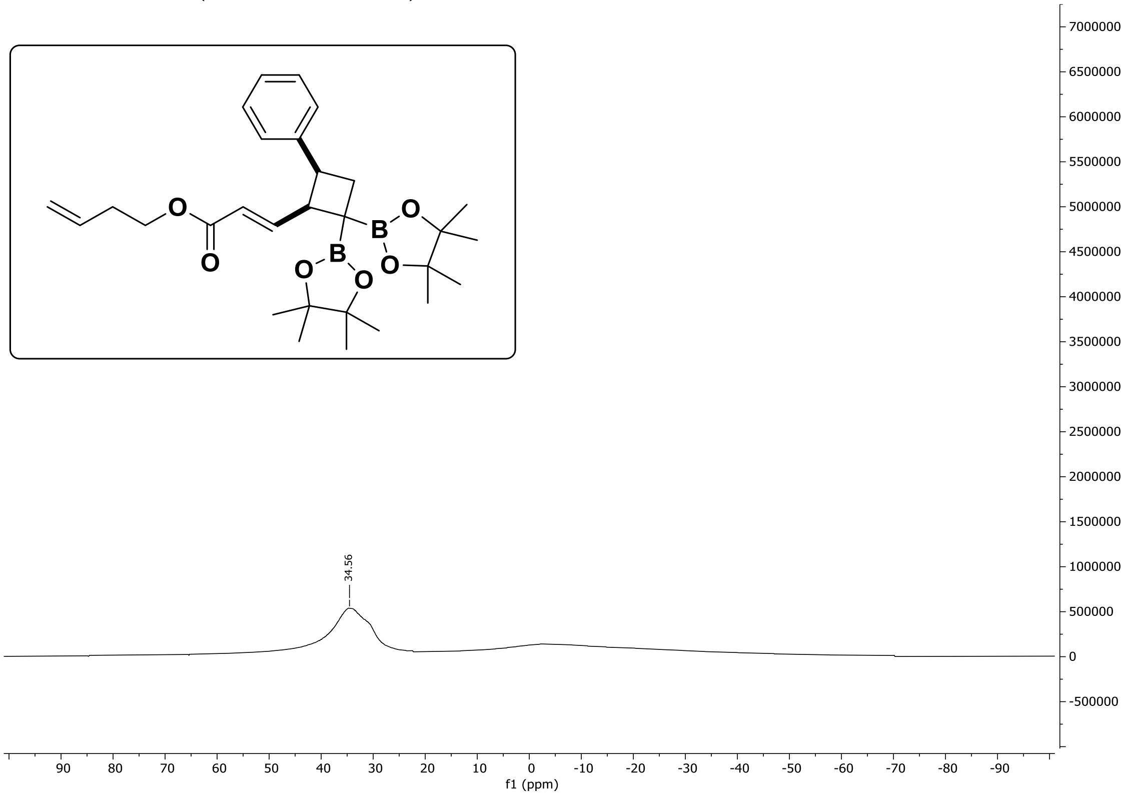

^{1}H-NMR of 20 (400 MHz, $CDCl_3$)

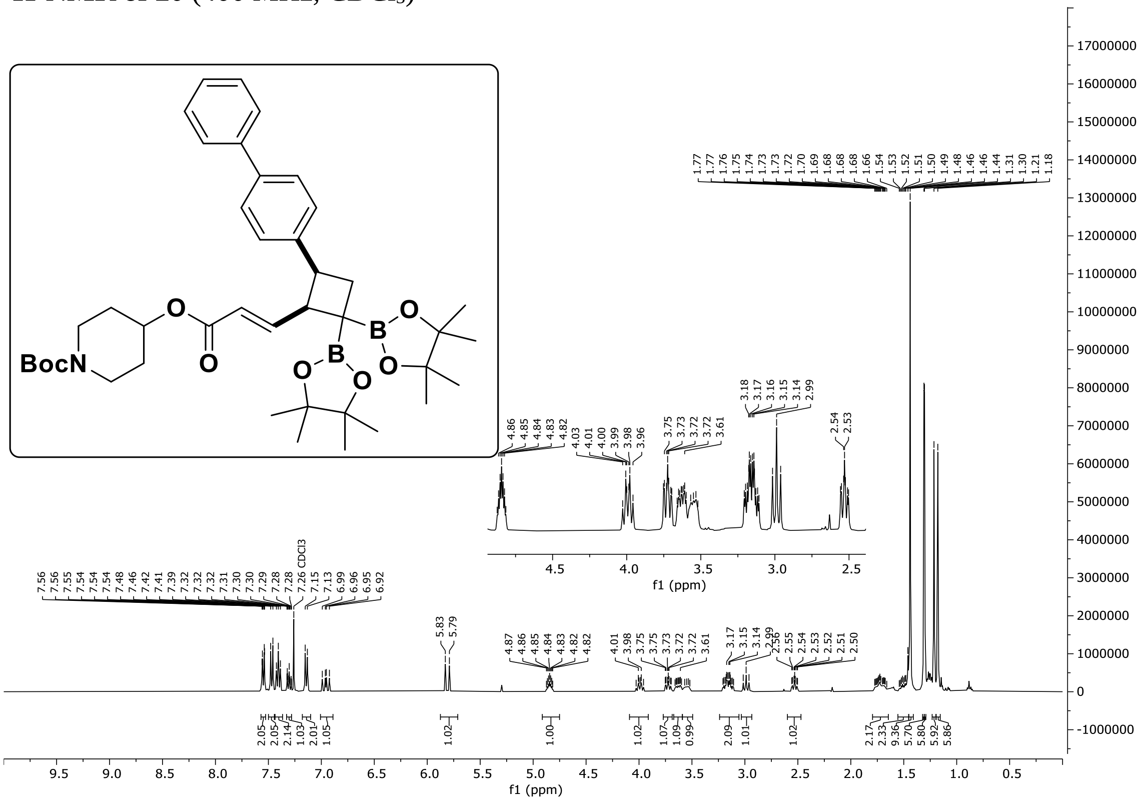

^{13}C-NMR of 20 (100 MHz, $CDCl_3$)

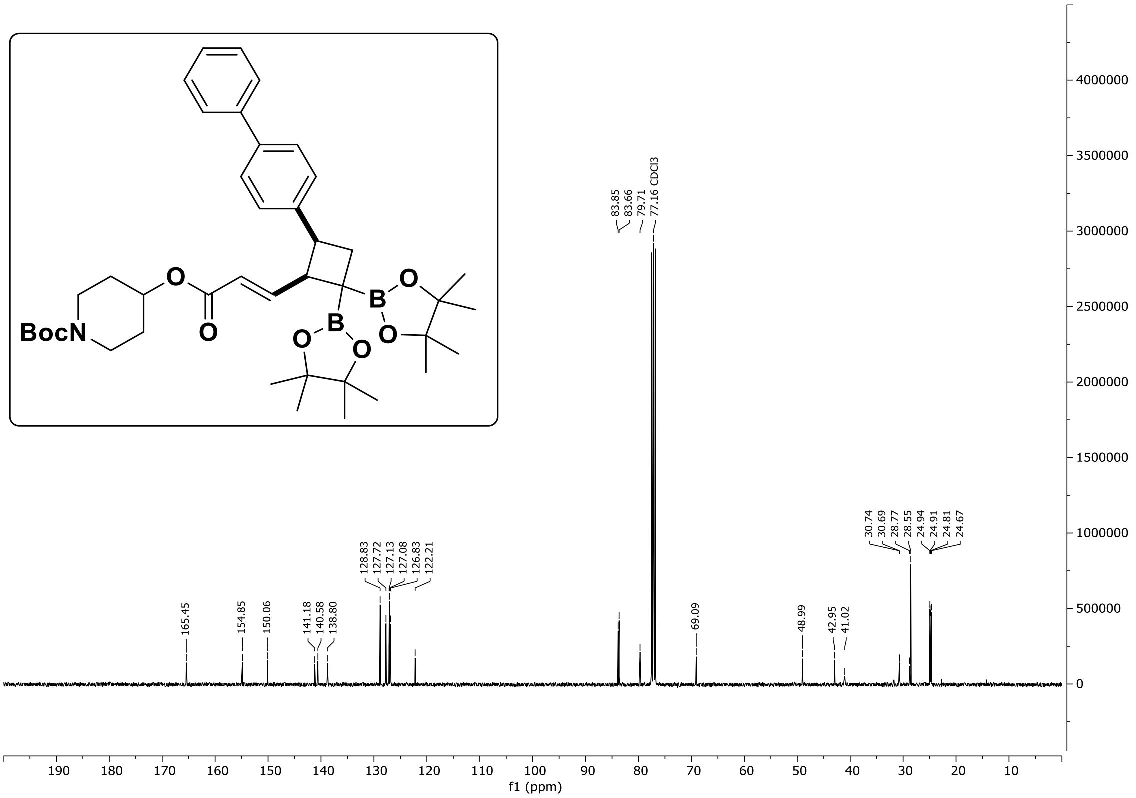

^{11}B NMR of 20 (128 MHz, $CDCl_3$)

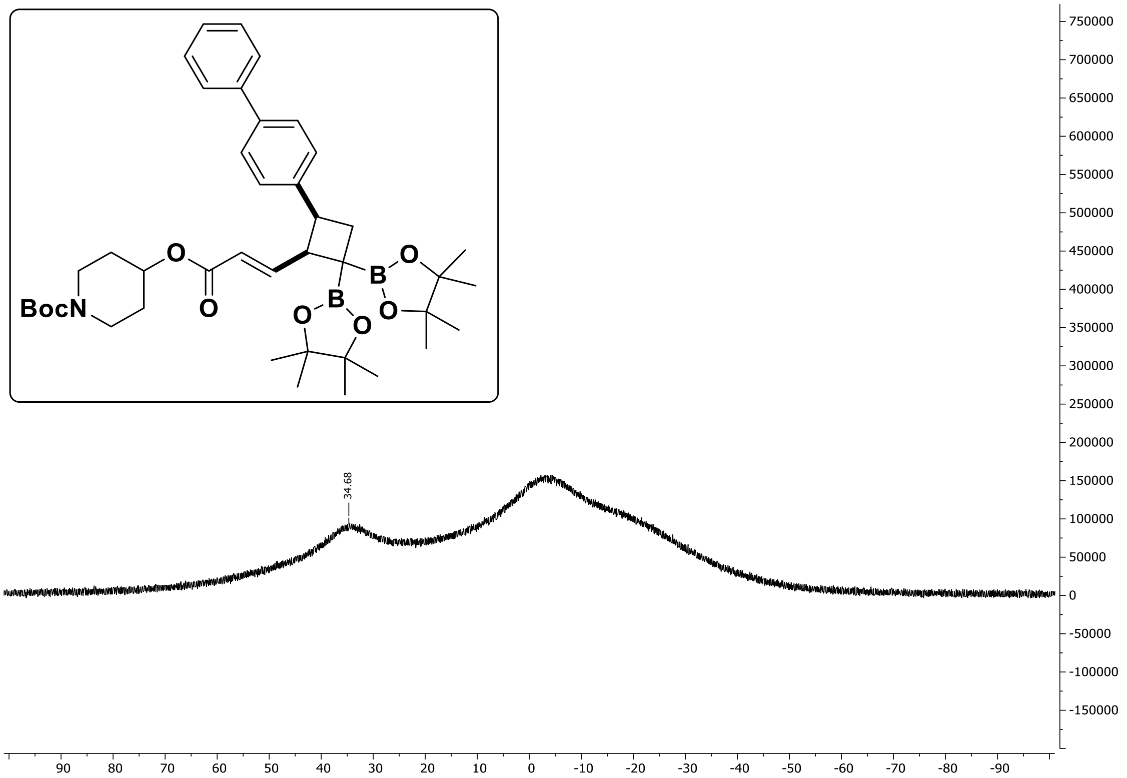

^{1}H-NMR of 21 (600 MHz, $CDCl_3$)

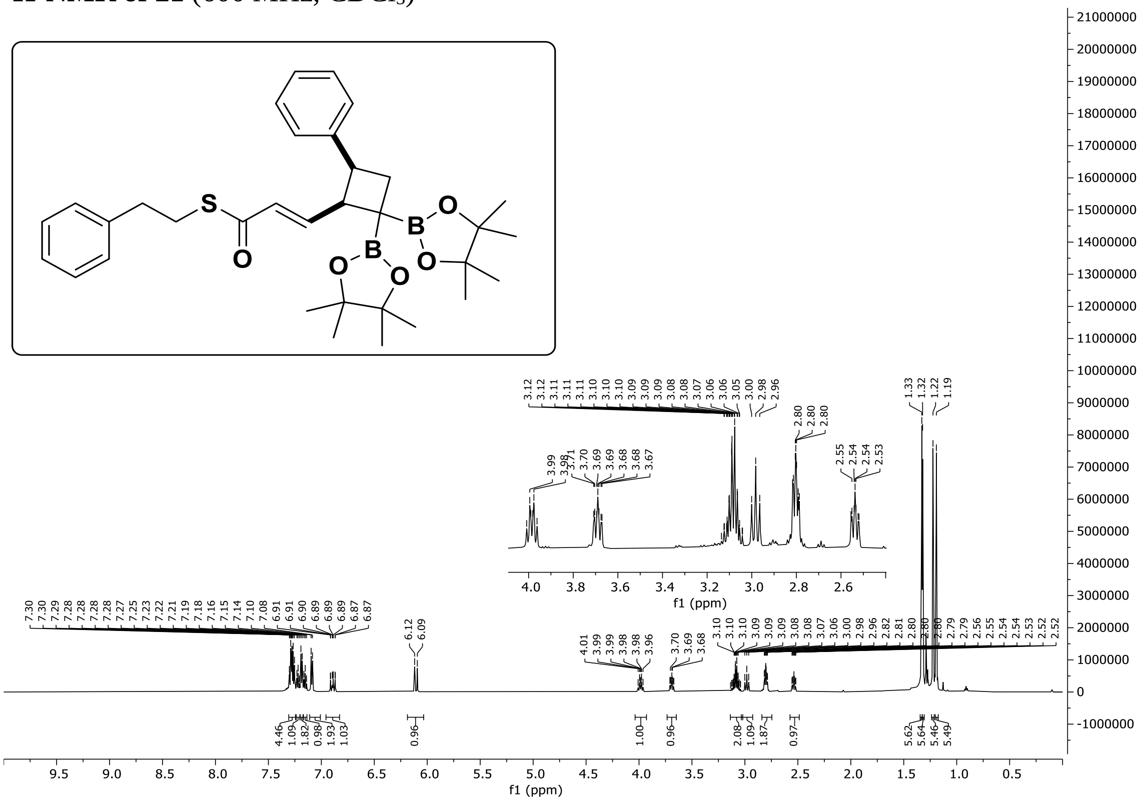

^{13}C-NMR of 21 (151 MHz, $CDCl_3$)

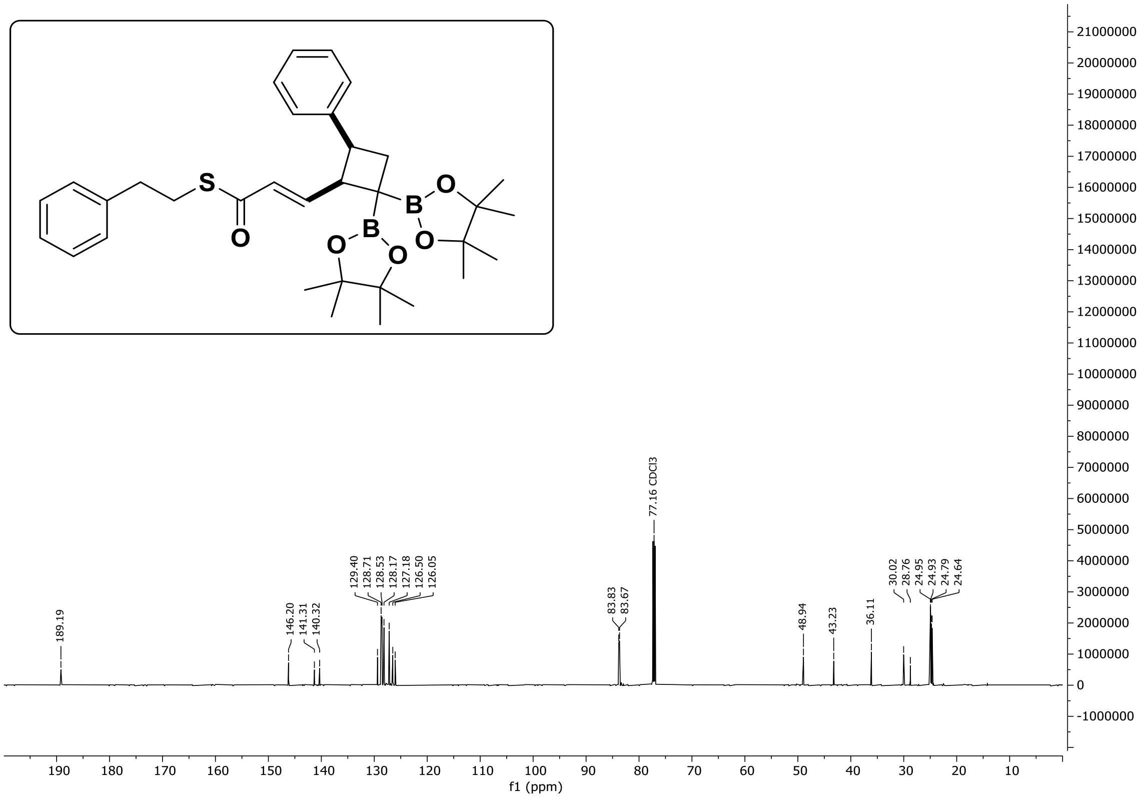

^{11}B NMR of 21 (192 MHz, $CDCl_3$)

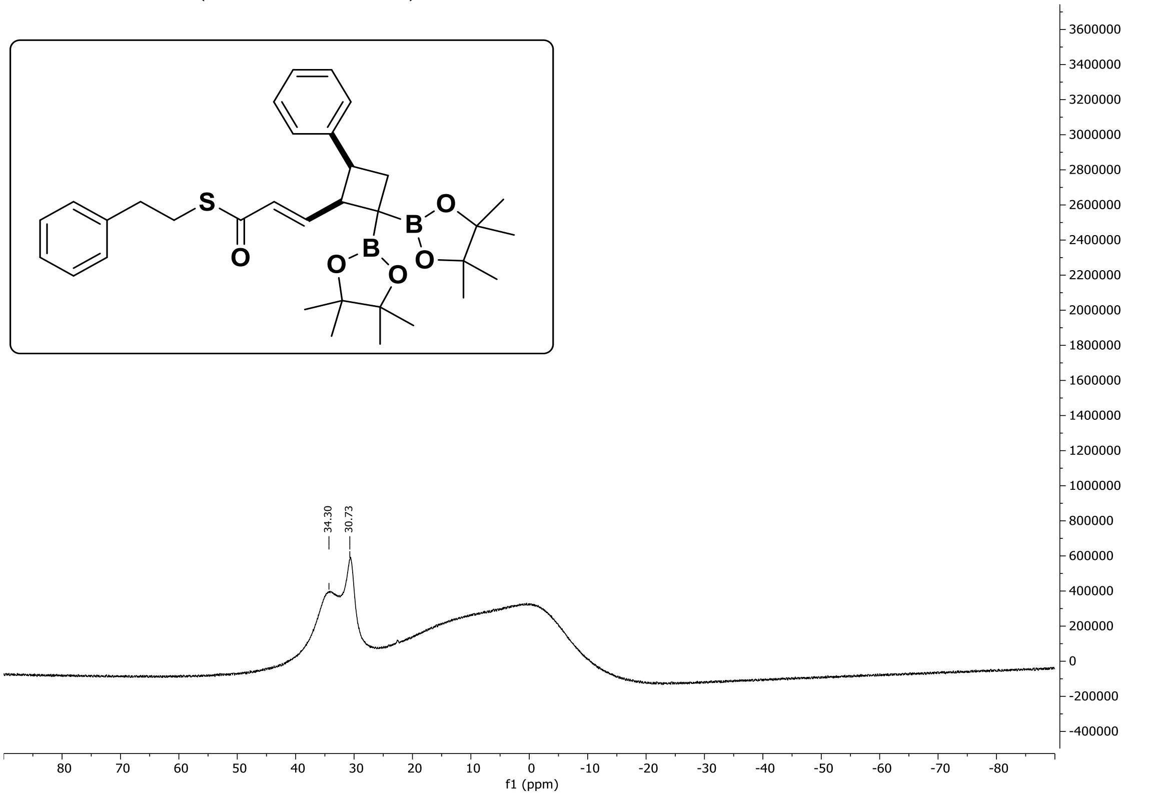

^{1}H-NMR of 22 (400 MHz, $CDCl_3$)

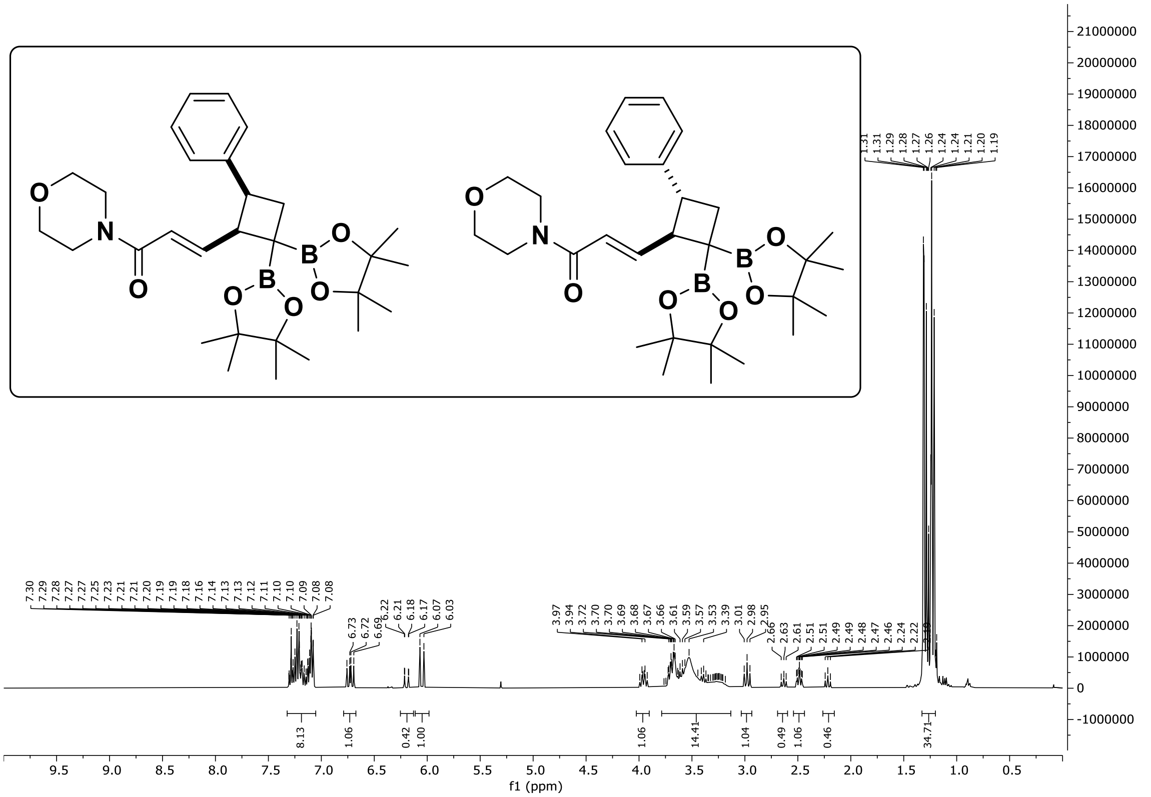

^{13}C-NMR of 22 (100 MHz, $CDCl_3$)

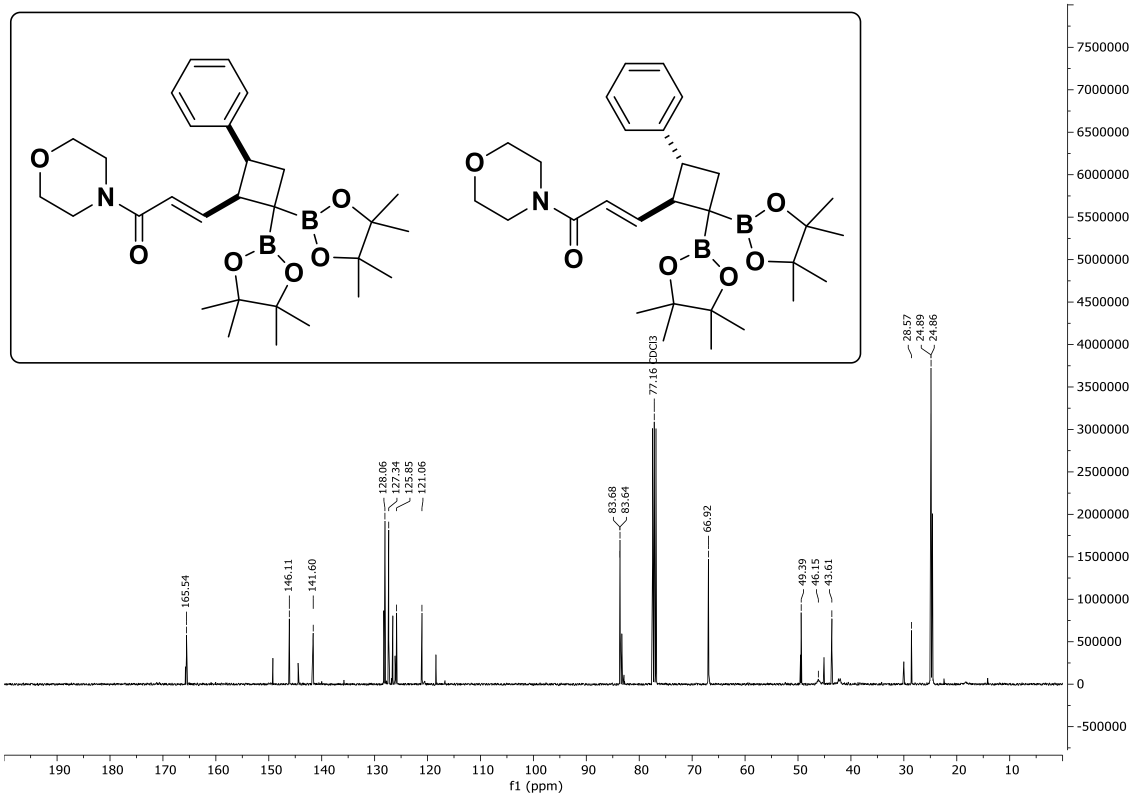

^{11}B NMR of 22 (128 MHz, $CDCl_3$)

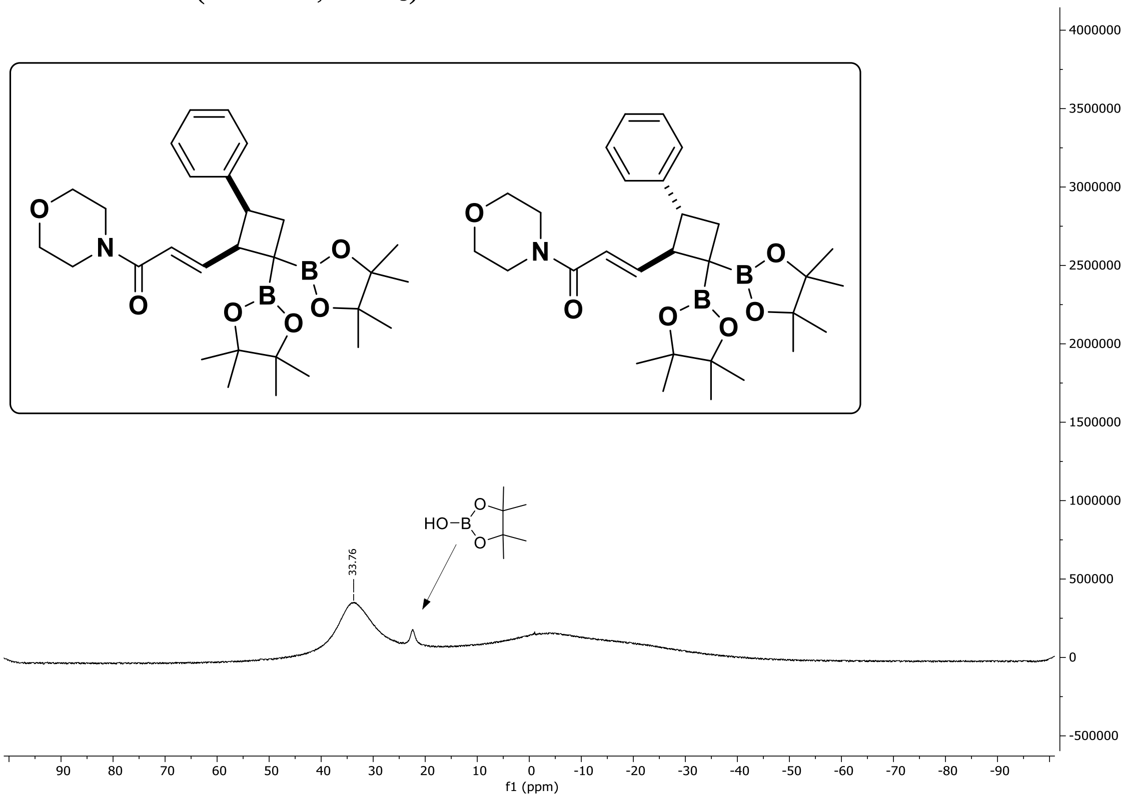

^{1}H-NMR of 23 (400 MHz, $CDCl_3$)

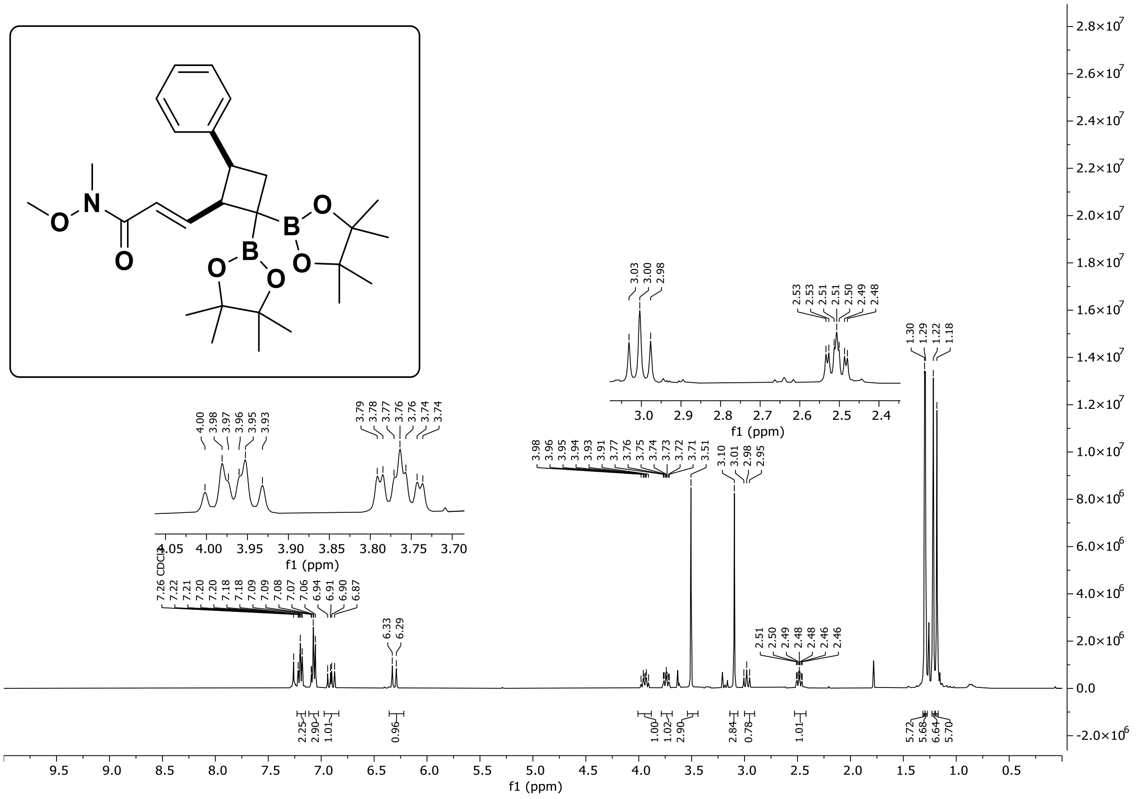

^{13}C-NMR of 23 (100 MHz, $CDCl_3$)

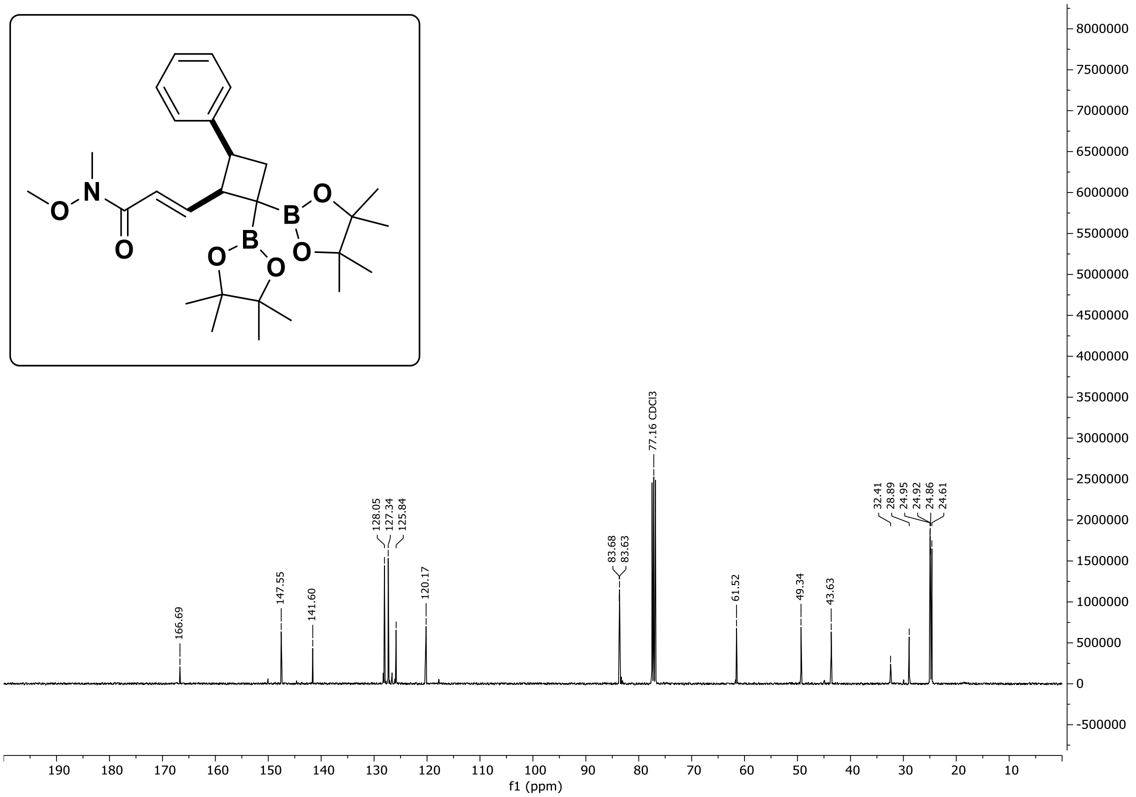

^{11}B NMR of 23 (128 MHz, $CDCl_3$)

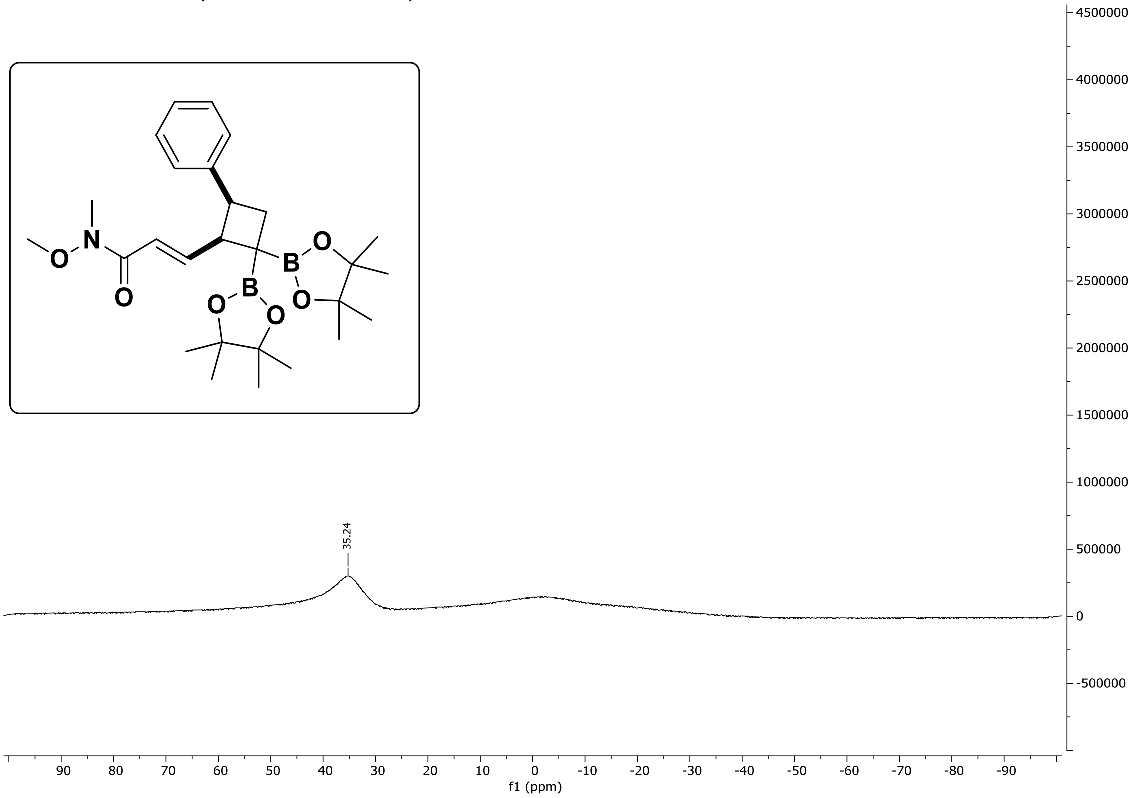

^{1}H-NMR of 24 (400 MHz, $CDCl_3$)

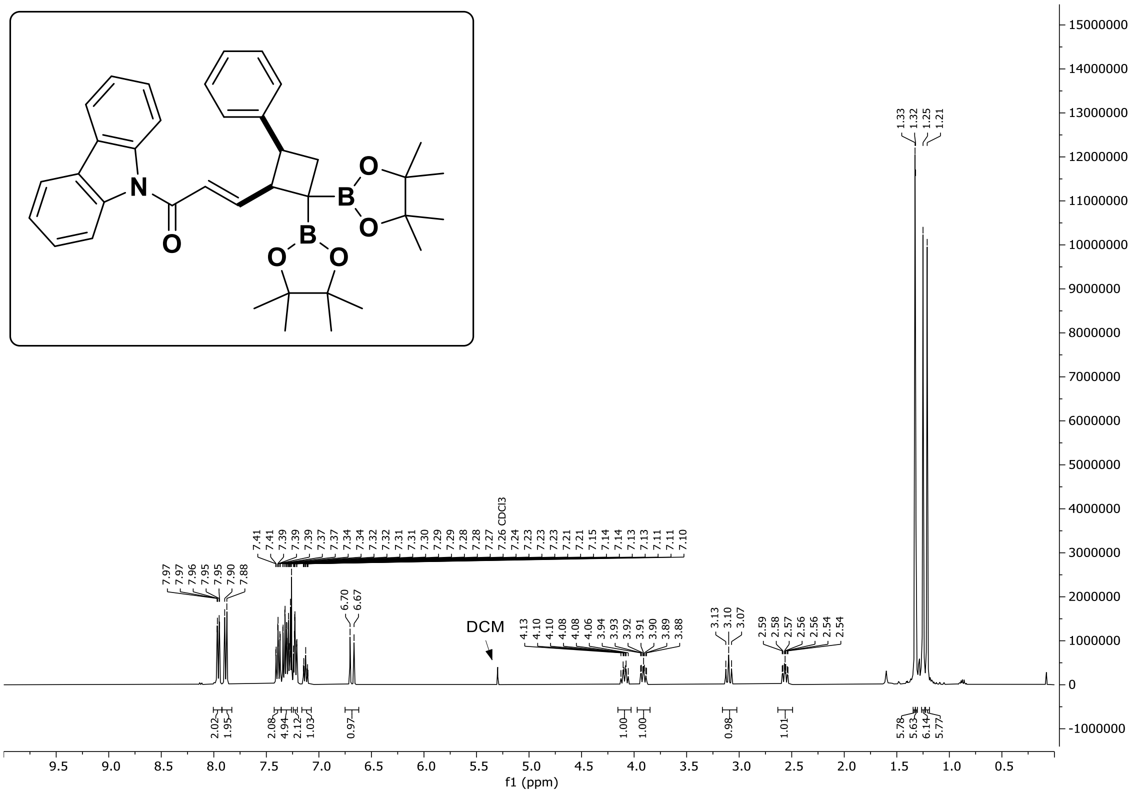

^{13}C-NMR of 24 (100 MHz, $CDCl_3$)

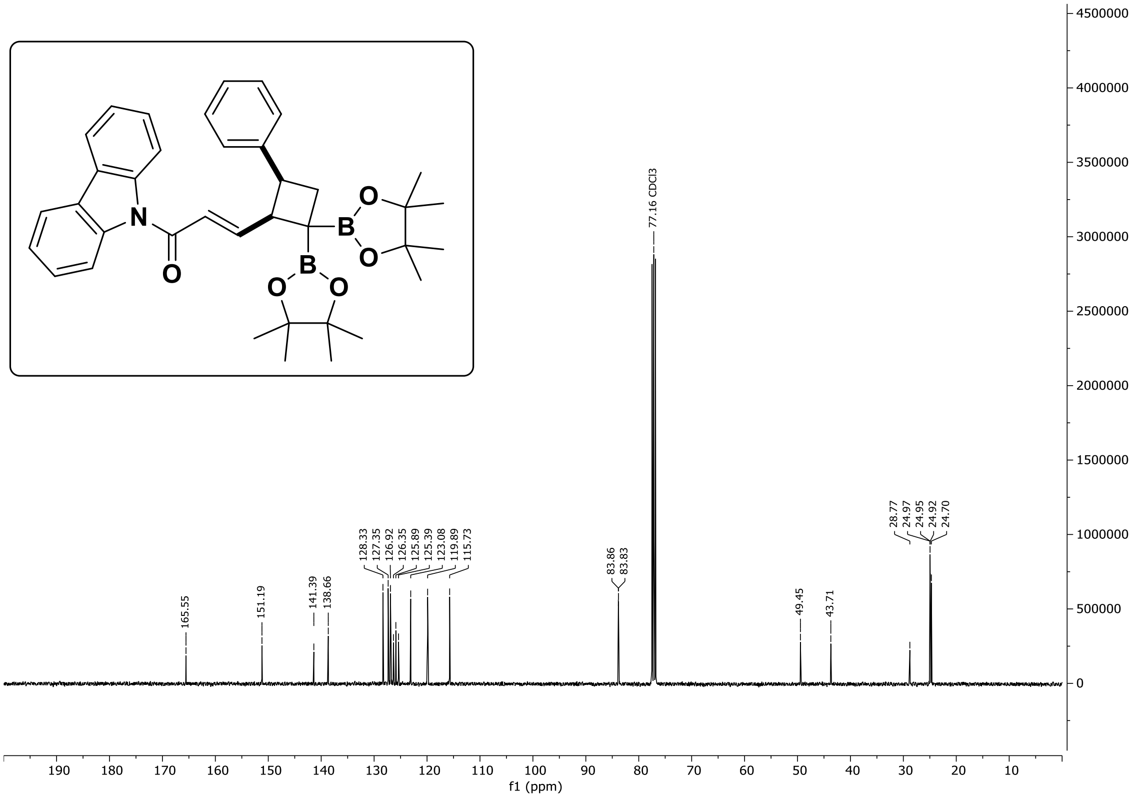

^{11}B NMR of 24 (128 MHz, $CDCl_3$)

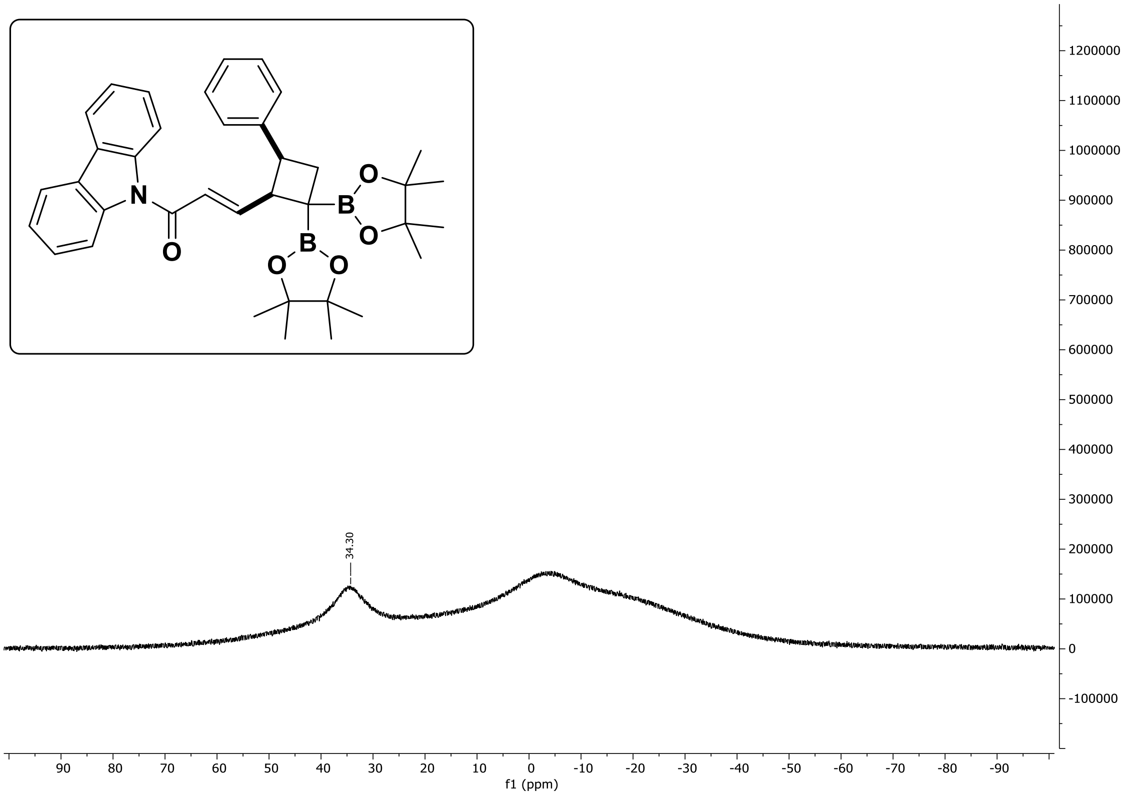

^{1}H-NMR of 25 (400 MHz, $CDCl_3$)

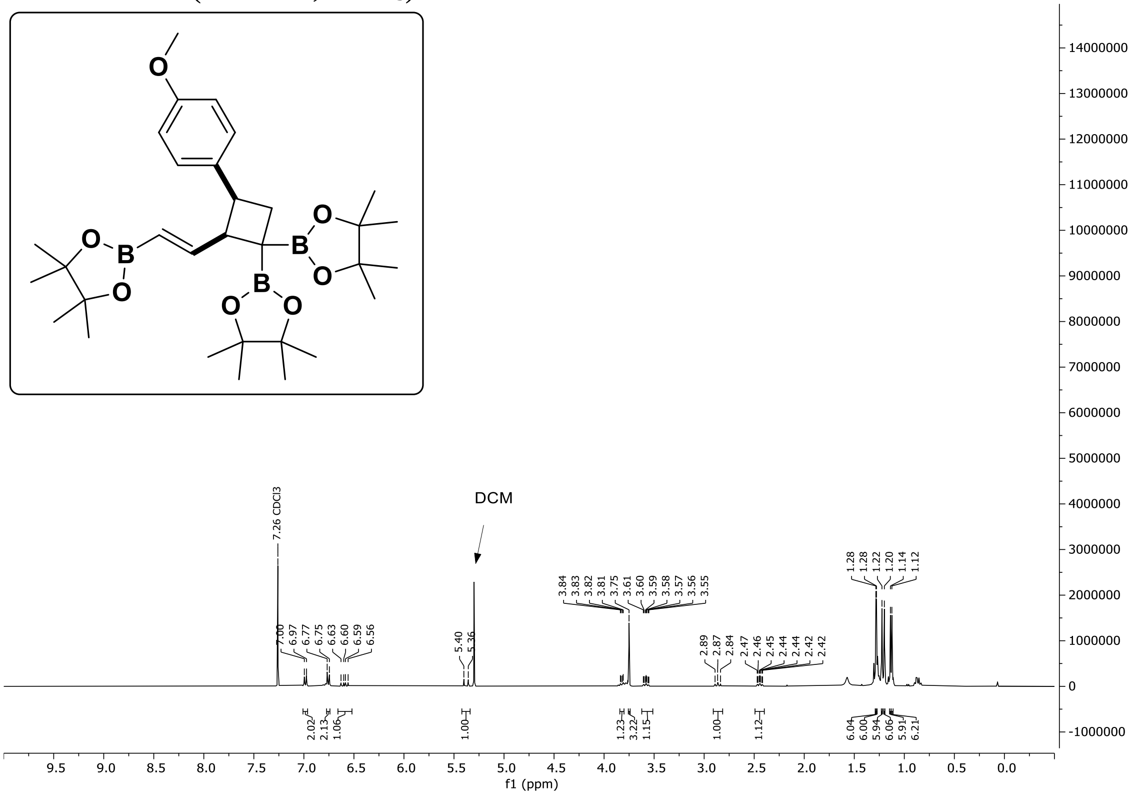

^{13}C-NMR of 25 (151 MHz, $CDCl_3$)

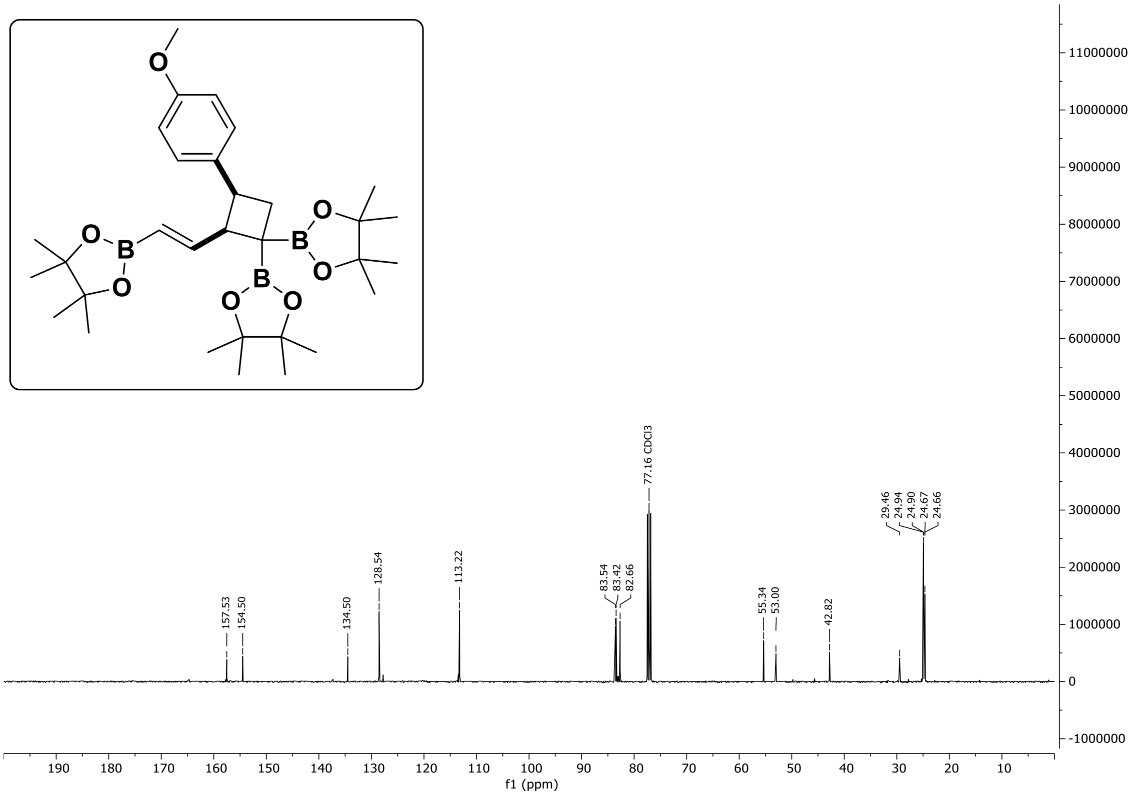

^{11}B NMR of 25 (128 MHz, $CDCl_3$)

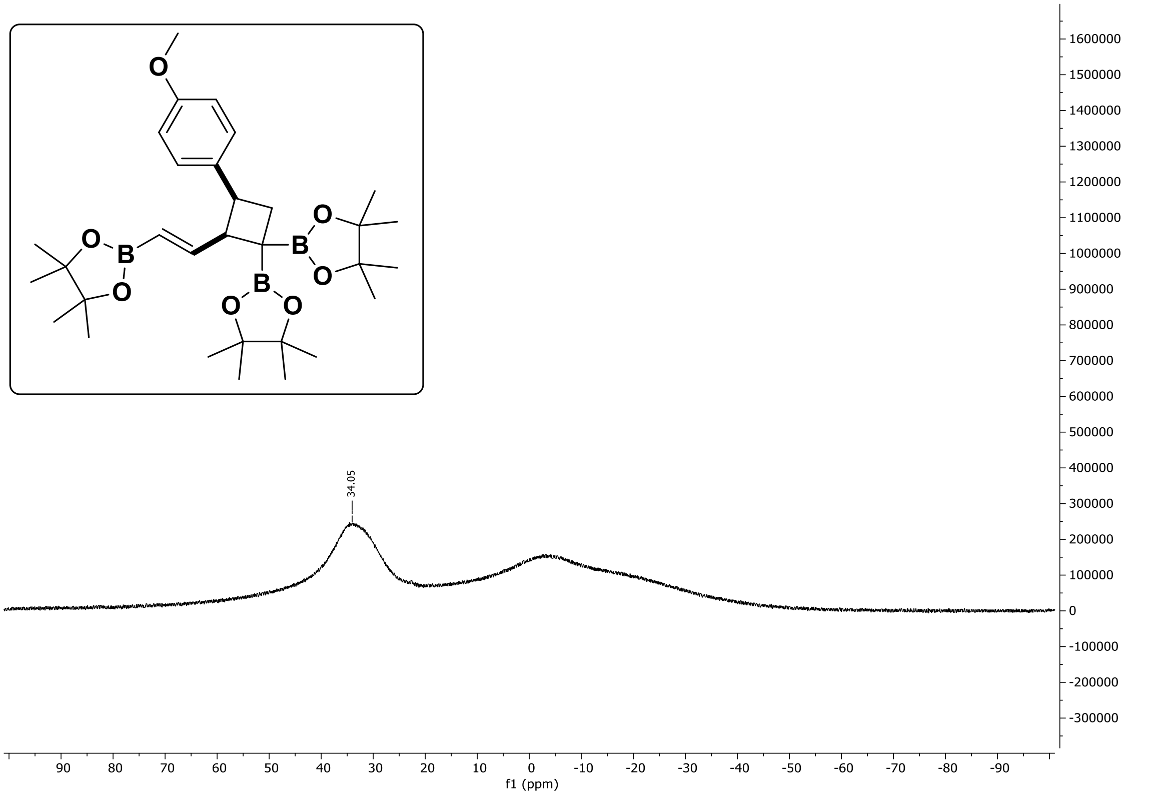

^{1}H-NMR of 26 (400 MHz, $CDCl_3$)

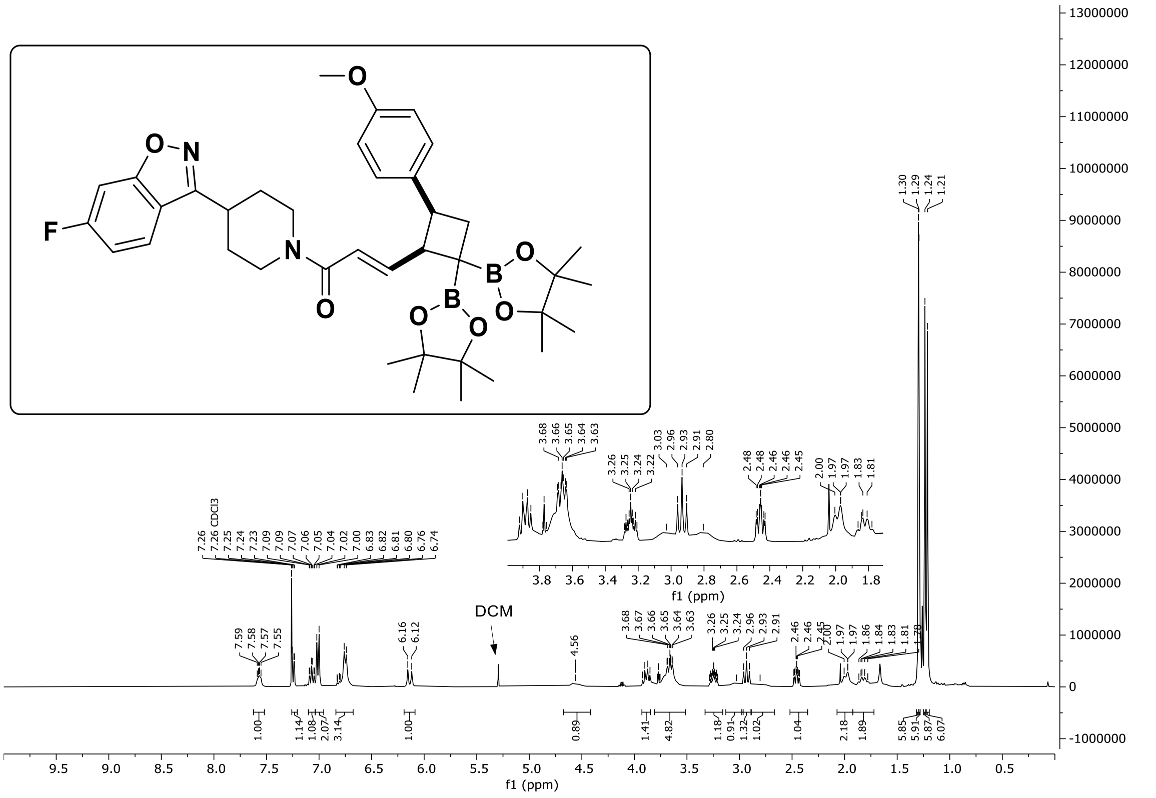

^{13}C-NMR of 26 (100 MHz, $CDCl_3$)

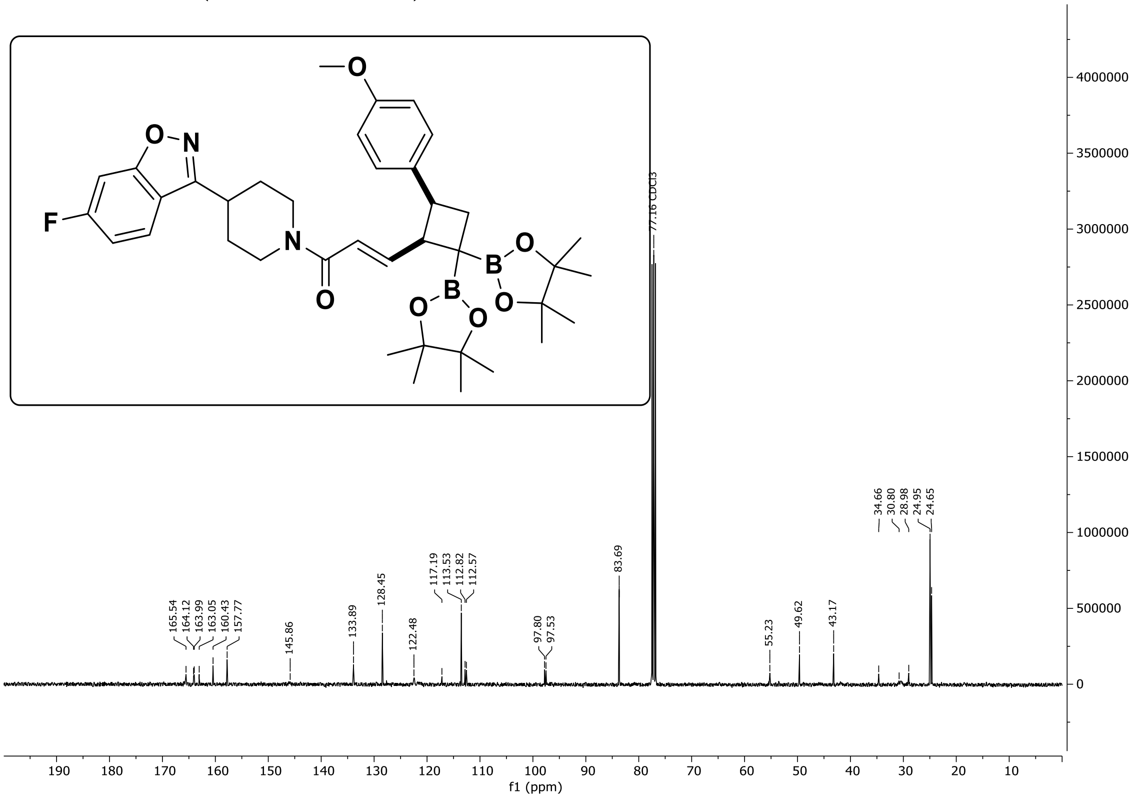

^{19}F-NMR of 26 (376 MHz, $CDCl_3$)

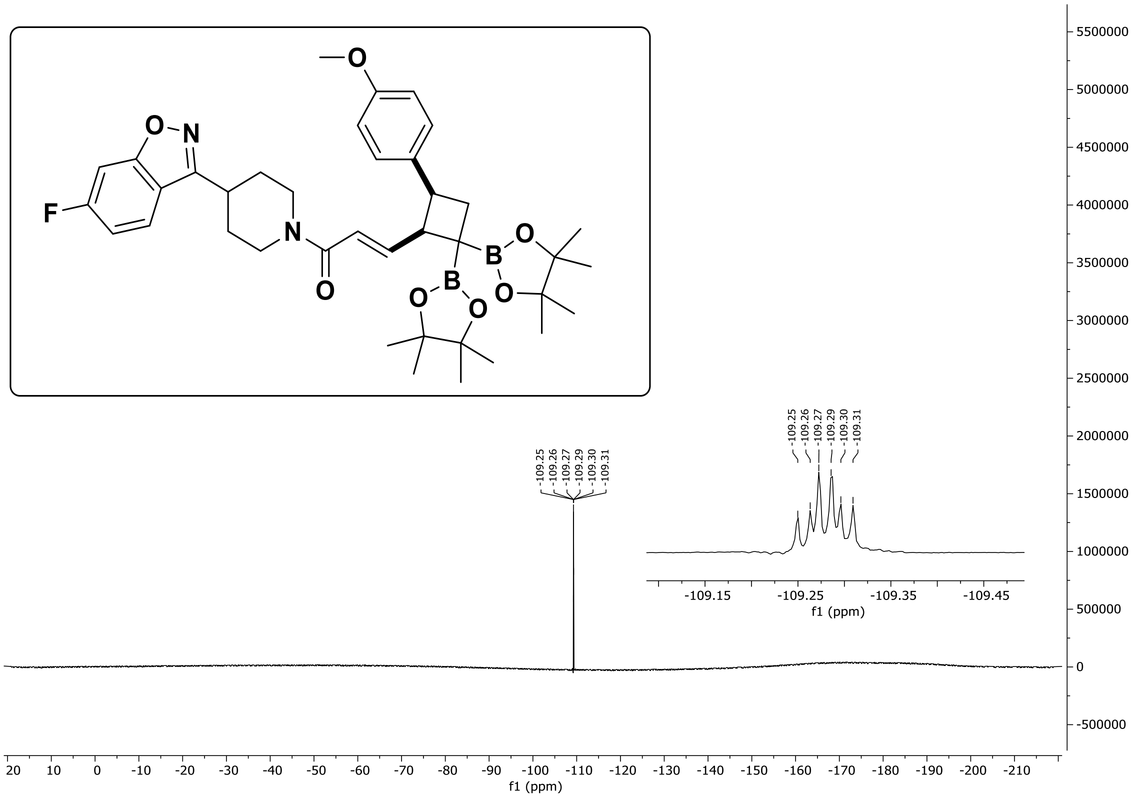

^{11}B NMR of 26 (128 MHz, $CDCl_3$)

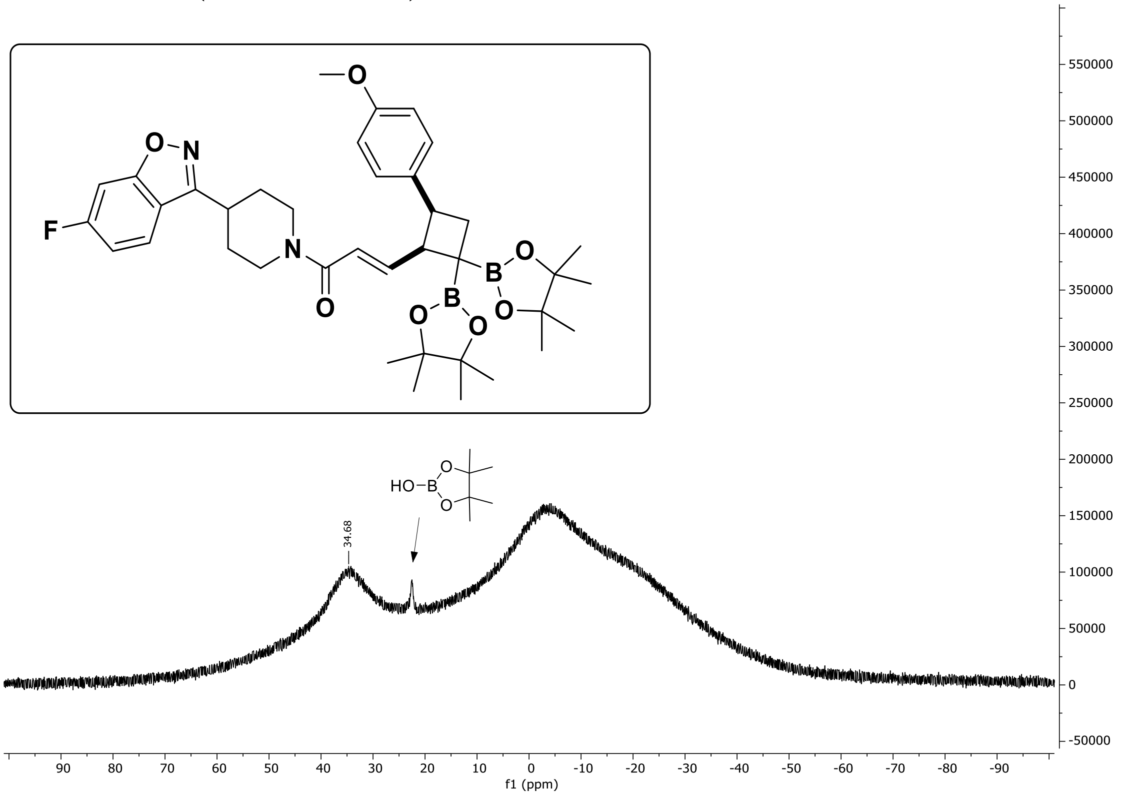

^{1}H-NMR of 27 (400 MHz, $CDCl_3$)

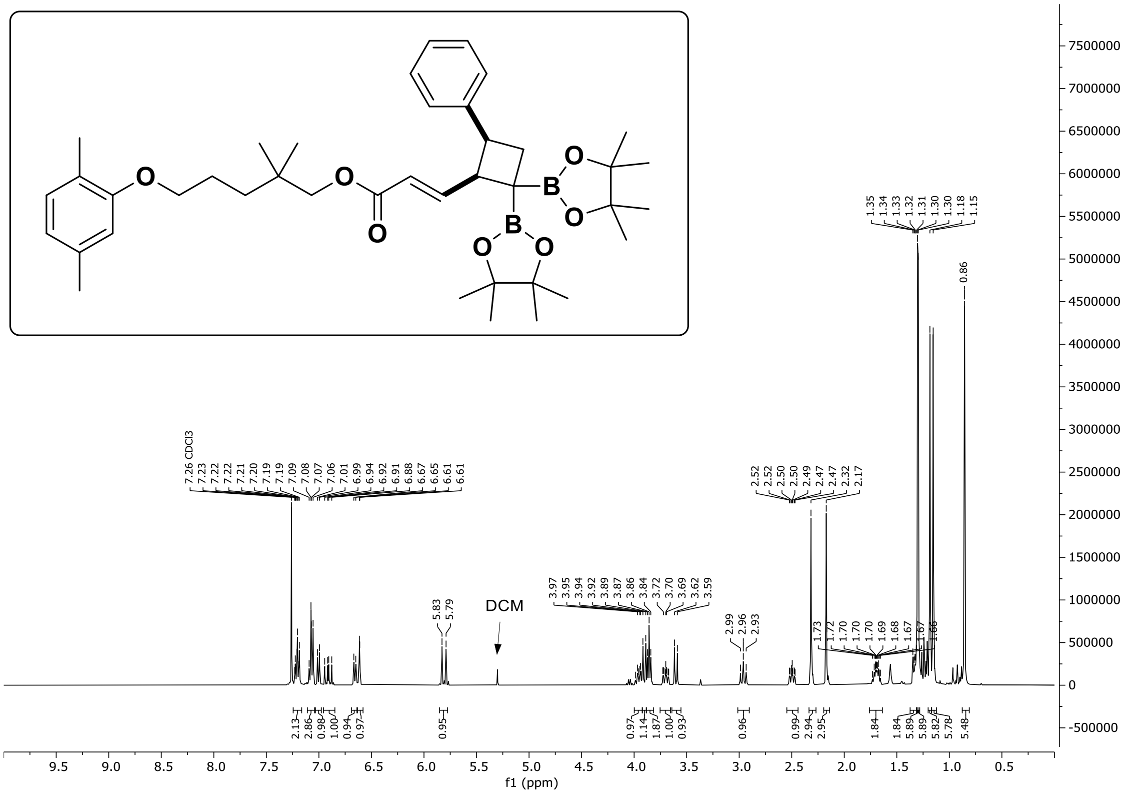

^{13}C-NMR of 27 (100 MHz, $CDCl_3$)

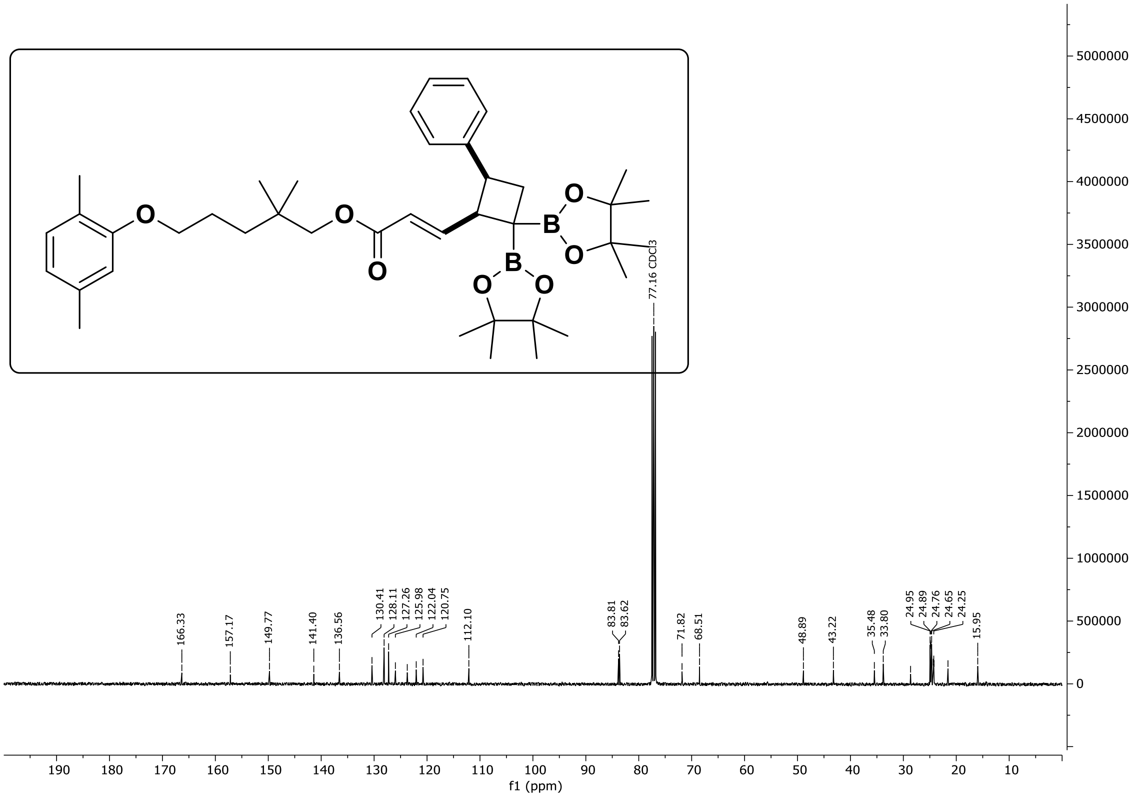

^{11}B NMR of 27 (128 MHz, $CDCl_3$)

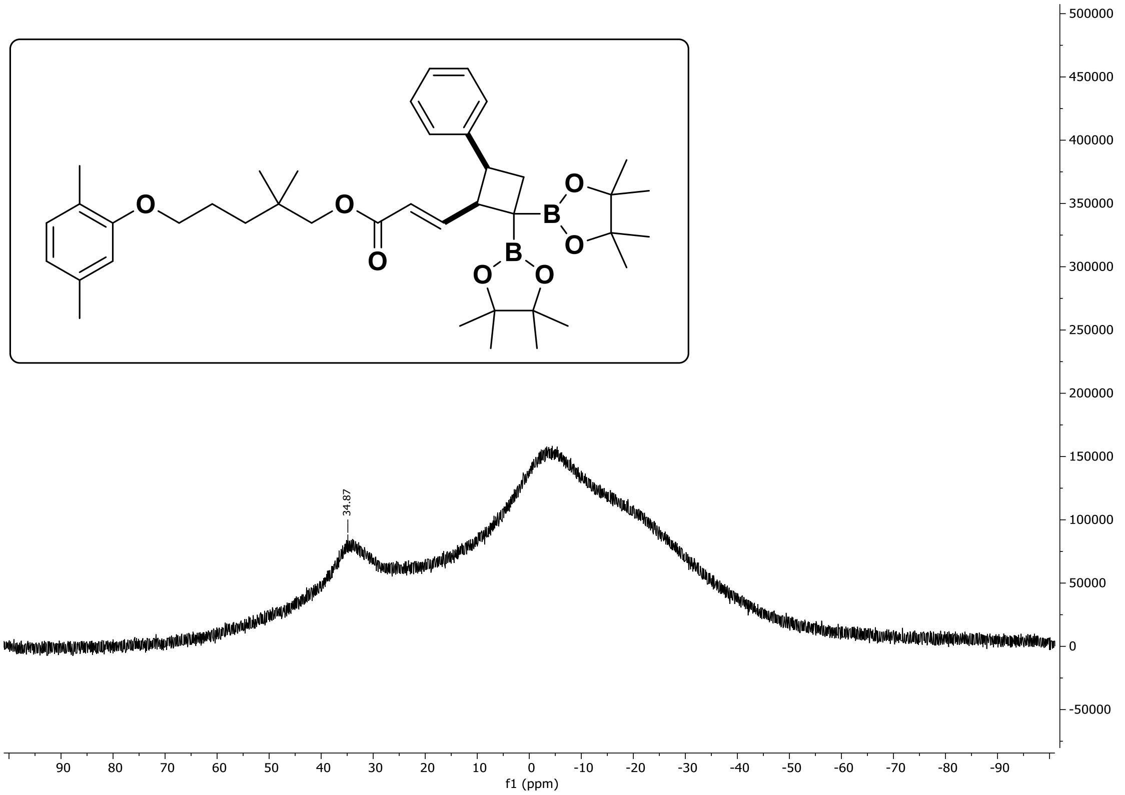

^{1}H-NMR of 28 (700 MHz, $CDCl_3$)

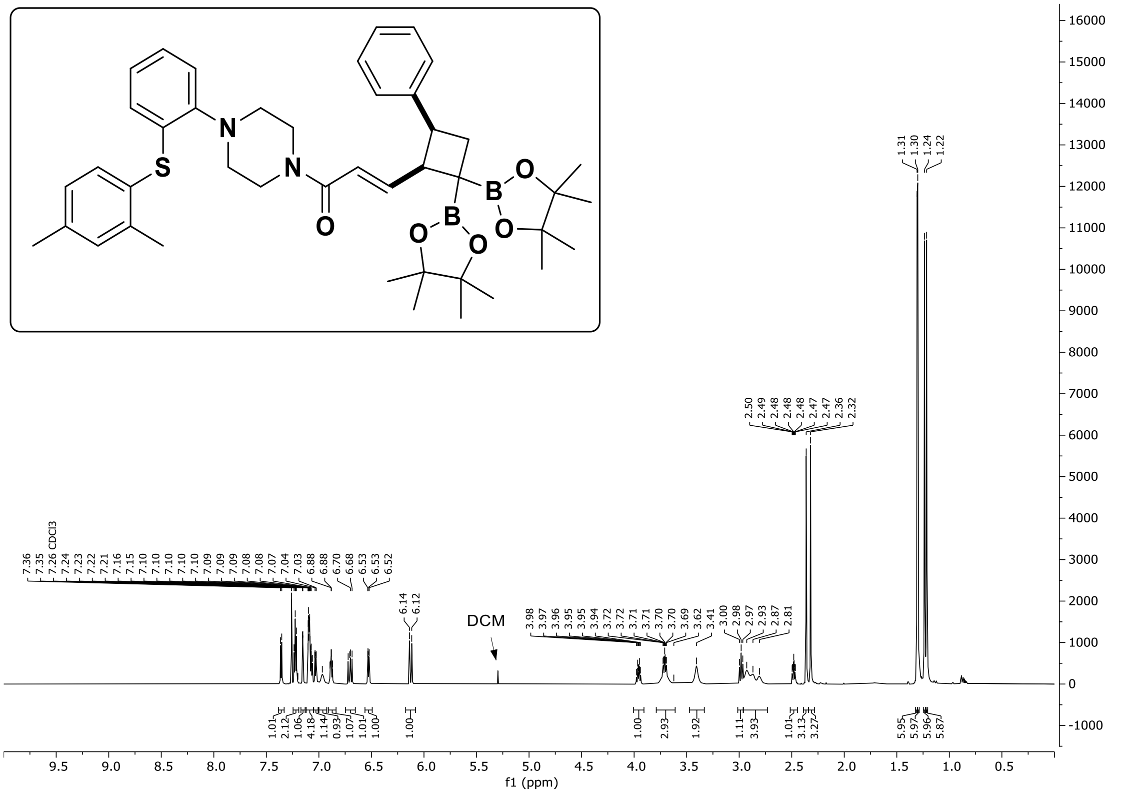

^{13}C-NMR of 28 (100 MHz, $CDCl_3$)

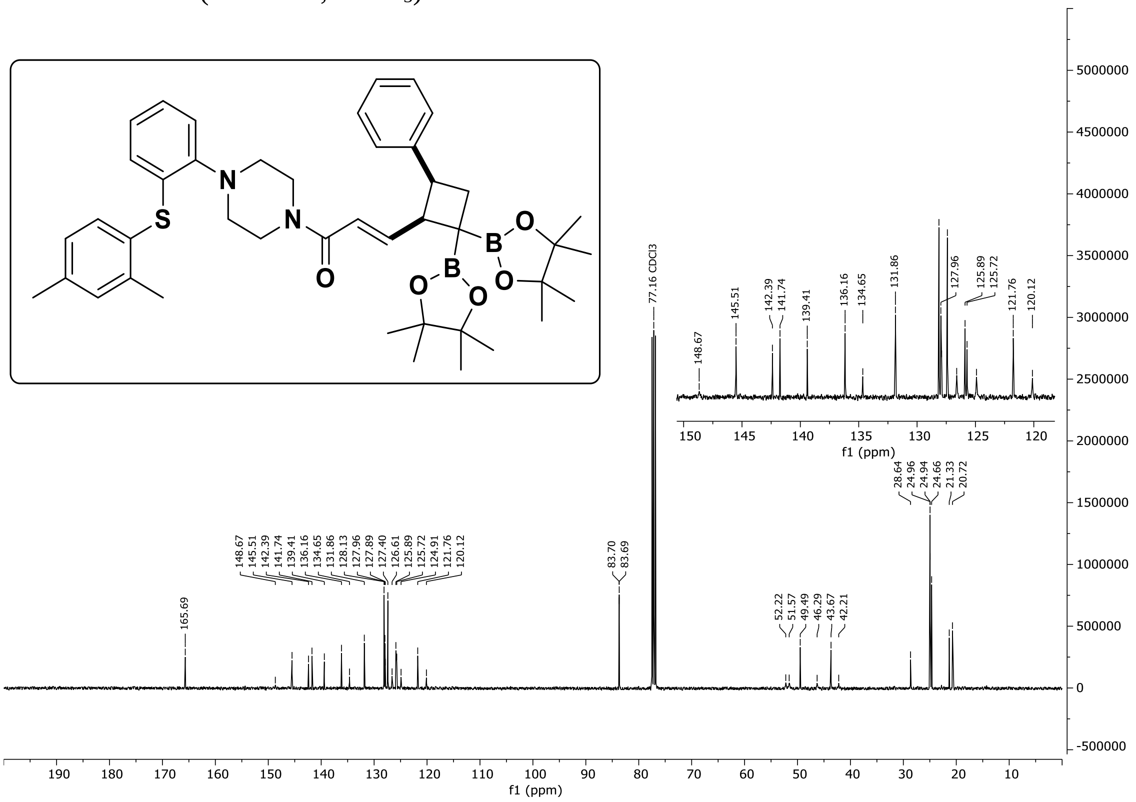

^{11}B NMR of 28 (128 MHz, $CDCl_3$)

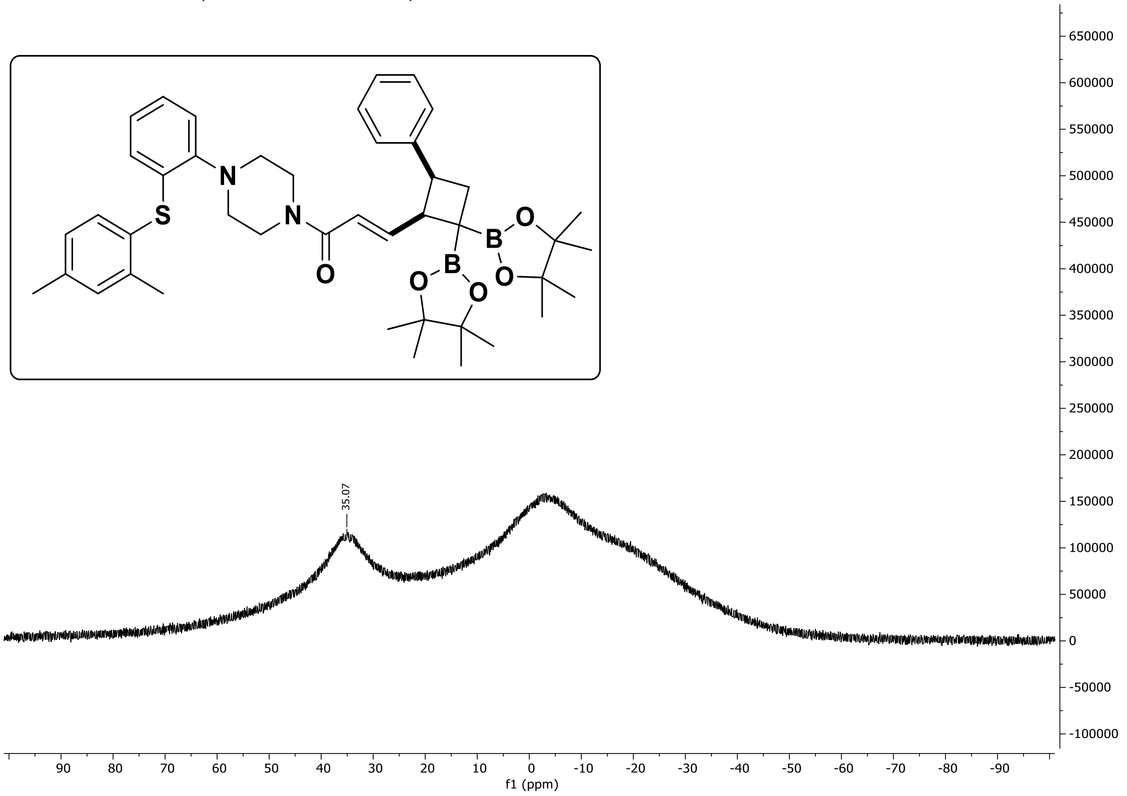

^{1}H-NMR of 29 (400 MHz, $CDCl_3$)

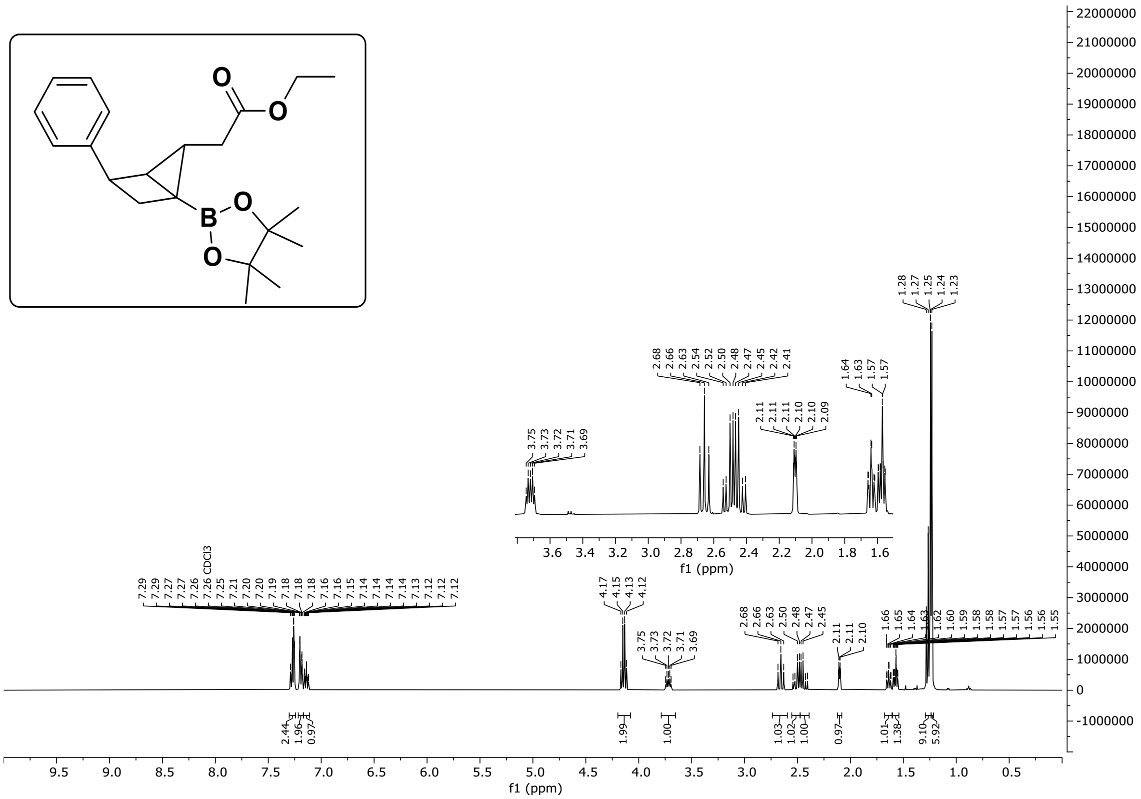

^{13}C-NMR of 29 (100 MHz, $CDCl_3$)

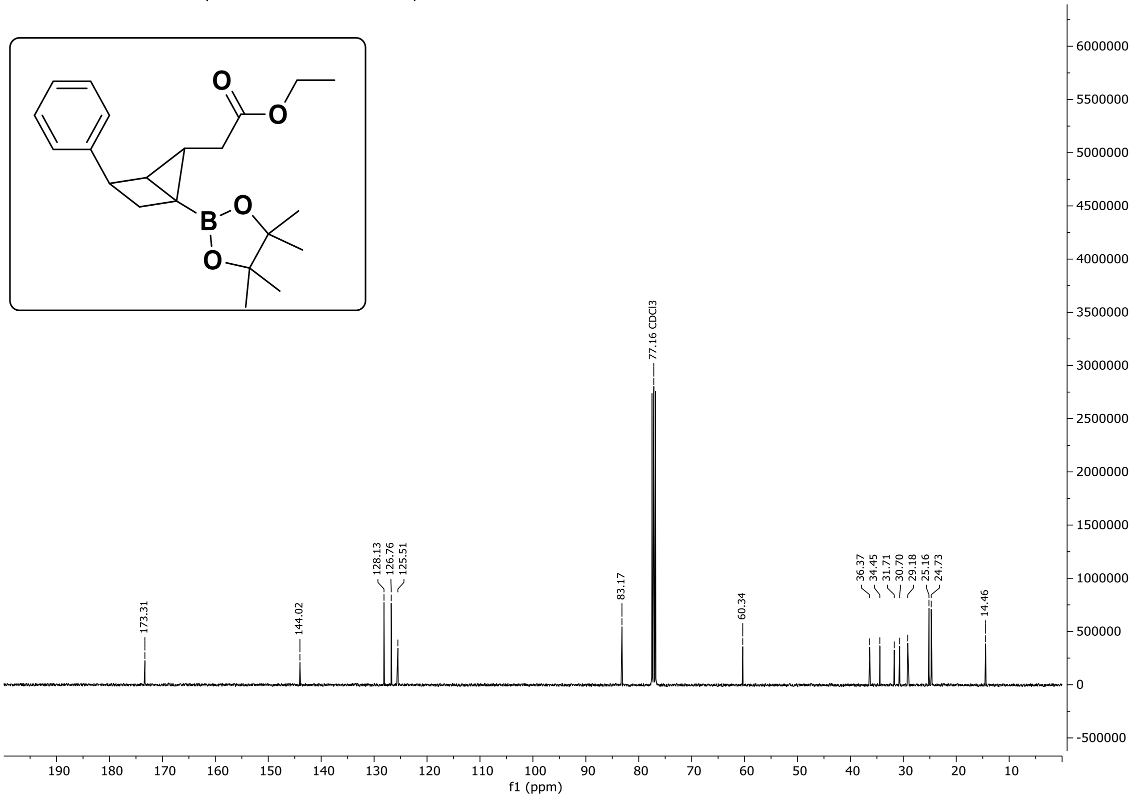

^{11}B NMR of 29 (128 MHz, $CDCl_3$)

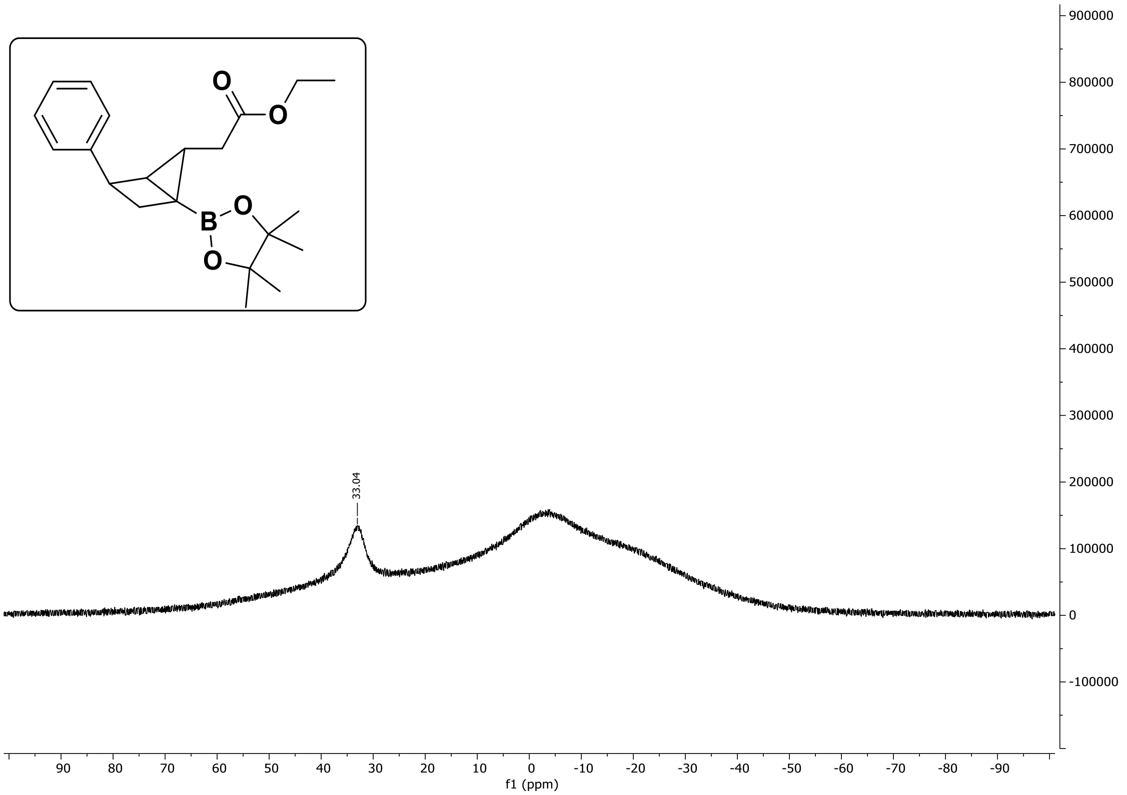

^{1}H-NMR of 30 (400 MHz, $CDCl_3$)

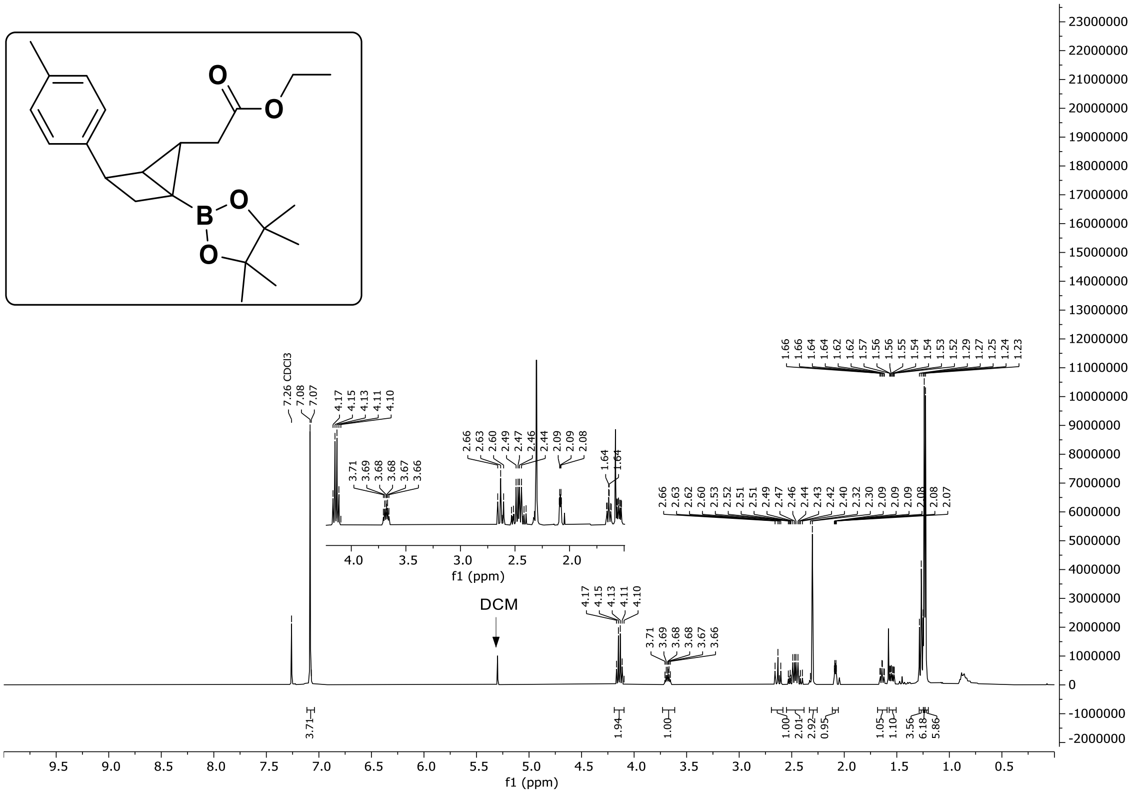

^{13}C-NMR of 30 (151 MHz, $CDCl_3$)

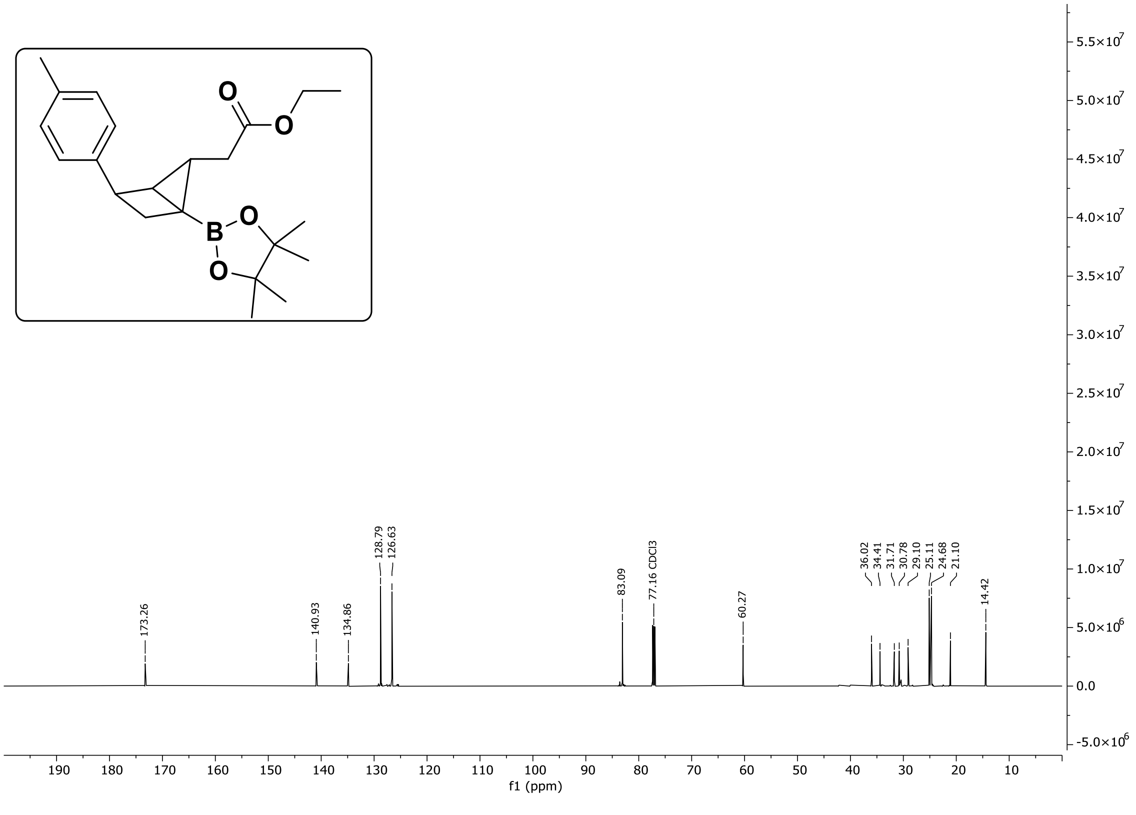

¹¹B NMR of 30 (192 MHz, $CDCl_3$)

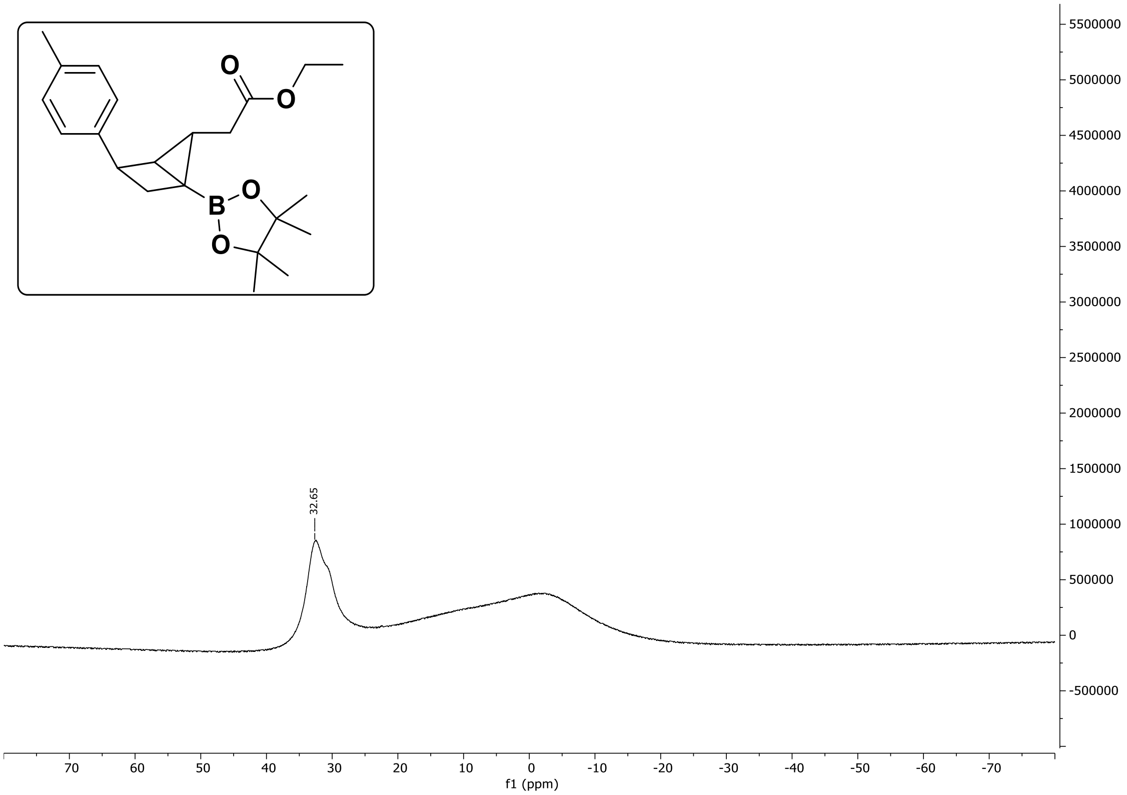

NOESY of 30:

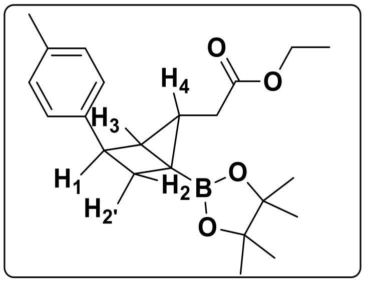

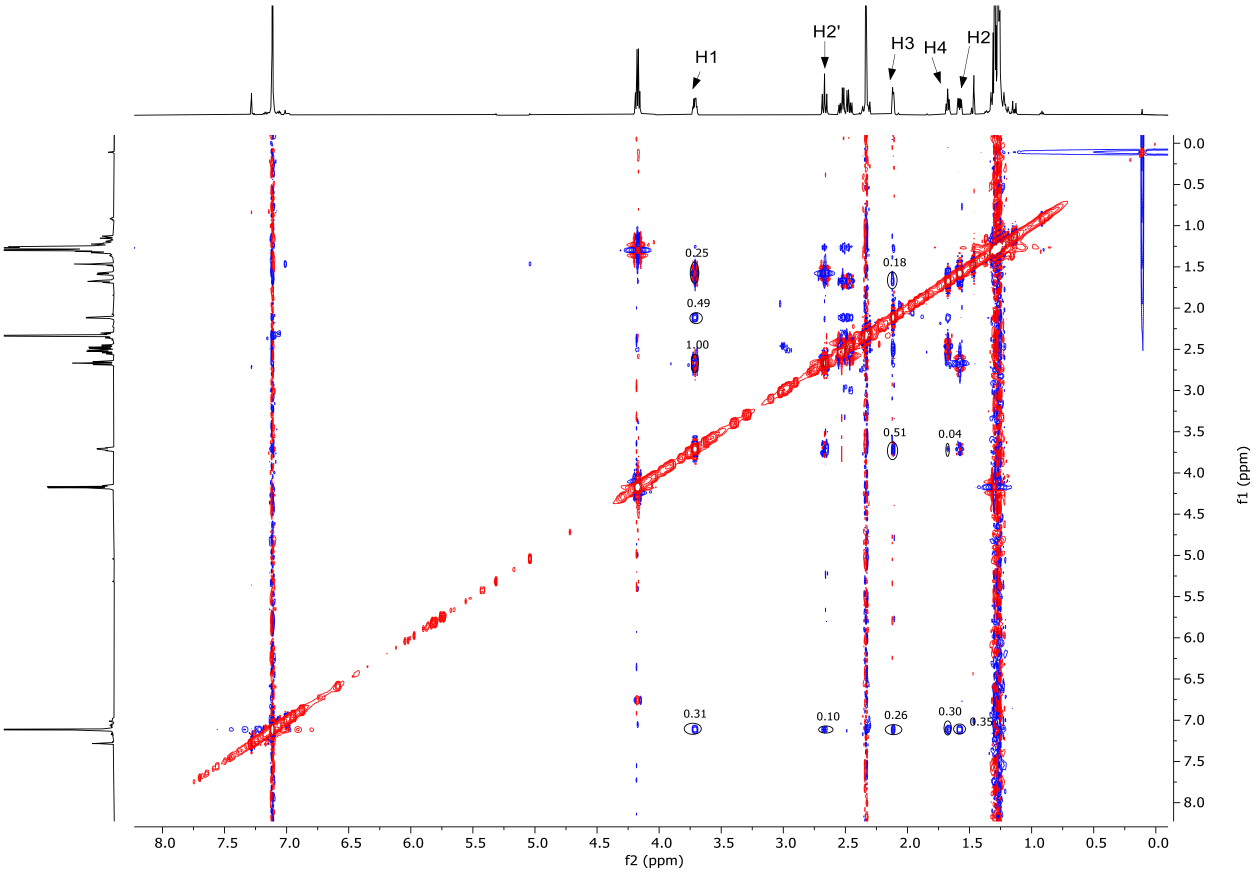

H1 has good nOe with H2', H3, but weak nOe with H2 and H4;

H2 has good nOe with ArH, but H2' has weak nOe with ArH;

H3 and H4 have good nOe with ArH.

^{1}H-NMR of 31 (400 MHz, $CDCl_3$)

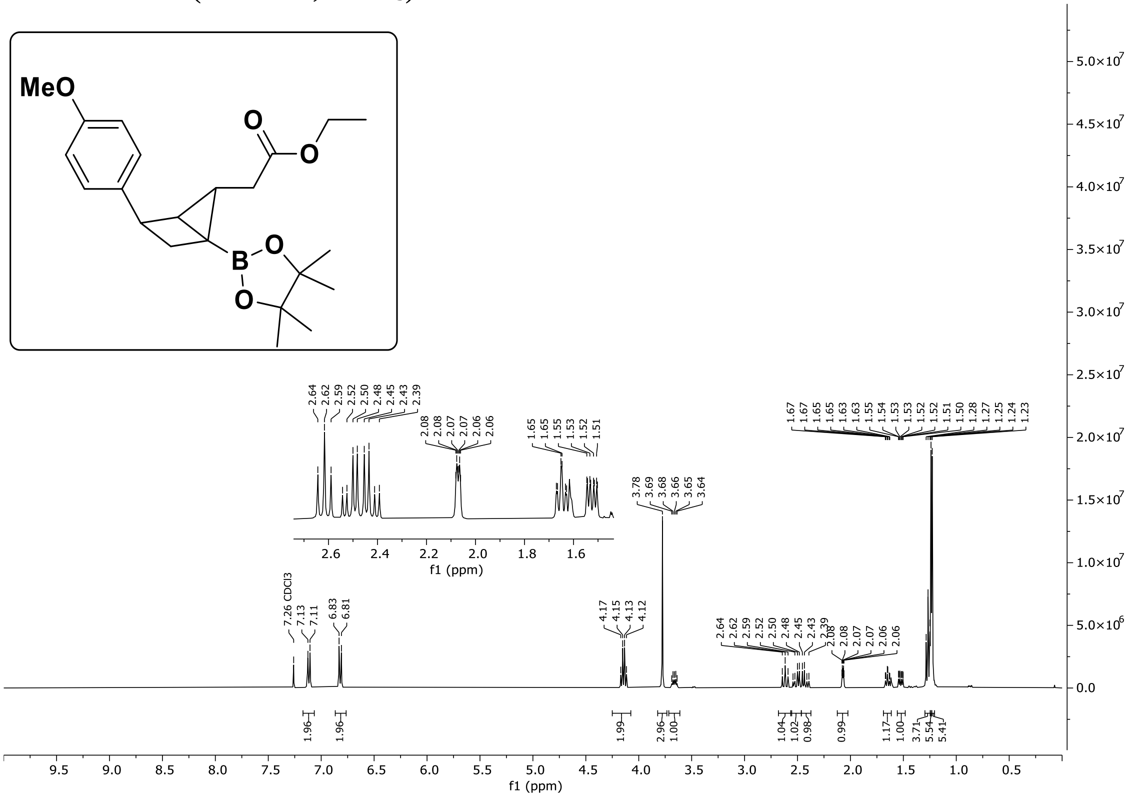

^{13}C-NMR of 31 (100 MHz, $CDCl_3$)

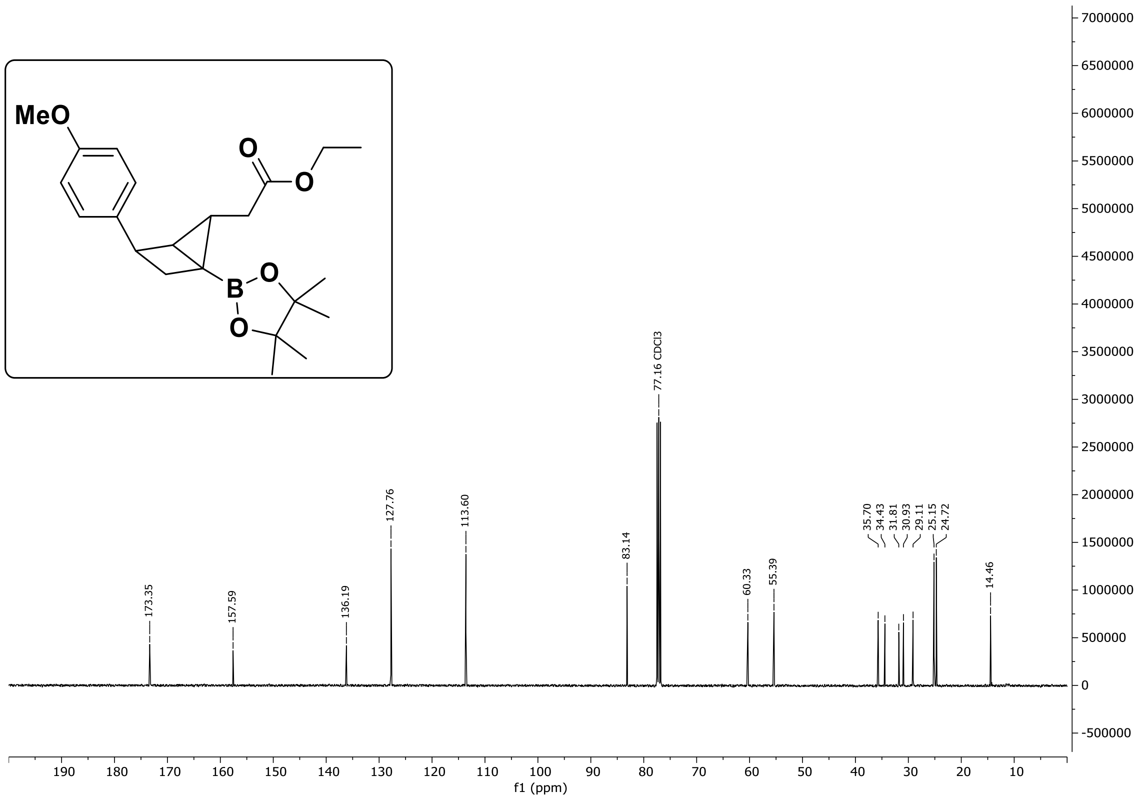

^{11}B NMR of 31 (128 MHz, $CDCl_3$)

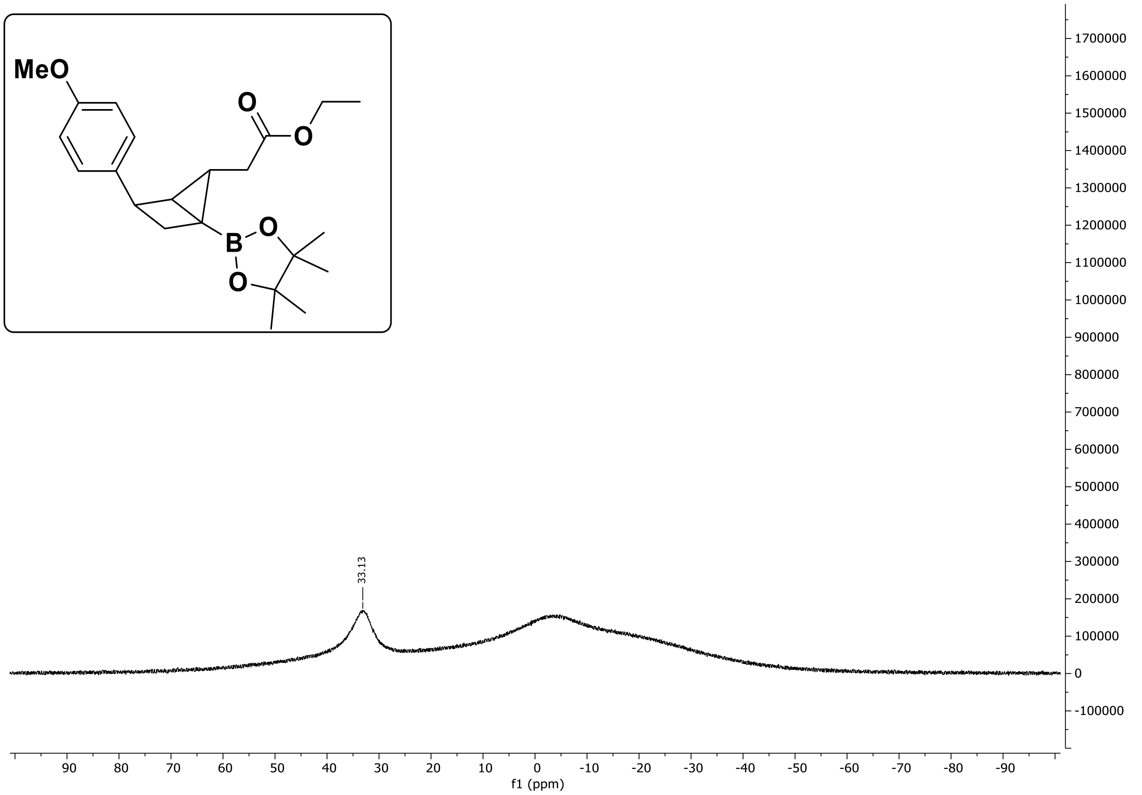

NOESY of 31:

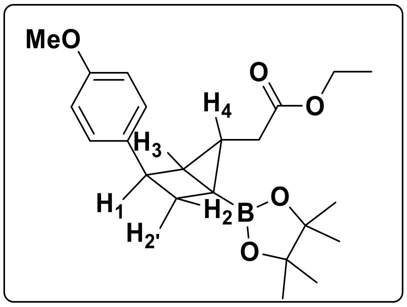

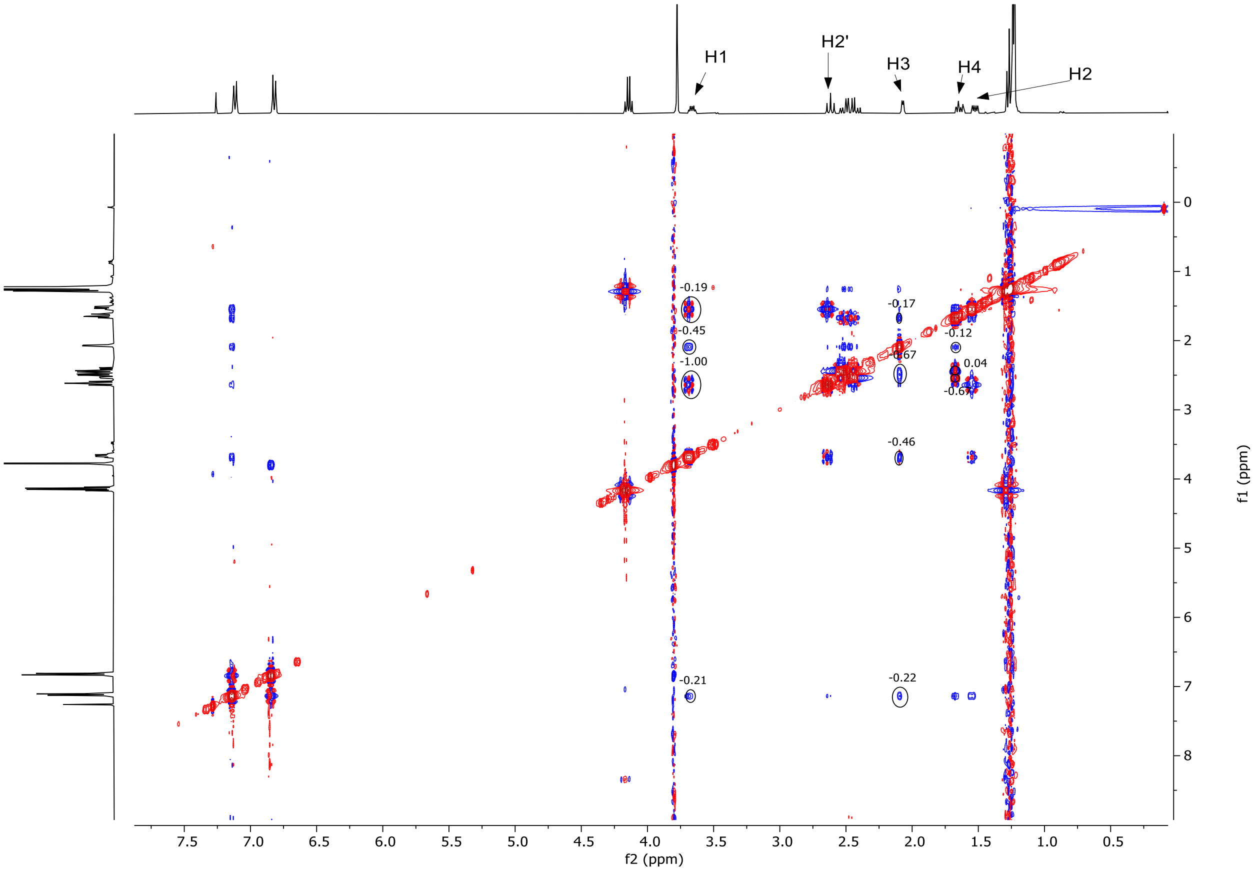

H1 has good nOe with H2’, H3, but weak nOe with H2 and H4;

H2 has good nOe with ArH, but H2’ has weak nOe with ArH;

H3 and H4 have good nOe with ArH.

^{1}H-NMR of 32 (400 MHz, $CDCl_3$)

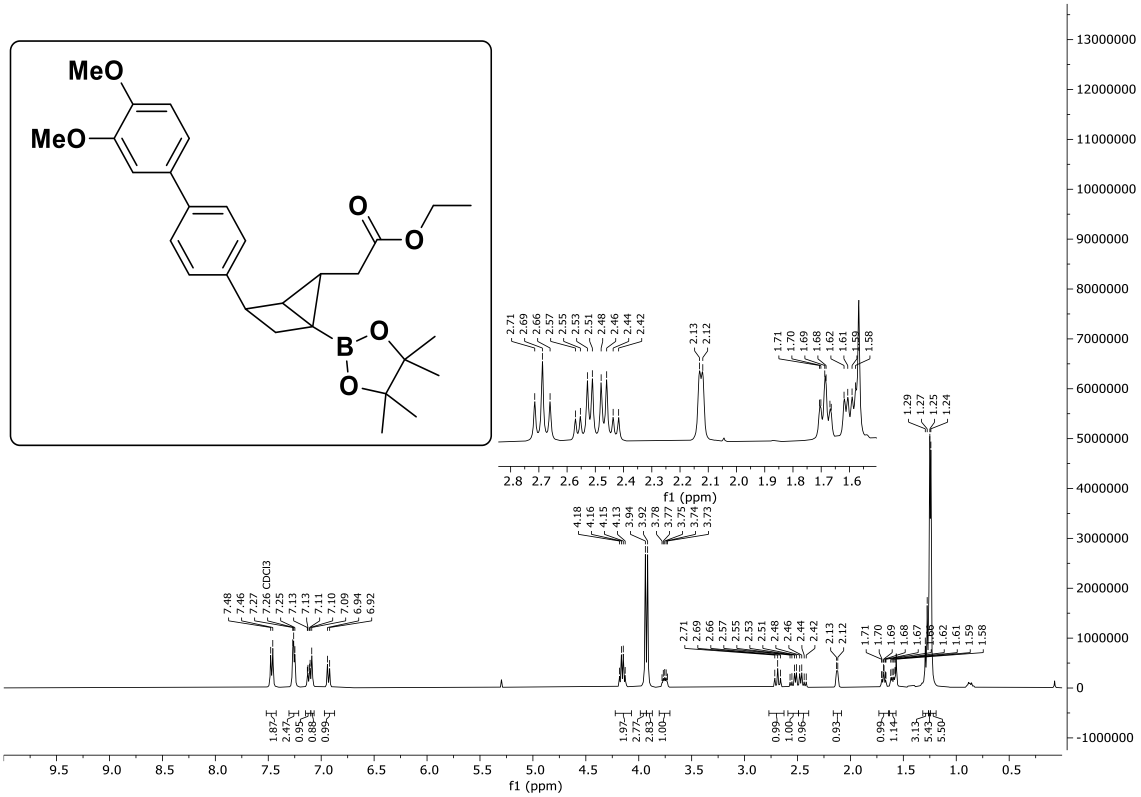

^{13}C-NMR of 32 (100 MHz, $CDCl_3$)

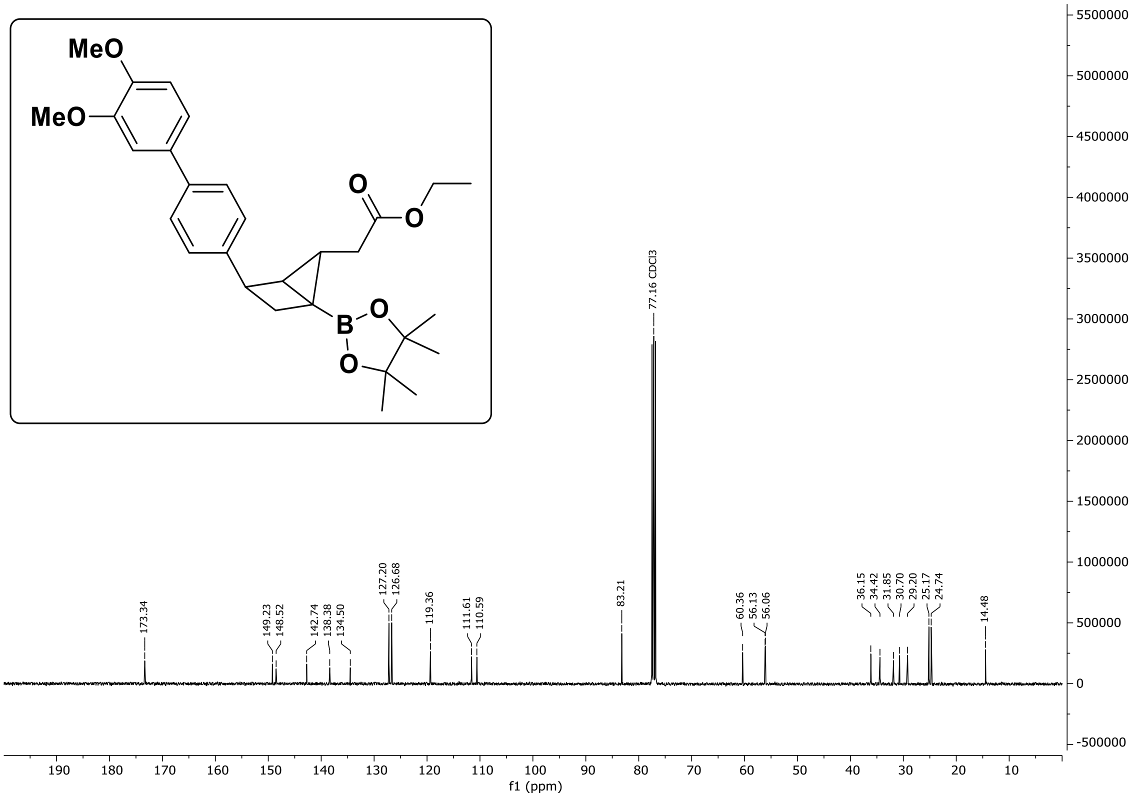

^{11}B NMR of 32 (128 MHz, $CDCl_3$)

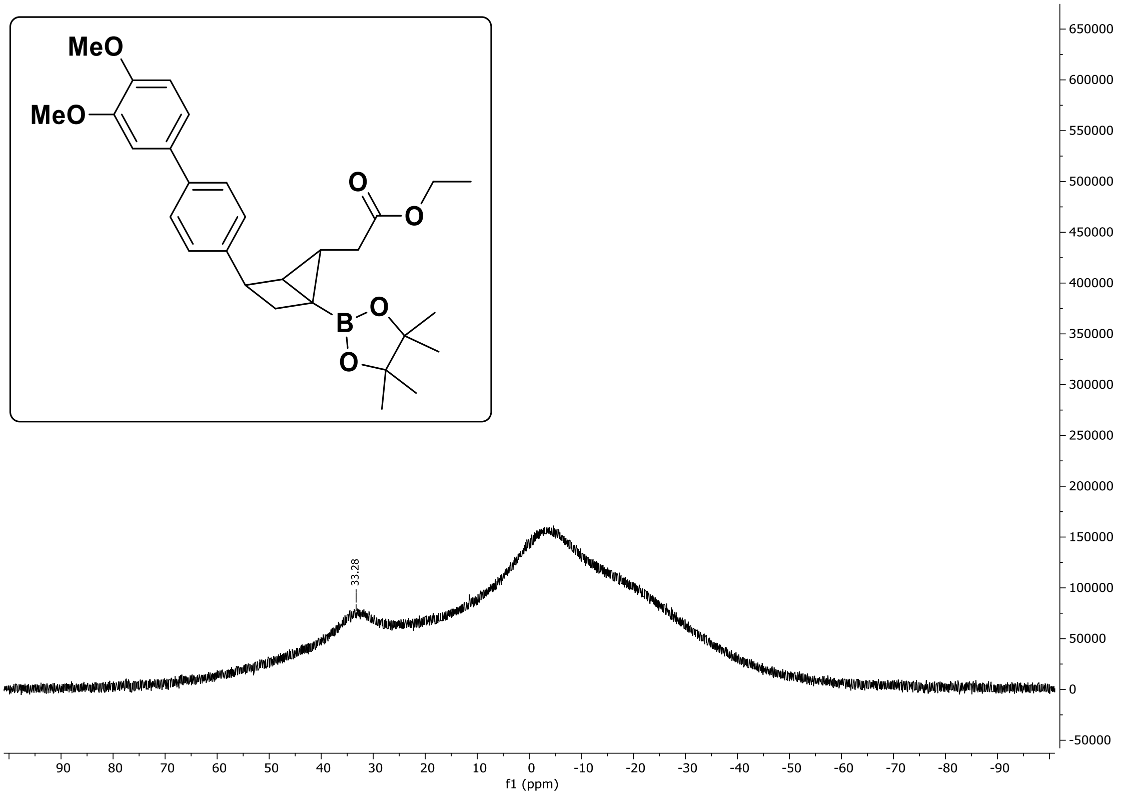

^{1}H-NMR of 33 (400 MHz, $CDCl_3$)

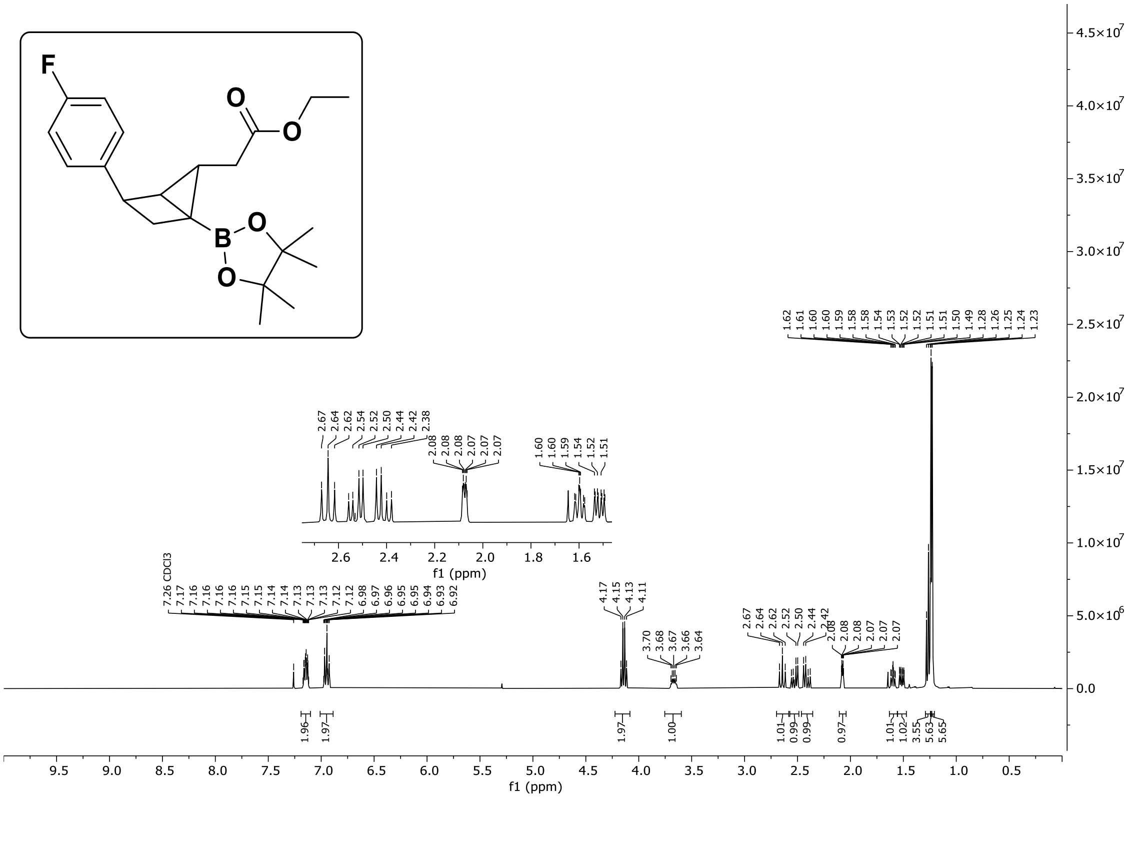

^{13}C-NMR of 33 (100 MHz, $CDCl_3$)

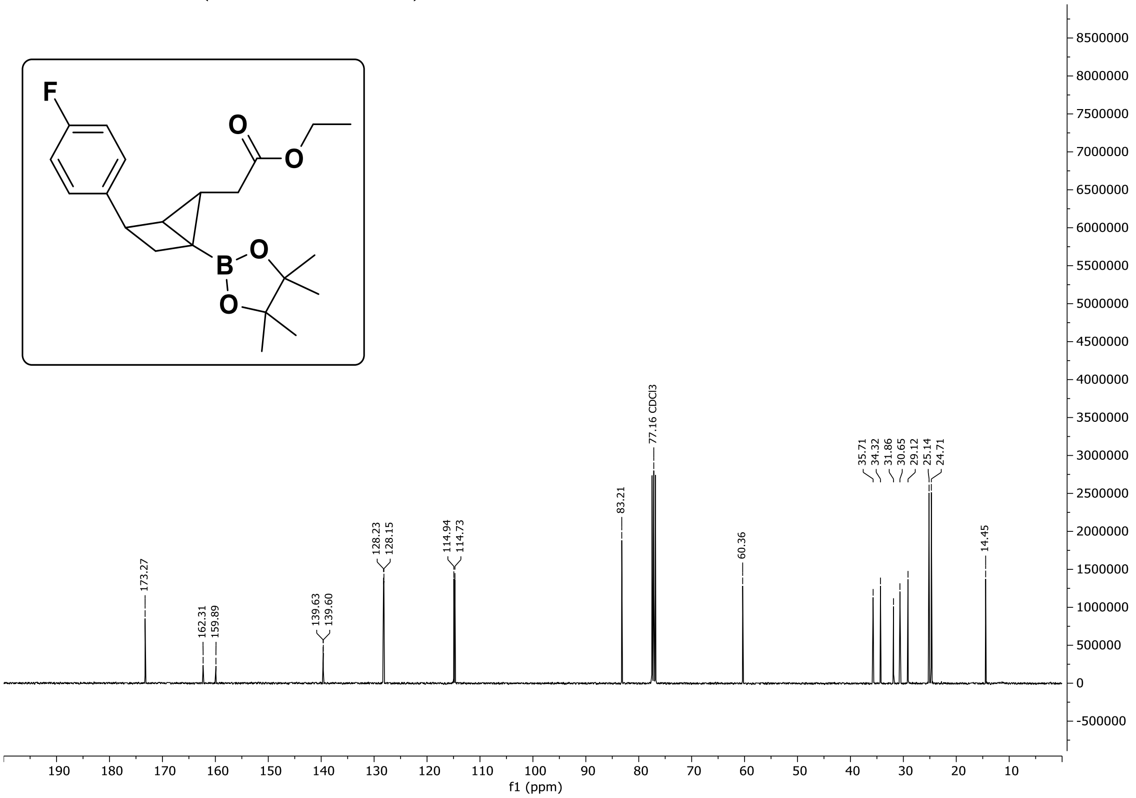

^{19}F-NMR of 33 (376 MHz, $CDCl_3$)

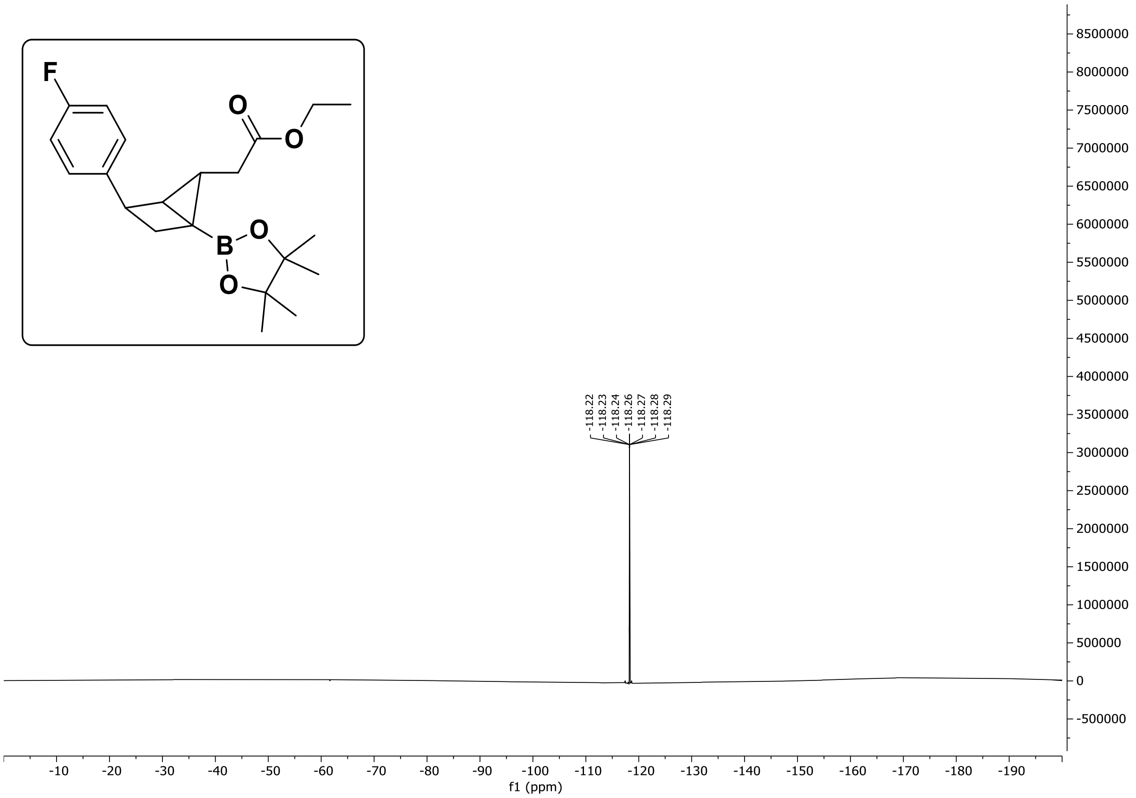

^{11}B NMR of 33 (128 MHz, $CDCl_3$)

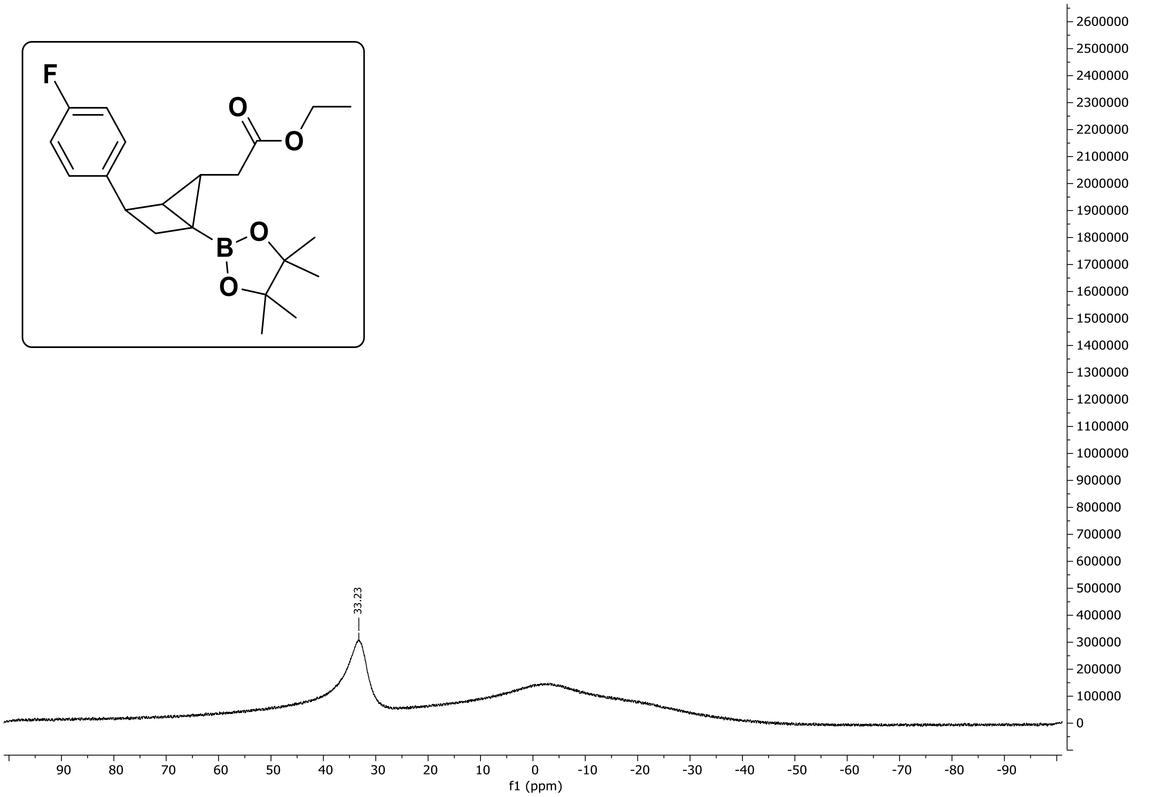

^{1}H-NMR of 34 (400 MHz, $CDCl_3$)

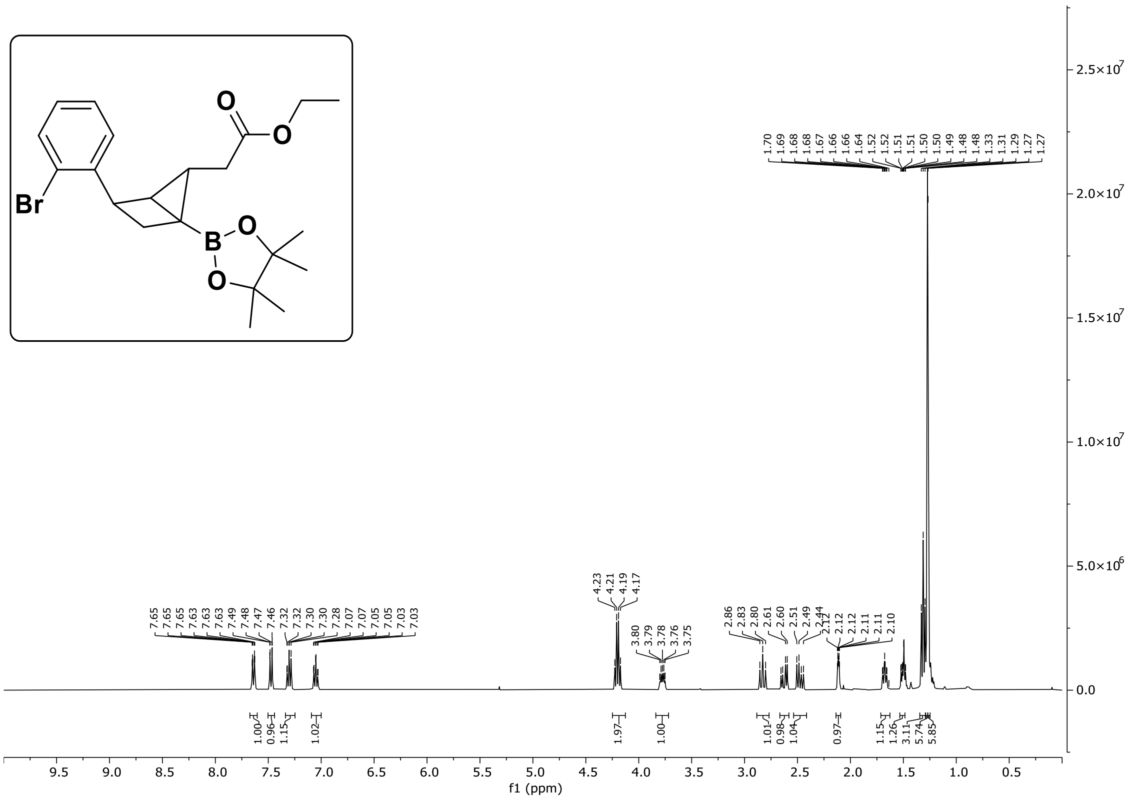

^{13}C-NMR of 34 (100 MHz, $CDCl_3$)

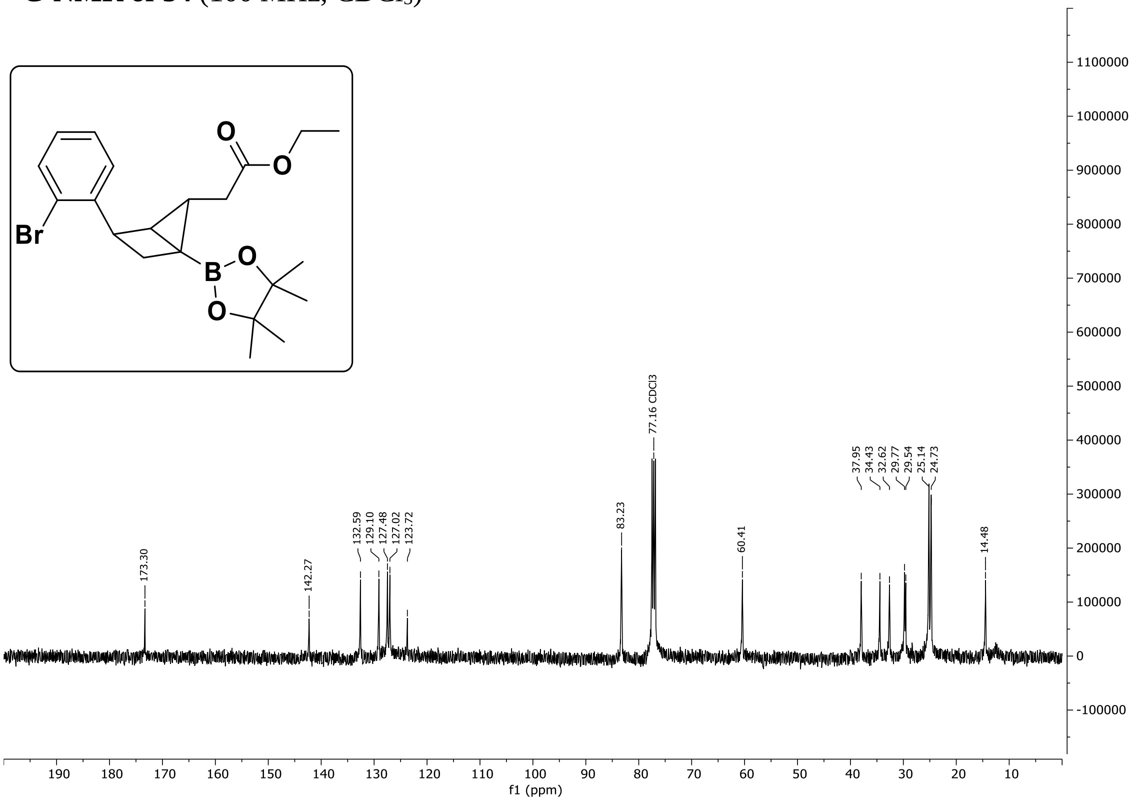

^{11}B NMR of 34 (128 MHz, $CDCl_3$)

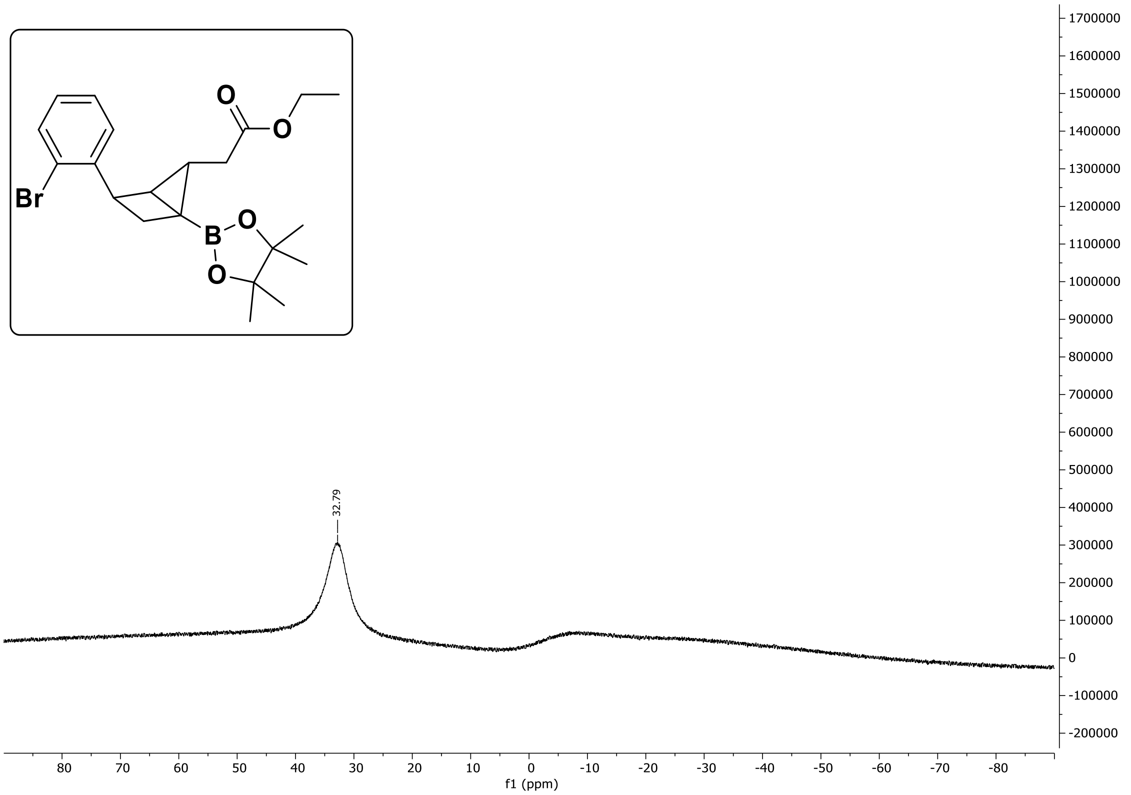

^{1}H-NMR of 35 (600 MHz, $CDCl_3$)

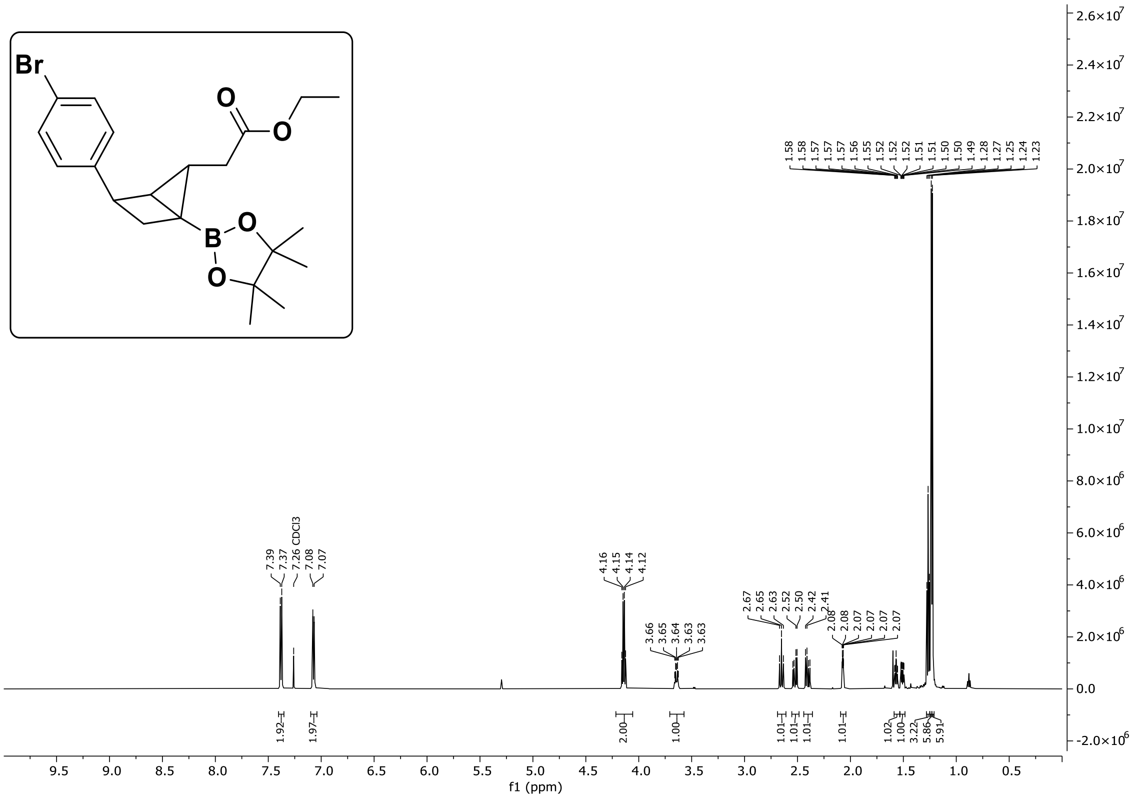

^{13}C-NMR of 35 (100 MHz, $CDCl_3$)

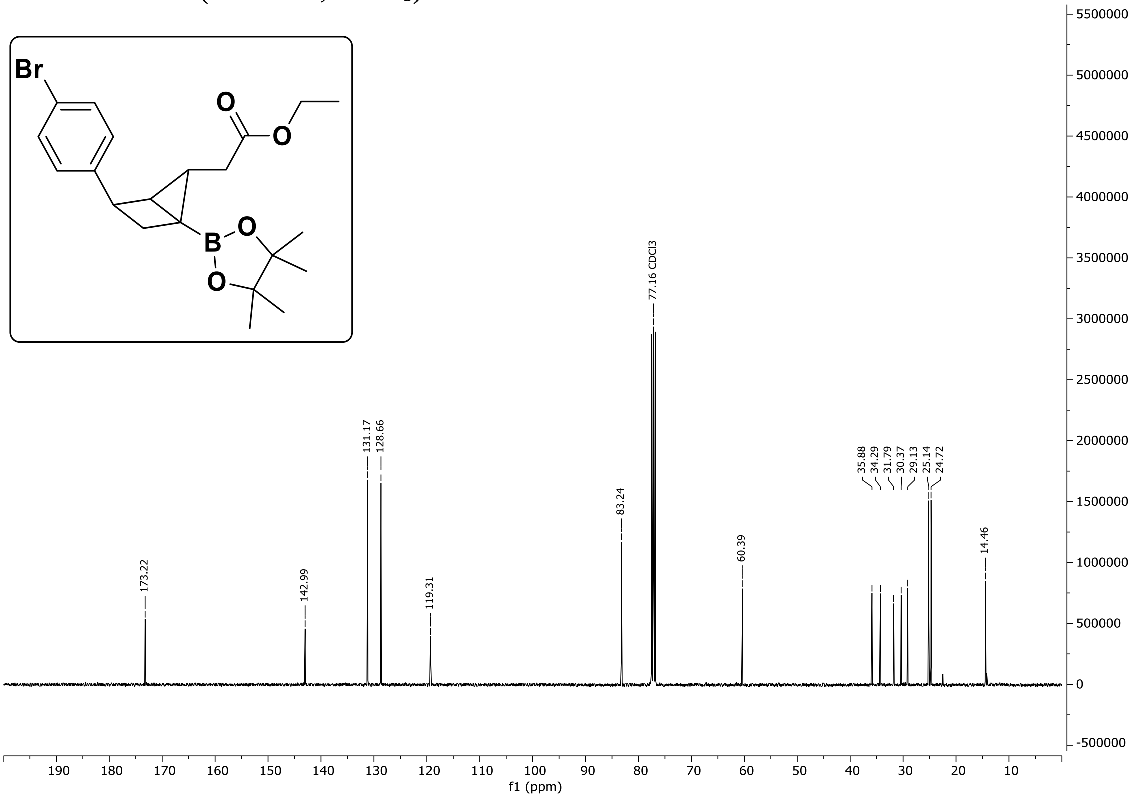

^{11}B NMR of 35 (128 MHz, $CDCl_3$)

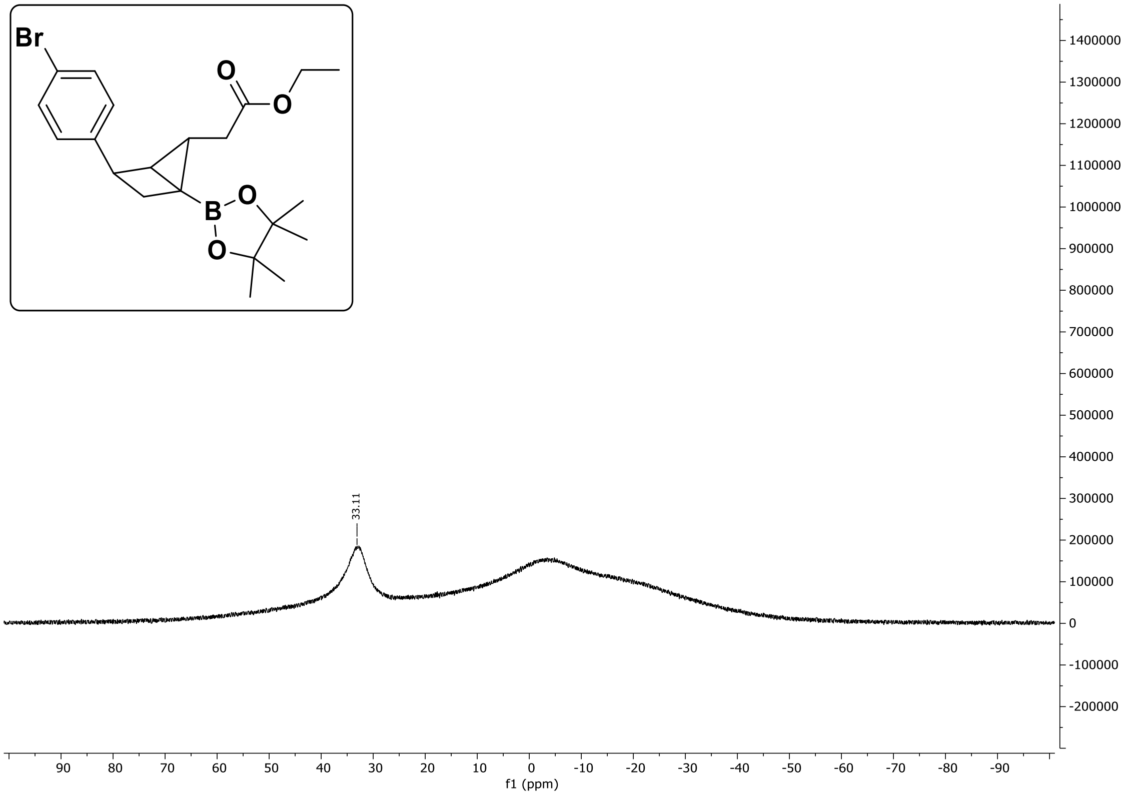

^{1}H-NMR of 36 (400 MHz, $CDCl_3$)

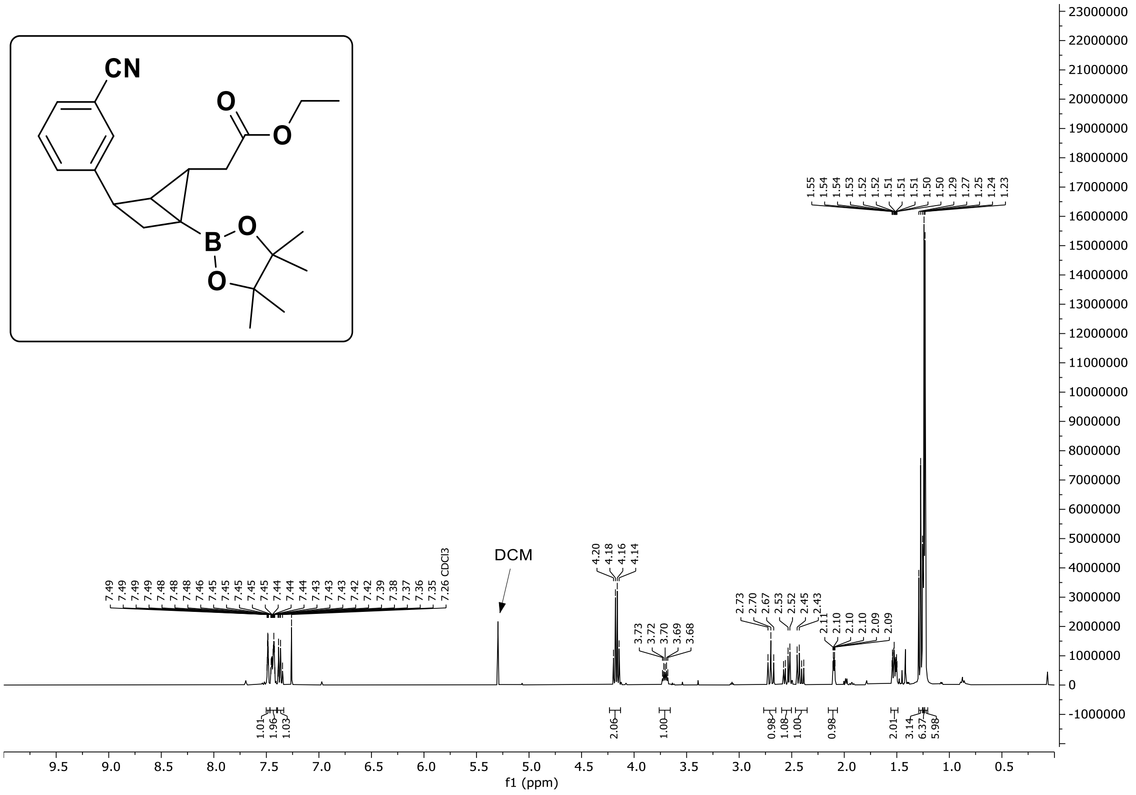

^{13}C-NMR of 36 (100 MHz, $CDCl_3$)

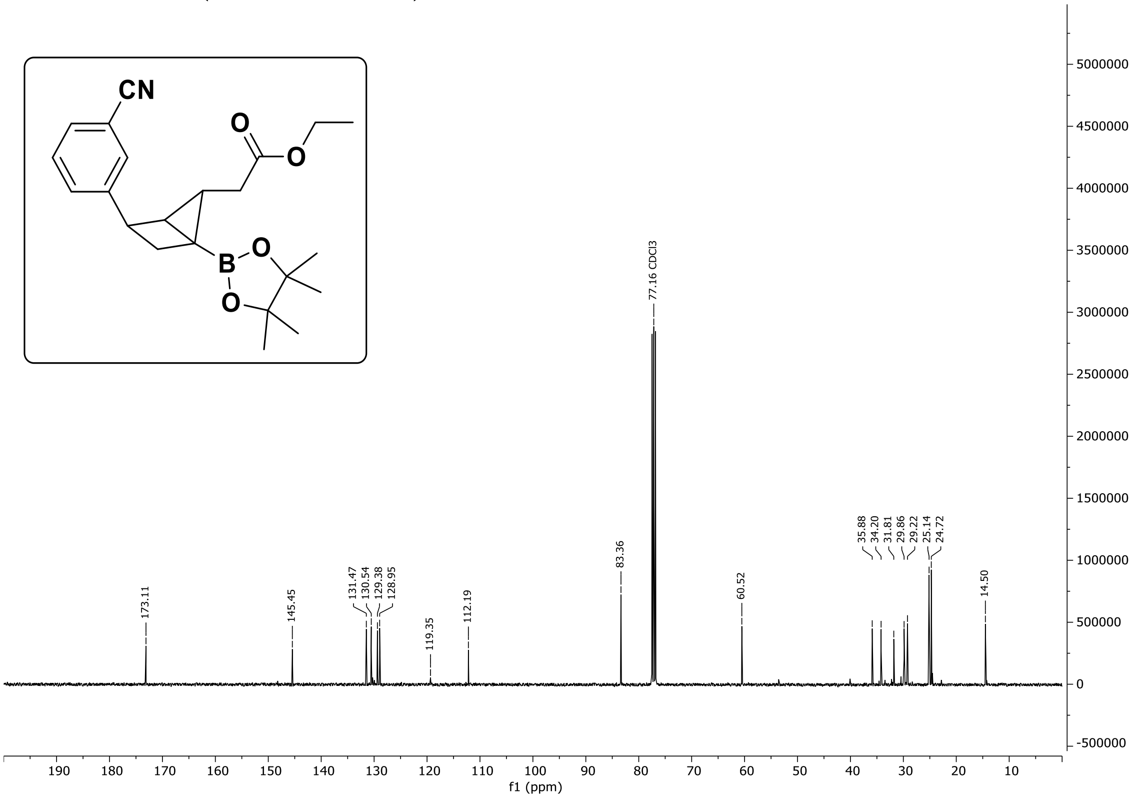

^{11}B NMR of 36 (128 MHz, $CDCl_3$)

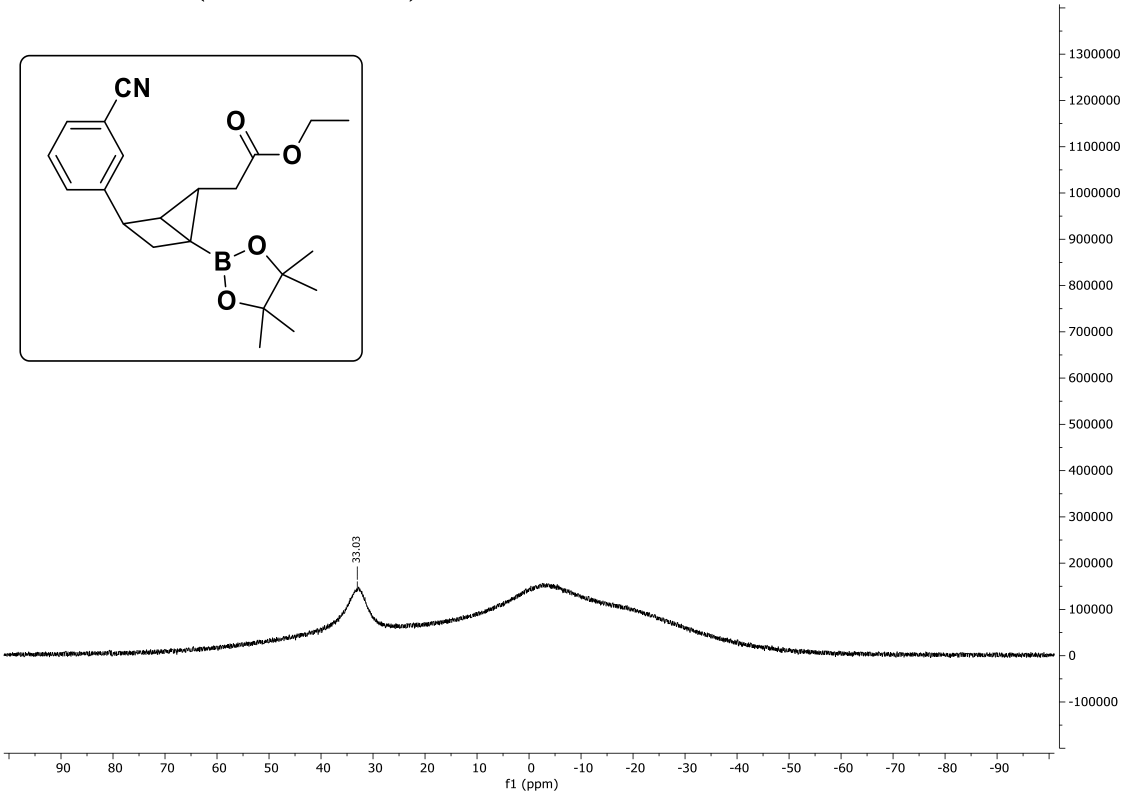

^{1}H-NMR of 37 (400 MHz, $CDCl_3$)

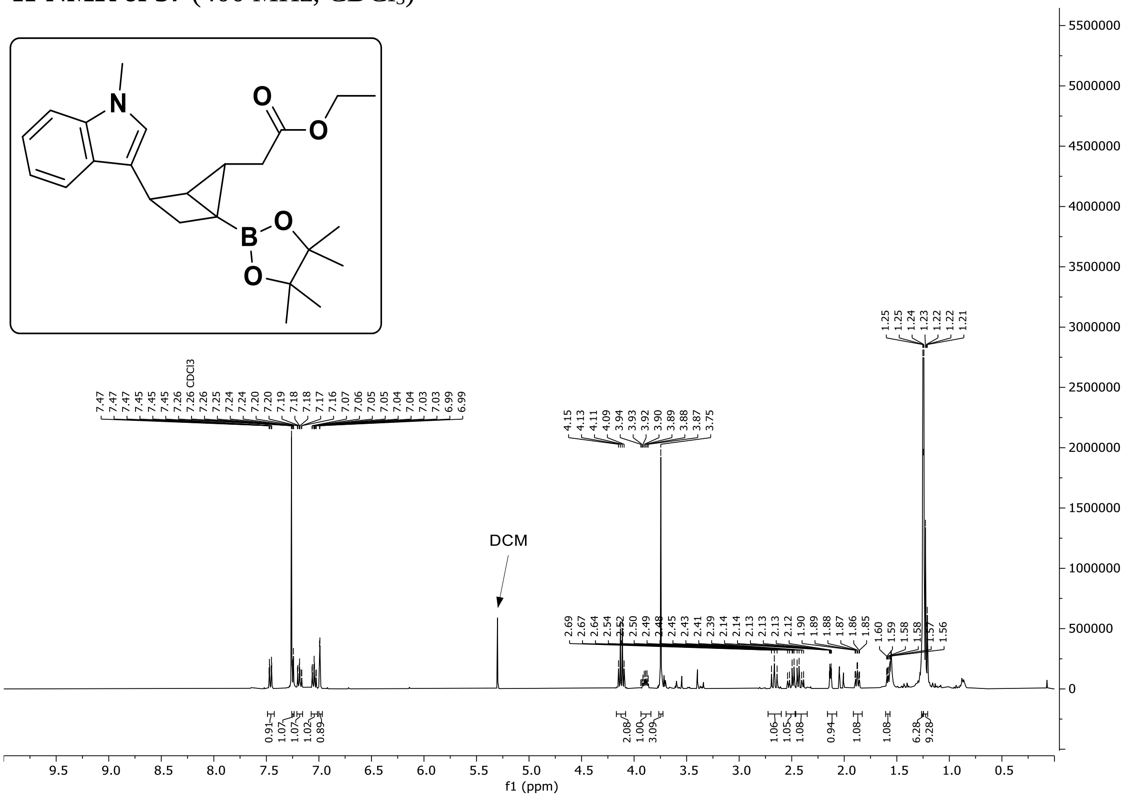

^{13}C-NMR of 37 (100 MHz, $CDCl_3$)

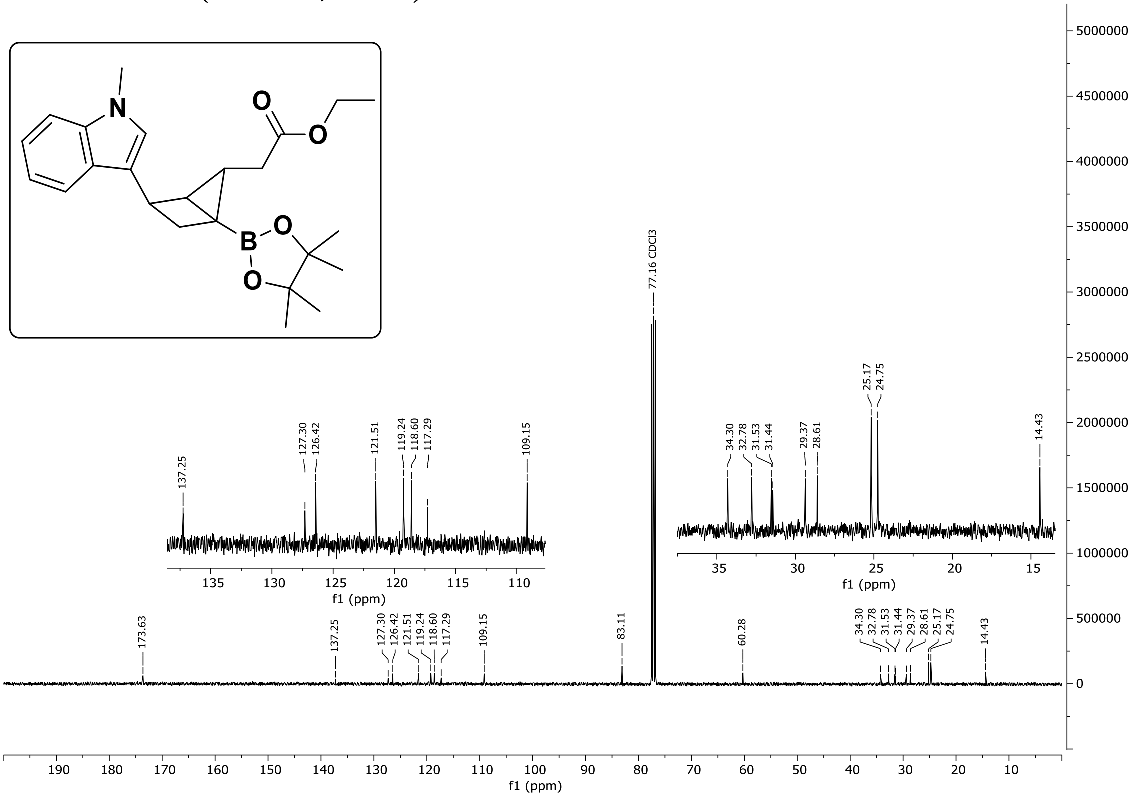

^{11}B NMR of 37 (128 MHz, $CDCl_3$)

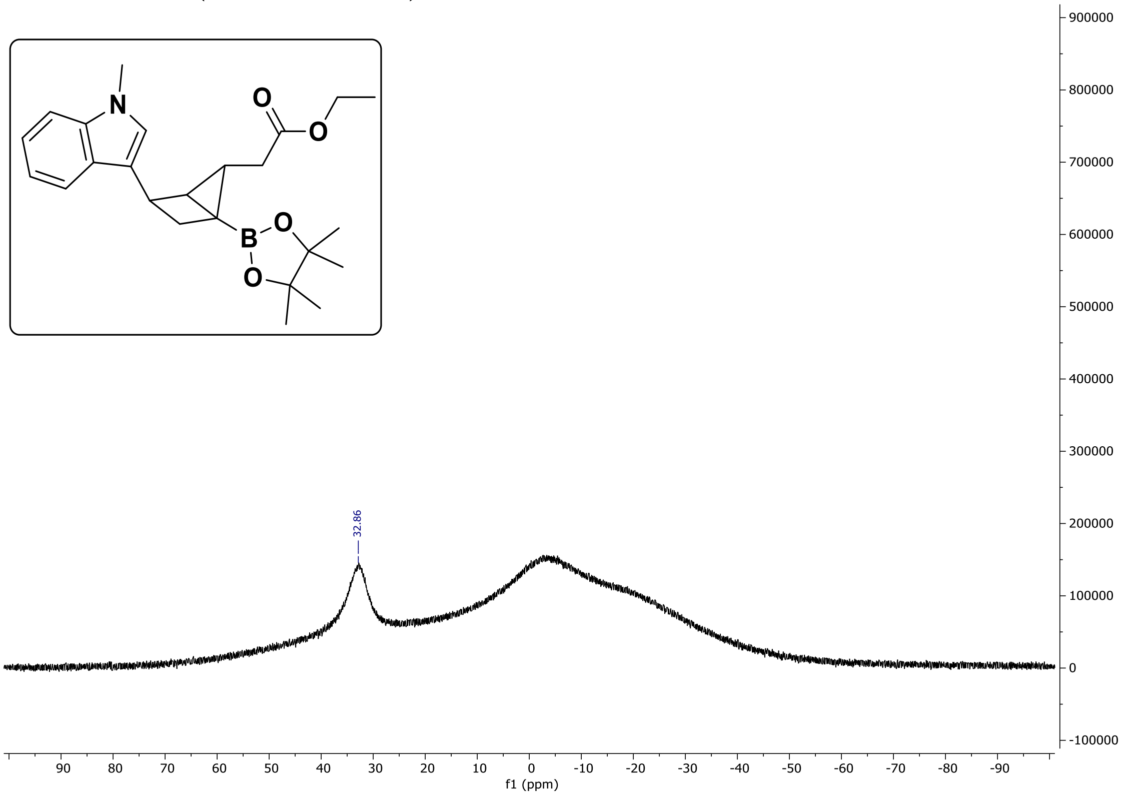

^{1}H-NMR of 38 (400 MHz, $CDCl_3$)

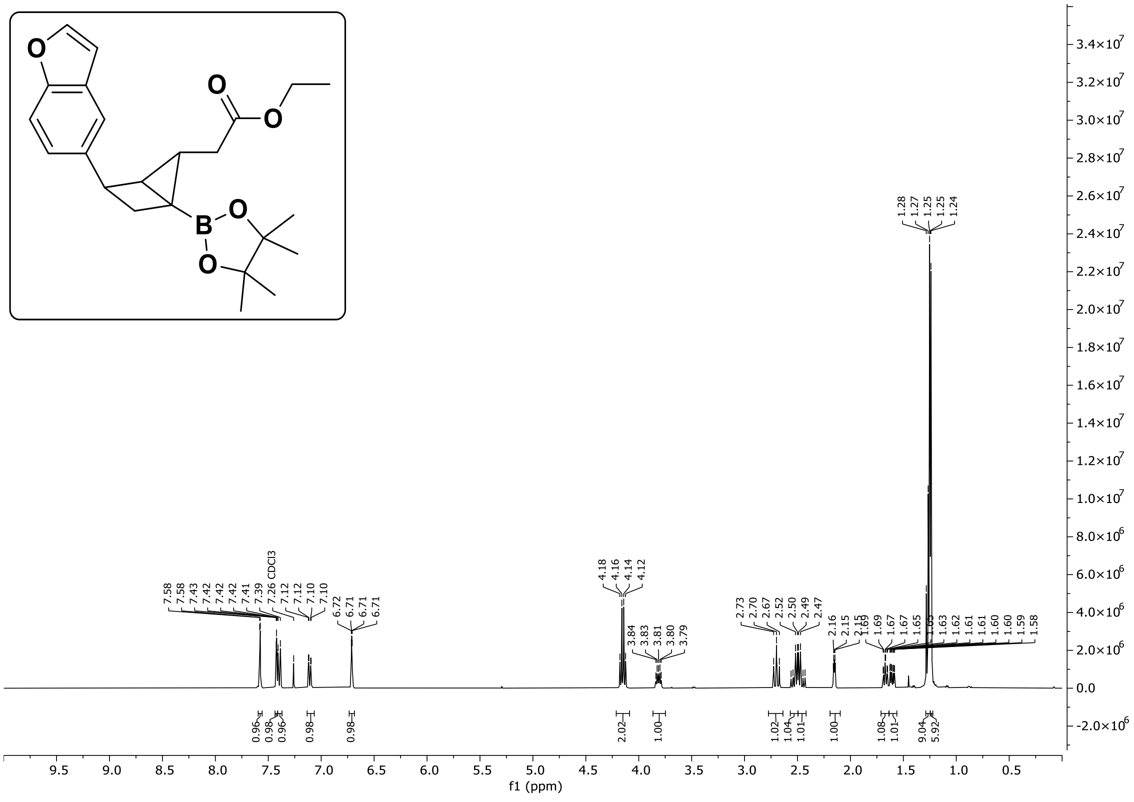

^{13}C-NMR of 38 (100 MHz, $CDCl_3$)

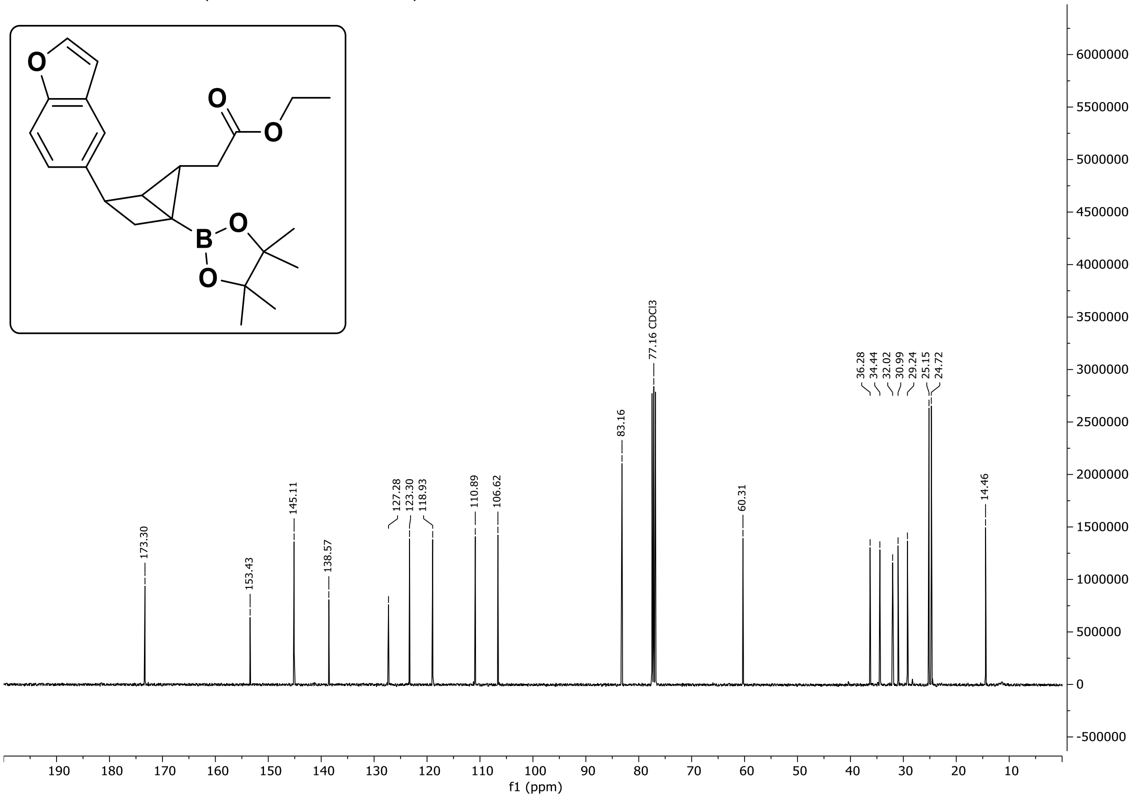

^{11}B NMR of 38 (128 MHz, $CDCl_3$)

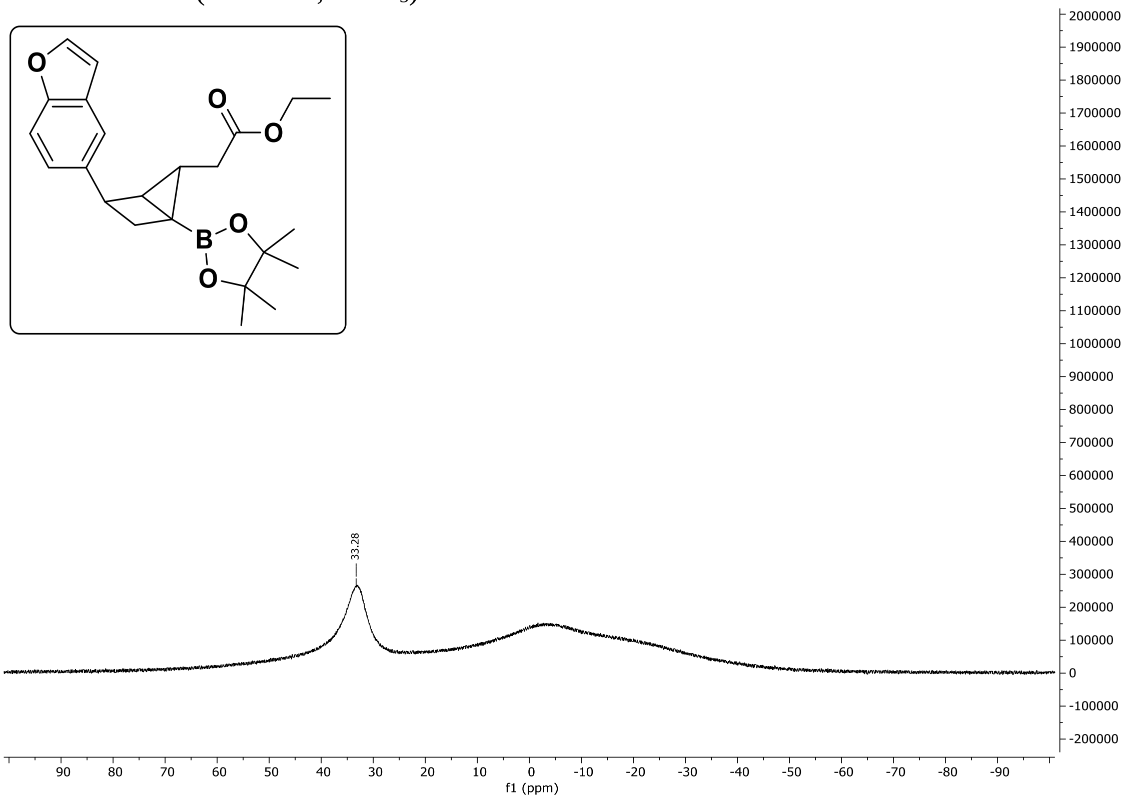

^{1}H-NMR of 39 (400 MHz, $CDCl_3$)

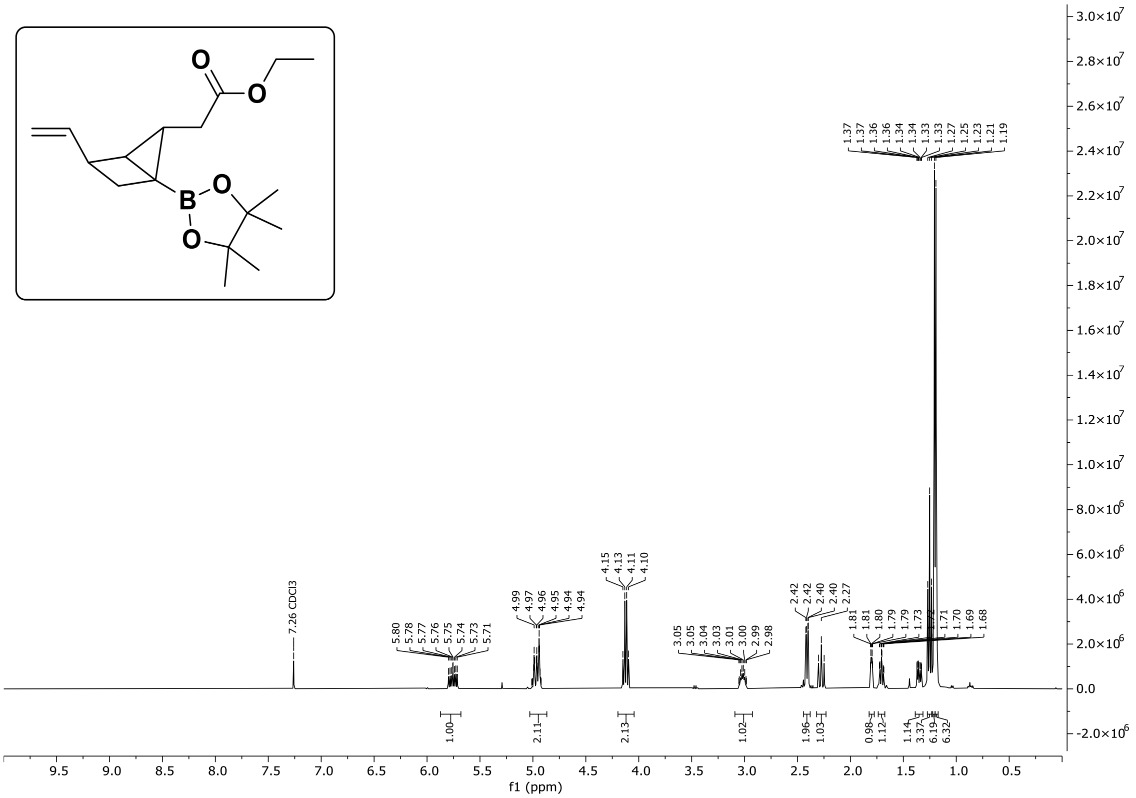

^{13}C-NMR of 39 (100 MHz, $CDCl_3$)

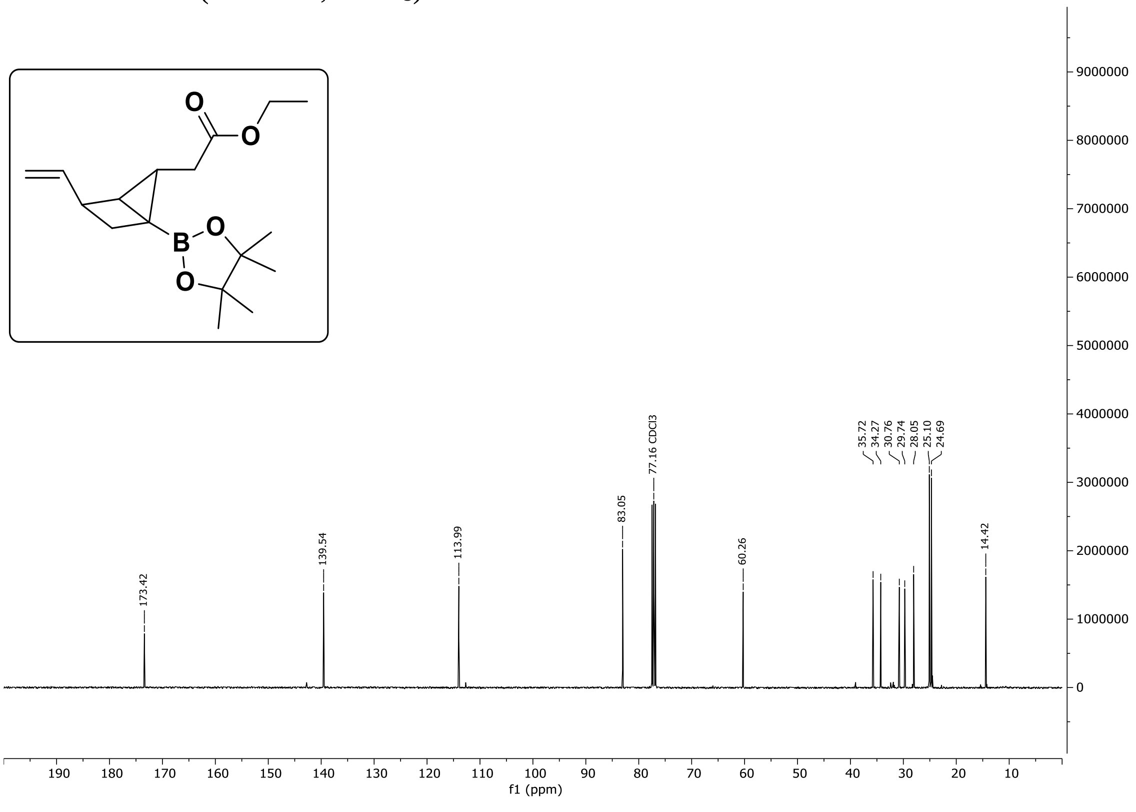

^{11}B NMR of 39 (128 MHz, $CDCl_3$)

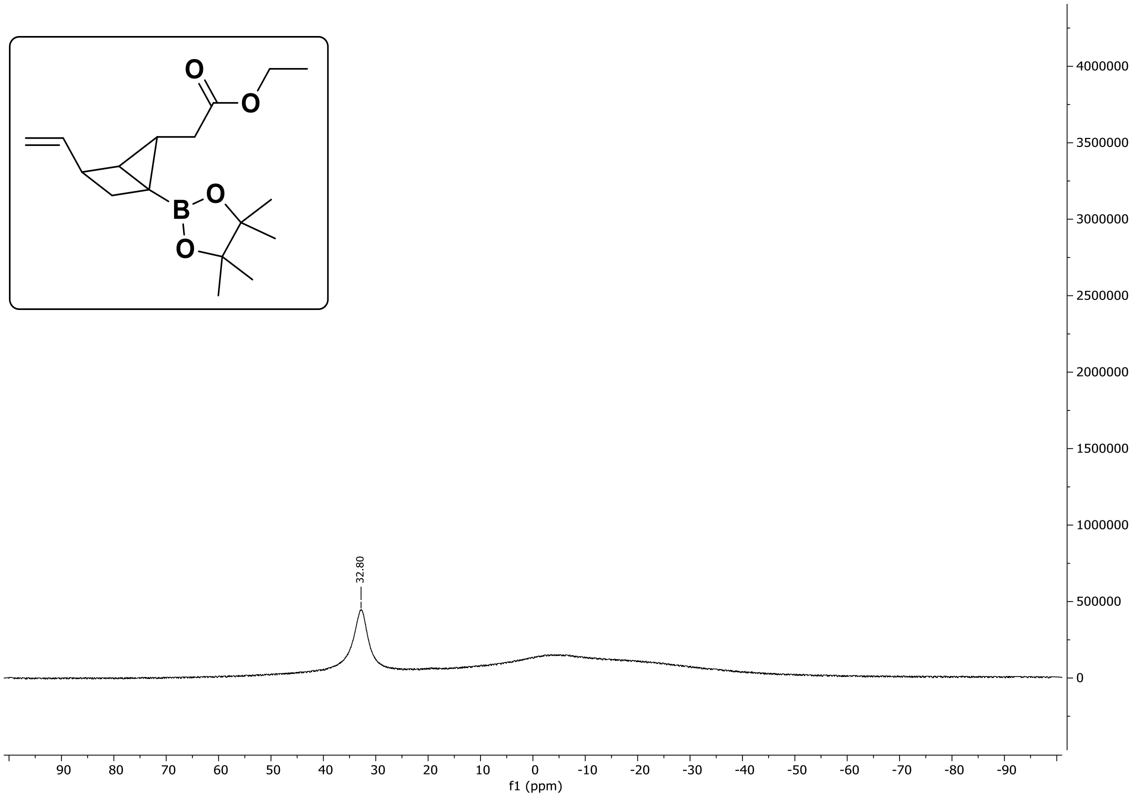

^{1}H-NMR of 40 (400 MHz, $CDCl_3$)

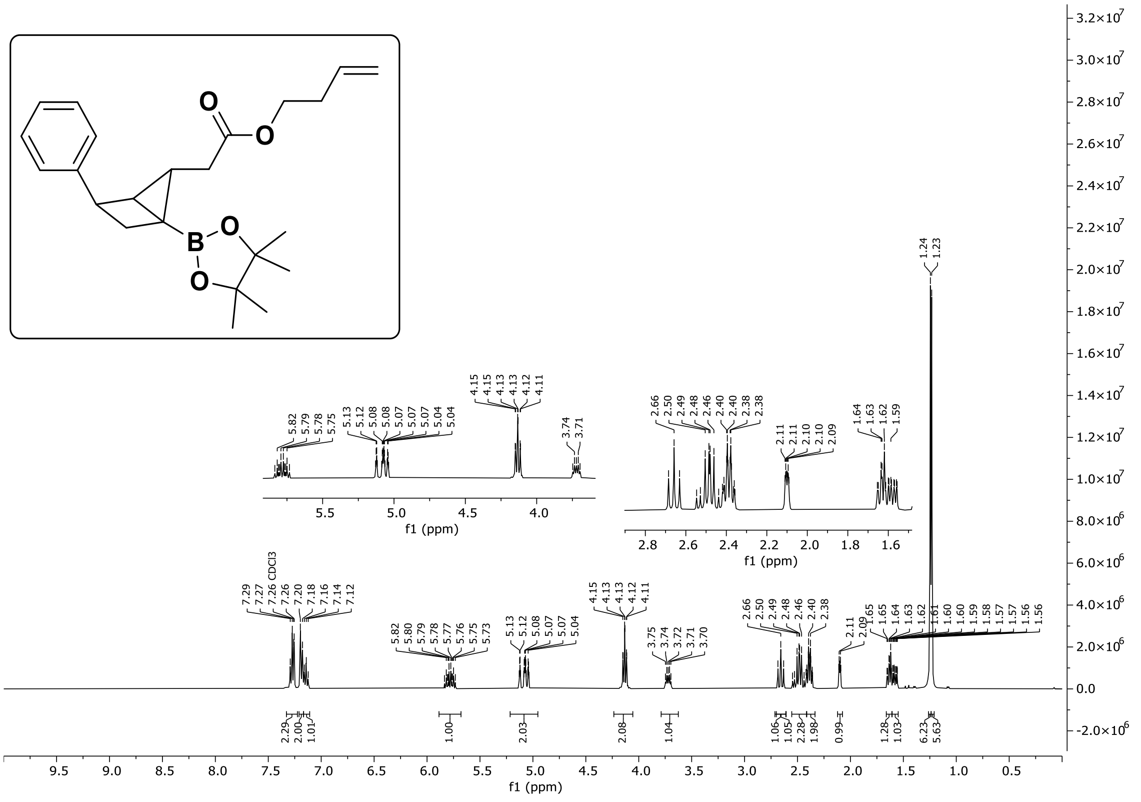

^{13}C-NMR of 40 (100 MHz, $CDCl_3$)

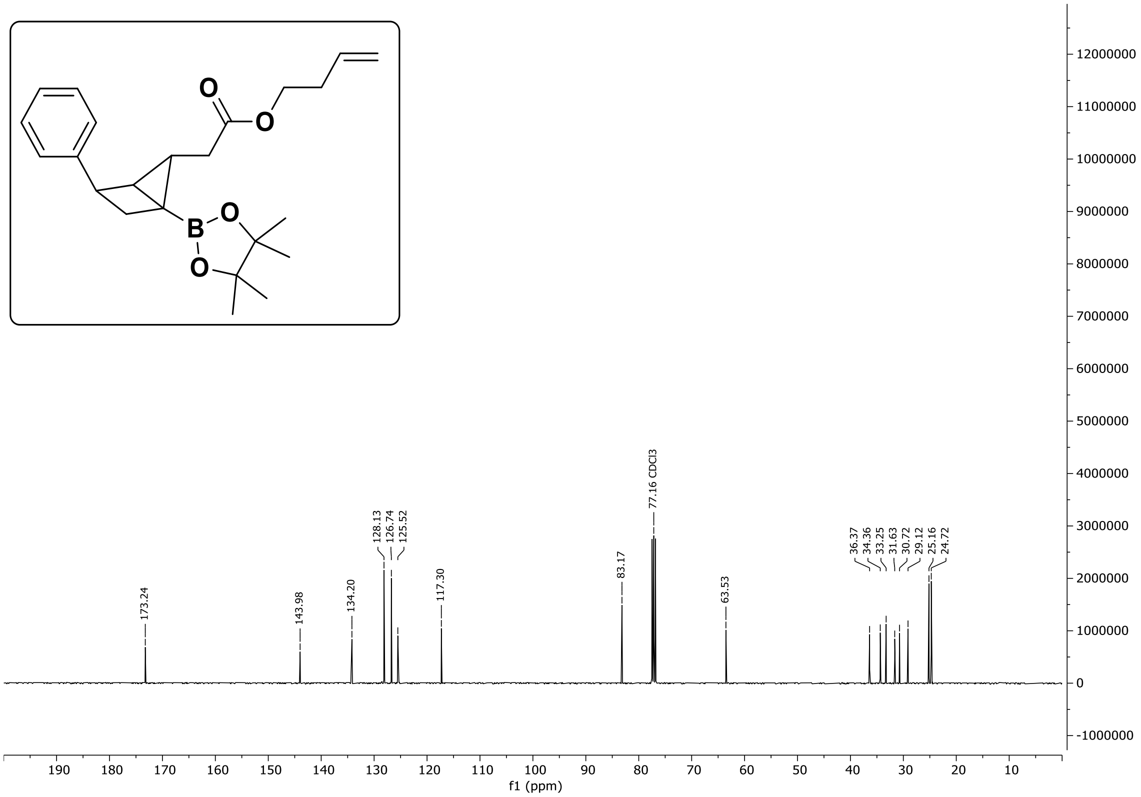

^{11}B NMR of 40 (128 MHz, $CDCl_3$)

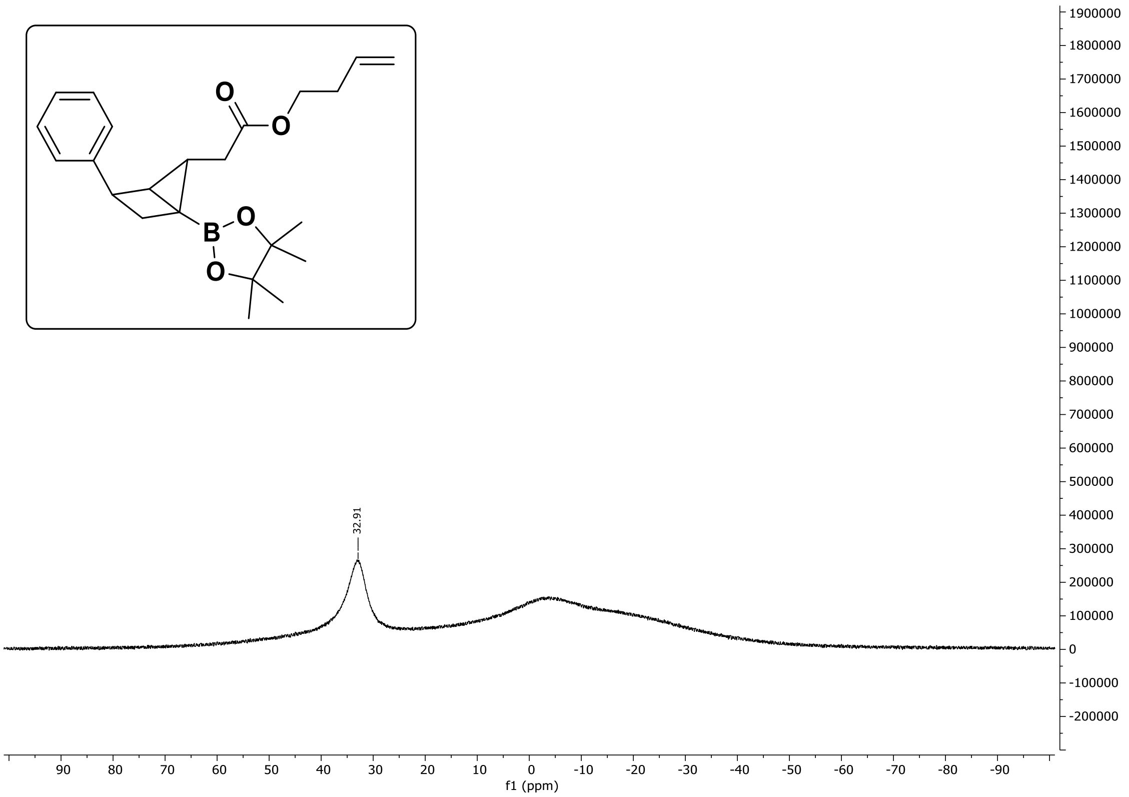

^{1}H-NMR of 41 (400 MHz, $CDCl_3$)

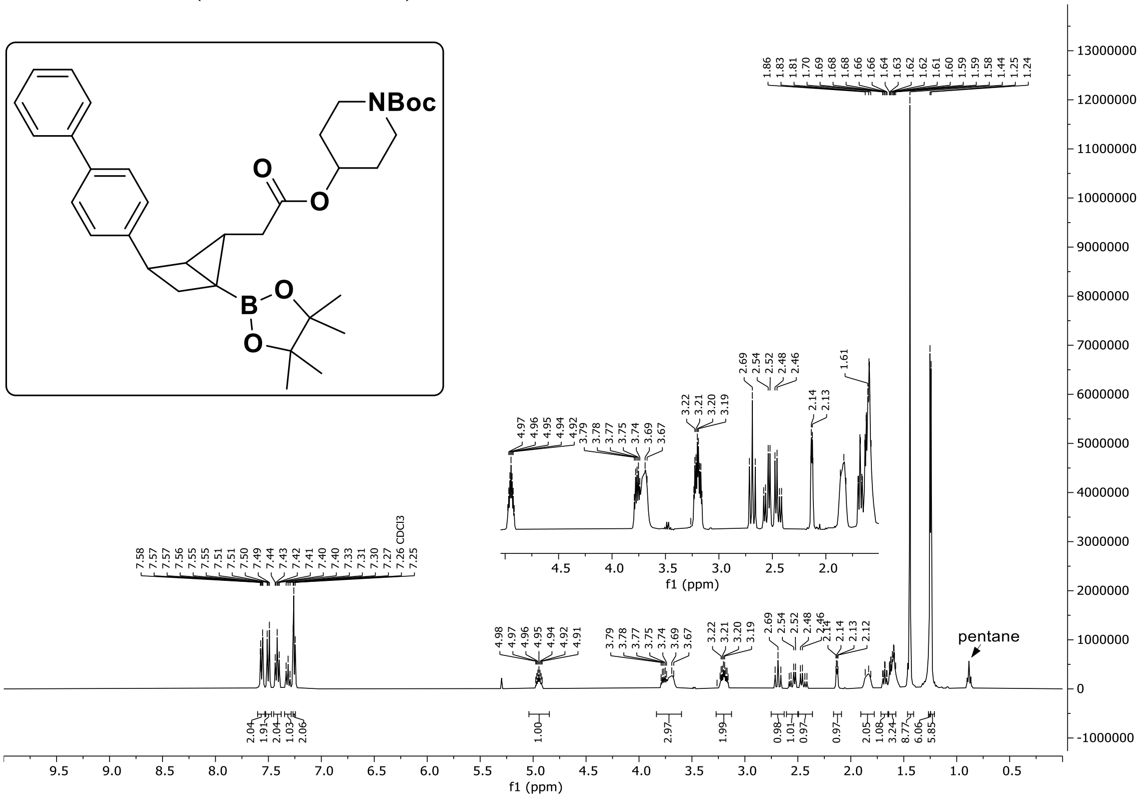

^{13}C-NMR of 41 (100 MHz, $CDCl_3$)

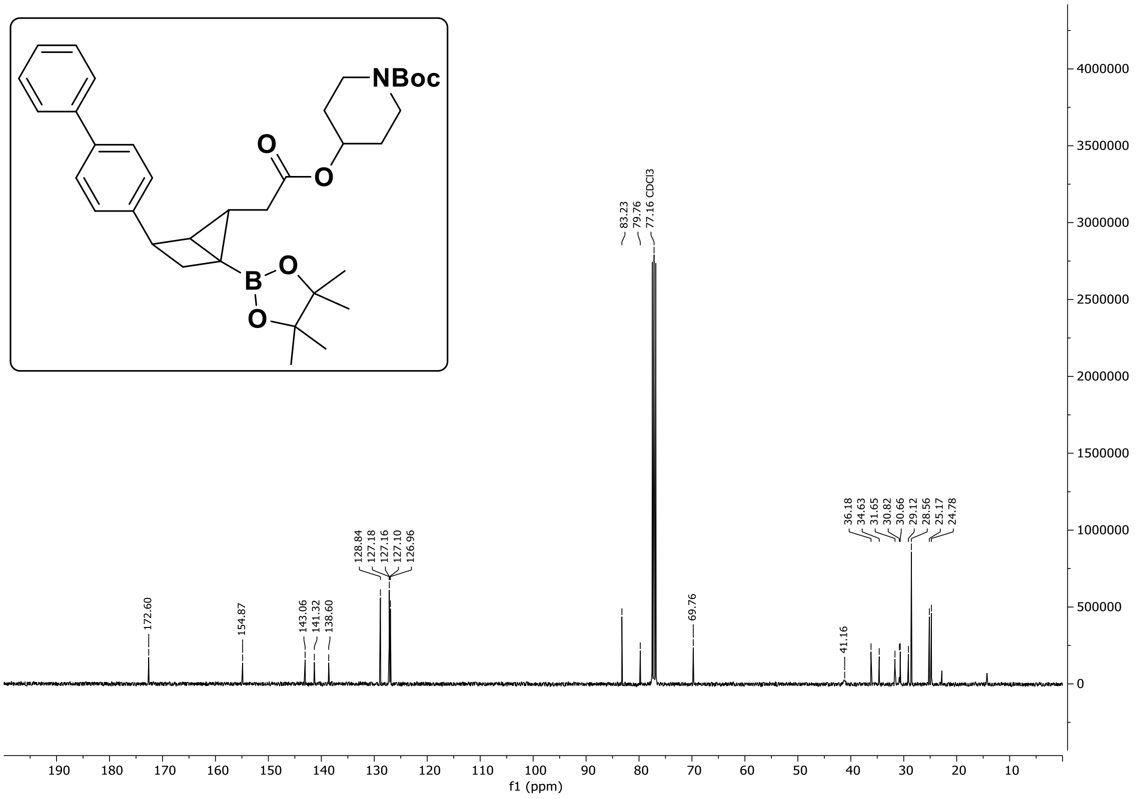

^{11}B NMR of 41 (128 MHz, $CDCl_3$)

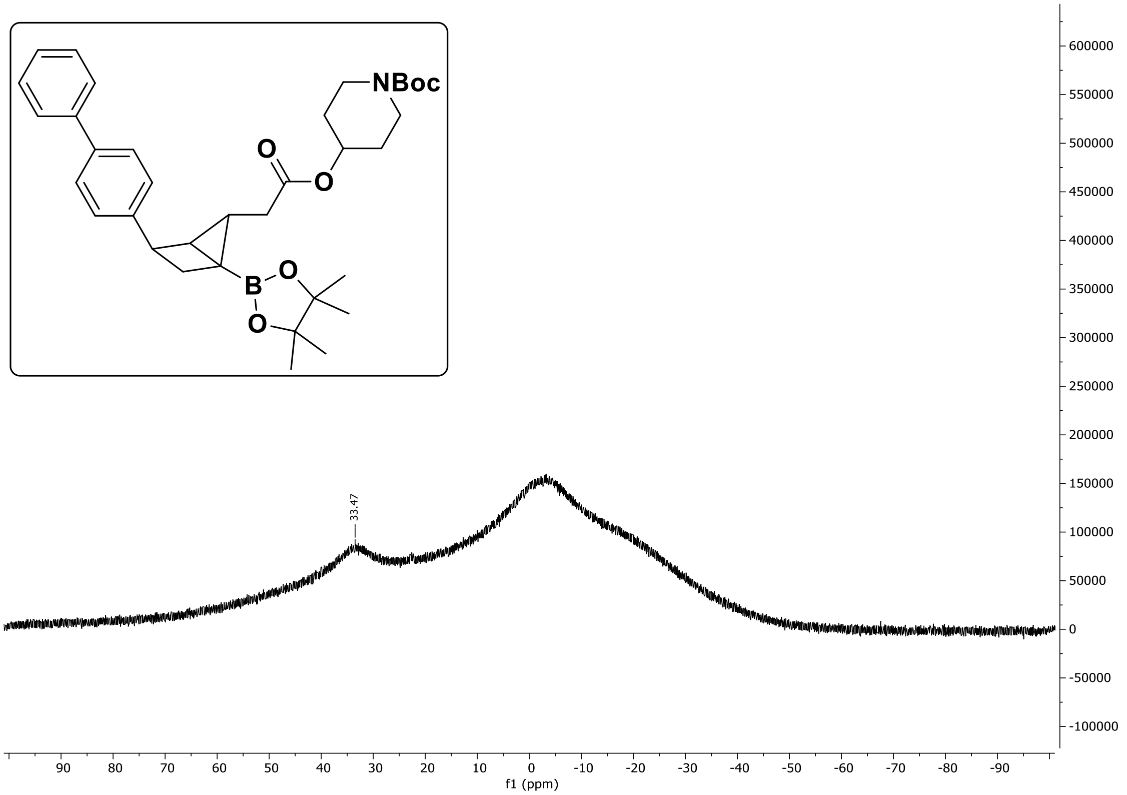

^{1}H-NMR of 42 (400 MHz, $CDCl_3$)

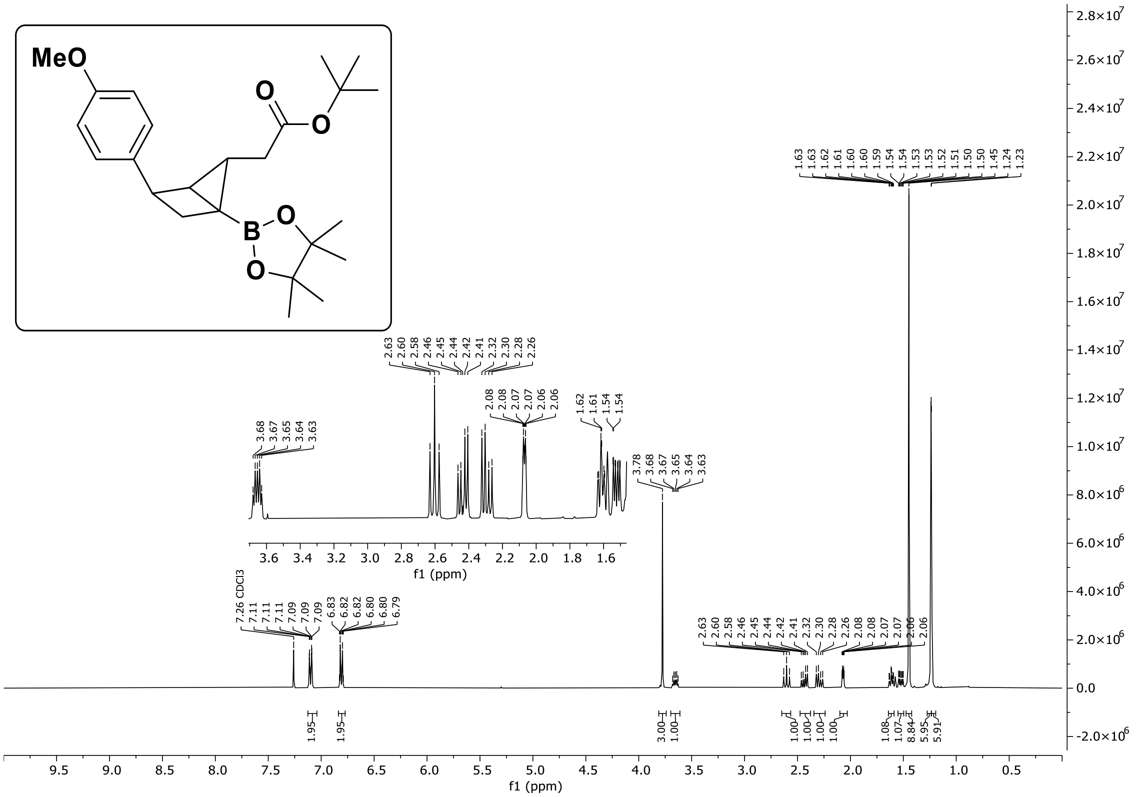

^{13}C-NMR of 42 (100 MHz, $CDCl_3$)

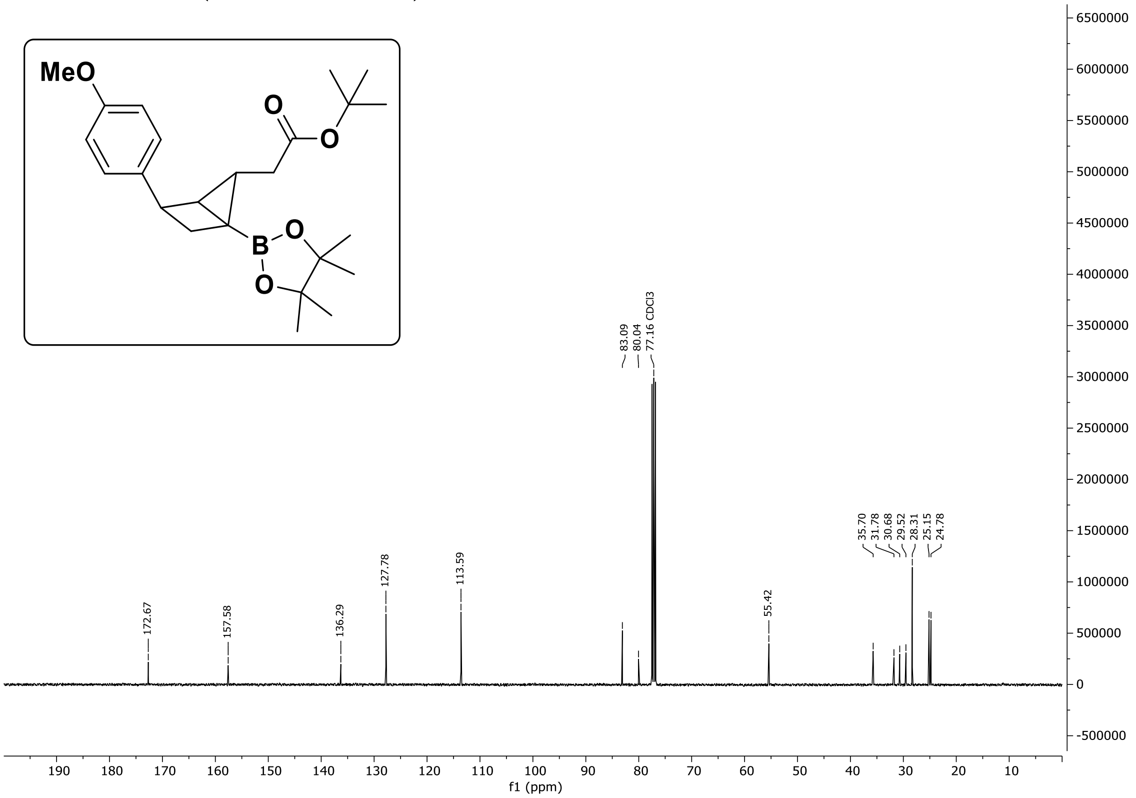

^{11}B NMR of 42 (128 MHz, $CDCl_3$)

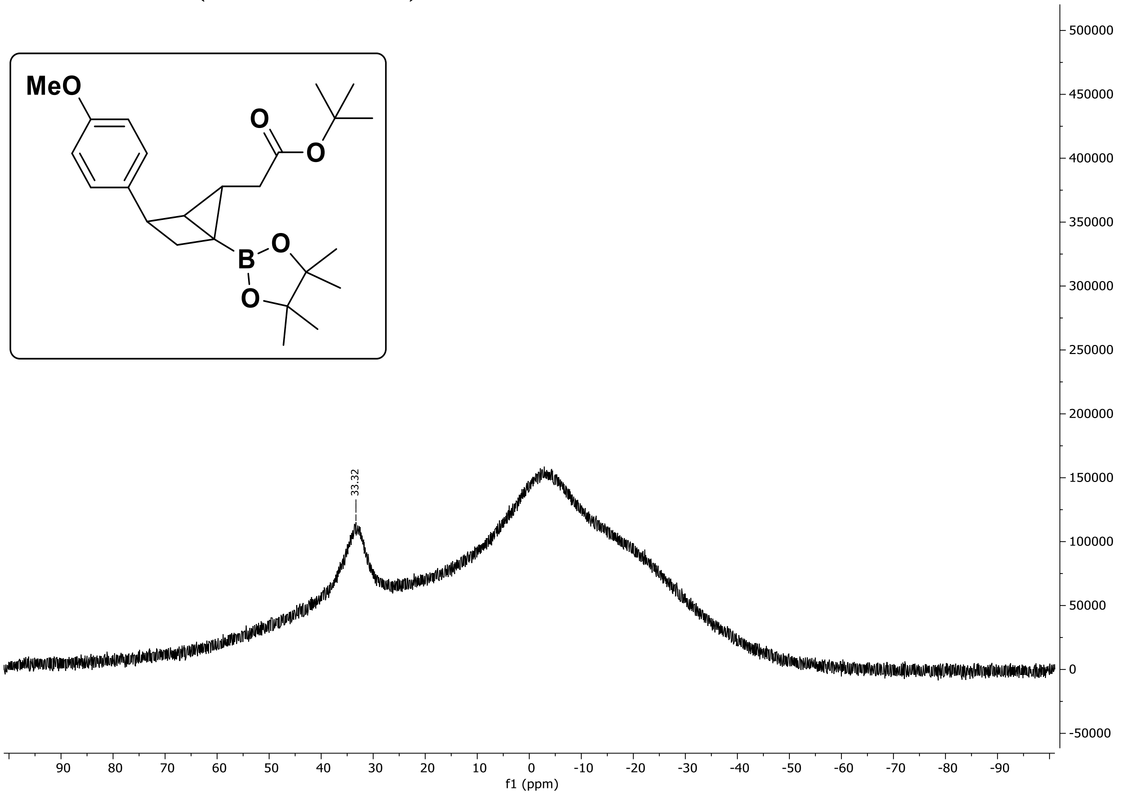

^{1}H-NMR of 43 (400 MHz, $CDCl_3$)

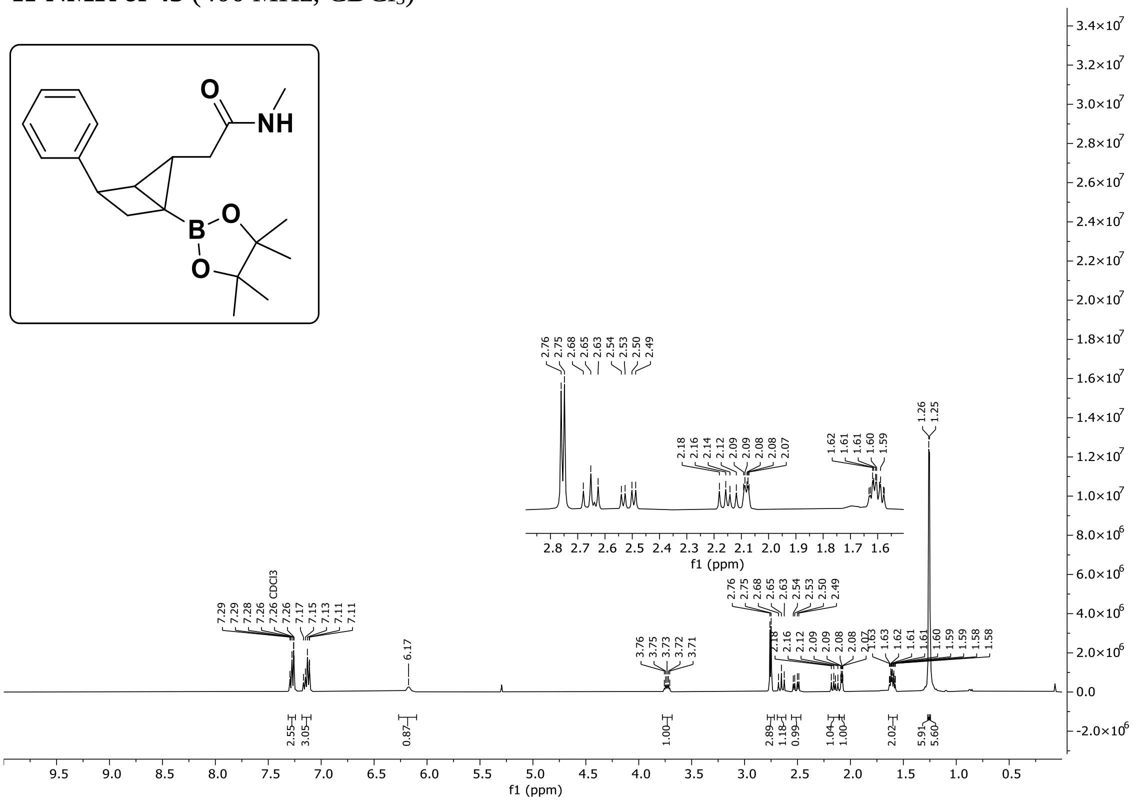

^{13}C-NMR of 43 (100 MHz, $CDCl_3$)

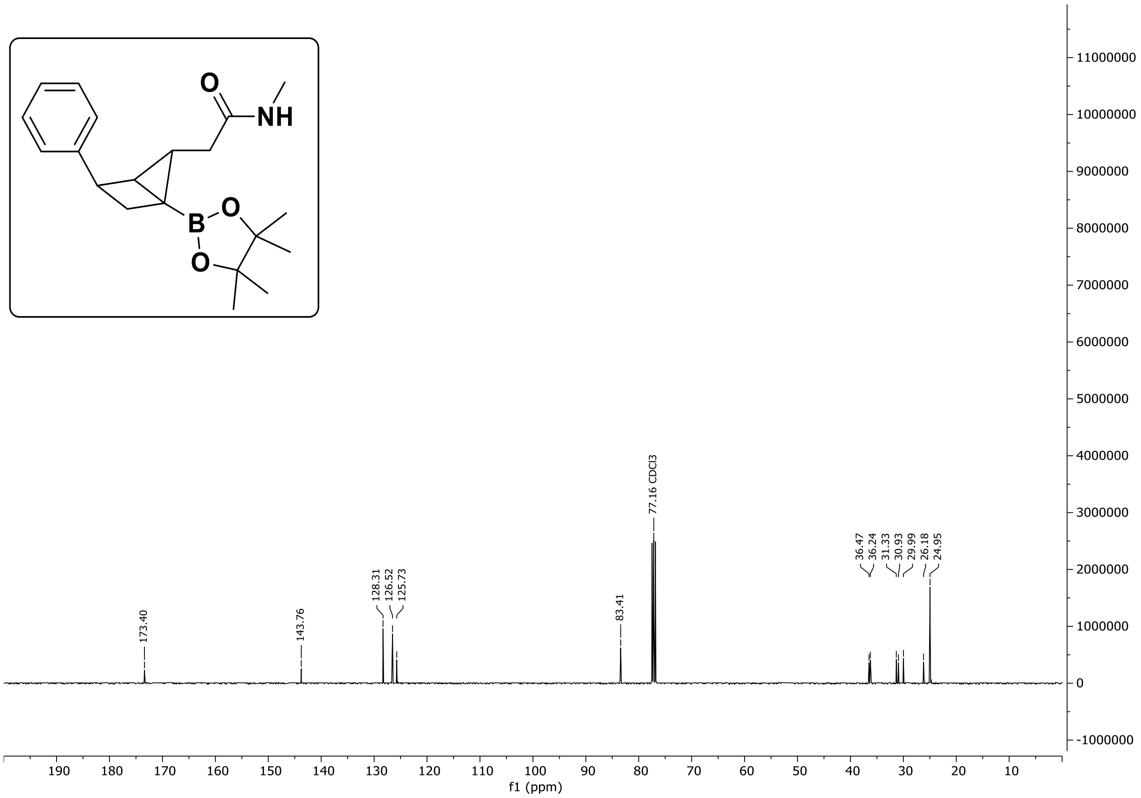

^{11}B NMR of 43 (128 MHz, $CDCl_3$)

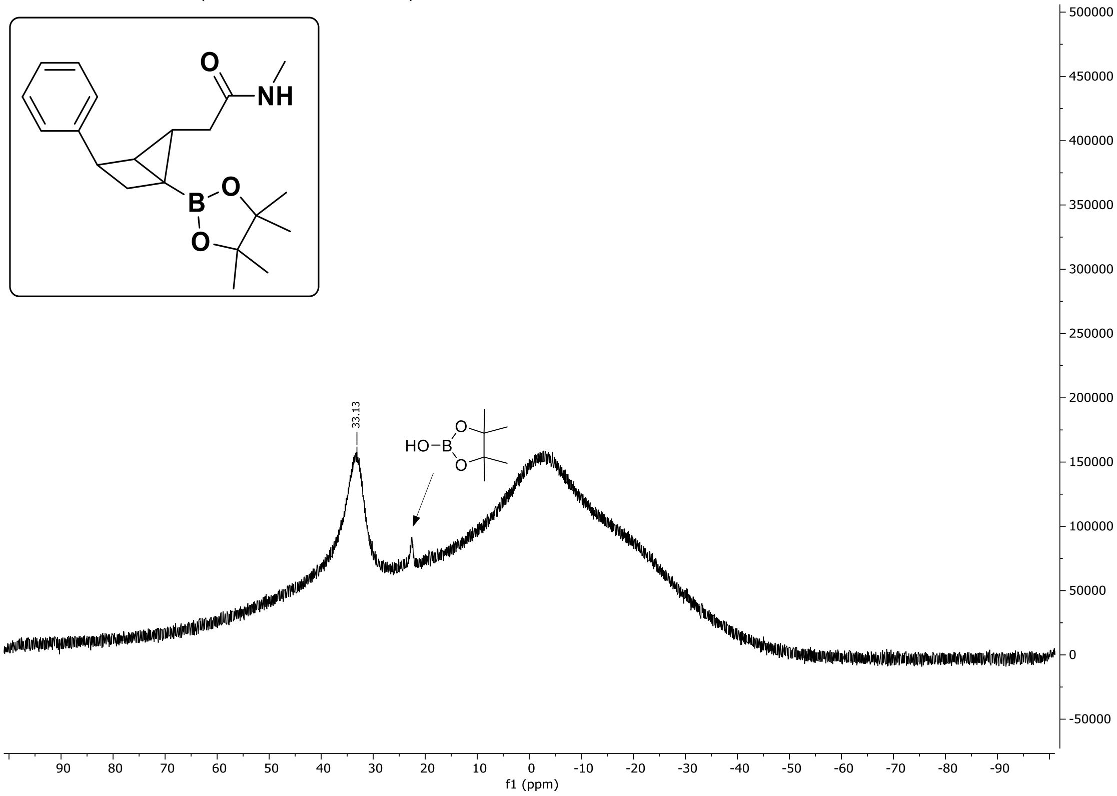

^{1}H-NMR of 44 (400 MHz, $CDCl_3$)

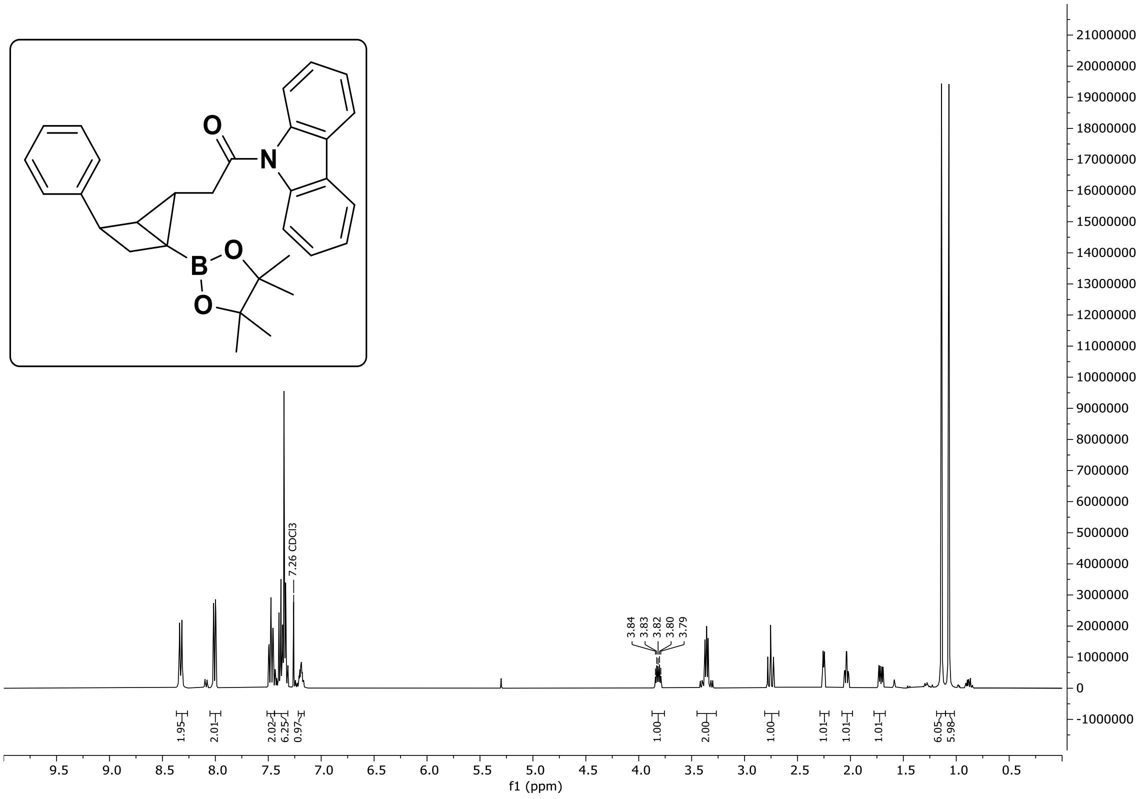

^{13}C-NMR of 44 (100 MHz, $CDCl_3$)

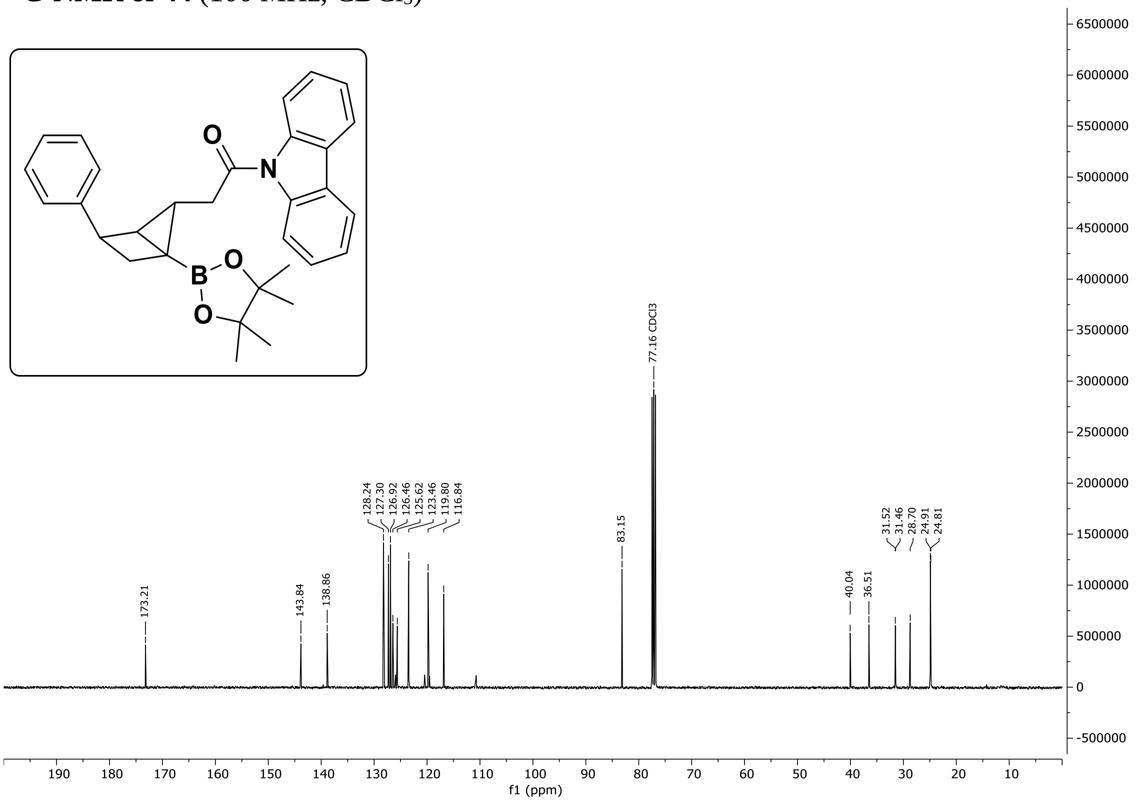

^{11}B NMR of 44 (128 MHz, $CDCl_3$)

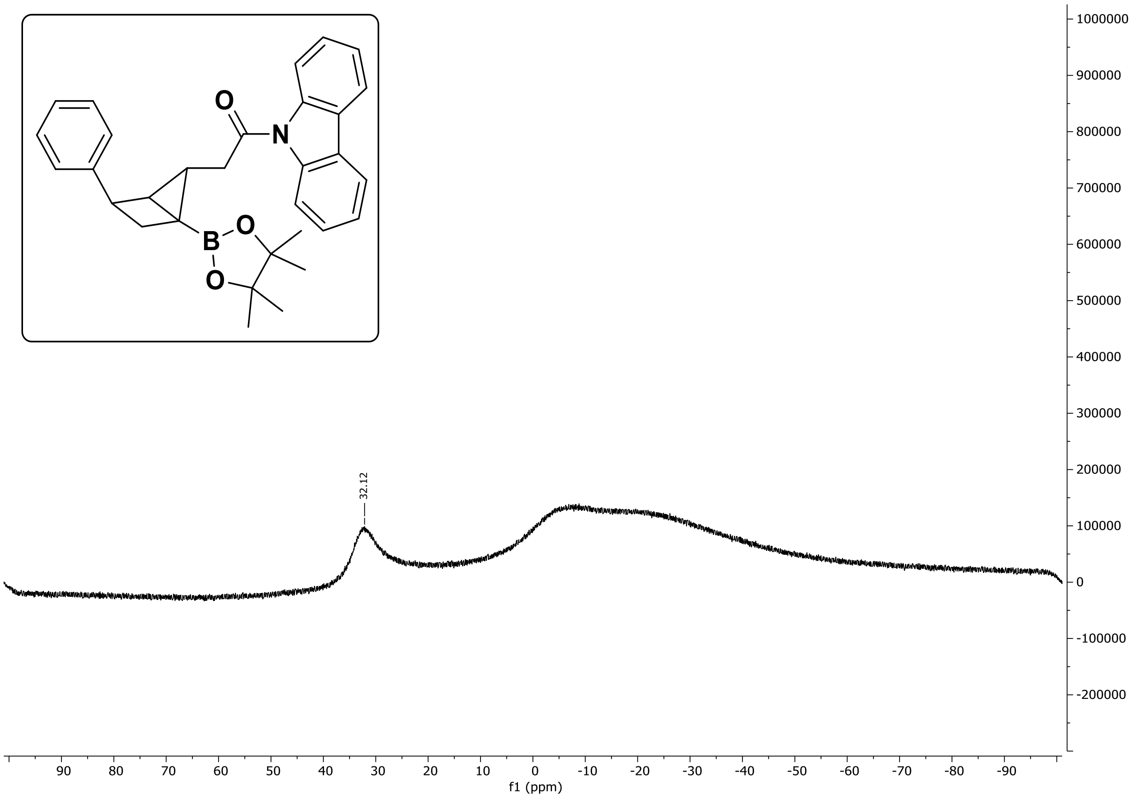

^{1}H-NMR of 45 (400 MHz, $CDCl_3$)

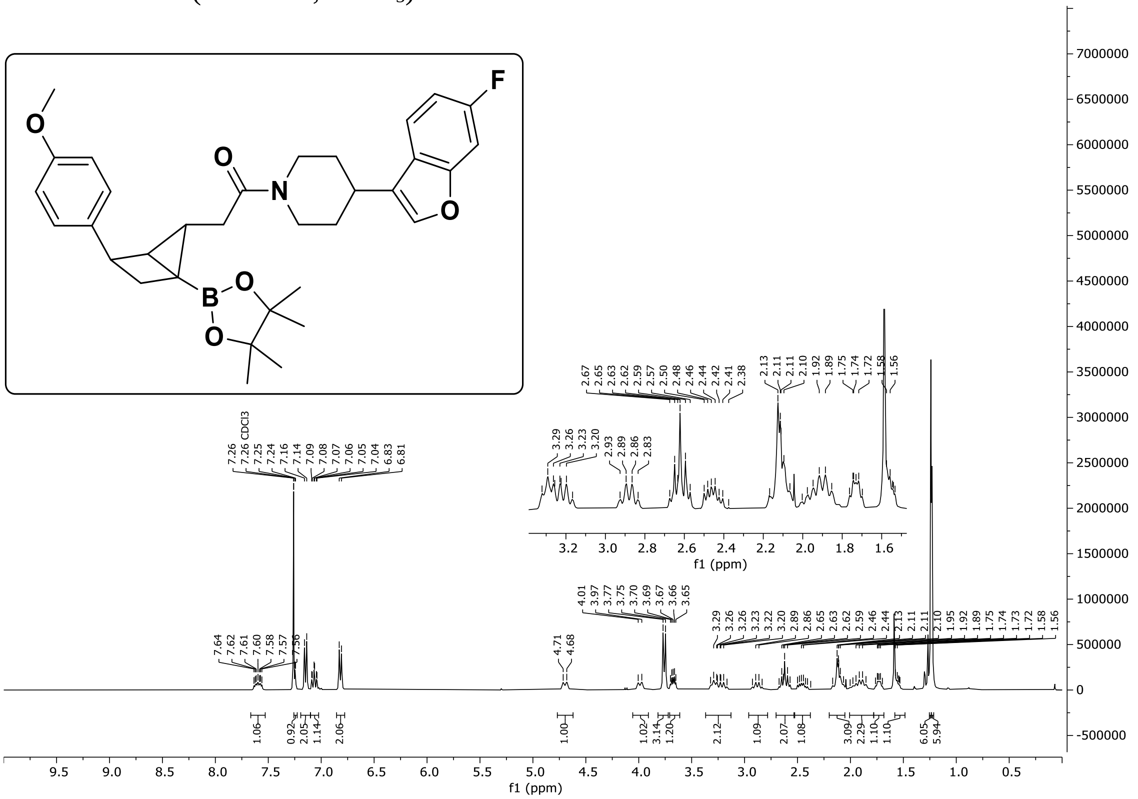

^{13}C-NMR of 45 (151 MHz, $CDCl_3$)

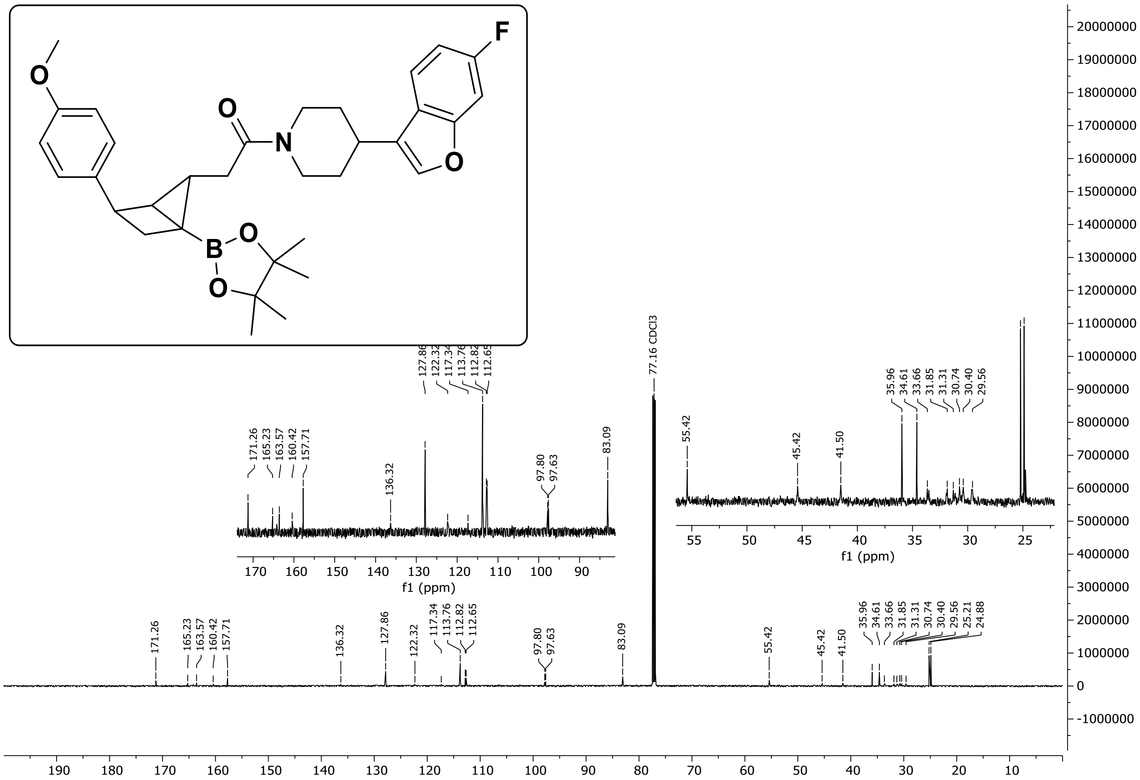

^{19}F-NMR of 45 (376 MHz, $CDCl_3$)

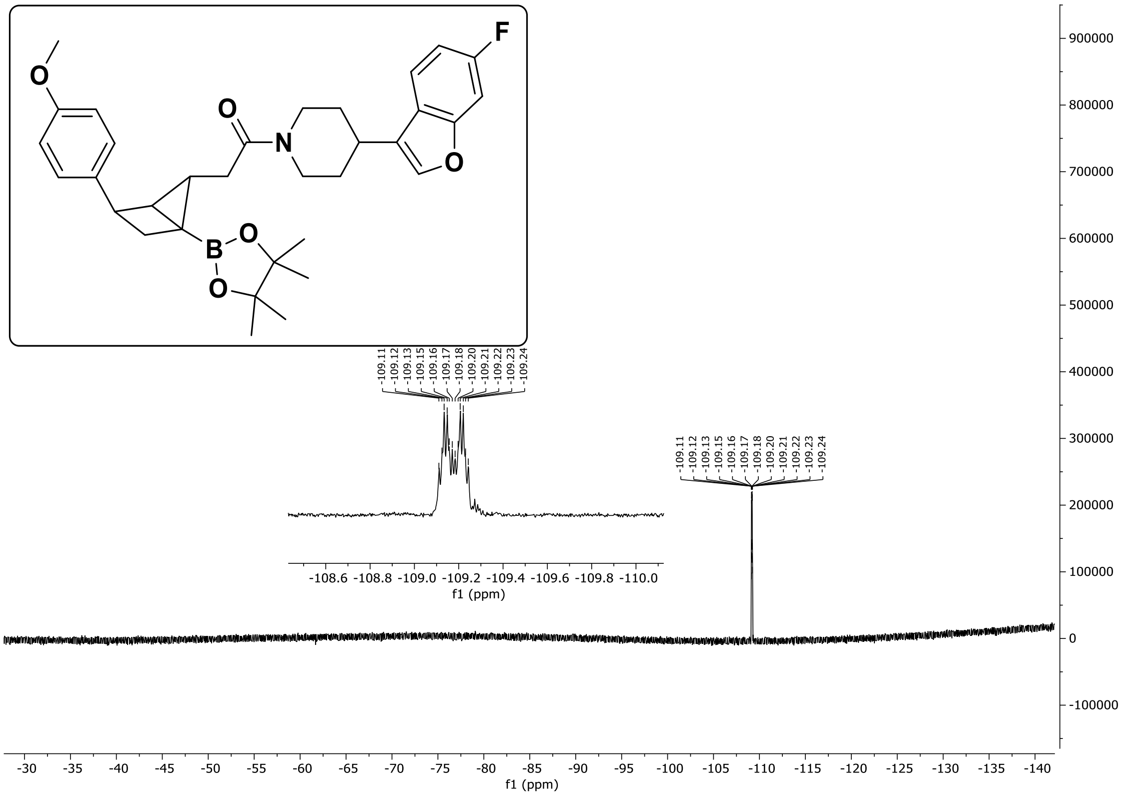

y

^{11}B NMR of 45 (128 MHz, $CDCl_3$)

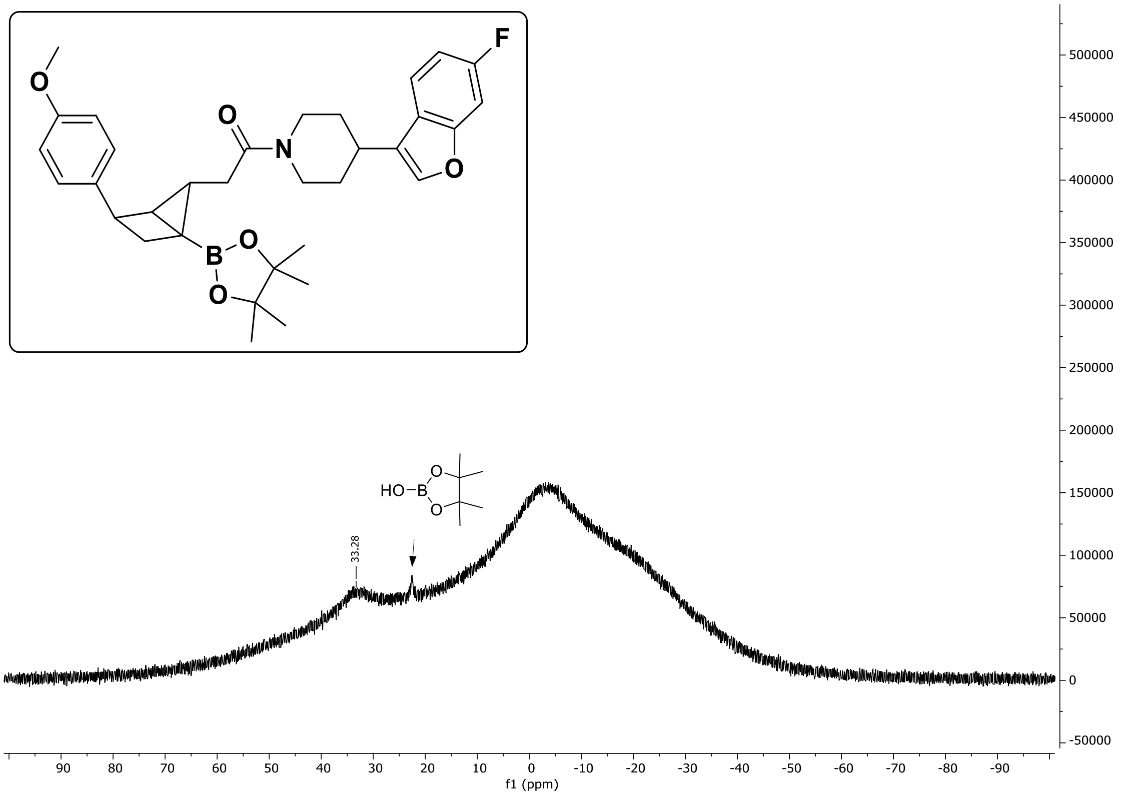

^{1}H-NMR of 46 (400 MHz, $CDCl_3$)

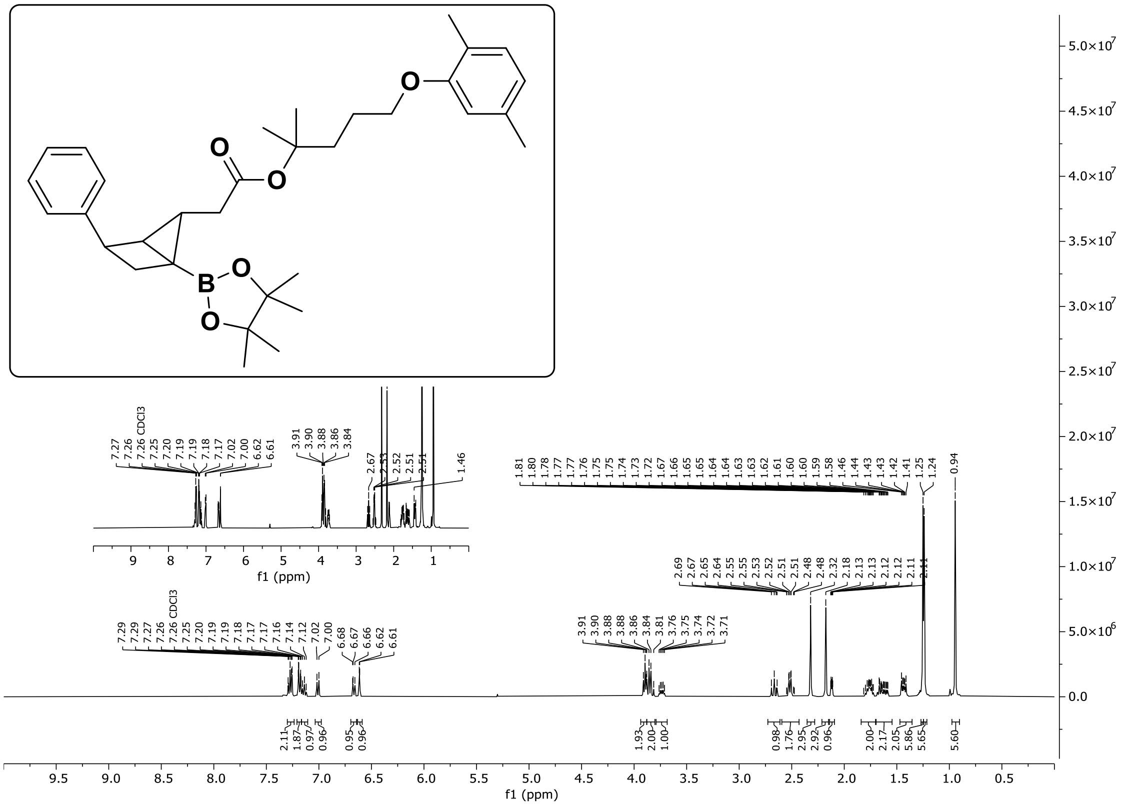

^{13}C-NMR of 46 (100 MHz, $CDCl_3$)

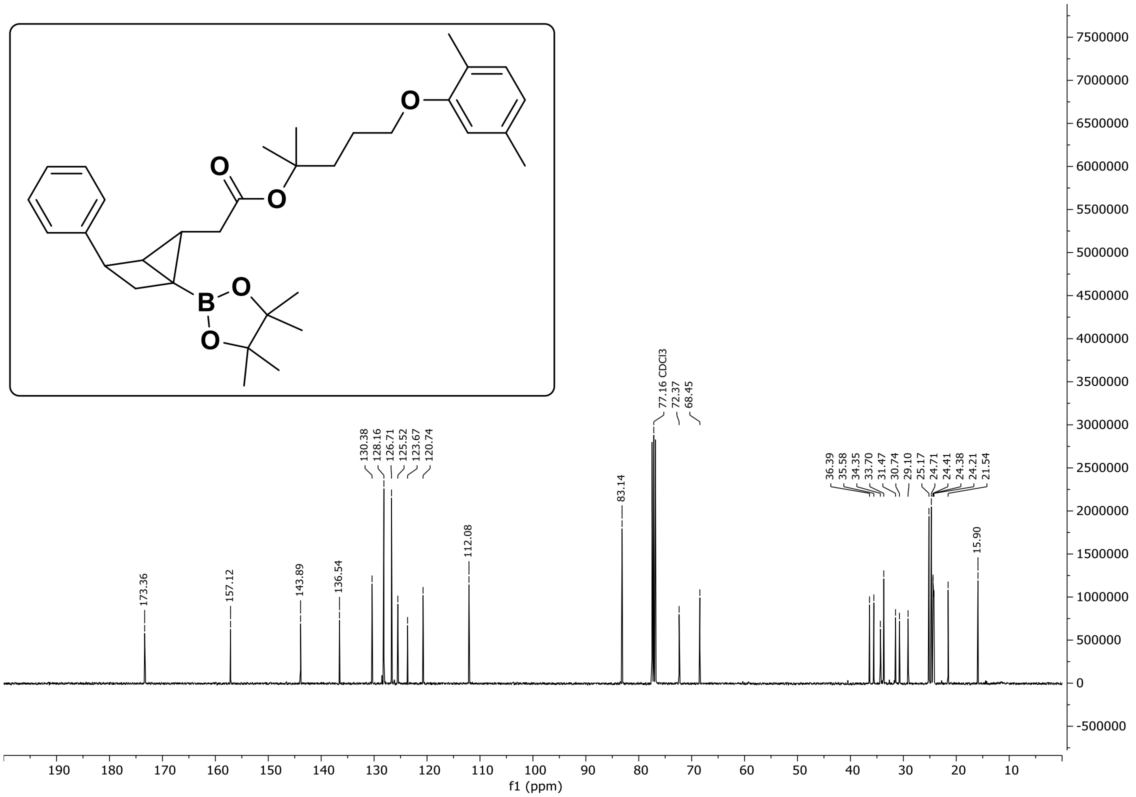

¹¹B NMR of 46 (128 MHz, $CDCl_3$)

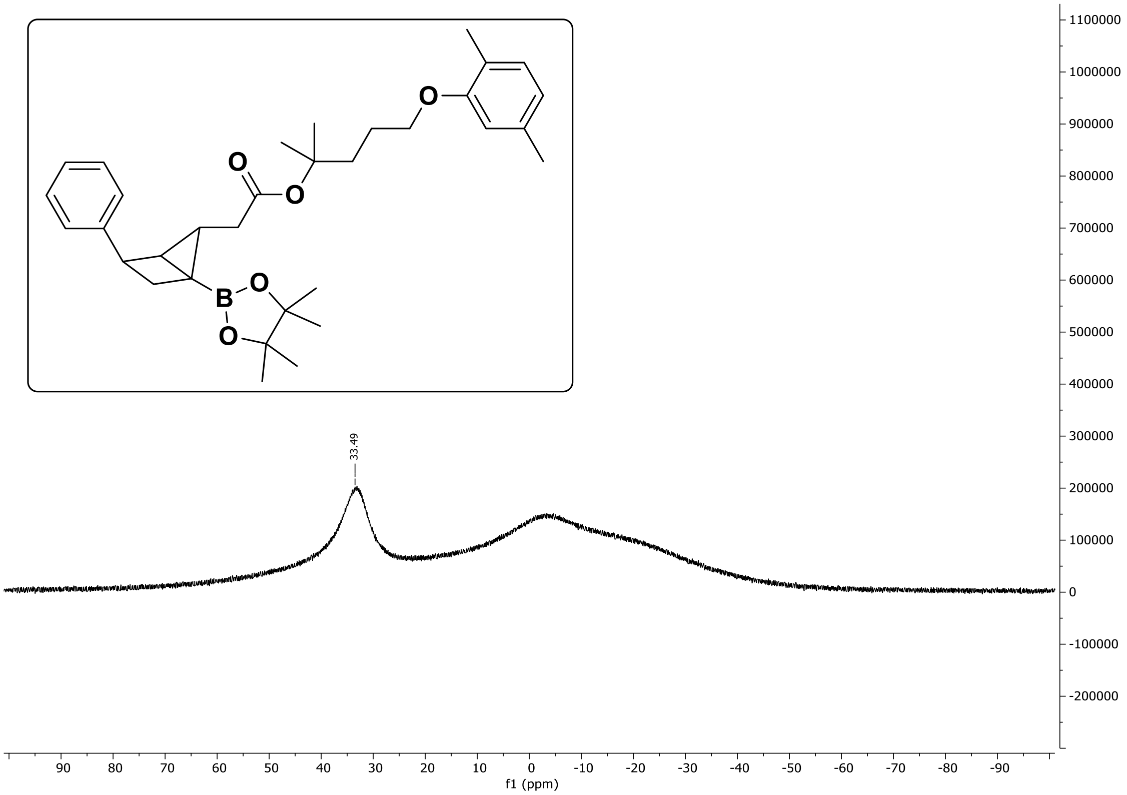

¹H-NMR of 47 (400 MHz, $CDCl_3$)

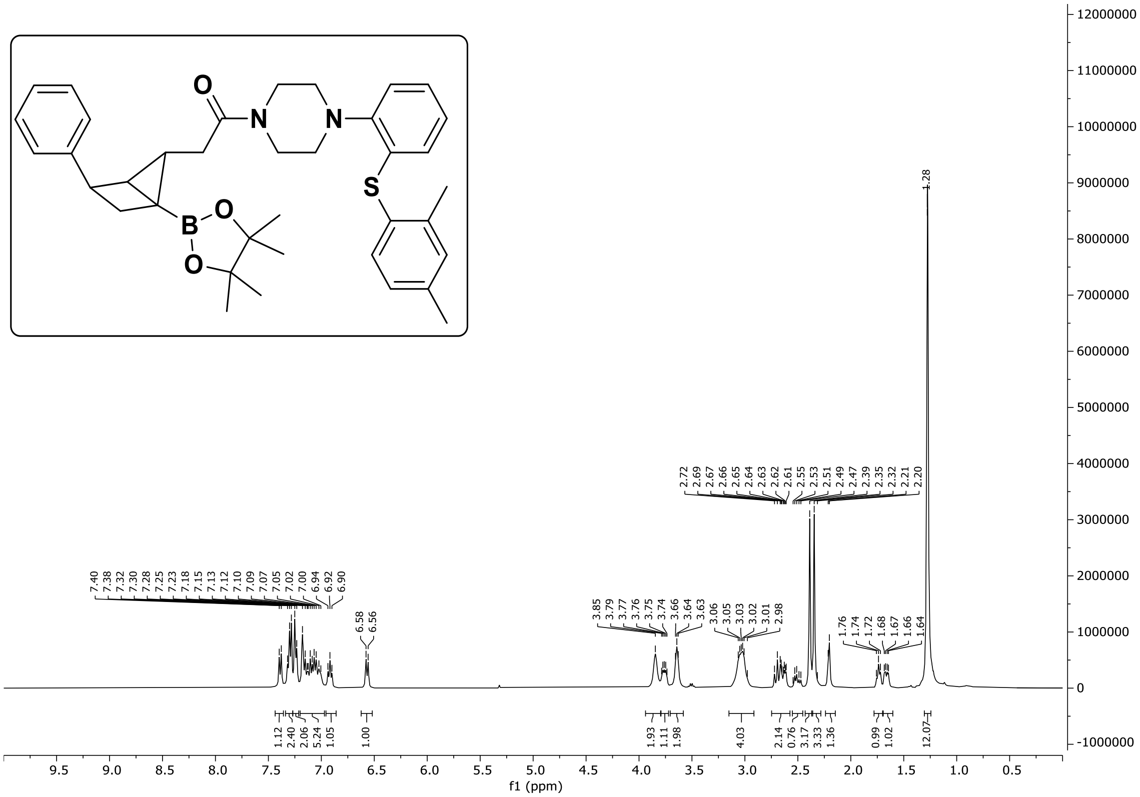

^{13}C-NMR of 47 (151 MHz, $CDCl_3$)

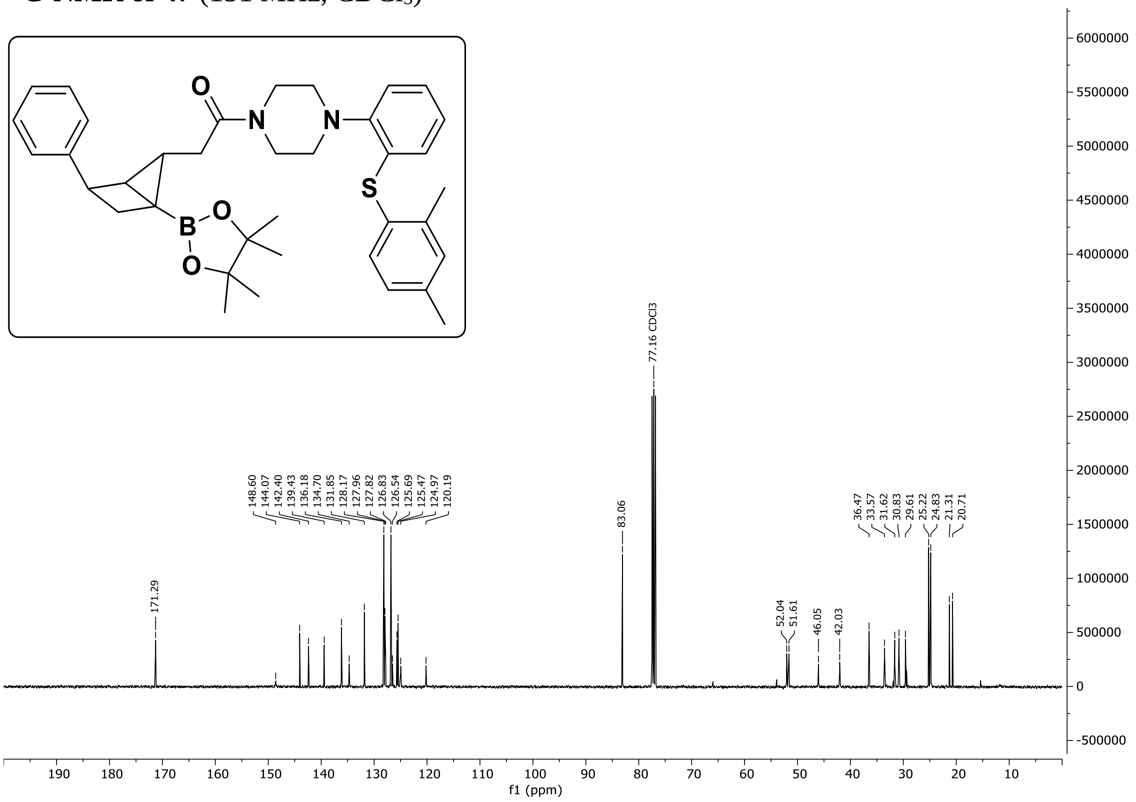

^{11}B NMR of 47 (128 MHz, $CDCl_3$)

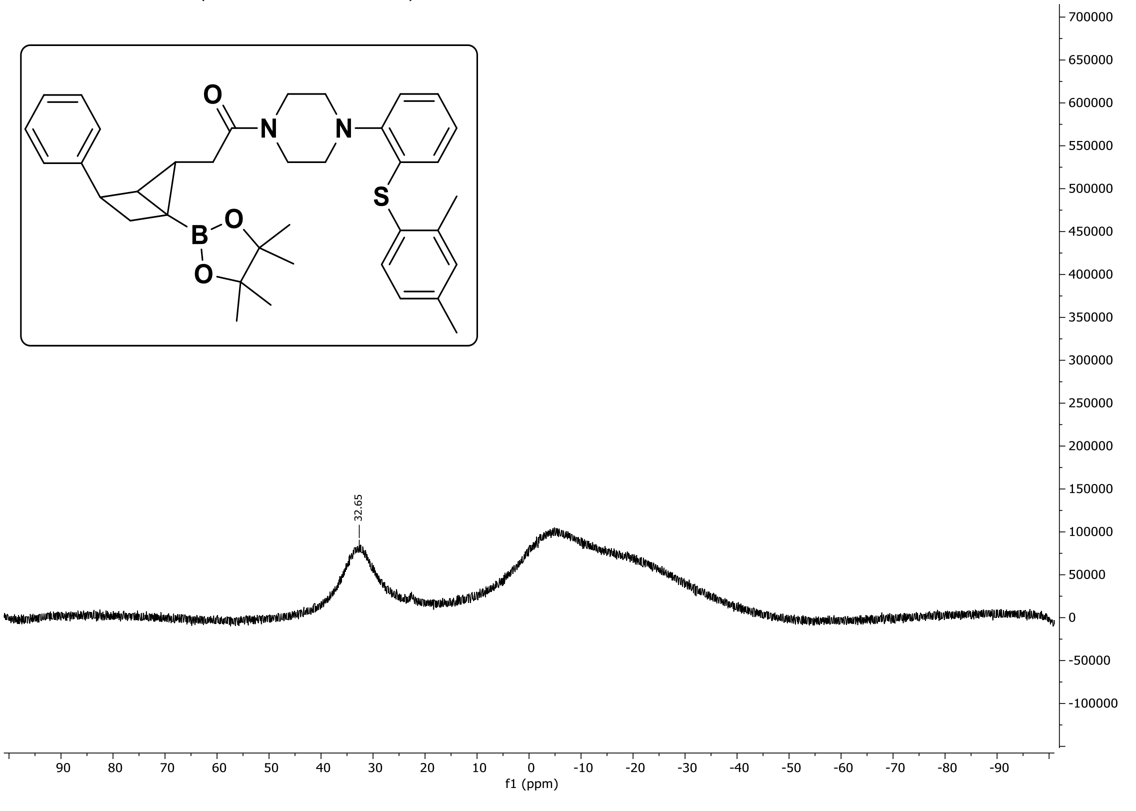

^{1}H-NMR of 48 (400 MHz, $CDCl_3$)

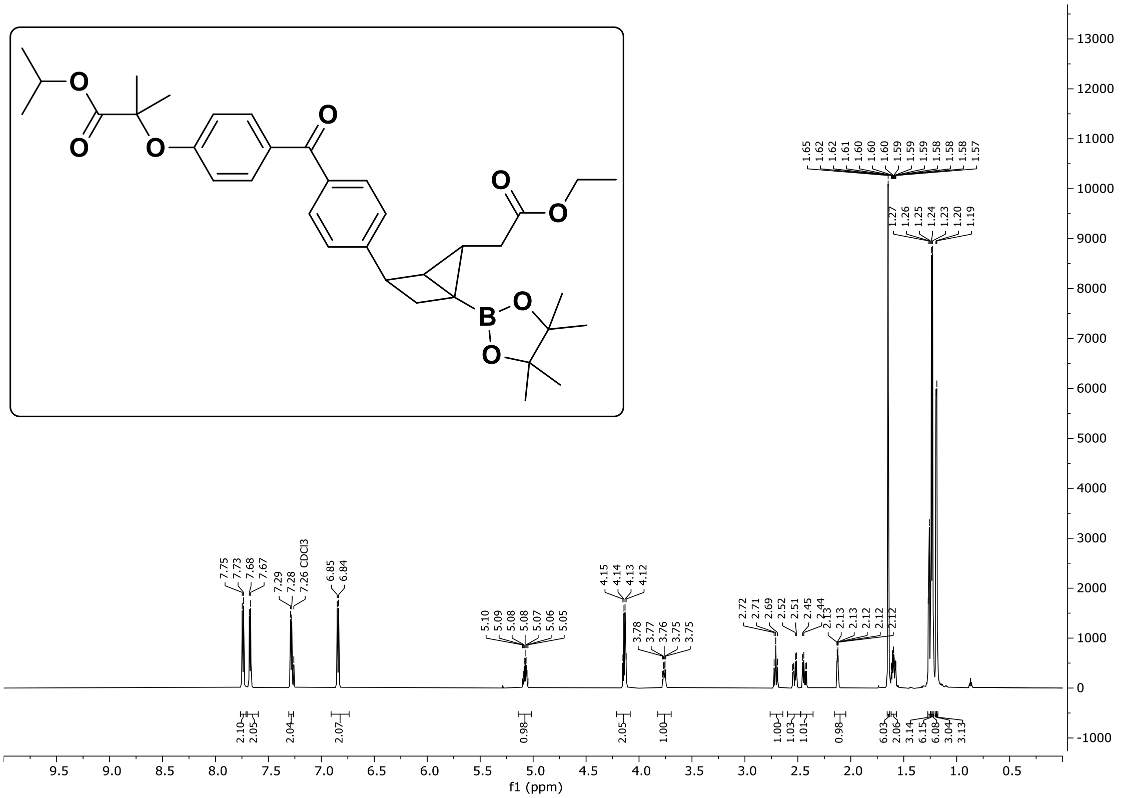

^{13}C-NMR of 48 (151 MHz, $CDCl_3$)

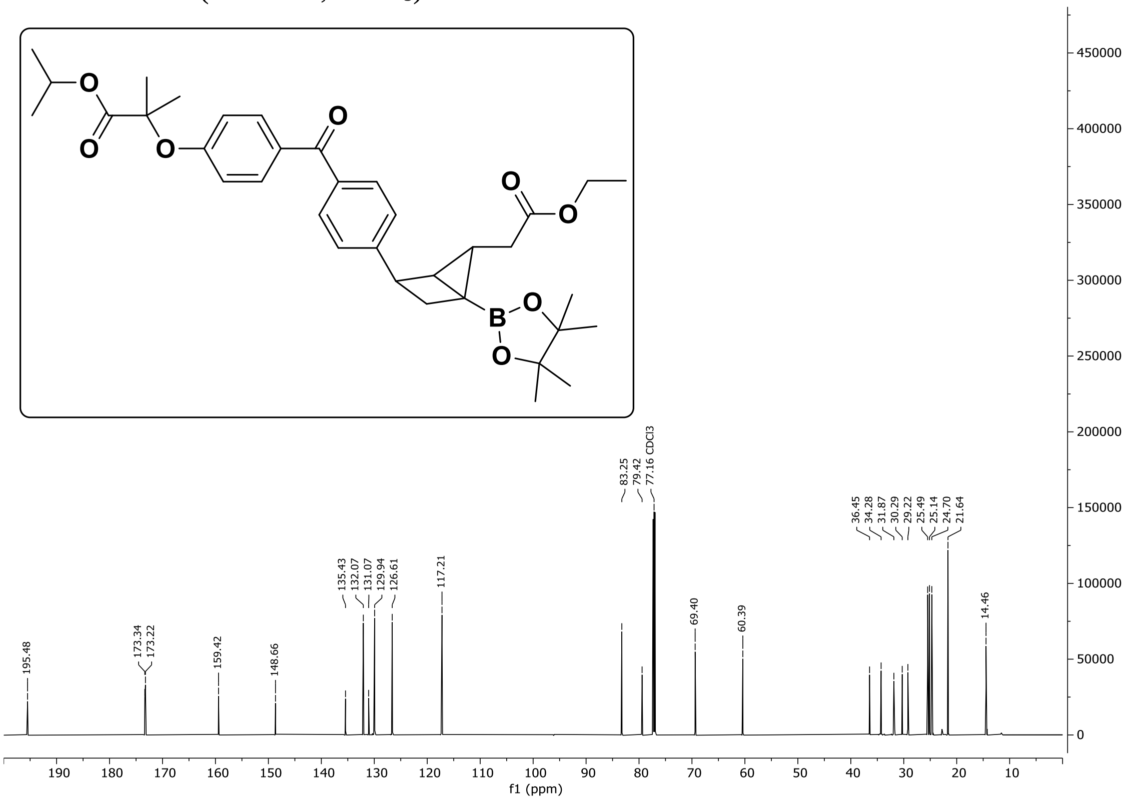

^{11}B NMR of 48 (128 MHz, $CDCl_3$)

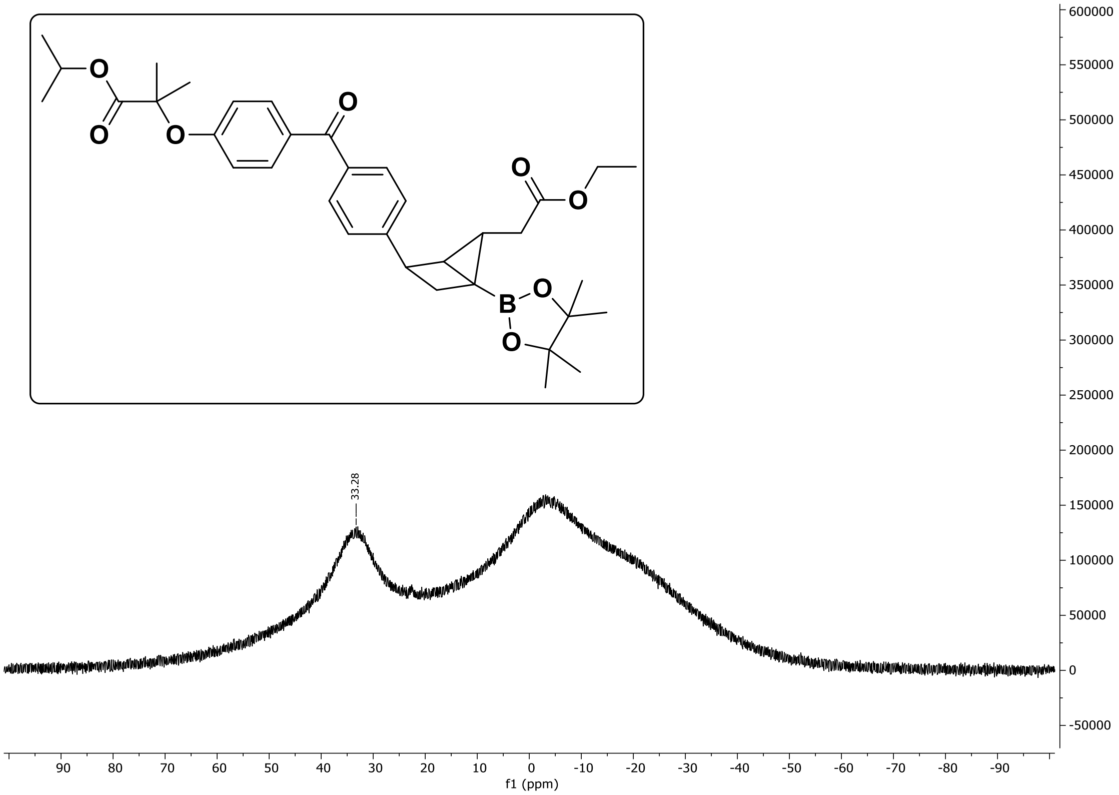

^{1}H-NMR of S31 (400 MHz, $CDCl_3$)

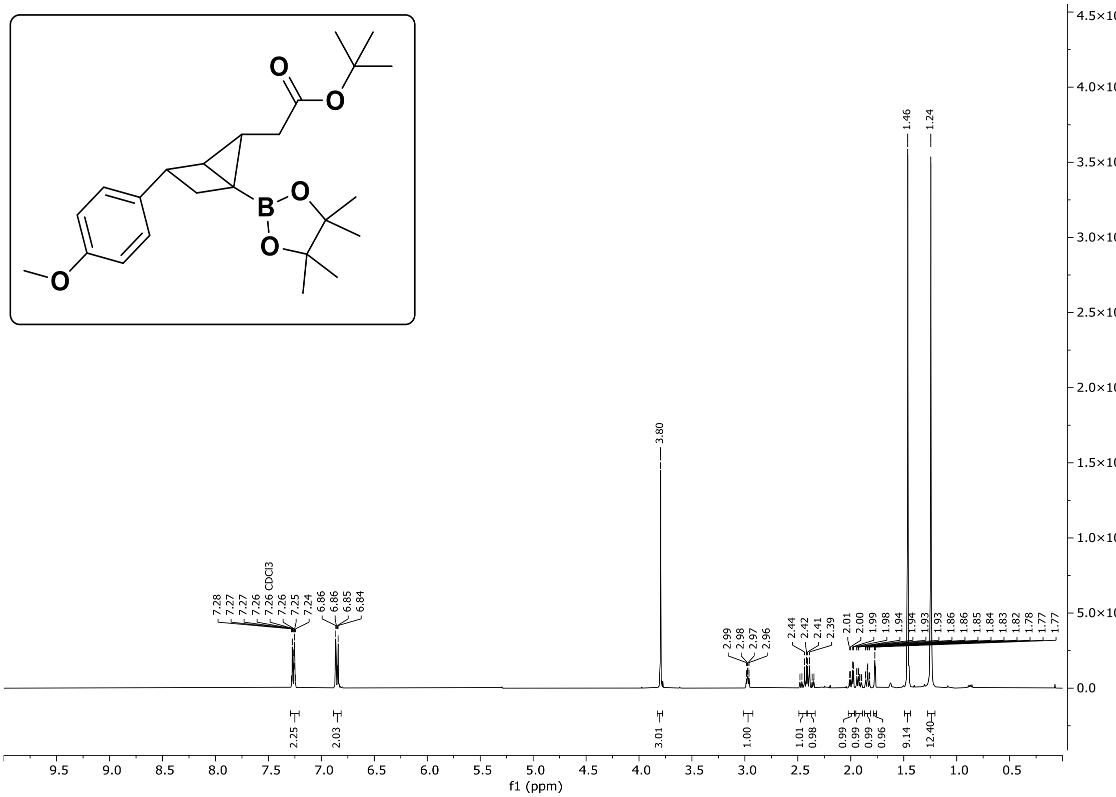

^{13}C-NMR of S31 (100 MHz, $CDCl_3$)

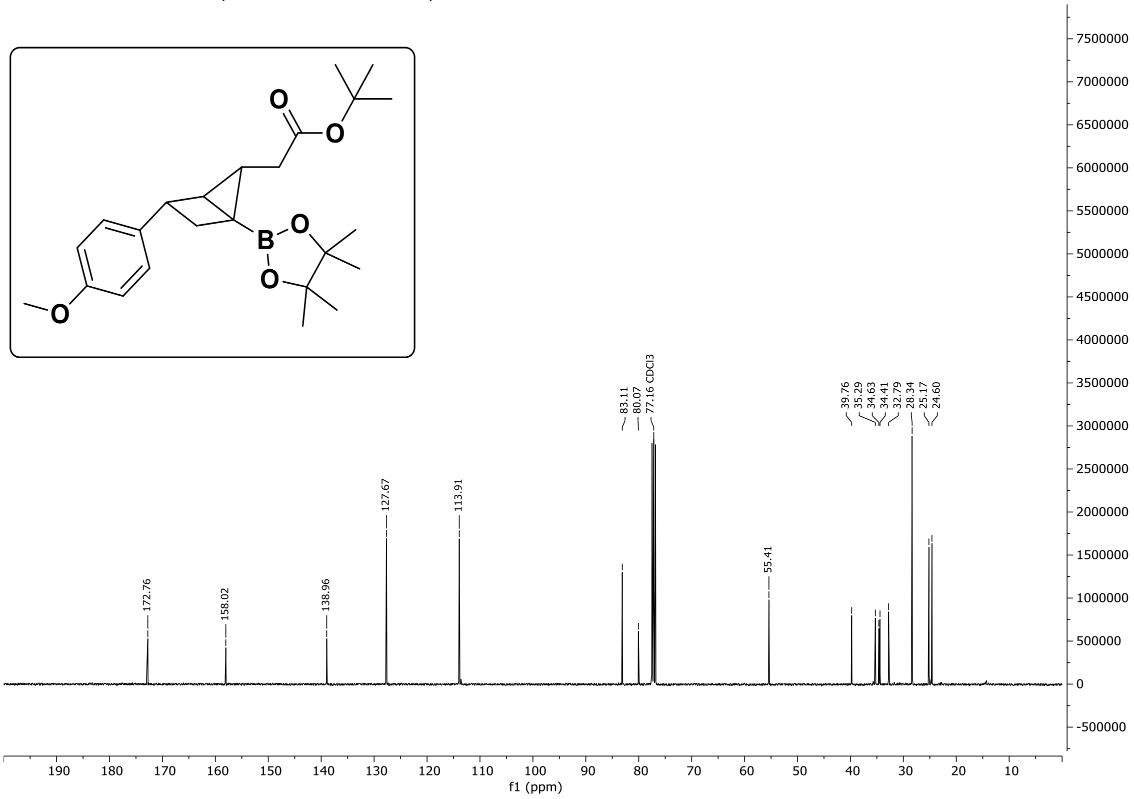

^{11}B NMR of S31 (128 MHz, $CDCl_3$)

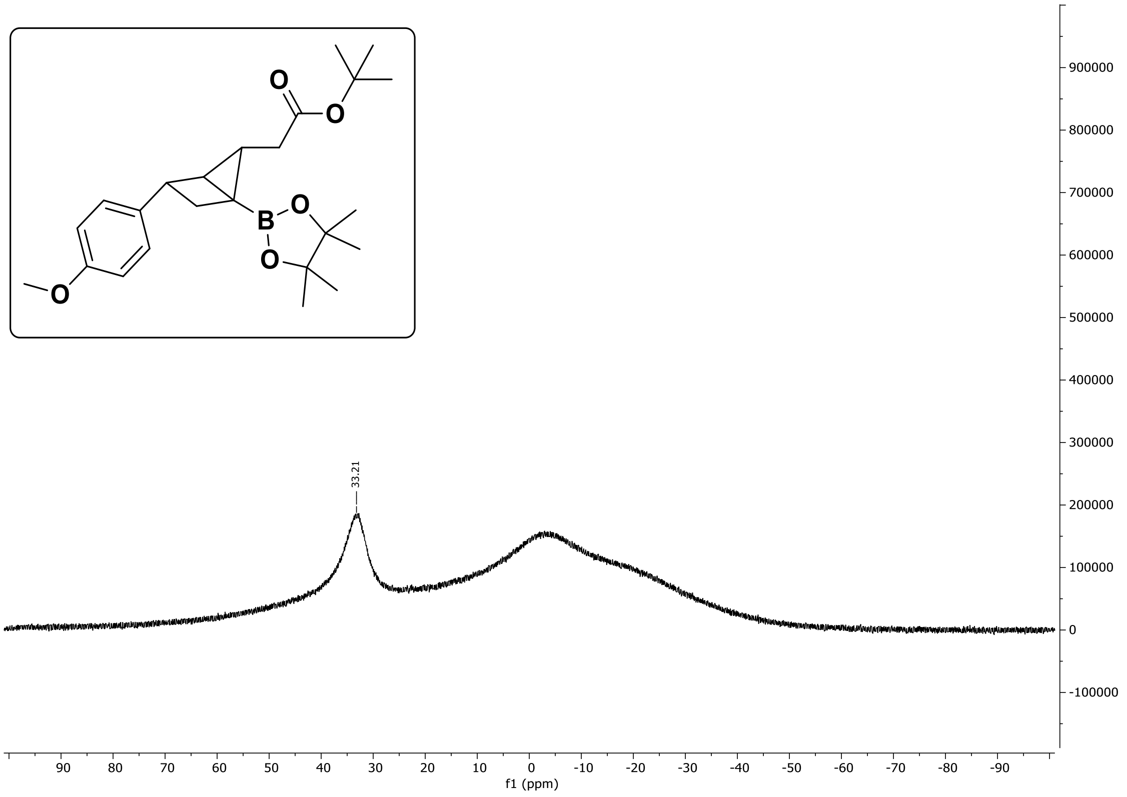

NOESY of S31:

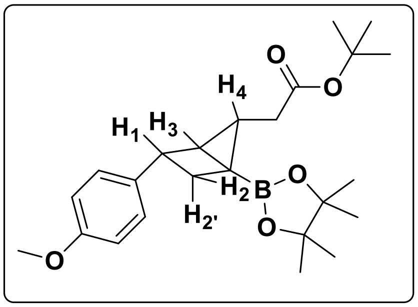

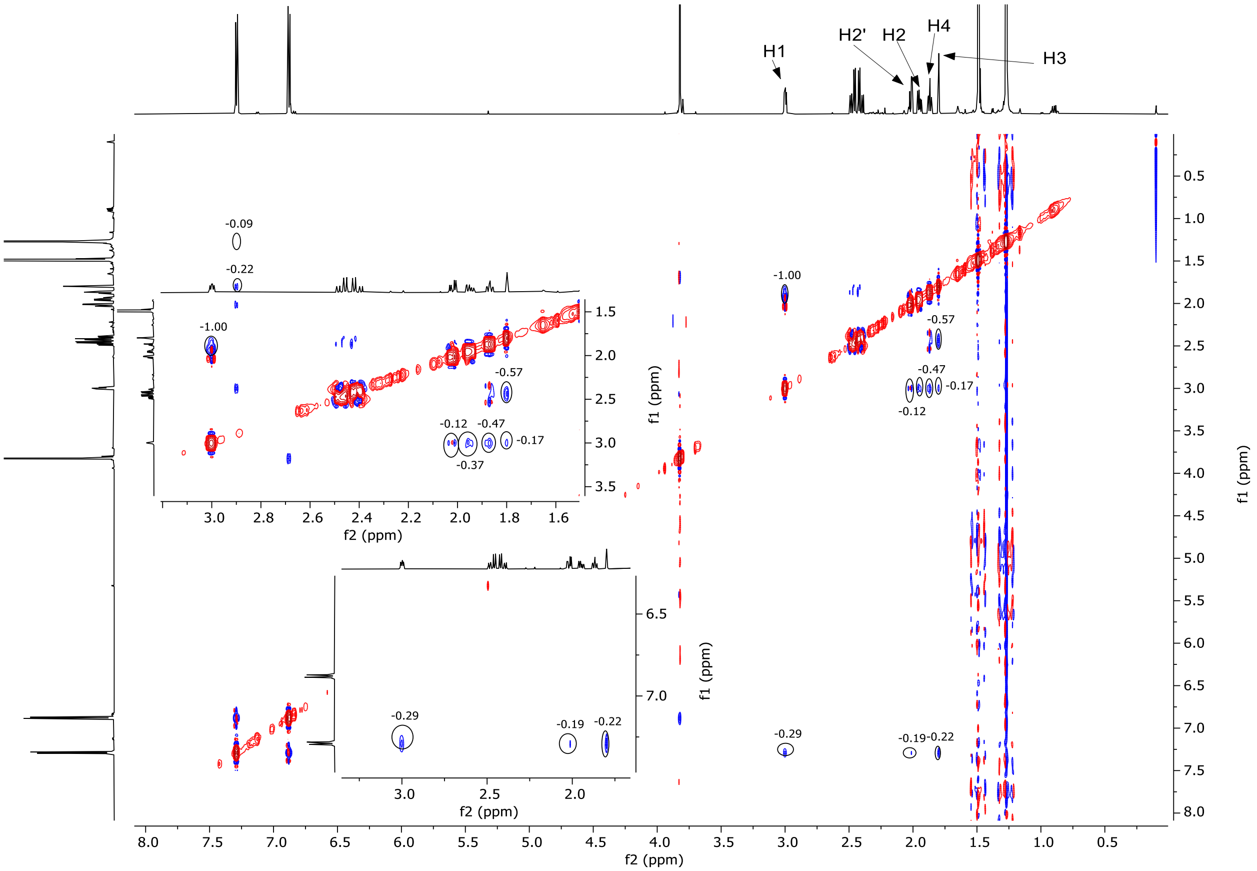

H4 has good nOe with H1, but H3 has weak nOe with H1;

H3 has good nOe with ArH, but H4 has no nOe with ArH.

^{1}H-NMR of 49 (400 MHz, $CDCl_3$)

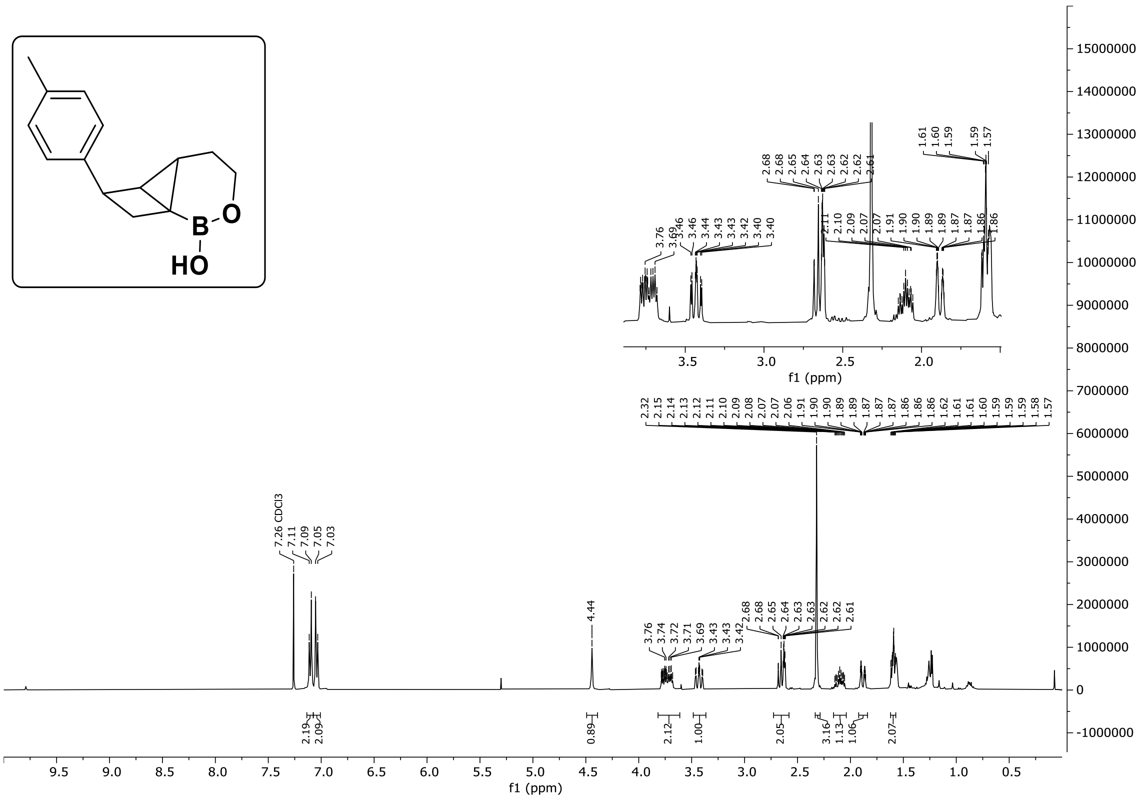

^{13}C-NMR of 49 (100 MHz, $CDCl_3$)

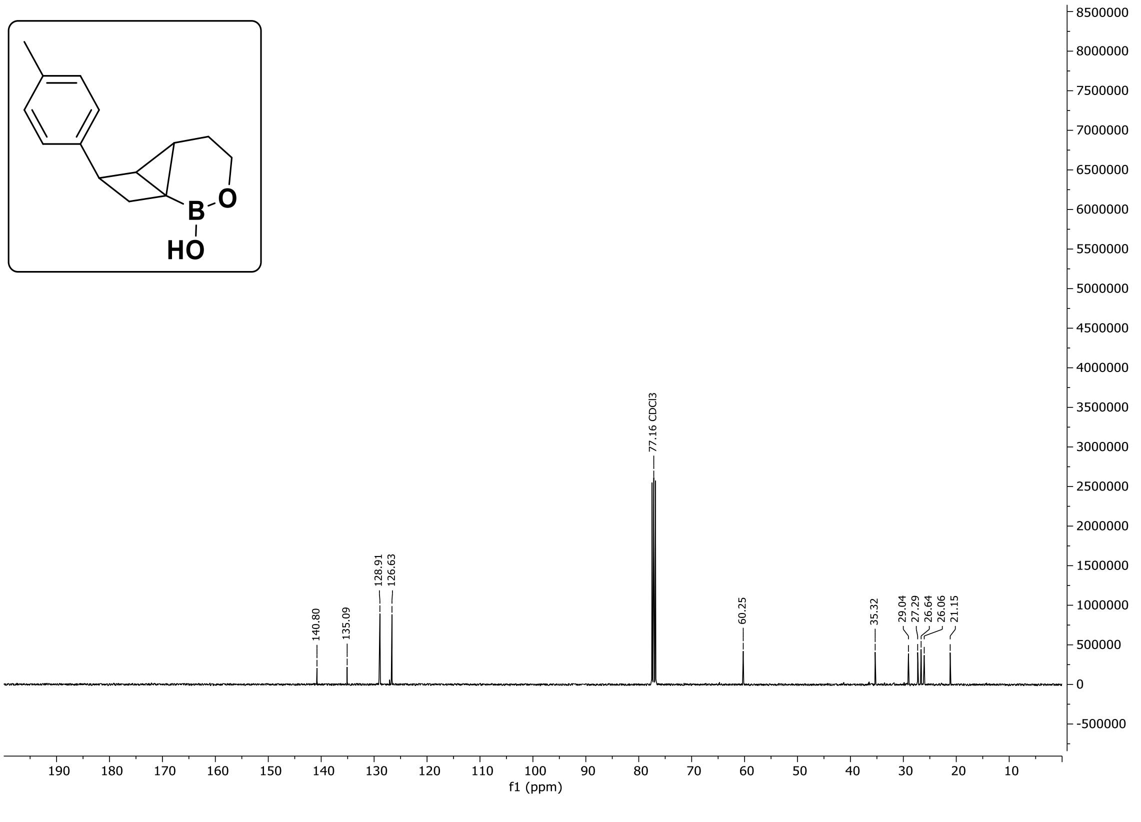

^{11}B NMR of 49 (128 MHz, $CDCl_3$)

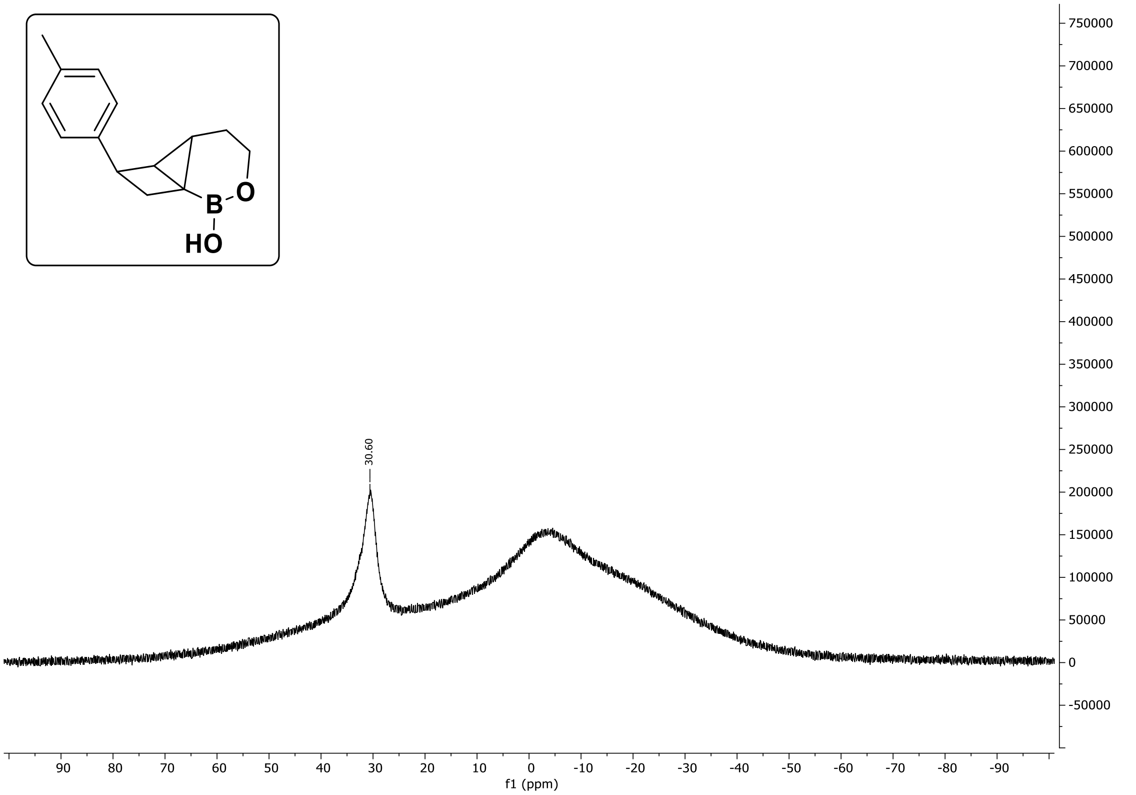

^{1}H NMR of 50 (400 MHz, Pyr)

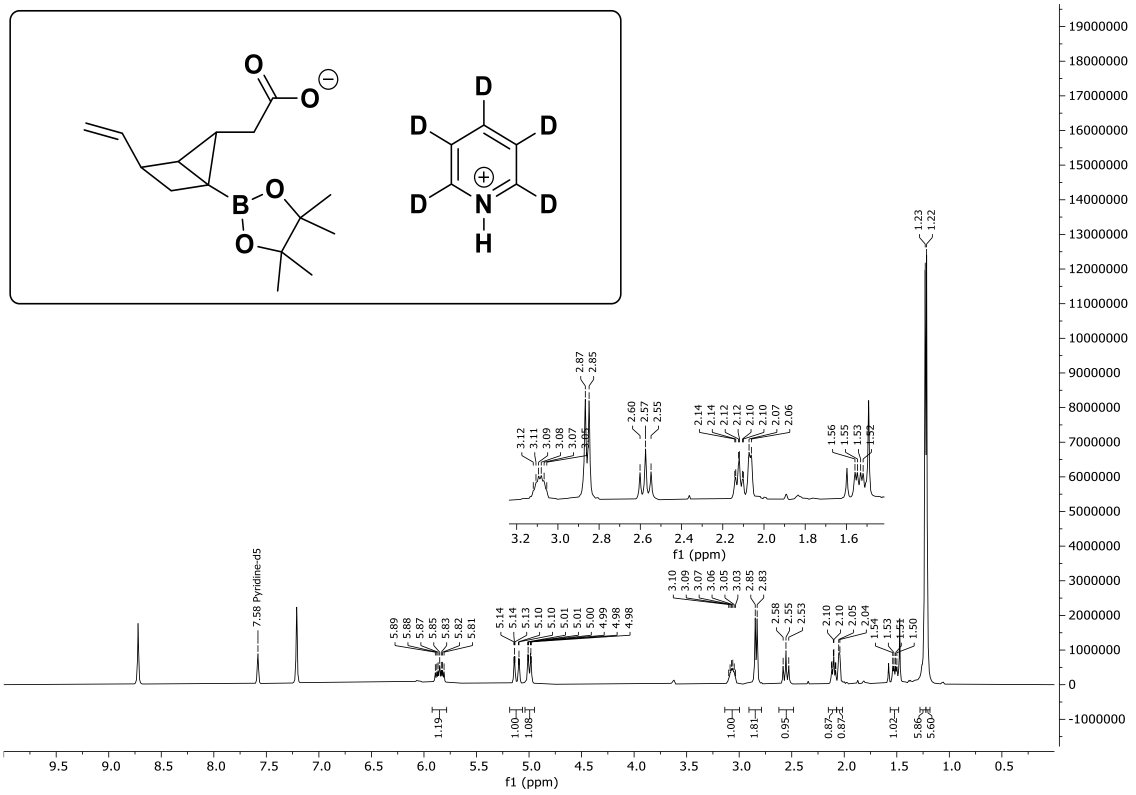

^{13}C NMR of 50 (100 MHz, Pyr)

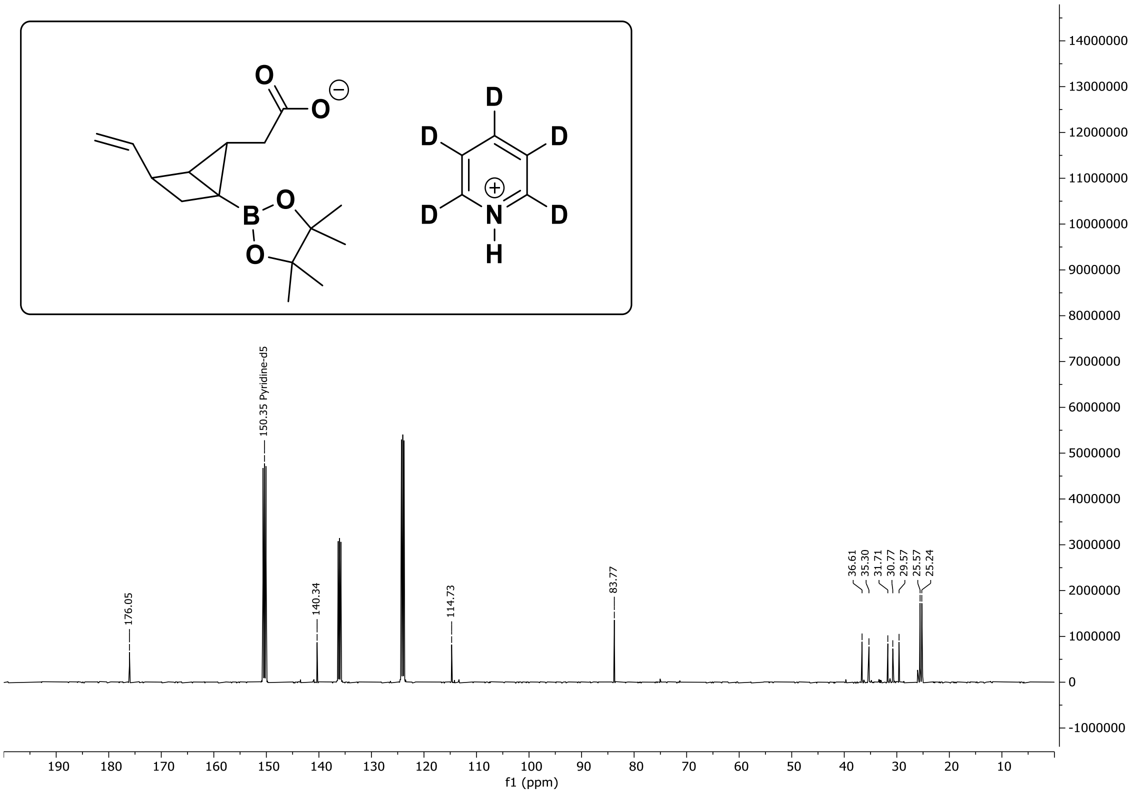

^{11}B NMR of 50 (128 MHz, Pyr)

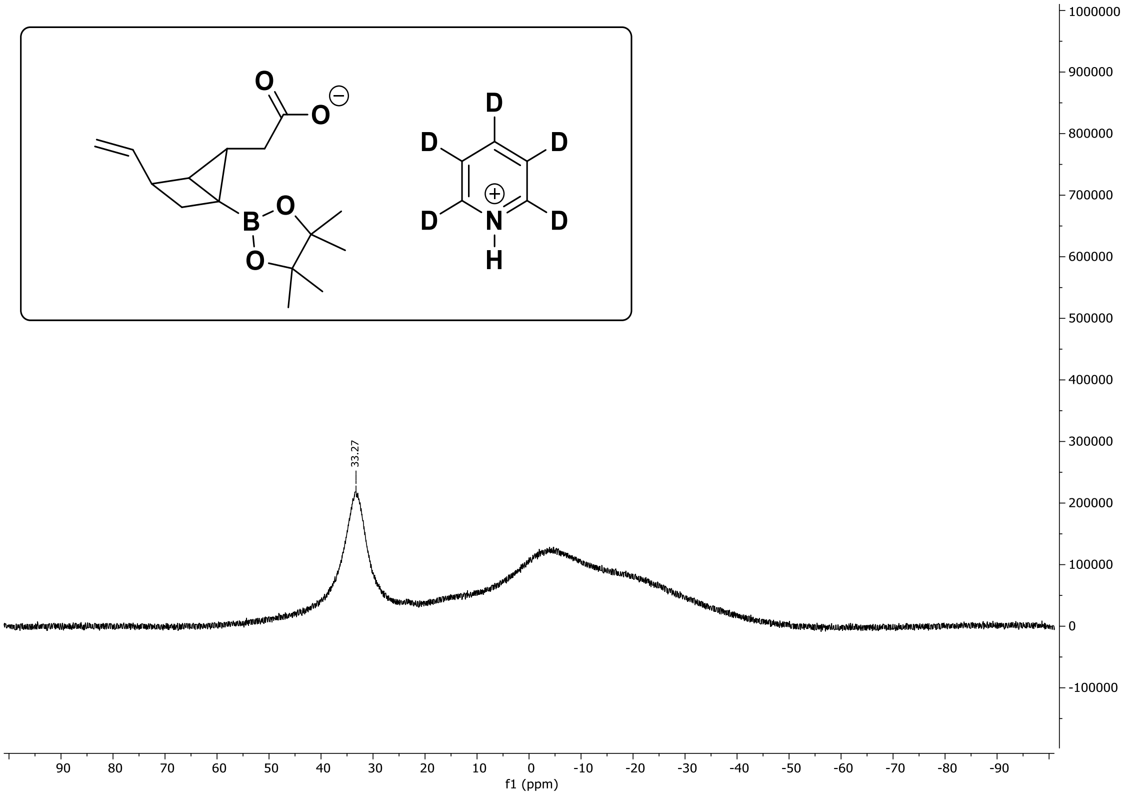

^{1}H-NMR of 51 (400 MHz, Acetone)

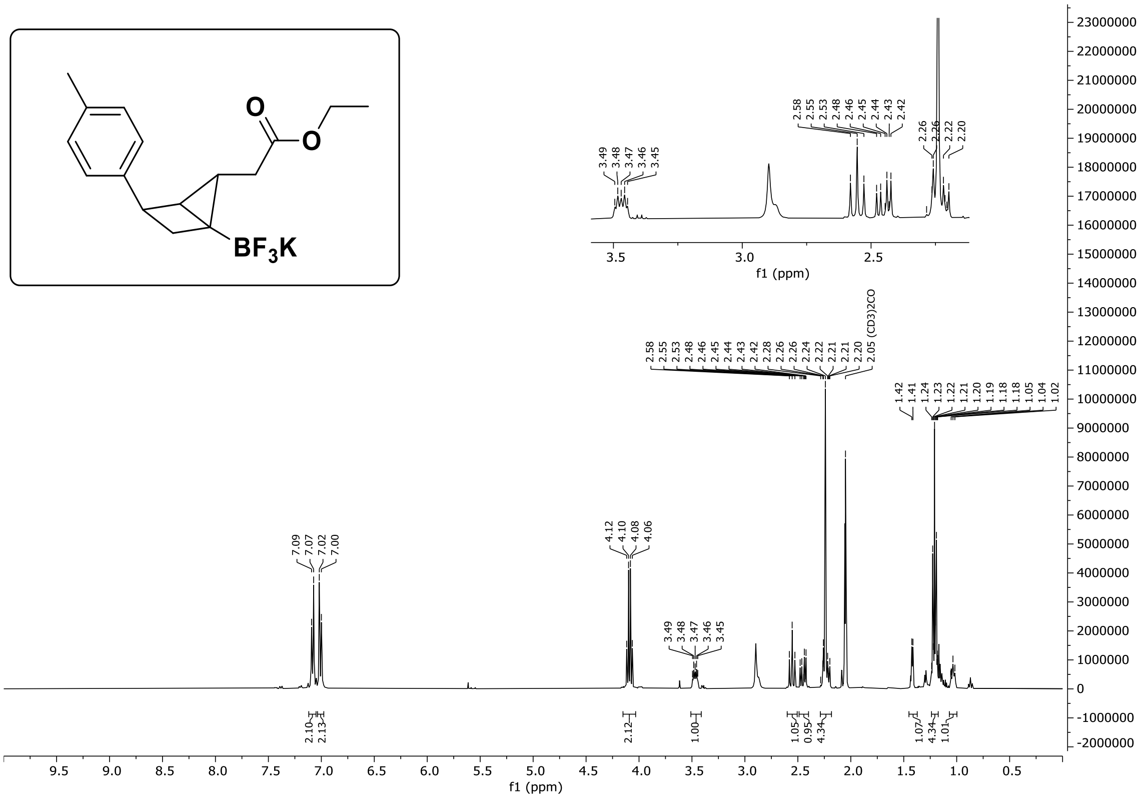

^{13}C-NMR of 51 (100 MHz, Acetone)

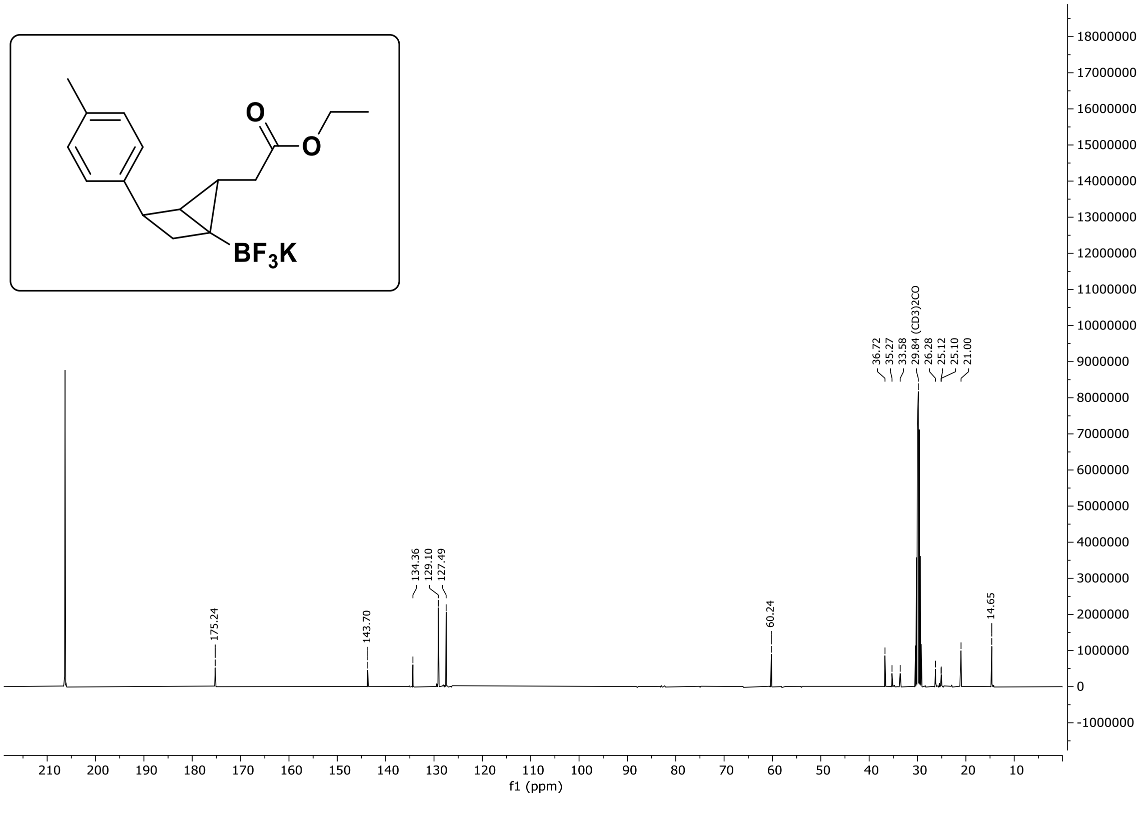

^{19}F-NMR of 51 (376 MHz, Acetone)

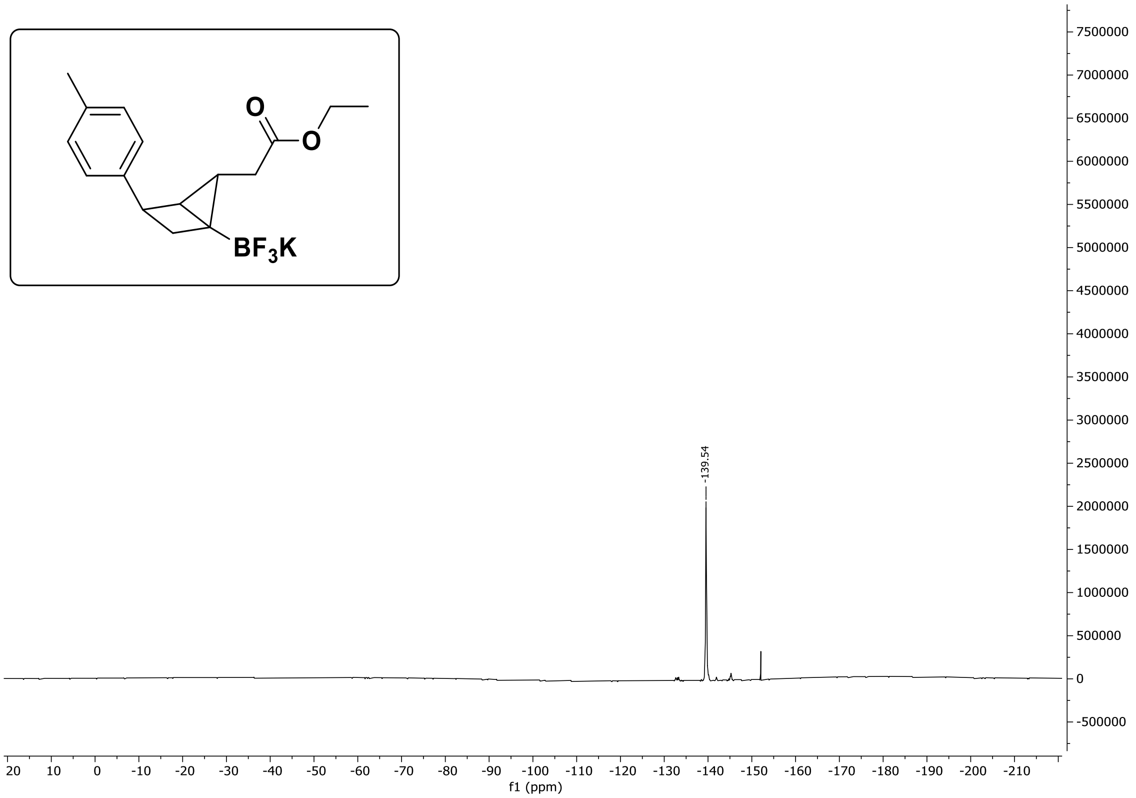

^{11}B NMR of 51 (128 MHz, Acetone)

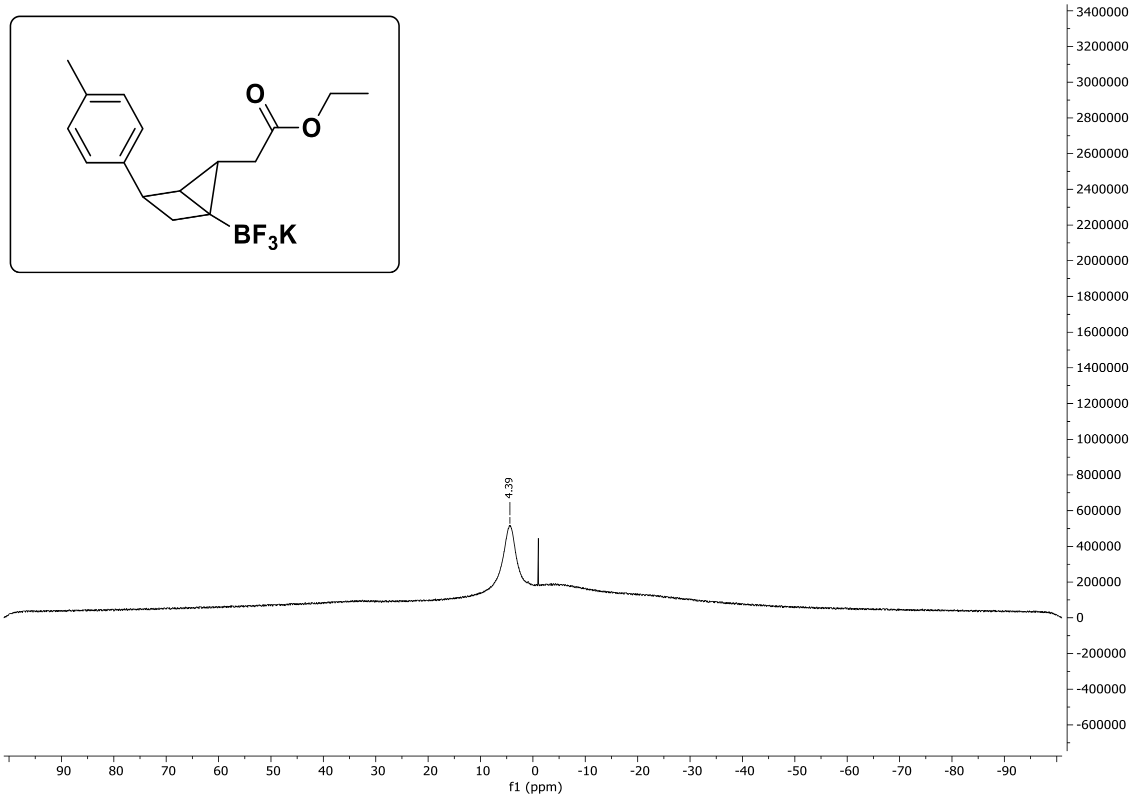

^{1}H-NMR of 52 (400 MHz, $CDCl_3$)

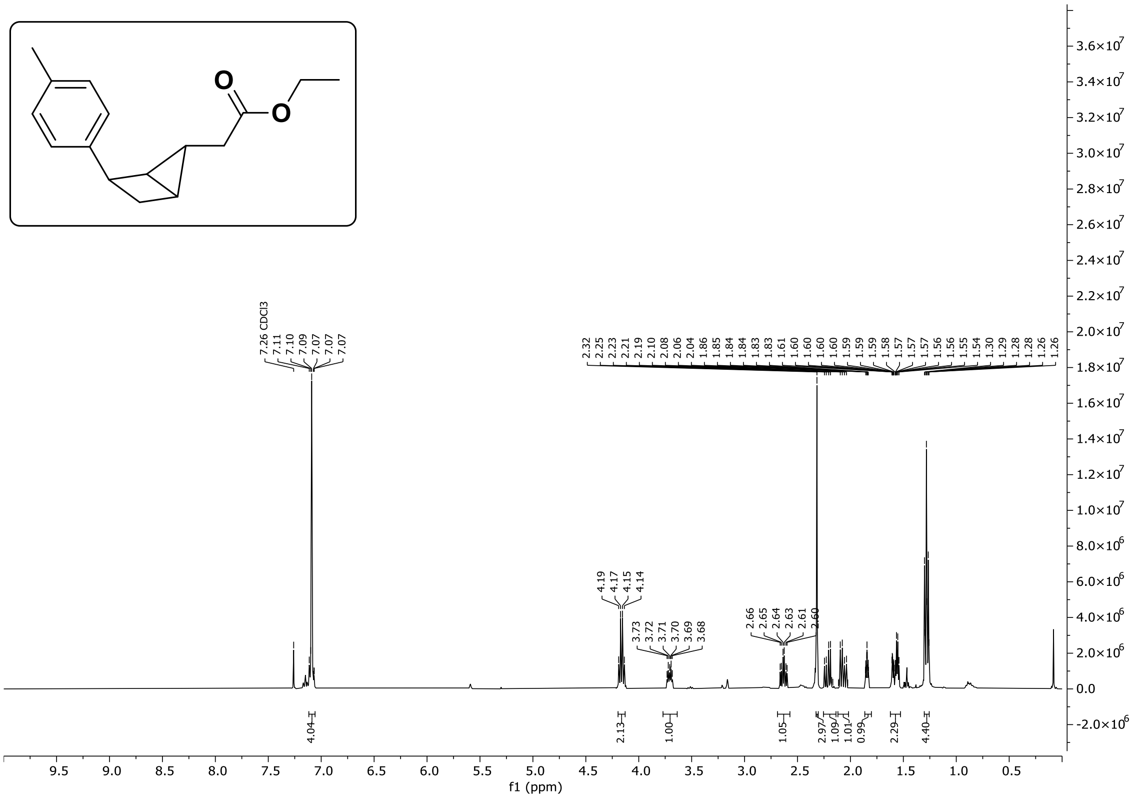

^{13}C-NMR of 52 (100 MHz, $CDCl_3$)

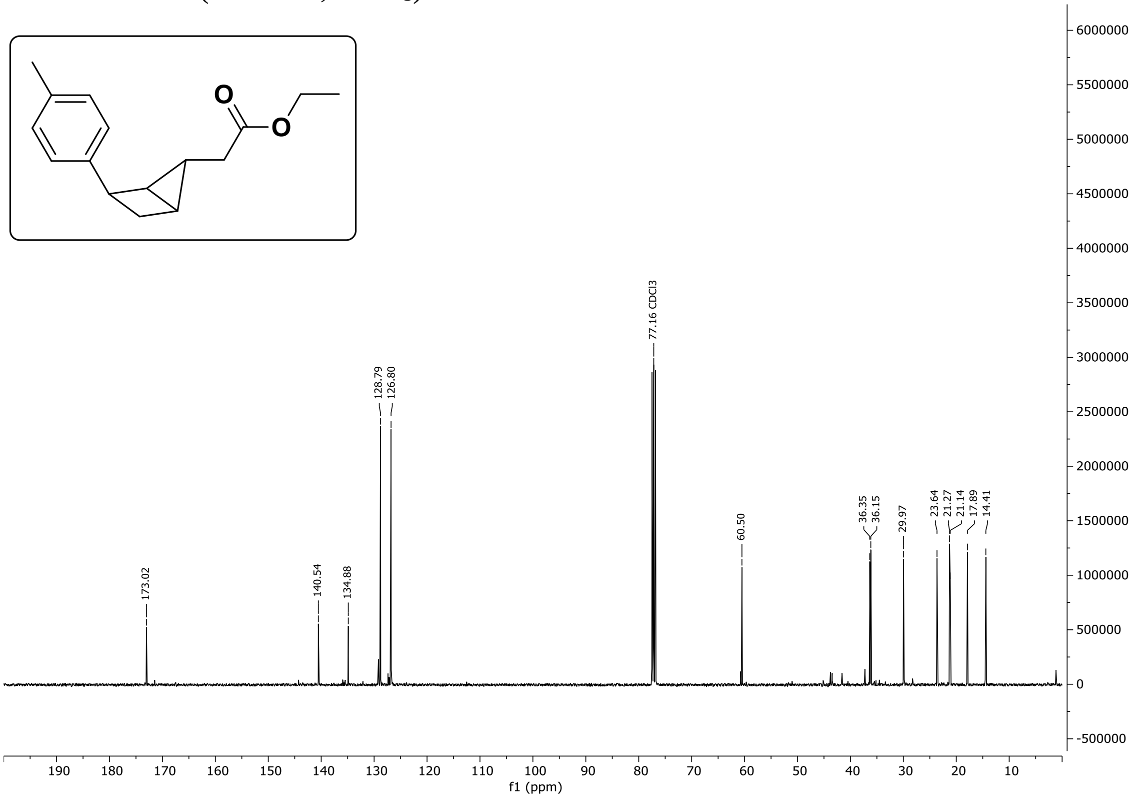

^{1}H-NMR of 53 (400 MHz, $CDCl_3$)

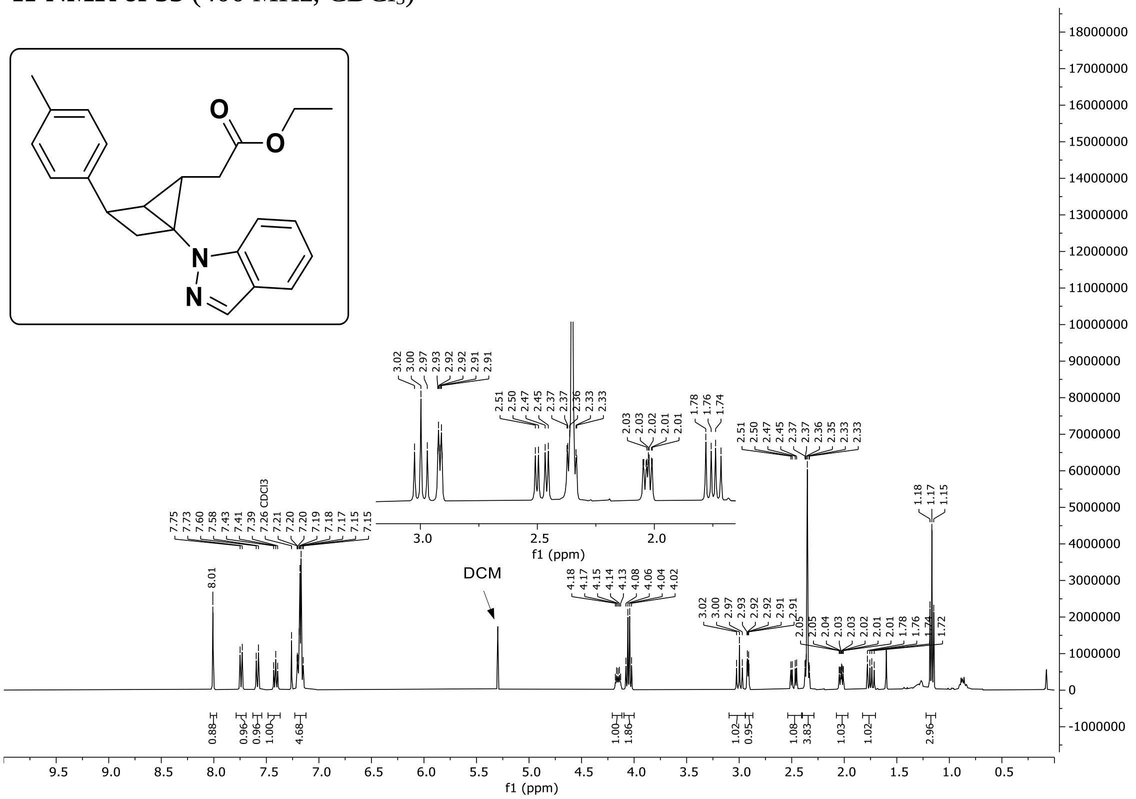

^{13}C-NMR of 53 (100 MHz, $CDCl_3$)

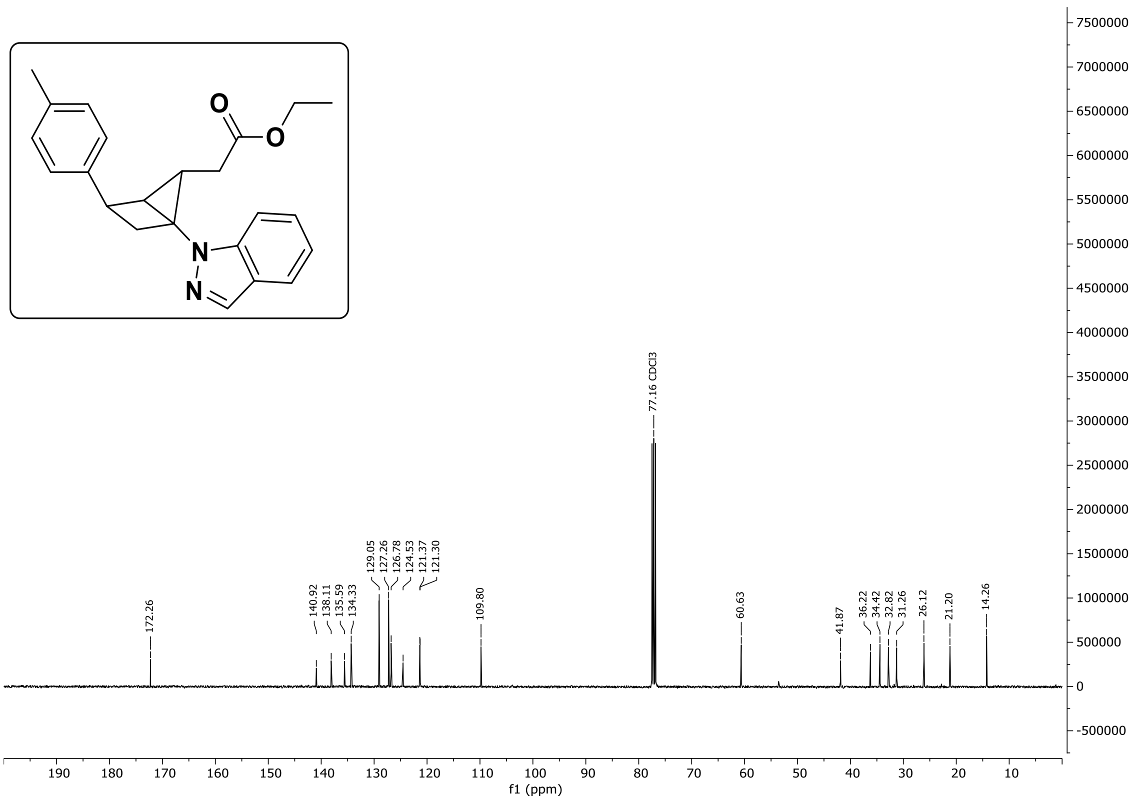

^{1}H-NMR of 54 (400 MHz, $CDCl_3$)

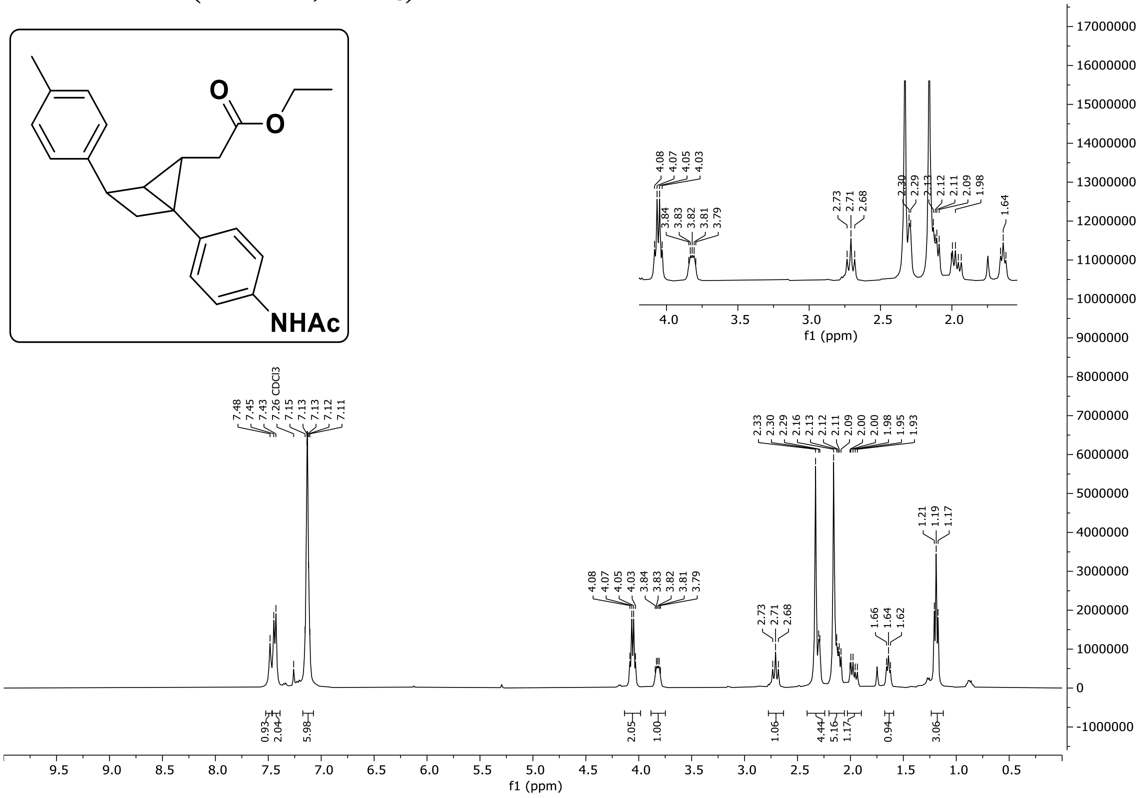

^{13}C-NMR of 54 (100 MHz, $CDCl_3$)

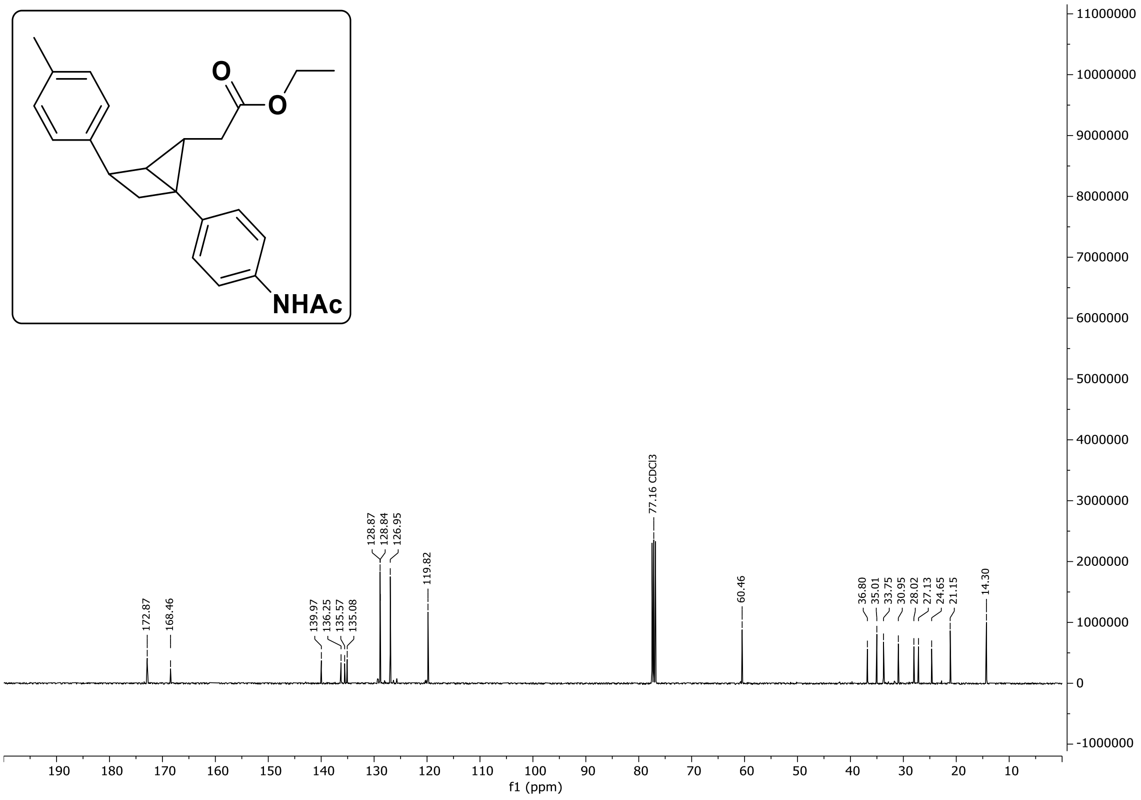

^{1}H-NMR of 55 (400 MHz, $CDCl_3$)

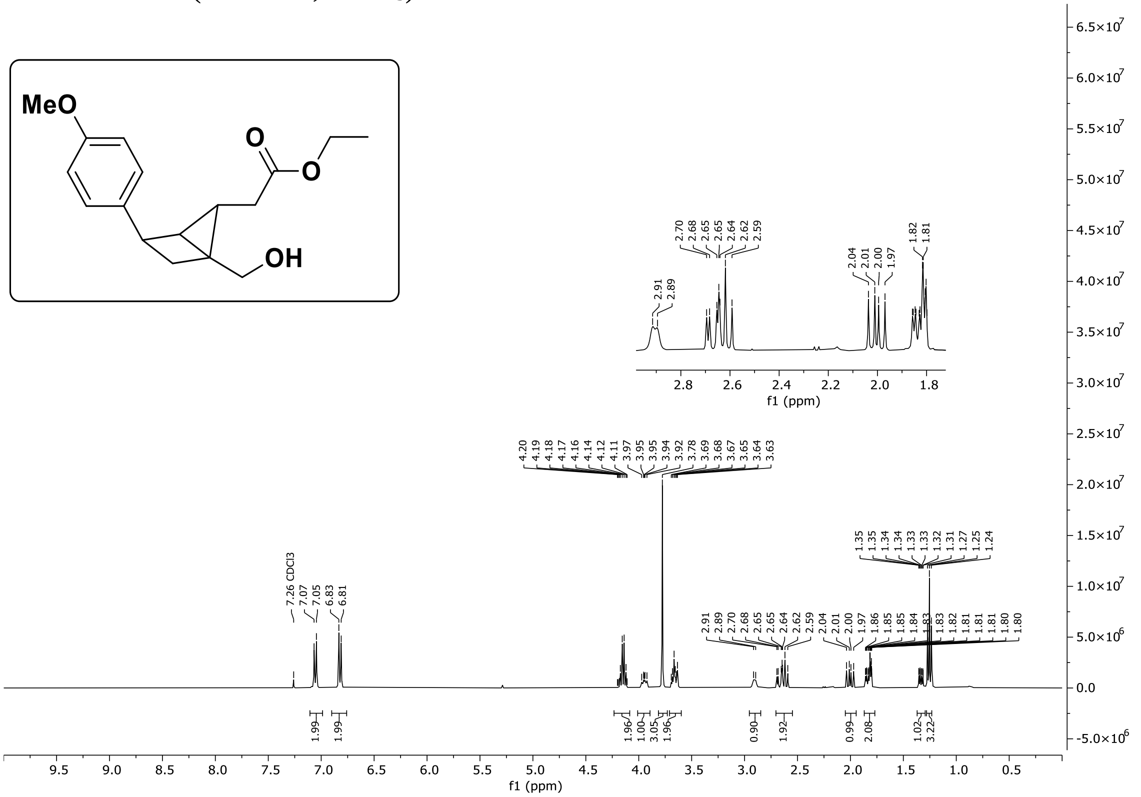

^{13}C-NMR of 55 (100 MHz, $CDCl_3$)

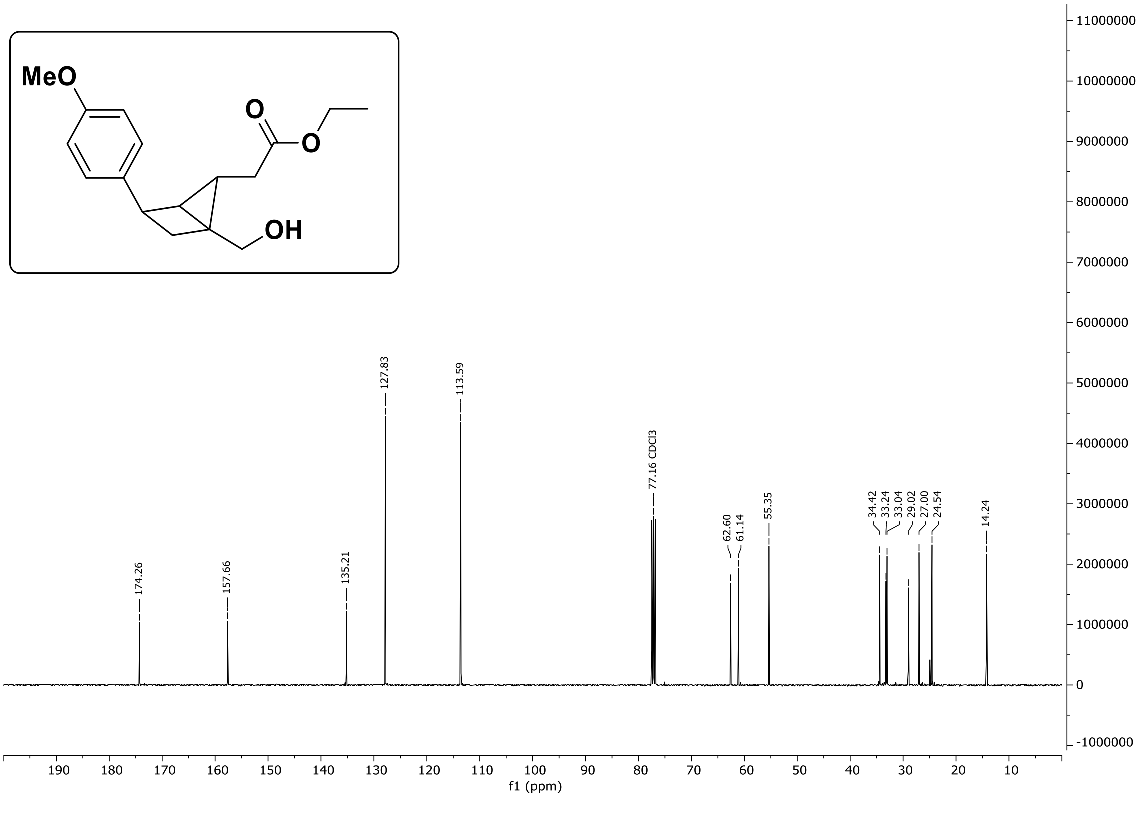

^{1}H-NMR of 56 (400 MHz, $CDCl_3$)

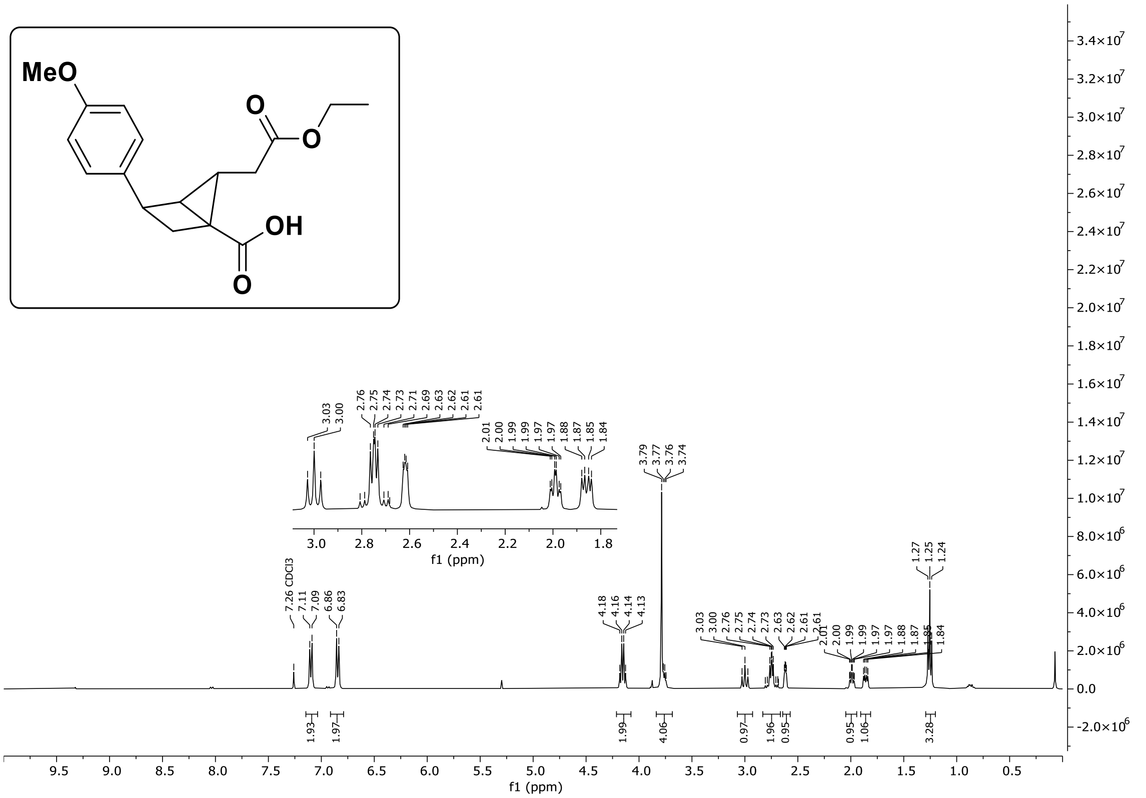

^{13}C-NMR of 56 (100 MHz, $CDCl_3$)

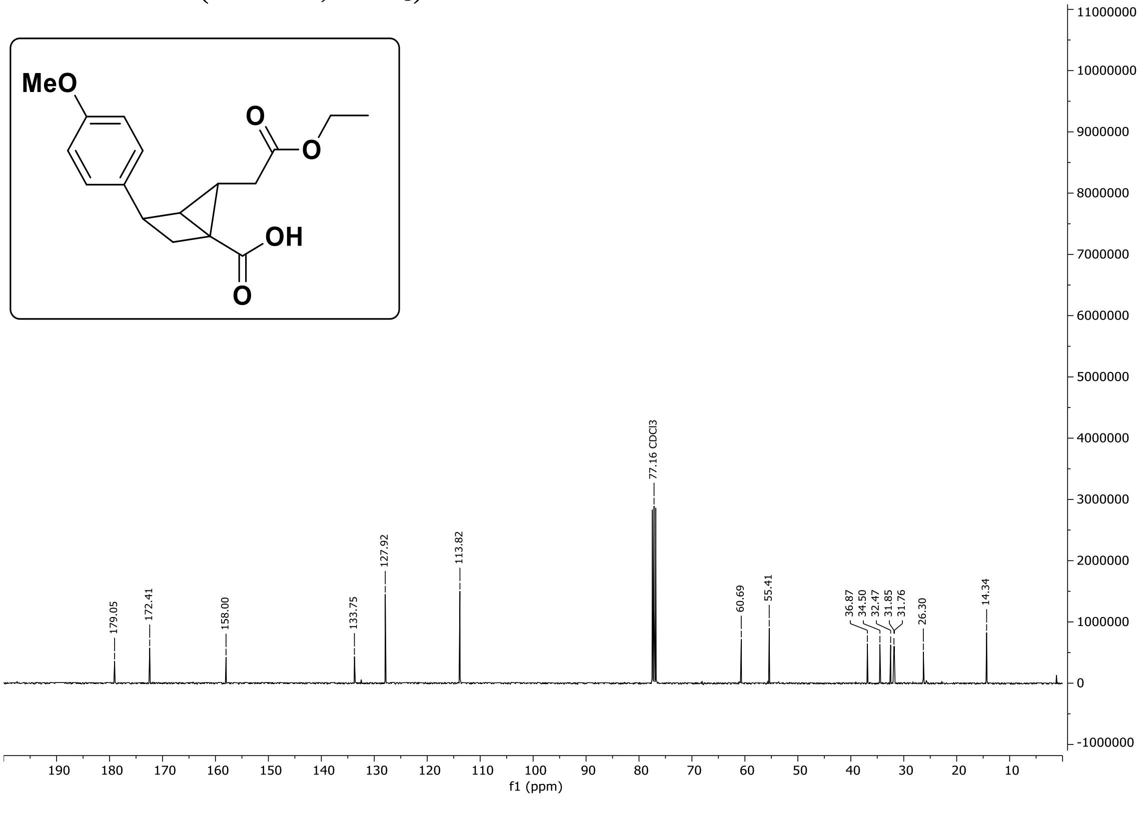

¹H-NMR of 57 (400 MHz, $CDCl_3$)

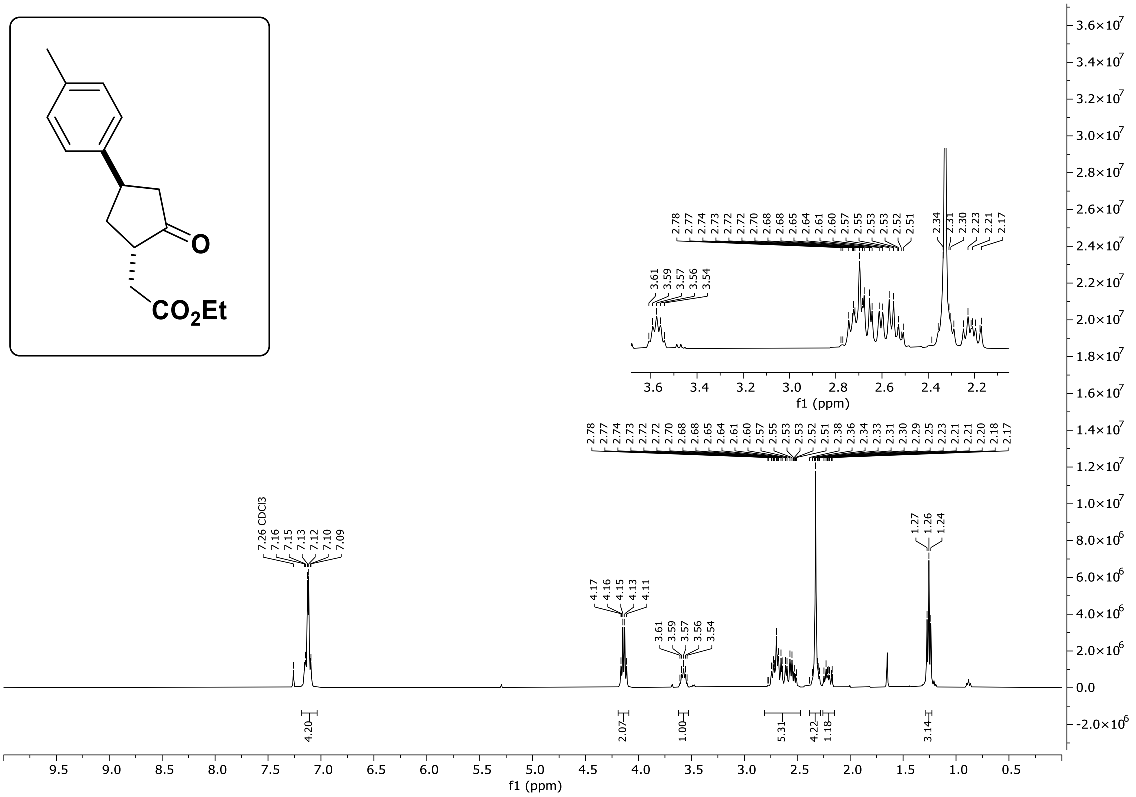

¹³C-NMR of 57 (100 MHz, $CDCl_3$)

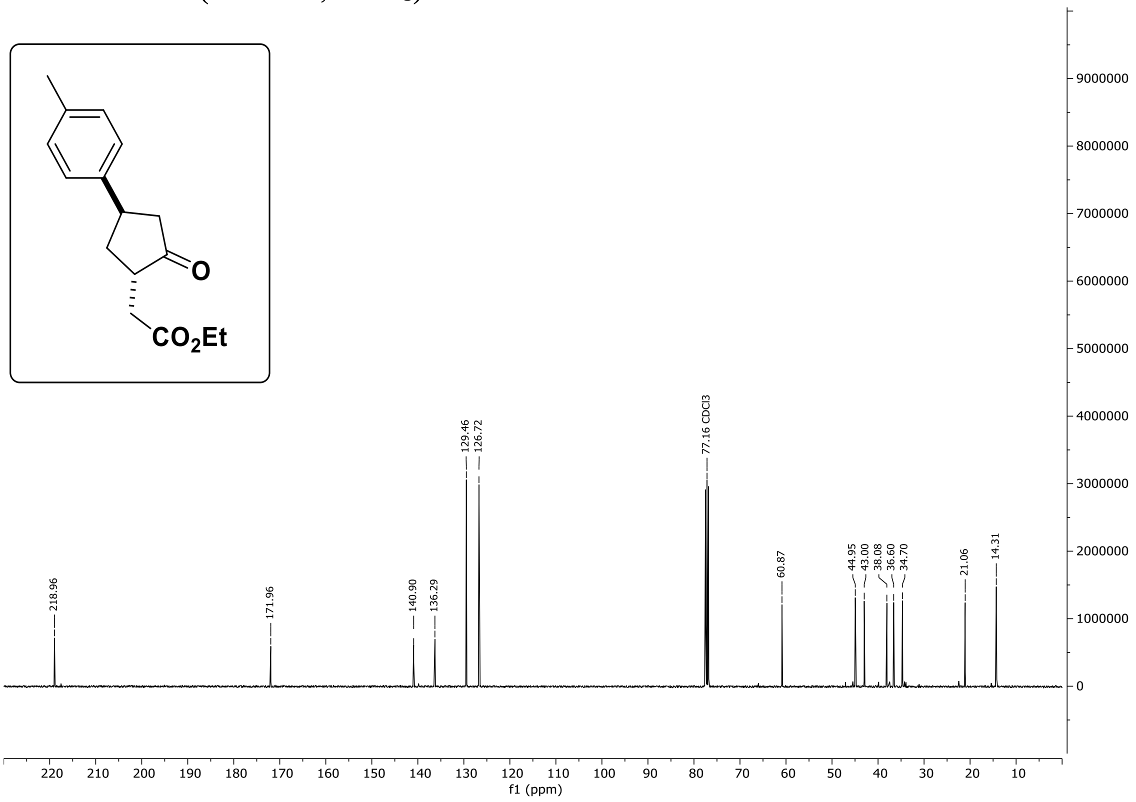

^{1}H-NMR of 58 (400 MHz, $CDCl_3$)

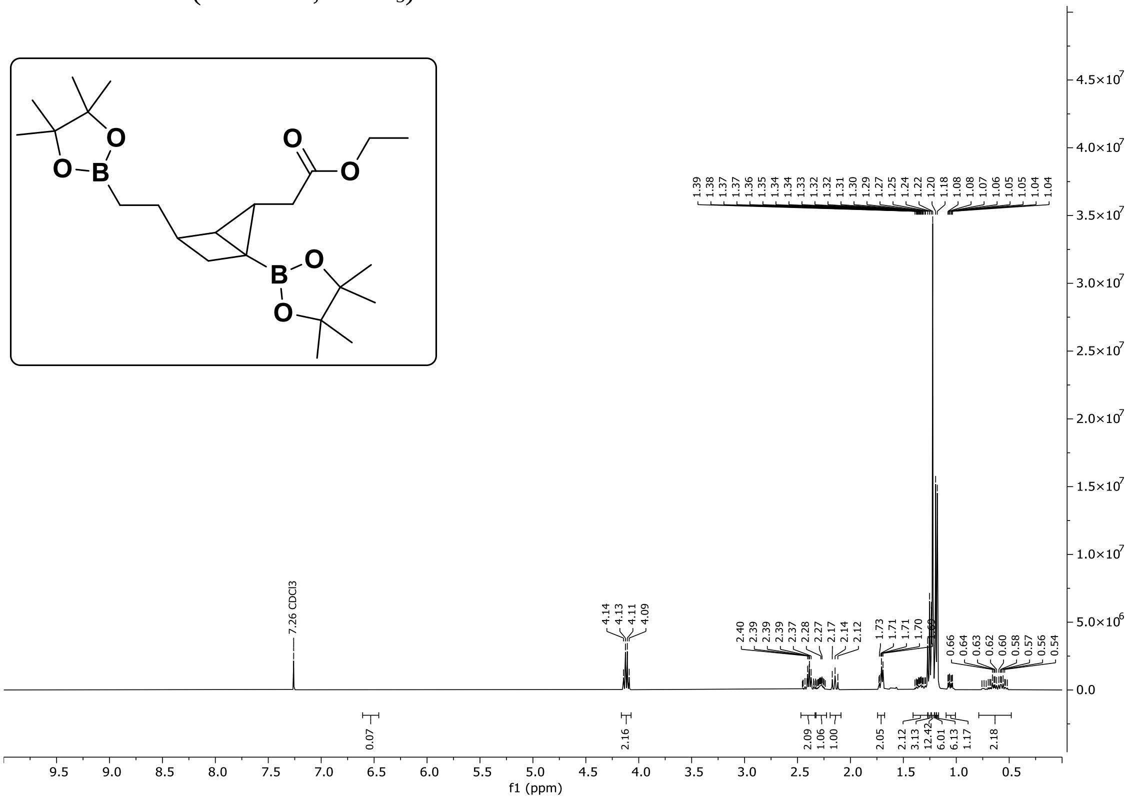

^{13}C-NMR of 58 (100 MHz, $CDCl_3$)

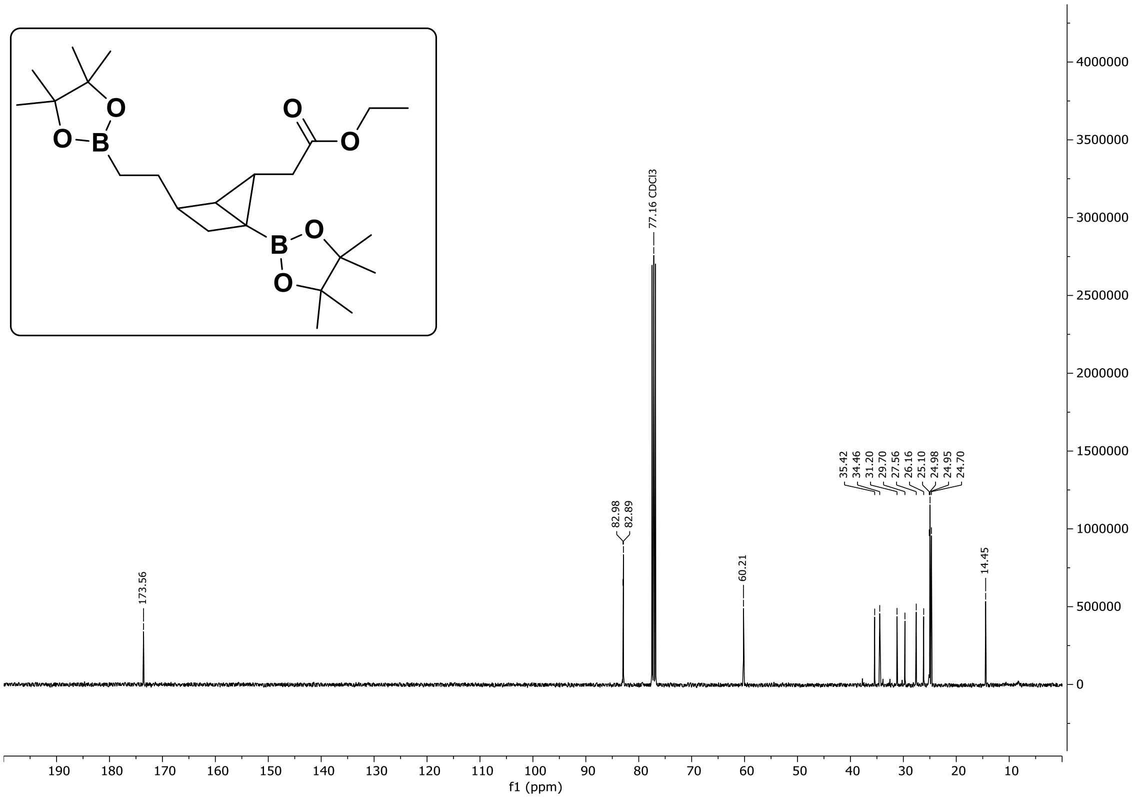

^{11}B NMR of 58 (128 MHz, $CDCl_3$)

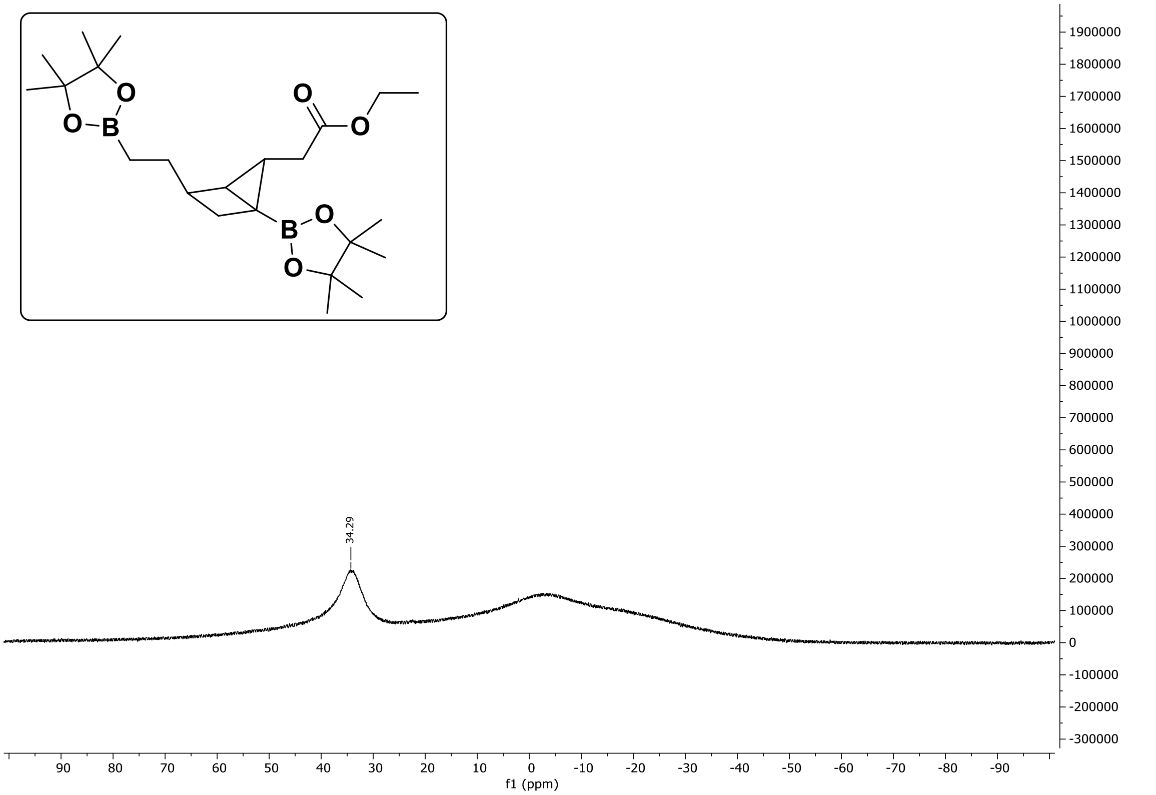

^{1}H-NMR of 59 (400 MHz, $CDCl_3$)

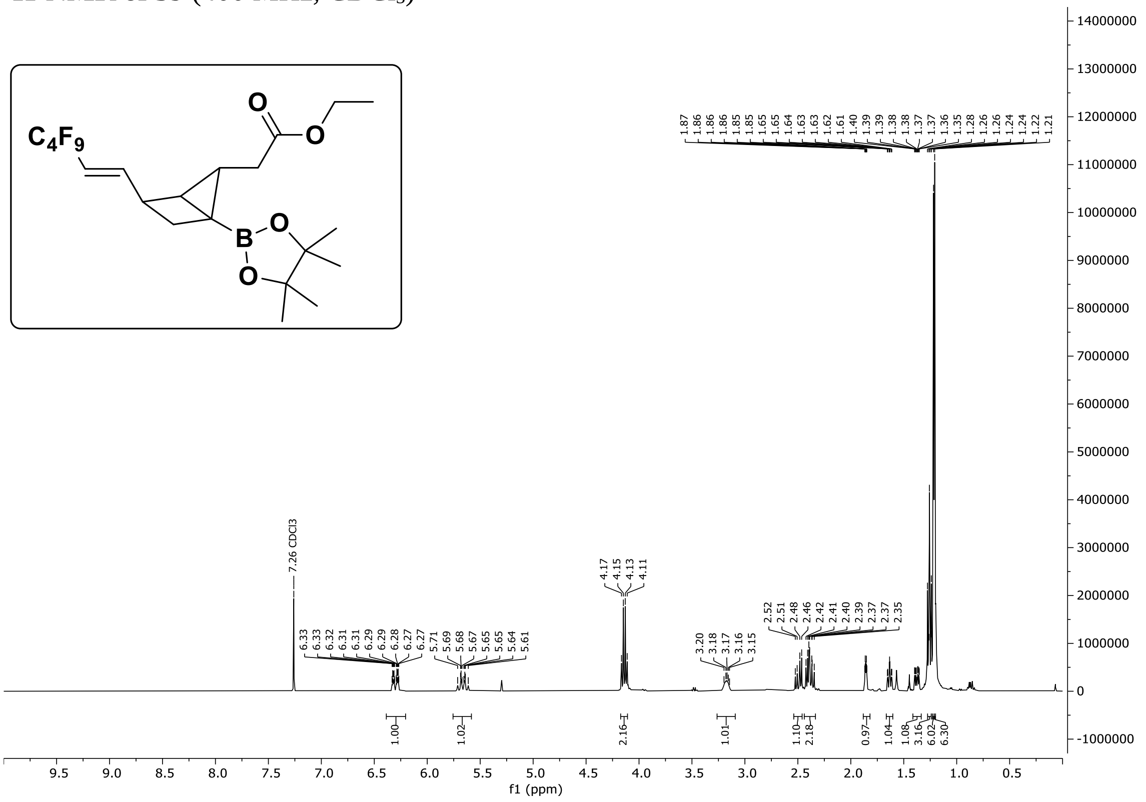

^{13}C-NMR of 59 (100 MHz, $CDCl_3$)

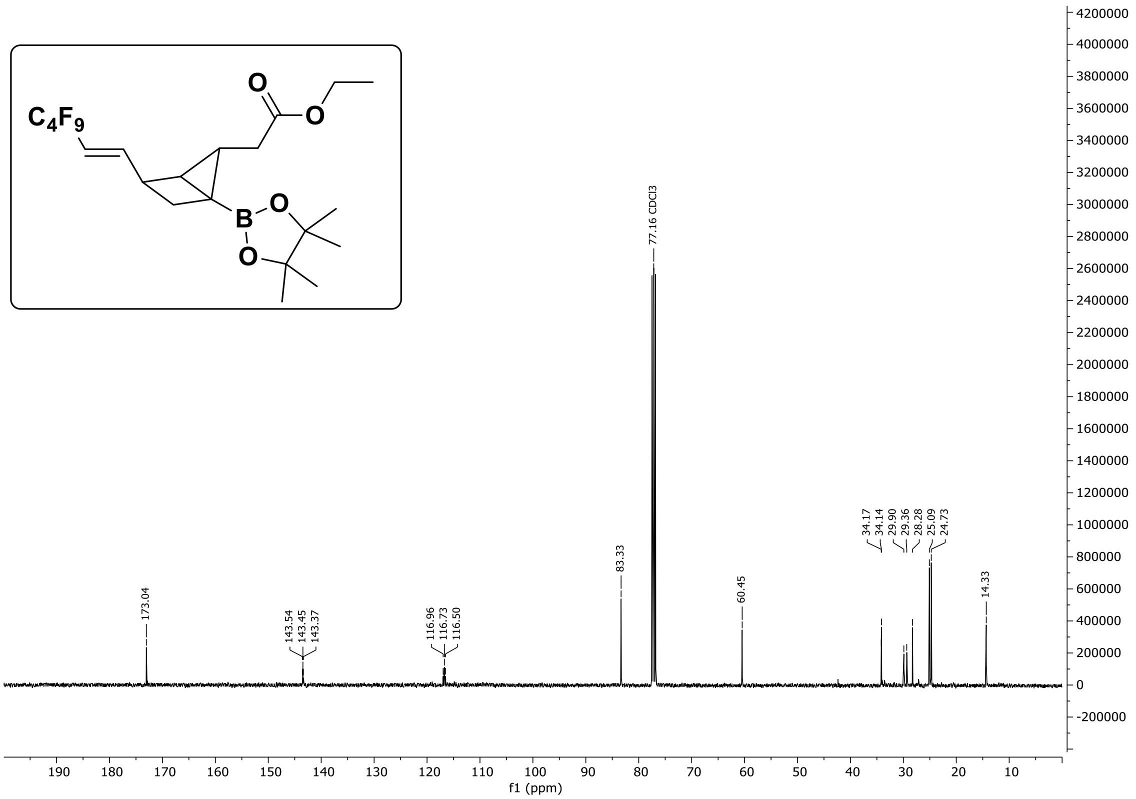

^{19}F-NMR of 59 (376 MHz, $CDCl_3$)

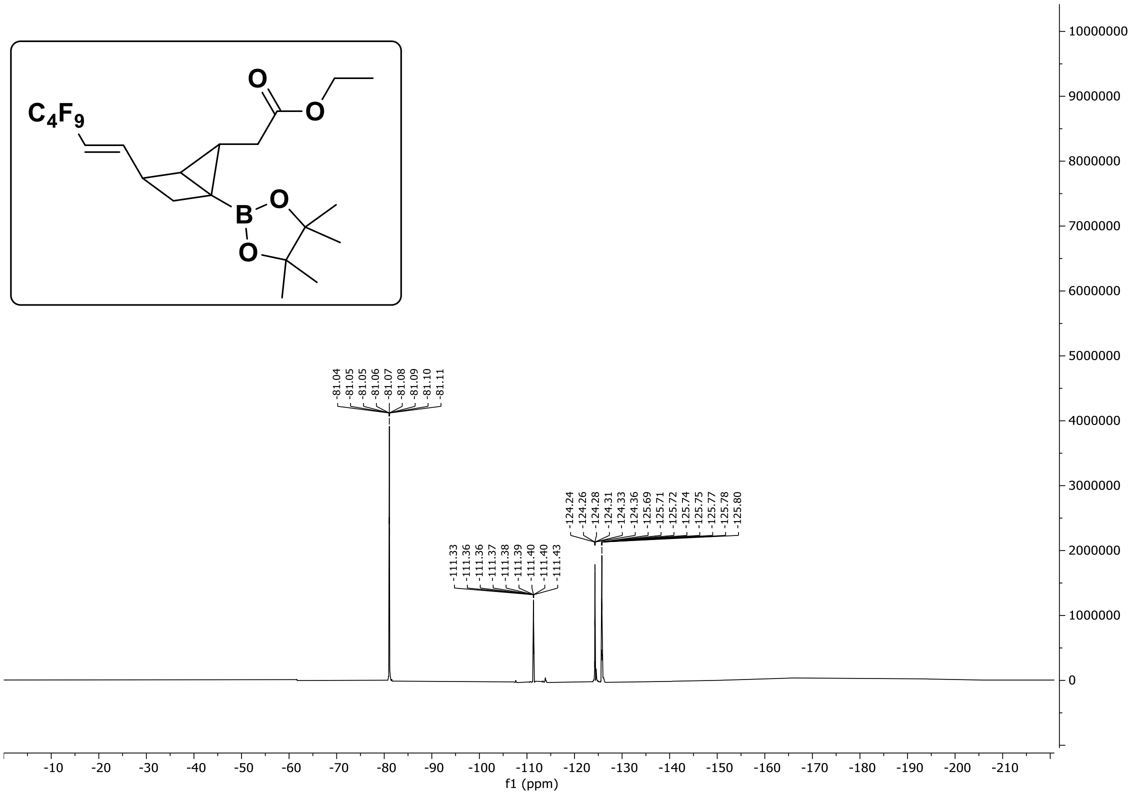

^{11}B NMR of 59 (128 MHz, $CDCl_3$)

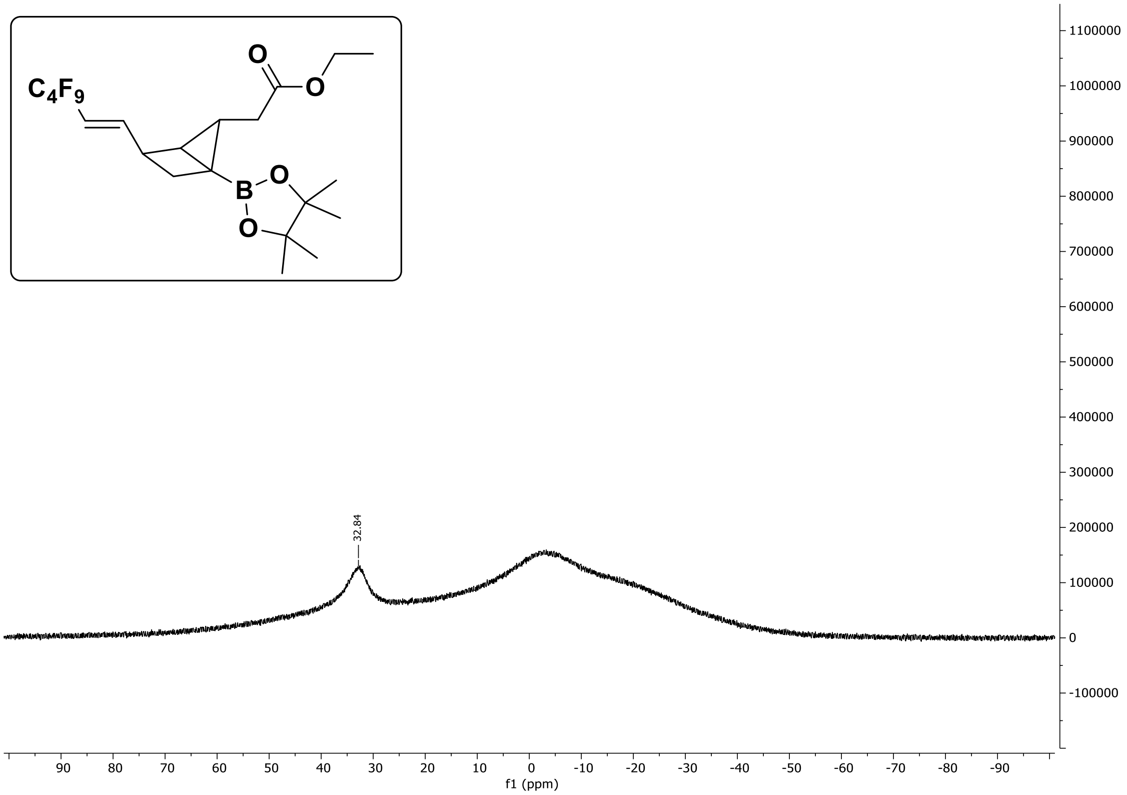

^{1}H-NMR of 61-major (600 MHz, $CDCl_3$)

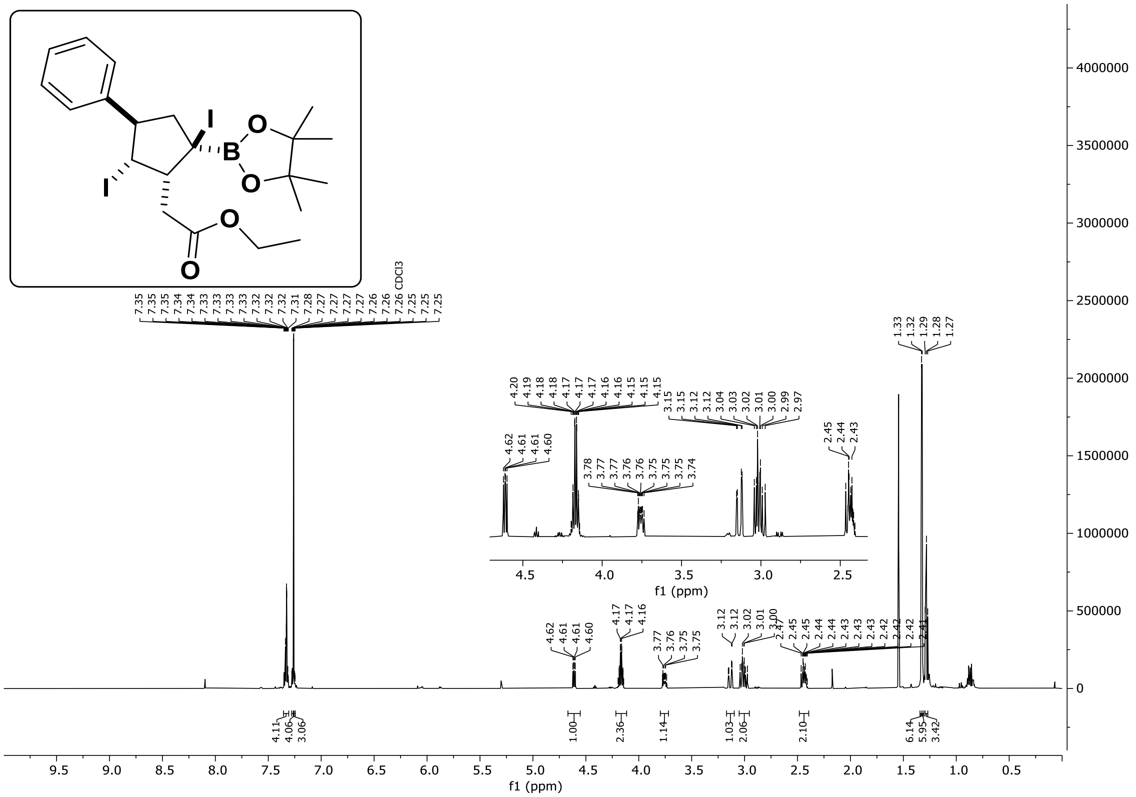

^{13}C-NMR of 61-major (151 MHz, $CDCl_3$)

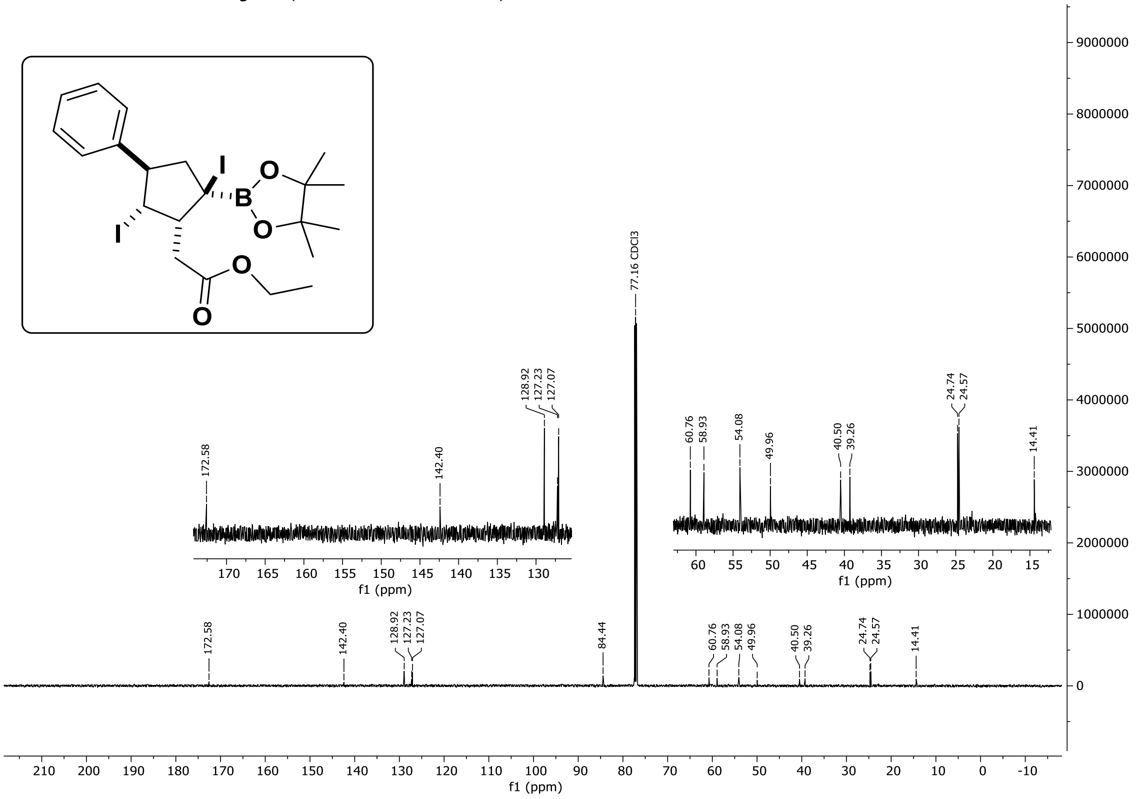

^{11}B NMR of 61-major (192 MHz, $CDCl_3$)

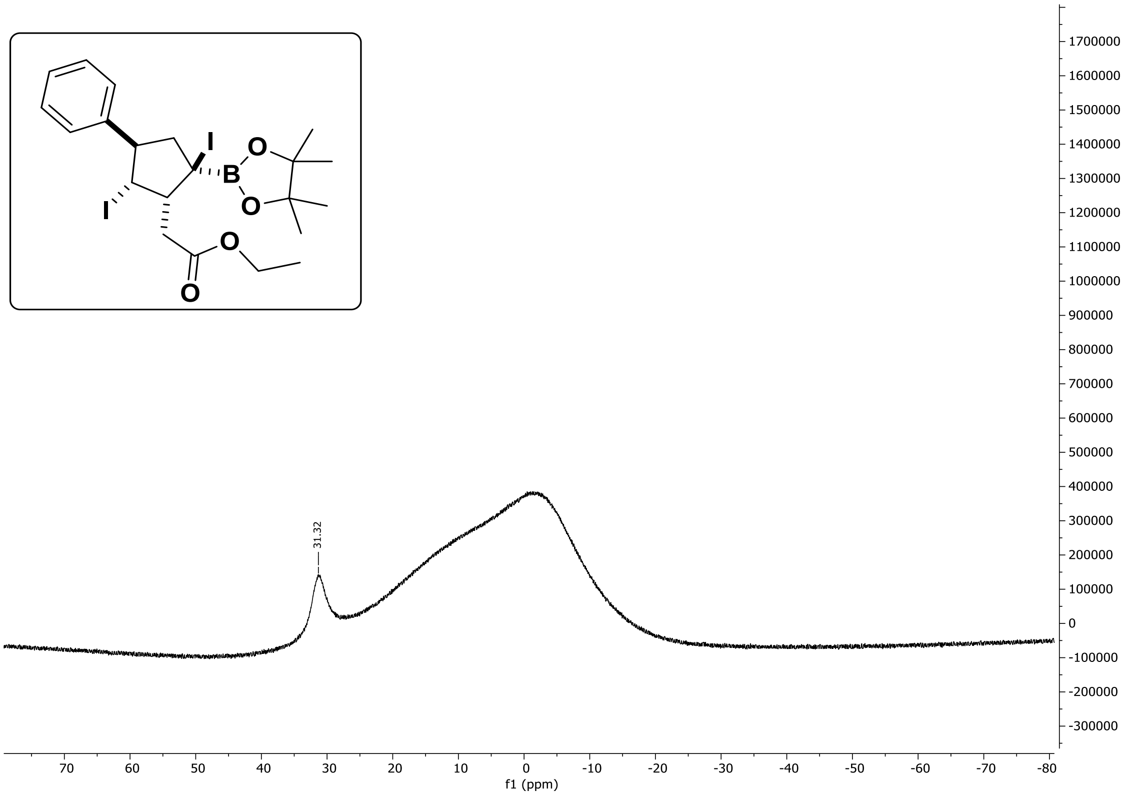

NOESY:

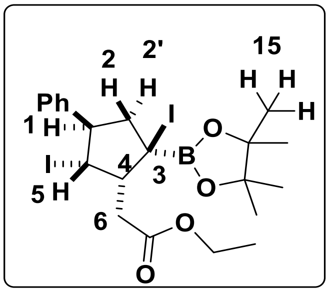

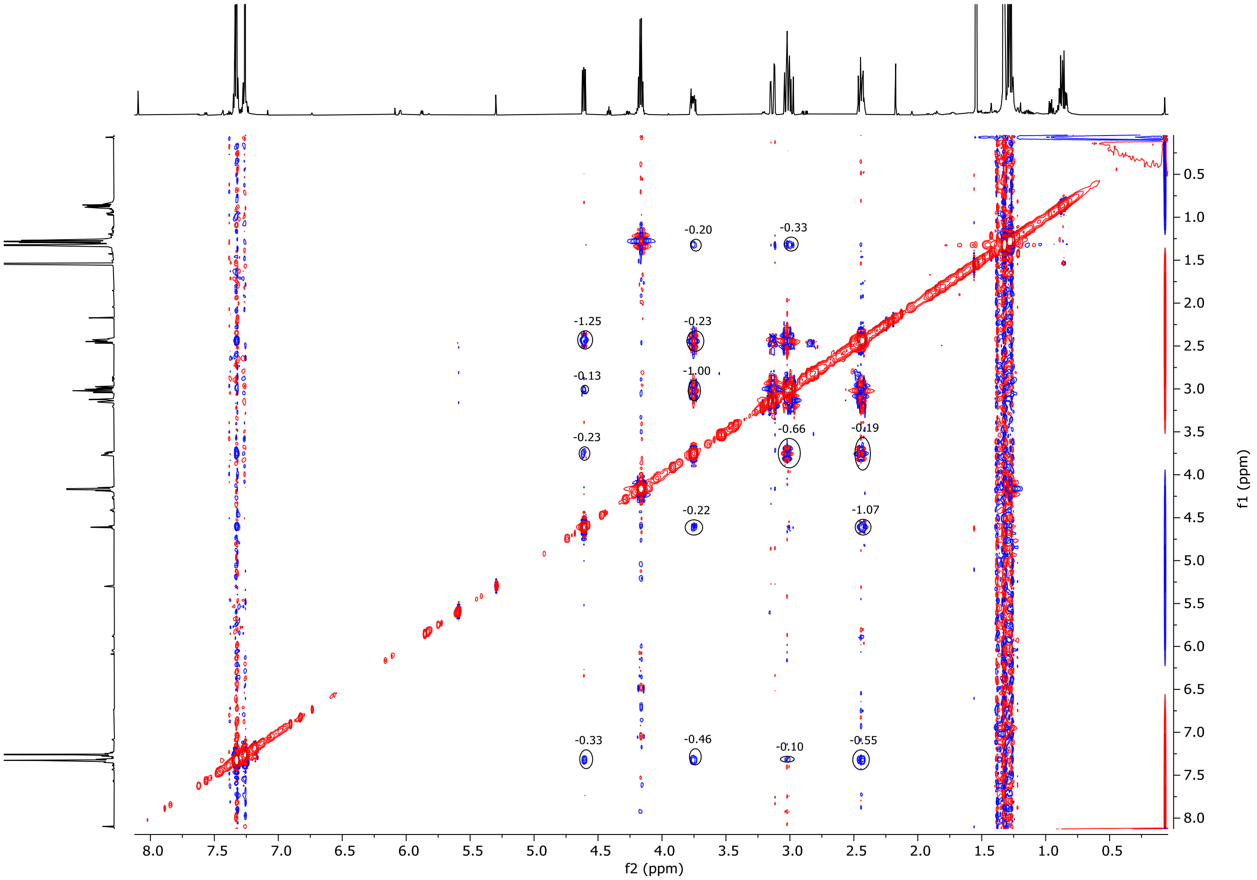

H1 has good NOE with H2', but weak NOE with H2;

H5 has no NOE with H2'; but good NOE with H2;

H1 and H2' have NOE with H15; H5 and H2 have no NOE with H15.

^{1}H-NMR of 61-minor (600 MHz, $CDCl_3$)

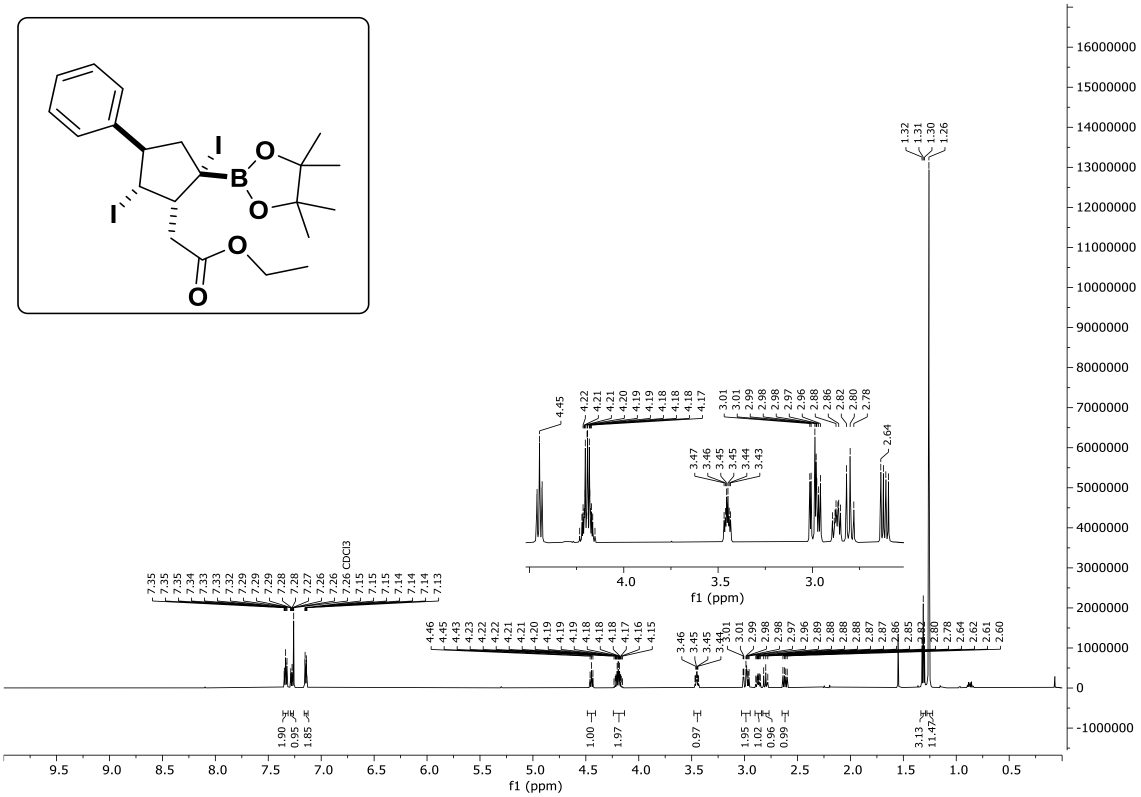

^{13}C-NMR of 61-minor (151 MHz, $CDCl_3$)

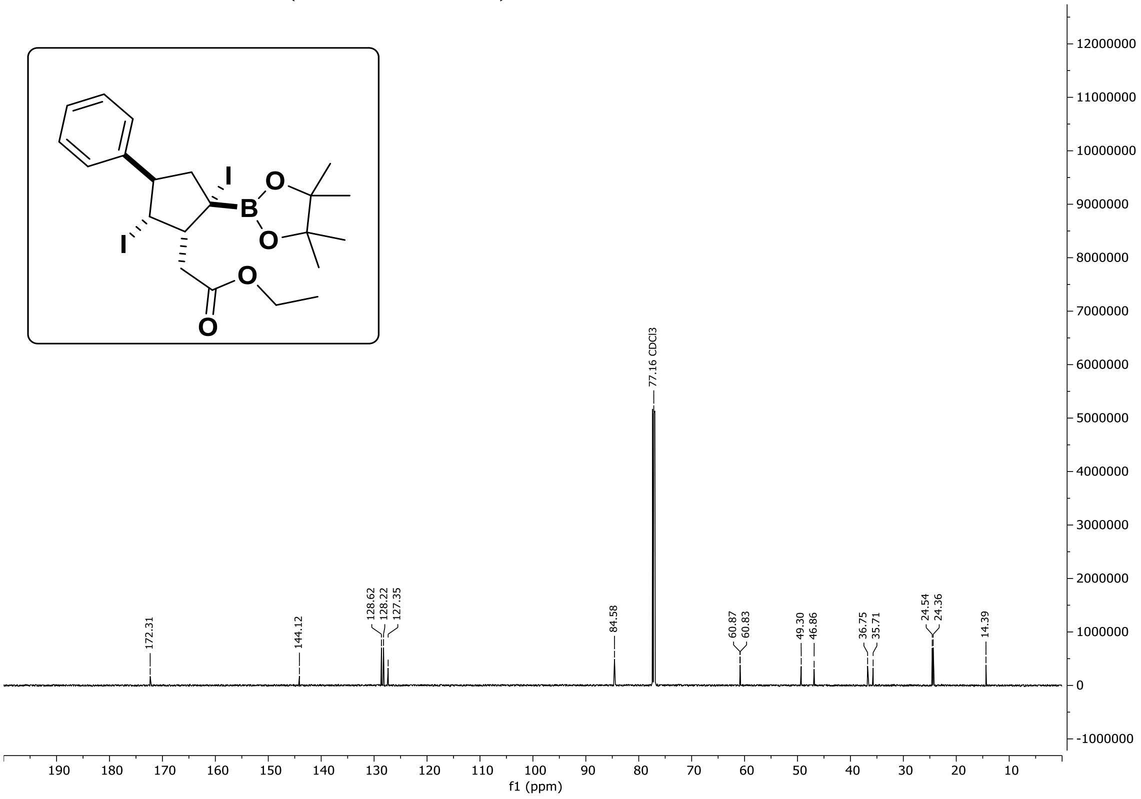

^{11}B NMR of 61-minor (192 MHz, $CDCl_3$)

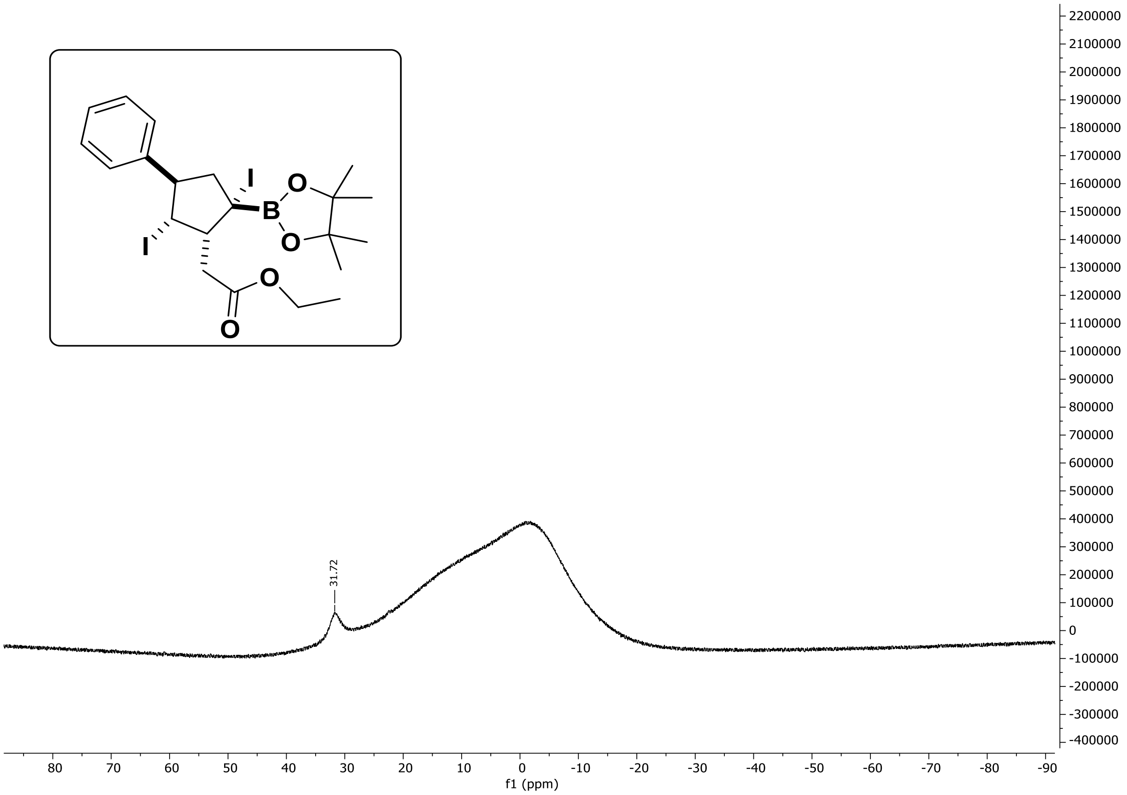

NOESY:

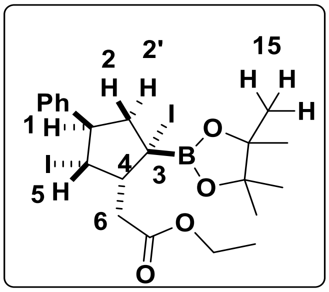

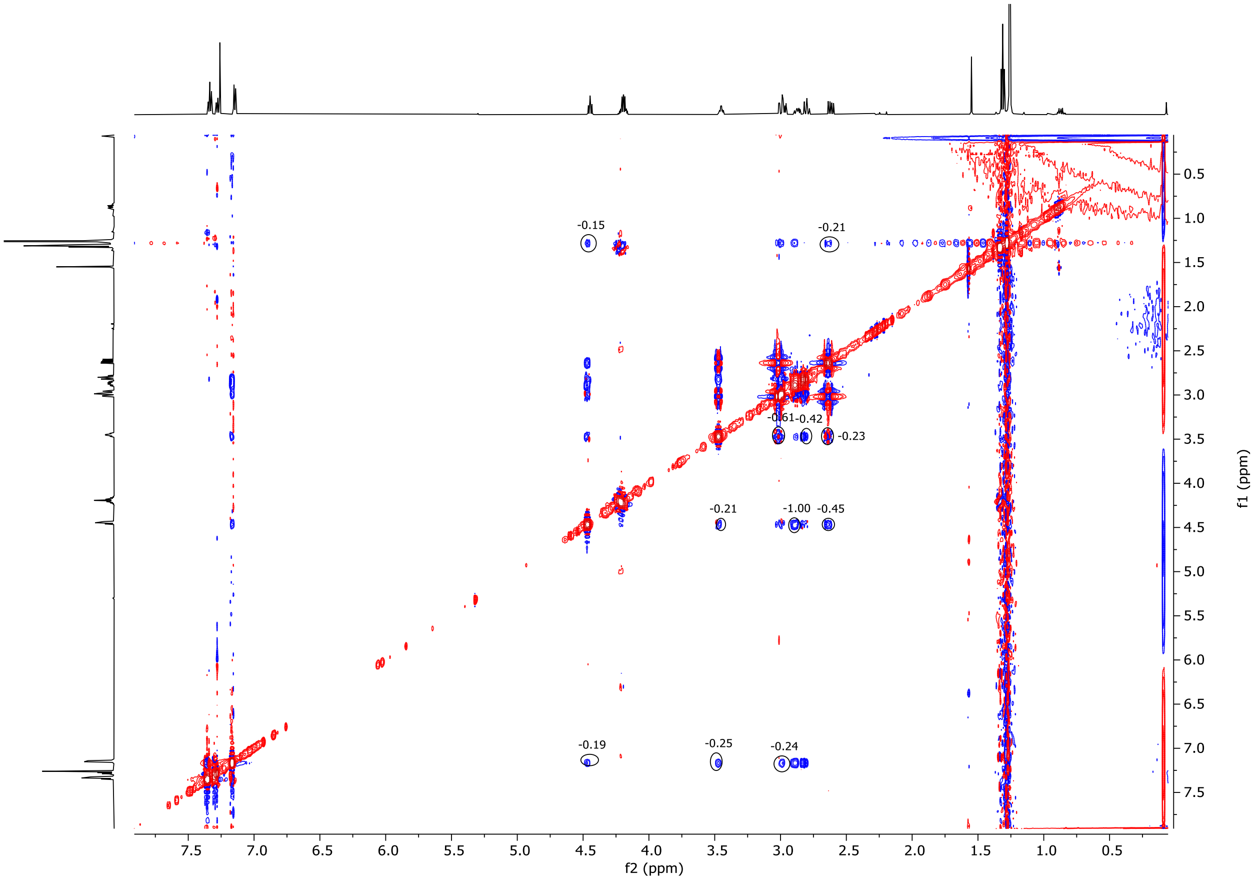

H1 has good NOE with H2', but weak NOE with H2;

H5 has no NOE with H2'; but good NOE with H2;

H1 has no NOE with H15; H5 and H2 have NOE with H15.

^{1}H-NMR of 62 (400 MHz, $CDCl_3$)

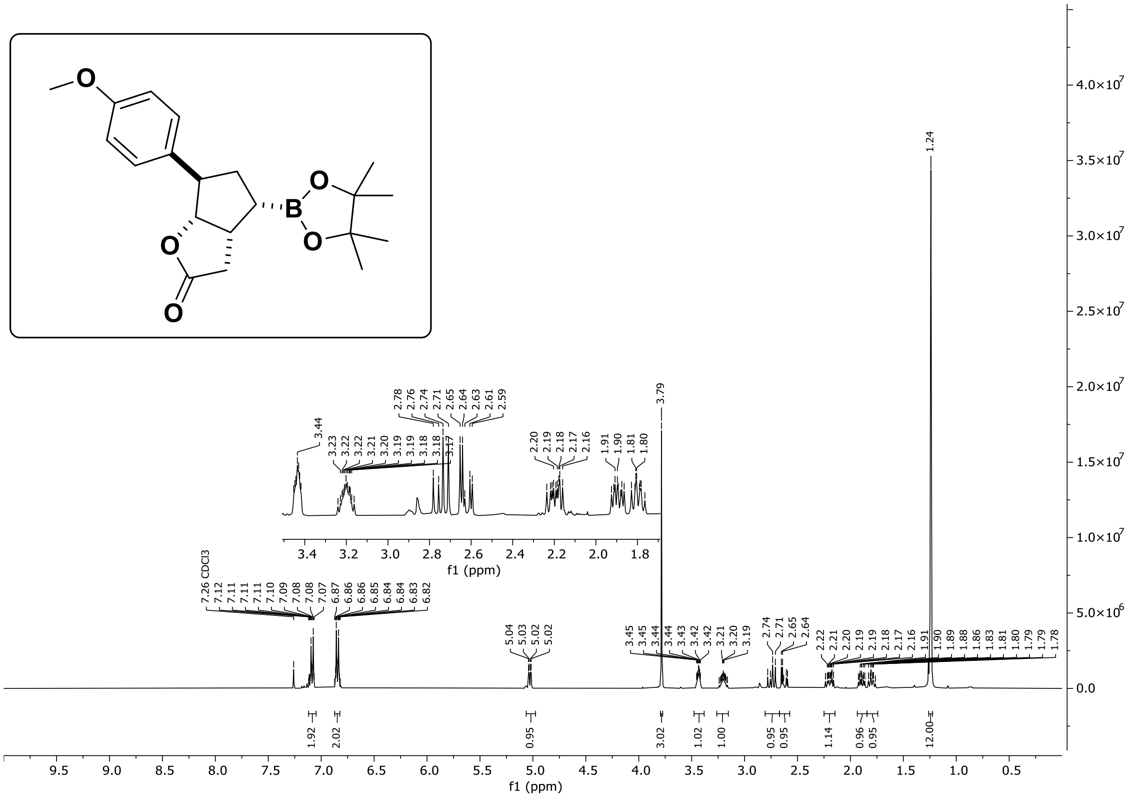

^{13}C-NMR of 62 (100 MHz, $CDCl_3$)

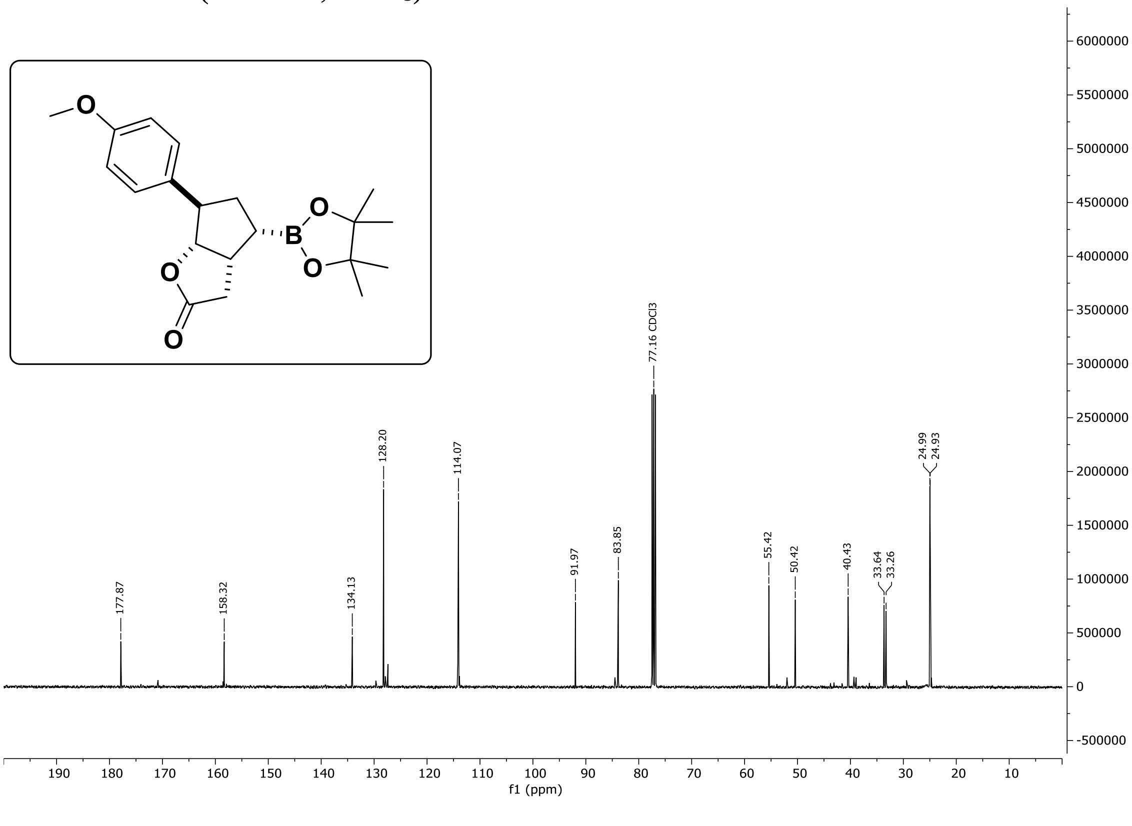

^{11}B NMR of 62 (128 MHz, $CDCl_3$)

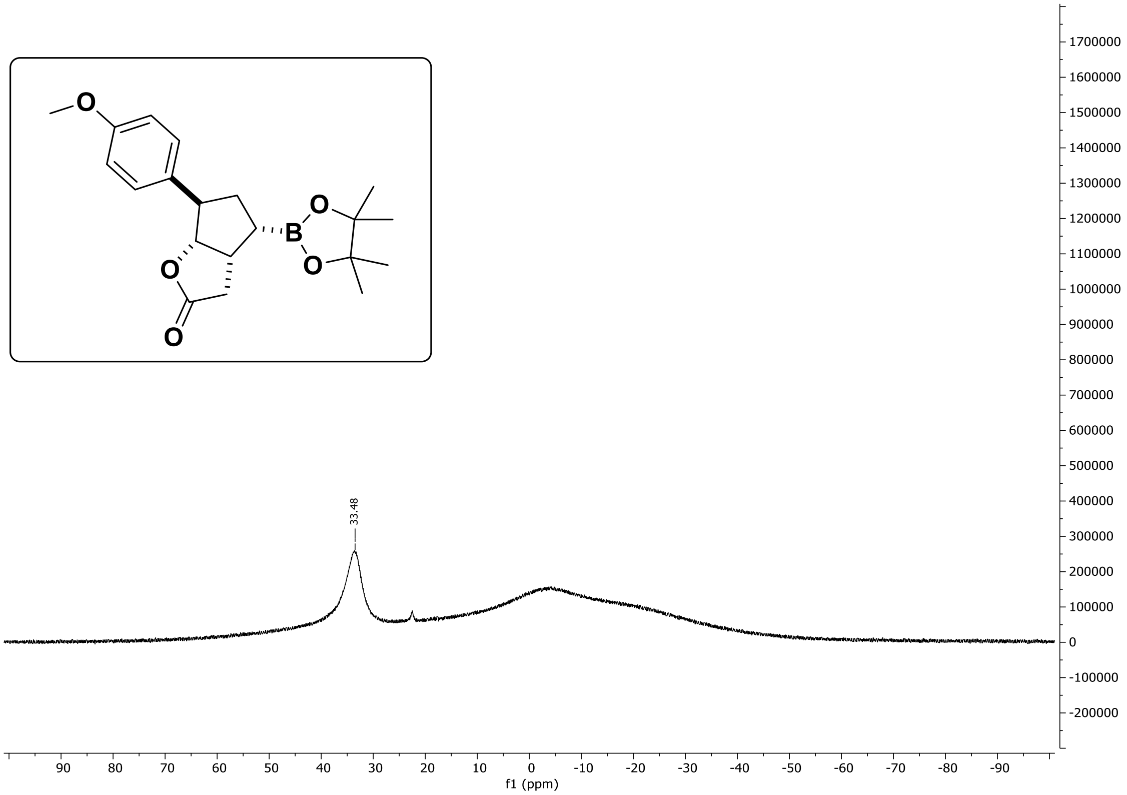

NOESY of 62:

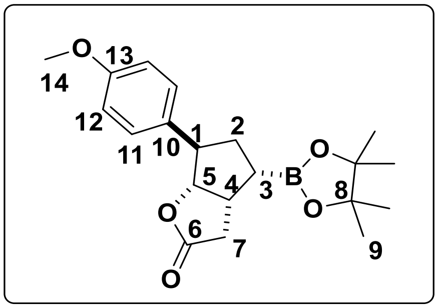

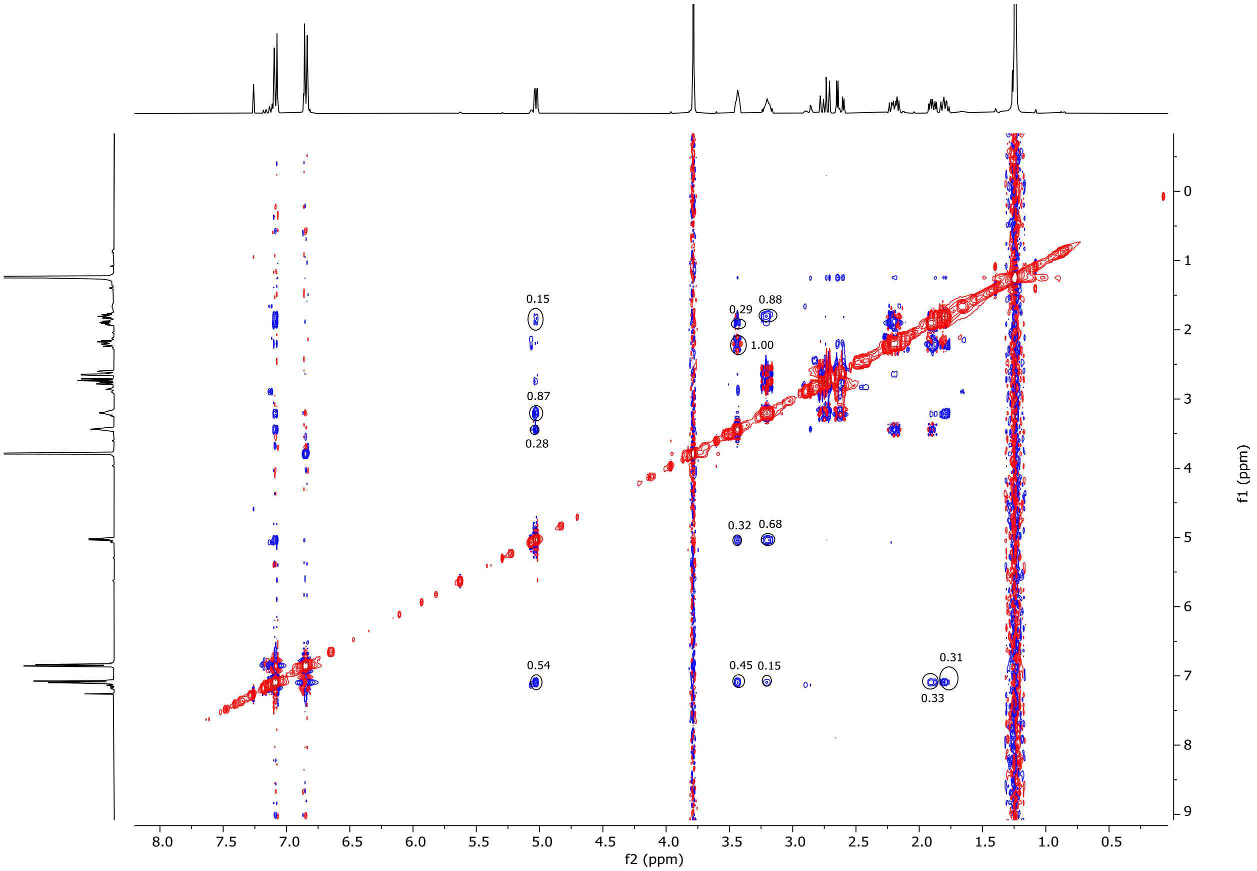

H5 has NOE with H4.

H4 has NOE with H3.

H3 and H4 have NOE with H11, which means H3, H4 and Ar are at same face.

^{1}H-NMR of S31 (400 MHz, $CDCl_3$)

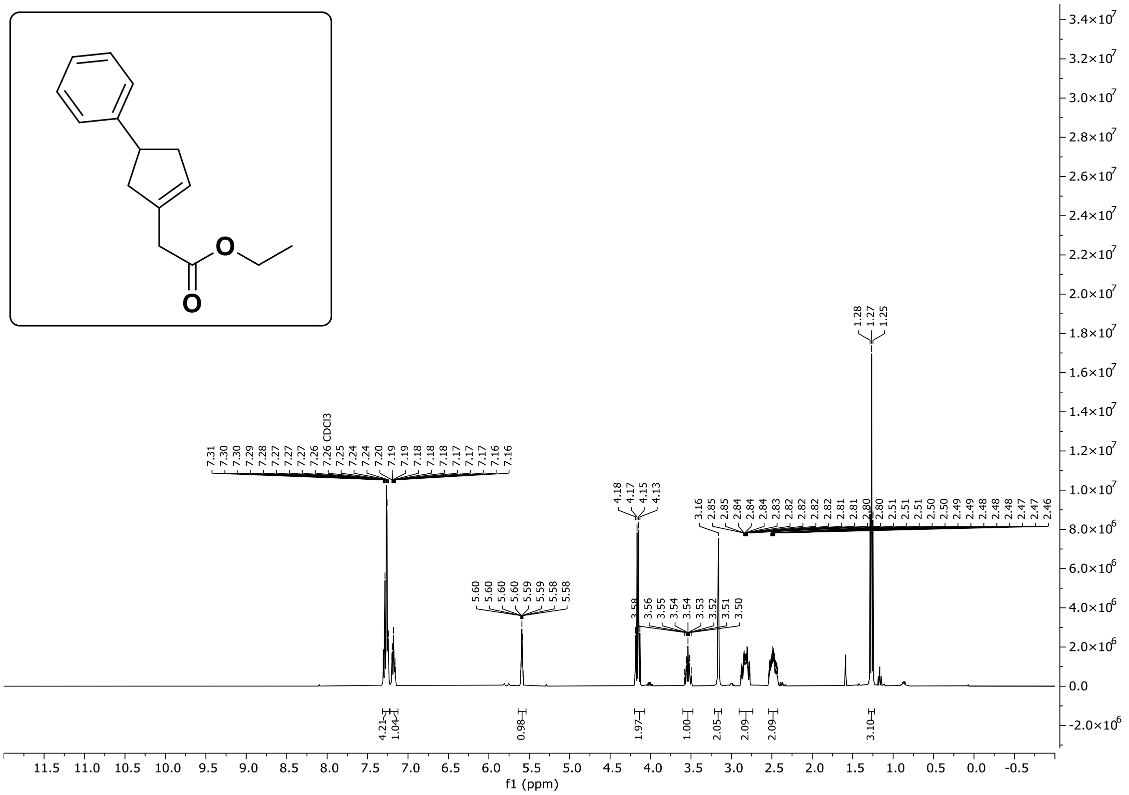

^{13}C-NMR of S31 (100 MHz, $CDCl_3$)

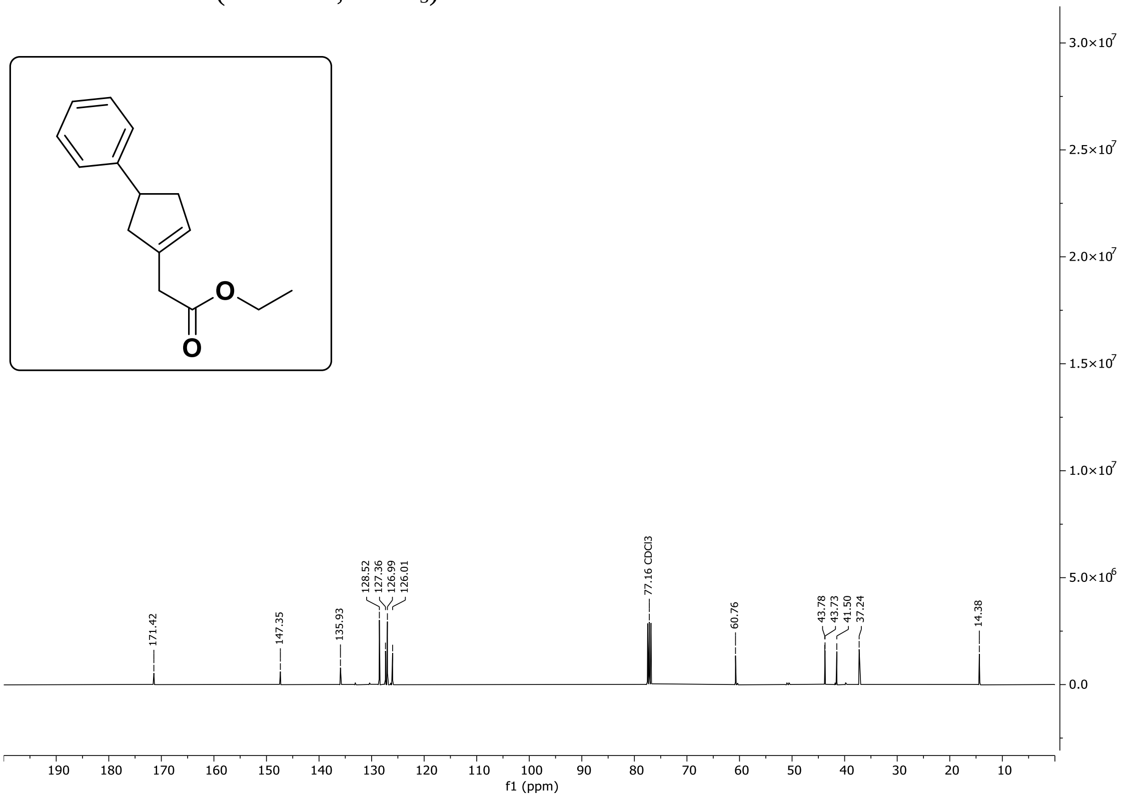

^{1}H-NMR of S32 (600 MHz, $CDCl_3$)

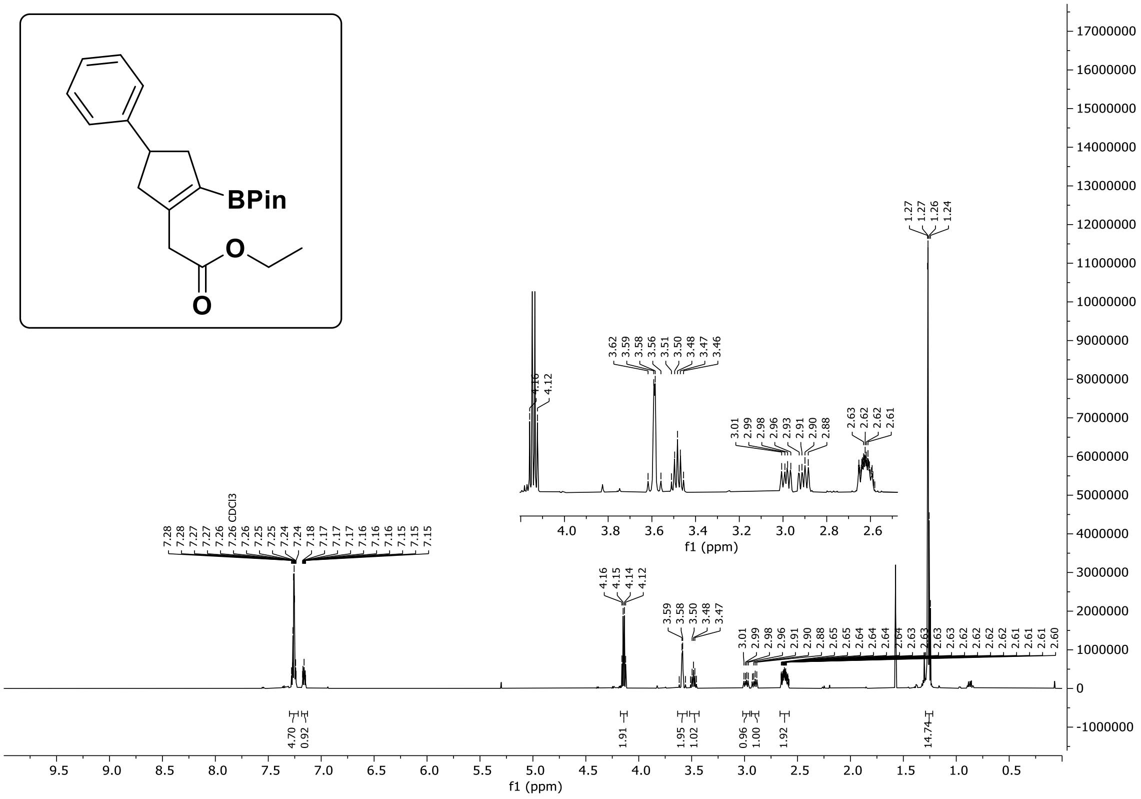

^{13}C-NMR of S32 (151 MHz, $CDCl_3$)

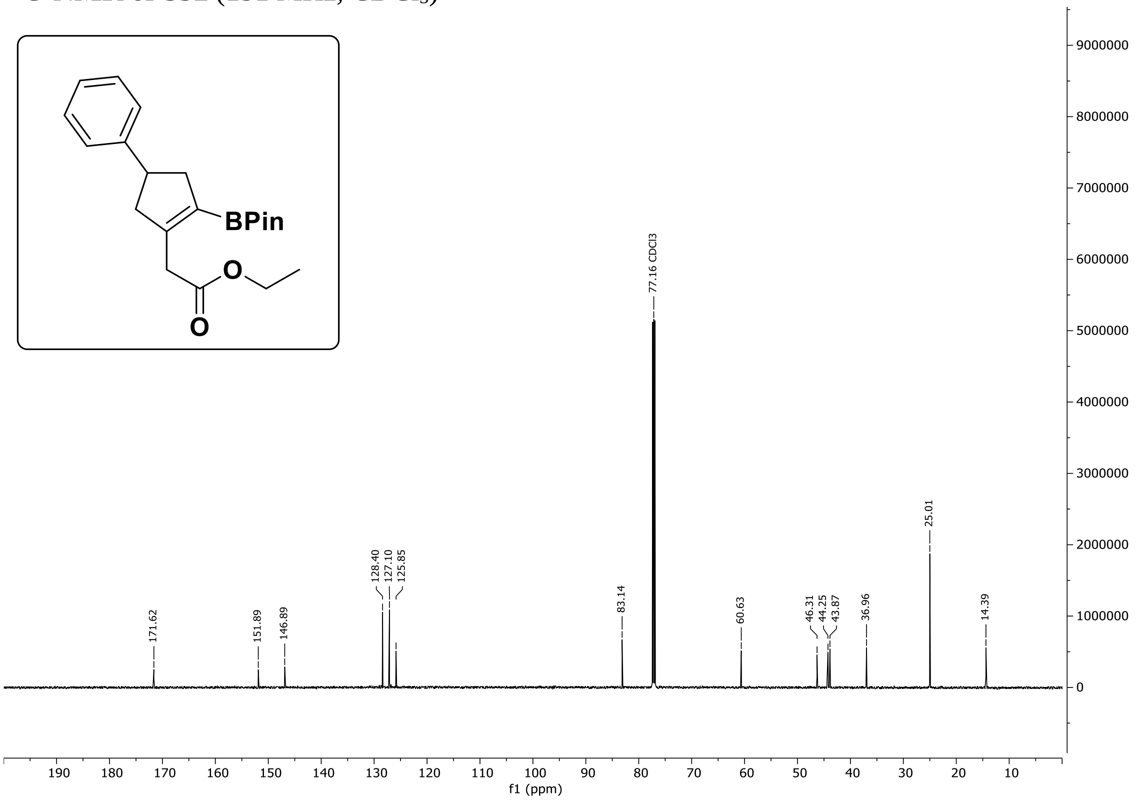

^{11}B NMR of S32 (128 MHz, $CDCl_3$)

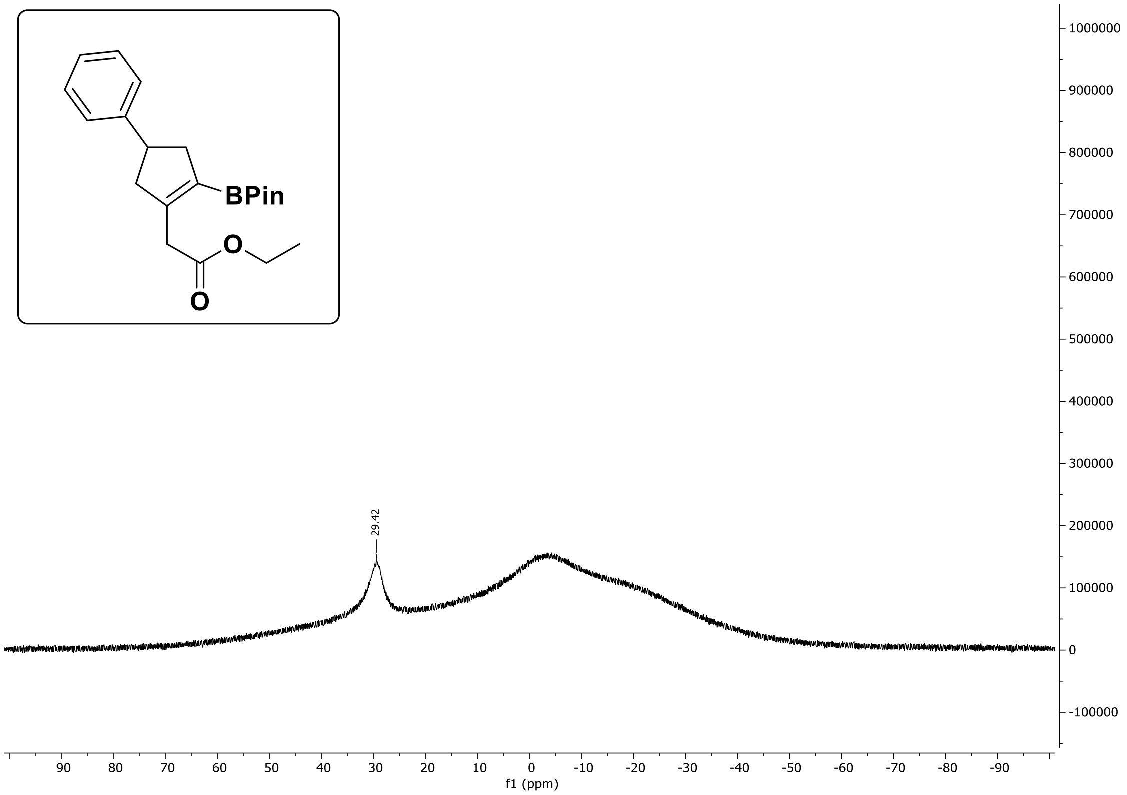

Supplement: Supplementary file 1 [file ja5c17624_si_001.pdf]
